# Combined Kinetic and Computational Analysis of the Palladium-Catalyzed Formylation of Aryl Bromides

**DOI:** 10.1021/acscatal.4c05324

**Published:** 2024-12-18

**Authors:** Georgina Rai, Lee J. Edwards, Rebecca L. Greenaway, Philip W. Miller, Katherine M. P. Wheelhouse, Mark R. Crimmin

**Affiliations:** † Molecular Sciences Research Hub, Imperial College London, 82 Wood Lane, Shepherds Bush, London W12 0BZ, U.K.; ‡ GSK Medicines Research Centre, GSK, Gunnels Wood Road, Stevenage, Hertfordshire SG1 2NY, U.K.

**Keywords:** palladium catalysis, formylation, kinetics, mechanistic study, automation

## Abstract

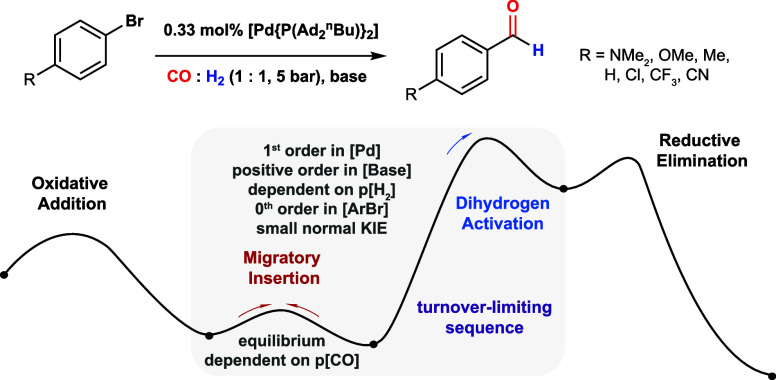

Aryl aldehydes are key synthetic intermediates in the manufacturing of active pharmaceutical ingredients. They are generated on scale (>1000 kg) through the palladium-catalyzed formylation of aryl bromides using syngas (CO/H_2_). The best-in-class catalyst system for this reaction employs di-1-adamantyl-*n*-butylphosphine (cata*CX*ium A), palladium(II) acetate, and tetramethylethylenediamine. Despite nearly 20 years since its initial report, a mechanistic understanding of this system remains incomplete. Here, we use automation, kinetic analysis, and DFT calculations to develop a mechanistic model for this best-in-class catalyst. We suggest that a combination of the migratory insertion step and dihydrogen activation step is likely involved in the turnover-limiting sequence. The reaction kinetics are responsive to the nature of the substrate, with electron-rich aryl bromides reacting faster and more selectively than their electron-poor counterparts due to the influence of electronics in the migratory insertion step. Our findings add additional insight into the proposed mechanism of palladium-catalyzed formylation of aryl bromides.

## Introduction

**Figure 1:**
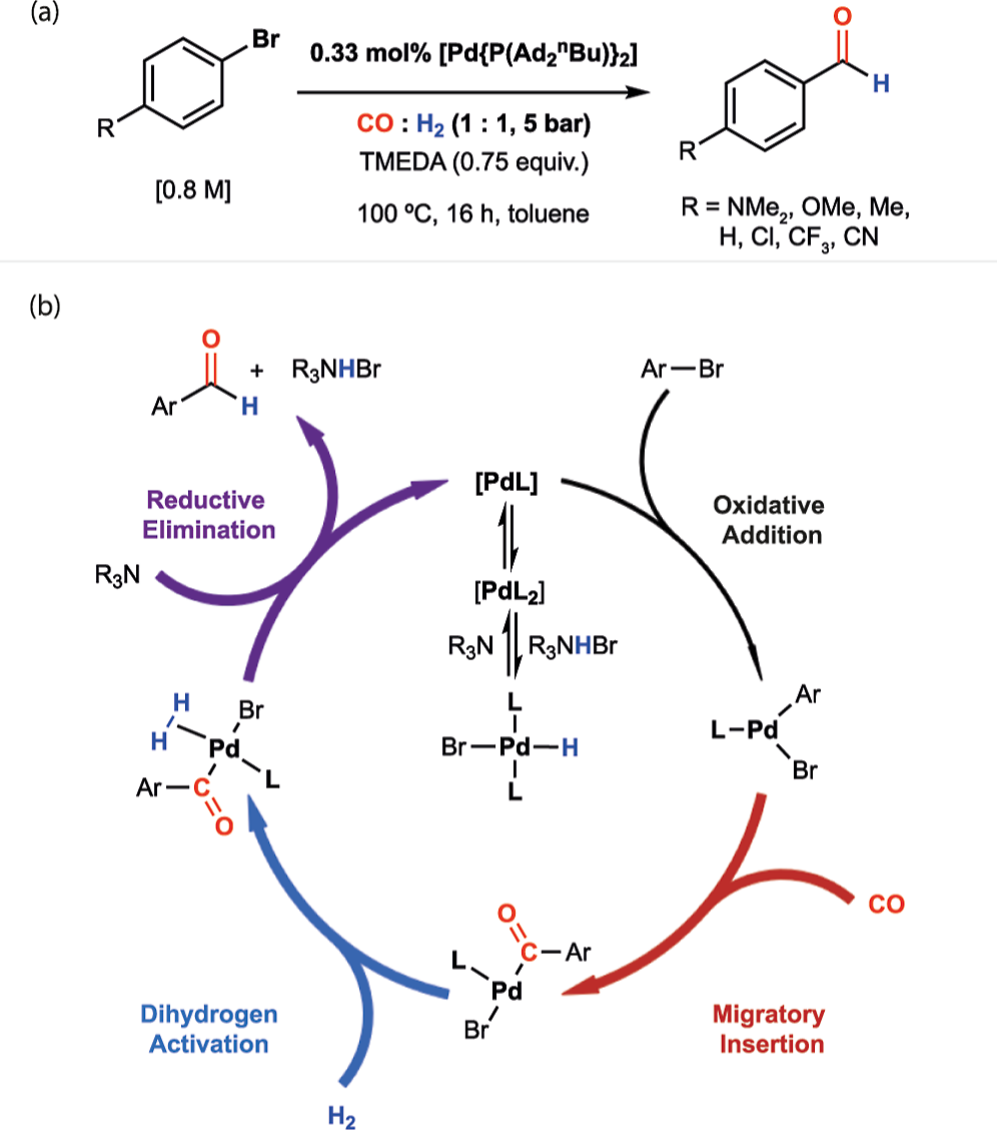
(a) Pd-catalyzed formylation of aryl bromides by CO/H_2_. (b) Proposed simplified mechanism based on current understanding (off-cycle resting states including dimeric Pd complexes and Pd carbonyl clusters not represented). L = PAd_2_^*n*^Bu.

The palladium-catalyzed formylation of aryl halides using mixtures of CO and H_2_ (syngas) has proven to be an enabling technology in the chemical manufacture of aromatic aldehydes.^1−9^ Production of these compounds on scale is essential due to their role as intermediates in the fine chemical sector.^10−13^ One of the most efficient systems for aryl bromide formylation reported to date employs a catalytic mixture of di-1-adamantyl-*n*-butylphosphine (PAd_2_^*n*^Bu, cata*CX*ium A) and palladium(II) acetate, along with substoichiometric quantities of a tetramethylethylenediamine (TMEDA) base (Figure 1a). The reaction operates at 5 bar of CO/H_2_ pressure and 100 °C and has been applied to the industrial manufacture of a pharmaceutical intermediate on a multi-1000 kg scale.^9,14,15^ The approach provides a much-needed alternative to the Bouveault aldehyde synthesis—a procedure that relies on in situ generation of organomagnesium reagents and has a low atom economy and functional group tolerance.^16−18^

The current mechanistic understanding of the palladium-catalyzed formylation of aryl halides using mixtures of CO and H_2_ derives from evaluating the reactivity of well-defined palladium complexes.^19^ The oxidative addition, migratory insertion, and reductive elimination steps all have experimental support (Figure 1b). The active catalyst is proposed to be monomeric, with dimeric species and palladium carbonyl clusters proposed to be off-cycle resting states that act as reservoirs for on-cycle species. It has been suggested that the palladium hydride bromide complex *trans*-[Pd(H)(Br)(PAd_2_^*n*^Bu)_2_] is an important off-cycle resting state. A combination of base-mediated reformation of [Pd(PAd_2_^*n*^Bu)_2_] from this complex, along with oxidative addition of the aryl bromide to [Pd(PAd_2_^*n*^Bu)_2_], is proposed to be the turnover-limiting step of catalysis.^19^ Despite the huge importance of this system, there are clear limitations with our understanding: little is known about the mechanistic step(s) that involve dihydrogen splitting; it is not clear how the proposed mechanism responds to changes in the aryl bromide substrate or partial pressure of gases; and robust information regarding catalyst activation and deactivation pathways is lacking.

Kinetic analysis would potentially provide information to address these questions. Although several studies have been published on the kinetic analysis of catalytic carbonylative processes,^20−22^ data collection can be challenging. Reactions involving gas–liquid interfaces are typically conducted in batch pressure reactors, where repeated sampling required for offline kinetic analysis has the potential to affect pressures and gas–liquid ratios. While in situ monitoring techniques circumvent this problem, they require bespoke reactor setups and do not address another key issue: the need to run large numbers of experiments to form a complete mechanistic picture (e.g., Hammett analysis, orders in catalyst, orders in reactants, order in base, Eyring analysis, and kinetic isotope effects (KIEs)). Automated data collection and parallel experimentation offer the potential to address these challenges and not only increase productivity in kinetic data collection but also enhance data integrity and reproducibility through the use of robotics.^23−25^

In this article, we report a detailed kinetic analysis of the best-in-class catalyst system for the formylation of aryl bromides. We use automation to accelerate kinetic analysis, allowing insight into the effects of catalyst concentration, reagent concentration, pressure, gas ratio, and temperature across a range of substrates. The kinetic analysis suggests that the turnover-limiting sequence involves a combination of a reversible migratory insertion step and a dihydrogen activation step. Kinetics were sensitive to the electronics of the substrate with two regimes observed for electron-rich (σ_p_ < 0) and electron-poor aryl bromides (σ_p_ > 0). DFT calculations were used in combination with kinetics to create a detailed mechanistic model for palladium-catalyzed formylation. This model predicts divergent behavior of electron-rich and electron-poor aryl bromides due to the influence of substrate electronics in the migratory insertion step.

## Results and Discussion

### Kinetic Data

#### Automated Workflow

**Figure 2:**
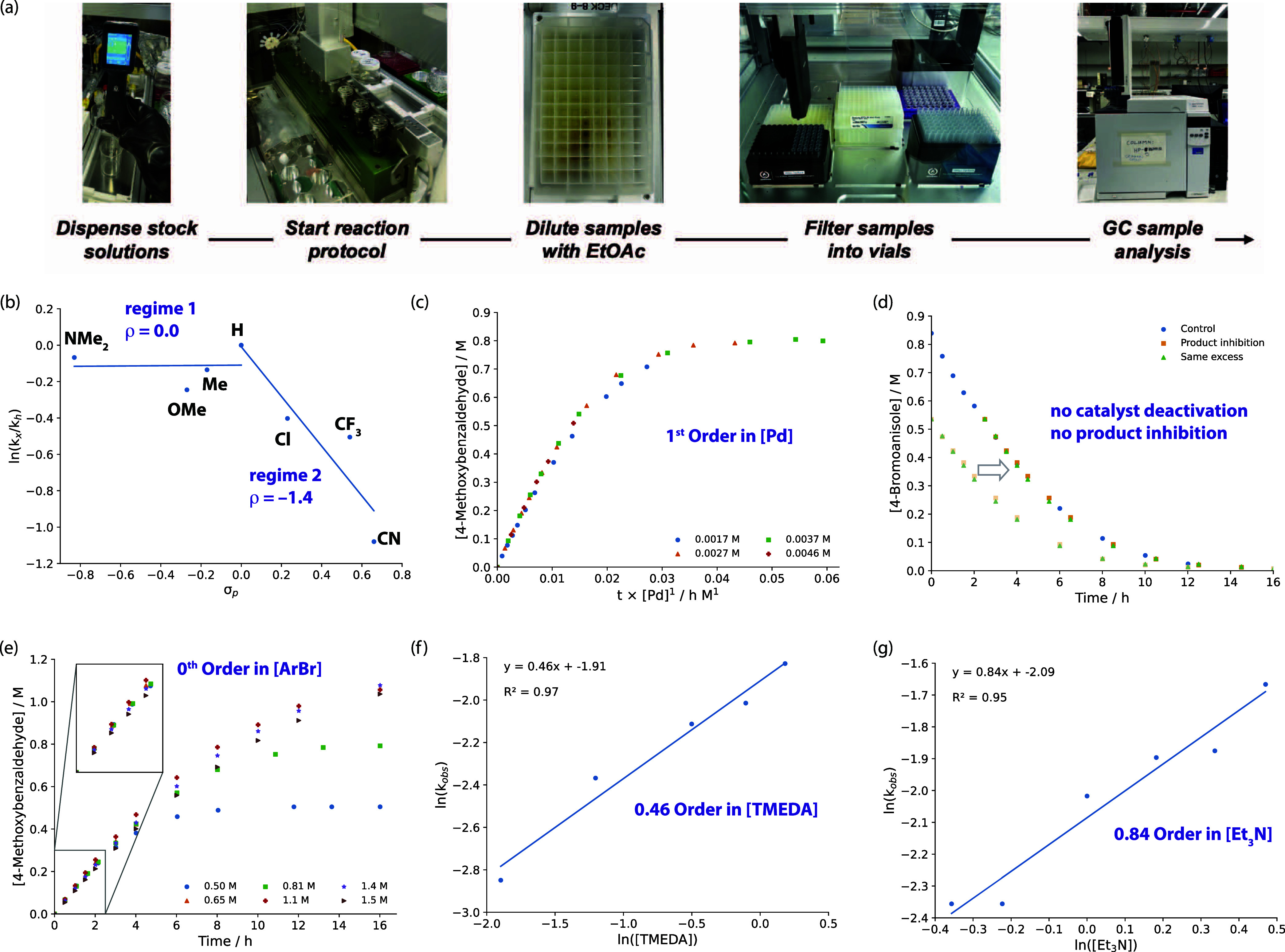
(a) Automated workflow used for collection of kinetic data, along with plots showing (b) Hammett plot of ln *k*_H_/ln *k*_X_ against σ_p_ for a series of 4-substituted aryl bromides. (c) VTNA plot showing 1st-order behavior in [Pd(PAd_2_^*n*^Bu)_2_] for formylation of 4-bromoanisole. (d) Overlay of concentration–time profile for 4-bromoanisole under standard conditions along with same-excess and product inhibition profiles. (e) VTNA plot showing 0th-order behavior in [ArBr] for formylation of 4-bromoanisole. (f) ln–ln plot showing partial order in TMEDA for formylation of 4-bromoanisole. (g) ln–ln plot showing partial order in Et_3_N for formylation of 4-bromoanisole.

The reaction of model substrate 4-bromoanisole (0.8 M in toluene) with a 1:1 mixture of CO/H_2_ catalyzed by 0.33 mol % [Pd(PAd_2_^*n*^Bu)_2_]^26^ was conducted at 100 °C under constant 5 bar pressure. We employed an Unchained Laboratories optimization sampling reactor (OSR) to automate sample collection and accelerate investigation of variables through parallel experimentation. The OSR can aliquot from eight parallel reactors with independent pressure and temperature control.^27,28^ Automated reaction sampling was combined with a streamlined approach to work up each aliquot using an Opentrons OT-2 liquid handling platform for an automated filtration step. Subsequent data analysis was carried out using reaction progress kinetic analysis (RPKA) or variable time normalization analysis (VTNA).^29−34^ The combined workflow allowed for an approximate 10-fold reduction of attended hours for experimentation compared to established nonautomated approaches (Figure 2a). Reaction rates of 4-bromoanisole formylation were reproducible across all eight individual reactors. Initial rates of formylation did not show a dependence on the stirrer rate (500 vs 800 rpm), suggesting that mass transfer of the gas is not limiting in this system.

#### Hammett Analysis

To gain an understanding of how the reaction responds to changes in the electronics of the aryl bromide, a Hammett analysis was carried out. Reaction rates acquired from separate kinetic runs for each substrate were compared. A plot of ln(*k*_X_/*k*_H_) vs σ_p_ does not fit a simple linear trend but rather shows an inflection point, suggestive of two different kinetic regimes (Figure 2b). For electron-donating substituents (σ_p_ < 0), the data can be fitted with ρ of zero. However, for more electron-poor substituents (σ_p_ > 0), a negative slope with ρ = −1.4 is observed. The data are consistent with a change in the turnover-limiting sequence as the electronics of the substrate are varied. Prior work examined the role of substrate electronics in the formylation of aryl bromides with this catalytic system. Based on competition experiments, it has been suggested that more electron-deficient substrates react faster, with Hammett data fitting ρ = +1.0.^14^ There are known issues using competition experiments to extract kinetic information.^35^ In this case, the Hammett data likely provide insight into a selectivity event rather than a turnover-limiting event (vide infra). Based on our observations, further kinetic analysis was conducted with both 4-bromoanisole (σ_p_ = −0.27) and 4-bromobenzotrifluoride (σ_p_ = +0.54) to ensure that the behavior of both kinetic regimes was captured.

#### Orders in Catalyst and Catalyst Deactivation

Using the optimized workflow, the orders in the catalyst and reagent were investigated. Variation of the concentration of [Pd(PAd_2_^*n*^Bu)_2_] across a 1.7 to 4.6 mM concentration range was investigated for the formylation of 0.8 M solutions of 4-bromoanisole in toluene at 100 °C, 5 bar of 1:1 CO/H_2_, and 0.75 equiv TMEDA. No induction period is observed in the kinetic profiles. The lack of an induction period suggests that [Pd(PAd_2_^*n*^Bu)_2_] is either an on-cycle species or undergoes a fast event, such as ligand dissociation, to generate an on-cycle species. VTNA shows a clear fit to first-order behavior in the catalyst (Figure 2c). The kinetic profiling was repeated with 4-bromobenzotrifluoride, and data again fit first order in catalyst. Using RPKA, a same-excess experiment with 4-bromoanisole was conducted to explore the possibility of catalyst deactivation. Time-shifted kinetic profiles show good overlap with the standard conditions, suggesting that catalyst deactivation is not significant under the conditions of the OSR kinetic runs (Figure 2d).

#### Orders in Substrate and Product Inhibition

The order in 4-bromoanisole was investigated for six different concentrations across a 0.5 to 1.5 M range. VTNA is consistent with a zero-order fit (Figure 2e). For 0.5 and 0.81 M kinetic runs, the profiles deviate from the fit as the reaction progresses due to full consumption of the substrate when present at lower concentrations. Kinetic runs using 4-bromobenzotrifluoride using four different concentrations across a 0.5 to 1.5 M range also fit zero-order behavior in the substrate.^36^ The possibility of product inhibition was investigated for 4-bromoanisole through spiking of reaction mixtures with the corresponding aldehyde and TMEDA·2HBr and comparison against a control sample. Time-shifted kinetic profiles again provide excellent overlap, suggesting that inhibition by either the product or amine-salt byproduct does not occur in this system (Figure 2d).

#### Order in Base

Order in base was investigated using either TMEDA or Et_3_N. The former amine is dibasic, while the latter is monobasic. Both were investigated to deconvolute the order in reagent from order in active basic sites. For the reaction of 4-bromoanisole, TMEDA initial concentrations were varied from 0.15 to 1.2 M, and initial rate data were collected. Plotting ln *k*_obs_ vs ln[TMEDA] gave an order of 0.46 (Figure 2f). Similarly, kinetic analysis using 0.7 to 1.6 M initial concentrations of Et_3_N gave an order of 0.84 (Figure 2g). For 4-bromobenzotrifluoride, an order of 0.53 in TMEDA was acquired. Hence, for both electron-rich and electron-deficient substrates, experimental data support a near first order in basic sites.

#### Varying Gas Ratios

Further kinetic runs were conducted in which the partial pressure of H_2_ (*P*_H_2__) was reduced by half, keeping the partial pressure of CO (*P*_CO_) constant by using a N_2_ balance (CO/H_2_/N_2_ 1:0.5:0.5). Initial rates of aldehyde formation reduced significantly across a range of electron-rich and -poor substrates, highlighting a likely role of H_2_ pressure in the turnover-limiting sequence (Table 1). A similar experiment in which the partial pressure of CO (*P*_CO_) was reduced by half, keeping the partial pressure of H_2_ (*P*_H_2__) constant using a N_2_ balance (CO/H_2_/N_2_ 0.5:1:0.5), also revealed an impact on the initial rates across a range of substrates but to a lesser extent.

**Table 1:** Initial Rates and Product Ratios for Formylation vs Hydrodebromination for the Formylation of Aryl Bromide Catalyzed by 0.33 mol % [Pd(PAd_2_^*n*^Bu)_2_], 0.75 equiv TMEDA, in Toluene, 0.8 M, 100 °C, and 5 bar Pressure

R	σ_p_	initial rate (M h^–1^)	ArCHO/ArH	initial rate (M h^–1^)	ArCHO/ArH	initial rate (M h^–1^)	ArCHO/ArH
		1:1 CO/H_2_		0.5:1:0.5 CO/H_2_/N_2_		1:0.5:0.5 CO/H_2_/N_2_	
CN	0.66	0.05	8:1	0.04	1.3:1	0.03	7:1
CF_3_	0.54	0.10	17:1	0.07	2.0:1	0.04	16:1
Cl	0.23	0.11	45:1^a^	0.09	1.8:1^b^	0.06	99:1
OMe	–0.27	0.12	>99:1	0.10	1.7:1	0.06	99:1
NMe_2_	–0.83	0.14	>99:1	0.12	2.4:1	0.08	99:1

a 0.5% terephthalaldehyde was produced in this reaction.

b 1% terephthalaldehyde and 3% benzaldehyde were produced in this reaction.

As only two partial pressures have been investigated and data are limited, the impact of partial pressure on rate should be treated only semiquantitatively; in particular, for *P*_CO_, there are only small changes in rate in changing partial pressure, and the data remain ambiguous. A much clearer impact of *P*_CO_ can be obtained from the careful monitoring of product distributions. Under the standard conditions (CO/H_2_ 1:1) in which the Hammett analysis was conducted, small amounts (2–10%) of hydrodebrominated side-products were observed for the most electron-deficient substrates, and virtually, no side-products were observed for electron-rich substrates. At low *P*_CO_, the amounts of hydrodebrominated products increase significantly, with the poorest selectivity for formylation observed for the most electron-deficient substrates. These selectivity data provide indirect insight into relative rates and suggest that the migratory insertion step becomes slower at low *P*_CO_ with hydrodebromination taking over as a competitive process to formylation (Table 1). In contrast, varying *P*_H_2__ had a limited effect on the product distribution compared to standard conditions. Prior work has investigated the effect of gas ratios in aryl bromide formylation under flow conditions with optimum yields and selectivity found for 1:3 ratios of CO/H_2_.^10^

### Mechanistic Analysis

#### Summary of Kinetics

In combination, the kinetic data suggest that the precise mechanism of formylation of aryl bromides catalyzed by [Pd(PAd_2_^*n*^Bu)_2_] is substrate-dependent. The Hammett analysis is consistent with a change in the turnover-limiting sequence with a bifurcation into two catalytic regimes. Regime 1 can be defined for electron-deficient substrates with σ_p_ > 0, and regime 2 is for electron-rich substrates with σ_p_ < 0. The kinetic behavior of the catalytic system across both regimes is, however, strikingly similar.

In both regimes, first-order behavior in the catalyst suggests that monomeric palladium intermediates are likely involved in the turnover-limiting step. Catalyst speciation appears consistent across the time course, with no evidence for deactivation or change in behavior across electron-rich and electron-poor substrates. For both electron-rich and electron-deficient substrates, the order in aryl bromide is zero, and there is no evidence of product inhibition. The observed zero-order behavior argues against oxidative addition being part of the turnover-limiting sequence but rather suggests that it is a fast step that does not contribute significantly to the overall rate. It appears, however, that the base plays a role in the turnover-limiting step, with clear partial positive order close to 1 for the number of active basic sites. There is also a marked dependence of the reaction rate on H_2_ partial pressure, suggestive of the role of dihydrogen in the turnover-limiting step.

The Hammett analysis shows that for electron-donating substituents (σ_p_ > 0), there is no net influence of charge stabilization on the rate of the reaction, while for electron-deficient substituents (σ_p_ < 0), the rate increases with increasing ability of the substrate to stabilize positive charge with ρ = −1.4. These data also effectively rule out oxidative addition (and its microscopic reverse reductive elimination) as being turnover-limiting. Addition of aryl halides to palladium(0) complexes has been studied under a variety of conditions and typically occurs with ρ = +2 to +5^37,38^ due to the ability of electron-withdrawing groups to stabilize the buildup of negative charge on the *ipso*-carbon in either oxidative addition or S_N_Ar transition states.^39^ Negative ρ values observed for electron-deficient substituents are instead consistent with the substrate playing a role of a nucleophile in the turnover-limiting sequence and suggest that the migratory insertion step, while known to be fast and reversible,^20,40^ may contribute to the overall turnover-limiting sequence. A hypothesis that is also consistent with the observation of increased side-product formation and the small reduction of rates of reaction under lower partial CO pressures.

#### DFT Calculations

**Figure 3:**
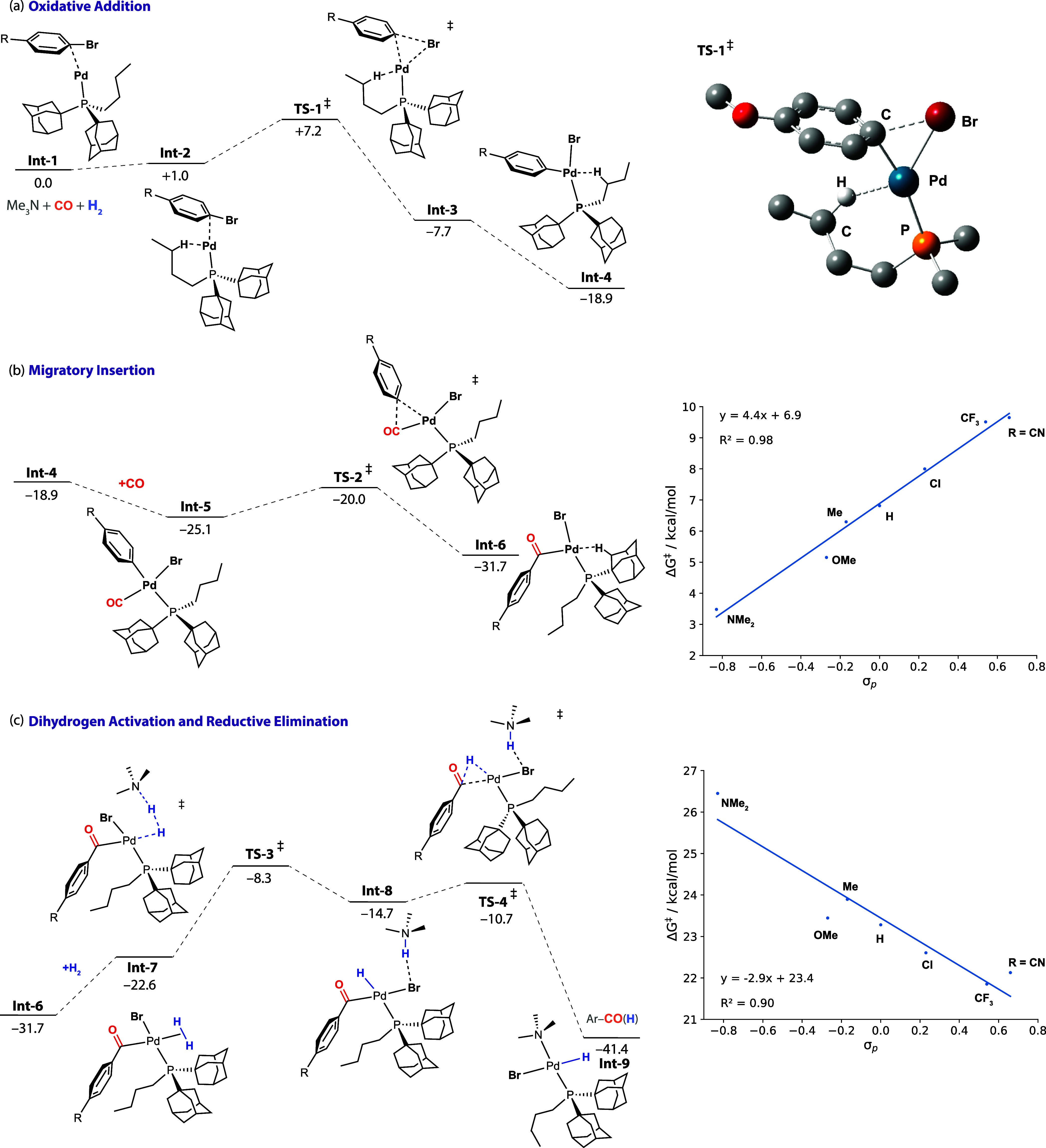
Calculated reaction profiles for the formylation of 4-bromoanisole for (a) oxidative addition, (b) migratory insertion, and (c) dihydrogen activation steps calculated at the ωB97x-D4/def2-TZVPPD/SMD (toluene)//ωB97x-D/def2-SVP (C,H)/def2-TZVPP (P,N,O,F,Cl,Br)/SDDAll (Pd) level of theory. For (b,c), the free energy relationship between σ_p_ and the activation barrier is also shown. Δ*G*_373K_^⧧^ values are in kcal mol^–1^. Adamantyl groups of PAd_2_^*n*^Bu have been truncated for clarity.

A series of DFT calculations were conducted to better understand the individual steps involved in catalytic turnover and the influence of substrate electronics on the said steps. Trimethylamine (Me_3_N) was used as a model for the base and [Pd(PAd_2_^*n*^Bu)] as the catalyst active site.^41^ A series of substrates were considered with varying Hammett parameters (e.g., R = CN, CF_3_, Cl, H, Me, OMe, and NMe_2_). DFT calculations were conducted using the ωB97x-D4 functional. Energies are reported following single-point corrections using the def2-TZVPPD basis set and an SMD (toluene) solvation model corrected for temperature and concentrations of reagents. A viable reaction pathway was calculated involving sequential (i) oxidative addition, (ii) CO coordination, (iii) migratory insertion, (iv) H_2_ coordination, (v) base-assisted dihydrogen activation, and (vi) reductive elimination (Figure 3).

This pathway can be considered to be initiated from **Int-1**, a weakly bound van der Waals complex between [Pd(PAd_2_^*n*^Bu)] and aryl bromide. **Int-1** is likely in equilibrium with **Int-2**, a conformer in which the ^*n*^Bu group of the phosphine ligand forms an agostic interaction through the γ-position of the carbon chain. Spectroscopic characterization of agostic interactions in these types of 3-coordinate complexes is common.^19,42^**Int-2** connects to the three-centered oxidative addition transition state **TS-1** via a low energy activation barrier (Δ*G*_373K_^⧧^ = 5.4–8.3 kcal mol^–1^). **TS-1** leads directly to the oxidative addition product **Int-3**, which can undergo facile cis/trans-isomerization to form **Int-4** with the strong *trans*-effect aryl ligand opposite the weakly coordinated agostic interaction (Δ*G*_373K_° = −10.8 to −13.1 kcal mol^–1^). Calculated activation barriers for the oxidative addition step decrease with electron-withdrawing groups and the increasing ability of the aryl moiety to stabilize the developing negative charge at the *ipso*-carbon (e.g., more positive σ_p_). Prior work has demonstrated that [Pd(PAd_2_^*n*^Bu)_2_] reacts with aryl bromides at 70 °C.^19^ Phosphine dissociation likely plays a significant role in determining the rate as the same complexes can be accessed at 25 °C from [Pd(dba)_2_] and 1 equiv of PAd_2_^*n*^Bu (dba = dibenzylideneacetone).^43^ Competition experiments are also consistent with predicted barriers for oxidative addition decreasing for more electron-deficient substrates.^14^ These predicted electronic effects do not match those observed in the Hammett analysis. Rather, oxidative addition is expected to be an accessible and nonreversible step that precedes the turnover-limiting sequence.

Carbon monoxide coordination to **Int-4** is exergonic and forms **Int-5**. From here, the migratory insertion is calculated to be a facile and reversible process. **Int-5** evolves to **TS-2** via a 1,1-insertion mechanism. Activation barriers for this step are again universally low (Δ*G*_373K_^⧧^ = 3.5–9.6 kcal mol^–1^). **TS-2** connects to the monomeric palladium acyl complex **Int-6**. Consistent with the low barrier, it has previously been shown that the reaction of three-coordinate palladium aryl complexes with CO occurs rapidly at 25 °C. An analogue of **Int-6** bearing P^*t*^Bu_3_ in place of PAd_2_^*n*^Bu has been isolated and crystallographically characterized. While this species is monomeric in the solid state, analogous complexes bearing PAd_2_^*n*^Bu are dimeric in the solid state.^19^ The overall reaction is only modestly exergonic (Δ*G*_373K_° = −2.5 to −8.5 kcal mol^–1^) and as such is likely to be reversible under catalytic conditions. The migratory insertion step is calculated to be more exergonic and occur with lower activation barriers for more electron-rich aryl substituents (e.g., more negative σ_p_). Based on DFT calculations alone, this step would be considered extremely facile and might not be considered as contributing to the turnover-limiting sequence. However, the calculations assume an available concentration of CO in the solution phase, which is likely misleading due to partitioning between the solution and gas phase, its limited solubility, and the potential to bind to off-cycle palladium species. Hence, while the absolute barriers are low, it remains likely that there is a contribution of the migratory insertion step to the turnover-limiting sequence based on the experimental Hammett analysis and the influence of CO pressure on selectivity (and the small effect seen on absolute rates).

Dihydrogen coordination to **Int-6** is moderately endergonic and leads to the formation of unstable dihydrogen complex **Int-7**. Experimental data supports the possible existence of dihydrogen complexes of palladium(II) in a four-coordinate environment.^44^ Deprotonation of **Int-7** by Me_3_N was calculated to occur through **TS-3**. **TS-3** is the highest barrier on the potential energy surface (Δ*G*_373K_^⧧^ = 21.9–26.4 kcal mol^–1^). **TS-3** progresses to **Int-8**, which is stabilized by a hydrogen bond between the liberated ammonium group, Me_3_NH, and the newly formed Pd bromide. Previously, it has been demonstrated that hydrogenolysis of dimeric analogues of **Int-6** occurs with optimum yields when conducted in the presence of TMEDA between 50 and 100 °C.^19,45^ A significant turnover-limiting contribution of the dihydrogen activation step is consistent with approximately first-order behavior in base (number of basic sites) and decreasing reaction rates with decreased dihydrogen pressure. This step alone does not explain the electronic effects observed across the two different kinetic regimes and needs to be considered alongside migratory insertion mentioned above as part of a turnover-limiting sequence.

Reductive elimination from **Int-8** via **TS-4** was calculated to be a low-energy process (Δ*G*_373K_^⧧^ = 2.4–3.6 kcal mol^–1^). The ammonium species was associated via a hydrogen bond throughout the reductive elimination step, ultimately forming the palladium hydride bromide complex **Int-9**. An analogue of **Int-9** in which the amine ligand is replaced by PAd_2_^*n*^Bu has been crystallographically characterized and proposed as an important off-cycle resting state.^19^ Dissociation of Me_3_NHBr from **Int-9** completes catalytic turnover, with both the aldehyde and ammonium salt side product liberated at this point.

#### Eyring Analysis

An Eyring analysis was conducted using 4-bromoanisole as a substrate across a 363–378 K temperature range at 5 K intervals. Under these conditions (kinetic regime 1), there is expected to be no or very little net influence of substrate electronics on the rate of reaction. The activation energy and activation entropy were derived as Δ*H*^⧧^ = +16.0 kcal mol^–1^ and Δ*S*^⧧^ = −36.6 cal K^–1^ mol^–1^, respectively. These data correspond to a Gibbs activation energy of Δ*G*_373K_^⧧^ = 29.6 kcal mol^–1^. This value is inconsistent with either oxidative addition, migratory insertion, or reductive elimination steps at a monophosphine palladium complex being turnover-limiting as each of these individual steps occurs with lower barriers based on DFT calculations. Rather, the experimental activation parameters most closely align with the barrier of the H_2_ splitting step from the DFT model (Δ*G*_373K_^⧧^ = 23.4 kcal mol^–1^).

#### Kinetic Isotope Effects

**Figure 4:**
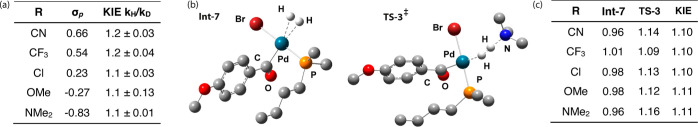
(a) Experimentally determined KIEs from comparison on rate constants from catalytic formylation reactions using 1:1 CO/H_2_ and CO/D_2_ (5 bar). (b) Computed structures for **Int-7** and **TS-3** annotated with key bond lengths, NPA charges, and Wiberg bond indices. (c) Calculated KIEs for dihydrogen binding to form **Int-7** and dihydrogen activation via **TS-3**. Calculated at the ωB97x-D4/def2-TZVPPD/SMD (toluene)//ωB97x-D/def2-SVP (C,H)/def2-TZVPP (P,N,O,F,Cl,Br)/SDDAll (Pd) level of theory. Adamantyl groups of PAd_2_^*n*^Bu have been truncated for clarity.

KIEs were measured by comparing absolute rates of reactions carried out with either CO/D_2_ or CO/H_2_ (1:1, 5 bar). Using the power of automation, KIEs were readily collected on a range of electron-rich and electron-poor substrates. These experiments revealed small KIE values ranging from 1.1 to 1.2 (Figure 4a). KIEs for dihydrogen activation can involve a contribution from both an inverse KIE for dihydrogen coordination and a normal KIE for the breaking of the H–H bond.^46−49^ DFT calculations were used to model the KIEs for the proposed dihydrogen activation steps. Dihydrogen binding to **Int-6** to form **Int-7** was calculated to occur with a small inverse KIE, while deprotonation of the dihydrogen complex **Int-7** through **TS-3** was predicted to occur with a small normal KIE (Figure 4b,c). Hence, DFT calculations predict that a base-assisted mechanism for hydrogen splitting, involving deprotonation of an intermediate Pd dihydrogen complex, should occur with a small normal KIE, in good agreement with the experimental data.

#### Mechanistic Model

**Figure 5:**
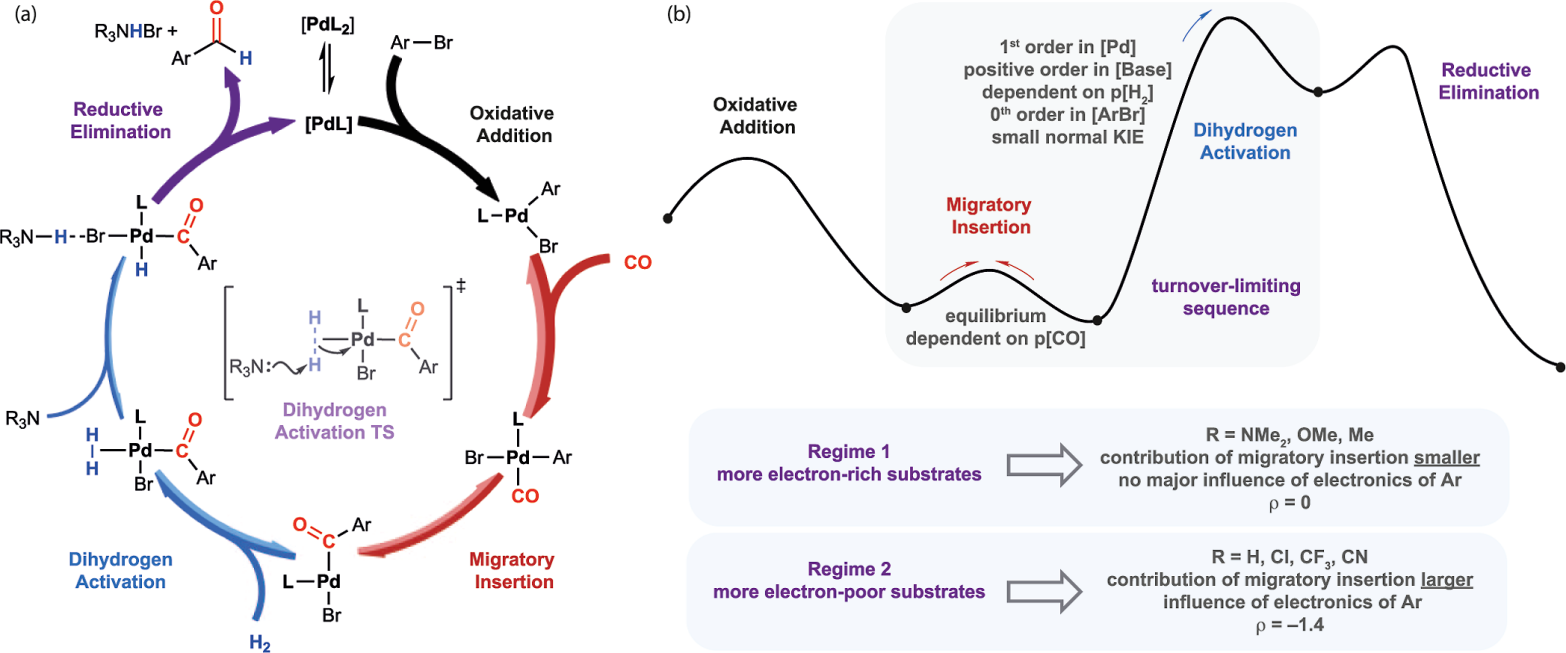
(a) Updated mechanism for the Pd-catalyzed formylation of aryl bromides by CO/H_2_ showing reversible steps. (b) Simplified reaction coordinate diagram for the catalytic cycle showing the turnover-limiting migratory insertion dihydrogen activation sequence, annotated with key findings from kinetics data and an explanation of two kinetic regimes determined by Hammett analysis.

The kinetic analysis and DFT calculations support an updated mechanistic model for the formylation of aryl bromides catalyzed by [Pd(PAd_2_^*n*^Bu)_2_]. The data are consistent with a turnover-limiting sequence involving migratory insertion and base-assisted dihydrogen activation steps. Migratory insertion can be considered to be a fast and reversible pre-equilibrium step that occurs prior to dihydrogen activation (Figure 5). While calculated to be facile, this equilibrium is expected to be sensitive to the concentration of CO in solution. Dihydrogen activation has the highest energy transition state on the potential energy surface, but migratory insertion is expected to contribute to the rate as it will determine the effective concentration of the key Pd acyl intermediate involved in this step (e.g., **Int-6**).

For electron-rich substrates, migratory insertion would be expected to be more facile with the equilibrium displaced further toward the products. For electron-poor substrates, the barriers of migratory insertion are higher, and the equilibrium is less displaced toward the products. Taken in combination, this model not only fits the complete kinetic data but also explains the two kinetic regimes apparent from the Hammett analysis. In regime 1, the contribution of the migratory insertion pre-equilibrium to turnover is small, and the electronic effect from this step and the dihydrogen activation step oppose each other, leading to a near net zero influence of electronics on the rate (ρ of zero). In regime 2, the contribution of the migratory insertion pre-equilibrium to the overall rate becomes more significant, and the electronic influence of this step now outweighs that of dihydrogen activation and results in more electron-deficient substrates reacting slower (ρ = −1.4). In the extreme, at low CO concentrations, this step becomes inefficient for electron-deficient substrates, leading to significant amounts of hydrodebromination side products.^50^

## Conclusions

In summary, through the use of robotics and an automated workflow, we have conducted a kinetic analysis of the palladium-catalyzed formylation of aryl bromides with CO/H_2_ mixtures using [Pd(PAd_2_^*n*^Bu)_2_]. Hammett analysis suggests that electron-rich and electron-deficient aryl bromides behave differently in this system. Thorough investigation of the order in catalyst, aryl bromide, and base along with the influence of partial pressure of CO and H_2_ was conducted for both electron-rich and electron-poor substrates. These studies revealed that while both types of aryl bromide substrates exhibit similar kinetic behavior, more electron-rich aryl bromides react faster and more selectively, with less formation of hydrodebromination side products. For both electron-rich and electron-poor substrates, product ratios are dependent on CO pressures, with increasing amounts of hydrodebromination at lower CO pressures. DFT calculations were used to probe plausible mechanisms for the oxidative addition, migratory insertion, H_2_ activation, and reductive elimination steps. Quantitative predictions from these calculations were compared with activation parameters acquired from an Eyring analysis and KIE acquired from side-by-side reactions with H_2_ and D_2_.

The combined kinetic and computational approaches allow a revised mechanistic model for palladium-catalyzed formylation to be proposed. Data are inconsistent with oxidative addition being turnover-limiting but rather support a turnover-limiting sequence involving a combination of a reversible migratory insertion step and a dihydrogen activation step. This sequence not only explains the kinetic behavior but also predicts the divergent behavior of electron-rich and electron-poor aryl bromides through the contribution of the migratory insertion equilibrium to the overall rate. Hence, for electron-rich substrates, this equilibrium is both faster to establish and more displaced toward the products, leading to fewer side reactions, and for electron-poor substrates, the equilibrium is slower to form and more displaced toward starting materials, resulting in a small reduction in the overall rate and opening up hydrodebromination side reactions. We believe our findings will have important implications in the development of both catalysts and optimum process conditions for the formylation of aryl halides with CO/H_2_ mixtures.

## Supporting Information Available

Experimental procedures, kinetic data, computational details, and Cartesian coordinates (PDF)
