# Supplementary material for: Combined Kinetic and Computational Analysis of the Palladium-Catalyzed Formylation of Aryl Bromides
Source: ACS Catal. 2024 Dec 18;15(1):343–51. doi: 10.1021/acscatal.4c05324 (PMC11705227; doi:10.1021/acscatal.4c05324)
Supplement: Supplementary file 1 — cs4c05324_si_001.pdf [file cs4c05324_si_001.pdf]

Supporting Information

A Combined Kinetic and Computational Analysis of the Palladium-Catalyzed Formylation of Aryl Bromides

Georgina Rai,[a] Lee J. Edwards,[b] Rebecca L. Greenaway,[a] Philip W. Miller,[a] Katherine M. P. Wheelhouse,[b] Mark R. Crimmin[a]**

[a]Molecular Sciences Research Hub, Imperial College London, 82 Wood Lane, Shepherds Bush, London, W12 0BZ, UK.
[b]GSK, GSK Medicines Research Centre, Gunnels Wood Road, Stevenage, Hertfordshire, SG1 2NY, UK.

m.crimmin@imperial.ac.uk
Katherine.m.wheelhouse@gsk.com

Table of Contents

1. Materials and methods S2

2. Experimental procedures and analytical data S3

3. Computational details S30

4. Cartesian coordinates of the stationary points S56

5. References S150

1. Materials and methods

General experimental

Unless otherwise stated, all manipulations were carried out using standard Schlenk and glovebox techniques, under inert atmosphere (dinitrogen or argon). An MBraun Labmaster glovebox was used, operating at <0.1 ppm H_2O and <0.1 ppm O_2. Solvents were dried over activated alumina from a solvent purification system (SPS) based upon the Grubbs design and degassed before use. Glassware and PTFE liners/stirrer bars were dried for >6 h prior to use at 120 °C. Benzene-d_6 and toluene-d_8 were degassed by freeze-pump-thaw cycles and stored over 3 Å molecular sieves before use. Methanol was dried over 20% m/v 3 Å molecular sieves for 5 days and sparged with argon before use. TMEDA was distilled from CaH_2 and degassed by freeze-pump-thaw cycles before use. All reagents were acquired from commercial suppliers (Sigma Aldrich, VWR, Fluorochem, Apollo Scientific) and used without further purification unless specified. Synthesis gas was acquired from BOC in three different ratios: $CO{:}H_2$ 1:1, $CO{:}H_2{:}N_2$ 0.5:1:0.5 and $CO{:}H_2{:}N_2$ 1:0.5:0.5, and used without further purification. Where liquids at 25 °C, reagents were distilled, dried over activated 3 Å molecular sieves and freeze-pump-thaw degassed prior to use. $[PdCl(\pi\text{-cinnamyl})]_2$, $[PdCp(\pi\text{-cinnamyl})]$, $[Pd(P(1\text{-Ad})_2{}^n\text{Bu})_2]$, and TMEDA·2HBr were prepared by modified literature procedures.[1-3]

^{1}H, ^{31}P{^{1}H} and ^{19}F NMR spectra were recorded on BRUKER 400 MHz or 500 MHz spectrometers. ^{1}H NMR chemical shifts (δ) were referenced to internal solvent resonances, while ^{31}P{^{1}H} and ^{19}F NMR chemical shifts were referenced to 85% H_3PO_4 and $CFCl_3$ respectively. Data were processed using the MestReNova software package. The coupling constants (J) are reported in Hertz (Hz). The following abbreviations are used to define multiplicities: s (singlet), d (doublet), t (triplet), q (quartet), m (multiplet), br s (broad signal). Quantitative gas chromatography (GC) analyses were performed with an Agilent Technologies 7820A GC with FID detector using an Agilent HP-5 column. The carrier gas used was helium. Hexadecane was used as internal standard.

High pressure (5-7 bar) reactions were performed either in a Parr Instruments® Series 4790 GP 25 mL reactor, a Parr Instruments® Series 4760 GP 300 mL reactor, or using an Unchained Labs Junior Platform equipped with an Optimisation Sampling Reactor. For the Parr Instruments® reactors, heating was supplied by a hot plate stirrer and an aluminium heating block and monitored using an internal thermocouple. Stirring was provided using a PTFE coated magnetic stirrer and the reactors were fitted with PTFE liners. For the Optimisation Sampling Reactor, maps were created in Library Studios for execution on the Unchained Labs' platform. Unchained Labs' LEA 9.2 Automation Studio was used to execute the libraries and monitor system diagnostics. Stock solutions were dispensed using a HandyStep® touch S repetitive pipette. Polyether ether ketone (PEEK) stir paddles attached to the underside of the module's stir top provided overhead stirring. Glass liners were used in reactor wells. All PTFE and glass liners and PTFE coated stirrers were subject to a rigorous cleaning procedure after each reaction: an acetone rinse, a distilled water rinse, then an aqua regia soak (10 – 20 minutes) followed by a thorough wash with distilled water and a final rinse with acetone. PEEK stir paddles were disposed of after every reaction and used new due to incompatibility with aqua regia.

Samples generated from reactions using the Optimisation Sampling Reactor were filtered and transferred to GC vials using the Opentrons OT-2 liquid handling platform (app version 7.2.1) equipped with the Opentrons 300 µL eight-channel and 1 mL single-channel pipettes and their corresponding proprietary pipette tips. Samples were held in a Porvair 2 mL 96-well polypropylene plate, then filtered through a Restek PPT3 96-well filter plate. GC vials were held using a polylactic acid block. OT-2 protocols were written using the Opentrons Protocol Designer.

2. Experimental procedures and analytical data

Gas calculations

The amount of CO/H_2 (mol) used in catalytic reactions was calculated using the ideal gas law.

$$mol\ of\ gas = \frac{pressure\ (bar) \times volume\ (L)}{0.08314\ (L\ bar\ K^{-1}\ mol^{-1}) \times temperature\ (K)}$$

Yield and conversion calculations – ^{1}H NMR spectroscopy

All ^{1}H NMR spectra integrals for 4-bromoanisole, 4-bromobenzotrifluoride, 4-methoxybenzaldehyde and 4-(trifluoromethyl)benzaldehyde were referenced to the internal standard. Methoxy proton integrals (OMe) and aromatic proton integrals (CF_3) were used for the following calculations:

$$Yield = \frac{Integral\ of\ product}{Integral\ of\ starting\ material\ at\ time = 0} \times 100\%$$

$$Conversion = \frac{(Integral\ of\ starting\ material\ at\ time = 0) - (Final\ integral\ of\ starting\ material)}{Integral\ of\ starting\ material\ at\ time = 0} \times 100\%$$

Yield and conversion calculations – GC-FID

Calibration curves were obtained for all substrates, aldehyde products and hydrodebrominated side products, referenced to the hexadecane internal standard. Concentrations of substrate and product were calculated from these given the known concentration of hexadecane internal standard in each reaction.

$$Yield = \frac{Concentration\ of\ product}{Concentration\ of\ starting\ material\ at\ time = 0} \times 100\%$$

$$Conversion = \frac{(Concentration\ of\ starting\ material\ at\ time = 0) - (Final\ concentration\ of\ starting\ material)}{Concentration\ of\ starting\ material\ at\ time = 0} \times 100\%$$

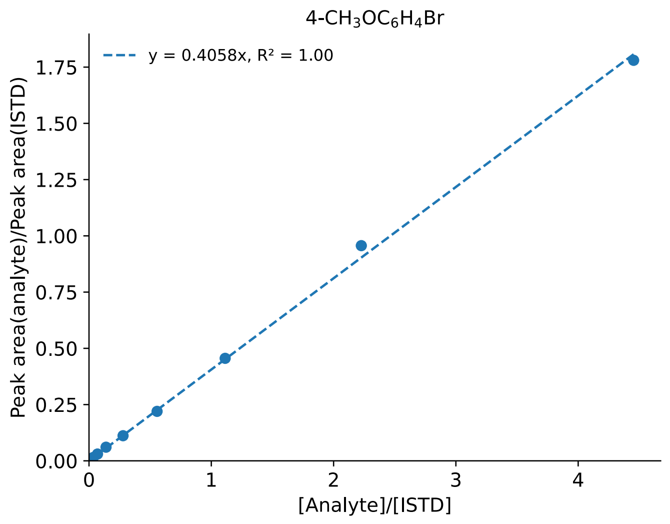

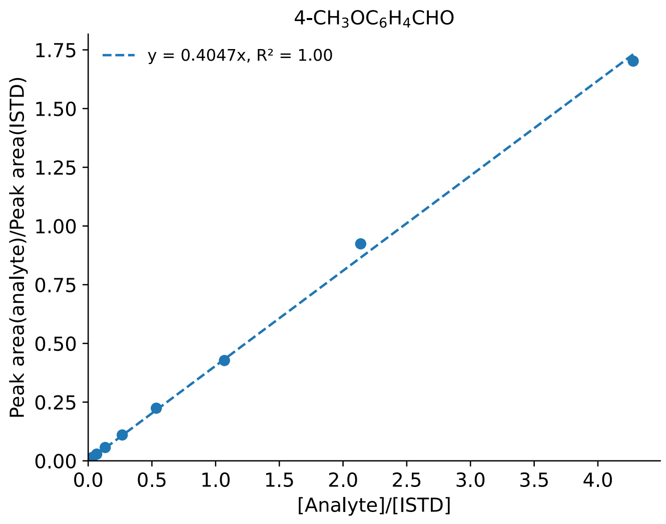

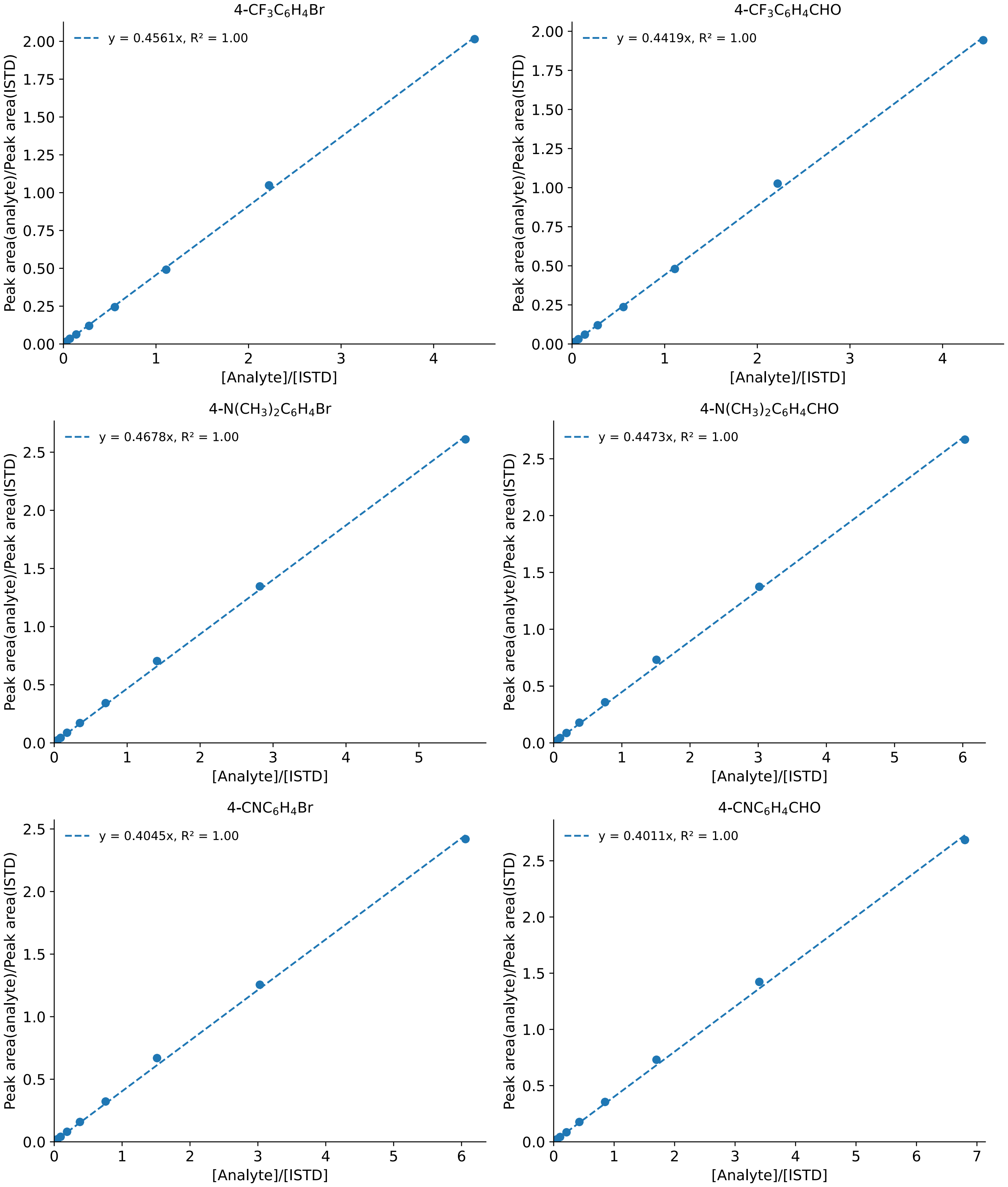
4-CF3C6H4Br
y = 0.4561x, R² = 1.00
Peak area(analyte)/Peak area(ISTD)
[Analyte]/[ISTD]
4-CF3C6H4CHO
y = 0.4419x, R² = 1.00
Peak area(analyte)/Peak area(ISTD)
[Analyte]/[ISTD]
4-N(CH3)2C6H4Br
y = 0.4678x, R² = 1.00
Peak area(analyte)/Peak area(ISTD)
[Analyte]/[ISTD]
4-N(CH3)2C6H4CHO
y = 0.4473x, R² = 1.00
Peak area(analyte)/Peak area(ISTD)
[Analyte]/[ISTD]
4-CNC6H4Br
y = 0.4045x, R² = 1.00
Peak area(analyte)/Peak area(ISTD)
[Analyte]/[ISTD]
4-CNC6H4CHO
y = 0.4011x, R² = 1.00
Peak area(analyte)/Peak area(ISTD)
[Analyte]/[ISTD]

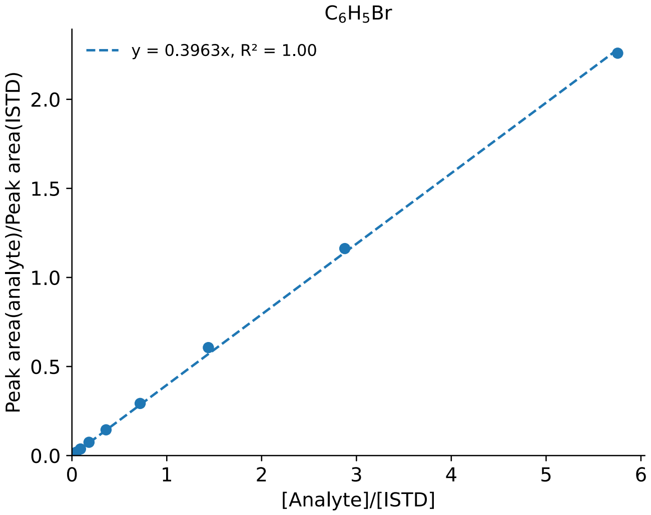
C_6H_5Br
y = 0.3963x, R² = 1.00
Peak area(analyte)/Peak area(ISTD)
[Analyte]/[ISTD]

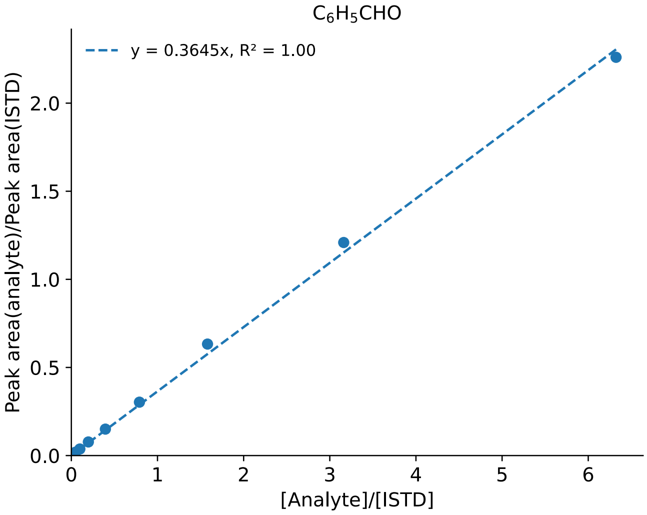
C_6H_5CHO
y = 0.3645x, R² = 1.00
Peak area(analyte)/Peak area(ISTD)
[Analyte]/[ISTD]

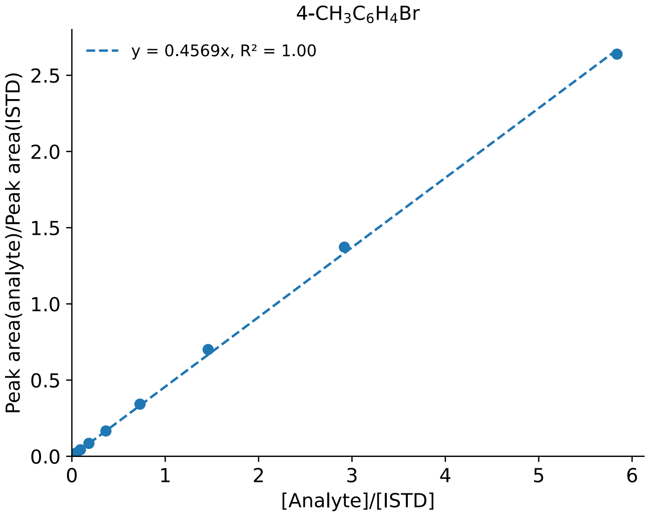
4-$CH_3C_6H_4Br$
y = 0.4569x, R² = 1.00
Peak area(analyte)/Peak area(ISTD)
[Analyte]/[ISTD]

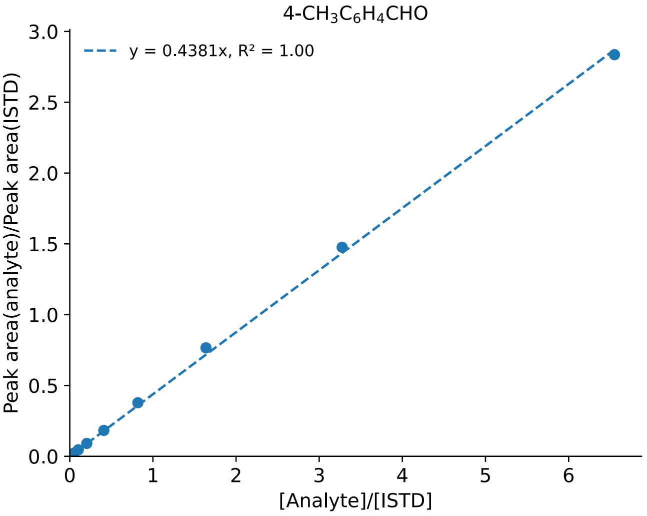
4-$CH_3C_6H_4CHO$
y = 0.4381x, R² = 1.00
Peak area(analyte)/Peak area(ISTD)
[Analyte]/[ISTD]

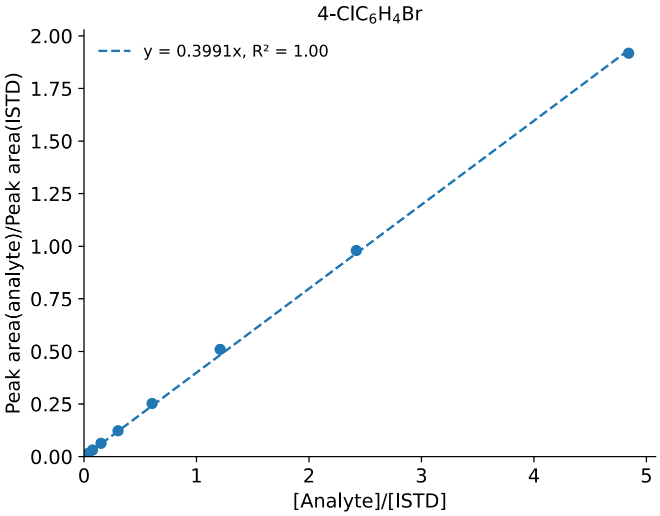
4-ClC_6H_4Br
y = 0.3991x, R² = 1.00
Peak area(analyte)/Peak area(ISTD)
[Analyte]/[ISTD]

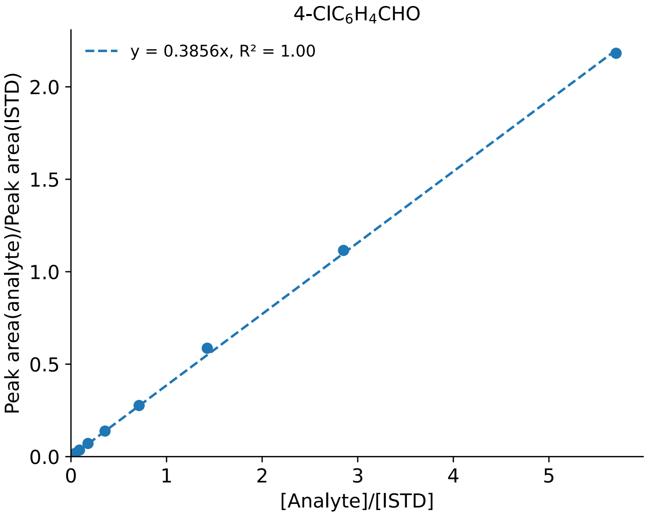
4-ClC_6H_4CHO
y = 0.3856x, R² = 1.00
Peak area(analyte)/Peak area(ISTD)
[Analyte]/[ISTD]

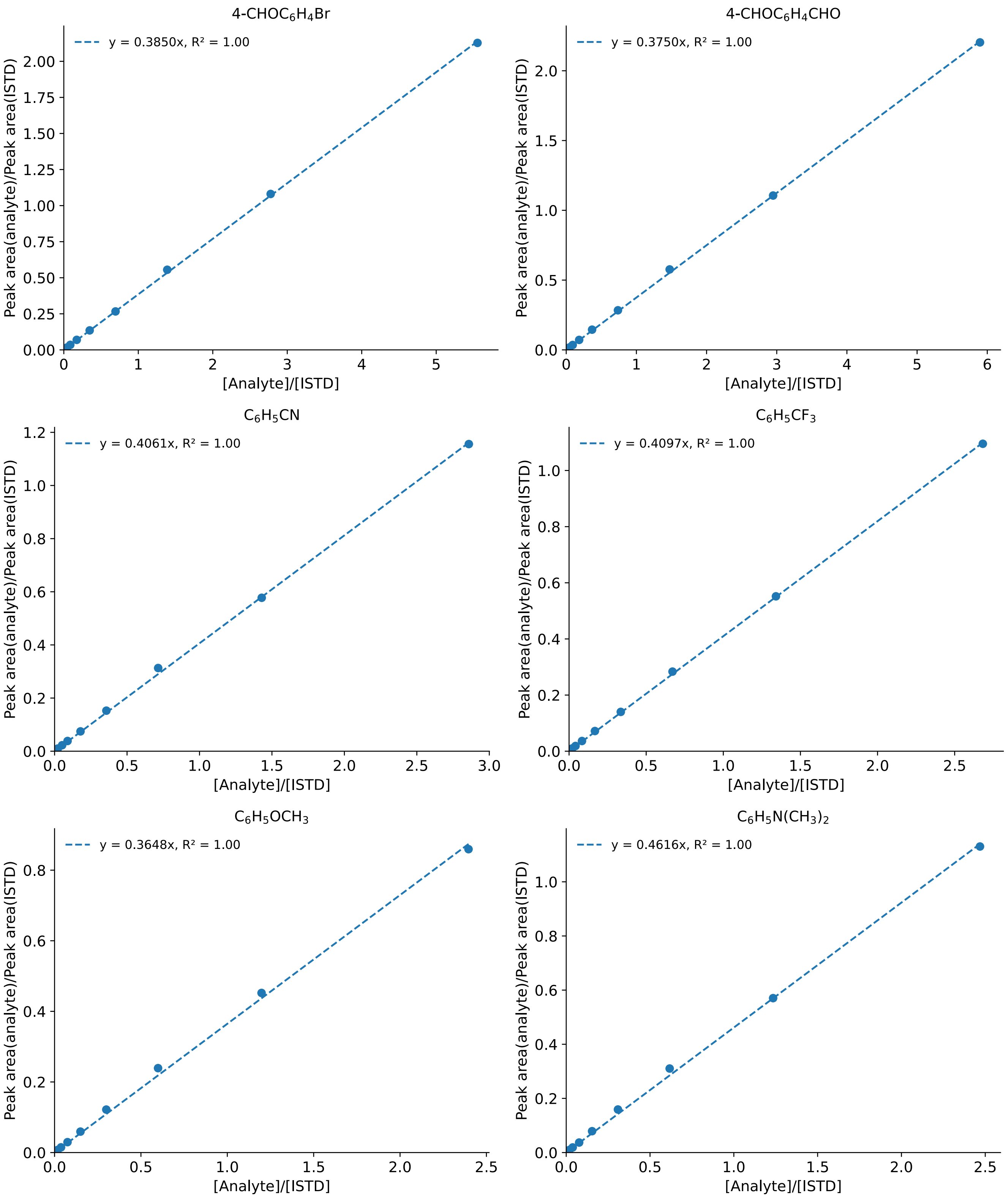
4-CHOC6H4Br
y = 0.3850x, R² = 1.00
4-CHOC6H4CHO
y = 0.3750x, R² = 1.00
C6H5CN
y = 0.4061x, R² = 1.00
C6H5CF3
y = 0.4097x, R² = 1.00
C6H5OCH3
y = 0.3648x, R² = 1.00
C6H5N(CH3)2
y = 0.4616x, R² = 1.00
Peak area(analyte)/Peak area(ISTD)
[Analyte]/[ISTD]

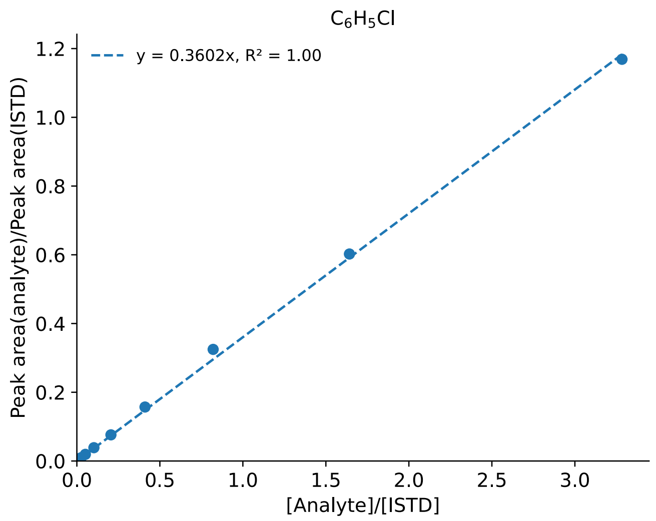

Figure S1. Calibration curves for all substrates, products and side products.

General procedure A for the formylation of aryl bromides – small scale, Parr® reactor

In a N_2 glovebox, a Parr Instruments® reactor equipped with a PTFE liner was charged with the appropriate catalyst precursor, either $Pd(OAc)_2$ (20 mM solution in toluene, 333 µL, 0.0066 mmol, 0.0033 equiv.) and $P(1\text{-}Ad)_2{}^nBu$ (7.2 mg, 0.02 mmol, 0.01 equiv.) or $[Pd(P(1\text{-}Ad)_2{}^nBu)_2]$ (6.7 mM solution in toluene, 1 mL, 0.0066 mmol, 0.0033 equiv.). Toluene (1-1.67 mL), *n*-dodecane (113.6 µL, 0.5 mmol, 0.25 equiv., internal standard), the corresponding aryl bromide (2 mmol, 1 equiv.) and TMEDA (225 µL, 1.5 mmol, 0.75 equiv.) were then added. To determine the initial substrate concentration, an aliquot (50 µL) of the reaction mixture was taken for quantitative ^1H NMR spectroscopy. The pressure vessel was sealed and removed from the glovebox. Inlet and outlet lines were fitted to the vessel, and the inlet valve was purged with N_2 (10 × 2 bar) before the vessel was opened to the gas line. The vessel was then purged with CO/H_2 (1:1, 5 × 5 bar) before being pressurised to 5 bar with CO/H_2. The reaction mixture was stirred at 100 °C, giving a final pressure of 6.5 bar, for 16 h, with stirring set to 700 rpm. The reactor was then cooled to room temperature, CO/H_2 vented, and the reactor opened in air. The reaction mixture was then filtered through a 0.2 µm PTFE filter, and an aliquot was taken for ^1H NMR spectroscopic analysis to determine yield and conversion.

Table S1. Reproduction of catalytic conditions and control reactions on 2 mmol scale.

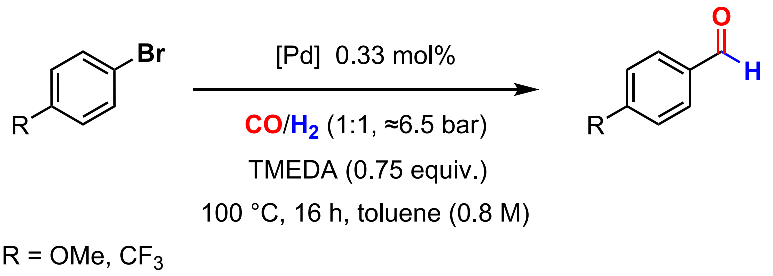

Entry	R	Catalyst precursor	Change from standard conditions	Conversion %[a]	Yield %[a]
**1**	OMe	$Pd(OAc)_2/P(1\text{-}Ad)_2{}^{n}Bu)_2$	N/A	100	100
**2**	CF_3	$Pd(OAc)_2/P(1\text{-}Ad)_2{}^{n}Bu)_2$	N/A	91	84
**3**	OMe	$[Pd(P(1\text{-}Ad)_2{}^{n}Bu)_2]$	N/A	98	98
**4**	CF_3	$[Pd(P(1\text{-}Ad)_2{}^{n}Bu)_2]$	N/A	94	86
**5**	OMe	N/A	No catalyst	0	0
**6**	OMe	$Pd(OAc)_2$	No ligand	0	0
**7**	OMe	$[Pd(P(1\text{-}Ad)_2{}^{n}Bu)_2]$	No TMEDA	1	1
**8**	OMe	$[Pd(P(1\text{-}Ad)_2{}^{n}Bu)_2]$	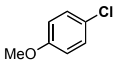 Substrate	0	0

[a]Determined by ^1H NMR spectroscopy using *n*-dodecane internal standard.

General procedure B for the formylation of aryl bromides – large scale, Parr® reactor

In a N_2 glovebox, a Parr Instruments® reactor equipped with a PTFE liner was charged with $[Pd(P(1\text{-}Ad)_2{}^{n}Bu)_2]$ (35.7 mg, 0.043 mmol, 0.0033 equiv.). Toluene (12 mL), hexadecane (952 μL, 3.25 mmol, 0.25 equiv., internal standard), the corresponding aryl bromide (13 mmol, 1 equiv.) and TMEDA (1.46 mL, 9.75 mmol, 0.75 equiv.) were then added. To determine the initial substrate concentration, two aliquots (2 x 50 μL) of the reaction mixture were taken for quantitative ^1H NMR spectroscopy and GC-FID. The pressure vessel was sealed and removed from the glovebox. Inlet and outlet lines were fitted to the vessel, and the inlet valve was purged with N_2 (10 × 2 bar) before the vessel was opened to the gas line. The vessel was then purged with CO/H_2 (1:1, 5 × 5 bar) before being pressurised to 5 bar with CO/H_2. The reaction mixture was stirred at 100 °C, giving a final pressure of 3.5 bar, for 16 h, with stirring set to 700 rpm. The reactor was then cooled to room temperature, CO/H_2 vented, and the reactor opened in air. The reaction mixture was then filtered through a 0.2 μm PTFE filter, and an aliquot was taken for ^1H NMR spectroscopy and GC-FID analysis to determine yield and conversion.

Table S2. Reproduction of catalytic conditions with two electronically different substrates on 13 mmol scale, comparing quantitative ^{1}H NMR spectroscopy and GC-FID.

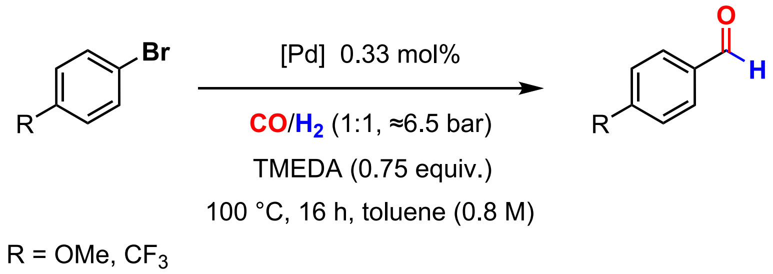

Entry	R	^{1}H NMR conversion %[a]	^{1}H NMR yield %[a]	GC conversion %[b]	GC yield %[b]
**1**	CF_3	100	95	100	93
**2**	OMe	99	99	99	99

[a]Determined by ^{1}H NMR spectroscopy using hexadecane internal standard.
[b]Determined by GC-FID using hexadecane internal standard.

Preparation of 4-methoxybenzaldehyde. Following general procedures A and B, 4-methoxybenzaldehyde was obtained in the above yields. ^{1}H NMR (400 MHz, $CDCl_3$) δ (ppm): 9.88 (s, 1H), 7.83 (d, *J* = 8.8 Hz, 2H), 6.99 (d, *J* = 8.7 Hz, 2H), 3.86 (s, 3H). Data are consistent with those previously reported.[4]

Preparation of 4-(trifluoromethyl)benzaldehyde. Following general procedures A and B, 4-(trifluoromethyl)benzaldehyde was obtained in the above yields. ^{1}H NMR (400 MHz, $CDCl_3$) δ (ppm): 10.08 (s, 1H), 7.99 (d, *J* = 8.0 Hz, 2H), 7.80 (d, *J* = 8.1 Hz, 2H). Data are consistent with those previously reported.[5]

Existing mechanistic hypothesis

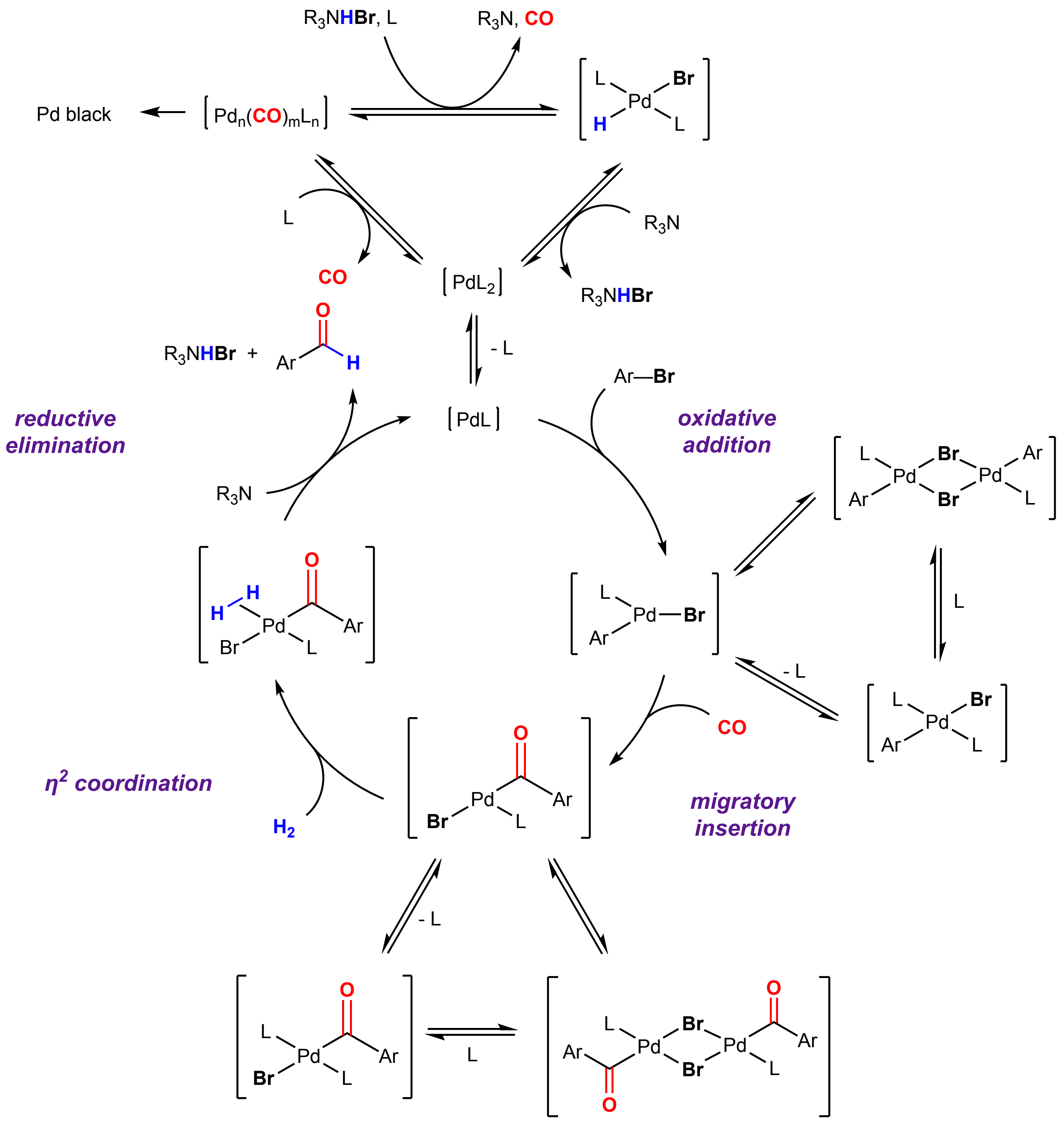

Figure S2. Existing mechanistic hypothesis for the palladium-catalysed formylation of aryl bromides with syngas and external amine base proposed by Beller and coworkers.[1] L = $PAd_2{}^nBu$.

Reproducibility testing using the OSR platform

General procedure C for the formylation of aryl bromides

In a N_2 purge (glove)box, the Optimisation Sampling Reactor module equipped with curve-bottom glass liners was charged with [Pd(P(1-Ad)$_2$^{n}Bu)$_2$] (0.01 M solution in toluene, 5.42 mL, 0.043 mmol, 0.0033 equiv.), toluene (3.16 mL), hexadecane (1.2 M solution in toluene, 2.71 mL, 3.25 mmol, 0.25 equiv., internal standard), 4-bromoanisole (4.8 M solution in toluene, 2.71 mL, 13 mmol, 1 equiv.) and TMEDA (3.2 M solution in toluene, 3.05 mL, 9.75 mmol, 0.75 equiv.). Equipped with overhead PEEK stir paddles, the pressure vessel was sealed and purged with N_2 (2 × 7 bar). To determine the initial substrate concentration, a sample (100 μL) was taken from all reactors and deposited into a 96-well sample plate, filled with EtOAc (500 μL) for quenching, and chased with toluene (250 μL). All reactors were then heated to 100 °C, set to stirring speed 800 rpm, then pressurised to 5 bar with CO/H_2 (1:1). Samples were taken according to the library design at 0.5, 1, 1.5, 2, 3, 4, 6, 8, 10, 12 and 16 h. After this, the reactors were cooled and depressurised. Collected samples were held in a 96-well sample plate, removed from the purge box once all sampling had completed, and diluted with ethyl acetate (EtOAc, 1 mL). Samples were filtered using a 0.45 μm 96-well filter plate under reduced pressure, then transposed into GC vials, and subject to GC-FID analysis to determine concentration of substrate and product.

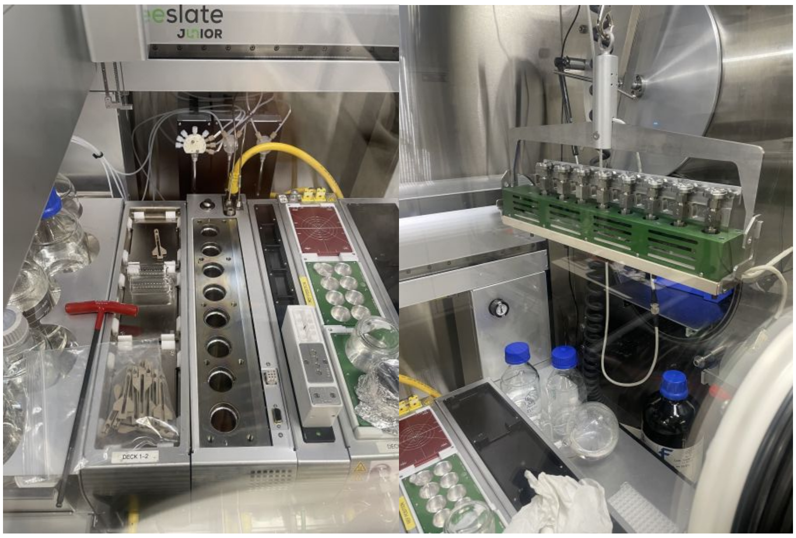

Figure S3. OSR Reactor module and stir top plate.

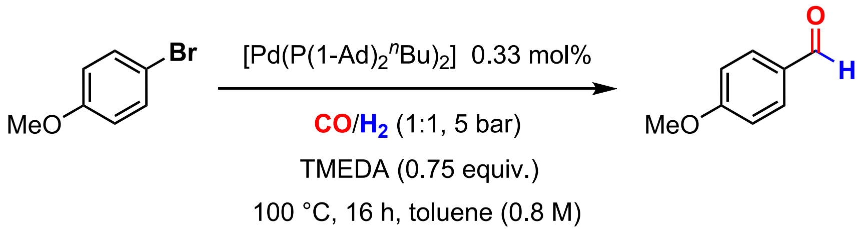

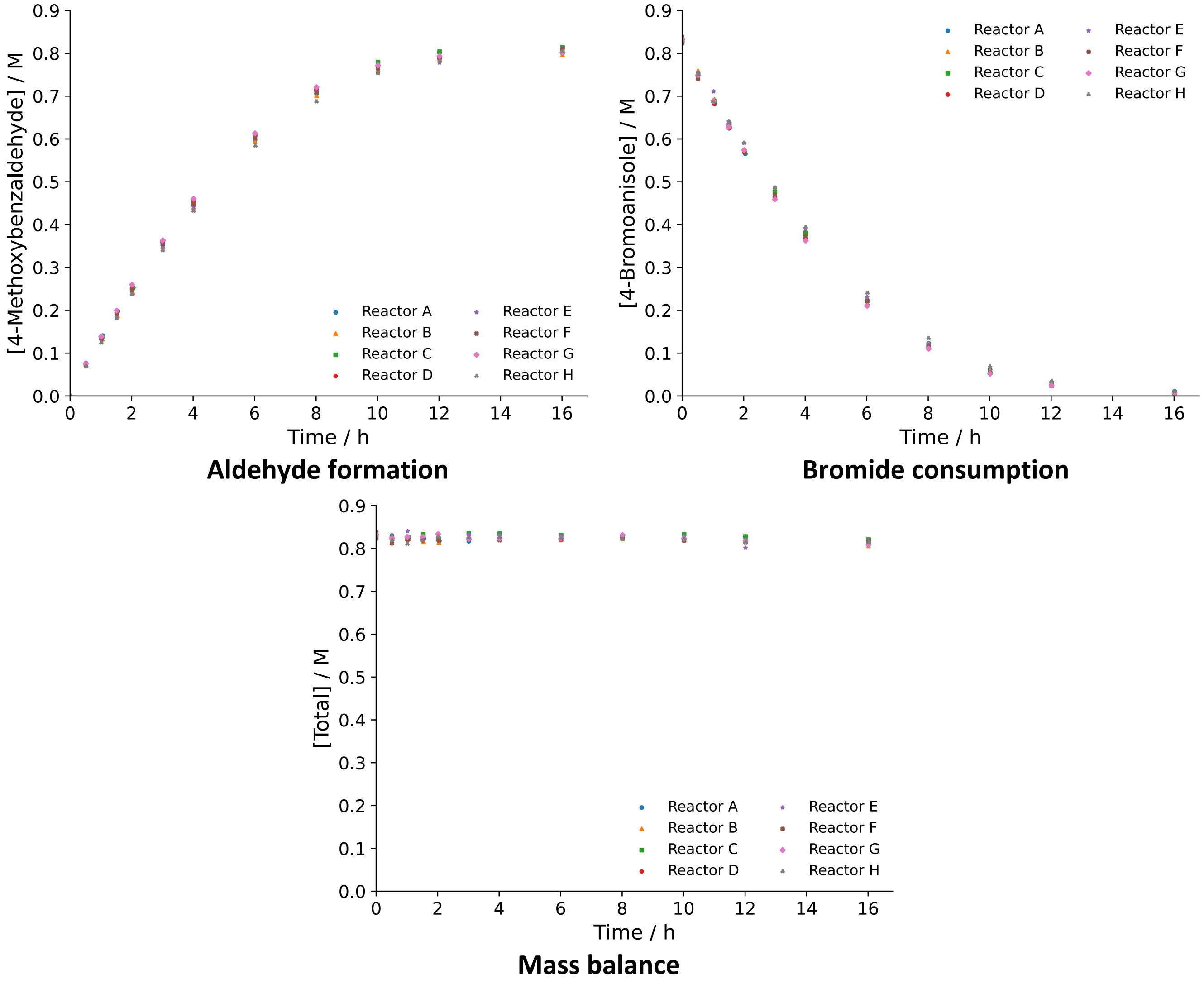

Figure S4. Concentration-time profiles for catalytic formylation of 4-bromoanisole under standard conditions for all eight reactors.

Variation of stirrer speed

The reproducibility test described above was repeated at a stirrer speed of 500 rpm. A reduction in initial rate would indicate mass transfer issues within the system.

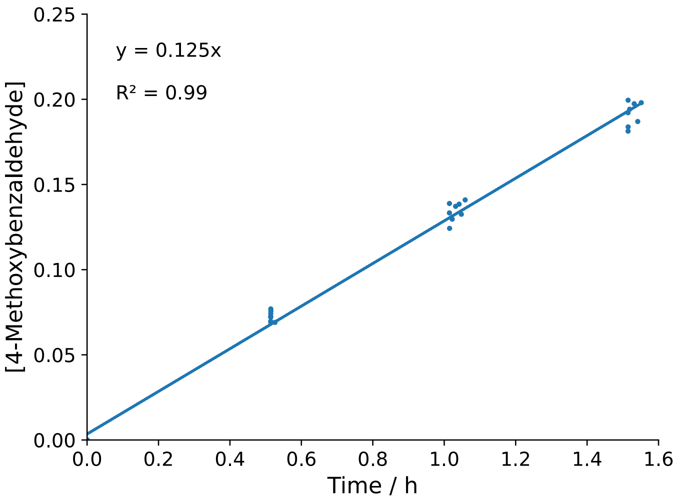

800 rpm

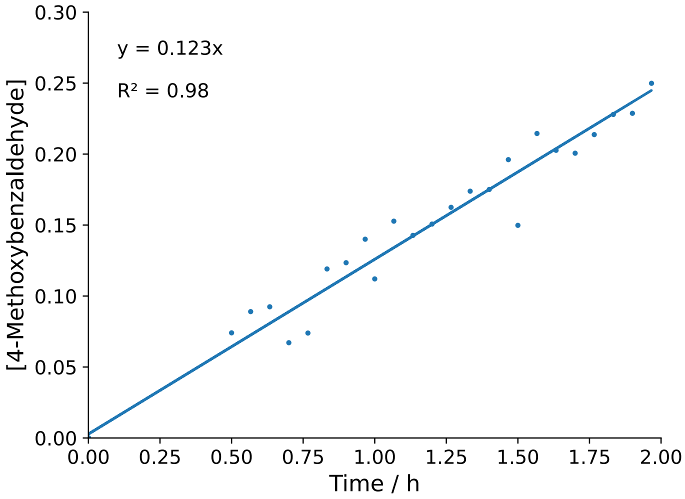

500 rpm

Stirrer speed / rpm	Initial rate / h^{-1}
500 rpm	0.123
800 rpm	0.125

Figure S5. Initial rates of catalytic formylation of 4-bromoanisole under standard conditions at different stirrer speeds.

Both stirrer speeds result in the same initial rates of reaction, supporting that there are no mass transfer limitations within the system. All subsequent reactions using the OSR platform were carried out at 800 rpm.

Kinetic data collection

General procedure D for the formylation of aryl bromides

In a N_2 purge (glove)box, the Optimisation Sampling Reactor module equipped with curve-bottom glass liners was charged with $[Pd(P(1\text{-}Ad)_2{}^{n}Bu)_2]$ (0.01 M solution in toluene, 5.42 mL, 0.043 mmol, 0.0033 equiv.), toluene (1.82-3.16 mL), hexadecane (1.2 M solution in toluene, 2.71 mL, 3.25 mmol, 0.25 equiv., internal standard), corresponding aryl bromide (4.8 M solution in toluene, 2.71 mL, 13 mmol, 1 equiv.) and TMEDA (3.2 M solution in toluene, 3.05 mL, 9.75 mmol, 0.75 equiv.) or Et_3N (4.4 M solution in toluene, 4.39 mL, 19.5 mmol, 1.5 equiv.). Equipped with overhead PEEK stir paddles, the pressure vessel was sealed and purged with N_2 (2 × 7 bar). To determine the initial substrate concentration, a sample (100 μL) was taken from all reactors and deposited into a 96-well sample plate, filled with EtOAc (500 μL) for quenching, and chased with toluene (250 μL). All reactors were then heated to 100 °C, set to stirring speed 800 rpm, then pressurised to 5 bar with CO/H_2 (1:1). Samples were taken according to the library design at 0.5, 1, 1.5, 2, 3, 4, 6, 8, 10, 12 and 16 h. After this, the reactors were cooled and depressurised. Collected samples were held in a 96-well sample plate, removed from the purge box once all sampling had completed, and diluted with EtOAc (1 mL). Using the OT-2, samples were filtered using a 0.45 μm 96-well filter plate under reduced pressure, then transposed into GC vials, and subject to GC-FID analysis to determine concentrations of substrate and product.

Hammett analysis

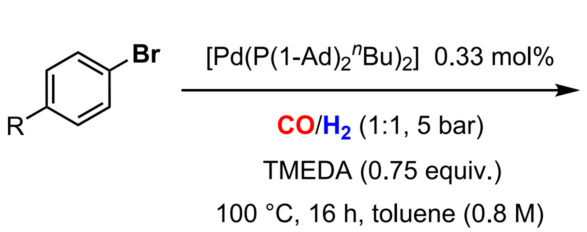

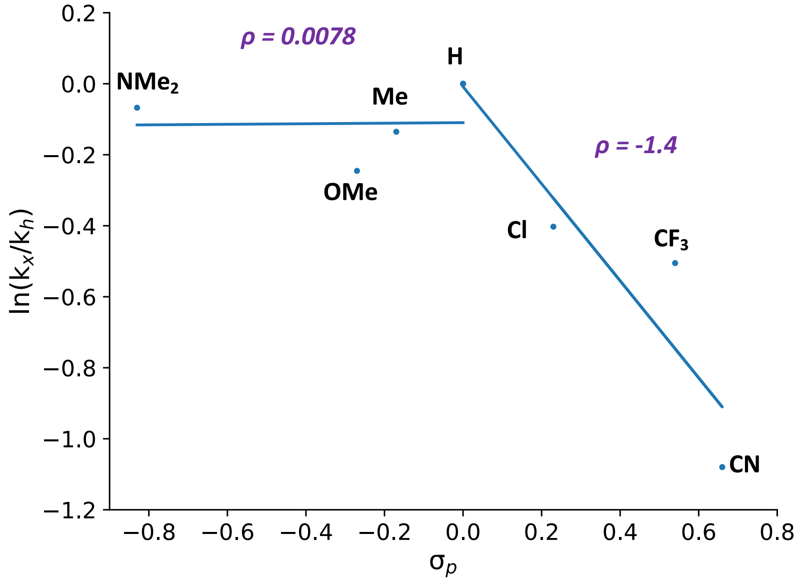

R	σ_p	Initial rate / h^{-1}
CN	0.66	0.054
CF_3	0.54	0.095
Cl	0.23	0.11
H	0	0.15
Me	-0.17	0.14
OMe	-0.27	0.12
NMe_2	-0.83	0.14

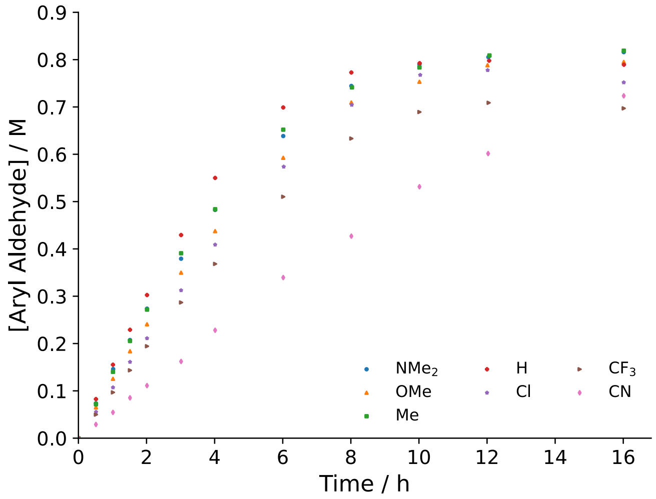

R	Conversion %[a]	Aldehyde yield %[a]	Hydrodebrominated product yield %[a]
CN	100	77	10
CF_3	100	87	5
Cl[b]	100	90	2
H	100	96	0
Me	99	101	0
OMe	99	96	0
NMe_2	99	95	0

[a]Determined by GC-FID using hexadecane internal standard.

[b]5 % terephthalaldehyde was produced in this reaction.

Figure S6. Hammett plot and overlay of concentration-time profiles of aldehyde formation for each Hammett substrate.

Order in reagents

Reaction orders in aryl bromide substrate and catalyst were determined using variable time normalisation analysis (VTNA) developed by Burés.[6,7] Reaction orders in base (TMEDA or Et_3N) were determined using the initial rates method, by determining the gradient in the linear section of the concentration *vs* time plot. Ln(k_{obs}) *vs* ln(concentration) plots were then constructed, where the gradient is equal to the order in that reagent.

Order in catalyst using VTNA (4-bromoanisole):

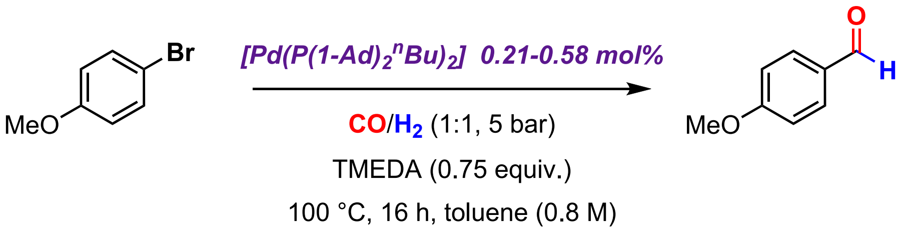

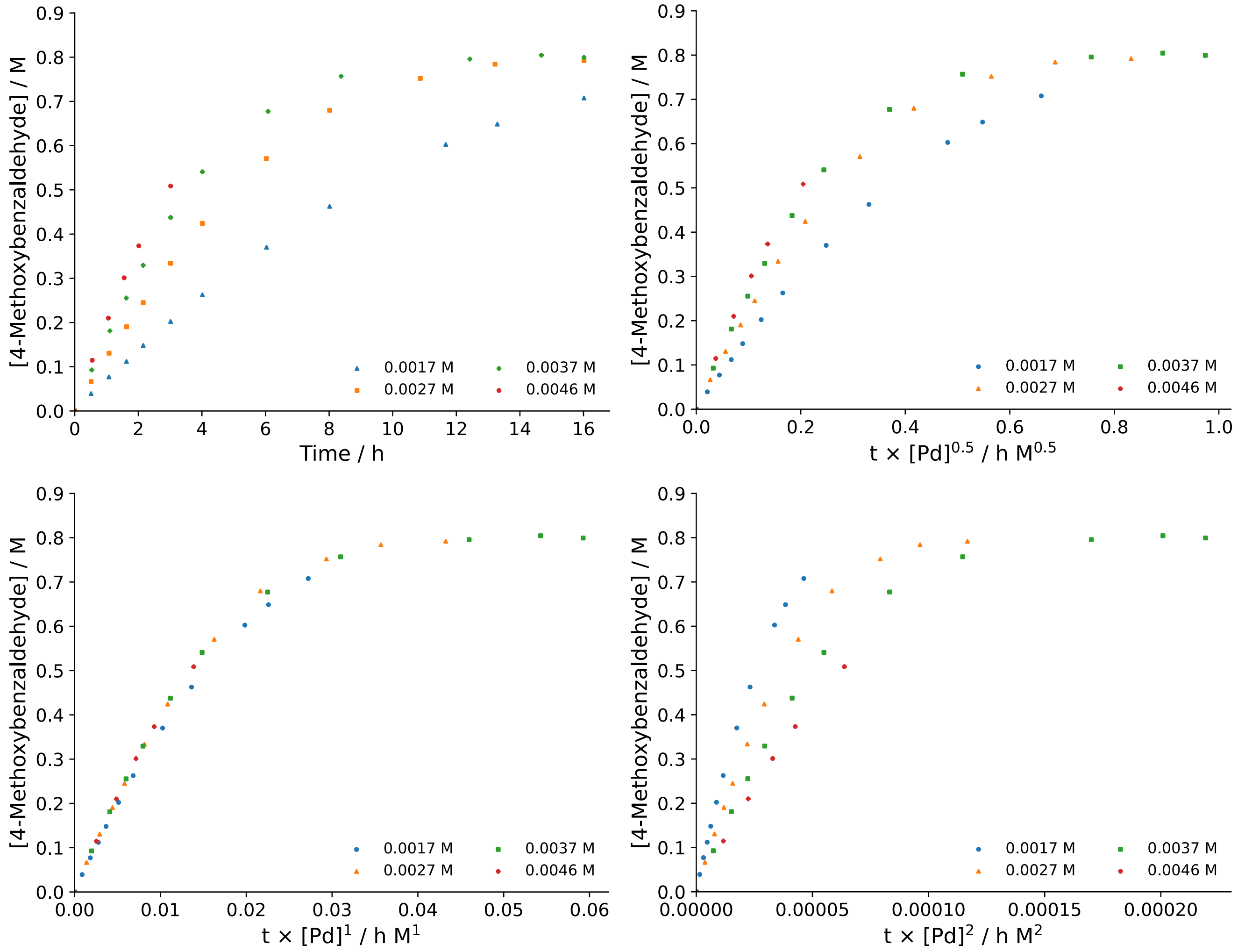

Figure S7. VTNA applied to the concentration-time profiles of 4-methoxybenzaldehyde formation at different concentrations of catalyst. Best overlay is seen with 1st order in catalyst.

Data for 0.0046 M catalyst concentration were collected up to 3 hours only.

Order in catalyst using VTNA (4-bromobenzotrifluoride):

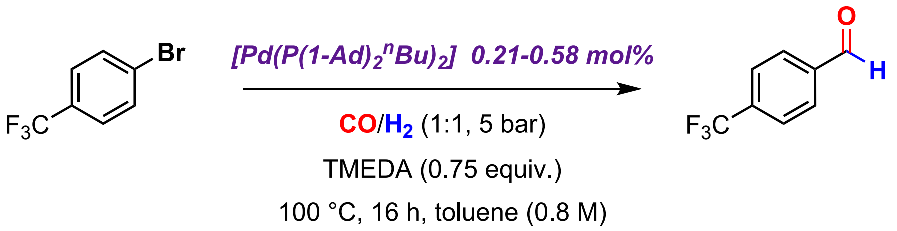

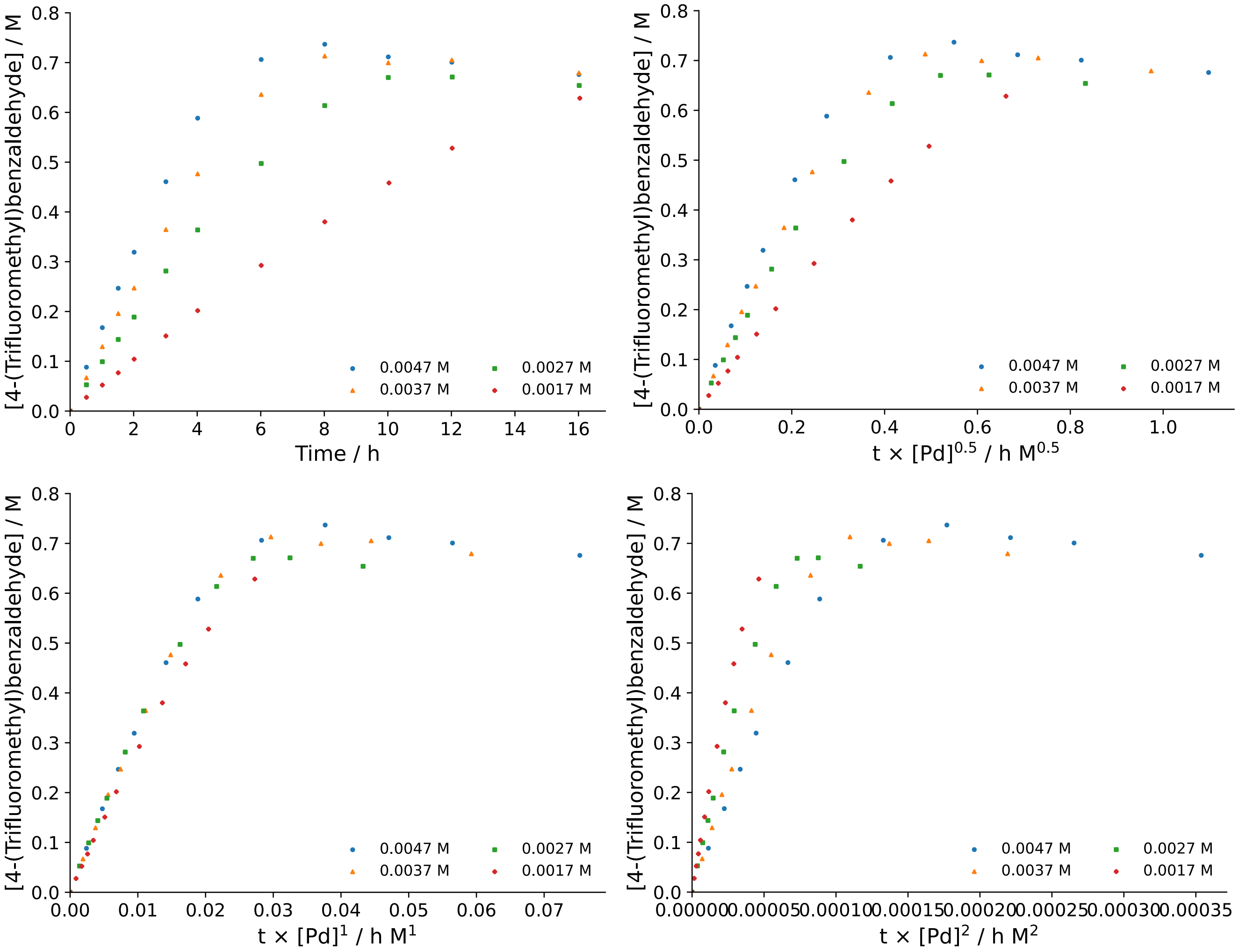

Figure S8. VTNA applied to the concentration-time profiles of 4-(trifluoromethyl)benzaldehyde formation at different concentrations of catalyst. Best overlay is seen with 1st order in catalyst.

Catalyst deactivation (same excess) and product inhibition

Catalyst deactivation and product inhibition were assessed based on the RPKA and VTNA methodologies.[6-8] The same excess reaction has a lower initial concentration of substrate and base in comparison to the standard conditions, reflecting 38% conversion of aryl bromide substrate. To the product inhibition experiment, the corresponding amount of products (aryl aldehyde and TMEDA·2HBr salt) were also added. These reactions were carried out according to general procedure C. The same excess and product inhibition concentration-time profiles were shifted by 2.5 h to consider the time taken to get to 38% conversion under the standard reaction conditions.

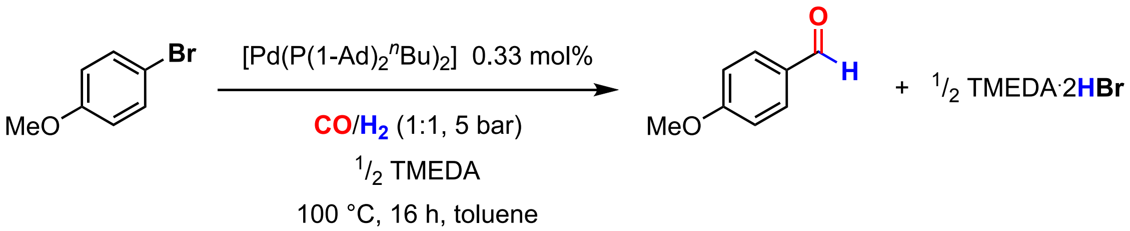

Experiment	[4-MeOC$_6$H$_4$Br]	[TMEDA]	[Catalyst]	[4-MeOC$_6$H$_4$CHO]	[TMEDA·2HBr]	Notes
**1**	0.8 M	0.6 M	0.0027 M	0 M	0 M	Control
**2**	0.5 M	0.45 M	0.0027 M	0 M	0 M	Same excess (without products)
**3**	0.5 M	0.45 M	0.0027 M	0.3 M	0.15 M	Product inhibition (with products)

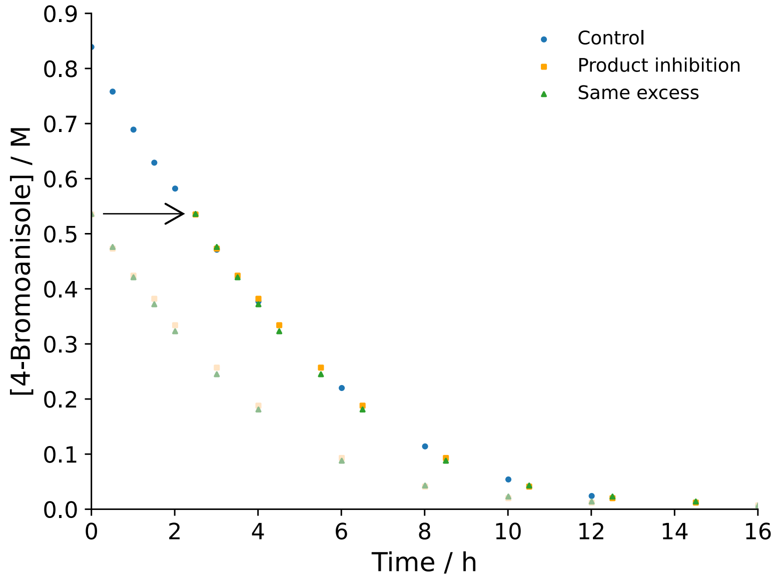

Figure S9. Overlay of concentration-time profiles for 4-bromoanisole consumption for the control reaction under standard conditions, the same excess profile and the and product inhibition profile, both as collected and time-shifted.

All three curves show good overlap, so we can deduce that there is no catalyst deactivation or product inhibition within this kinetic region when using 4-bromoanisole as a substrate.

Order in 4-bromoanisole using VTNA:

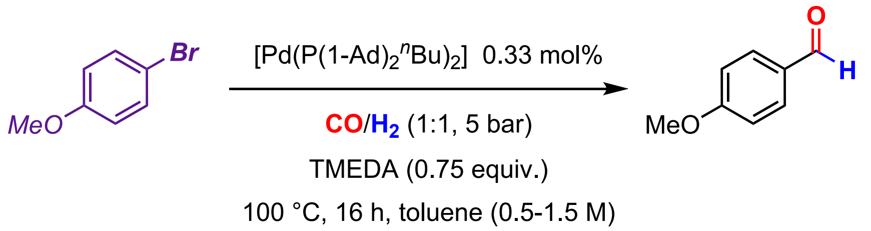

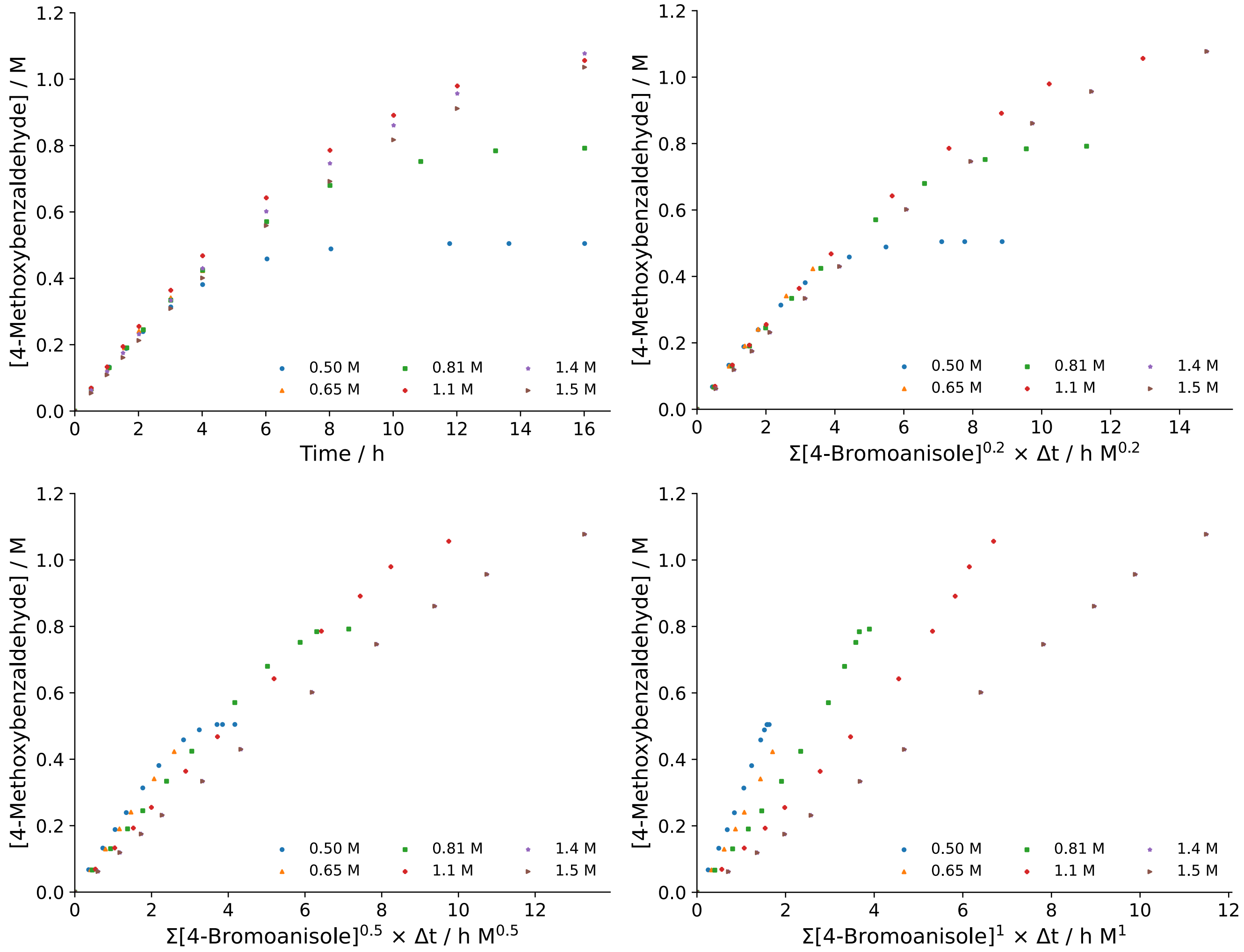

Figure S10. VTNA applied to the concentration-time profiles of 4-methoxybenzaldehyde formation at different concentrations of 4-bromoanisole substrate. Best overlay is seen with zero order in 4-bromoanisole.

Data for 0.65 M 4-bromoanisole concentration were collected up to 3 hours only.

Timecourse profiles at 0.50 M and 0.81 M 4-bromoanisole concentrations plateau due to full consumption of substrate. Experiments at higher concentrations of 4-bromoanisole (1.4 and 1.5 M) confirm good overlap of data sets for a zero order fit at high conversion. Taking this into account, the best overlayed profile is the zero order VTNA plot – which shows good overlap at low conversions (before substrate is consumed).

Order in 4-bromobenzotrifluoride using VTNA:

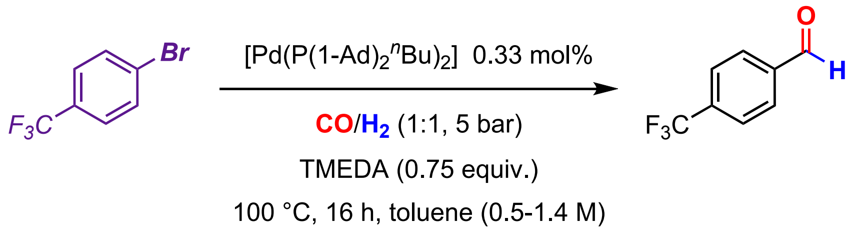

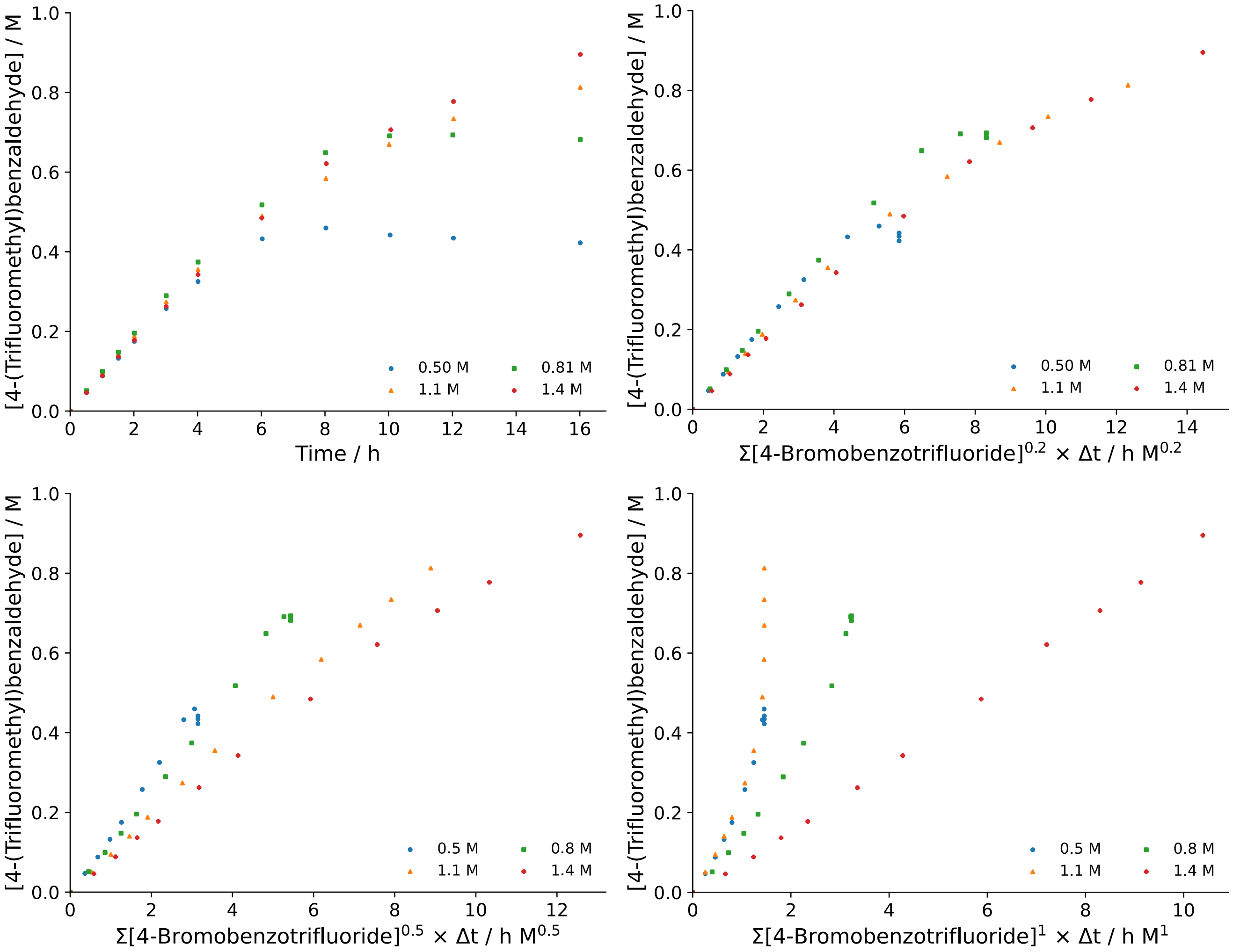

Figure S11. VTNA applied to the concentration-time profiles of 4-(trifluoromethyl)benzaldehyde formation at different concentrations of 4-bromobenzotrifluoride substrate. Best overlay is seen with zero order in substrate.

Timecourse profiles at 0.50 M and 0.81 M 4-bromobenzotrifluoride concentrations plateau due to full consumption of substrate. Experiments at higher concentrations of 4-bromobenzotrifluoride (1.1 and 1.4 M) confirm good overlap of data sets for a zero order fit at high conversion. Taking this into account, the best overlayed profile is the zero order VTNA plot – which shows good overlap at low conversions (before substrate is consumed).

Order in TMEDA using initial rates (4-bromoanisole):

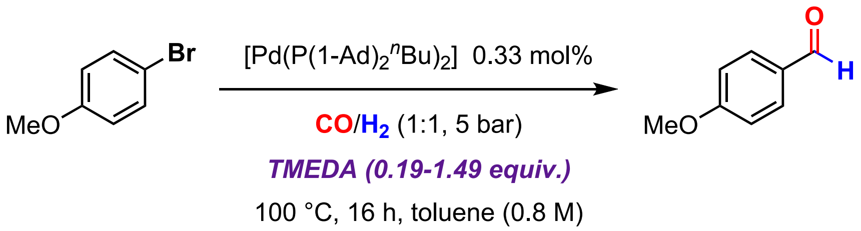

[TMEDA] / M	Initial rate / h^{-1}
0.15	0.0579
0.3	0.0937
0.6	0.1209
0.9	0.1334
1.2	0.1607

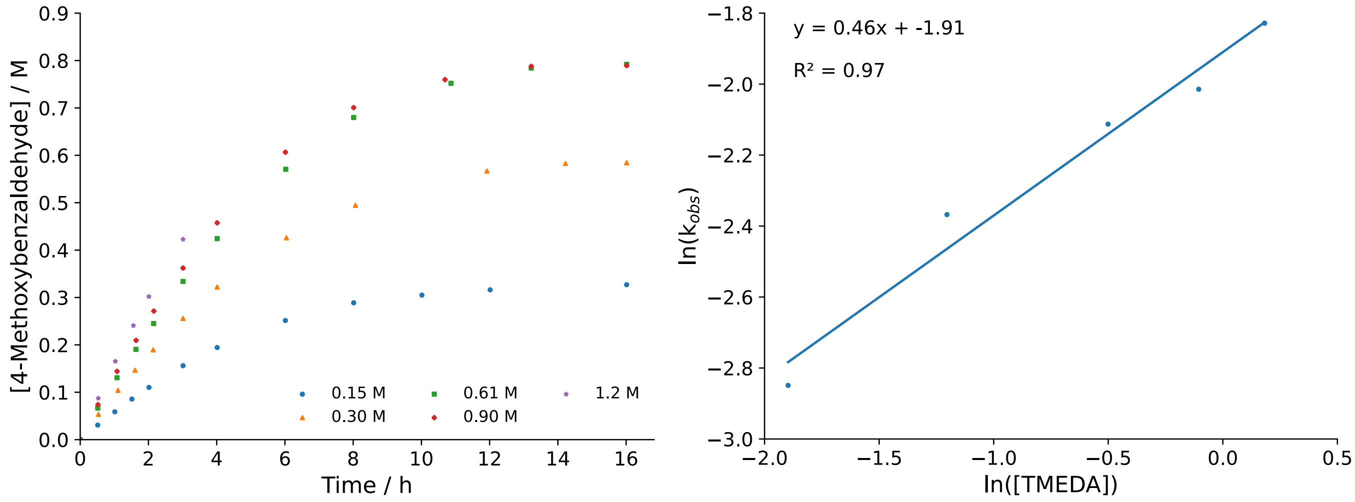

Figure S12. Initial rates method applied to the concentration-time profiles of 4-methoxybenzaldehyde formation at different concentrations of TMEDA. Plot of ln(k_{obs}) against ln([TMEDA]) gives a gradient, and hence order in TMEDA, of 0.46.

Data for 1.2 M TMEDA concentration were collected up to 3 hours only.

Order in Et_3N using initial rates (4-bromoanisole):

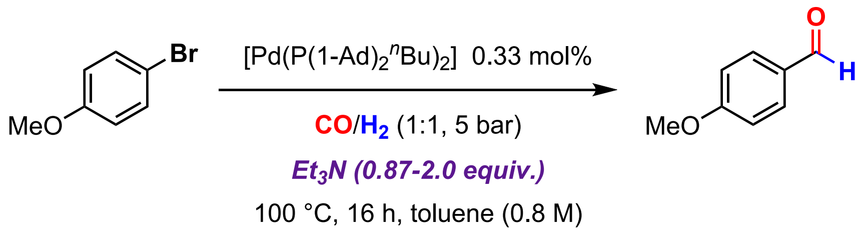

$[Et_3N]$ / M	Initial rate / h^{-1}
0.7	0.0948
0.8	0.0914
1.0	0.1330
1.2	0.1501
1.4	0.1533
1.6	0.1889

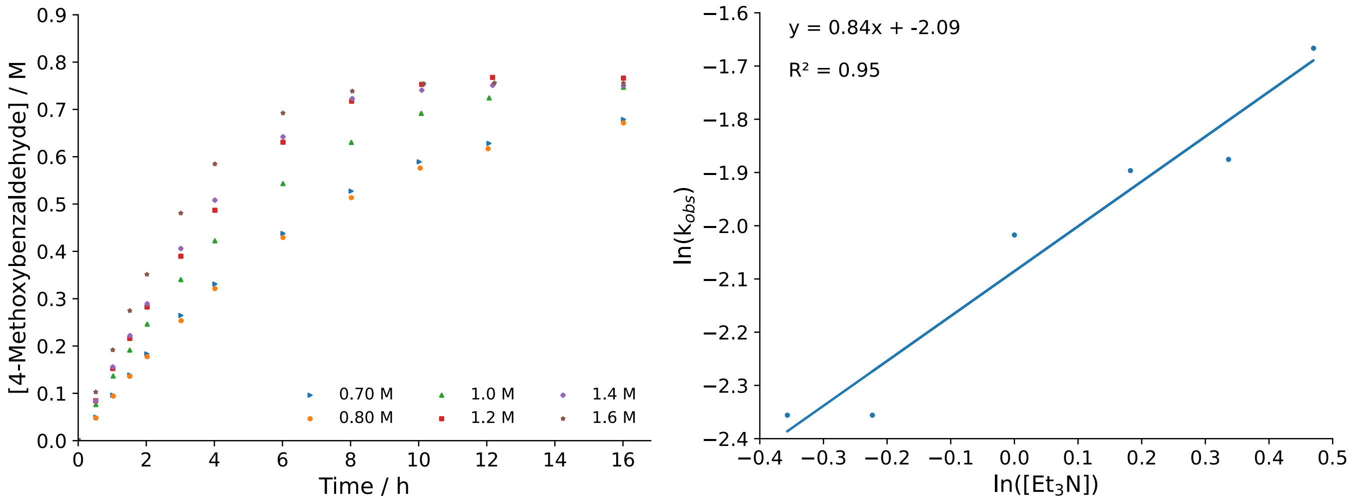

Figure S13. Initial rates method applied to the concentration-time profiles of 4-methoxybenzaldehyde formation at different concentrations of Et_3N. Plot of $\ln(k_{obs})$ against $\ln([Et_3N])$ gives a gradient, and hence order in Et_3N, of 0.84.

Order in TMEDA using initial rates (4-bromobenzotrilfluoride):

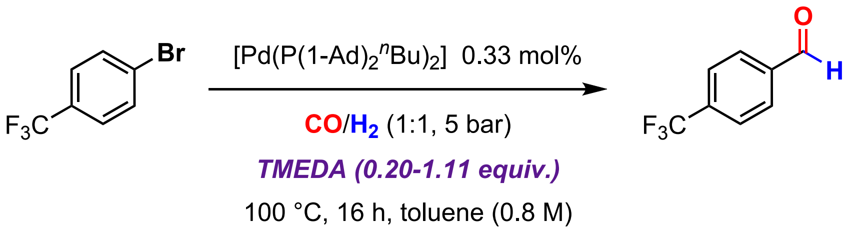

[TMEDA] / M	Initial rate / h^{-1}
0.16	0.0395
0.3	0.0651
0.6	0.0979
0.9	0.1067
1.2	0.1152

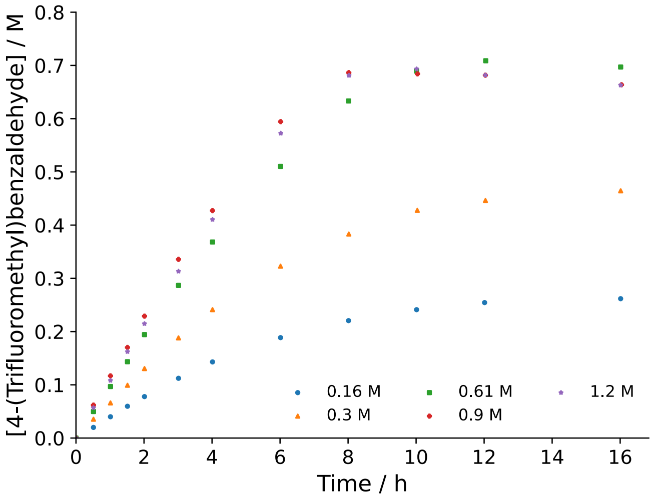

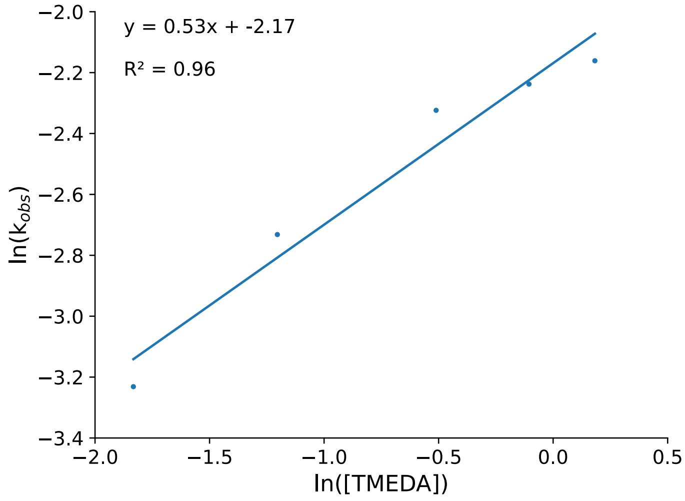

Figure S14. Initial rates method applied to the concentration-time profiles of 4-(trifluoromethyl)benzaldehyde formation at different concentrations of TMEDA. Plot of ln(k_{obs}) against ln([TMEDA]) gives a gradient, and hence order in TMEDA, of 0.53.

Control reactions

For each set of parallel reactions with CO/H_2 (1:1), a control reaction was run in a different reactor each time to ensure reproducibility of the kinetic data.

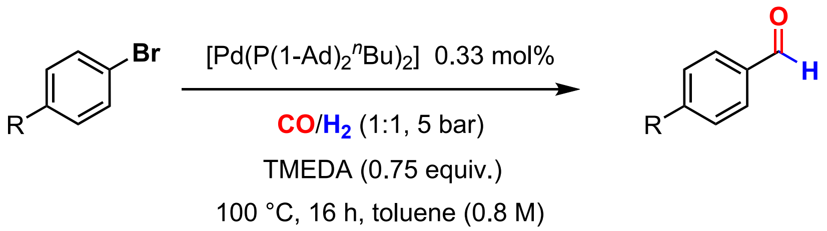

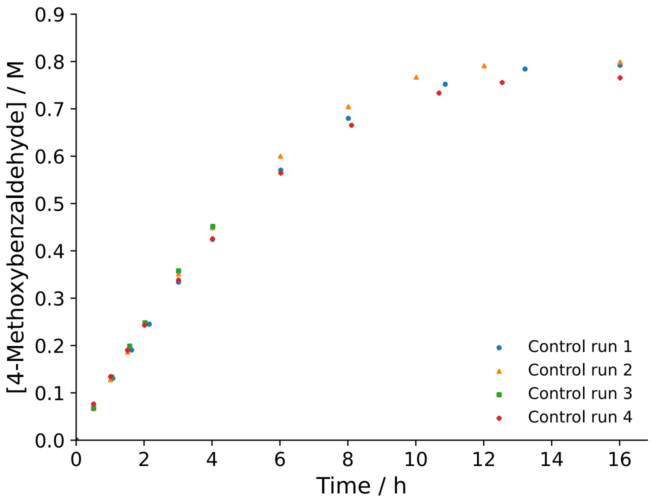

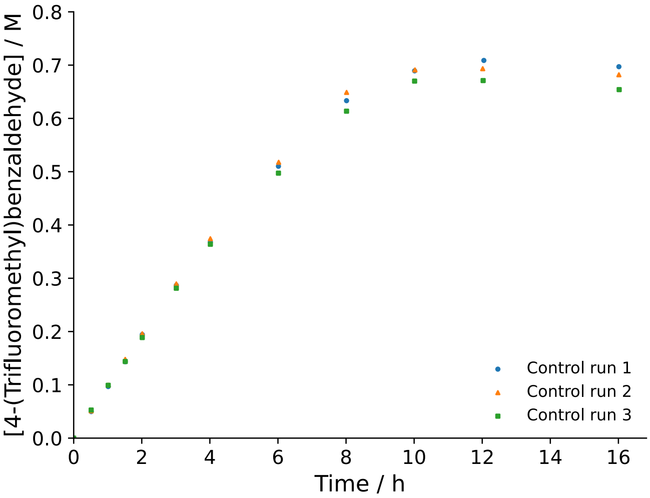

R	Average initial rate / h^{-1}	Standard deviation
OMe	0.1221	0.004
CF_3	0.0961	0.002

Figure S15. Overlay of concentration-time profiles for repeat reactions under standard conditions for 4-methoxybenzaldehyde (left) and 4-(trifluoromethyl)benzaldehyde (right) formation.

Data for control run 3 were collected up to 3 hours only.

Varying gas ratios

General procedure E for the formylation of aryl bromides

In a N_2 purge (glove)box, the Optimisation Sampling Reactor module equipped with curve-bottom glass liners was charged with [Pd(P(1-Ad)$_2{}^n$Bu)$_2$] (0.01 M solution in toluene, 5.42 mL, 0.043 mmol, 0.0033 equiv.), toluene (3.16 mL), hexadecane (1.2 M solution in toluene, 2.71 mL, 3.25 mmol, 0.25 equiv., internal standard), corresponding aryl bromide (4.8 M solution in toluene, 2.71 mL, 13 mmol, 1 equiv.) and TMEDA (3.2 M solution in toluene, 3.05 mL, 9.75 mmol, 0.75 equiv.). Equipped with overhead PEEK stir paddles, the pressure vessel was sealed and purged with N_2 (2 × 7 bar). To determine the initial substrate concentration, a sample (100 μL) was taken from all reactors and deposited into a 96-well sample plate, filled with EtOAc (500 μL) for quenching, and chased with toluene (250 μL). All reactors were then heated to 100 °C, set to stirring speed 800 rpm, then pressurised to 5 bar with CO/H_2/N_2 (0.5:1:0.5 or 1:0.5:0.5). Samples were taken according to the library design at 0.5, 1, 1.5, 2, 3, 4, 6, 8, 10, 12 and 16 h. After this, the reactors were cooled and depressurised. Collected samples were held in a 96-well sample plate, removed from the purge box once all sampling had completed, and diluted with EtOAc (1 mL). Using the OT-2, samples were filtered using a 0.45 μm 96-well filter plate under reduced pressure, then transposed into GC vials, and subject to GC-FID analysis to determine concentrations of substrate, product, and hydrodebrominated side product.

Low P_{CO}

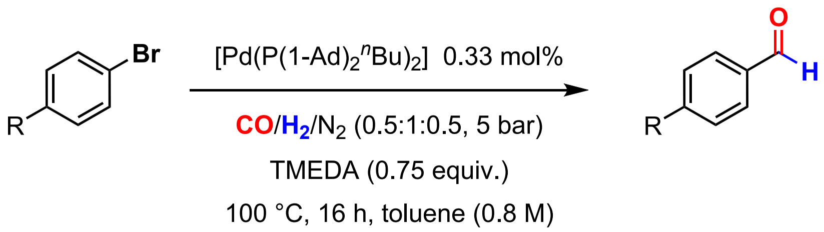

R	Conversion %[a]	Aldehyde yield %[a]	Hydrodebrominated product yield %[a]
CN	82	43	32
CF_3	100	55	27
Cl[b]	98	57	31
OMe	100	58	35
NMe_2	67	47	20

[a]Determined by GC-FID using hexadecane internal standard.
[b]1 % terephthalaldehyde and 3 % benzaldehyde were produced in this reaction.
Figure S16. Yields and conversions for different substrates at low CO partial pressure.

Low P_{H_2}

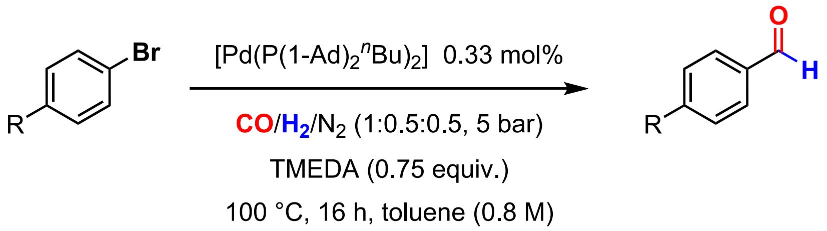

R	Conversion %[a]	Aldehyde yield %[a]	Hydrodebrominated product yield %[a]
CN	37	26	4
CF_3	48	32	2
Cl	44	41	0
OMe	44	44	0
NMe_2	44	44	0

[a]Determined by GC-FID using hexadecane internal standard.

Figure S17. Yields and conversions for different substrates at low H_2 partial pressure.

Table S3. Initial rate values for catalytic formylation of different substrates under standard conditions, low CO partial pressure, and low H_2 partial pressure.

R	σ_p	Initial rate / M h^{-1}	Initial rate low CO / M h^{-1}	Initial rate low H_2 / M h^{-1}
CN	0.66	0.054	0.043	0.025
CF_3	0.54	0.095	0.067	0.043
Cl	0.23	0.11	0.085	0.055
OMe	-0.27	0.12	0.10	0.064
NMe_2	-0.83	0.14	0.12	0.080

Eyring Analysis

General procedure

In a N_2 purge (glove)box, the Optimisation Sampling Reactor module equipped with curve-bottom glass liners was charged with $[Pd(P(1\text{-}Ad)_2{}^nBu)_2]$ (0.01 M solution in toluene, 5.42 mL, 0.043 mmol, 0.0033 equiv.), toluene (3.16 mL), hexadecane (1.2 M solution in toluene, 2.71 mL, 3.25 mmol, 0.25 equiv., internal standard), corresponding aryl bromide (4.8 M solution in toluene, 2.71 mL, 13 mmol, 1 equiv.) and TMEDA (3.2 M solution in toluene, 3.05 mL, 9.75 mmol, 0.75 equiv.). Equipped with overhead PEEK stir paddles, the pressure vessel was sealed and purged with N_2 (2×7 bar). To determine the initial substrate concentration, a sample (100 μL) was taken from all reactors and deposited into a 96-well sample plate, filled with EtOAc (500 μL) for quenching, and chased with toluene (250 μL). Four reactors were then set to 90 °C, 95 °C, 100 °C and 105 °C respectively, set to stirring speed 800 rpm, then pressurised to 5 bar with CO/H_2 (1:1). Samples were taken according to the library design at 0.5, 1, 1.5, 2, 3, and 4 h. After this, the reactors were cooled and depressurised. Collected samples were held in a 96-well sample plate, removed from the purge box once all sampling had completed, and diluted with EtOAc (1 mL). Using the OT-2, samples were filtered using a 0.45 μm 96-well filter plate under reduced pressure, then transposed into GC vials, and subject to GC-FID analysis to determine concentrations of substrate and product.

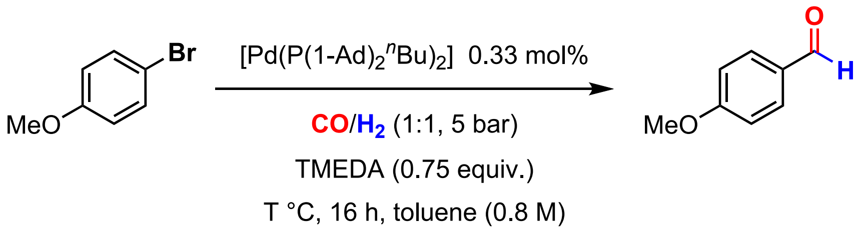

Temperature / K	1/T	k_{obs} / x 10^{-5} s^{-1}	ln(k/T)
363.15	0.00275	1.71	-16.87
368.15	0.00272	2.66	-16.44
373.15	0.00268	3.55	-16.17
378.15	0.00264	4.31	-15.99

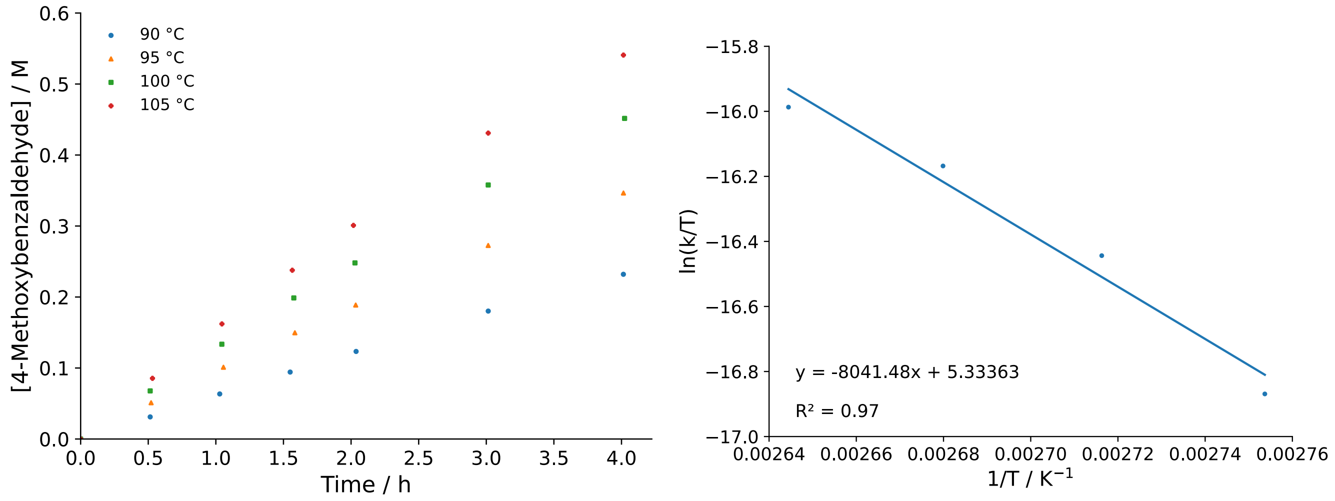

Activation parameter	Value
**$\Delta H^{\ddagger}$ / kcal mol^{-1}**	+16.0
**$\Delta S^{\ddagger}$ / cal mol^{-1} K^{-1}**	-36.6
**$\Delta G_{373K}^{\ddagger}$ / kcal mol^{-1}**	29.6

Figure S18. Overlay of concentration-time profiles of 4-methoxybenzaldehyde formation at different temperatures, and the corresponding Eyring plot. Table below describes activation parameters found using the Eyring plot.

Four rate constants were determined using the initial rates method. A plot of ln(k_{obs}/T) against 1/T allowed calculation of the thermodynamic parameters using the Eyring equation.

Kinetic Isotope Effect

General procedure

In a N_2 purge (glove)box, the Optimisation Sampling Reactor module equipped with curve-bottom glass liners was charged with [Pd(P(1-Ad)$_2$^{n}Bu)$_2$] (0.01 M solution in toluene, 5.42 mL, 0.043 mmol, 0.0033 equiv.), toluene (3.16 mL), hexadecane (1.2 M solution in toluene, 2.71 mL, 3.25 mmol, 0.25 equiv., internal standard), corresponding aryl bromide (4.8 M solution in toluene, 2.71 mL, 13 mmol, 1 equiv.) and TMEDA (3.2 M solution in toluene, 3.05 mL, 9.75 mmol, 0.75 equiv.). Equipped with overhead PEEK stir paddles, the pressure vessel was sealed and purged with N_2 (2 × 7 bar). To determine the initial substrate concentration, a sample (100 µL) was taken from all reactors and deposited into a 96-well sample plate, filled with EtOAc (500 µL) for quenching, and chased with toluene (250 µL). All reactors were then heated to 100 °C, set to stirring speed 800 rpm, then pressurised to 5 bar with CO/D_2 (1:1). Samples were taken according to the library design at 0.5, 1, 1.5, 2, 3, 4, 6, 8, 10, 12 and 16 h. After this, the reactors were cooled and depressurised. Collected samples were held in a 96-well sample plate, removed from the purge box once all sampling had completed, and diluted with EtOAc (1 mL). Using the OT-2, samples were filtered using a 0.45 µm 96-well filter plate under reduced pressure, then transposed into GC vials, and subject to GC-FID analysis to determine concentrations of substrate and product. ^{1}H NMR spectroscopic analysis was carried out on the final reaction mixtures to ensure 100% D-incorporation.

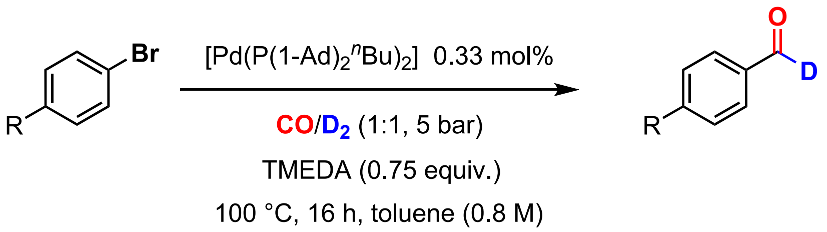

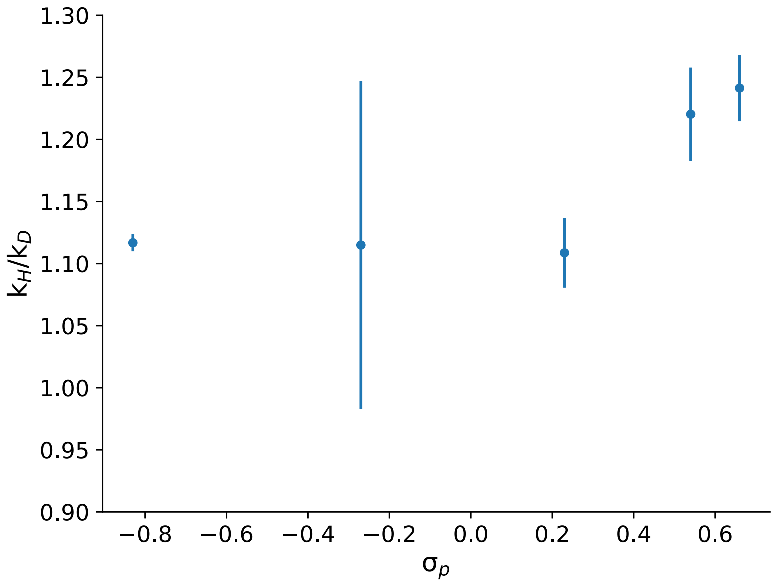

R	σ_p	KIE k_H/k_D
CN	0.66	1.2 ± 0.03
CF_3	0.54	1.2 ± 0.04
Cl	0.23	1.1 ± 0.03
OMe	-0.27	1.1 ± 0.13
NMe_2	-0.83	1.1 ± 0.01

Figure S19. k_H/k_D for different substrates when using CO/D_2 instead of CO/H_2. Values are plotted as the mean of two experimental values, with error bars showing the standard deviation.

3. Computational details

Methods

DFT calculations for geometry optimisations were carried out using Gaussian 09 (Revision D.01).[9] Geometry optimisations were performed using the ωB97x-D density functional.[10] A functional screening was performed using the following functionals: B3PW91,[11,12] PBE0,[13] M06L[14] and M062X,[15] including dispersion corrections described by Grimme's D3 corrections,[16] with added Becke-Johnson dampening where appropriate.[17-20] Metal atoms (Pd) were described with Stuttgart SDDAll RECPs and associated basis sets,[21] while a hybrid basis set was used for the other atoms: def2-svp (C, H), def2-tzvp(p) (O, N, P, F, Br, Cl), def2-tzvp(p)d (Pd–***H***) (BS1).[22] Def2-tzvp(p)d is not natively incorporated into the Gaussian suite of programs, and so this basis set needed to be defined manually. The specific function for metal hydrides was obtained from the Basis Set Exchange (https://www.basissetexchange.org/).[23-25] Each metal hydride was defined as follows:

```
Hydrogen:
S   3   1.00
   34.0613410          0.60251978D-02
    5.1235746          0.45021094D-01
    1.1646626          0.20189726
S   1   1.00
    0.32723041          1.0000000
S   1   1.00
    0.10307241          1.0000000
P   1   1.00
    1.40700000          1.0000000
P   1   1.00
    0.38800000          1.0000000
P   1   1.00
    0.95774129632D-01     1.0000000
D   1   1.00
    1.05700000          1.0000000
```

Geometry optimisation calculations were performed without symmetry constraints and an ultrafine integrations grid (keyword int=ultrafine). Solvent effects were incorporated into the optimization process using the SMD solvation model in toluene.[26]

Frequency analyses for all stationary points were performed to confirm the nature of the structures as either minima (no imaginary frequency) or transition states (only one imaginary frequency). Intrinsic reaction coordinate (IRC) calculations followed by full geometry optimisations on final points were used to connect transition states and minima located on the potential energy surface allowing a full energy profile (calculated at 298.15 K, 1 atm) of the reaction to be constructed. The graphical user interface used to visualise the various properties of the intermediates and transition states was GaussView 6.0.16.[27]

Conformer searches of intermediates were conducted with CREST version 2.12 using default settings,[28,29] and CENSO version 1.2.0 including thermal correction to 373.15 K.[30] The lowest energy conformers were subjected to additional DFT calculations. Manual conformer searches were conducted for transition states.

Thermal corrections at the same level of theory as geometry optimisations were applied using the *GoodVibes* program,[31] which incorporates Grimme's quasi-harmonic approximation to the vibrational entropy below a cut-off of 100 cm^{-1} and a frequency scaling factor = 1.0. The entropic terms for the frequencies below the

cut-off are obtained from the free-rotor approximation while the standard rigid-rotor harmonic oscillator (RRHO) approximation is retained for those above the cut-off. A dampening function is used to interpolate between these two expressions close to the cut-off frequency. To account for experimental conditions, a temperature of 373.15 K and concentrations of 0.8 mol L^{-1} (aryl bromide), 1.2 mol L^{-1} (NMe_3), 0.02 mol L^{-1} (CO), 0.01 mol L^{-1} (H_2) and 0.0027 mol L^{-1} (catalytic species) were used. The concentrations of CO and H_2 were calculated using experimentally determined solubility constants at 373.15 K with toluene as a solvent.[32] The dissolved gases were assumed to obey Henry's Law.

Single point energies were calculated using ORCA 5.0.3,[33,34] with the ωB97x-D4 density functional[35,36] and SMD solvation model in toluene, and all atoms were modelled with the def2-tzvp(p)d basis set (BS2). The corresponding ECP was used for Pd. Calculations were performed with the resolution of identity approximation for the Coulomb integrals, and chain of spheres approximation for the exchange integrals (RIJCOSX) with the def2/j auxiliary basis set.

Natural bond order (NBO) analysis was performed with BS1, using NBO version 6.0.[37,38]

Oxidative addition pathway

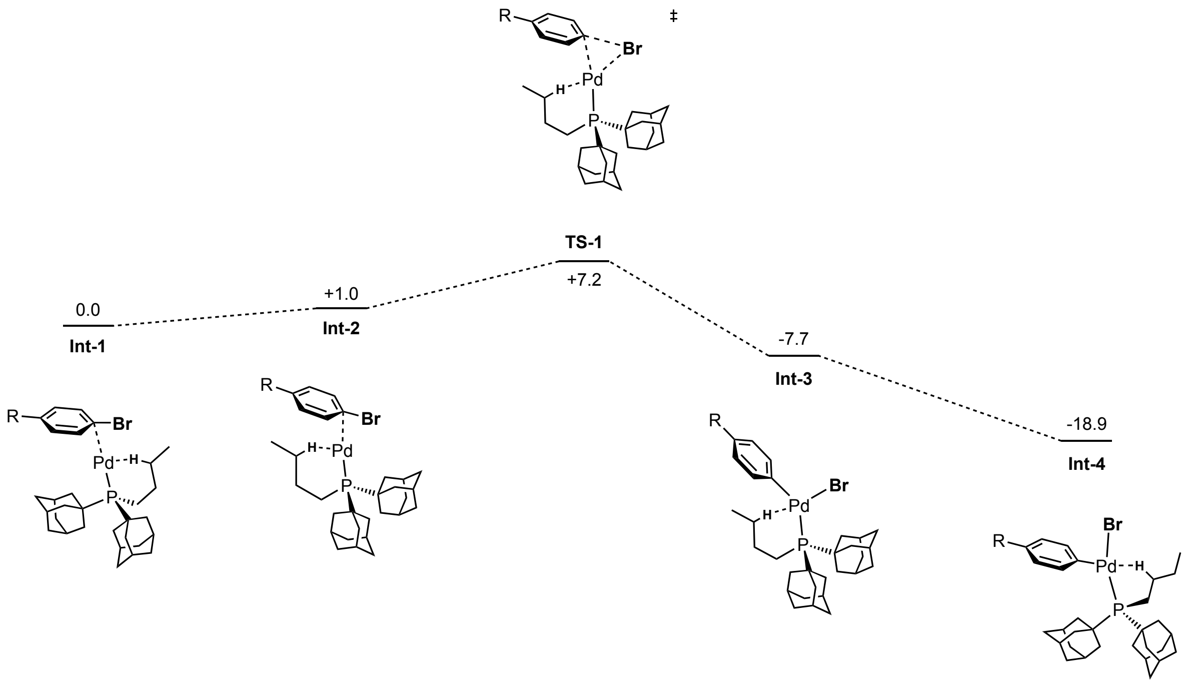

R	Int-1	Int-2	TS-1	$\Delta G^{\ddagger}_{TS-1}$	Int-3	Int-4
CN	0.0	-0.3	+5.1	**5.4**	-10.3	-23.4
CF_3	0.0	+0.8	+6.9	**6.9**	-8.5	-19.6
Cl	0.0	+1.9	+7.9	**7.9**	-7.7	-18.9
H	0.0	+1.9	+7.8	**7.8**	-6.5	-17.3
Me	0.0	+1.9	+8.2	**8.2**	-6.4	-18.0
OMe	0.0	+1.0	+7.2	**7.2**	-7.7	-18.9
NMe_2	0.0	+0.8	+8.3	**8.3**	-6.4	-17.2

Figure S20. DFT calculated pathway for the oxidative addition of 4-bromoanisole and other Hammett substrates to the active catalytic species. Energy values reported in kcal mol^{-1}.

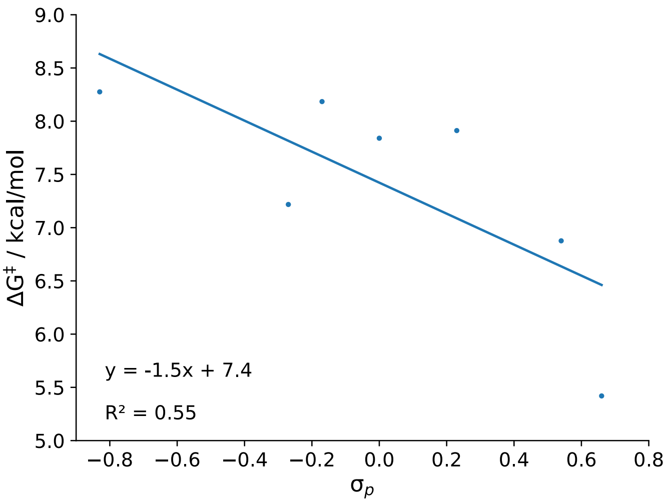

Figure S21. Free energy relationship between σ_p and activation barrier $\Delta G^{\ddagger}_{373K}$ for oxidative addition.

Migratory insertion pathway

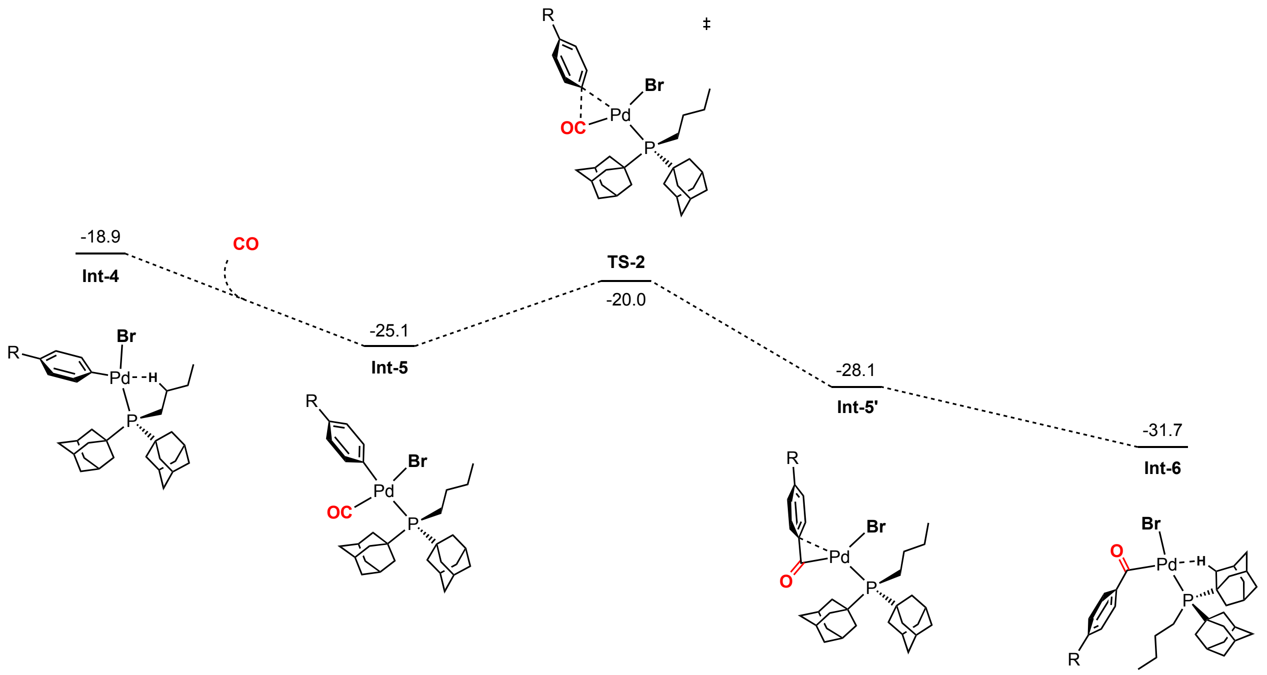

R	Int-4	Int-5	TS-2	$\Delta G^{‡}_{TS-2}$	Int-5'	Int-6
CN	-23.4	-26.1	-16.4	**9.6**	-25.3	-29.2
CF_3	-19.6	-25.2	-15.7	**9.5**	-22.5	-27.7
Cl	-18.9	-24.9	-16.9	**8.0**	-23.9	-28.7
H	-17.3	-24.2	-17.4	**6.8**	-24.3	-28.4
Me	-18.0	-23.9	-17.6	**6.3**	-25.2	-29.4
OMe	-18.9	-25.1	-20.0	**5.2**	-28.1	-31.7
NMe_2	-17.2	-23.9	-20.4	**3.5**	-29.5	-32.3

Figure S22. DFT calculated pathway for the migratory insertion of CO to **Int-4**. Energy values reported in kcal mol^{-1}.

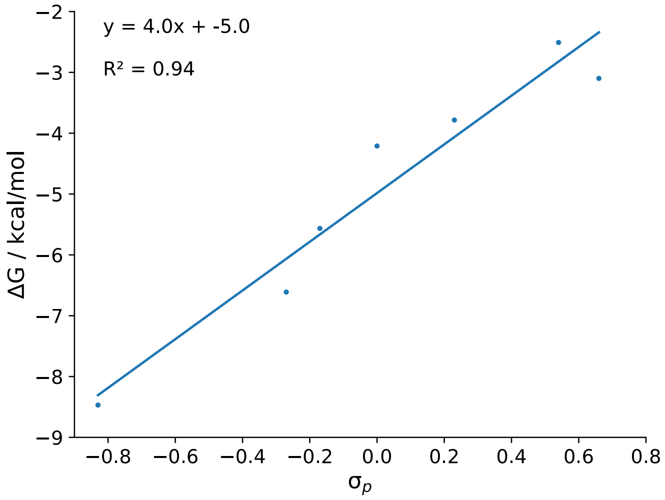

Figure S23. Free energy relationship between σ_p and free energy change ΔG_{373K} for migratory insertion.

Plausible alternative geometries of **Int-5** and **TS-2** involve the phosphine *trans* to the carbonyl (**Figure S24**). While **Int-S1** for all substrates are similar in energy to **Int-5**, transition states (**TS-S1**) are higher in energy than **TS-2** and thus this pathway is likely not operating.

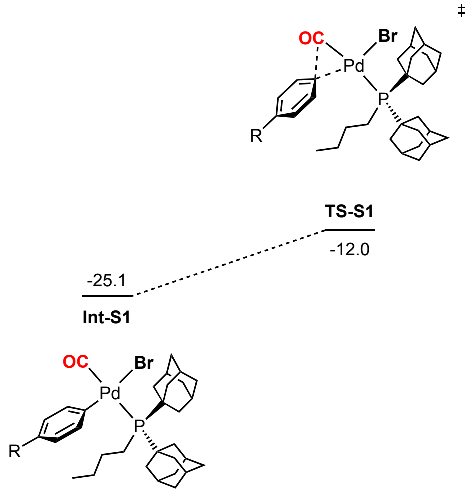

R	Int-S1	TS-S1	$\Delta G^{\ddagger}_{TS\text{-}S1}$
CN	-26.3	-7.4	**18.8**
CF_3	-25.3	-7.0	**18.3**
Cl	-24.7	-8.4	**16.3**
H	-23.7	-8.0	**15.7**
Me	-23.9	-9.0	**14.9**
OMe	-25.1	-12.0	**13.1**
NMe_2	-24.3	-13.0	**11.2**

Figure S24. DFT calculated pathway for the migratory insertion of CO from **Int-S1**. Energy values reported in kcal mol^{-1}.

Dihydrogen activation lowest energy pathway

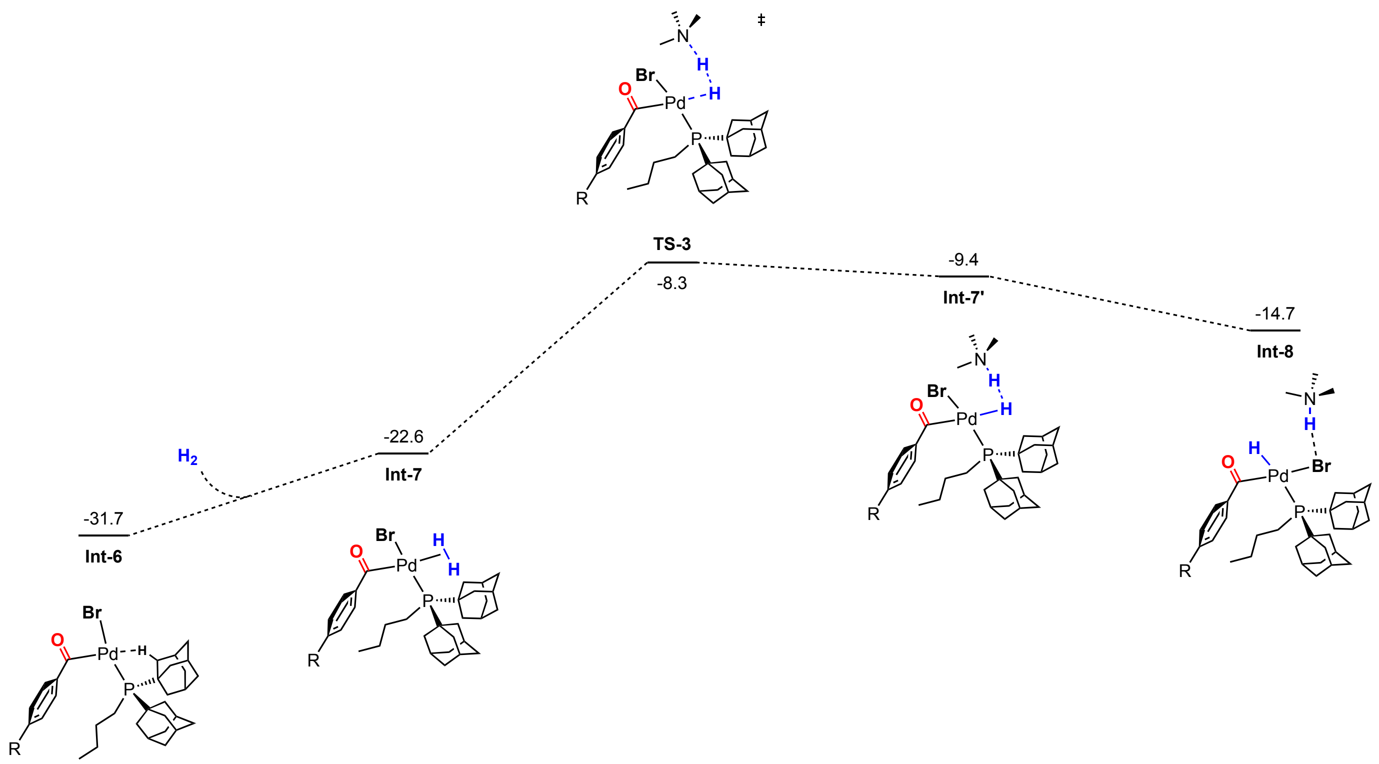

R	Int-6	Int-7	TS-3	$\Delta G^{\ddagger}_{TS-3}$	Int-7'	Int-8
CN	-29.2	-20.0	-7.0	**22.1**	-10.0	-14.3
CF_3	-27.7	-19.1	-5.8	**21.9**	-8.4	-12.5
Cl	-28.7	-19.8	-6.1	**22.6**	-8.2	-13.1
H	-28.4	-18.9	-5.1	**23.3**	-7.1	-12.1
Me	-29.4	-20.0	-5.5	**23.9**	-6.7	-12.8
OMe	-31.7	-22.6	-8.3	**23.4**	-9.4	-14.7
NMe_2	-32.3	-22.2	-5.9	**26.4**	-8.1	-14.6

Figure S25. DFT calculated pathway for base-assisted dihydrogen splitting. Energy values reported in kcal mol^{-1}. An assumption has been made that there is a low energy transition state for the cis/trans isomerisation of **Int-7'** to **Int-8**.

Int-8 may also be considered with a proton-hydride interaction instead of a proton-bromide interaction:

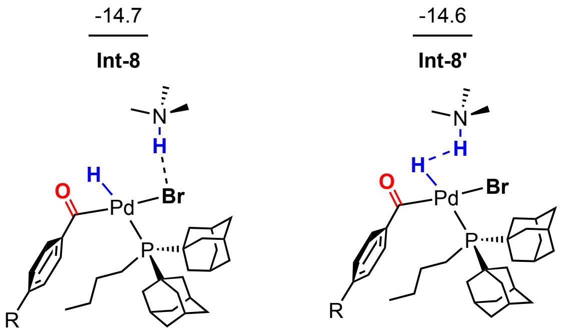

Figure S26. Possible isomerisation of **Int-8** (R = OMe). Energy values reported in kcal mol^{-1}.

A plausible alternative geometry of **TS-3** involves the phosphine *trans* to the hydride (**Figure S27**, **TS-S2**). This transition state is 0.9 kcal mol^{-1} higher in energy than with acyl *trans* to the hydride, so is likely competitive and also occurring.

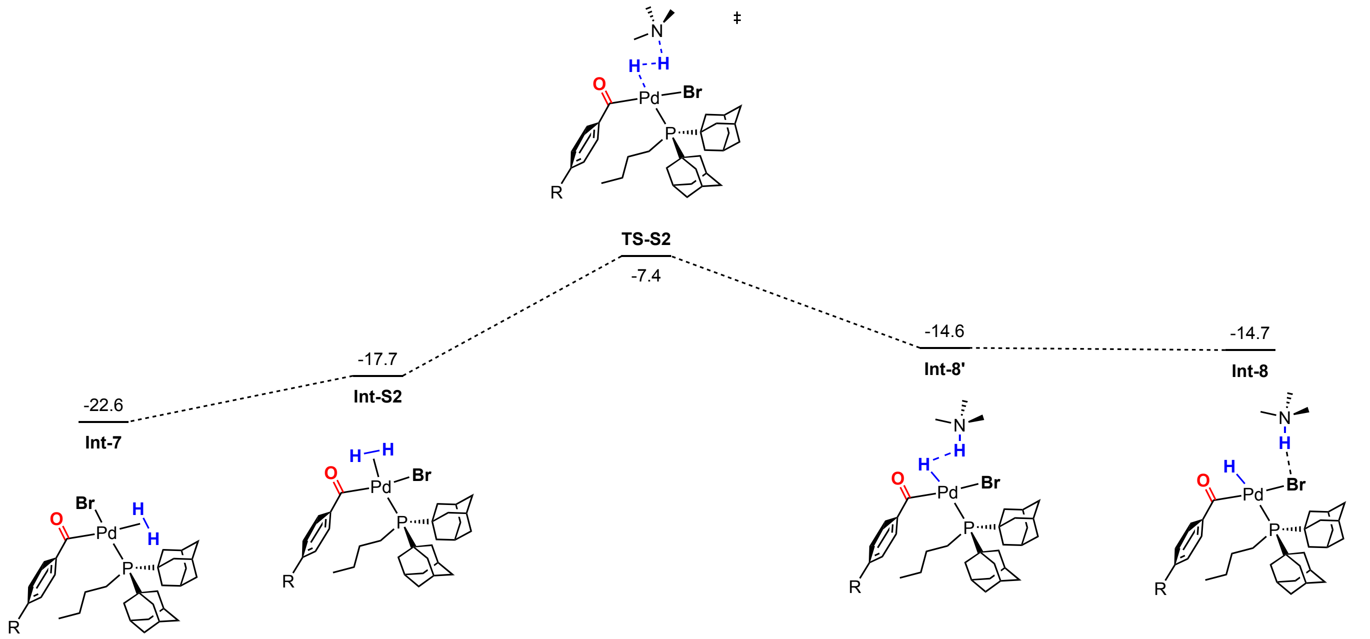

Figure S27. DFT calculated pathway involving an alternative **TS-3** isomer (**TS-S2**) for the dihydrogen activation step (R = OMe). Energy values reported in kcal mol^{-1}.

When considering the geometry where the bromide is *trans* to the hydride (**Figure S28**), the energy gap increases by 8.2 kcal mol^{-1}, and hence this pathway is unlikely to occur.

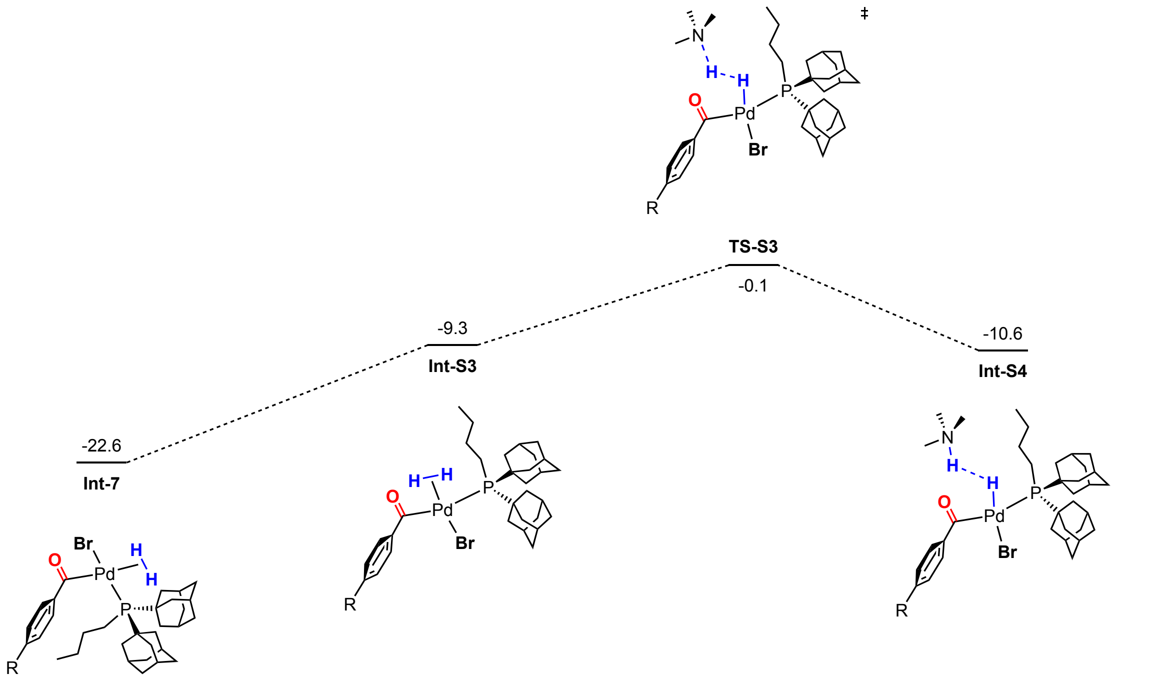

Figure S28. DFT calculated pathway involving an alternative **TS-3** isomer (**TS-S3**) for the dihydrogen activation step (R = OMe). Energy values reported in kcal mol^{-1}.

Alternative mechanisms considered for dihydrogen activation

A. Deprotonation of the σ-dihydrogen palladium complex with Br^-. This pathway has an energy gap of 25.8 kcal mol^{-1} before reaching the transition state, which is already 2.4 kcal mol^{-1} higher in energy than the lowest energy pathway (**Int-6** to **TS-3**). Therefore, this pathway can be discounted.

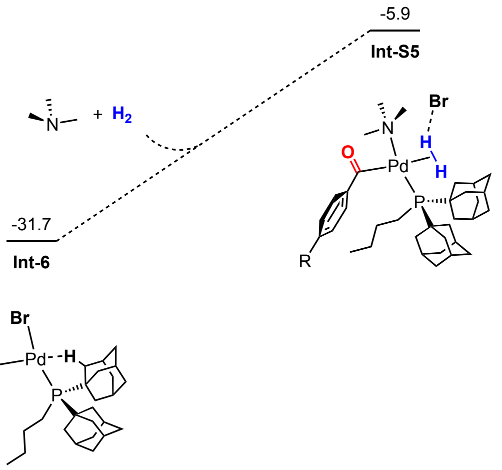

Figure S29. DFT calculated pathway involving NMe_3 displacement of Br^- and subsequent deprotonation of the σ-dihydrogen palladium complex with Br^- for the dihydrogen activation step (R = OMe). Energy values reported in kcal mol^{-1}.

B. Concerted dihydrogen activation and reductive elimination of aryl aldehyde. This pathway has an energy gap of 29.8 kcal mol^{-1} (**Int-6** to **TS-S4**) which is 6.4 kcal mol^{-1} higher in energy than the lowest energy pathway (**Int-6** to **TS-3**). Therefore, this pathway can be discounted.

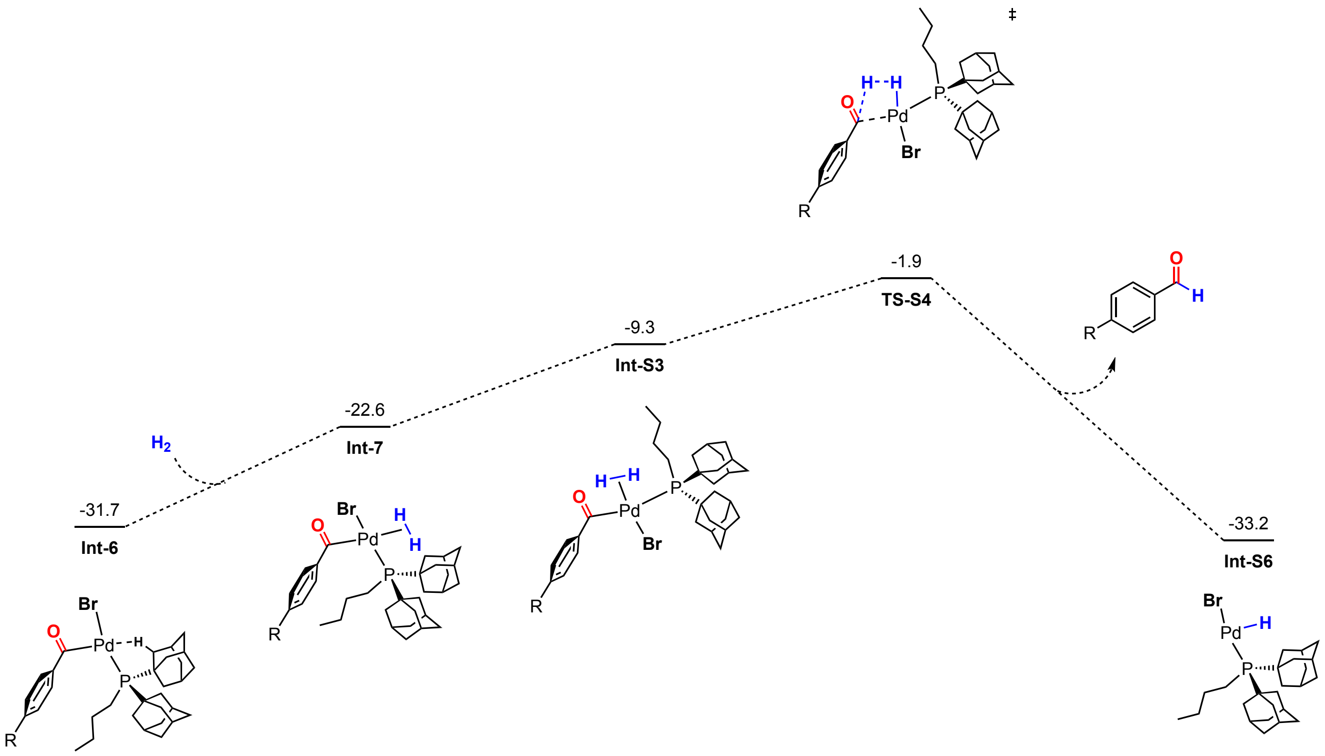

Figure S30. DFT calculated pathway involving base-free concerted dihydrogen splitting and reductive elimination of aryl aldehyde for the dihydrogen activation step (R = OMe). Energy values reported in kcal mol^{-1}.

Reductive elimination of aldehyde product

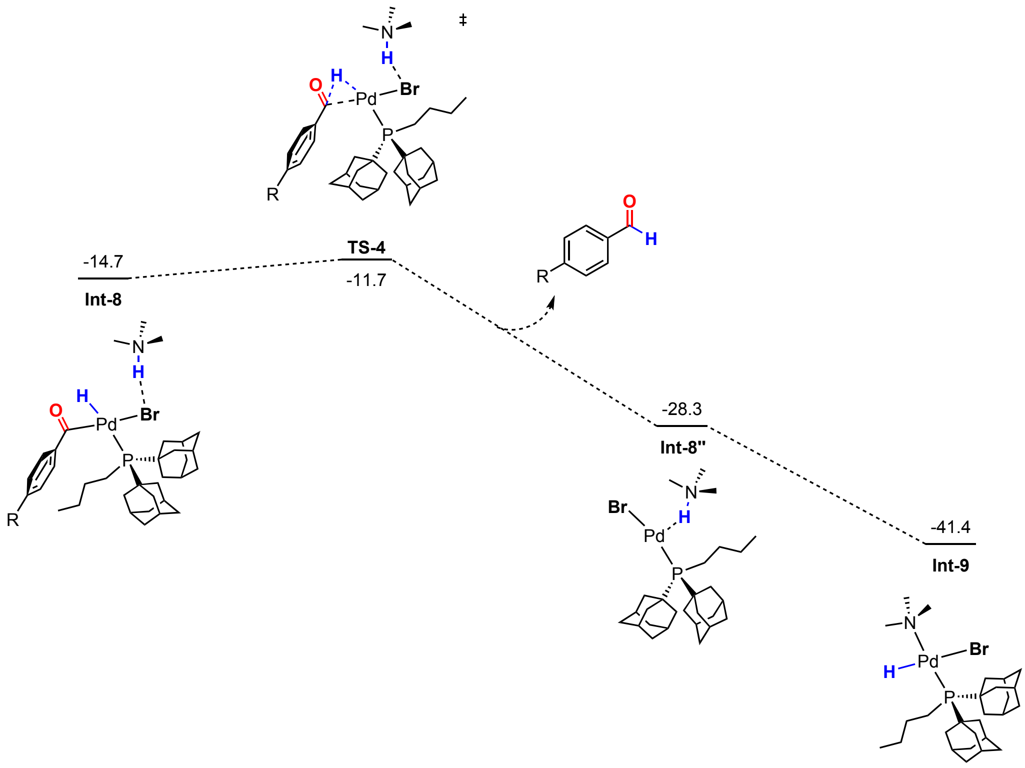

R	Int-8	TS-4	$\Delta G^{\ddagger}_{TS-4}$	Int-8''	Int-9
CN	-14.3	-11.6	**2.7**	-24.3	-37.4
CF_3	-12.5	-10.1	**2.4**	-24.2	-37.2
Cl	-13.1	-9.7	**3.4**	-24.3	-37.3
H	-12.1	-12.1	**2.6**	-24.0	-37.1
Me	-12.8	-9.2	**3.6**	-25.3	-38.3
OMe	-14.7	-11.7	**3.0**	-28.3	-41.4
NMe_2	-14.6	-11.5	**3.1**	-28.4	-41.5

Figure S31. DFT calculated pathway for reductive elimination of aryl aldehyde and regeneration of catalyst. Energy values reported in kcal mol^{-1}.

Int-9 may also be considered with a proton-bromide interaction instead of a proton-palladium interaction:

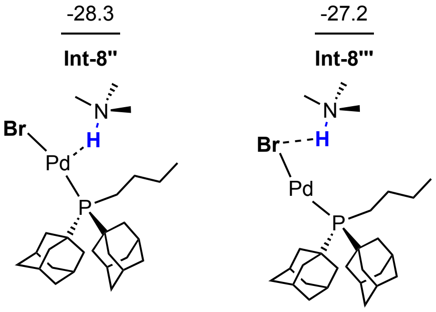

Figure S32. Possible isomerisation of **Int-8''**. Energy values reported in kcal mol^{-1}.

When considering the geometry where the bromide is *trans* to the hydride (**Figure S33**), the energy gap increases to 19.8 kcal mol^{-1}, and hence this pathway is unlikely to occur.

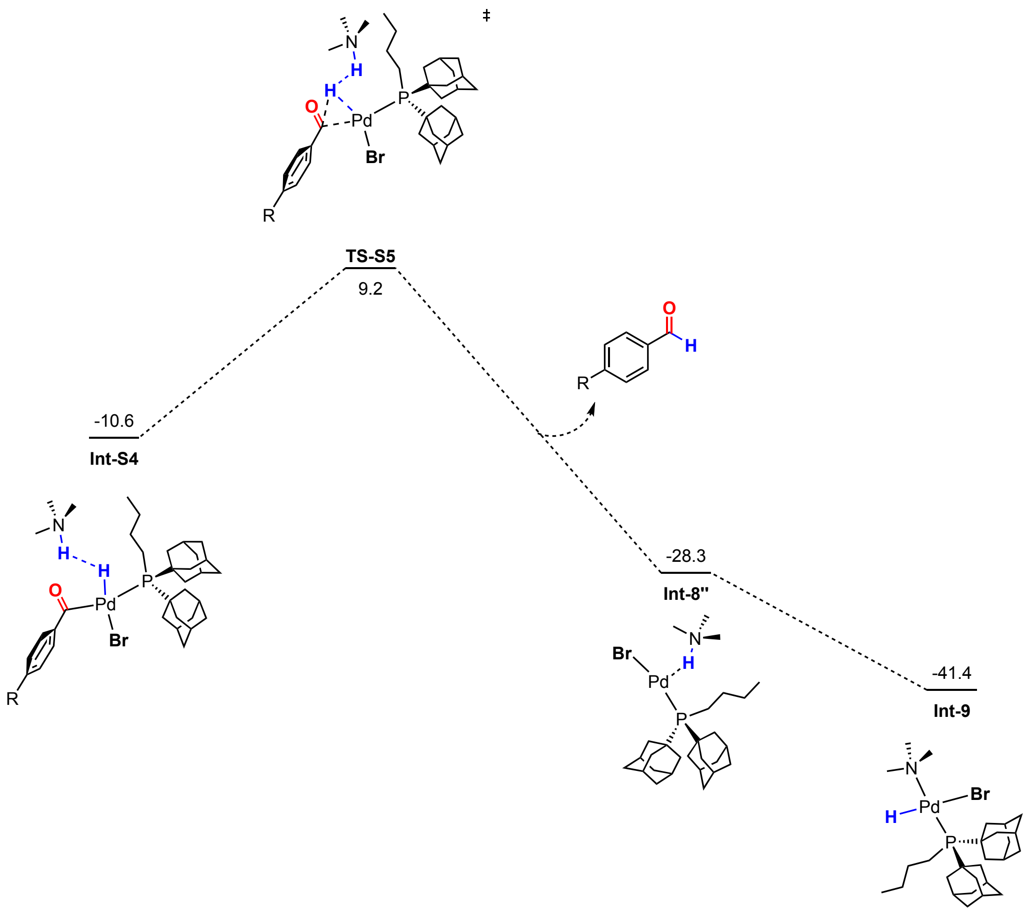

Figure S33. DFT calculated pathway for reductive elimination of aryl aldehyde involving an alternative **TS-4** isomer (**TS-S5**, R = OMe). Energy values reported in kcal mol^{-1}.

Alternative mechanisms considered for reductive elimination

A. Reductive elimination of aryl aldehyde from three-coordinate palladium-acyl-hydride complex. This pathway has an energy gap of 9.2 kcal mol^{-1} (**Int-8** to **TS-S6**), which is 6.2 kcal mol^{-1} higher in energy than the lowest energy pathway (**Int-8** to **TS-4**). Therefore, this pathway can be discounted.

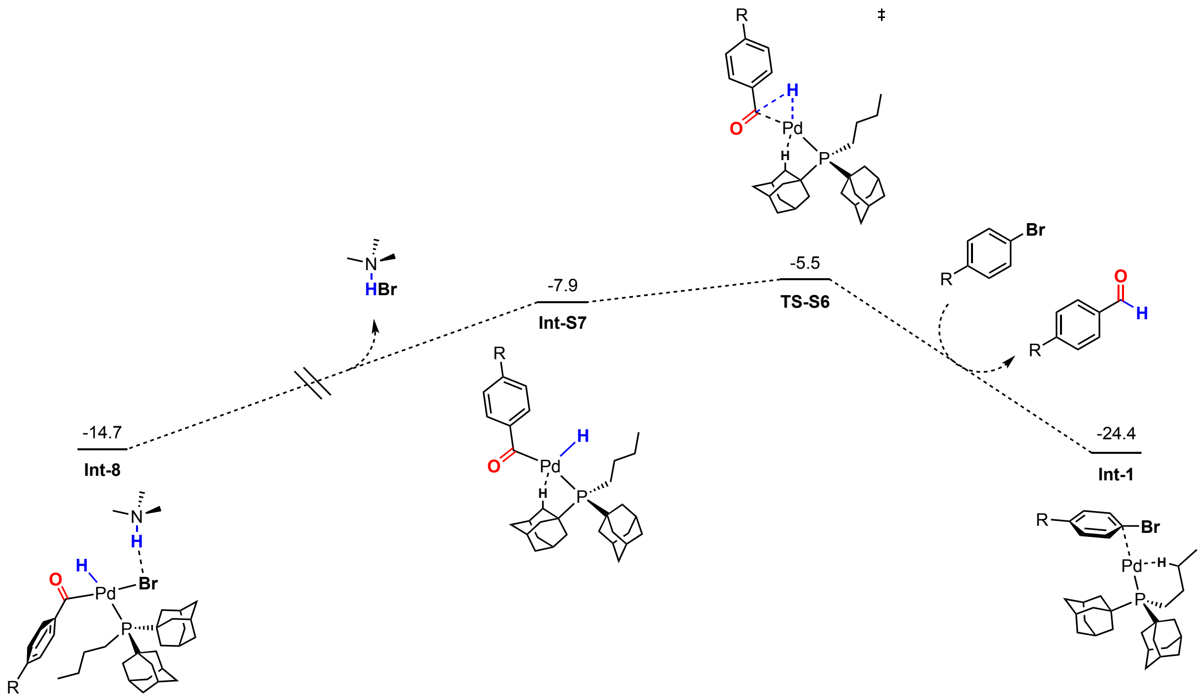

Figure S34. DFT calculated pathway for reductive elimination of aryl aldehyde from a three-coordinate palladium-acyl-hydride complex and regeneration of catalyst. Energy values reported in kcal mol^{-1}.

B. Reductive elimination *via* amine proton shuttle. This pathway has an energy gap of 18.2 kcal mol^{-1} (**Int-S4** to **TS-S7**), which is 15.2 kcal mol^{-1} higher in energy than the lowest energy pathway (**Int-8** to **TS-4**). Therefore, this pathway can be discounted.

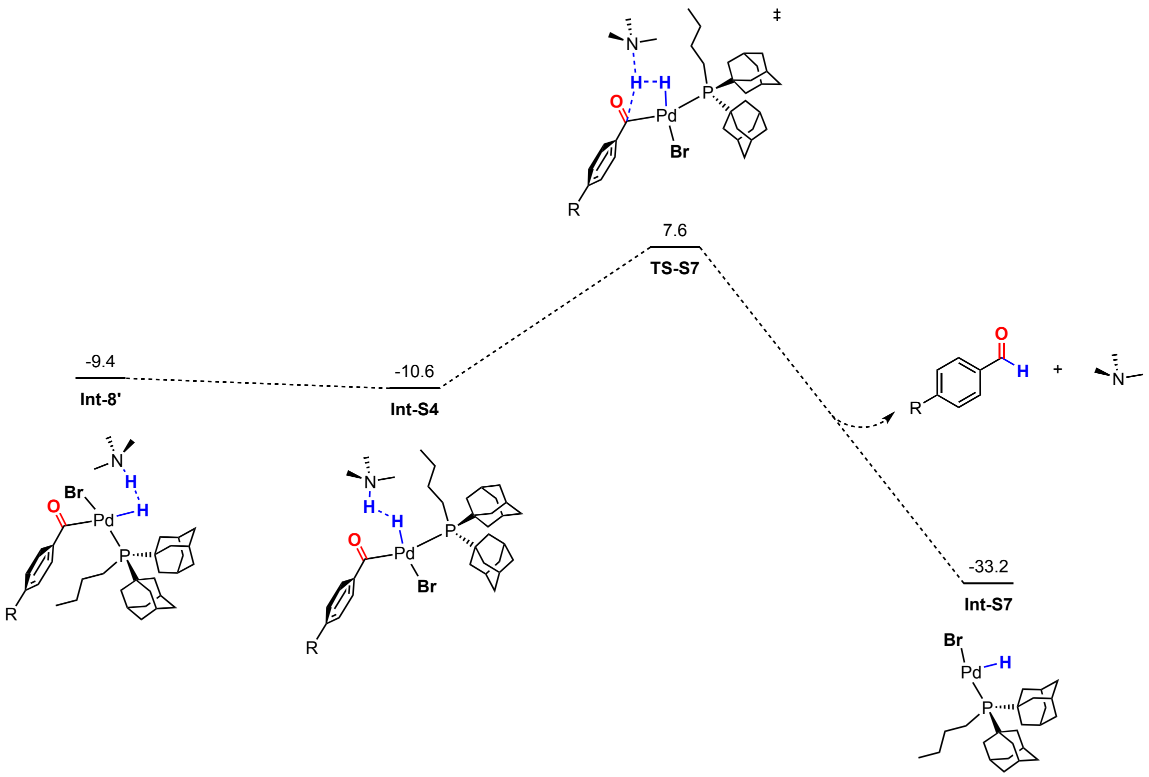

Figure S35. DFT calculated pathway for reductive elimination of aryl aldehyde *via* NMe_3 acting as a proton shuttle. Energy values reported in kcal mol^{-1}.

Functional testing

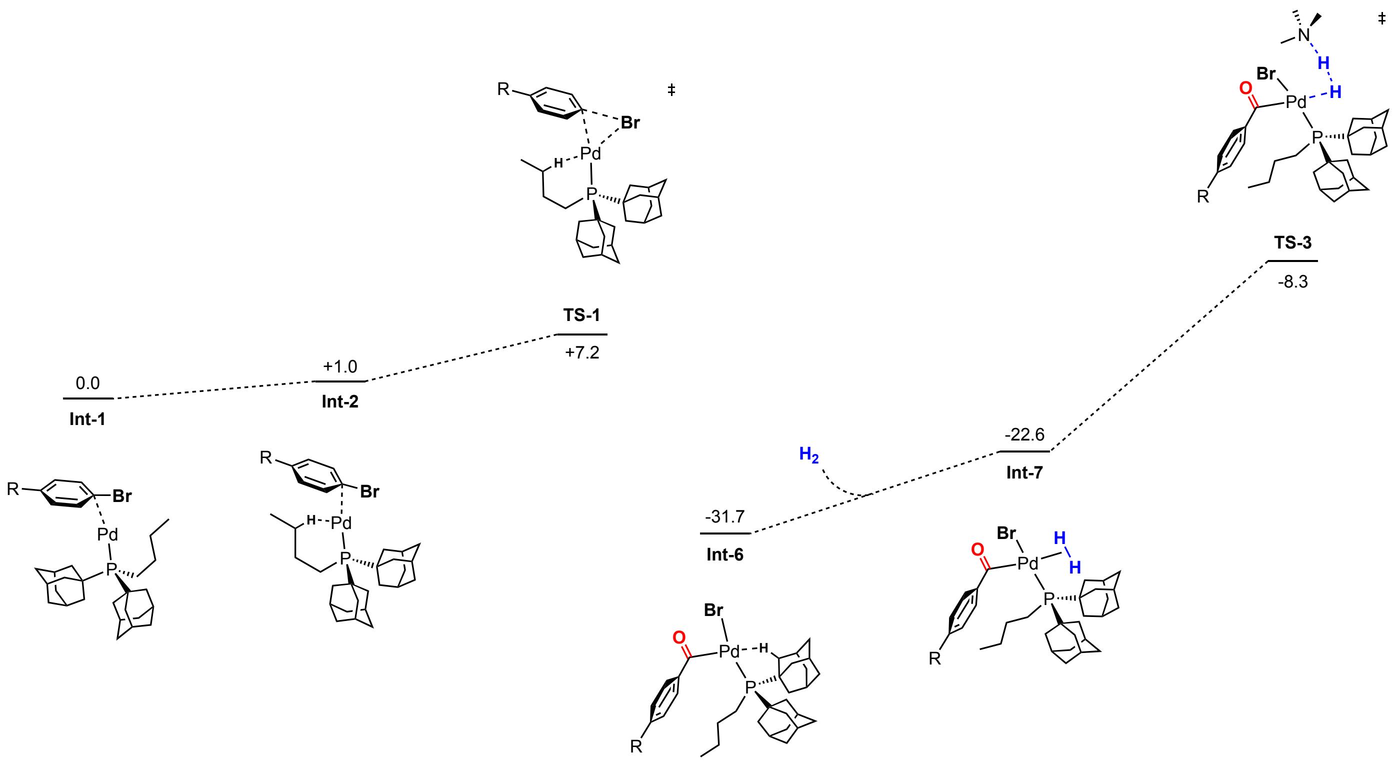

Functional	$\Delta G^{\ddagger}_{TS-1}$ (cor.)	$\Delta G^{\ddagger}_{TS-3}$ (cor.)
ωB97xD	7.2	23.4
M06L	6.4	23.8
B3PW91	5.5	23.2
M062X	7.8	28.4
PBE0	6.2	22.2

Figure S36. DFT calculated activation barriers for oxidative addition and dihydrogen activation steps using different functionals (R = OMe). Energy values reported in kcal mol^{-1}.

Kinetic Isotope Effect

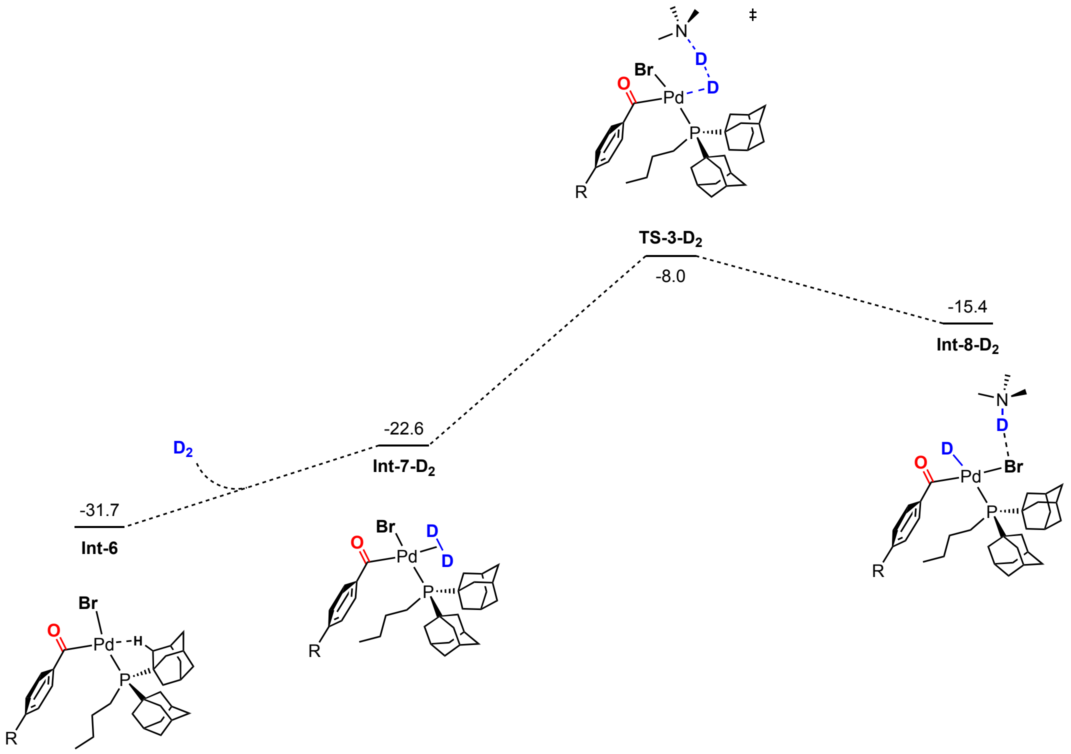

R	Int-6	Int-7-D$_2$	TS-3-D$_2$	$\Delta G^{\ddagger}_{TS\text{-}3\text{-}D2}$	Int-8-D$_2$
CN	-29.2	-20.2	-6.8	**22.4**	-14.9
CF_3	-27.7	-19.1	-5.6	**22.1**	-13.1
Cl	-28.7	-19.9	-5.8	**22.9**	-13.7
OMe	-31.7	-22.6	-8.0	**23.8**	-15.4
NMe_2	-32.3	-22.3	-5.5	**26.8**	-15.2

Figure S37. DFT calculated pathway for base-assisted dideuterium splitting. Energy values reported in kcal mol^{-1}.

Table S4. Calculated KIEs for dihydrogen coordination step, subsequent dihydrogen splitting step and combined.

R	Int-7-D$_2$ KIE	TS-3-D$_2$ KIE	Overall KIE
CN	0.96	1.14	1.10
CF_3	1.01	1.10	1.09
Cl	0.98	1.13	1.10
OMe	0.98	1.12	1.11
NMe_2	0.96	1.16	1.11

Key geometrical parameters of stationary points for the lowest energy formylation pathway

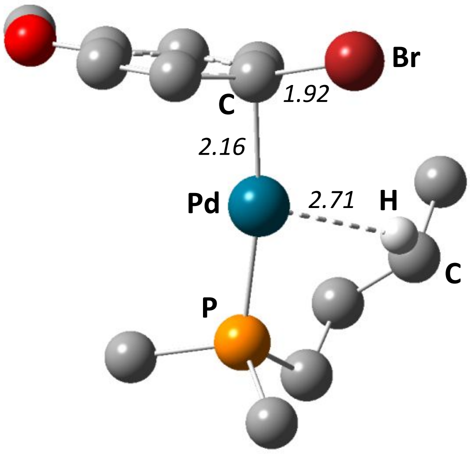

Figure S38. Selected bond lengths (in Å) for **Int-1** (R = OMe). Some hydrogens and adamantyl carbons have been omitted for clarity.

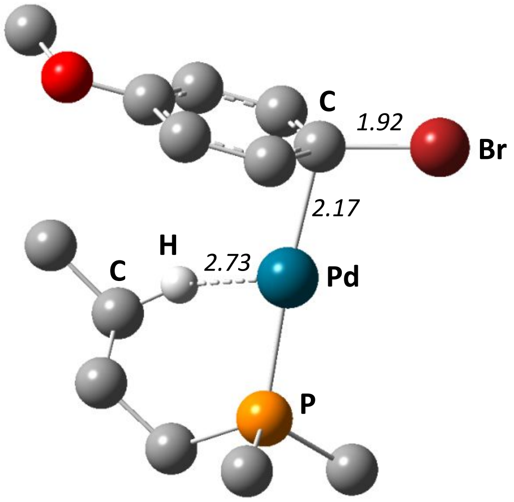

Figure S39. Selected bond lengths (in Å) for **Int-2** (R = OMe). Some hydrogens and adamantyl carbons have been omitted for clarity.

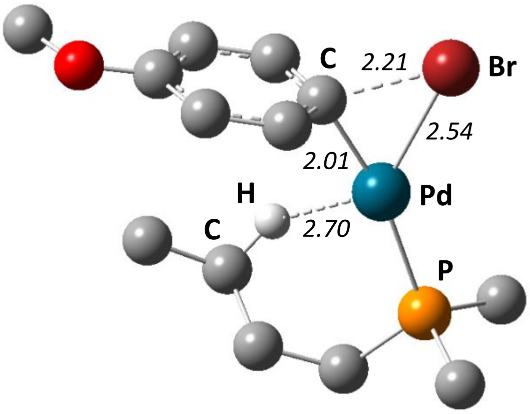

Figure S40. Selected bond lengths (in Å) for **TS-1** (R = OMe). Some hydrogens and adamantyl carbons have been omitted for clarity.

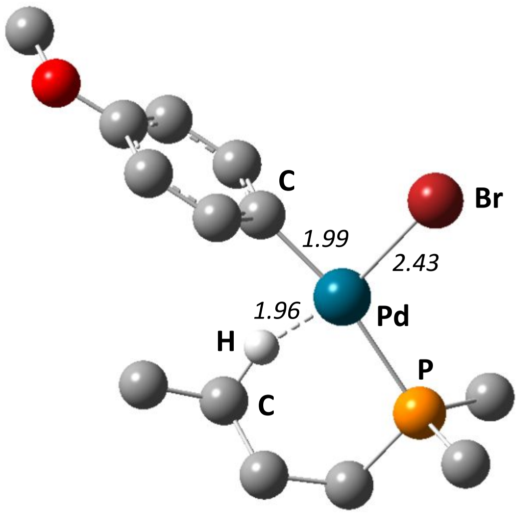

Figure S41. Selected bond lengths (in Å) for **Int-3** (R = OMe). Some hydrogens and adamantyl carbons have been omitted for clarity.

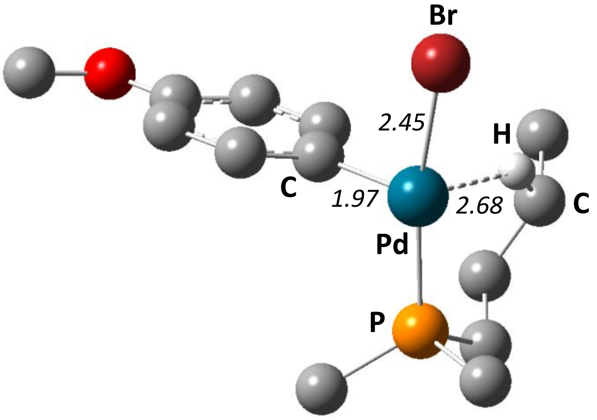

Figure S42. Selected bond lengths (in Å) for **Int-4** (R = OMe). Some hydrogens and adamantyl carbons have been omitted for clarity.

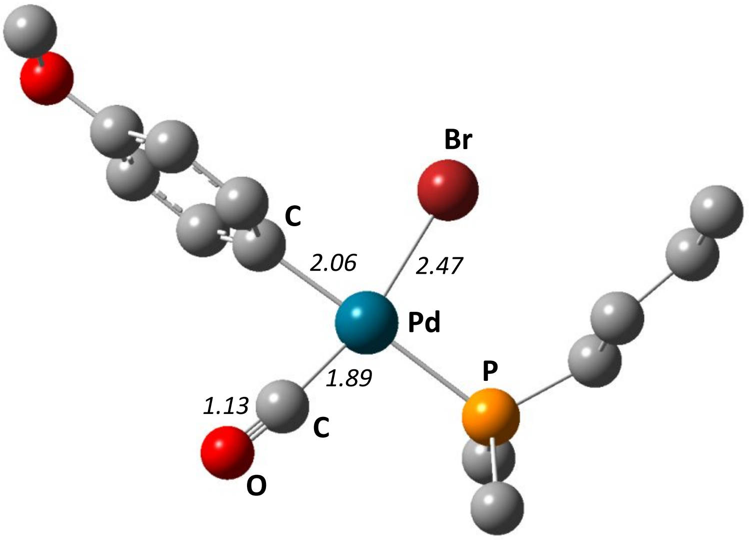

Figure S43. Selected bond lengths (in Å) for **Int-5** (R = OMe). Some hydrogens and adamantyl carbons have been omitted for clarity.

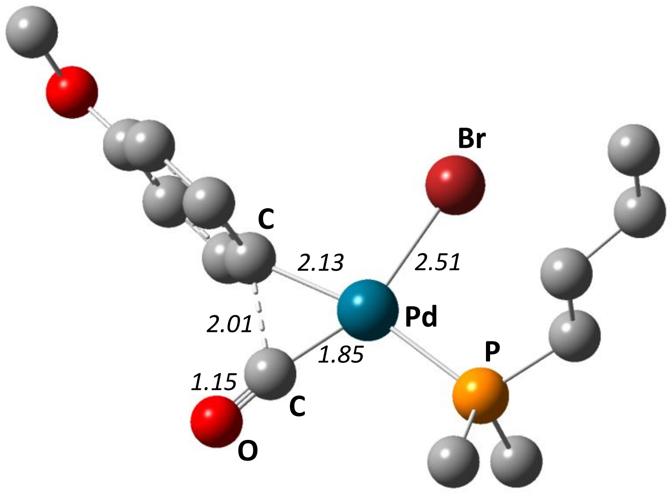

Figure S44. Selected bond lengths (in Å) for **TS-2** (R = OMe). Some hydrogens and adamantyl carbons have been omitted for clarity.

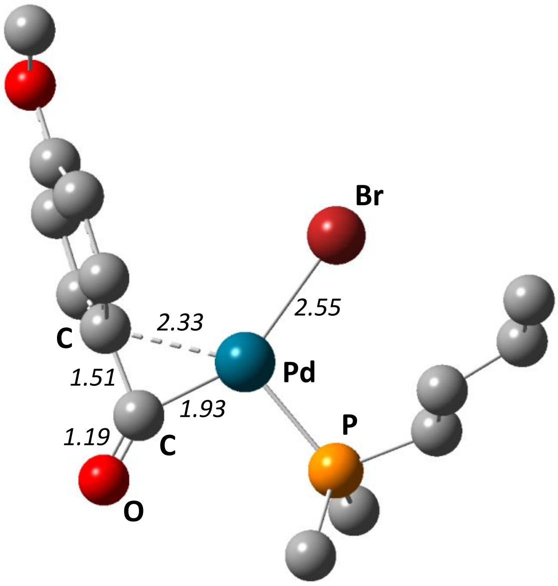

Figure S45. Selected bond lengths (in Å) for **Int-5'** (R = OMe). Some hydrogens and adamantyl carbons have been omitted for clarity.

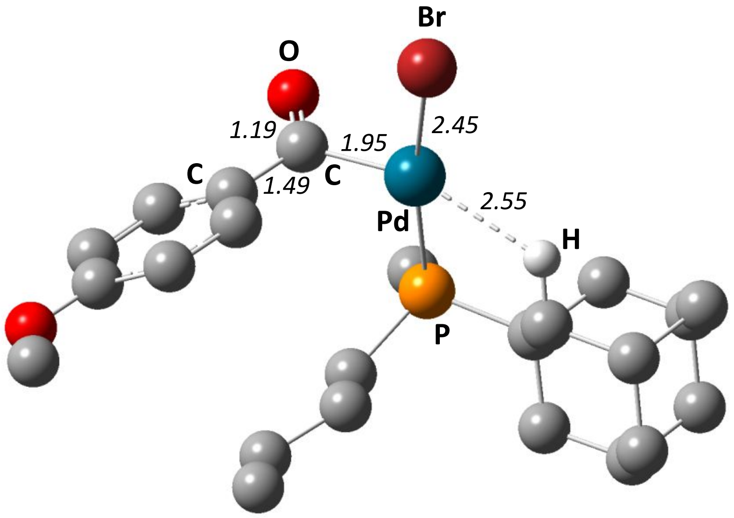

Figure S46. Selected bond lengths (in Å) for **Int-6** (R = OMe). Some hydrogens and adamantyl carbons have been omitted for clarity.

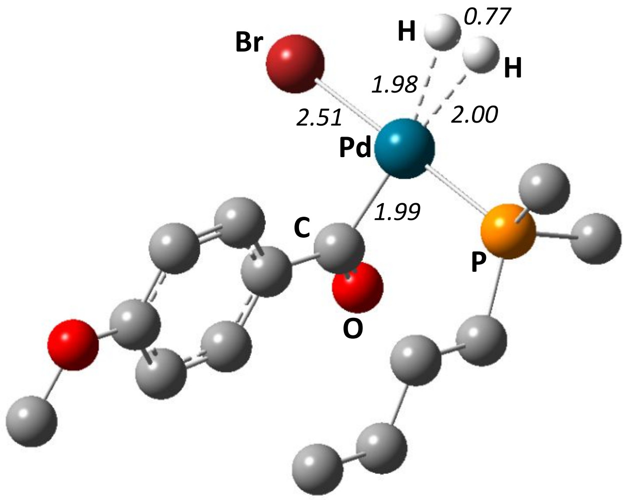

Figure S47. Selected bond lengths (in Å) for **Int-7** (R = OMe). Some hydrogens and adamantyl carbons have been omitted for clarity.

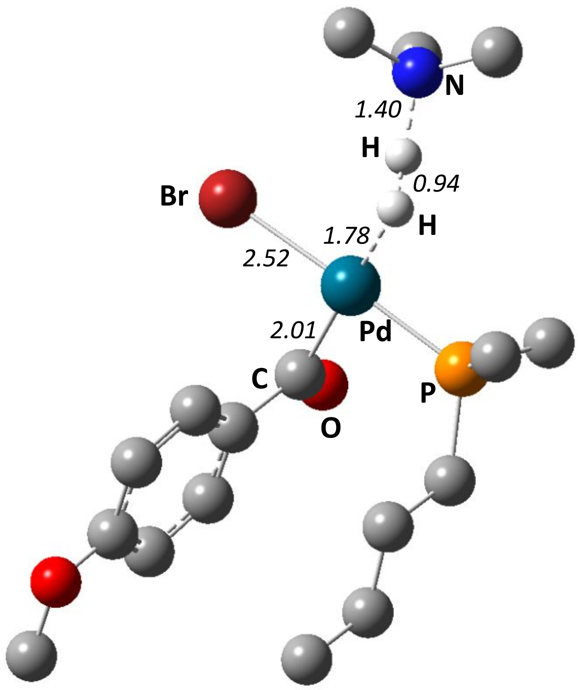

Figure S48. Selected bond lengths (in Å) for **TS-3** (R = OMe). Some hydrogens and adamantyl carbons have been omitted for clarity.

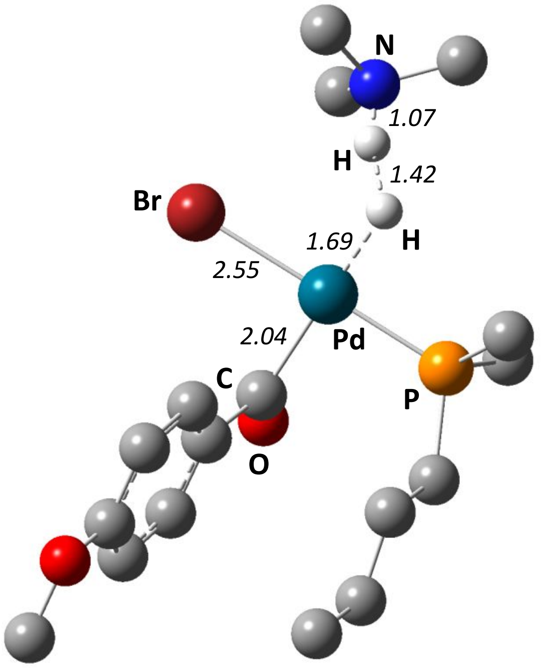

Figure S49. Selected bond lengths (in Å) for **Int-7'** (R = OMe). Some hydrogens and adamantyl carbons have been omitted for clarity.

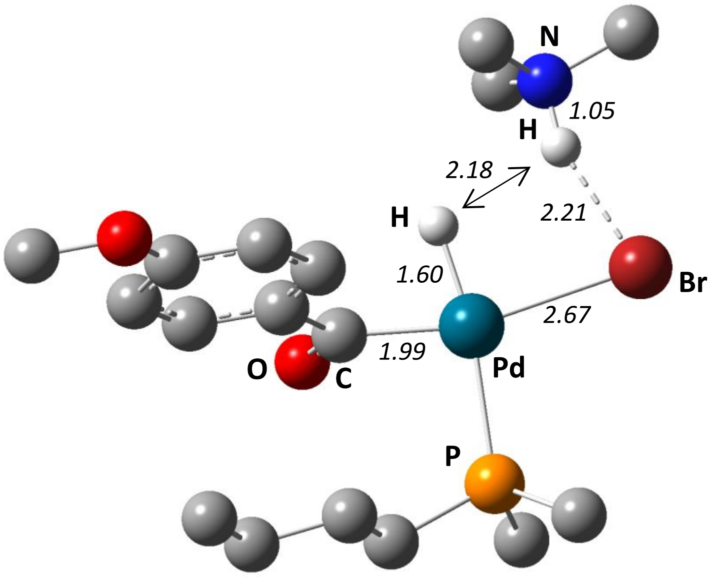

Figure S50. Selected bond lengths (in Å) for **Int-8** (R = OMe). Some hydrogens and adamantyl carbons have been omitted for clarity.

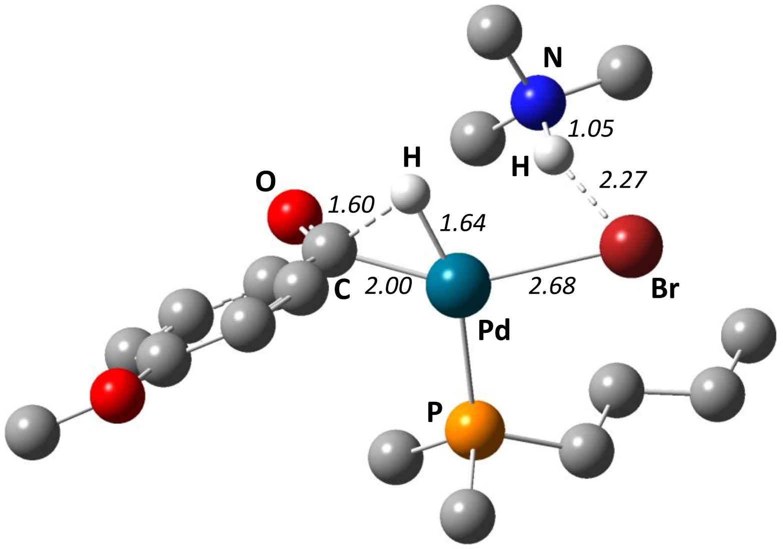

Figure S51. Selected bond lengths (in Å) for **TS-4** (R = OMe). Some hydrogens and adamantyl carbons have been omitted for clarity.

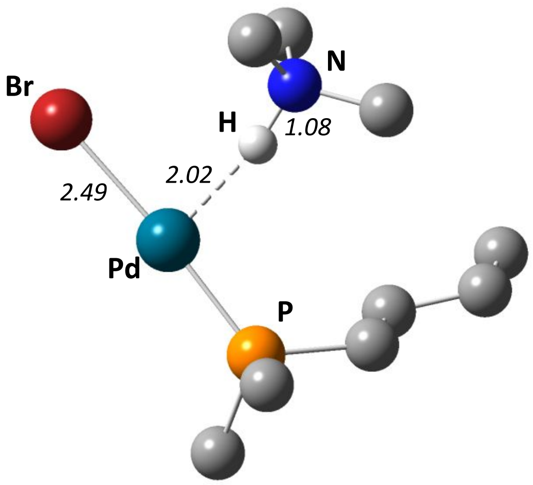

Figure S52. Selected bond lengths (in Å) for **Int-8'** (R = OMe). Some hydrogens and adamantyl carbons have been omitted for clarity.

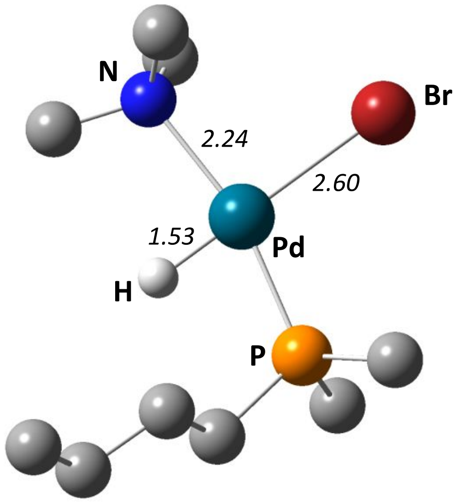

Figure S53. Selected bond lengths (in Å) for **Int-9** (R = OMe). Some hydrogens and adamantyl carbons have been omitted for clarity.

Key geometrical parameters of stationary points for the dideuterium activation step

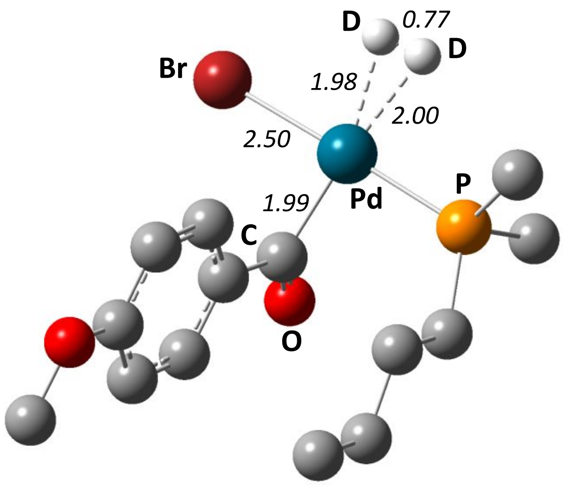

Figure S54. Selected bond lengths (in Å) for **Int-7-D$_2$** (R = OMe). Some hydrogens and adamantyl carbons have been omitted for clarity.

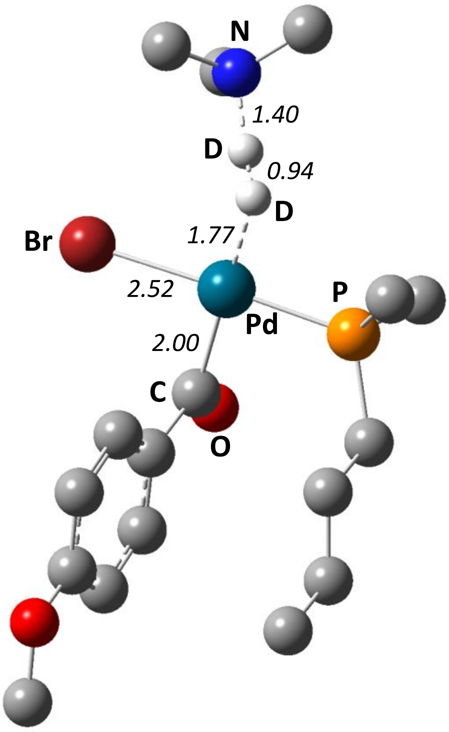

Figure S55. Selected bond lengths (in Å) for **TS-3-D$_2$** (R = OMe). Some hydrogens and adamantyl carbons have been omitted for clarity.

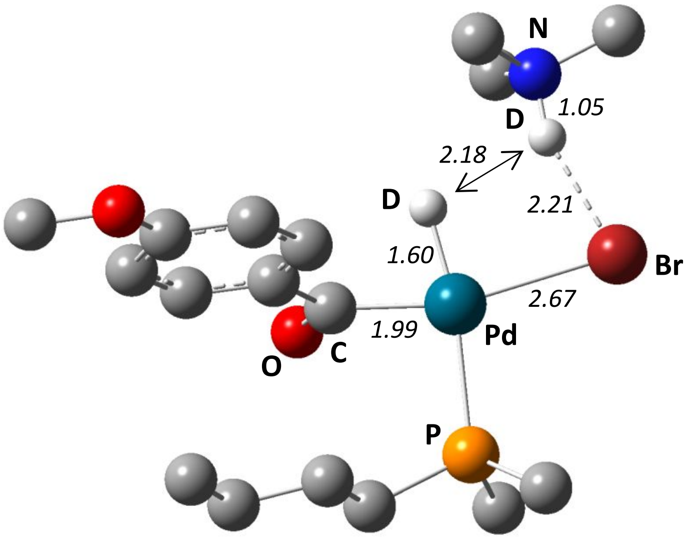

Figure S56. Selected bond lengths (in Å) for **Int-8-D$_2$** (R = OMe). Some hydrogens and adamantyl carbons have been omitted for clarity.

NBO analysis – NPA charges

Oxidative addition

R	Int-1 NPA charges			TS-1 NPA charges			Int-4 NPA charges		
	C	Pd	Br	C	Pd	Br	C	Pd	Br
CN	-0.19	0.061	0.074	-0.12	0.23	-0.14	-0.040	0.39	-0.63
CF_3	-0.21	0.073	0.068	-0.13	0.21	-0.15	-0.038	0.40	-0.61
Cl	-0.22	0.052	0.065	-0.14	0.20	-0.16	-0.056	0.39	-0.62
H	-0.22	0.039	0.056	-0.14	0.19	-0.17	-0.052	0.38	-0.63
Me	-0.22	0.020	0.053	-0.14	0.19	-0.18	-0.054	0.38	-0.63
OMe	-0.24	0.0079	0.054	-0.16	0.18	-0.18	-0.074	0.38	-0.63
NMe_2	-0.26	-0.016	0.048	-0.18	0.17	-0.19	-0.085	0.38	-0.63

Figure S57. Natural population analysis (NPA) charges calculated using NBO v6.0 on stationary points along the reaction pathway for oxidative addition.

Migratory insertion

R	Int-5 NPA charges			TS-2 NPA charges			Int-6 NPA charges		
	C_{Ar}	Pd	C_{CO}	C_{Ar}	Pd	C_{CO}	C_{Ar}	Pd	C_{CO}
CN	-0.20	0.42	0.59	-0.25	0.41	0.56	-0.16	0.32	0.57
CF_3	-0.20	0.42	0.59	-0.26	0.41	0.55	-0.17	0.32	0.58
Cl	-0.22	0.42	0.59	-0.27	0.41	0.56	-0.19	0.32	0.58
H	-0.22	0.42	0.59	-0.27	0.41	0.56	-0.19	0.31	0.58
Me	-0.22	0.42	0.59	-0.27	0.41	0.56	-0.20	0.31	0.59
OMe	-0.24	0.41	0.59	-0.29	0.41	0.56	-0.23	0.31	0.59
NMe_2	-0.25	0.41	0.59	-0.30	0.41	0.56	-0.26	0.30	0.59

Figure S58. Natural population analysis (NPA) charges calculated using NBO v6.0 on stationary points along the reaction pathway for migratory insertion.

Dihydrogen activation

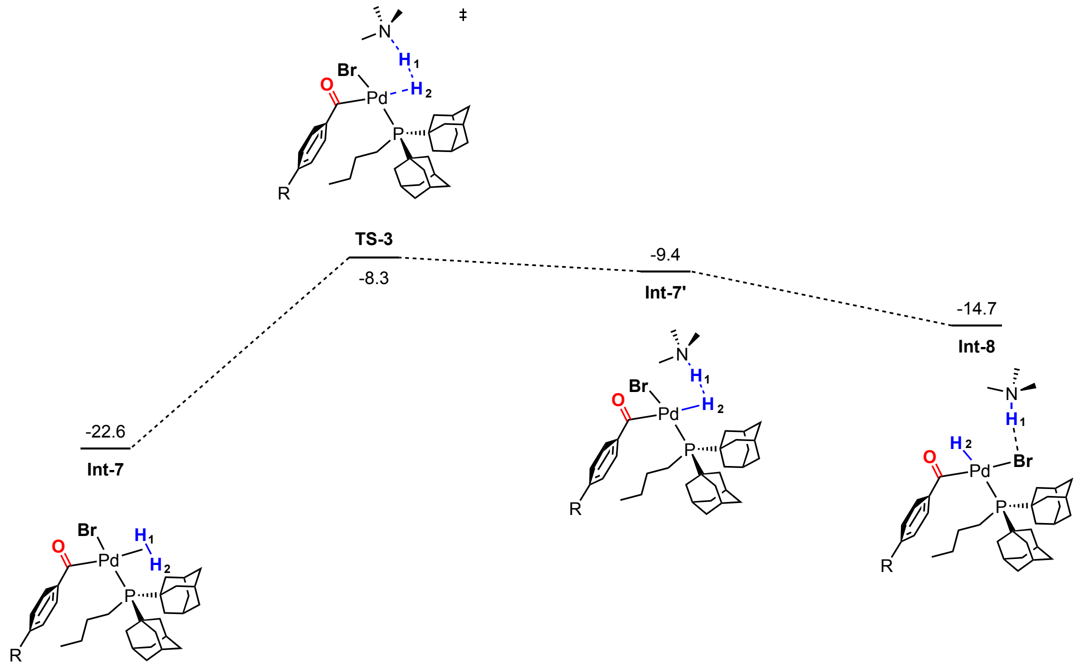

R	Int-7 NPA charges				TS-3 NPA charges					Int-8 NPA charges				
	H_1	H_2	Pd	Br	H_1	H_2	Pd	Br	N	H_1	H_2	Pd	Br	N
CN	0.073	-0.0087	0.32	-0.61	0.25	-0.24	0.33	-0.64	-0.55	0.51	-0.26	0.21	-0.69	-0.57
CF_3	0.073	-0.0094	0.32	-0.61	0.25	-0.25	0.33	-0.64	-0.55	0.51	-0.26	0.21	-0.69	-0.57
Cl	0.073	-0.011	0.31	-0.62	0.25	-0.25	0.33	-0.64	-0.55	0.51	-0.26	0.21	-0.70	-0.57
H	0.072	-0.012	0.31	-0.62	0.26	-0.26	0.33	-0.65	-0.55	0.52	-0.27	0.20	-0.70	-0.57
Me	0.071	-0.013	0.31	-0.62	0.26	-0.26	0.33	-0.65	-0.55	0.52	-0.27	0.20	-0.70	-0.57
OMe	0.071	-0.013	0.31	-0.62	0.26	-0.26	0.32	-0.65	-0.55	0.51	-0.32	0.23	-0.70	-0.56
NMe_2	0.068	-0.014	0.31	-0.63	0.26	-0.26	0.32	-0.65	-0.55	0.52	-0.27	0.20	-0.71	-0.58

Figure S59. Natural population analysis (NPA) charges calculated using NBO v6.0 on stationary points along the reaction pathway for dihydrogen activation.

Reductive elimination

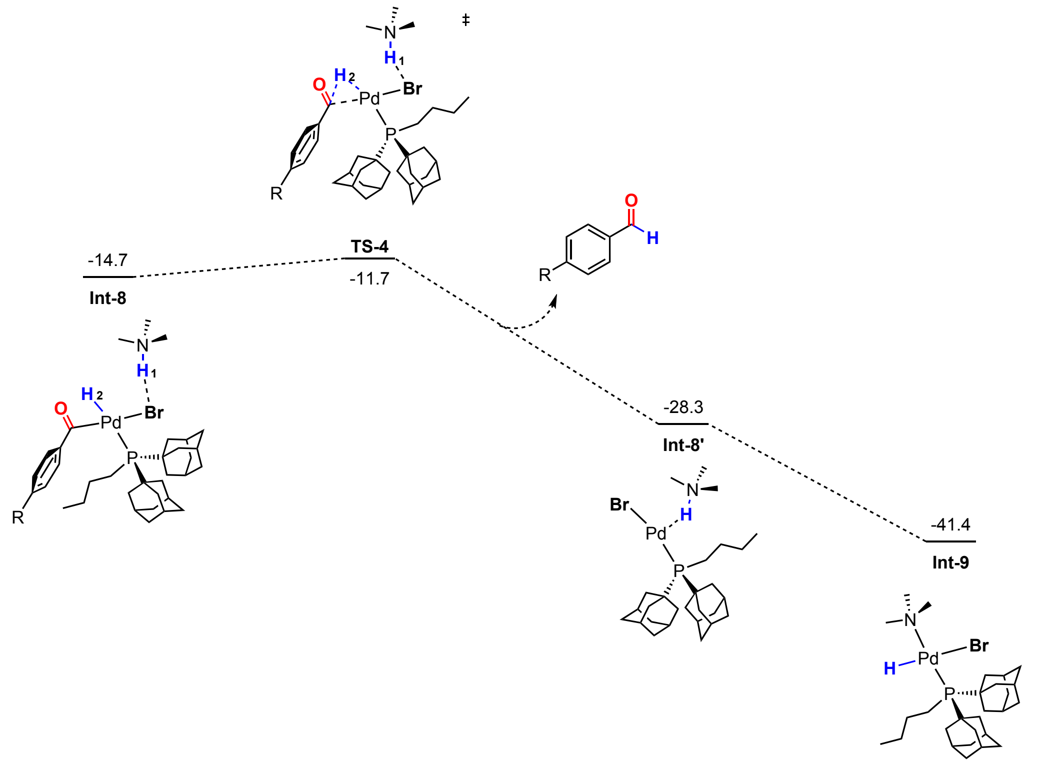

R	Int-8 NPA charges					TS-4 NPA charges			Int-9 NPA charges			
	H_1	H_2	Pd	Br	N	C	H	Pd	Pd	H	Br	N
CN	0.51	-0.26	0.21	-0.69	-0.57	0.35	-0.11	0.19				
CF_3	0.51	-0.26	0.21	-0.69	-0.57	0.35	-0.11	0.18				
Cl	0.51	-0.26	0.21	-0.70	-0.57	0.36	-0.11	0.18				
H	0.52	-0.27	0.20	-0.70	-0.57	0.36	-0.11	0.18	0.30	-0.12	-0.73	-0.55
Me	0.52	-0.27	0.20	-0.70	-0.57	0.36	-0.11	0.18				
OMe	0.51	-0.32	0.23	-0.70	-0.56	0.36	-0.11	0.18				
NMe_2	0.52	-0.27	0.20	-0.71	-0.58	0.37	-0.11	0.17				

Figure S60. Natural population analysis (NPA) charges calculated using NBO v6.0 on stationary points along the reaction pathway for reductive elimination.

4. Cartesian coordinates

1-bromo-4-chlorobenzene.log

SCF (RwB97XD) = -3265.24147997
E(SCF)+ZPE(0 K)= -3265.159610
H(298 K)= -3265.151842
G(298 K)= -3265.192596
Lowest Frequency = 94.1155cm-1

C	-2.170030	0.000076	0.000663
C	-1.482578	1.212322	0.000648
C	-0.089907	1.212079	0.000712
C	0.598361	-0.000048	0.000789
C	-0.090015	-1.212112	0.000806
C	-1.482686	-1.212231	0.000741
H	-2.029896	2.156133	0.000586
H	0.451968	2.158848	0.000699
H	0.451775	-2.158930	0.000867
H	-2.030089	-2.155993	0.000754
Br	2.491286	-0.000133	0.000869
Cl	-3.907247	0.000151	0.000584

4-bromoanisole.log

SCF (RwB97XD) = -2920.12068944
E(SCF)+ZPE(0 K)= -2919.996338
H(298 K)= -2919.987278
G(298 K)= -2920.030365
Lowest Frequency = 90.3066cm-1

C	-2.256626	0.232555	0.003103
C	-1.630977	-1.018369	-0.000459
C	-0.237032	-1.100100	-0.004354
C	0.529077	0.058979	-0.004671
C	-0.088110	1.312185	-0.001244
C	-1.472809	1.395643	0.002661
H	-2.209874	-1.941848	-0.000311
H	0.243206	-2.079947	-0.007070
H	0.511341	2.224077	-0.001519
H	-1.970128	2.367261	0.005422
O	-3.596628	0.419587	0.007116
C	-4.432684	-0.718832	0.007269
H	-4.274758	-1.338605	0.905590
H	-5.464323	-0.345664	0.011395
H	-4.280400	-1.334524	-0.894826
Br	2.421807	-0.058706	-0.009599

4-bromobenzonitrile.log

SCF (RwB97XD) = -2897.83028662
E(SCF)+ZPE(0 K)= -2897.740109
H(298 K)= -2897.731758
G(298 K)= -2897.773586
Lowest Frequency = 81.4546cm-1

C	-2.331418	0.000026	-0.000006
C	-1.632370	1.214827	-0.000006
C	-0.242422	1.215415	-0.000005
C	0.442531	0.000063	-0.000003
C	-0.242390	-1.215307	-0.000003
C	-1.632338	-1.214757	-0.000004
H	-2.177411	2.160047	-0.000007
H	0.302993	2.159770	-0.000005
H	0.303050	-2.159647	-0.000002
H	-2.177354	-2.159991	-0.000004
Br	2.331776	0.000089	-0.000002
C	-3.770148	0.000007	-0.000007
N	-4.920572	-0.000009	-0.000009

4-bromobenzotrifluoride.log

SCF (RwB97XD) = -3142.69158030
E(SCF)+ZPE(0 K)= -3142.595287
H(298 K)= -3142.585130
G(298 K)= -3142.632360
Lowest Frequency = 27.1329cm-1

C	-1.536621	0.006855	-0.021989
C	-0.846656	-0.617202	1.021016
C	0.543043	-0.621395	1.034388
C	1.236408	0.003239	-0.003463
C	0.558098	0.627304	-1.047558
C	-0.835265	0.627670	-1.053862
H	-1.394668	-1.103921	1.830791
H	1.081686	-1.108202	1.848231
H	1.107190	1.113288	-1.854933
H	-1.368186	1.116657	-1.870201
C	-3.044489	-0.005209	0.001727
F	-3.532605	-1.260776	0.014965
F	-3.529428	0.602878	1.101872
F	-3.582810	0.614482	-1.058566
Br	3.128116	0.001757	0.009806

4-bromo-nn-dimethylaniline.log

SCF (RwB97XD) = -2939.52375288
E(SCF)+ZPE(0 K)= -2939.358888
H(298 K)= -2939.347984
G(298 K)= -2939.395727
Lowest Frequency = 41.1581cm-1

C	-2.025971	0.001263	-0.009739
C	-1.290693	-1.204454	-0.071515
C	0.099224	-1.202231	-0.086257
C	0.800310	-0.000050	-0.038138
C	0.101451	1.202741	0.024410
C	-1.288439	1.206224	0.038671
H	-1.802434	-2.165114	-0.110750
H	0.634707	-2.152092	-0.136148
H	0.638695	2.152097	0.063808
H	-1.798396	2.167304	0.088737
Br	2.698372	-0.000866	-0.056494
N	-3.398386	0.002224	0.002450
C	-4.118955	1.250297	0.091633
H	-3.910556	1.910941	-0.768274
H	-5.196282	1.048690	0.102616
H	-3.874388	1.805064	1.014635
C	-4.122147	-1.246011	-0.049770
H	-3.892295	-1.818002	-0.965625
H	-3.901979	-1.891208	0.819262
H	-5.199198	-1.042608	-0.049765

4-bromotoluene.log

SCF (RwB97XD) = -2844.90810052
E(SCF)+ZPE(0 K)= -2844.789257
H(298 K)= -2844.780820
G(298 K)= -2844.822953
Lowest Frequency = 47.0956cm-1

C	-2.675724	0.005816	0.008421
C	-1.952990	1.203120	-0.010563
C	-0.558454	1.211643	-0.016955
C	0.129081	0.001611	-0.003464
C	-0.562959	-1.208586	0.016393
C	-1.954799	-1.195918	0.022319
H	-2.487427	2.156628	-0.020589
H	-0.015548	2.158055	-0.031411
H	-0.021581	-2.155945	0.028810
H	-2.492780	-2.147730	0.039005
Br	2.026039	-0.002517	-0.009577
C	-4.180740	-0.003591	-0.002661
H	-4.592486	1.003782	0.150386
H	-4.564059	-0.378972	-0.965155
H	-4.582573	-0.658901	0.785068

4-chlorobenzaldehyde.log

SCF (RwB97XD) = -804.917760266
E(SCF)+ZPE(0 K)= -804.816486
H(298 K)= -804.808077
G(298 K)= -804.849366
Lowest Frequency = 77.6147cm-1

C	-1.272184	0.000371	-0.000793
C	-0.490649	1.159137	-0.001041
C	0.892475	1.040621	-0.000869
C	1.494115	-0.224804	-0.000454
C	0.694478	-1.372043	-0.000213
C	-0.693152	-1.267814	-0.000385
H	-0.967580	2.140195	-0.001363
H	1.522986	1.932381	-0.001057
H	1.161616	-2.360702	0.000112
H	-1.322974	-2.158432	-0.000200
C	2.969682	-0.365268	-0.000257
H	3.338553	-1.419209	0.000152
O	3.747380	0.558554	-0.000507
Cl	-3.001110	0.143660	-0.000994

4-cyanobenzaldehyde.log

SCF (RwB97XD) = -437.504850072
E(SCF)+ZPE(0 K)= -437.395331
H(298 K)= -437.386308
G(298 K)= -437.428742
Lowest Frequency = 72.9861cm-1

C	-1.428273	0.010041	-0.000307
C	-0.634228	1.168506	-0.000042
C	0.747764	1.048841	0.000633
C	1.341779	-0.219488	0.001024
C	0.545675	-1.368622	0.000727
C	-0.840571	-1.261768	0.000064
H	-1.107268	2.151623	-0.000350
H	1.382804	1.937010	0.000874
H	1.015045	-2.355769	0.001032
H	-1.468592	-2.153820	-0.000152
C	2.822801	-0.361712	0.001843
H	3.192905	-1.414440	0.002058
O	3.594093	0.565286	0.002282
C	-2.863310	0.130906	-0.000996
N	-4.009419	0.227368	-0.001535

4-dimethylaminobenzaldehyde.log

SCF (RwB97XD) = -479.204349067
E(SCF)+ZPE(0 K)= -479.019609
H(298 K)= -479.008269
G(298 K)= -479.055525
Lowest Frequency = 75.2178cm-1

C	-1.090564	0.007861	0.000738
C	-0.454318	1.274976	0.001176
C	0.927332	1.369287	-0.000845
C	1.740287	0.227591	-0.003414
C	1.115146	-1.030289	-0.003649
C	-0.261334	-1.146271	-0.001541
H	-1.044606	2.190259	0.003031
H	1.392612	2.359811	-0.000540
H	1.736867	-1.928804	-0.005638
H	-0.704782	-2.141016	-0.001843
C	3.200369	0.362816	-0.006260
H	3.566740	1.419711	-0.005257
O	3.994173	-0.554670	-0.009643
N	-2.450002	-0.102129	0.002316
C	-3.077307	-1.405955	0.001400
H	-2.805058	-1.994581	0.893785
H	-4.166088	-1.283756	0.002858
H	-2.807161	-1.992360	-0.893099
C	-3.274744	1.086197	0.003685
H	-3.096514	1.707631	0.897651
H	-3.097820	1.708752	-0.889767
H	-4.331331	0.795764	0.004296

4-trifluoromethylbenzaldehyde.log

SCF (RwB97XD) = -682.366393074
E(SCF)+ZPE(0 K)= -682.250767
H(298 K)= -682.239963
G(298 K)= -682.287675
Lowest Frequency = 28.5550cm-1

C	-0.630881	-0.007843	0.052049
C	0.123903	-0.245502	-1.101553
C	1.510543	-0.233372	-1.029789
C	2.145175	0.018080	0.192517
C	1.383468	0.257450	1.339399
C	-0.006526	0.246411	1.273649
H	-0.374386	-0.441904	-2.052193
H	2.115993	-0.419601	-1.919107
H	1.883399	0.451753	2.292144
H	-0.600882	0.431221	2.169736
C	-2.138673	0.026534	-0.037712
F	-2.724368	-0.332531	1.115893
F	-2.608874	-0.792036	-0.992208
F	-2.584546	1.264088	-0.331704
C	3.628457	0.034282	0.289664
H	4.027966	0.235668	1.312403
O	4.375756	-0.150840	-0.639050

benzaldehyde.log

SCF (RwB97XD) = -345.304085146
E(SCF)+ZPE(0 K)= -345.193180
H(298 K)= -345.185918
G(298 K)= -345.223901
Lowest Frequency = 95.0158cm-1

C	-2.242712	-0.277964	0.003454
C	-1.779603	1.037527	0.002302
C	-0.408804	1.286328	-0.001376
C	0.498582	0.221096	-0.004214
C	0.027522	-1.098912	-0.002867
C	-1.340391	-1.345749	0.001018
H	-2.488427	1.868199	0.004191
H	-0.035428	2.314485	-0.002367
H	0.748193	-1.919602	-0.004861
H	-1.710595	-2.373166	0.002103
C	1.952729	0.507974	-0.009266
H	2.214120	1.594211	-0.008071
O	2.822480	-0.330583	-0.014766
H	-3.317154	-0.475940	0.006268

bromobenzene.log

SCF (RwB97XD) = -2805.62841673
E(SCF)+ZPE(0 K)= -2805.536933
H(298 K)= -2805.530349
G(298 K)= -2805.567767
Lowest Frequency = 173.3284cm-1

C	-3.042526	0.000172	-0.000087
C	-2.343108	1.206389	0.000201
C	-0.948747	1.214362	0.000406
C	-0.264314	-0.000103	0.000315
C	-0.948987	-1.214433	0.000034
C	-2.343347	-1.206183	-0.000170
H	-2.883669	2.155466	0.000270
H	-0.401094	2.158063	0.000633
H	-0.401521	-2.158242	-0.000036
H	-2.884097	-2.155153	-0.000397
Br	1.632557	-0.000293	0.000569
H	-4.134515	0.000280	-0.000248

cataxcium-a.log

SCF (RwB97XD) = -1278.59475483
E(SCF)+ZPE(0 K)= -1277.999438
H(298 K)= -1277.976581
G(298 K)= -1278.049922
Lowest Frequency = 27.8664cm-1

C	3.267194	-2.350788	0.193998
H	4.264350	-2.822284	0.184177
H	2.526334	-3.167920	0.218267
C	3.071958	-1.501187	-1.070345
H	3.185543	-2.136167	-1.963927
C	3.113128	-1.464276	1.438469
H	3.248020	-2.073663	2.346650
C	4.159300	-0.343312	1.406665
H	5.177297	-0.767915	1.414300
H	4.065642	0.285801	2.307513
C	4.116220	-0.376977	-1.099203
H	3.993333	0.228729	-2.012991
H	5.133666	-0.801767	-1.130010
C	3.949782	0.505050	0.146086
H	4.686349	1.324498	0.122438
C	2.536044	1.107770	0.153476
H	2.412111	1.769916	1.027801
H	2.415216	1.734050	-0.744237
C	1.658477	-0.893270	-1.063390
H	0.916965	-1.706487	-1.084545
H	1.506821	-0.301610	-1.981810
C	1.705975	-0.847474	1.455176
H	0.960193	-1.650876	1.542783
H	1.584414	-0.207952	2.345505
C	1.459198	-0.004373	0.180732
P	-0.218156	0.828176	0.325124
C	-1.456089	-0.412395	-0.332823
H	-1.197962	-0.576264	-1.393935
C	-2.891785	0.177729	-0.278655
C	-1.491965	-1.794318	0.361214
H	-2.907831	1.176336	-0.743380
C	-3.385703	0.294743	1.173296
C	-3.842448	-0.746084	-1.058039
H	-0.491664	-2.247320	0.349322
C	-1.981118	-1.670738	1.812273
C	-2.444434	-2.717890	-0.417997
H	-4.398374	0.731807	1.180859
H	-2.736637	0.976915	1.744235
C	-3.403833	-1.092005	1.831056
H	-3.518349	-0.822868	-2.109539
H	-4.857825	-0.315241	-1.064825
C	-3.864216	-2.136714	-0.407428
H	-1.303777	-1.024156	2.393725
H	-1.966583	-2.663660	2.292539
H	-2.087772	-2.837400	-1.454940
H	-2.443186	-3.721881	0.039177
H	-3.752429	-1.003183	2.872518
C	-4.348262	-2.015939	1.046217
H	-4.541856	-2.799029	-0.970171
H	-4.382133	-3.013309	1.516460
H	-5.375552	-1.614751	1.071155
C	-0.211055	1.984886	-1.127027
H	0.497706	1.628578	-1.893279
H	-1.204535	1.925862	-1.597793
C	0.076727	3.441641	-0.763831
H	-0.693847	3.795349	-0.056818
H	1.032863	3.516265	-0.218667
C	0.120801	4.367107	-1.978435
H	0.900518	4.011049	-2.674788
H	-0.833134	4.288877	-2.529205
C	0.385353	5.823980	-1.618073
H	-0.400296	6.221047	-0.955069
H	1.347488	5.937171	-1.092592
H	0.418950	6.463294	-2.513607

co.log

SCF (RwB97XD) = -113.270608626
E(SCF)+ZPE(0 K)= -113.265497
H(298 K)= -113.262192
G(298 K)= -113.284614
Lowest Frequency = 2243.6566cm-1

C	-0.642909	0.000000	0.000000
O	0.482211	0.000000	0.000000

d2.log

SCF (RwB97XD) = -1.17620153124
E(SCF)+ZPE(0 K)= -1.169069
H(298 K)= -1.165764
G(298 K)= -1.182193
Lowest Frequency = 3130.8202cm-1

H	0.371823	0.000000	0.000000
H	-0.371823	0.000000	0.000000

h2.log

SCF (RwB97XD) = -1.17130246782
E(SCF)+ZPE(0 K)= -1.161267
H(298 K)= -1.157962
G(298 K)= -1.172793
Lowest Frequency = 4405.0346cm-1

H	0.379079	0.000000	0.000000
H	-0.379079	0.000000	0.000000

int-1-cf3.log

SCF (RwB97XD) = -4549.27740193
E(SCF)+ZPE(0 K)= -4548.584618
H(298 K)= -4548.548737
G(298 K)= -4548.653442
Lowest Frequency = 14.9593cm-1

C	-1.818107	-0.977403	-2.447364
H	-1.562404	-1.906652	-2.976948
H	-2.908350	-1.010425	-2.301469
C	-1.419493	0.209412	-3.333112
H	-2.190822	0.342988	-4.111217
H	-1.411993	1.141616	-2.744983
C	-0.061066	0.039995	-4.008660
H	0.704545	-0.166309	-3.236350
C	-2.269583	-0.330078	0.381394
C	-3.549565	-1.165251	0.574803
H	-3.299285	-2.138846	1.022369
H	-4.032837	-1.368116	-0.395282
C	-4.534701	-0.432892	1.503507
H	-5.440299	-1.050398	1.617264
C	-3.876290	-0.222432	2.874298
H	-3.620814	-1.196217	3.325521
H	-4.581413	0.274261	3.561654
C	-2.610996	0.630675	2.706262
H	-2.125886	0.771626	3.685532
C	-2.984135	1.993263	2.111228
H	-3.671776	2.530921	2.785278
H	-2.083836	2.619793	2.002634
C	-3.641657	1.779200	0.743013
H	-3.898408	2.753474	0.297730
C	-2.665429	1.053578	-0.193519
H	-3.144712	0.937683	-1.177657
H	-1.758853	1.663881	-0.343188
C	-4.908912	0.928450	0.902420
H	-5.634898	1.443162	1.553802
H	-5.397019	0.790062	-0.077248
C	-1.627756	-0.085312	1.768183
H	-0.715340	0.518335	1.641765
H	-1.317278	-1.035159	2.225447
C	-0.745050	-2.880496	-0.541322
C	-0.368715	-3.180510	0.924868
H	0.464660	-2.528479	1.237166
H	-1.221550	-2.958113	1.583969

C	0.013632	-4.658881	1.097249
H	0.275819	-4.836092	2.152601
C	1.212602	-4.996479	0.202389
H	2.082570	-4.381211	0.486070
H	1.503517	-6.051486	0.339401
C	0.839656	-4.734079	-1.262473
H	1.700237	-4.961192	-1.911667
C	-0.355620	-5.610906	-1.658132
H	-0.094714	-6.677819	-1.557733
H	-0.616444	-5.441662	-2.716529
C	-1.552074	-5.271381	-0.760156
H	-2.416807	-5.893071	-1.042704
C	-1.929440	-3.790074	-0.939354
H	-2.813683	-3.565985	-0.327611
H	-2.215569	-3.617796	-1.988216
C	0.470075	-3.254010	-1.430180
H	0.247741	-3.046606	-2.490137
H	1.329937	-2.618528	-1.158568
C	-1.183740	-5.536355	0.706157
H	-2.044894	-5.314940	1.359151
H	-0.938344	-6.602043	0.848650
P	-0.991411	-1.040911	-0.793491
Pd	0.982119	0.175812	-0.700459
C	1.086863	3.578013	0.118919
C	1.479319	3.446178	-1.228895
C	2.273015	2.385689	-1.627450
C	2.247569	1.515338	0.670998
C	1.451770	2.618319	1.049954
H	1.167388	4.188709	-1.966173
H	2.614674	2.311373	-2.660760
H	2.663855	0.868664	1.445089
H	1.148561	2.712077	2.093611
C	0.214415	4.738128	0.501525
C	2.696028	1.415343	-0.674248
Br	4.185014	0.290994	-1.103634
F	0.021144	4.831544	1.827268
F	0.728656	5.917135	0.096027
F	-1.011643	4.652976	-0.063054
C	0.359256	1.260001	-4.815814
H	0.417336	2.155040	-4.175453
H	1.346081	1.116031	-5.282277
H	-0.360831	1.478796	-5.621192
H	-0.085224	-0.851753	-4.659298

int-1-cl.log

SCF (RwB97XD) = -4671.82453685
E(SCF)+ZPE(0 K)= -4671.146489
H(298 K)= -4671.112775
G(298 K)= -4671.213686
Lowest Frequency = 8.4558cm-1

C	-1.834386	-0.670387	-2.303739
H	-1.545315	-1.574031	-2.860032
H	-2.924292	-0.741231	-2.168098
C	-1.457915	0.558649	-3.140886
H	-2.227520	0.714210	-3.916489
H	-1.469623	1.464219	-2.512455
C	-0.090900	0.437137	-3.810862
H	0.655338	0.136096	-3.052562
H	-0.122309	-0.379658	-4.553352
C	0.370258	1.727821	-4.472650
H	1.341765	1.599383	-4.973785
H	0.488030	2.529831	-3.725708
H	-0.352939	2.078958	-5.227050
C	-2.319414	-0.112633	0.540639
C	-3.551270	-1.015776	0.740427
H	-3.245276	-1.980324	1.172274
H	-4.035142	-1.231711	-0.226805
C	-4.560687	-0.348897	1.691306
H	-5.429395	-1.016361	1.810855
C	-3.894376	-0.117474	3.055082
H	-3.577363	-1.080494	3.489876
H	-4.615912	0.330542	3.758796
C	-2.681014	0.806599	2.880193
H	-2.190144	0.965872	3.853274
C	-3.138241	2.151943	2.305136
H	-3.847445	2.641796	2.993369
H	-2.274380	2.828345	2.195914
C	-3.799863	1.915092	0.942247
H	-4.118655	2.877157	0.509602
C	-2.796383	1.253553	-0.013699
H	-3.277933	1.124947	-0.995299
H	-1.924456	1.913290	-0.162097
C	-5.017039	0.995258	1.109840
H	-5.760334	1.463738	1.776504
H	-5.511382	0.839642	0.135826
C	-1.672372	0.157463	1.921042
H	-0.798206	0.814964	1.787338
H	-1.297207	-0.775813	2.363997
C	-0.708340	-2.584457	-0.434722
C	-0.329712	-2.900481	1.027634
H	0.481985	-2.228568	1.354744
H	-1.190433	-2.714917	1.687911
C	0.096824	-4.369352	1.174081
H	0.357744	-4.559643	2.227515
C	1.311307	-4.651287	0.280883
H	2.159569	-4.014567	0.582848
H	1.634843	-5.699077	0.399336
C	0.939421	-4.371899	-1.181247
H	1.811011	-4.559054	-1.828605
C	-0.224011	-5.279534	-1.601486
H	0.070520	-6.339247	-1.518485
H	-0.483081	-5.099306	-2.658468
C	-1.436661	-4.995104	-0.706190
H	-2.279181	-5.638607	-1.006624
C	-1.859838	-3.523345	-0.860087
H	-2.756264	-3.340203	-0.252678
H	-2.141890	-3.339108	-1.908173
C	0.522745	-2.901599	-1.323265
H	0.299053	-2.680578	-2.380146
H	1.358987	-2.242579	-1.035652
C	-1.069860	-5.275987	0.757540
H	-1.941531	-5.093495	1.408499
H	-0.792154	-6.336057	0.881726
P	-1.013123	-0.747802	-0.648734
Pd	0.905046	0.522294	-0.439042
C	1.545936	2.478143	2.405057
C	1.258782	3.287959	1.300579
C	1.752190	2.946903	0.043647
C	2.768741	0.935542	0.999849
C	2.270525	1.301982	2.262093
H	0.658695	4.190937	1.421634
H	1.587033	3.615260	-0.802855
H	3.443252	0.081225	0.921850
H	2.469555	0.665585	3.125375
C	2.563375	1.791006	-0.115382
Br	3.689313	1.641522	-1.658282
Cl	0.952418	2.936125	3.975461

int-1-cn.log

SCF (RwB97XD) = -4304.41319927
E(SCF)+ZPE(0 K)= -4303.726145
H(298 K)= -4303.691891
G(298 K)= -4303.793575
Lowest Frequency = 9.7394cm-1

C	0.334946	-0.231325	-2.848886
H	0.622393	-1.127490	-3.422286
H	-0.732445	-0.061122	-3.048143
C	1.095862	0.998563	-3.342431
H	0.903921	1.844456	-2.658639
H	2.182279	0.818118	-3.311596
C	0.708071	1.397210	-4.765912
H	0.874945	0.539497	-5.440851
H	-0.375532	1.604933	-4.800688
C	1.477162	2.608973	-5.277428
H	1.300535	3.490919	-4.640905
H	1.178353	2.873462	-6.303257

H 2.562824 2.419818 -5.285705
C -0.728513 -2.049378 -0.838573
C -0.230676 -3.367490 -1.465838
H 0.722385 -3.675466 -1.011262
H -0.043125 -3.230336 -2.543808
C -1.266422 -4.487154 -1.254003
H -0.882882 -5.415278 -1.707740
C -1.486140 -4.699175 0.251099
H -0.538135 -4.989632 0.735032
H -2.197318 -5.525274 0.417771
C -2.023276 -3.405073 0.878264
H -2.175496 -3.548238 1.959598
C -3.351590 -3.021231 0.219128
H -4.102855 -3.812262 0.379203
H -3.749700 -2.104144 0.683462
C -3.119427 -2.804160 -1.279788
H -4.063228 -2.507169 -1.764775
C -2.084846 -1.689916 -1.499304
H -1.959248 -1.551752 -2.583170
H -2.454028 -0.734842 -1.088630
C -2.596822 -4.100283 -1.914078
H -3.334086 -4.910428 -1.786277
H -2.456218 -3.962163 -2.999514
C -1.006963 -2.274173 0.669024
H -1.388230 -1.337353 1.107460
H -0.081618 -2.517565 1.206231
C 2.199933 -1.025457 -0.703651
C 2.333774 -1.839735 0.602707
H 1.847034 -1.297133 1.430740
H 1.825013 -2.808949 0.504818
C 3.810525 -2.099384 0.938838
H 3.860220 -2.690084 1.867539
C 4.545324 -0.767948 1.130270
H 4.099269 -0.205735 1.967606
H 5.601960 -0.949304 1.389245
C 4.444521 0.048120 -0.163906
H 4.953622 1.016560 -0.034897
C 5.093851 -0.729736 -1.316869
H 6.161201 -0.905764 -1.102520
H 5.046528 -0.138694 -2.247067
C 4.364650 -2.067097 -1.503694
H 4.825573 -2.628509 -2.332146
C 2.888562 -1.802745 -1.846371
H 2.375127 -2.758328 -2.028188
H 2.838563 -1.230293 -2.785205
C 2.965400 0.307443 -0.485025
H 2.889542 0.949784 -1.373458
H 2.493511 0.864272 0.342620
C 4.456872 -2.886586 -0.209346
H 3.946451 -3.856223 -0.336952
H 5.511326 -3.107607 0.026238
P 0.409235 -0.564552 -1.040120
Pd -0.309085 1.177188 0.302386
C -2.691362 0.577258 2.755068
C -3.070296 1.229455 1.579055
C -2.254672 2.229736 1.023583
C -1.045872 2.591389 1.682317
C -0.643261 1.876394 2.846654
C -1.459925 0.887808 3.372087
H -4.013832 0.978669 1.091459
H -2.632687 2.828677 0.193418
H 0.280582 2.150639 3.357868
H -1.152118 0.352637 4.271560
Br -0.203633 4.259293 1.280418
C -3.523998 -0.455164 3.301035
N -4.184037 -1.296647 3.729352

int-1-h.log

SCF (RwB97XD) = -4212.20927246
E(SCF)+ZPE(0 K)= -4211.521671
H(298 K)= -4211.489114
G(298 K)= -4211.587722
Lowest Frequency = 9.6490cm-1

C -1.930736 -0.098710 -2.399480
H -1.636778 -0.885907 -3.109114
H -3.025832 -0.169135 -2.313143
C -1.505678 1.257074 -2.977877
H -2.244742 1.571197 -3.735284
H -1.525487 2.029505 -2.191856
C -0.117833 1.232443 -3.614106
H 0.600446 0.806482 -2.889725
H -0.128278 0.546140 -4.479200
C 0.371385 2.607657 -4.045062
H 1.363354 2.551384 -4.518659
H 0.457449 3.282314 -3.177619
H -0.318101 3.077419 -4.765811
C -2.504126 -0.076659 0.481862
C -3.759546 -0.969467 0.475169
H -3.488339 -2.005627 0.726789
H -4.216592 -0.989141 -0.528396
C -4.785146 -0.466149 1.506407
H -5.669840 -1.122148 1.476632
C -4.157836 -0.508997 2.907171
H -3.872124 -1.543543 3.162698
H -4.893012 -0.183042 3.661997
C -2.922167 0.401064 2.940411
H -2.459800 0.364537 3.940010
C -3.332789 1.841543 2.613599
H -4.051691 2.215567 3.361997
H -2.450674 2.502220 2.651505
C -3.956747 1.878193 1.213511
H -4.242516 2.911521 0.958954
C -2.937066 1.380090 0.178785
H -3.390527 1.447082 -0.822151
H -2.047543 2.032745 0.177511
C -5.196623 0.974024 1.174703
H -5.950858 1.329282 1.896816
H -5.664047 1.014509 0.176305
C -1.896788 -0.084452 1.905927
H -1.004530 0.562056 1.921931
H -1.556775 -1.094003 2.176709
C -0.917709 -2.363170 -0.895633
C -0.600657 -2.963148 0.490665
H 0.215240 -2.391584 0.965030
H -1.479579 -2.879745 1.147854
C -0.216621 -4.445866 0.367243
H 0.000522 -4.841468 1.372455
C 1.023183 -4.590426 -0.524500
H 1.874742 -4.048900 -0.079880
H 1.317369 -5.650919 -0.598256
C 0.712721 -4.027176 -1.917242
H 1.602996 -4.112940 -2.560454
C -0.455949 -4.801518 -2.540299
H -0.191936 -5.866487 -2.653054
H -0.670299 -4.414480 -3.550797
C -1.693596 -4.655037 -1.645959
H -2.539930 -5.202778 -2.091184
C -2.074960 -3.168559 -1.527917
H -2.988156 -3.077366 -0.924734
H -2.315515 -2.779651 -2.529273
C 0.337098 -2.544480 -1.788961
H 0.157997 -2.120450 -2.790978
H 1.177692 -1.977498 -1.355219
C -1.388578 -5.220452 -0.251890
H -2.279807 -5.138030 0.393024
H -1.141447 -6.293078 -0.322438
P -1.167280 -0.509298 -0.764997
Pd 0.766266 0.627265 -0.247531
C 1.283787 2.175648 2.885761
C 1.118605 3.113738 1.860349
C 1.689345 2.906920 0.603763
C 2.559942 0.751620 1.382361
C 1.978134 0.995187 2.638016
H 0.548806 4.028379 2.038726
H 1.620791 3.676425 -0.166855
H 3.199931 -0.120600 1.238388
H 2.092234 0.246014 3.424517
C 2.463580 1.740116 0.367834

Br	3.683554	1.708546	-1.112441
H	0.852693	2.361298	3.871411

int-1-me.log

SCF (RwB97XD) = -4251.48960763
E(SCF)+ZPE(0 K)= -4250.774706
H(298 K)= -4250.740380
G(298 K)= -4250.842355
Lowest Frequency = 10.7861cm-1

C	-1.793494	-0.644538	-2.228454
H	-1.455933	-1.527023	-2.791325
H	-2.881070	-0.766658	-2.110837
C	-1.467656	0.607733	-3.052104
H	-2.228343	0.718215	-3.844609
H	-1.544818	1.509586	-2.423187
C	-0.083095	0.573234	-3.694338
H	0.671760	0.380522	-2.908683
H	-0.025423	-0.279759	-4.393277
C	0.270357	1.863738	-4.419835
H	1.277885	1.815465	-4.861040
H	0.247958	2.721354	-3.728008
H	-0.440738	2.078365	-5.234572
C	-2.350488	-0.155893	0.612833
C	-3.566667	-1.090935	0.750859
H	-3.252025	-2.059664	1.167103
H	-4.017164	-1.292359	-0.235315
C	-4.619299	-0.471613	1.687941
H	-5.476933	-1.159641	1.761009
C	-4.001088	-0.265573	3.077889
H	-3.677601	-1.233984	3.495558
H	-4.753806	0.147985	3.770026
C	-2.801561	0.686163	2.965930
H	-2.344391	0.825149	3.958906
C	-3.265846	2.039529	2.415122
H	-4.001990	2.499389	3.095881
H	-2.409920	2.731913	2.346029
C	-3.884756	1.829078	1.028043
H	-4.210738	2.797154	0.614341
C	-2.842638	1.214528	0.082963
H	-3.297076	1.099806	-0.913091
H	-1.981146	1.894192	-0.028984
C	-5.086775	0.880247	1.133292
H	-5.859098	1.312383	1.791566
H	-5.549632	0.745037	0.141026
C	-1.752281	0.083607	2.019968
H	-0.885784	0.757699	1.931750
H	-1.377241	-0.857433	2.446159
C	-0.632995	-2.548894	-0.379911
C	-0.265999	-2.885181	1.080637
H	0.516071	-2.192883	1.435668
H	-1.143788	-2.745539	1.729808
C	0.210253	-4.341217	1.201474
H	0.462401	-4.546065	2.254306
C	1.447093	-4.560134	0.321188
H	2.267426	-3.900490	0.649626
H	1.805842	-5.598345	0.422107
C	1.086895	-4.261580	-1.140166
H	1.974163	-4.403250	-1.777694
C	-0.037867	-5.199253	-1.598289
H	0.291320	-6.250139	-1.534876
H	-0.287877	-5.003710	-2.654756
C	-1.272351	-4.977352	-0.715304
H	-2.087867	-5.642306	-1.042831
C	-1.744784	-3.518011	-0.841613
H	-2.654273	-3.381261	-0.241063
H	-2.021481	-3.320132	-1.888536
C	0.621582	-2.803688	-1.255694
H	0.405481	-2.566502	-2.310674
H	1.430215	-2.123231	-0.940450
C	-0.916960	-5.278926	0.747147
H	-1.804167	-5.142445	1.388337
H	-0.603237	-6.331073	0.852020
P	-0.994220	-0.717777	-0.559774
Pd	0.869651	0.613585	-0.340073
C	0.861553	4.004149	0.766577
C	1.272409	3.970923	-0.583520
C	2.097173	2.969175	-1.070521
C	2.549313	1.940871	-0.201231
C	2.103725	1.926590	1.143818
C	1.275248	2.974806	1.603305
H	0.940962	4.758184	-1.266257
H	2.439064	2.988249	-2.106667
H	2.533917	1.227384	1.863108
H	0.972219	2.972363	2.653625
Br	4.062140	0.883666	-0.726166
C	-0.027250	5.113794	1.259940
H	-0.211651	5.033823	2.340624
H	0.417885	6.102335	1.065657
H	-1.004439	5.094009	0.750451

int-1-nme2.log

SCF (RwB97XD) = -4346.10581948
E(SCF)+ZPE(0 K)= -4345.344445
H(298 K)= -4345.307673
G(298 K)= -4345.414599
Lowest Frequency = 7.1720cm-1

C	-1.846176	0.084378	-1.719811
H	-1.536460	-0.463112	-2.622067
H	-2.929882	-0.085318	-1.627371
C	-1.535382	1.572502	-1.926911
H	-2.334726	2.022131	-2.541753
H	-1.553184	2.103603	-0.961174
C	-0.188357	1.823204	-2.600895
H	0.598967	1.291386	-2.035084
H	-0.199228	1.377831	-3.611555
C	0.172393	3.299568	-2.687407
H	1.138403	3.449018	-3.193670
H	0.253136	3.742566	-1.681386
H	-0.588703	3.872995	-3.242065
C	-2.299138	-0.697915	1.072050
C	-3.513689	-1.617581	0.842683
H	-3.187679	-2.667464	0.795200
H	-4.001541	-1.385349	-0.118770
C	-4.529105	-1.470694	1.990135
H	-5.385873	-2.135388	1.793026
C	-3.858997	-1.868891	3.312675
H	-3.522395	-2.918512	3.265218
H	-4.584059	-1.801196	4.141186
C	-2.663129	-0.943254	3.574681
H	-2.168782	-1.233706	4.516286
C	-3.143064	0.509705	3.669902
H	-3.853743	0.626523	4.505980
H	-2.286271	1.173504	3.872406
C	-3.812277	0.900976	2.346926
H	-4.150028	1.949089	2.396018
C	-2.806863	0.761125	1.194661
H	-3.298170	1.076820	0.261459
H	-1.948394	1.435221	1.353909
C	-5.012679	-0.017436	2.079894
H	-5.759580	0.084606	2.885208
H	-5.511520	0.275108	1.140334
C	-1.649882	-1.069315	2.427494
H	-0.788676	-0.406622	2.607377
H	-1.257667	-2.095872	2.400203
C	-0.655339	-2.430487	-0.915691
C	-0.250458	-3.361573	0.246381
H	0.555191	-2.890417	0.834717
H	-1.104358	-3.509569	0.925100
C	0.202254	-4.730942	-0.283961
H	0.481069	-5.369147	0.570039
C	1.409029	-4.555153	-1.214746
H	2.249871	-4.102807	-0.663209
H	1.753221	-5.536973	-1.581119
C	1.011833	-3.657938	-2.394188
H	1.877103	-3.514706	-3.061123
C	-0.141414	-4.305734	-3.171698

H	0.171050	-5.283914	-3.574586
H	-0.417705	-3.675627	-4.033971
C	-1.345893	-4.479000	-2.237896
H	-2.181726	-4.936717	-2.791398
C	-1.796217	-3.104571	-1.710964
H	-2.686373	-3.235263	-1.081197
H	-2.098637	-2.475617	-2.562303
C	0.567742	-2.289786	-1.859039
H	0.323522	-1.623884	-2.703474
H	1.395403	-1.809193	-1.311721
C	-0.953847	-5.379732	-1.058448
H	-1.820369	-5.529177	-0.391971
H	-0.654874	-6.376405	-1.424688
P	-0.985780	-0.696690	-0.278109
Pd	0.869518	0.409300	0.463935
C	0.806397	2.029270	3.612547
C	0.901948	2.996375	2.579723
C	1.699862	2.789484	1.463495
C	2.368205	0.626649	2.345827
C	1.570545	0.855991	3.477073
H	0.335004	3.924404	2.634899
H	1.780059	3.572891	0.708227
H	3.018881	-0.249408	2.327345
H	1.549687	0.083419	4.243993
C	2.464467	1.605606	1.328858
Br	3.904501	1.553280	0.063778
N	-0.032561	2.219983	4.688063
C	-0.039101	1.260348	5.765423
H	-0.803103	1.542122	6.499786
H	-0.291836	0.250723	5.400650
H	0.933296	1.199544	6.289106
C	-0.698944	3.488237	4.865767
H	-1.349972	3.727614	4.008557
H	-1.338411	3.440351	5.755098
H	0.009552	4.326361	5.001080

int-1-ome.log

SCF (RwB97XD) = -4326.70196700
E(SCF)+ZPE(0 K)= -4325.981004
H(298 K)= -4325.946048
G(298 K)= -4326.048945
Lowest Frequency = 8.3249cm-1

C	-1.455103	-0.274714	-2.420362
H	-1.134459	-1.108058	-3.062634
H	-2.555027	-0.317022	-2.405577
C	-0.961800	1.035971	-3.046607
H	-1.654126	1.325510	-3.856495
H	-0.998109	1.850776	-2.305554
C	0.454274	0.949283	-3.609976
H	1.131208	0.583107	-2.816834
H	0.480983	0.193847	-4.415082
C	0.966604	2.283391	-4.134893
H	1.993502	2.198939	-4.522403
H	0.972317	3.044788	-3.337595
H	0.333095	2.669137	-4.950582
C	-2.208485	-0.035397	0.409872
C	-3.514101	-0.852338	0.367834
H	-3.325076	-1.885386	0.695352
H	-3.905681	-0.904796	-0.661966
C	-4.571820	-0.229986	1.296596
H	-5.491573	-0.834341	1.241772
C	-4.041061	-0.227428	2.737176
H	-3.836950	-1.260328	3.066574
H	-4.802412	0.183502	3.421343
C	-2.756591	0.610315	2.805145
H	-2.363193	0.606479	3.834285
C	-3.054320	2.050390	2.371768
H	-3.796216	2.509837	3.046675
H	-2.136173	2.656986	2.438185
C	-3.583097	2.039844	0.932480
H	-3.787737	3.071623	0.602467
C	-2.533622	1.422183	-0.003155
H	-2.921112	1.452010	-1.033227
H	-1.607265	2.020396	0.015192
C	-4.871860	1.209445	0.859654
H	-5.647266	1.649102	1.509302
H	-5.271048	1.218087	-0.168733
C	-1.697248	0.008037	1.870798
H	-0.771716	0.605678	1.912485
H	-1.434826	-1.000658	2.219505
C	-0.688713	-2.492792	-0.723101
C	-0.509517	-3.023156	0.715410
H	0.312747	-2.479890	1.211985
H	-1.421001	-2.837941	1.303633
C	-0.224956	-4.533055	0.708110
H	-0.106359	-4.875858	1.748502
C	1.059415	-4.817103	-0.080448
H	1.915185	-4.307970	0.393125
H	1.279907	-5.897908	-0.071121
C	0.886907	-4.322633	-1.522764
H	1.811456	-4.509284	-2.092343
C	-0.287344	-5.057087	-2.182769
H	-0.089668	-6.141800	-2.211907
H	-0.405306	-4.723084	-3.227489
C	-1.570483	-4.772488	-1.391864
H	-2.420790	-5.290455	-1.864269
C	-1.852236	-3.259534	-1.392116
H	-2.797887	-3.068956	-0.867685
H	-1.990611	-2.921732	-2.430740
C	0.609970	-2.812911	-1.508777
H	0.532513	-2.441978	-2.544348
H	1.457046	-2.278571	-1.047512
C	-1.404058	-5.265274	0.052231
H	-2.329379	-5.081240	0.624236
H	-1.230359	-6.354312	0.063842
P	-0.820964	-0.621840	-0.717932
Pd	1.123608	0.440601	-0.111800
C	1.011589	3.271261	2.016953
C	1.248153	3.413057	0.654628
C	2.132788	2.531515	-0.008790
C	2.527895	1.376697	2.107995
C	1.651182	2.240816	2.737120
H	0.774823	4.201665	0.070631
H	2.426440	2.755565	-1.035965
H	3.044522	0.606869	2.683270
H	1.438344	2.134014	3.802082
C	2.803543	1.522275	0.721103
Br	4.366233	0.681109	-0.004964
O	0.174989	4.058202	2.740131
C	-0.515219	5.087417	2.067491
H	-1.143647	4.695231	1.250116
H	-1.162421	5.566898	2.812617
H	0.178614	5.841665	1.658719

int-2-cf3.log

SCF (RwB97XD) = -4549.27668154
E(SCF)+ZPE(0 K)= -4548.583451
H(298 K)= -4548.547718
G(298 K)= -4548.652396
Lowest Frequency = 11.1034cm-1

C	-2.301363	-0.122397	-1.912832
H	-1.929204	-0.835373	-2.661962
H	-3.377711	-0.330019	-1.819433
C	-2.056055	1.299153	-2.432170
H	-2.848699	1.556968	-3.155795
H	-2.145209	2.026476	-1.607016
C	-0.694754	1.464976	-3.104385
H	0.094809	1.149810	-2.399725
H	-0.631775	0.777146	-3.965772
C	-0.421056	2.889400	-3.564575
H	0.555183	2.969117	-4.067189
H	-0.419767	3.588445	-2.712396
H	-1.187845	3.238279	-4.275754
C	-2.876301	-0.460468	0.943183
C	-3.949503	-1.556823	0.810143
H	-3.495279	-2.544805	0.978912

H	-4.375598	-1.564325	-0.206944
C	-5.067409	-1.344716	1.845856
H	-5.822351	-2.137637	1.721415
C	-4.467538	-1.418351	3.257054
H	-4.010888	-2.408824	3.422224
H	-5.261297	-1.300552	4.013792
C	-3.411219	-0.316241	3.420583
H	-2.966796	-0.373204	4.426977
C	-4.065607	1.056090	3.220912
H	-4.852412	1.218732	3.976454
H	-3.317086	1.854731	3.354964
C	-4.662605	1.126758	1.810373
H	-5.122896	2.114648	1.648788
C	-3.551992	0.924202	0.769901
H	-3.986887	1.020450	-0.236702
H	-2.791187	1.717791	0.869305
C	-5.720402	0.027976	1.637580
H	-6.540413	0.171127	2.360986
H	-6.166618	0.086854	0.630419
C	-2.298647	-0.510205	2.378477
H	-1.532267	0.275288	2.490171
H	-1.793217	-1.469324	2.559555
C	-0.834633	-2.248565	-0.591562
C	-0.346943	-2.867859	0.734680
H	0.333462	-2.165664	1.246028
H	-1.203409	-3.037929	1.405449
C	0.357151	-4.210177	0.481330
H	0.693466	-4.622688	1.446137
C	1.564874	-4.000196	-0.441151
H	2.286415	-3.311994	0.030736
H	2.089713	-4.956440	-0.604328
C	1.085860	-3.420361	-1.778216
H	1.948450	-3.252445	-2.442888
C	0.097148	-4.387606	-2.441445
H	0.586257	-5.354745	-2.645781
H	-0.233871	-3.982638	-3.412646
C	-1.108573	-4.590885	-1.515142
H	-1.827881	-5.279052	-1.987715
C	-1.808043	-3.242184	-1.265077
H	-2.691947	-3.408879	-0.635006
H	-2.171167	-2.842288	-2.224358
C	0.394213	-2.075565	-1.522132
H	0.089077	-1.628287	-2.482149
H	1.102704	-1.369534	-1.061858
C	-0.632183	-5.179910	-0.179958
H	-1.493208	-5.354765	0.487145
H	-0.151730	-6.158387	-0.347317
P	-1.478274	-0.511540	-0.302545
Pd	0.196449	0.941285	0.374156
C	3.120936	1.582549	-1.377650
C	3.136113	0.706150	-0.299213
C	2.448756	1.019342	0.889019
C	1.707674	3.119580	-0.148073
C	2.391015	2.781700	-1.306429
H	3.695047	-0.229580	-0.353359
H	2.600003	0.401124	1.775297
H	1.183285	4.073415	-0.078786
H	2.358669	3.458228	-2.161512
C	1.749787	2.254131	0.979248
Br	1.246766	2.941455	2.692390
C	3.784842	1.192357	-2.666522
F	2.936466	0.530484	-3.487435
F	4.839253	0.378386	-2.479264
F	4.226539	2.256539	-3.361476

int-2-cl.log

SCF (RwB97XD) = -4671.82382520
E(SCF)+ZPE(0 K)= -4671.145058
H(298 K)= -4671.111593
G(298 K)= -4671.210466
Lowest Frequency = 16.6529cm-1

C	-1.750781	-0.326563	-2.277473
H	-1.418826	-1.139021	-2.940366
H	-2.845827	-0.420959	-2.218705
C	-1.343568	1.010893	-2.910130
H	-2.095581	1.288997	-3.668736
H	-1.357894	1.813814	-2.155387
C	0.034231	0.975386	-3.566094
H	0.775668	0.638450	-2.819226
H	0.034205	0.216601	-4.368756
C	0.462708	2.323832	-4.127618
H	1.460169	2.269653	-4.590502
H	0.501697	3.087680	-3.334756
H	-0.240934	2.683400	-4.896376
C	-2.408658	-0.168840	0.577327
C	-3.654453	-1.075682	0.570805
H	-3.381022	-2.095117	0.881241
H	-4.076177	-1.148207	-0.445514
C	-4.719957	-0.535786	1.541126
H	-5.596607	-1.202435	1.511613
C	-4.141251	-0.506885	2.962728
H	-3.855902	-1.525452	3.276083
H	-4.904512	-0.152862	3.675819
C	-2.915522	0.416547	2.995135
H	-2.488378	0.431320	4.010678
C	-3.328585	1.835566	2.586226
H	-4.076311	2.235703	3.291439
H	-2.456026	2.509132	2.623277
C	-3.904215	1.800706	1.165610
H	-4.190295	2.818092	0.853988
C	-2.845149	1.266664	0.190216
H	-3.266255	1.280324	-0.826660
H	-1.964103	1.930448	0.184615
C	-5.133334	0.882232	1.127390
H	-5.915178	1.262916	1.805715
H	-5.566157	0.869853	0.112859
C	-1.850996	-0.106798	2.019664
H	-0.964346	0.547678	2.035896
H	-1.512567	-1.099797	2.347067
C	-0.766562	-2.508237	-0.637614
C	-0.495270	-3.039183	0.786161
H	0.300393	-2.440703	1.261670
H	-1.397313	-2.932192	1.407592
C	-0.096896	-4.522532	0.747082
H	0.085727	-4.867490	1.777402
C	1.175800	-4.698282	-0.091093
H	2.007072	-4.129734	0.358049
H	1.478511	-5.758825	-0.104232
C	0.912803	-4.204098	-1.519491
H	1.826759	-4.312604	-2.125048
C	-0.226700	-5.017437	-2.146879
H	0.049280	-6.084080	-2.198092
H	-0.406664	-4.680999	-3.181899
C	-1.497426	-4.839573	-1.306135
H	-2.322949	-5.415022	-1.755242
C	-1.893611	-3.352897	-1.273807
H	-2.829813	-3.241509	-0.710755
H	-2.097991	-3.014864	-2.301293
C	0.521321	-2.720621	-1.476122
H	0.374702	-2.346204	-2.503026
H	1.342860	-2.127405	-1.039301
C	-1.240291	-5.335232	0.123503
H	-2.155311	-5.229247	0.730581
H	-0.983757	-6.407784	0.113320
P	-1.033479	-0.652568	-0.605597
Pd	0.933567	0.455407	-0.100623
C	1.847413	3.841006	-0.601407
C	2.740083	3.081922	-1.375700
C	3.208379	1.869675	-0.896219
C	1.860828	2.154132	1.134865
C	1.399860	3.389014	0.627541
H	3.065960	3.445623	-2.350847
H	3.931412	1.295284	-1.477165
H	1.645614	1.895570	2.173133
H	0.709525	3.988971	1.222268
C	2.794380	1.397641	0.378176
Br	3.827827	0.024685	1.226524
Cl	1.278385	5.365141	-1.218230

int-2-cn.log

SCF (RwB97XD) = -4304.41483638
E(SCF)+ZPE(0 K)= -4303.727363
H(298 K)= -4303.693502
G(298 K)= -4303.792857
Lowest Frequency = 14.3090cm-1

C	-1.861049	-0.129451	-2.200300
H	-1.476568	-0.885828	-2.899964
H	-2.945522	-0.307565	-2.136373
C	-1.565043	1.259948	-2.778469
H	-2.333256	1.494768	-3.535467
H	-1.661295	2.031146	-1.997135
C	-0.186659	1.378038	-3.423794
H	0.585060	1.122107	-2.675273
H	-0.099640	0.627168	-4.229110
C	0.084407	2.766943	-3.984906
H	1.066611	2.824727	-4.476426
H	0.066773	3.528459	-3.188366
H	-0.675678	3.054188	-4.730014
C	-2.495684	-0.141648	0.667262
C	-3.679092	-1.127972	0.633838
H	-3.334418	-2.139534	0.894902
H	-4.108583	-1.183190	-0.380259
C	-4.764474	-0.703144	1.638526
H	-5.596014	-1.424326	1.588347
C	-4.169087	-0.698242	3.053505
H	-3.814743	-1.708668	3.318929
H	-4.943483	-0.426339	3.790180
C	-3.006089	0.301904	3.114057
H	-2.566483	0.300044	4.124156
C	-3.518006	1.706686	2.774468
H	-4.281313	2.023454	3.504827
H	-2.691974	2.435048	2.833882
C	-4.109466	1.696349	1.359866
H	-4.466016	2.705228	1.097161
C	-3.031290	1.277317	0.349801
H	-3.466082	1.306732	-0.660923
H	-2.196182	1.998395	0.364792
C	-5.275644	0.701204	1.292666
H	-6.071996	0.999445	1.994858
H	-5.719619	0.705132	0.282872
C	-1.922371	-0.105172	2.104793
H	-1.081877	0.608207	2.143499
H	-1.512783	-1.085654	2.384957
C	-0.713915	-2.307779	-0.666655
C	-0.383283	-2.877249	0.729520
H	0.378016	-2.246688	1.220388
H	-1.280088	-2.857251	1.366862
C	0.113188	-4.327341	0.622215
H	0.337858	-4.700566	1.634136
C	1.379068	-4.381819	-0.242572
H	2.177996	-3.775922	0.216819
H	1.753873	-5.417163	-0.303955
C	1.056517	-3.850049	-1.644850
H	1.963612	-3.872054	-2.269523
C	-0.037510	-4.711929	-2.287571
H	0.308486	-5.754263	-2.387818
H	-0.258373	-4.346790	-3.304690
C	-1.301341	-4.653934	-1.420092
H	-2.095456	-5.264300	-1.879303
C	-1.794336	-3.199287	-1.319900
H	-2.725118	-3.173566	-0.737818
H	-2.039776	-2.833440	-2.328778
C	0.569530	-2.398966	-1.533563
H	0.381062	-1.993388	-2.541378
H	1.358640	-1.770724	-1.086698
C	-0.984945	-5.189199	-0.016667
H	-1.893035	-5.170625	0.609460
H	-0.657968	-6.240791	-0.076276
P	-1.110081	-0.478877	-0.551124
Pd	0.793789	0.705791	0.063373
C	2.914214	3.102872	-1.483824
C	3.633305	1.896949	-1.334858
C	3.464724	1.120184	-0.201860
C	1.796559	2.719218	0.642512
C	1.982532	3.489919	-0.518219
H	4.334905	1.582278	-2.108795
H	4.058068	0.215554	-0.063035
H	1.197018	3.121766	1.460523
H	1.418984	4.416296	-0.639816
C	2.573583	1.542314	0.824480
Br	2.766590	0.788474	2.572030
C	3.137068	3.927340	-2.636877
N	3.321069	4.588561	-3.561752

int-2-h.log

SCF (RwB97XD) = -4212.20898927
E(SCF)+ZPE(0 K)= -4211.520582
H(298 K)= -4211.488374
G(298 K)= -4211.584036
Lowest Frequency = 18.0264cm-1

C	-1.694643	0.027337	-2.302660
H	-1.310588	-0.739929	-2.990734
H	-2.781119	-0.142259	-2.245785
C	-1.388385	1.406275	-2.901714
H	-2.144342	1.624376	-3.676509
H	-1.497998	2.192027	-2.136940
C	0.001412	1.516069	-3.524190
H	0.759453	1.304079	-2.748839
H	0.115791	0.734619	-4.296518
C	0.266826	2.884385	-4.137199
H	1.275175	2.941989	-4.574985
H	0.186294	3.679644	-3.378948
H	-0.456154	3.114863	-4.937440
C	-2.350747	0.054715	0.558219
C	-3.548969	-0.913547	0.524674
H	-3.222571	-1.927932	0.797942
H	-3.971352	-0.971924	-0.492316
C	-4.636188	-0.463374	1.516388
H	-5.477831	-1.172884	1.466639
C	-4.052516	-0.453001	2.936253
H	-3.714768	-1.465802	3.214188
H	-4.829268	-0.162914	3.663667
C	-2.875368	0.530482	2.996393
H	-2.444552	0.532661	4.010383
C	-3.363722	1.939160	2.638484
H	-4.128141	2.274858	3.359403
H	-2.527183	2.655406	2.696765
C	-3.943688	1.923124	1.219166
H	-4.283326	2.934586	0.943664
C	-2.863528	1.477757	0.222597
H	-3.290296	1.502583	-0.791808
H	-2.017536	2.185923	0.236391
C	-5.124153	0.944758	1.152934
H	-5.921402	1.261616	1.846106
H	-5.560343	0.944866	0.139658
C	-1.789698	0.097003	2.000319
H	-0.937077	0.795529	2.037088
H	-1.398375	-0.887317	2.293055
C	-0.601237	-2.153428	-0.742172
C	-0.298390	-2.718878	0.661835
H	0.471475	-2.100509	1.154147
H	-1.200942	-2.673812	1.289873
C	0.166308	-4.180707	0.572454
H	0.370810	-4.550502	1.589977
C	1.440597	-4.271029	-0.276857
H	2.247286	-3.679235	0.186951
H	1.792254	-5.315303	-0.325874
C	1.146205	-3.743470	-1.686858
H	2.059956	-3.790503	-2.300565
C	0.041220	-4.587085	-2.335274
H	0.364822	-5.637954	-2.422234
H	-0.159753	-4.226158	-3.358131
C	-1.231127	-4.493341	-1.483585
H	-2.033204	-5.090124	-1.947204

C	-1.692542	-3.027254	-1.401310
H	-2.630005	-2.976496	-0.831913
H	-1.917049	-2.665541	-2.416532
C	0.689714	-2.281107	-1.592860
H	0.521119	-1.880285	-2.606198
H	1.486127	-1.665246	-1.142062
C	-0.943343	-5.023383	-0.072122
H	-1.858303	-4.978412	0.542655
H	-0.639770	-6.082736	-0.118865
P	-0.954714	-0.312224	-0.642817
Pd	0.950476	0.854163	-0.080886
C	2.748730	3.496620	-1.662534
C	3.553315	2.348121	-1.711858
C	3.591132	1.461825	-0.643168
C	1.960997	2.858064	0.550411
C	1.947045	3.734753	-0.555414
H	4.164499	2.144373	-2.593713
H	4.252004	0.593835	-0.663452
H	1.456055	3.143807	1.474985
H	1.312530	4.622759	-0.512803
C	2.818540	1.728794	0.517481
Br	3.268131	0.822908	2.147434
H	2.744952	4.195550	-2.500661

int-2-me.log

SCF (RwB97XD) = -4251.48881100
E(SCF)+ZPE(0 K)= -4250.772777
H(298 K)= -4250.738783
G(298 K)= -4250.838749
Lowest Frequency = 13.5013cm-1

C	-1.696199	-0.166543	-2.326562
H	-1.353809	-0.976767	-2.986800
H	-2.789573	-0.277590	-2.263831
C	-1.313223	1.173100	-2.968855
H	-2.070387	1.432375	-3.729395
H	-1.341730	1.980052	-2.218799
C	0.065527	1.158697	-3.623768
H	0.812155	0.843578	-2.873188
H	0.080243	0.394959	-4.421651
C	0.466710	2.512256	-4.192526
H	1.466268	2.477593	-4.652754
H	0.489322	3.276085	-3.399697
H	-0.242875	2.854836	-4.963858
C	-2.351721	-0.015106	0.527473
C	-3.584490	-0.939525	0.519454
H	-3.296469	-1.954941	0.829675
H	-4.004356	-1.017781	-0.497231
C	-4.658767	-0.416603	1.489274
H	-5.525844	-1.095731	1.458022
C	-4.081709	-0.381306	2.911397
H	-3.782266	-1.396249	3.223402
H	-4.850537	-0.039245	3.624492
C	-2.869239	0.559507	2.945948
H	-2.443126	0.579041	3.961826
C	-3.303288	1.972855	2.539306
H	-4.058033	2.360111	3.244407
H	-2.440942	2.659150	2.578757
C	-3.876726	1.931851	1.117916
H	-4.177666	2.945592	0.807826
C	-2.808952	1.414671	0.142892
H	-3.229128	1.423894	-0.874577
H	-1.936542	2.090082	0.139621
C	-5.092367	0.995810	1.076942
H	-5.880588	1.364277	1.754760
H	-5.523623	0.978396	0.061736
C	-1.796388	0.053303	1.970483
H	-0.918173	0.719360	1.988229
H	-1.444099	-0.935093	2.296946
C	-0.674508	-2.327351	-0.687447
C	-0.393123	-2.852995	0.736288
H	0.394870	-2.242547	1.209175
H	-1.295514	-2.757758	1.359135
C	0.024769	-4.330931	0.699063
H	0.214583	-4.671622	1.729563
C	1.297510	-4.491597	-0.142006
H	2.121978	-3.910547	0.303623
H	1.614586	-5.548036	-0.153561
C	1.023927	-4.004004	-1.570622
H	1.937618	-4.101672	-2.178534
C	-0.106158	-4.833788	-2.193349
H	0.183288	-5.897035	-2.242941
H	-0.293008	-4.501845	-3.228640
C	-1.376844	-4.670041	-1.349748
H	-2.196319	-5.256848	-1.795421
C	-1.791745	-3.188279	-1.319338
H	-2.728269	-3.088603	-0.754808
H	-2.002928	-2.855560	-2.347189
C	0.613443	-2.525691	-1.529169
H	0.458789	-2.155439	-2.556442
H	1.427530	-1.919652	-1.096812
C	-1.109537	-5.159726	0.080080
H	-2.024265	-5.064243	0.689396
H	-0.839444	-6.229032	0.071144
P	-0.965892	-0.472942	-0.655274
Pd	0.978199	0.647352	-0.146062
C	2.412861	3.794183	-1.158175
C	3.224040	2.761973	-1.667386
C	3.478748	1.606845	-0.941306
C	2.063217	2.456595	0.863242
C	1.827135	3.613025	0.092163
H	3.670853	2.869585	-2.659042
H	4.143161	0.838776	-1.340039
H	1.724062	2.422231	1.900165
H	1.186506	4.393471	0.511374
C	2.922409	1.452903	0.352924
Br	3.672117	0.136946	1.529903
C	2.198102	5.064086	-1.938218
H	1.167222	5.433598	-1.831876
H	2.869147	5.864410	-1.584639
H	2.399654	4.918468	-3.009090

int-2-nme2.log

SCF (RwB97XD) = -4346.10490917
E(SCF)+ZPE(0 K)= -4345.343366
H(298 K)= -4345.306772
G(298 K)= -4345.412263
Lowest Frequency = 14.3898cm-1

C	-1.937373	0.044751	-2.045300
H	-1.497123	-0.619297	-2.803203
H	-3.014530	-0.181937	-2.044415
C	-1.680627	1.496074	-2.469696
H	-2.446127	1.789968	-3.209248
H	-1.804960	2.176252	-1.611193
C	-0.297130	1.715891	-3.076762
H	0.468194	1.397132	-2.347004
H	-0.183160	1.061498	-3.959609
C	-0.045123	3.163312	-3.474048
H	0.940533	3.282025	-3.948265
H	-0.070994	3.820379	-2.589729
H	-0.807504	3.527394	-4.182927
C	-2.695020	-0.326256	0.758929
C	-3.824262	-1.351167	0.539490
H	-3.444022	-2.370587	0.701984
H	-4.195656	-1.304604	-0.497894
C	-4.983088	-1.099331	1.519923
H	-5.773697	-1.844352	1.333931
C	-4.466640	-1.242368	2.958679
H	-4.076193	-2.261488	3.120532
H	-5.292766	-1.097414	3.675195
C	-3.359760	-0.208090	3.207648
H	-2.975711	-0.314506	4.234841
C	-3.922347	1.204384	3.008885
H	-4.740080	1.396550	3.723927
H	-3.137323	1.953553	3.204405
C	-4.434628	1.342321	1.570281
H	-4.826647	2.359695	1.410130

C	-3.284001	1.096588	0.583504
H	-3.664141	1.232305	-0.440760
H	-2.487210	1.844932	0.734809
C	-5.544506	0.313391	1.314798
H	-6.392386	0.487417	1.998541
H	-5.932359	0.422125	0.287679
C	-2.204398	-0.441314	2.222496
H	-1.402061	0.294938	2.394567
H	-1.763189	-1.430837	2.406749
C	-0.741735	-2.230565	-0.720723
C	-0.447325	-2.947358	0.613825
H	0.261925	-2.347025	1.208680
H	-1.370956	-3.037905	1.205239
C	0.117175	-4.354351	0.364937
H	0.312881	-4.835355	1.336719
C	1.421095	-4.257325	-0.436431
H	2.169291	-3.677990	0.129817
H	1.844596	-5.263127	-0.597638
C	1.137950	-3.577187	-1.782570
H	2.073261	-3.491354	-2.359095
C	0.114258	-4.402697	-2.572892
H	0.510306	-5.412447	-2.774161
H	-0.078078	-3.931505	-3.551566
C	-1.188326	-4.495600	-1.768328
H	-1.932766	-5.079371	-2.333691
C	-1.749667	-3.083078	-1.525463
H	-2.706284	-3.163274	-0.992810
H	-1.965762	-2.612892	-2.497092
C	0.580679	-2.169771	-1.528886
H	0.415707	-1.657834	-2.491790
H	1.316016	-1.563603	-0.975595
C	-0.911008	-5.178857	-0.422115
H	-1.846045	-5.269946	0.156490
H	-0.534233	-6.202510	-0.586159
P	-1.225009	-0.440905	-0.407838
Pd	0.507872	0.828462	0.378935
C	2.866070	3.038977	-1.262661
C	3.232318	1.747654	-0.845894
C	2.842952	1.242170	0.406040
C	1.694146	3.334816	0.865775
C	2.082660	3.818776	-0.371728
H	3.842538	1.106515	-1.479914
H	3.265909	0.295010	0.746128
H	1.122947	3.975220	1.539781
H	1.763071	4.821432	-0.651846
C	2.083300	2.042090	1.290698
Br	2.006809	1.615576	3.159084
N	3.232015	3.533001	-2.495206
C	3.083160	4.942097	-2.775806
H	3.456904	5.151278	-3.785225
H	2.026370	5.254136	-2.749989
H	3.645116	5.577020	-2.066256
C	4.057955	2.730790	-3.364375
H	3.590771	1.752462	-3.561804
H	4.172118	3.239140	-4.329518
H	5.069071	2.550086	-2.952043

int-2-ome.log

SCF (RwB97XD) = -4326.70071999
E(SCF)+ZPE(0 K)= -4325.979550
H(298 K)= -4325.944755
G(298 K)= -4326.046944
Lowest Frequency = 9.3483cm-1

C	-1.579617	-0.295534	-2.375113
H	-1.195517	-1.099847	-3.019393
H	-2.675254	-0.399131	-2.392286
C	-1.142758	1.049293	-2.970239
H	-1.836690	1.319922	-3.785251
H	-1.226795	1.848426	-2.216270
C	0.283009	1.035606	-3.514823
H	0.965670	0.697024	-2.714988
H	0.354794	0.289841	-4.326444
C	0.738704	2.397723	-4.018794
H	1.768716	2.358351	-4.406367
H	0.708454	3.142351	-3.207281
H	0.092035	2.767506	-4.831713
C	-2.436767	-0.166296	0.425567
C	-3.681389	-1.067779	0.312287
H	-3.435212	-2.092357	0.628695
H	-4.026505	-1.124735	-0.733656
C	-4.816227	-0.537789	1.206797
H	-5.690165	-1.200522	1.100910
C	-4.347175	-0.532144	2.668592
H	-4.089566	-1.556427	2.987076
H	-5.161254	-0.185943	3.327391
C	-3.124359	0.385341	2.807815
H	-2.775368	0.382753	3.853002
C	-3.501191	1.811961	2.390631
H	-4.299232	2.205389	3.042680
H	-2.631725	2.480885	2.503659
C	-3.967690	1.800347	0.930161
H	-4.226521	2.823590	0.613482
C	-2.839712	1.276547	0.029859
H	-3.183469	1.306240	-1.015393
H	-1.958300	1.936110	0.100910
C	-5.193092	0.887820	0.784412
H	-6.023739	1.261378	1.406533
H	-5.546435	0.892450	-0.260667
C	-1.990702	-0.128511	1.907288
H	-1.103655	0.519359	2.001171
H	-1.682954	-1.128070	2.244627
C	-0.705527	-2.487110	-0.694931
C	-0.532214	-3.031564	0.738717
H	0.228068	-2.436549	1.272634
H	-1.475393	-2.930206	1.296672
C	-0.131960	-4.514510	0.714490
H	-0.019297	-4.867673	1.752069
C	1.194565	-4.683684	-0.036289
H	1.992062	-4.118055	0.473660
H	1.498364	-5.744101	-0.036901
C	1.028696	-4.177828	-1.475510
H	1.981584	-4.283190	-2.018832
C	-0.066106	-4.985860	-2.184614
H	0.212349	-6.052434	-2.224845
H	-0.176551	-4.641833	-3.226942
C	-1.390658	-4.812989	-1.430714
H	-2.184375	-5.384033	-1.939056
C	-1.786981	-3.325766	-1.413259
H	-2.759248	-3.218631	-0.914469
H	-1.920831	-2.979065	-2.449515
C	0.635781	-2.694454	-1.446270
H	0.558716	-2.312100	-2.477906
H	1.424960	-2.104285	-0.950795
C	-1.230597	-5.321370	0.008686
H	-2.184123	-5.220221	0.554463
H	-0.973841	-6.394029	0.005824
P	-0.973529	-0.629450	-0.659705
Pd	0.920811	0.487682	0.003792
C	2.265820	3.748989	-0.780977
C	3.190921	2.797494	-1.245857
C	3.429426	1.638825	-0.515528
C	1.789627	2.347263	1.149623
C	1.562510	3.511651	0.398582
H	3.732245	2.947686	-2.179405
H	4.180004	0.926817	-0.861995
H	1.348176	2.260600	2.143929
H	0.847569	4.256893	0.751123
C	2.756758	1.408326	0.706574
Br	3.471174	0.112768	1.927587
O	1.989731	4.914112	-1.420896
C	2.789052	5.278908	-2.525602
H	2.667191	4.581935	-3.370746
H	2.447234	6.273396	-2.839695
H	3.856095	5.337806	-2.252498

int-3-cf3.log

SCF (RwB97XD) = -4549.28482099

E(SCF)+ZPE(0 K)= -4548.591349
H(298 K)= -4548.555626
G(298 K)= -4548.660767
Lowest Frequency = 10.0692cm-1

C	-2.259316	-0.746899	-1.935349
H	-1.849268	-1.497532	-2.626928
H	-3.337992	-0.953789	-1.869384
C	-2.007653	0.645201	-2.523812
H	-2.639963	0.767970	-3.419163
H	-2.325780	1.428984	-1.819480
C	-0.561180	0.897990	-2.932744
H	0.159135	0.615930	-2.112219
H	-0.267866	0.192700	-3.728276
C	-0.263016	2.327019	-3.350320
H	0.800534	2.458966	-3.596553
H	-0.508331	3.036313	-2.545150
H	-0.854219	2.603998	-4.237160
C	-2.820234	-0.694080	0.958488
C	-3.984650	-1.704499	0.964662
H	-3.614579	-2.703447	1.239151
H	-4.432496	-1.787926	-0.039735
C	-5.057130	-1.277908	1.982333
H	-5.876411	-2.014311	1.962078
C	-4.433039	-1.234440	3.384225
H	-4.054983	-2.233014	3.660934
H	-5.197392	-0.962804	4.131161
C	-3.288015	-0.212456	3.401601
H	-2.826866	-0.186945	4.401261
C	-3.831770	1.176185	3.046617
H	-4.586431	1.493716	3.785349
H	-3.017752	1.919345	3.076252
C	-4.451963	1.127710	1.645352
H	-4.831371	2.124512	1.369397
C	-3.388866	0.706967	0.619699
H	-3.851556	0.701809	-0.378968
H	-2.570078	1.447344	0.599790
C	-5.600340	0.110203	1.619410
H	-6.387770	0.407564	2.331600
H	-6.064505	0.087209	0.618854
C	-2.215076	-0.617529	2.380213
H	-1.387084	0.106315	2.394875
H	-1.782946	-1.584244	2.671543
C	-0.991528	-2.842605	-0.356341
C	-0.615549	-3.356496	1.050595
H	0.122323	-2.681822	1.514244
H	-1.506218	-3.361426	1.696581
C	-0.059087	-4.786451	0.968973
H	0.198894	-5.121651	1.985869
C	1.193797	-4.808653	0.084242
H	1.971953	-4.156315	0.513500
H	1.612178	-5.827964	0.042270
C	0.825606	-4.330045	-1.325886
H	1.722393	-4.329903	-1.965335
C	-0.242367	-5.252272	-1.927239
H	0.145785	-6.281058	-2.008434
H	-0.496198	-4.922920	-2.949076
C	-1.492548	-5.226966	-1.038350
H	-2.267185	-5.882265	-1.467828
C	-2.046584	-3.792939	-0.966412
H	-2.965703	-3.789697	-0.364965
H	-2.326760	-3.464975	-1.979554
C	0.280376	-2.897509	-1.243204
H	0.056286	-2.530633	-2.259195
H	1.053058	-2.232552	-0.823794
C	-1.129051	-5.711252	0.372222
H	-2.026440	-5.716310	1.013783
H	-0.757835	-6.748835	0.331865
P	-1.473582	-1.041308	-0.286124
Pd	0.433844	0.452587	-0.223961
C	3.647641	3.797806	-1.329830
C	3.939537	2.455379	-1.591053
C	3.063834	1.459147	-1.167816
C	1.611938	3.135886	-0.215963
C	2.485291	4.138596	-0.637700
H	4.856833	2.188050	-2.120934
H	3.310247	0.412741	-1.365733
H	0.712946	3.415288	0.340515
H	2.261341	5.184027	-0.418117
C	1.880280	1.793124	-0.498374
Br	1.478021	0.041922	1.927023
C	4.587723	4.858980	-1.831444
F	4.482920	5.034621	-3.169125
F	5.878284	4.551013	-1.596617
F	4.364283	6.059848	-1.271556

int-3-cl.log

SCF (RwB97XD) = -4671.83325107
E(SCF)+ZPE(0 K)= -4671.154160
H(298 K)= -4671.120776
G(298 K)= -4671.220236
Lowest Frequency = 12.1342cm-1

C	-1.981962	-0.406840	-2.057636
H	-1.581045	-1.156139	-2.756150
H	-3.066268	-0.587981	-2.008870
C	-1.688276	0.987801	-2.621290
H	-2.307889	1.140209	-3.521041
H	-1.993865	1.768819	-1.908412
C	-0.231599	1.209118	-3.011201
H	0.468553	0.914093	-2.179928
H	0.055762	0.500199	-3.805676
C	0.104099	2.632529	-3.419352
H	1.173788	2.740754	-3.650219
H	-0.136287	3.344611	-2.615126
H	-0.468188	2.926279	-4.313317
C	-2.574805	-0.377577	0.830554
C	-3.763274	-1.359599	0.818612
H	-3.419499	-2.370148	1.084293
H	-4.205443	-1.421515	-0.189840
C	-4.832507	-0.918083	1.833028
H	-5.668331	-1.635108	1.800364
C	-4.217811	-0.902002	3.239641
H	-3.864554	-1.911385	3.509912
H	-4.981305	-0.619743	3.983564
C	-3.049879	0.093178	3.274612
H	-2.595751	0.099557	4.277780
C	-3.558850	1.497171	2.928211
H	-4.311939	1.825208	3.663967
H	-2.728447	2.221303	2.970829
C	-4.168865	1.475570	1.521880
H	-4.523080	2.483296	1.251991
C	-3.107583	1.039401	0.500589
H	-3.561890	1.054689	-0.501838
H	-2.271549	1.760528	0.494417
C	-5.340589	0.485477	1.478956
H	-6.125709	0.795276	2.188447
H	-5.798248	0.481763	0.475164
C	-1.978933	-0.327316	2.257405
H	-1.135567	0.378252	2.284502
H	-1.569519	-1.305600	2.542978
C	-0.807212	-2.566990	-0.494367
C	-0.473641	-3.115810	0.910240
H	0.282139	-2.477043	1.395187
H	-1.373574	-3.096848	1.543054
C	0.030493	-4.564274	0.815176
H	0.258551	-4.924173	1.830785
C	1.296787	-4.619263	-0.048893
H	2.090884	-4.002865	0.403617
H	1.678112	-5.652638	-0.100242
C	0.970540	-4.105776	-1.457072
H	1.877549	-4.128720	-2.081552
C	-0.119599	-4.979326	-2.090380
H	0.232202	-6.020463	-2.180265
H	-0.343329	-4.626260	-3.111336
C	-1.383325	-4.920923	-1.222457
H	-2.174101	-5.540405	-1.675078
C	-1.885343	-3.468589	-1.137358
H	-2.814664	-3.440224	-0.552402

H	-2.134567	-3.115171	-2.150048
C	0.476790	-2.655302	-1.360746
H	0.283694	-2.264541	-2.374216
H	1.265623	-2.025575	-0.918033
C	-1.062436	-5.439490	0.186283
H	-1.970310	-5.420633	0.812659
H	-0.729216	-6.489502	0.136161
P	-1.225249	-0.750017	-0.404168
Pd	0.733499	0.672296	-0.289913
C	4.034348	3.934198	-1.337587
C	4.306825	2.594894	-1.609501
C	3.401949	1.617038	-1.193788
C	1.986674	3.317822	-0.237347
C	2.883410	4.306096	-0.647074
H	5.221046	2.318072	-2.137959
H	3.627363	0.567298	-1.400322
H	1.091803	3.616142	0.316608
H	2.692133	5.357586	-0.424915
C	2.223647	1.970737	-0.529019
Br	1.761668	0.166328	1.848471
Cl	5.158274	5.162469	-1.858240

int-3-cn.log

SCF (RwB97XD) = -4304.42468264
E(SCF)+ZPE(0 K)= -4303.737304
H(298 K)= -4303.703355
G(298 K)= -4303.803588
Lowest Frequency = 12.0896cm-1

C	-1.962191	-0.353832	-2.093294
H	-1.555854	-1.101798	-2.790055
H	-3.044792	-0.543997	-2.042177
C	-1.681728	1.041610	-2.660977
H	-2.300416	1.184045	-3.562888
H	-1.997129	1.822246	-1.952170
C	-0.227331	1.277122	-3.050136
H	0.479086	0.988210	-2.219868
H	0.068629	0.569357	-3.842365
C	0.094692	2.702868	-3.460642
H	1.163125	2.821452	-3.692227
H	-0.154334	3.414521	-2.658690
H	-0.480036	2.988094	-4.355628
C	-2.548859	-0.316539	0.796754
C	-3.731965	-1.305188	0.788652
H	-3.382405	-2.312948	1.057288
H	-4.174672	-1.372586	-0.219182
C	-4.802644	-0.866115	1.802635
H	-5.634695	-1.587576	1.772533
C	-4.186790	-0.842968	3.208585
H	-3.828259	-1.849783	3.481363
H	-4.950960	-0.562453	3.952386
C	-3.024009	0.158351	3.239749
H	-2.568949	0.169705	4.242417
C	-3.540444	1.558735	2.889941
H	-4.294404	1.885008	3.625500
H	-2.713721	2.287260	2.929798
C	-4.151869	1.530087	1.484375
H	-4.511476	2.535183	1.211988
C	-3.089711	1.096507	0.462918
H	-3.546028	1.105753	-0.538622
H	-2.257766	1.822390	0.453013
C	-5.318373	0.533765	1.445105
H	-6.104460	0.841313	2.154412
H	-5.776874	0.525044	0.441747
C	-1.951664	-0.259230	2.222760
H	-1.112427	0.451310	2.247338
H	-1.536866	-1.234525	2.510697
C	-0.768077	-2.499865	-0.523350
C	-0.429042	-3.041033	0.882956
H	0.322888	-2.395447	1.364861
H	-1.328448	-3.026024	1.516553
C	0.085063	-4.486272	0.792293
H	0.316984	-4.840794	1.808876
C	1.350462	-4.535580	-0.073387
H	2.141052	-3.912230	0.375779
H	1.738819	-5.566410	-0.121630
C	1.018732	-4.029563	-1.483000
H	1.924966	-4.048578	-2.108685
C	-0.066372	-4.912687	-2.111591
H	0.292480	-5.951607	-2.198385
H	-0.294007	-4.564792	-3.133437
C	-1.329151	-4.860033	-1.241930
H	-2.116272	-5.486509	-1.691155
C	-1.841212	-3.411010	-1.161411
H	-2.769641	-3.386823	-0.574876
H	-2.094561	-3.063134	-2.174964
C	0.515292	-2.582090	-1.391304
H	0.318090	-2.196419	-2.405959
H	1.300773	-1.946009	-0.951665
C	-1.002718	-5.371239	0.168185
H	-1.909779	-5.356466	0.795802
H	-0.662298	-6.419026	0.121298
P	-1.201159	-0.686804	-0.439967
Pd	0.741856	0.753904	-0.345331
C	4.023141	4.049369	-1.395396
C	4.297836	2.700311	-1.666376
C	3.403092	1.717514	-1.253810
C	1.973531	3.410394	-0.287786
C	2.860719	4.402530	-0.694531
H	5.211818	2.428915	-2.198689
H	3.630864	0.669145	-1.462691
H	1.075751	3.696675	0.266958
H	2.659245	5.451843	-0.469584
C	2.221713	2.064905	-0.586989
Br	1.774487	0.269592	1.795294
C	4.934716	5.070528	-1.833872
N	5.661774	5.888881	-2.191032

int-3-h.log

SCF (RwB97XD) = -4212.21778966
E(SCF)+ZPE(0 K)= -4211.528939
H(298 K)= -4211.496877
G(298 K)= -4211.592284
Lowest Frequency = 12.8246cm-1

C	-1.718516	-0.060169	-2.197695
H	-1.310425	-0.806174	-2.895583
H	-2.805393	-0.230548	-2.173857
C	-1.398543	1.336820	-2.741336
H	-1.999360	1.505772	-3.650920
H	-1.709002	2.113740	-2.025878
C	0.068337	1.544954	-3.099513
H	0.743926	1.238393	-2.253953
H	0.363713	0.839125	-3.893904
C	0.426917	2.967876	-3.490052
H	1.501996	3.065835	-3.699401
H	0.180630	3.675714	-2.683864
H	-0.125678	3.275773	-4.391800
C	-2.372300	-0.058052	0.677633
C	-3.566682	-1.031843	0.635295
H	-3.234334	-2.047222	0.896930
H	-3.991118	-1.081181	-0.381449
C	-4.651074	-0.592982	1.634602
H	-5.490423	-1.304654	1.580938
C	-4.062027	-0.592546	3.052378
H	-3.719406	-1.606162	3.320614
H	-4.837700	-0.312106	3.784437
C	-2.889017	0.395072	3.117017
H	-2.453206	0.390390	4.128327
C	-3.383113	1.805075	2.773329
H	-4.147593	2.131549	3.498041
H	-2.549284	2.523656	2.837011
C	-3.967320	1.799121	1.355910
H	-4.310595	2.811280	1.088331
C	-2.889602	1.365323	0.350964
H	-3.324150	1.393509	-0.659972
H	-2.048980	2.080875	0.367934
C	-5.144178	0.816675	1.283613

H -5.939870 1.125510 1.981713
H -5.583929 0.823985 0.271841
C -1.802588 -0.024054 2.115723
H -0.954652 0.674992 2.163957
H -1.404954 -1.007639 2.399894
C -0.601203 -2.249115 -0.635495
C -0.303203 -2.817419 0.769228
H 0.449784 -2.192487 1.276088
H -1.215248 -2.794766 1.384337
C 0.186291 -4.270573 0.668271
H 0.388292 -4.644675 1.684280
C 1.470294 -4.330132 -0.168936
H 2.260987 -3.728236 0.308253
H 1.840792 -5.367286 -0.224456
C 1.180441 -3.796484 -1.577594
H 2.100444 -3.822214 -2.182734
C 0.094967 -4.651048 -2.244241
H 0.437654 -5.694925 -2.338141
H -0.102948 -4.284591 -3.265839
C -1.186735 -4.588394 -1.403376
H -1.974112 -5.193907 -1.880266
C -1.674453 -3.131542 -1.312293
H -2.616225 -3.099511 -0.747825
H -1.897105 -2.763758 -2.326051
C 0.700188 -2.341930 -1.474808
H 0.533111 -1.937212 -2.487431
H 1.485476 -1.725337 -1.008024
C -0.902422 -5.126291 0.005950
H -1.823554 -5.104121 0.612652
H -0.579786 -6.179450 -0.048759
P -0.999392 -0.427772 -0.532375
Pd 0.975584 0.971134 -0.348002
C 4.298626 4.240898 -1.318826
C 4.560900 2.898339 -1.595197
C 3.658143 1.908453 -1.199340
C 2.229297 3.603474 -0.237597
C 3.134667 4.590927 -0.634686
H 5.478943 2.616946 -2.117884
H 3.880511 0.858650 -1.410446
H 1.327419 3.889211 0.312409
H 2.931435 5.638934 -0.399134
C 2.475246 2.261035 -0.542554
Br 1.970166 0.420465 1.799308
H 5.008471 5.012947 -1.625031

int-3-me.log

SCF (RwB97XD) = -4251.49679684
E(SCF)+ZPE(0 K)= -4250.780667
H(298 K)= -4250.746737
G(298 K)= -4250.846227
Lowest Frequency = 12.8764cm-1

C -1.883959 -0.240215 -2.154677
H -1.468022 -0.988581 -2.845344
H -2.966321 -0.434545 -2.114495
C -1.601531 1.154645 -2.724139
H -2.220363 1.298732 -3.625877
H -1.915798 1.935084 -2.014096
C -0.144192 1.385847 -3.107208
H 0.548791 1.084865 -2.274130
H 0.147514 0.685077 -3.907554
C 0.185825 2.814221 -3.502912
H 1.255695 2.928611 -3.729841
H -0.055883 3.517188 -2.691140
H -0.386780 3.114844 -4.394572
C -2.506485 -0.212768 0.726277
C -3.680683 -1.211114 0.702012
H -3.325332 -2.216250 0.973171
H -4.110463 -1.280803 -0.311265
C -4.767704 -0.783659 1.703627
H -5.593520 -1.511914 1.661884
C -4.169690 -0.758249 3.117299
H -3.806182 -1.762678 3.392454
H -4.945658 -0.485750 3.852035
C -3.015510 0.252489 3.164882
H -2.572988 0.265350 4.173218
C -3.539450 1.649313 2.811998
H -4.305059 1.967668 3.539173
H -2.719160 2.384321 2.863165
C -4.133258 1.618878 1.398893
H -4.498143 2.621659 1.124749
C -3.054600 1.196923 0.390115
H -3.497244 1.206456 -0.617624
H -2.228128 1.928905 0.393498
C -5.290718 0.612779 1.342953
H -6.088315 0.911831 2.043141
H -5.736508 0.602456 0.333827
C -1.927637 -0.154104 2.159909
H -1.093137 0.561715 2.195915
H -1.509467 -1.127156 2.450606
C -0.684824 -2.371917 -0.573865
C -0.351123 -2.908951 0.835108
H 0.386624 -2.252173 1.324016
H -1.256698 -2.906763 1.460162
C 0.183848 -4.346948 0.749643
H 0.411169 -4.698501 1.768387
C 1.457821 -4.378763 -0.104146
H 2.235037 -3.743999 0.352276
H 1.861126 -5.404140 -0.148626
C 1.132132 -3.877249 -1.516745
H 2.044393 -3.883777 -2.134001
C 0.065181 -4.775274 -2.155333
H 0.438898 -5.809314 -2.238915
H -0.157613 -4.430252 -3.179248
C -1.206370 -4.739955 -1.297581
H -1.980351 -5.377650 -1.754092
C -1.739073 -3.298028 -1.221413
H -2.672971 -3.287278 -0.643137
H -1.988633 -2.953293 -2.237050
C 0.607547 -2.437301 -1.429824
H 0.414260 -2.054370 -2.446234
H 1.379358 -1.789061 -0.983795
C -0.885681 -5.246979 0.115419
H -1.798660 -5.244959 0.734707
H -0.530358 -6.290057 0.071408
P -1.135976 -0.561913 -0.492468
Pd 0.799221 0.899144 -0.349952
C 4.013305 4.266440 -1.452804
C 4.294072 2.915210 -1.699168
C 3.434750 1.905967 -1.265048
C 1.980032 3.569144 -0.306844
C 2.846692 4.574097 -0.745822
H 5.208108 2.646560 -2.237981
H 3.689120 0.860903 -1.463805
H 1.083488 3.844593 0.256993
H 2.610504 5.619389 -0.524881
C 2.254144 2.229580 -0.588945
Br 1.822412 0.432000 1.802248
C 4.961758 5.343462 -1.912174
H 4.529823 6.345697 -1.778352
H 5.906636 5.310382 -1.345899
H 5.218502 5.226513 -2.976563

int-3-nme2.log

SCF (RwB97XD) = -4346.11053776
E(SCF)+ZPE(0 K)= -4345.348532
H(298 K)= -4345.312102
G(298 K)= -4345.417067
Lowest Frequency = 11.5058cm-1

C -2.136101 -0.502853 -2.083552
H -1.709281 -1.238649 -2.781014
H -3.212956 -0.723878 -2.034080
C -1.892562 0.900131 -2.651020
H -2.529919 1.035802 -3.541155
H -2.210699 1.670105 -1.931197
C -0.445931 1.162826 -3.054626
H 0.260660 0.871049 -2.231807

H	-0.153801	0.475213	-3.866248
C	-0.151322	2.601189	-3.442840
H	0.912429	2.739894	-3.684595
H	-0.393626	3.291514	-2.620358
H	-0.743789	2.899308	-4.322427
C	-2.735785	-0.505817	0.801022
C	-3.887641	-1.529639	0.776488
H	-3.508668	-2.528823	1.037705
H	-4.322746	-1.601025	-0.234404
C	-4.977410	-1.134267	1.788384
H	-5.787329	-1.880284	1.746017
C	-4.370673	-1.106698	3.198359
H	-3.983209	-2.104980	3.462980
H	-5.147471	-0.857345	3.940450
C	-3.238631	-0.071125	3.246665
H	-2.789841	-0.056698	4.252231
C	-3.795700	1.316495	2.908585
H	-4.563171	1.612168	3.643422
H	-2.991333	2.068874	2.960129
C	-4.398214	1.284060	1.499158
H	-4.786988	2.280684	1.235594
C	-3.317370	0.893983	0.479973
H	-3.766855	0.901430	-0.524754
H	-2.507233	1.643926	0.483434
C	-5.533543	0.253102	1.442436
H	-6.333234	0.528629	2.149919
H	-5.985329	0.241198	0.435979
C	-2.148914	-0.445641	2.231282
H	-1.329545	0.287553	2.267349
H	-1.708032	-1.411748	2.511430
C	-0.868039	-2.609280	-0.525363
C	-0.505948	-3.144566	0.877158
H	0.217974	-2.469972	1.362608
H	-1.405172	-3.170442	1.510819
C	0.067279	-4.566830	0.778585
H	0.314161	-4.917545	1.793111
C	1.333178	-4.559507	-0.087471
H	2.097116	-3.906050	0.364967
H	1.763575	-5.573417	-0.141184
C	0.980333	-4.059339	-1.494123
H	1.886580	-4.037971	-2.119908
C	-0.067971	-4.982724	-2.127598
H	0.332846	-6.005738	-2.221074
H	-0.310369	-4.638048	-3.147176
C	-1.331557	-4.986972	-1.257354
H	-2.092104	-5.643350	-1.710073
C	-1.902761	-3.560678	-1.167580
H	-2.830687	-3.579071	-0.579875
H	-2.172180	-3.217399	-2.178626
C	0.417325	-2.635016	-1.393880
H	0.204235	-2.251961	-2.406224
H	1.175158	-1.967990	-0.951534
C	-0.983232	-5.492651	0.149607
H	-1.889900	-5.519259	0.777646
H	-0.599665	-6.525272	0.096192
P	-1.364584	-0.811330	-0.429395
Pd	0.537856	0.694855	-0.283390
C	3.662836	4.139297	-1.455214
C	3.988579	2.780219	-1.654532
C	3.152705	1.763702	-1.195098
C	1.658105	3.394599	-0.276150
C	2.485582	4.421114	-0.730974
H	4.906116	2.501365	-2.172160
H	3.449578	0.724546	-1.364322
H	0.761768	3.660307	0.293094
H	2.205235	5.449798	-0.505483
C	1.957847	2.055939	-0.534828
Br	1.585237	0.247372	1.860946
N	4.470309	5.154160	-1.949149
C	4.223470	6.512905	-1.526160
H	4.919273	7.185682	-2.043094
H	3.204774	6.833366	-1.794256
H	4.351945	6.658618	-0.435939
C	5.801116	4.826412	-2.406637
H	5.772296	4.125608	-3.255557
H	6.298215	5.738594	-2.759453
H	6.430330	4.376985	-1.614338

int-3-ome.log

SCF (RwB97XD) = -4326.70917204
E(SCF)+ZPE(0 K)= -4325.987991
H(298 K)= -4325.953260
G(298 K)= -4326.054520
Lowest Frequency = 13.5367cm-1

C	-1.912455	-0.390597	-2.192092
H	-1.428431	-1.117261	-2.861371
H	-2.985761	-0.632773	-2.208388
C	-1.662786	1.017478	-2.743734
H	-2.243807	1.139339	-3.673556
H	-2.043414	1.780439	-2.047327
C	-0.200487	1.313305	-3.057036
H	0.464984	1.046870	-2.191042
H	0.159697	0.626377	-3.841375
C	0.081751	2.755299	-3.440754
H	1.153474	2.917762	-3.625595
H	-0.223354	3.446964	-2.640660
H	-0.467692	3.031239	-4.354716
C	-2.680267	-0.397278	0.655112
C	-3.810065	-1.441966	0.568896
H	-3.427812	-2.432954	0.854836
H	-4.185760	-1.524004	-0.464703
C	-4.962380	-1.064241	1.516059
H	-5.754565	-1.825418	1.430159
C	-4.437848	-1.021890	2.958161
H	-4.048170	-2.012215	3.248275
H	-5.260743	-0.784905	3.653117
C	-3.329445	0.034544	3.067191
H	-2.938804	0.060102	4.096617
C	-3.891480	1.410810	2.692541
H	-4.705260	1.694168	3.380893
H	-3.105484	2.178227	2.787143
C	-4.411598	1.363521	1.251223
H	-4.802447	2.352149	0.962197
C	-3.267504	0.990927	0.296622
H	-3.660023	0.988263	-0.731727
H	-2.473046	1.756067	0.343865
C	-5.523080	0.311689	1.133901
H	-6.366066	0.574232	1.794344
H	-5.917230	0.288815	0.103659
C	-2.177123	-0.322939	2.116333
H	-1.374962	0.425490	2.196102
H	-1.735353	-1.280001	2.424509
C	-0.709988	-2.476574	-0.554698
C	-0.425439	-3.003802	0.868478
H	0.259242	-2.318323	1.393832
H	-1.360158	-3.040359	1.447931
C	0.171997	-4.418390	0.808308
H	0.361967	-4.763505	1.836860
C	1.487531	-4.395262	0.019834
H	2.213932	-3.731039	0.516293
H	1.934069	-5.403252	-0.005837
C	1.213139	-3.902765	-1.406832
H	2.154921	-3.869298	-1.977288
C	0.217787	-4.842611	-2.099095
H	0.637624	-5.860215	-2.163635
H	0.032764	-4.505468	-3.133107
C	-1.095704	-4.861778	-1.306846
H	-1.818608	-5.529503	-1.802589
C	-1.690956	-3.443357	-1.256047
H	-2.652236	-3.474439	-0.725419
H	-1.903491	-3.105541	-2.282355
C	0.625473	-2.486164	-1.344211
H	0.467770	-2.106830	-2.368079
H	1.346257	-1.808728	-0.858179
C	-0.826296	-5.359581	0.120068
H	-1.768669	-5.397091	0.692566
H	-0.426404	-6.386971	0.093156
P	-1.237316	-0.686085	-0.494260

Pd	0.627652	0.849992	-0.251423
C	3.782354	4.315941	-1.154955
C	4.131126	2.987031	-1.413350
C	3.265802	1.955573	-1.028596
C	1.723555	3.564655	-0.126523
C	2.574317	4.598553	-0.503843
H	5.072017	2.734665	-1.903315
H	3.562700	0.921515	-1.226035
H	0.794286	3.808250	0.397210
H	2.323680	5.639830	-0.289488
C	2.047146	2.231652	-0.411863
Br	1.562181	0.404955	1.944180
O	4.548287	5.391323	-1.482551
C	5.793040	5.161561	-2.101147
H	5.679495	4.666088	-3.080731
H	6.250511	6.147409	-2.255591
H	6.461310	4.556335	-1.465150

int-4-cf3.log

SCF (RwB97XD) = -4549.30566435
E(SCF)+ZPE(0 K)= -4548.611084
H(298 K)= -4548.575414
G(298 K)= -4548.679289
Lowest Frequency = 14.1707cm-1

C	-1.596219	-0.813808	-2.315668
H	-1.314406	-1.699799	-2.902093
H	-2.688583	-0.873230	-2.197770
C	-1.203960	0.433744	-3.115406
H	-1.978748	0.593505	-3.884252
H	-1.225042	1.331754	-2.478367
C	0.152083	0.325914	-3.809620
H	0.948298	0.192214	-3.054900
H	0.167987	-0.589116	-4.427001
C	0.472687	1.530623	-4.685064
H	1.464812	1.433731	-5.150311
H	0.470617	2.467709	-4.104845
H	-0.267419	1.646553	-5.493138
C	-2.137294	-0.376148	0.539687
C	-3.438205	-1.206698	0.584153
H	-3.224173	-2.225943	0.938702
H	-3.880229	-1.298420	-0.421641
C	-4.452836	-0.551742	1.538635
H	-5.369244	-1.162781	1.547976
C	-3.857059	-0.490232	2.952045
H	-3.636546	-1.508052	3.315852
H	-4.587659	-0.050532	3.650928
C	-2.573672	0.350179	2.925694
H	-2.130606	0.386660	3.933398
C	-2.892155	1.772054	2.447622
H	-3.596627	2.260046	3.141129
H	-1.974355	2.382556	2.434274
C	-3.495581	1.706414	1.039859
H	-3.717182	2.724384	0.683042
C	-2.487116	1.062111	0.078269
H	-2.924701	1.042539	-0.931700
H	-1.575470	1.676035	0.028409
C	-4.780551	0.868429	1.060123
H	-5.525900	1.329330	1.729075
H	-5.229230	0.836099	0.053005
C	-1.559231	-0.297052	1.970908
H	-0.633841	0.290588	1.962528
H	-1.302169	-1.298453	2.340662
C	-0.641068	-2.892942	-0.522533
C	-0.382983	-3.332571	0.935439
H	0.421488	-2.722738	1.380412
H	-1.286837	-3.169901	1.540856
C	-0.007900	-4.821511	0.991078
H	0.167409	-5.099476	2.042049
C	1.264009	-5.070460	0.170779
H	2.105207	-4.489742	0.584038
H	1.548696	-6.133799	0.225554
C	1.013919	-4.668484	-1.288130
H	1.925991	-4.829324	-1.883000
C	-0.143088	-5.493477	-1.866979
H	0.116454	-6.564679	-1.854054
H	-0.316550	-5.217463	-2.920493
C	-1.410996	-5.245856	-1.038696
H	-2.244772	-5.834093	-1.453343
C	-1.784650	-3.754621	-1.102227
H	-2.714292	-3.584907	-0.541510
H	-1.988134	-3.483249	-2.148802
C	0.646372	-3.178321	-1.344107
H	0.525102	-2.862183	-2.392284
H	1.516684	-2.616626	-0.933722
C	-1.162527	-5.654932	0.419047
H	-2.076440	-5.499781	1.016595
H	-0.920030	-6.728747	0.475614
P	-0.861954	-1.037779	-0.643486
Pd	1.361278	-0.442946	-0.476787
C	0.711956	4.072276	0.898266
C	0.656742	3.742074	-0.456886
C	0.861901	2.425440	-0.862594
C	1.213332	1.769695	1.431607
C	1.005372	3.084984	1.840482
H	0.454131	4.512131	-1.204073
H	0.825999	2.188305	-1.925220
H	1.462637	1.016018	2.180176
H	1.079008	3.338771	2.899710
C	0.401931	5.474844	1.341008
C	1.104021	1.423772	0.080843
Br	3.804332	-0.271729	-0.450164
F	0.991381	5.791929	2.506824
F	0.788406	6.396596	0.440687
F	-0.927023	5.660037	1.524641

int-4-cl.log

SCF (RwB97XD) = -4671.85368325
E(SCF)+ZPE(0 K)= -4671.173698
H(298 K)= -4671.140350
G(298 K)= -4671.238275
Lowest Frequency = 16.3005cm-1

C	-1.607861	-0.537913	-2.236095
H	-1.322687	-1.421595	-2.824439
H	-2.699092	-0.605763	-2.111740
C	-1.229711	0.712353	-3.038320
H	-2.007343	0.863096	-3.806175
H	-1.259029	1.610902	-2.402430
C	0.126383	0.617047	-3.734337
H	0.924354	0.489375	-2.980496
H	0.149256	-0.297212	-4.352684
C	0.436381	1.825342	-4.608603
H	1.427892	1.735651	-5.076614
H	0.430184	2.761227	-4.026497
H	-0.306606	1.937928	-5.414557
C	-2.132352	-0.103678	0.623276
C	-3.424884	-0.946306	0.680717
H	-3.198375	-1.962875	1.035069
H	-3.874724	-1.043569	-0.321147
C	-4.437354	-0.299786	1.643024
H	-5.347804	-0.919514	1.661256
C	-3.829553	-0.230192	3.050890
H	-3.595727	-1.245299	3.414010
H	-4.558170	0.203300	3.755767
C	-2.554802	0.622740	3.011736
H	-2.103323	0.665544	4.015450
C	-2.891354	2.040604	2.534041
H	-3.594595	2.522823	3.232945
H	-1.979014	2.659092	2.511760
C	-3.506318	1.966325	1.131685
H	-3.741580	2.981234	0.774673
C	-2.499747	1.330332	0.162493
H	-2.945737	1.304934	-0.843696
H	-1.594084	1.952551	0.104390
C	-4.782927	1.116253	1.165001
H	-5.526685	1.571211	1.839828
H	-5.240100	1.077857	0.161905

C	-1.542082	-0.015286	2.049027
H	-0.623596	0.583091	2.030393
H	-1.270093	-1.012953	2.418340
C	-0.631069	-2.611520	-0.449787
C	-0.361345	-3.049765	1.006510
H	0.444146	-2.436963	1.445422
H	-1.261357	-2.889743	1.618375
C	0.019424	-4.537299	1.060283
H	0.202770	-4.814247	2.110168
C	1.286808	-4.781642	0.231618
H	2.128283	-4.197226	0.639028
H	1.576122	-5.843833	0.285038
C	1.025351	-4.381306	-1.225804
H	1.934143	-4.538843	-1.826557
C	-0.132010	-5.211446	-1.796393
H	0.131704	-6.281677	-1.784360
H	-0.313468	-4.936899	-2.848953
C	-1.395338	-4.967983	-0.959983
H	-2.229675	-5.559578	-1.368775
C	-1.774929	-3.478192	-1.021727
H	-2.701937	-3.312256	-0.455648
H	-1.985939	-3.208172	-2.067156
C	0.651665	-2.892605	-1.280095
H	0.521220	-2.577892	-2.327609
H	1.522043	-2.326490	-0.877032
C	-1.135832	-5.375311	0.496319
H	-2.046414	-5.223057	1.099693
H	-0.889138	-6.448243	0.551926
P	-0.860397	-0.756585	-0.568733
Pd	1.358101	-0.149016	-0.412758
C	0.724662	4.358995	0.980663
C	0.633118	4.041595	-0.371554
C	0.825283	2.720944	-0.779823
C	1.230250	2.063229	1.500887
C	1.038158	3.379733	1.920020
H	0.414911	4.818831	-1.106171
H	0.760366	2.487694	-1.842248
H	1.493857	1.309688	2.245152
H	1.138120	3.641001	2.975016
C	1.092163	1.717621	0.153270
Br	3.802594	0.022391	-0.393447
Cl	0.461265	6.001117	1.502223

int-4-cn.log

SCF (RwB97XD) = -4304.44714444
E(SCF)+ZPE(0 K)= -4303.758898
H(298 K)= -4303.724777
G(298 K)= -4303.824684
Lowest Frequency = 20.1184cm-1

C	0.266990	0.098654	-2.250932
H	0.555692	-0.721092	-2.928151
H	-0.820259	0.221726	-2.359501
C	0.935897	1.412115	-2.655129
H	0.768429	2.170670	-1.870095
H	2.027652	1.283781	-2.725237
C	0.416169	1.943815	-3.989745
H	0.565179	1.177246	-4.769764
H	-0.675138	2.095398	-3.918872
C	1.087786	3.244671	-4.409956
H	0.924871	4.037503	-3.662465
H	0.695339	3.607828	-5.371698
H	2.176197	3.114946	-4.522516
C	-0.535654	-2.002002	-0.395117
C	0.092901	-3.209060	-1.128152
H	1.086001	-3.442755	-0.720137
H	0.229432	-2.974363	-2.196737
C	-0.806346	-4.448884	-0.978945
H	-0.326073	-5.293025	-1.498703
C	-0.971750	-4.783992	0.510271
H	0.010492	-5.000565	0.963187
H	-1.584273	-5.693350	0.625878
C	-1.635636	-3.602451	1.231680
H	-1.750074	-3.837385	2.301580
C	-3.008669	-3.317321	0.612360
H	-3.671752	-4.190215	0.728971
H	-3.490113	-2.472989	1.132839
C	-2.829407	-2.985986	-0.873302
H	-3.807536	-2.759493	-1.325456
C	-1.930063	-1.750213	-1.029461
H	-1.825720	-1.535382	-2.102929
H	-2.413160	-0.879591	-0.565696
C	-2.182973	-4.173925	-1.596264
H	-2.821940	-5.068062	-1.510323
H	-2.081084	-3.953656	-2.672152
C	-0.747913	-2.357014	1.096068
H	-1.215932	-1.505395	1.611747
H	0.212766	-2.541284	1.594403
C	2.317703	-0.763748	-0.269809
C	2.586623	-1.683111	0.944375
H	2.094751	-1.273861	1.842649
H	2.168459	-2.684436	0.773144
C	4.097750	-1.818088	1.194967
H	4.248526	-2.491031	2.053350
C	4.701162	-0.442717	1.502025
H	4.242940	-0.018797	2.410830
H	5.781629	-0.535596	1.699219
C	4.459599	0.486968	0.307844
H	4.866435	1.487102	0.521606
C	5.126316	-0.092386	-0.947243
H	6.215068	-0.168053	-0.792963
H	4.970480	0.581405	-1.806227
C	4.536229	-1.476973	-1.247962
H	5.014556	-1.897581	-2.146538
C	3.027268	-1.343444	-1.511805
H	2.607886	-2.326751	-1.769443
H	2.870109	-0.690258	-2.383185
C	2.948127	0.617449	0.065262
H	2.764227	1.345289	-0.735861
H	2.497702	1.032388	0.992838
C	4.768203	-2.410274	-0.052120
H	4.355754	-3.410553	-0.266677
H	5.848506	-2.540394	0.124106
P	0.488156	-0.443861	-0.518918
Pd	0.158838	1.263860	0.967159
Br	0.240401	3.125509	2.561786
C	-1.762423	1.325246	0.600492
C	-2.670294	0.816580	1.537598
C	-2.241112	1.910531	-0.577510
C	-4.035858	0.841236	1.276357
H	-2.317055	0.392748	2.479053
C	-3.606131	1.941120	-0.843721
H	-1.550134	2.338898	-1.305952
C	-4.509867	1.391838	0.076470
H	-4.740651	0.433602	2.003601
H	-3.974875	2.388246	-1.768907
C	-5.918373	1.396476	-0.208321
N	-7.045773	1.392779	-0.441823

int-4-h.log

SCF (RwB97XD) = -4212.23798322
E(SCF)+ZPE(0 K)= -4211.548036
H(298 K)= -4211.515948
G(298 K)= -4211.610379
Lowest Frequency = 20.8718cm-1

C	-0.761947	0.920518	-2.198726
H	-1.670332	1.497101	-2.416670
H	0.071490	1.556463	-2.532448
C	-0.796572	-0.385389	-3.001530
H	-0.521871	-0.144794	-4.042809
H	-0.019951	-1.073690	-2.636981
C	-2.149007	-1.101426	-3.009500
H	-2.019937	-2.072976	-3.513881
H	-2.450704	-1.339945	-1.973476
C	-3.271392	-0.333921	-3.698982
H	-4.195645	-0.930270	-3.725005
H	-3.006760	-0.081030	-4.738699

H	-3.515616	0.607686	-3.181394
C	1.157201	1.027210	0.022353
C	1.440015	0.728107	1.512219
H	1.078339	-0.276832	1.761051
H	0.901796	1.434275	2.158493
C	2.946235	0.822703	1.802368
H	3.110981	0.610515	2.870621
C	3.697884	-0.205476	0.947949
H	3.350702	-1.223940	1.187737
H	4.777854	-0.168257	1.167796
C	3.448851	0.094829	-0.534763
H	3.974527	-0.645634	-1.157866
C	3.944438	1.506841	-0.873462
H	5.028696	1.585506	-0.689288
H	3.784350	1.718758	-1.944199
C	3.190339	2.529452	-0.013511
H	3.534948	3.547131	-0.256762
C	1.680408	2.443666	-0.297569
H	1.159889	3.186224	0.325763
H	1.485338	2.707530	-1.349800
C	3.444885	2.235881	1.471302
H	4.521091	2.323255	1.694661
H	2.925204	2.978872	2.099580
C	1.945594	0.001610	-0.832177
H	1.785321	0.193941	-1.904091
H	1.585840	-1.017253	-0.624674
C	-1.718063	2.207245	0.224515
C	-3.170994	1.680291	0.065177
H	-3.385088	1.430175	-0.985857
H	-3.327000	0.749532	0.655747
C	-4.188775	2.709774	0.576053
H	-5.201403	2.300277	0.440554
C	-4.037036	4.010095	-0.224103
H	-4.240178	3.825391	-1.292255
H	-4.776737	4.751488	0.119759
C	-2.615336	4.557502	-0.042202
H	-2.499783	5.489665	-0.617659
C	-2.357437	4.833192	1.445324
H	-3.068823	5.587969	1.819137
H	-1.345536	5.249379	1.584996
C	-2.505414	3.527126	2.237355
H	-2.307419	3.713594	3.304350
C	-1.479712	2.505714	1.720823
H	-1.540930	1.574981	2.309515
H	-0.468213	2.914371	1.863159
C	-1.595926	3.529975	-0.564623
H	-0.581667	3.941493	-0.469900
H	-1.774128	3.364688	-1.637771
C	-3.926443	2.976077	2.063659
H	-4.048360	2.044469	2.640785
H	-4.663655	3.696131	2.454627
P	-0.647522	0.787732	-0.366068
Pd	-1.851019	-0.940705	0.570977
Br	-3.522901	-2.397908	1.619411
C	-0.439181	-2.315426	0.535934
C	0.279695	-2.594223	1.702063
C	-0.191609	-3.061019	-0.618418
C	1.279591	-3.568884	1.691847
H	0.062144	-2.060592	2.629204
C	0.810174	-4.034825	-0.621966
H	-0.775428	-2.894213	-1.524113
C	1.558485	-4.283896	0.527345
H	1.838374	-3.772594	2.608911
H	0.999050	-4.605456	-1.534906
H	2.342675	-5.044284	0.521465

int-4-me.log

SCF (RwB97XD) = -4251.51780293

E(SCF)+ZPE(0 K)= -4250.800407

H(298 K)= -4250.766489

G(298 K)= -4250.866175

Lowest Frequency = 7.2936cm-1

C	-1.552125	-0.077077	-2.061957
H	-1.216750	-0.793289	-2.825550
H	-2.612858	-0.309713	-1.884282
C	-1.394902	1.336912	-2.632795
H	-2.248722	1.522936	-3.306302
H	-1.472536	2.091891	-1.835131
C	-0.102283	1.547530	-3.418122
H	0.766126	1.386802	-2.753959
H	-0.028462	0.773995	-4.202451
C	-0.008652	2.925949	-4.059676
H	0.941023	3.050771	-4.600811
H	-0.066586	3.729287	-3.307601
H	-0.828645	3.090508	-4.777427
C	-1.870403	-0.245810	0.857042
C	-3.044690	-1.247700	0.836353
H	-2.669443	-2.272316	0.977243
H	-3.566561	-1.222676	-0.134200
C	-4.039507	-0.925112	1.965831
H	-4.866338	-1.651955	1.923230
C	-3.324347	-1.032892	3.319816
H	-2.944478	-2.057940	3.467899
H	-4.034013	-0.831813	4.139391
C	-2.165356	-0.028424	3.362988
H	-1.636449	-0.110056	4.325780
C	-2.706874	1.395814	3.189227
H	-3.400549	1.643452	4.009962
H	-1.879240	2.122929	3.229752
C	-3.427342	1.498258	1.839865
H	-3.809555	2.521622	1.699094
C	-2.442010	1.187059	0.704535
H	-2.969911	1.286419	-0.256438
H	-1.623534	1.922267	0.707330
C	-4.588237	0.496804	1.789724
H	-5.320056	0.720448	2.583509
H	-5.121294	0.582614	0.827838
C	-1.172294	-0.340502	2.233015
H	-0.336984	0.368530	2.270504
H	-0.752632	-1.343512	2.387128
C	-0.189171	-2.293973	-0.774562
C	0.260701	-2.951422	0.548265
H	1.027403	-2.330258	1.041376
H	-0.592552	-3.023418	1.238878
C	0.809840	-4.363379	0.290127
H	1.121692	-4.799188	1.252269
C	2.013049	-4.291851	-0.658627
H	2.818351	-3.686925	-0.209976
H	2.424085	-5.300628	-0.827238
C	1.571619	-3.672738	-1.990557
H	2.430968	-3.602863	-2.674851
C	0.465186	-4.526179	-2.623882
H	0.845630	-5.539262	-2.833808
H	0.156169	-4.092629	-3.589779
C	-0.732988	-4.597989	-1.668003
H	-1.532413	-5.206091	-2.120225
C	-1.278396	-3.180966	-1.418005
H	-2.160849	-3.238968	-0.766067
H	-1.615657	-2.754591	-2.374667
C	1.034214	-2.257664	-1.731428
H	0.773796	-1.774807	-2.686489
H	1.868916	-1.669501	-1.289246
C	-0.293151	-5.225644	-0.338574
H	-1.153048	-5.302208	0.348012
H	0.074934	-6.251113	-0.507424
P	-0.638102	-0.492553	-0.518191
Pd	1.495033	0.369337	-0.380824
Br	3.904589	0.821255	-0.471640
C	1.048977	2.052049	0.544187
C	0.581822	3.160210	-0.159375
C	1.260593	2.164439	1.921489
C	0.272299	4.344555	0.515297
H	0.449437	3.116247	-1.240189
C	0.946142	3.349915	2.584489
H	1.676168	1.330366	2.490372
C	0.433167	4.458618	1.898606
H	-0.102720	5.198095	-0.057219
H	1.110762	3.413550	3.664434

C	0.098180	5.730613	2.632768
H	-0.412885	6.451635	1.978784
H	-0.556421	5.534502	3.496272
H	1.007575	6.218346	3.019498

int-4-nme2.log

SCF (RwB97XD) = -4346.13159045
E(SCF)+ZPE(0 K)= -4345.367992
H(298 K)= -4345.331720
G(298 K)= -4345.435243
Lowest Frequency = 18.0802cm-1

C	-1.430934	0.443335	-1.879427
H	-2.383177	0.697427	-1.389872
H	-1.452227	0.955908	-2.851590
C	-1.340511	-1.064206	-2.139272
H	-1.075499	-1.605805	-1.217883
H	-2.349032	-1.416135	-2.416159
C	-0.371644	-1.443709	-3.257031
H	-0.634769	-0.878483	-4.168191
H	0.650783	-1.122309	-2.988551
C	-0.376261	-2.934457	-3.570359
H	-1.369610	-3.268407	-3.911655
H	-0.116001	-3.536744	-2.684793
H	0.349619	-3.179319	-4.359939
C	0.060277	2.926444	-1.702447
C	0.863348	3.901882	-0.813789
H	1.807675	3.433393	-0.489321
H	0.288693	4.137707	0.094163
C	1.154046	5.208060	-1.569806
H	1.727380	5.875651	-0.907735
C	1.968269	4.909339	-2.834618
H	2.931419	4.444501	-2.566702
H	2.199282	5.845783	-3.368094
C	1.165994	3.970493	-3.744362
H	1.747594	3.738115	-4.649632
C	-0.166484	4.625971	-4.129504
H	0.020384	5.556702	-4.689978
H	-0.739299	3.959021	-4.795415
C	-0.973127	4.925930	-2.858998
H	-1.932648	5.393510	-3.131078
C	-1.262874	3.614006	-2.107937
H	-1.870519	3.830086	-1.218197
H	-1.864795	2.958144	-2.754426
C	-0.173870	5.873533	-1.954686
H	0.014700	6.825967	-2.476981
H	-0.754864	6.112975	-1.048249
C	0.885191	2.661781	-2.992664
H	0.363525	1.953812	-3.655733
H	1.872162	2.208707	-2.750520
C	-0.745260	1.320892	0.841215
C	0.369656	1.768131	1.814071
H	1.237019	1.105995	1.708162
H	0.706701	2.785236	1.574214
C	-0.134779	1.732710	3.264331
H	0.682148	2.060234	3.926769
C	-0.552570	0.302147	3.625971
H	0.308508	-0.379073	3.526823
H	-0.888905	0.255905	4.675306
C	-1.680206	-0.144122	2.688176
H	-1.980724	-1.175510	2.931636
C	-2.883638	0.796973	2.831230
H	-3.269961	0.770083	3.863656
H	-3.703855	0.465675	2.172107
C	-2.456821	2.225075	2.467441
H	-3.317470	2.906644	2.559085
C	-1.952133	2.267561	1.013801
H	-1.659971	3.299576	0.769421
H	-2.770752	1.990363	0.328979
C	-1.184189	-0.112751	1.235390
H	-1.998952	-0.455330	0.579008
H	-0.344703	-0.812730	1.110682
C	-1.333903	2.678599	3.411140
H	-1.031863	3.712704	3.173330
H	-1.693942	2.681279	4.453412
P	-0.089266	1.238041	-0.899908
Pd	2.046589	0.490821	-1.319640
Br	4.347358	0.131743	-2.100462
C	2.094000	-0.936473	0.044047
C	2.757751	-0.734802	1.254296
C	1.520909	-2.184085	-0.189477
C	2.794640	-1.729993	2.229933
H	3.263567	0.210857	1.460217
C	1.552438	-3.187645	0.778778
H	1.027445	-2.398162	-1.137271
C	2.189414	-2.986504	2.019729
H	3.320066	-1.515806	3.160251
H	1.076182	-4.139488	0.545175
N	2.231984	-3.981034	2.985631
C	1.401123	-5.150282	2.820110
H	0.319627	-4.911551	2.789263
H	1.655047	-5.694505	1.897297
H	1.574193	-5.839363	3.656108
C	2.643400	-3.630917	4.325070
H	3.679233	-3.257738	4.341906
H	1.995106	-2.860290	4.786591
H	2.613123	-4.525075	4.959981

int-4-ome.log

SCF (RwB97XD) = -4326.73003728
E(SCF)+ZPE(0 K)= -4326.007467
H(298 K)= -4325.972897
G(298 K)= -4326.072687
Lowest Frequency = 17.6432cm-1

C	-1.758887	-0.498494	-2.186564
H	-1.528738	-1.408297	-2.759292
H	-2.845553	-0.524237	-2.015087
C	-1.373759	0.713869	-3.041563
H	-2.174003	0.864912	-3.785891
H	-1.352971	1.631773	-2.433585
C	-0.047205	0.555664	-3.781334
H	0.771761	0.419989	-3.051797
H	-0.075862	-0.373577	-4.376999
C	0.270651	1.731857	-4.695498
H	1.242790	1.600216	-5.193439
H	0.313012	2.680624	-4.136423
H	-0.495168	1.848865	-5.479258
C	-2.151070	0.029961	0.677494
C	-3.472205	-0.758081	0.805482
H	-3.272087	-1.773343	1.178725
H	-3.964134	-0.863157	-0.175498
C	-4.421539	-0.047986	1.787752
H	-5.354635	-0.629676	1.856001
C	-3.757959	0.031865	3.169839
H	-3.551601	-0.982540	3.551137
H	-4.440538	0.512917	3.889959
C	-2.452223	0.830159	3.060716
H	-1.961489	0.878462	4.045734
C	-2.750273	2.248738	2.560121
H	-3.405704	2.775856	3.273329
H	-1.814537	2.827089	2.485193
C	-3.422330	2.165599	1.184628
H	-3.630286	3.180399	0.810579
C	-2.480130	1.466163	0.195030
H	-2.966581	1.433611	-0.791981
H	-1.553962	2.049376	0.085636
C	-4.729202	1.369026	1.287098
H	-5.429151	1.869473	1.976610
H	-5.224137	1.325245	0.302307
C	-1.504011	0.127463	2.077605
H	-0.562741	0.685001	2.008418
H	-1.260709	-0.871813	2.462372
C	-0.777473	-2.553456	-0.388265
C	-0.446728	-2.963344	1.063424
H	0.399958	-2.367588	1.444273
H	-1.308178	-2.756815	1.715381
C	-0.114818	-4.461695	1.138990

H	0.115892	-4.717304	2.185035
C	1.096447	-4.773599	0.251470
H	1.977562	-4.207518	0.596135
H	1.352729	-5.843393	0.321328
C	0.769312	-4.404210	-1.201010
H	1.637712	-4.611271	-1.844888
C	-0.445762	-5.206747	-1.684334
H	-0.219932	-6.285312	-1.654984
H	-0.674884	-4.954587	-2.733255
C	-1.651976	-4.893757	-0.789113
H	-2.528348	-5.464543	-1.134775
C	-1.980581	-3.392739	-0.873110
H	-2.869300	-3.178583	-0.263611
H	-2.238429	-3.142499	-1.912917
C	0.447208	-2.904723	-1.277422
H	0.270109	-2.612469	-2.324495
H	1.356244	-2.359070	-0.939809
C	-1.327432	-5.272110	0.662014
H	-2.197445	-5.071630	1.309612
H	-1.115096	-6.351500	0.733766
P	-0.946960	-0.694218	-0.545609
Pd	1.292051	-0.146476	-0.482760
C	0.778945	4.434814	0.804558
C	0.645675	4.057200	-0.537122
C	0.805808	2.727787	-0.913918
C	1.270683	2.131303	1.364486
C	1.110219	3.466041	1.755054
H	0.417604	4.823189	-1.281344
H	0.705735	2.465278	-1.966877
H	1.547408	1.397810	2.124379
H	1.256628	3.726737	2.803489
C	1.085488	1.744712	0.039725
Br	3.742338	-0.060446	-0.539083
O	0.581364	5.751524	1.079784
C	0.702143	6.177131	2.417689
H	0.494546	7.255027	2.420830
H	-0.027678	5.673056	3.074692
H	1.719487	6.011184	2.810942

int-5-cf3.log

SCF (RwB97XD) = -4662.60813615
E(SCF)+ZPE(0 K)= -4661.904265
H(298 K)= -4661.866251
G(298 K)= -4661.975523
Lowest Frequency = 14.6651cm-1

C	2.338692	-0.172532	1.992129
H	2.094196	-1.241978	2.044195
H	3.436947	-0.088330	2.010785
C	1.715581	0.500409	3.215718
H	2.069645	1.539134	3.313759
H	0.621504	0.548688	3.092310
C	2.036046	-0.252730	4.505458
H	1.658867	-1.285469	4.417418
H	3.131605	-0.331999	4.618525
C	1.436632	0.406002	5.740916
H	1.680420	-0.156490	6.654912
H	0.338638	0.459721	5.667418
H	1.812354	1.433822	5.872660
C	2.159347	2.160158	0.190441
C	1.021333	2.962932	0.876664
H	0.908895	2.656405	1.925885
H	0.059774	2.746658	0.384954
C	1.296371	4.472235	0.805075
H	0.475035	5.002659	1.312005
C	2.626664	4.788612	1.500733
H	2.577797	4.497496	2.563362
H	2.820601	5.873559	1.473623
C	3.760296	4.031295	0.797363
H	4.717752	4.247396	1.297383
C	3.833678	4.461037	-0.672828
H	4.049538	5.539855	-0.743345
H	4.657943	3.933874	-1.182064
C	2.499423	4.141791	-1.359537
H	2.548699	4.431746	-2.421006
C	2.238962	2.628289	-1.280647
H	1.306598	2.391254	-1.814142
H	3.049643	2.106358	-1.807437
C	1.365172	4.904310	-0.664686
H	1.538913	5.990551	-0.735968
H	0.404916	4.699002	-1.167025
C	3.496387	2.519182	0.883074
H	4.329297	1.973783	0.416953
H	3.474135	2.224561	1.941308
C	2.709476	-0.716032	-0.852240
C	4.209305	-0.358437	-0.941720
H	4.344118	0.679260	-1.278141
H	4.677709	-0.439818	0.052600
C	4.925135	-1.295399	-1.929652
H	5.988541	-1.011049	-1.975661
C	4.290088	-1.148635	-3.319304
H	4.388331	-0.108520	-3.673707
H	4.818687	-1.786239	-4.046935
C	2.809145	-1.545039	-3.249511
H	2.347511	-1.433997	-4.243514
C	2.681257	-2.997386	-2.776058
H	3.182114	-3.676691	-3.485626
H	1.619345	-3.292346	-2.740914
C	3.311131	-3.126601	-1.384554
H	3.206972	-4.161467	-1.022807
C	2.598042	-2.193755	-0.393207
H	3.060407	-2.323172	0.596025
H	1.539357	-2.480852	-0.290604
C	2.073894	-0.626863	-2.262535
H	1.015781	-0.931317	-2.205577
H	2.089685	0.404769	-2.639706
C	4.796311	-2.748055	-1.455015
H	5.266178	-2.865963	-0.464226
H	5.329566	-3.422092	-2.145663
P	1.727230	0.338777	0.345508
Pd	-0.631893	-0.134083	0.018211
Br	-0.693941	-1.883323	1.756133
C	-2.630578	-0.539688	-0.264172
C	-3.597525	0.187544	0.438954
C	-3.045712	-1.558339	-1.126824
C	-4.950023	-0.118766	0.309751
H	-3.300641	0.993900	1.114351
C	-4.397780	-1.869744	-1.263181
H	-2.311299	-2.139194	-1.690349
C	-5.351134	-1.153759	-0.538288
H	-5.693753	0.446811	0.875549
H	-4.705830	-2.677199	-1.930082
C	-6.816844	-1.451896	-0.696030
F	-7.485143	-1.359164	0.469551
F	-7.421827	-0.590021	-1.545454
F	-7.045394	-2.684876	-1.180599
C	-0.948667	1.042613	-1.427167
O	-1.227415	1.689479	-2.315443

int-5-cl.log

SCF (RwB97XD) = -4785.15660410
E(SCF)+ZPE(0 K)= -4784.467589
H(298 K)= -4784.431838
G(298 K)= -4784.535465
Lowest Frequency = 16.0207cm-1

C	1.478565	-0.219394	-2.244056
H	0.799312	0.507985	-2.708610
H	2.505724	0.073154	-2.514844
C	1.133286	-1.595864	-2.812314
H	1.870970	-2.348494	-2.491928
H	0.155752	-1.919059	-2.419032
C	1.076377	-1.585230	-4.338865
H	0.325193	-0.845589	-4.663544
H	2.045682	-1.236536	-4.737116
C	0.738446	-2.948808	-4.927744
H	0.707495	-2.916260	-6.027449
H	-0.245738	-3.299030	-4.577561

H	1.483719	-3.707680	-4.638292
C	2.416650	-1.166262	0.388386
C	3.737136	-1.365477	-0.394638
H	4.287694	-0.416878	-0.469762
H	3.529836	-1.696345	-1.421846
C	4.620205	-2.418235	0.295974
H	5.549893	-2.530643	-0.284209
C	4.952497	-1.960714	1.721768
H	5.504325	-1.006094	1.695380
H	5.607811	-2.697130	2.215465
C	3.650542	-1.795113	2.515513
H	3.877964	-1.451237	3.536900
C	2.906307	-3.134272	2.573435
H	3.525510	-3.890345	3.083677
H	1.978739	-3.028582	3.160672
C	2.577745	-3.588558	1.146094
H	2.030148	-4.543684	1.175110
C	1.686946	-2.534178	0.473106
H	1.394742	-2.880672	-0.527679
H	0.754107	-2.426946	1.048715
C	3.874613	-3.757962	0.344300
H	4.509416	-4.530421	0.808908
H	3.646413	-4.101760	-0.678480
C	2.771394	-0.736814	1.829914
H	1.857934	-0.582310	2.422867
H	3.313588	0.218729	1.823316
C	1.757916	1.819602	-0.177584
C	1.315903	2.280566	1.233913
H	0.228849	2.133193	1.342230
H	1.799871	1.680018	2.016149
C	1.646556	3.764064	1.451966
H	1.342404	4.045512	2.472740
C	0.876884	4.606701	0.428571
H	-0.208606	4.463607	0.559207
H	1.082449	5.678822	0.583418
C	1.298977	4.185644	-0.983470
H	0.737538	4.768894	-1.730002
C	2.804070	4.423320	-1.163402
H	3.040369	5.492081	-1.030390
H	3.110880	4.150742	-2.187009
C	3.574569	3.580920	-0.138918
H	4.657366	3.738729	-0.265627
C	3.268675	2.088251	-0.351752
H	3.854822	1.502016	0.370229
H	3.595885	1.781432	-1.358511
C	0.994812	2.696115	-1.205548
H	1.295122	2.433516	-2.230213
H	-0.088660	2.509680	-1.133342
C	3.155448	3.987349	1.280713
H	3.713716	3.395576	2.025933
H	3.404482	5.046194	1.460988
P	1.242852	0.037326	-0.447787
Pd	-1.063128	-0.201764	0.274023
Br	-1.980304	0.201262	-1.980933
C	-3.016821	-0.384579	0.903240
C	-3.628002	-1.640818	0.952582
C	-3.765886	0.741329	1.257157
C	-4.968082	-1.773603	1.319373
H	-3.067194	-2.541524	0.688965
C	-5.106126	0.625160	1.627574
H	-3.314807	1.736637	1.231846
C	-5.697488	-0.635437	1.652751
H	-5.442239	-2.756470	1.343915
H	-5.688298	1.510074	1.891283
Cl	-7.373145	-0.791270	2.111117
C	-0.757974	-0.439465	2.123614
O	-0.675255	-0.553105	3.248884

int-5-cn.log

SCF (RwB97XD) = -4417.74597264
E(SCF)+ZPE(0 K)= -4417.048523
H(298 K)= -4417.012093
G(298 K)= -4417.117255
Lowest Frequency = 15.1418cm-1

C	1.045370	-0.009001	2.611581
H	0.367184	-0.810257	2.938079
H	2.047652	-0.305194	2.957607
C	0.610696	1.289662	3.293109
H	1.362025	2.077698	3.130443
H	-0.320926	1.668247	2.836276
C	0.400730	1.113791	4.796584
H	-0.365984	0.338706	4.968724
H	1.330808	0.726407	5.247484
C	-0.010901	2.403747	5.494302
H	-0.952636	2.798807	5.080383
H	0.756461	3.185857	5.377364
H	-0.163079	2.246077	6.572549
C	2.295303	1.176291	0.224740
C	1.613674	2.570483	0.195636
H	1.209707	2.824890	1.186322
H	0.765760	2.548831	-0.503869
C	2.612300	3.652017	-0.241979
H	2.096479	4.625133	-0.239942
C	3.797921	3.689349	0.730927
H	3.449000	3.936724	1.747953
H	4.507970	4.479314	0.435352
C	4.497214	2.323680	0.731004
H	5.344676	2.338935	1.434851
C	5.001692	2.002917	-0.681360
H	5.734027	2.761535	-1.003894
H	5.522784	1.030641	-0.686412
C	3.810309	1.968614	-1.646539
H	4.159729	1.722956	-2.661789
C	2.823509	0.878704	-1.198359
H	1.982303	0.819056	-1.903520
H	3.343815	-0.089704	-1.216911
C	3.112610	3.334006	-1.656255
H	3.811969	4.115244	-1.997916
H	2.267121	3.322907	-2.362846
C	3.504391	1.241268	1.189004
H	4.016026	0.268636	1.234454
H	3.176567	1.479923	2.210976
C	1.458101	-1.826329	0.372632
C	0.518988	-2.770404	1.169184
H	0.658271	-2.636992	2.251822
H	-0.534663	-2.535566	0.940766
C	0.806632	-4.241269	0.828206
H	0.123874	-4.874678	1.416544
C	2.260755	-4.568520	1.193321
H	2.423341	-4.423922	2.274663
H	2.477101	-5.627091	0.974301
C	3.201112	-3.660407	0.391209
H	4.247887	-3.884875	0.651413
C	2.979494	-3.888880	-1.110641
H	3.216405	-4.933316	-1.373013
H	3.659466	-3.247926	-1.696526
C	1.520835	-3.571709	-1.466210
H	1.359410	-3.723247	-2.544797
C	1.214203	-2.105156	-1.130871
H	0.168249	-1.876088	-1.386591
H	1.831655	-1.450556	-1.758246
C	2.915977	-2.187646	0.730800
H	3.616563	-1.551687	0.171793
H	3.103076	-2.011619	1.802742
C	0.583050	-4.484130	-0.668328
H	-0.466231	-4.274960	-0.935415
H	0.774608	-5.541878	-0.913701
P	0.992259	-0.056364	0.772991
Pd	-1.291713	0.331847	0.031706
Br	-0.771775	0.946794	-2.303049
C	-3.260168	0.602190	-0.501554
C	-3.888817	1.832832	-0.279208
C	-3.985133	-0.432862	-1.103514
C	-5.207990	2.039748	-0.671958
H	-3.345961	2.654307	0.194910
C	-5.304236	-0.239994	-1.501484
H	-3.515653	-1.402530	-1.285277
C	-5.921085	1.000767	-1.286280

H -5.689867 3.004948 -0.504051
H -5.860582 -1.047890 -1.980721
C -7.285860 1.204327 -1.689689
N -8.380309 1.365757 -2.008778
C -2.067307 -0.157772 1.684421
O -2.598536 -0.462902 2.637386

int-5-h.log

SCF (RwB97XD) = -4325.54110789
E(SCF)+ZPE(0 K)= -4324.842783
H(298 K)= -4324.808153
G(298 K)= -4324.908695
Lowest Frequency = 21.0407cm-1

C -0.979566 -0.275193 2.205312
H -0.155584 0.282105 2.670497
H -1.919150 0.156045 2.586058
C -0.837711 -1.738100 2.627391
H -1.733208 -2.314851 2.345505
H 0.014372 -2.193709 2.097317
C -0.615687 -1.876088 4.132509
H 0.308631 -1.339027 4.404336
H -1.437267 -1.370698 4.670159
C -0.517552 -3.326060 4.588261
H -0.344980 -3.396241 5.672962
H 0.315063 -3.843730 4.085562
H -1.441054 -3.883882 4.361674
C -2.238970 -0.790603 -0.413405
C -3.521337 -0.836106 0.452041
H -3.891646 0.180553 0.646099
H -3.309593 -1.290300 1.430010
C -4.617097 -1.656235 -0.248999
H -5.512916 -1.663213 0.391839
C -4.954163 -1.019053 -1.603005
H -5.328431 0.008290 -1.457001
H -5.758260 -1.585846 -2.100681
C -3.696320 -1.002659 -2.481124
H -3.924327 -0.531368 -3.450180
C -3.204385 -2.437471 -2.705087
H -3.978782 -3.027402 -3.222748
H -2.312898 -2.437551 -3.354452
C -2.869541 -3.070413 -1.349212
H -2.500652 -4.097561 -1.497486
C -1.767318 -2.248290 -0.665515
H -1.479125 -2.730427 0.278798
H -0.867431 -2.246796 -1.300839
C -4.122669 -3.092294 -0.464098
H -4.911760 -3.699082 -0.938262
H -3.894397 -3.564771 0.506004
C -2.603982 -0.175679 -1.783504
H -1.716375 -0.121884 -2.431101
H -2.971123 0.852459 -1.658231
C -1.032730 1.972535 0.348133
C -0.619093 2.490636 -1.052278
H 0.416069 2.177717 -1.267919
H -1.253704 2.057782 -1.837369
C -0.705817 4.022966 -1.113656
H -0.431659 4.349268 -2.129524
C 0.268608 4.622628 -0.093587
H 1.300653 4.315062 -0.330351
H 0.240927 5.724049 -0.138209
C -0.117483 4.140440 1.309084
H 0.588195 4.546749 2.050474
C -1.542279 4.604209 1.638987
H -1.600228 5.705123 1.617542
H -1.817752 4.287165 2.658740
C -2.517481 4.005008 0.617976
H -3.545284 4.324812 0.852668
C -2.457859 2.468859 0.673994
H -3.184597 2.062487 -0.043691
H -2.761859 2.120456 1.674493
C -0.059016 2.606579 1.376219
H -0.325438 2.297141 2.397045
H 0.968520 2.253582 1.194406
C -2.137432 4.474492 -0.792897
H -2.842022 4.059787 -1.533716
H -2.211703 5.572468 -0.859739
P -0.819187 0.110697 0.423715
Pd 1.366483 -0.427845 -0.491032
Br 2.481811 -0.398260 1.713139
C 3.224454 -0.825870 -1.291006
C 3.654066 -2.144984 -1.462758
C 4.080689 0.220123 -1.648303
C 4.932110 -2.413822 -1.957924
H 2.998600 -2.978217 -1.194801
C 5.357978 -0.049431 -2.144493
H 3.763232 1.259481 -1.526013
C 5.787432 -1.367073 -2.300358
H 5.259370 -3.450154 -2.076141
H 6.021181 0.778165 -2.409651
H 6.786988 -1.577928 -2.687909
C 0.909312 -0.470705 -2.323229
O 0.727607 -0.490973 -3.442736

int-5-me.log

SCF (RwB97XD) = -4364.82017236
E(SCF)+ZPE(0 K)= -4364.094367
H(298 K)= -4364.057939
G(298 K)= -4364.162366
Lowest Frequency = 19.8095cm-1

C -1.144493 0.224244 2.289649
H -0.883118 1.290752 2.309674
H -2.208735 0.144707 2.562677
C -0.259774 -0.482153 3.316944
H -0.578476 -1.527399 3.456315
H 0.777887 -0.510729 2.946942
C -0.284040 0.222537 4.672027
H 0.059609 1.262229 4.538930
H -1.326020 0.284319 5.032609
C 0.581605 -0.471744 5.715341
H 0.249091 -1.508298 5.889190
H 0.548317 0.054813 6.681251
H 1.634553 -0.509851 5.393343
C -2.162678 0.846050 -0.370258
C -3.645123 0.534785 -0.070702
H -3.837705 0.604926 1.012238
H -3.895610 -0.491276 -0.376962
C -4.561688 1.512001 -0.827148
H -5.609544 1.263533 -0.594039
C -4.261795 2.952970 -0.394701
H -4.449729 3.072709 0.685379
H -4.934684 3.652763 -0.917401
C -2.798326 3.283124 -0.715099
H -2.566105 4.308914 -0.388447
C -2.559898 3.153918 -2.223800
H -3.205595 3.859143 -2.773029
H -1.516309 3.413722 -2.467444
C -2.857042 1.713201 -2.653813
H -2.675069 1.600031 -3.734315
C -1.920796 0.758666 -1.898136
H -2.072590 -0.264475 -2.265643
H -0.874871 1.027854 -2.118634
C -4.318028 1.366202 -2.335792
H -4.995321 2.031651 -2.896336
H -4.542883 0.335071 -2.657641
C -1.884619 2.313649 0.050317
H -0.826549 2.564639 -0.125825
H -2.066350 2.445963 1.126558
C -1.419548 -2.062717 0.446618
C -2.557336 -2.435849 1.427566
H -2.281217 -2.170123 2.457446
H -3.472040 -1.874937 1.187488
C -2.848808 -3.944655 1.367474
H -3.662341 -4.172366 2.074493
C -1.587784 -4.724896 1.761309
H -1.284655 -4.462505 2.788815
H -1.792862 -5.808174 1.753559

C	-0.458108	-4.394315	0.777341
H	0.454929	-4.941652	1.059641
C	-0.878954	-4.785615	-0.644449
H	-1.076221	-5.868882	-0.700674
H	-0.063323	-4.570100	-1.354922
C	-2.137765	-3.999514	-1.029744
H	-2.439910	-4.259636	-2.056602
C	-1.850895	-2.490175	-0.974429
H	-2.758889	-1.950296	-1.276822
H	-1.069065	-2.239140	-1.706938
C	-0.159222	-2.888679	0.820892
H	0.659771	-2.661034	0.120727
H	0.202710	-2.612620	1.820959
C	-3.273565	-4.333647	-0.054131
H	-4.190696	-3.790511	-0.337937
H	-3.510432	-5.409442	-0.101011
P	-0.937551	-0.248876	0.533861
Pd	1.278701	0.226272	-0.343371
Br	1.794979	1.894390	1.405841
C	3.133050	0.653618	-1.136784
C	3.295458	1.709735	-2.038272
C	4.256669	-0.085703	-0.763497
C	4.559948	2.035500	-2.527799
H	2.433173	2.302999	-2.355153
C	5.520837	0.245046	-1.257979
H	4.162021	-0.923449	-0.066872
C	5.696273	1.311128	-2.145488
H	4.665403	2.874070	-3.223071
H	6.388769	-0.342325	-0.943551
C	7.061636	1.694461	-2.654464
H	7.799950	0.901168	-2.468006
H	7.047265	1.896928	-3.736404
H	7.425286	2.608783	-2.157299
C	1.235463	-0.891175	-1.863441
O	1.277231	-1.515446	-2.809906

int-5-nme2.log

SCF (RwB97XD) = -4459.43373951
E(SCF)+ZPE(0 K)= -4458.662085
H(298 K)= -4458.623274
G(298 K)= -4458.732912
Lowest Frequency = 15.6519cm-1

C	2.104512	-0.612518	1.881581
H	1.766623	-1.656195	1.829128
H	3.206193	-0.624610	1.873747
C	1.570113	-0.027714	3.189046
H	2.017790	0.958937	3.388779
H	0.482370	0.127053	3.103559
C	1.846672	-0.945156	4.378661
H	1.370655	-1.922028	4.189891
H	2.932020	-1.137163	4.447699
C	1.338558	-0.376368	5.697020
H	0.249635	-0.211641	5.663278
H	1.813764	0.590901	5.928955
H	1.545390	-1.057486	6.536502
C	2.079093	1.907775	0.347331
C	3.463496	2.086057	1.015788
H	4.233940	1.532459	0.460569
H	3.453223	1.680178	2.037175
C	3.845567	3.574494	1.077926
H	4.835558	3.663088	1.553133
C	3.899495	4.154901	-0.341020
H	4.660003	3.624818	-0.938934
H	4.198558	5.215629	-0.307877
C	2.519996	4.015357	-0.997374
H	2.552443	4.415444	-2.023133
C	1.477066	4.781752	-0.175312
H	1.734368	5.853345	-0.139581
H	0.485552	4.703819	-0.651970
C	1.427450	4.198343	1.241718
H	0.670373	4.731247	1.838414
C	1.034998	2.715734	1.164534
H	0.937254	2.307113	2.179571
H	0.041975	2.626461	0.696682
C	2.803100	4.335522	1.906933
H	3.082383	5.399337	1.985075
H	2.769719	3.935418	2.934210
C	2.141785	2.527133	-1.067301
H	1.173026	2.419340	-1.577316
H	2.887243	2.005858	-1.683772
C	2.366629	-0.872662	-1.010725
C	1.705132	-0.591702	-2.383459
H	0.626832	-0.811843	-2.321266
H	1.798564	0.468303	-2.657585
C	2.336501	-1.460769	-3.481049
H	1.858185	-1.215290	-4.442516
C	2.105240	-2.939441	-3.148388
H	1.024553	-3.153717	-3.104276
H	2.530046	-3.581094	-3.938168
C	2.760578	-3.256661	-1.799700
H	2.584162	-4.312017	-1.538624
C	4.268884	-2.987681	-1.883668
H	4.728438	-3.626104	-2.656474
H	4.753989	-3.241912	-0.926468
C	4.499875	-1.508337	-2.215694
H	5.580859	-1.302236	-2.269637
C	3.886446	-0.623240	-1.117079
H	4.092669	0.429127	-1.358241
H	4.374676	-0.835022	-0.151770
C	2.151208	-2.376904	-0.697217
H	2.630164	-2.640732	0.256604
H	1.076361	-2.591505	-0.584032
C	3.842337	-1.175016	-3.562350
H	4.014142	-0.115322	-3.816566
H	4.300149	-1.775411	-4.365884
P	1.496500	0.122442	0.319639
Pd	-0.901619	-0.111745	0.006500
Br	-1.087835	-2.019006	1.571195
C	-2.934481	-0.284919	-0.305182
C	-3.837867	0.456205	0.457779
C	-3.455522	-1.172252	-1.247895
C	-5.215361	0.303753	0.306674
H	-3.475608	1.163916	1.209215
C	-4.829975	-1.334696	-1.412708
H	-2.785403	-1.776830	-1.865683
C	-5.749182	-0.589975	-0.644520
H	-5.874106	0.895387	0.941953
H	-5.180457	-2.054042	-2.152599
N	-7.117883	-0.722655	-0.824874
C	-7.616064	-1.819902	-1.621015
H	-7.254584	-1.758842	-2.659760
H	-7.328606	-2.810308	-1.218367
H	-8.711582	-1.773809	-1.658177
C	-8.012622	-0.120833	0.135745
H	-9.050227	-0.309081	-0.166842
H	-7.877810	-0.517789	1.160582
H	-7.881028	0.972029	0.175110
C	-1.178716	1.219502	-1.300200
O	-1.419660	1.981811	-2.106050

int-5-ome.log

SCF (RwB97XD) = -4440.03262320
E(SCF)+ZPE(0 K)= -4439.301422
H(298 K)= -4439.264335
G(298 K)= -4439.370105
Lowest Frequency = 15.4417cm-1

C	-1.993846	0.327083	-1.878816
H	-3.092257	0.242789	-1.861125
H	-1.749601	1.397902	-1.888629
C	-1.416325	-0.283500	-3.156100
H	-0.318371	-0.334423	-3.074818
H	-1.773383	-1.316882	-3.293050
C	-1.784622	0.531410	-4.394606
H	-2.883665	0.614304	-4.462583
H	-1.404246	1.559087	-4.270050
C	-1.232132	-0.064210	-5.682822

H	-0.132071	-0.118989	-5.653323
H	-1.510367	0.541837	-6.558422
H	-1.611966	-1.085231	-5.850921
C	-2.266218	0.727942	0.997923
C	-2.185217	2.226032	0.602360
H	-2.689960	2.394527	-0.359934
H	-1.134116	2.527990	0.468729
C	-2.865145	3.106940	1.661892
H	-2.782332	4.158103	1.343879
C	-4.343618	2.712238	1.771772
H	-4.851534	2.870705	0.805700
H	-4.855176	3.348741	2.512724
C	-4.443291	1.238481	2.183948
H	-5.502075	0.942681	2.257344
C	-3.754862	1.035518	3.540701
H	-4.260907	1.634792	4.315624
H	-3.831480	-0.020544	3.850704
C	-2.280360	1.447842	3.432856
H	-1.779845	1.295956	4.402426
C	-1.577338	0.581282	2.377957
H	-0.523960	0.895278	2.294664
H	-1.572751	-0.466380	2.708576
C	-2.180415	2.920871	3.020490
H	-2.656809	3.564557	3.778468
H	-1.122471	3.224906	2.956755
C	-3.758256	0.352704	1.129049
H	-3.870708	-0.699417	1.426475
H	-4.265438	0.470791	0.157589
C	-1.739415	-2.088806	-0.201979
C	-3.103291	-2.423350	-0.852461
H	-3.918199	-1.904813	-0.327887
H	-3.126350	-2.080054	-1.896100
C	-3.357289	-3.939466	-0.825215
H	-4.335677	-4.138561	-1.290696
C	-3.362125	-4.438126	0.625096
H	-4.164211	-3.939693	1.195217
H	-3.571286	-5.520274	0.654715
C	-1.999267	-4.144166	1.265392
H	-1.998228	-4.485290	2.312667
C	-0.894816	-4.865831	0.484039
H	-1.061002	-5.955360	0.510679
H	0.086558	-4.678596	0.951302
C	-0.894433	-4.363398	-0.964581
H	-0.094748	-4.863851	-1.533101
C	-0.629641	-2.850707	-0.975293
H	-0.565417	-2.493916	-2.012371
H	0.351377	-2.650618	-0.516844
C	-1.749419	-2.627218	1.246847
H	-0.793792	-2.408108	1.746307
H	-2.537540	-2.136483	1.834441
C	-2.253542	-4.655616	-1.613832
H	-2.255000	-4.314260	-2.662590
H	-2.440980	-5.741997	-1.629151
P	-1.317830	-0.260434	-0.283163
Pd	1.045865	0.220767	0.002495
Br	1.038430	2.058992	-1.651355
C	3.041019	0.645548	0.311562
C	3.436453	1.548842	1.306310
C	4.025205	0.070757	-0.489057
C	4.774685	1.888767	1.472892
H	2.691730	2.012618	1.959316
C	5.376073	0.404140	-0.334869
H	3.753276	-0.642495	-1.272081
C	5.754756	1.320362	0.649458
H	5.081648	2.601895	2.241112
H	6.113890	-0.057642	-0.991687
O	7.035714	1.712939	0.885177
C	8.049790	1.212085	0.045287
H	8.989551	1.665095	0.386674
H	8.139181	0.114516	0.117823
H	7.882887	1.491538	-1.008956
C	1.454438	-1.035003	1.349981
O	1.778287	-1.740291	2.177743

int-5prime-cf3.log

SCF (RwB97XD) = -4662.61309542
E(SCF)+ZPE(0 K)= -4661.907966
H(298 K)= -4661.870226
G(298 K)= -4661.979406
Lowest Frequency = 13.1194cm-1

C	2.201101	-0.177533	1.928086
H	1.956096	-1.247703	1.963135
H	3.298912	-0.091981	1.966893
C	1.554537	0.482542	3.145972
H	1.892402	1.525908	3.251511
H	0.461417	0.513941	3.007208
C	1.866251	-0.273478	4.435925
H	1.501406	-1.309451	4.336225
H	2.960957	-0.339803	4.565619
C	1.239765	0.371598	5.665008
H	1.475810	-0.192814	6.579851
H	0.142540	0.411675	5.573905
H	1.600774	1.403272	5.807895
C	2.062342	2.169312	0.129512
C	0.915115	2.979955	0.787815
H	0.769015	2.668125	1.831988
H	-0.028236	2.773056	0.260761
C	1.212362	4.485648	0.731887
H	0.384135	5.024325	1.218925
C	2.526646	4.782226	1.465595
H	2.445273	4.487195	2.525291
H	2.734977	5.864769	1.448633
C	3.669856	4.014243	0.789606
H	4.615843	4.216034	1.317071
C	3.788789	4.450211	-0.676004
H	4.019963	5.526851	-0.733685
H	4.620818	3.915745	-1.164736
C	2.470786	4.149216	-1.400137
H	2.551499	4.443859	-2.458390
C	2.186892	2.639143	-1.338834
H	1.263752	2.409591	-1.887268
H	3.009459	2.111020	-1.840864
C	1.327527	4.923570	-0.733335
H	1.517815	6.008016	-0.793129
H	0.380091	4.731657	-1.263217
C	3.384720	2.505012	0.863215
H	4.223294	1.950577	0.417789
H	3.330371	2.210034	1.920202
C	2.587682	-0.717109	-0.917840
C	4.094347	-0.378275	-0.979570
H	4.248832	0.659318	-1.306889
H	4.546663	-0.473905	0.020906
C	4.811232	-1.319716	-1.962752
H	5.878776	-1.048839	-1.990907
C	4.198947	-1.157349	-3.360878
H	4.316096	-0.117057	-3.708428
H	4.729962	-1.798825	-4.083404
C	2.711802	-1.533353	-3.314580
H	2.266348	-1.410655	-4.314466
C	2.557443	-2.986470	-2.851297
H	3.059379	-3.669182	-3.556957
H	1.491277	-3.267352	-2.832535
C	3.164915	-3.131804	-1.451522
H	3.041938	-4.166893	-1.096457
C	2.449650	-2.194867	-0.465712
H	2.895550	-2.336172	0.529289
H	1.385453	-2.468608	-0.380657
C	1.975353	-0.608672	-2.335376
H	0.911849	-0.892542	-2.292347
H	2.010130	0.424280	-2.704850
C	4.656074	-2.772810	-1.497825
H	5.109720	-2.902271	-0.500966
H	5.190357	-3.450221	-2.184386
P	1.621309	0.353160	0.274263
Pd	-0.594482	-0.190596	0.133550
Br	-0.990994	-2.002905	1.847360
C	-2.554033	0.297645	-1.142008
C	-3.365605	0.798537	-0.115079

C -2.975558 -0.810531 -1.897279
C -4.601458 0.209226 0.143670
H -3.034758 1.653629 0.478295
C -4.205637 -1.393493 -1.637067
H -2.330692 -1.208394 -2.683577
C -5.013791 -0.882811 -0.614595
H -5.232197 0.598178 0.943323
H -4.537567 -2.253216 -2.222046
C -6.358595 -1.527702 -0.372816
F -6.944333 -1.093137 0.750850
F -7.209965 -1.269751 -1.386349
F -6.261687 -2.864177 -0.282580
C -1.162614 0.875007 -1.371436
O -0.804074 1.587310 -2.255227

int-5prime-cl.log

SCF (RwB97XD) = -4785.16424382
E(SCF)+ZPE(0 K)= -4784.474023
H(298 K)= -4784.438516
G(298 K)= -4784.542722
Lowest Frequency = 16.0333cm-1

C 1.486505 -0.212625 -2.238017
H 0.815775 0.525354 -2.698224
H 2.517560 0.065648 -2.508793
C 1.120321 -1.583234 -2.807031
H 1.848209 -2.346895 -2.489922
H 0.139378 -1.892009 -2.410315
C 1.053386 -1.567605 -4.332846
H 0.308441 -0.817671 -4.647497
H 2.024300 -1.231196 -4.737930
C 0.691604 -2.924735 -4.922169
H 0.653293 -2.889123 -6.021567
H -0.295573 -3.260024 -4.565837
H 1.427256 -3.695615 -4.639612
C 2.425141 -1.157535 0.401859
C 3.729807 -1.363765 -0.407252
H 4.279062 -0.415713 -0.501378
H 3.506155 -1.707172 -1.426741
C 4.624824 -2.410489 0.277026
H 5.542971 -2.528592 -0.320499
C 4.984279 -1.942209 1.692636
H 5.536502 -0.988393 1.647608
H 5.648741 -2.675883 2.178584
C 3.697059 -1.768016 2.507834
H 3.942905 -1.417351 3.522681
C 2.953729 -3.106705 2.590618
H 3.583767 -3.860016 3.092268
H 2.038841 -2.995691 3.195207
C 2.596416 -3.571106 1.173380
H 2.048862 -4.525821 1.219641
C 1.692900 -2.520969 0.510505
H 1.380280 -2.873735 -0.482596
H 0.775558 -2.397360 1.105388
C 3.878024 -3.748512 0.348741
H 4.520426 -4.518458 0.807223
H 3.631145 -4.099137 -0.667510
C 2.804669 -0.713265 1.833602
H 1.896883 -0.554132 2.430860
H 3.353704 0.238290 1.804644
C 1.742154 1.827849 -0.163804
C 1.324659 2.276352 1.257820
H 0.242967 2.113374 1.385694
H 1.825753 1.675034 2.026853
C 1.647679 3.761166 1.475165
H 1.360816 4.035126 2.502985
C 0.854205 4.604008 0.470338
H -0.227876 4.452707 0.619428
H 1.055125 5.676937 0.626472
C 1.253284 4.192739 -0.951236
H 0.674683 4.775368 -1.685022
C 2.753189 4.441366 -1.157338
H 2.984657 5.511054 -1.023155
H 3.042778 4.175927 -2.187828
C 3.547699 3.599395 -0.151014
H 4.627010 3.764493 -0.296812
C 3.248693 2.105525 -0.366531
H 3.852125 1.518597 0.340208
H 3.558301 1.808520 -1.381680
C 0.954770 2.702475 -1.174982
H 1.236705 2.446635 -2.206471
H -0.126331 2.509218 -1.083467
C 3.151721 3.996096 1.277842
H 3.727121 3.404130 2.009543
H 3.397165 5.055806 1.458368
P 1.255029 0.041180 -0.439233
Pd -0.978207 -0.184302 0.011823
Br -2.155593 0.196211 -2.198199
C -2.501472 -0.455239 1.808641
C -3.153332 -1.649099 1.461470
C -3.244701 0.728473 1.957574
C -4.533141 -1.667136 1.286881
H -2.577704 -2.568621 1.332754
C -4.621220 0.714238 1.784471
H -2.737256 1.661991 2.210307
C -5.253343 -0.485090 1.448929
H -5.045891 -2.591137 1.017495
H -5.204877 1.628190 1.899915
Cl -6.971411 -0.500112 1.226499
C -0.983657 -0.413112 1.924953
O -0.313315 -0.454996 2.907409

int-5prime-cn.log

SCF (RwB97XD) = -4417.75442426
E(SCF)+ZPE(0 K)= -4417.055303
H(298 K)= -4417.019407
G(298 K)= -4417.123734
Lowest Frequency = 13.2922cm-1

C 0.942965 -0.230103 2.623807
H 0.210239 -1.000235 2.897935
H 1.916708 -0.559946 3.018679
C 0.502096 1.081108 3.273522
H 1.289311 1.845718 3.180268
H -0.378021 1.480663 2.740818
C 0.156807 0.903316 4.751106
H -0.645553 0.152273 4.843774
H 1.031286 0.485443 5.279554
C -0.275415 2.201909 5.419810
H -1.168121 2.624068 4.930552
H 0.519859 2.963479 5.372774
H -0.522395 2.044723 6.480566
C 2.281965 0.971767 0.270774
C 1.591742 2.361973 0.237275
H 1.168751 2.609596 1.220959
H 0.757198 2.339657 -0.479704
C 2.589900 3.451339 -0.181278
H 2.067433 4.420908 -0.180268
C 3.762086 3.490912 0.807560
H 3.398672 3.732317 1.820886
H 4.471773 4.285632 0.524210
C 4.467892 2.128867 0.811193
H 5.307196 2.145406 1.524711
C 4.989818 1.814677 -0.596691
H 5.723816 2.576637 -0.907189
H 5.514030 0.844054 -0.598739
C 3.811324 1.780978 -1.578277
H 4.174864 1.541247 -2.589847
C 2.823059 0.685105 -1.148242
H 1.986965 0.629208 -1.862230
H 3.347228 -0.280856 -1.170205
C 3.109079 3.143605 -1.590725
H 3.809709 3.929223 -1.919420
H 2.272637 3.131915 -2.307937
C 3.475821 1.039542 1.254030
H 3.993592 0.070947 1.303331
H 3.128163 1.270535 2.271505
C 1.499198 -2.048385 0.407500

C	0.564297	-3.002558	1.195197
H	0.683646	-2.857608	2.278389
H	-0.487728	-2.782440	0.951851
C	0.883658	-4.469086	0.866479
H	0.201793	-5.110924	1.446552
C	2.336585	-4.768983	1.258378
H	2.477599	-4.614885	2.341360
H	2.576209	-5.824648	1.049486
C	3.274271	-3.848939	0.466764
H	4.320246	-4.052690	0.746340
C	3.083660	-4.090329	-1.037214
H	3.342821	-5.132146	-1.288666
H	3.763013	-3.441492	-1.615139
C	1.626212	-3.800639	-1.420001
H	1.486051	-3.960477	-2.500337
C	1.288596	-2.337914	-1.099277
H	0.242714	-2.129370	-1.373471
H	1.905025	-1.675664	-1.719565
C	2.958968	-2.378422	0.791482
H	3.656843	-1.736417	0.235988
H	3.127214	-2.190426	1.864613
C	0.690592	-4.724272	-0.632359
H	-0.357103	-4.534133	-0.918846
H	0.904262	-5.779932	-0.868692
P	1.000279	-0.287850	0.800467
Pd	-0.991713	0.141051	-0.206024
Br	-0.780853	0.789046	-2.629596
C	-3.335744	0.118008	0.548677
C	-3.634825	1.483619	0.441284
C	-4.129690	-0.835191	-0.107786
C	-4.733688	1.895640	-0.303465
H	-3.007912	2.222794	0.944760
C	-5.224396	-0.426932	-0.855034
H	-3.880620	-1.895782	-0.032357
C	-5.525435	0.940310	-0.952557
H	-4.974812	2.955816	-0.390382
H	-5.846784	-1.160293	-1.369599
C	-6.662151	1.365323	-1.727551
N	-7.575058	1.705843	-2.338570
C	-2.096584	-0.336953	1.305000
O	-2.066789	-0.872902	2.369544

int-5prime-h.log

SCF (RwB97XD) = -4325.55135898
E(SCF)+ZPE(0 K)= -4324.851378
H(298 K)= -4324.817111
G(298 K)= -4324.917388
Lowest Frequency = 19.8494cm-1

C	-1.022266	-0.253145	2.211050
H	-0.224345	0.328325	2.692068
H	-1.982658	0.145186	2.575818
C	-0.838685	-1.712559	2.627715
H	-1.698576	-2.322943	2.308393
H	0.051405	-2.125943	2.125726
C	-0.663523	-1.854713	4.138459
H	0.221333	-1.273257	4.447121
H	-1.529583	-1.400286	4.651502
C	-0.503360	-3.302640	4.582967
H	-0.369113	-3.376379	5.672853
H	0.375823	-3.766827	4.108132
H	-1.385094	-3.907723	4.314797
C	-2.228453	-0.770570	-0.438997
C	-3.508864	-0.857162	0.427644
H	-3.899567	0.148569	0.639738
H	-3.291270	-1.326813	1.396857
C	-4.587439	-1.686073	-0.289955
H	-5.482901	-1.722197	0.350706
C	-4.936991	-1.033375	-1.633346
H	-5.333141	-0.016817	-1.469964
H	-5.729310	-1.608991	-2.140168
C	-3.678605	-0.975221	-2.508389
H	-3.914682	-0.493531	-3.470395
C	-3.157603	-2.396066	-2.756730
H	-3.920575	-2.993535	-3.283235
H	-2.266684	-2.366050	-3.405175
C	-2.809718	-3.044766	-1.411525
H	-2.419773	-4.061778	-1.576452
C	-1.724043	-2.211671	-0.714678
H	-1.423214	-2.703039	0.221551
H	-0.827668	-2.169974	-1.351457
C	-4.062829	-3.107728	-0.528070
H	-4.838982	-3.722516	-1.013334
H	-3.826344	-3.591239	0.434754
C	-2.602032	-0.137112	-1.799269
H	-1.709777	-0.058033	-2.434610
H	-2.994707	0.879014	-1.653281
C	-1.065717	2.005642	0.362718
C	-0.651413	2.535094	-1.031686
H	0.386924	2.232047	-1.240999
H	-1.271817	2.095863	-1.823125
C	-0.758084	4.066002	-1.083413
H	-0.482578	4.401484	-2.095869
C	0.201491	4.673393	-0.053868
H	1.239123	4.381134	-0.285604
H	0.159356	5.774739	-0.091570
C	-0.186404	4.177599	1.343591
H	0.508803	4.588732	2.092184
C	-1.619577	4.619953	1.667066
H	-1.692442	5.720066	1.651048
H	-1.896912	4.293645	2.683406
C	-2.580089	4.013532	0.636510
H	-3.613642	4.317818	0.866696
C	-2.500889	2.477854	0.684794
H	-3.218271	2.065000	-0.038301
H	-2.804290	2.121228	1.682553
C	-0.107954	2.644377	1.402736
H	-0.377275	2.326436	2.420103
H	0.925388	2.304767	1.226216
C	-2.197762	4.496230	-0.769324
H	-2.891525	4.075540	-1.516763
H	-2.287502	5.593470	-0.830100
P	-0.844765	0.147854	0.432283
Pd	1.296419	-0.371169	-0.187044
Br	2.640223	-0.374012	1.964414
C	2.639668	-0.791813	-2.089184
C	3.023759	-2.117994	-1.829129
C	3.616141	0.210895	-2.228827
C	4.375747	-2.438071	-1.732120
H	2.261955	-2.892417	-1.711908
C	4.961389	-0.120714	-2.129520
H	3.309244	1.243388	-2.411861
C	5.339261	-1.442322	-1.879790
H	4.676077	-3.467872	-1.530380
H	5.721778	0.655173	-2.236815
H	6.398123	-1.695255	-1.792800
C	1.167113	-0.414984	-2.110459
O	0.464185	-0.191369	-3.044512

int-5prime-me.log

SCF (RwB97XD) = -4364.83237562
E(SCF)+ZPE(0 K)= -4364.104585
H(298 K)= -4364.068561
G(298 K)= -4364.173312
Lowest Frequency = 16.1199cm-1

C	-1.153901	0.261545	2.234894
H	-0.919324	1.334518	2.237918
H	-2.210865	0.156738	2.527266
C	-0.232182	-0.410297	3.252464
H	-0.509194	-1.466774	3.396866
H	0.801069	-0.398430	2.868801
C	-0.264221	0.297123	4.605793
H	0.038823	1.348024	4.463884
H	-1.302208	0.319918	4.982521
C	0.643472	-0.360370	5.637158
H	0.353809	-1.408356	5.819513
H	0.605626	0.167971	6.601970

H	1.691755	-0.358937	5.298079
C	-2.199735	0.857619	-0.422611
C	-3.674307	0.539766	-0.089250
H	-3.847533	0.624417	0.995906
H	-3.924023	-0.491616	-0.377081
C	-4.610011	1.502343	-0.841381
H	-5.651712	1.249732	-0.586086
C	-4.311534	2.949992	-0.430972
H	-4.480787	3.081065	0.650891
H	-4.998001	3.639702	-0.949448
C	-2.856147	3.285719	-0.781709
H	-2.624808	4.316618	-0.470639
C	-2.644483	3.139998	-2.292918
H	-3.304201	3.835162	-2.838561
H	-1.606912	3.403352	-2.557871
C	-2.939774	1.692342	-2.700241
H	-2.776761	1.567139	-3.782387
C	-1.985134	0.751097	-1.951892
H	-2.135234	-0.277108	-2.302990
H	-0.943340	1.017136	-2.190881
C	-4.392764	1.340405	-2.352301
H	-5.084735	1.995216	-2.907541
H	-4.616085	0.304168	-2.658069
C	-1.922364	2.330910	-0.022400
H	-0.868644	2.584539	-0.222063
H	-2.083672	2.475589	1.055382
C	-1.418197	-2.043237	0.403600
C	-2.524168	-2.417328	1.421038
H	-2.223329	-2.144035	2.441860
H	-3.450872	-1.866990	1.201959
C	-2.802421	-3.929366	1.378804
H	-3.593421	-4.158732	2.110698
C	-1.522788	-4.693202	1.743383
H	-1.194884	-4.419057	2.760254
H	-1.716803	-5.778607	1.749999
C	-0.424644	-4.358999	0.725307
H	0.501252	-4.895577	0.986480
C	-0.881610	-4.765698	-0.681113
H	-1.070009	-5.851537	-0.722229
H	-0.089328	-4.546872	-1.415632
C	-2.158710	-3.994612	-1.036377
H	-2.485168	-4.266273	-2.052749
C	-1.885163	-2.481987	-1.003508
H	-2.810613	-1.956211	-1.277602
H	-1.122800	-2.222442	-1.750555
C	-0.138719	-2.850472	0.747894
H	0.650426	-2.614659	0.018310
H	0.248303	-2.561191	1.735426
C	-3.262666	-4.334595	-0.027179
H	-4.193617	-3.803801	-0.288711
H	-3.489269	-5.413406	-0.057187
P	-0.965833	-0.224657	0.478513
Pd	1.147681	0.349374	-0.192684
Br	1.912753	2.109732	1.474743
C	2.740969	-0.029393	-1.878183
C	2.925551	1.201786	-2.530411
C	3.821636	-0.624192	-1.204000
C	4.174250	1.809446	-2.521593
H	2.085595	1.675210	-3.044034
C	5.064540	-0.004720	-1.207561
H	3.680491	-1.575107	-0.684651
C	5.260408	1.220630	-1.859733
H	4.308849	2.765837	-3.032244
H	5.899947	-0.476496	-0.684738
C	6.597838	1.904554	-1.813423
H	6.725957	2.602736	-2.652378
H	6.695736	2.484277	-0.881137
H	7.422163	1.177302	-1.836000
C	1.361024	-0.661467	-1.819081
O	0.828167	-1.394265	-2.590534

int-5prime-nme2.log

SCF (RwB97XD) = -4459.45324376
E(SCF)+ZPE(0 K)= -4458.679801
H(298 K)= -4458.641318
G(298 K)= -4458.751263
Lowest Frequency = 11.8138cm-1

C	1.845148	-0.564719	1.796437
H	1.481880	-1.597695	1.710033
H	2.944661	-0.606344	1.851936
C	1.252732	0.017876	3.079356
H	1.701707	0.997620	3.309153
H	0.172438	0.185947	2.937465
C	1.454774	-0.913203	4.273166
H	0.964052	-1.876412	4.054911
H	2.531365	-1.130980	4.389345
C	0.902281	-0.341889	5.572484
H	-0.179305	-0.148194	5.490624
H	1.391820	0.610356	5.835338
H	1.052578	-1.035508	6.413877
C	1.989869	1.966996	0.280404
C	3.335972	2.077698	1.036559
H	4.113596	1.489384	0.528360
H	3.244409	1.674147	2.054952
C	3.781562	3.547055	1.127106
H	4.742205	3.588709	1.665277
C	3.951196	4.126560	-0.283243
H	4.722439	3.562650	-0.834821
H	4.297134	5.172078	-0.225144
C	2.610811	4.050436	-1.025144
H	2.726384	4.450607	-2.044904
C	1.555875	4.865440	-0.267438
H	1.862669	5.923389	-0.209028
H	0.595072	4.836484	-0.806886
C	1.388765	4.282102	1.140877
H	0.621109	4.849047	1.691363
C	0.933339	2.819437	1.032066
H	0.751769	2.413014	2.037214
H	-0.025222	2.770668	0.493957
C	2.724528	4.353836	1.892375
H	3.048050	5.403162	1.993641
H	2.607630	3.953163	2.913475
C	2.167805	2.581701	-1.126889
H	1.226019	2.511288	-1.688834
H	2.932636	2.029244	-1.690774
C	2.217392	-0.818638	-1.084993
C	1.665339	-0.474150	-2.490080
H	0.573888	-0.623417	-2.495702
H	1.837951	0.581084	-2.736600
C	2.313090	-1.363278	-3.561317
H	1.917833	-1.070162	-4.546986
C	1.968849	-2.829538	-3.276559
H	0.876205	-2.974418	-3.308667
H	2.404571	-3.484489	-4.049604
C	2.510925	-3.210078	-1.894095
H	2.250552	-4.255855	-1.666172
C	4.035386	-3.037162	-1.874016
H	4.504563	-3.691336	-2.627794
H	4.437847	-3.337938	-0.892236
C	4.380965	-1.570210	-2.160031
H	5.474086	-1.434078	-2.139227
C	3.753768	-0.663667	-1.086741
H	4.038893	0.377546	-1.293985
H	4.163440	-0.920853	-0.096497
C	1.888295	-2.310722	-0.815530
H	2.285148	-2.621526	0.161592
H	0.796863	-2.457063	-0.775501
C	3.836275	-1.173002	-3.538940
H	4.090374	-0.121999	-3.758274
H	4.307760	-1.787798	-4.323643
P	1.339331	0.208499	0.213744
Pd	-0.930601	-0.047082	-0.042224
Br	-1.495394	-2.029925	1.474954
C	-2.795857	0.696306	-1.164449
C	-3.601005	1.157372	-0.102442
C	-3.279865	-0.365516	-1.957964
C	-4.835733	0.592322	0.152849
H	-3.245148	1.975328	0.528913

C	-4.510719	-0.935628	-1.709244
H	-2.666409	-0.744416	-2.779126
C	-5.323670	-0.480794	-0.636230
H	-5.418289	0.974226	0.989017
H	-4.840362	-1.757777	-2.341735
N	-6.525664	-1.059759	-0.372060
C	-6.958445	-2.216556	-1.128413
H	-7.051609	-1.987293	-2.202100
H	-6.265056	-3.067202	-1.012313
H	-7.944742	-2.533782	-0.771920
C	-7.293691	-0.642450	0.782286
H	-8.225635	-1.216925	0.823978
H	-6.745874	-0.812577	1.725032
H	-7.563308	0.424419	0.724587
C	-1.408744	1.246930	-1.386796
O	-0.989670	2.056392	-2.152236

int-5prime-ome.log

SCF (RwB97XD) = -4440.04712368
E(SCF)+ZPE(0 K)= -4439.314030
H(298 K)= -4439.277329
G(298 K)= -4439.383437
Lowest Frequency = 12.1668cm-1

C	-1.827029	0.316586	-1.814250
H	-2.924072	0.216964	-1.843107
H	-1.596288	1.390442	-1.802270
C	-1.186643	-0.272666	-3.070953
H	-0.091336	-0.287580	-2.947156
H	-1.505041	-1.316683	-3.221928
C	-1.531008	0.536556	-4.319747
H	-2.628327	0.578566	-4.437106
H	-1.194204	1.576139	-4.171379
C	-0.899428	-0.027940	-5.585412
H	0.199451	-0.041056	-5.506505
H	-1.161247	0.573896	-6.469005
H	-1.232295	-1.061393	-5.776389
C	-2.175377	0.716068	1.056460
C	-2.070921	2.214334	0.667572
H	-2.542732	2.390779	-0.309738
H	-1.013654	2.509178	0.569447
C	-2.779024	3.096139	1.707670
H	-2.679036	4.147765	1.396109
C	-4.263233	2.711721	1.768602
H	-4.739572	2.877636	0.787833
H	-4.793178	3.349122	2.495820
C	-4.386413	1.237071	2.170991
H	-5.449044	0.948187	2.208650
C	-3.743504	1.023997	3.548510
H	-4.270574	1.624049	4.308844
H	-3.836768	-0.032553	3.851718
C	-2.263518	1.426030	3.489687
H	-1.795960	1.267265	4.474400
C	-1.533147	0.557253	2.456250
H	-0.474952	0.859137	2.404010
H	-1.544152	-0.491220	2.780922
C	-2.140358	2.900125	3.087038
H	-2.637073	3.544614	3.831385
H	-1.078871	3.197201	3.058282
C	-3.674602	0.351580	1.133458
H	-3.806144	-0.701546	1.417994
H	-4.148025	0.482346	0.146680
C	-1.633995	-2.107461	-0.131587
C	-2.965935	-2.426821	-0.853475
H	-3.801867	-1.899832	-0.371171
H	-2.933275	-2.087240	-1.898220
C	-3.235398	-3.940613	-0.837658
H	-4.190211	-4.131074	-1.353601
C	-3.319867	-4.438738	0.610595
H	-4.145919	-3.932290	1.137932
H	-3.541008	-5.519033	0.628272
C	-1.989654	-4.156074	1.319826
H	-2.045167	-4.496584	2.365895
C	-0.853668	-4.889829	0.597280
H	-1.032591	-5.977866	0.613683
H	0.102738	-4.710846	1.115294
C	-0.772928	-4.387836	-0.849386
H	0.050446	-4.896673	-1.375494
C	-0.492457	-2.877849	-0.846121
H	-0.370915	-2.520877	-1.878828
H	0.459235	-2.682179	-0.329978
C	-1.721029	-2.642277	1.316943
H	-0.786777	-2.426654	1.853199
H	-2.536701	-2.145406	1.860349
C	-2.099083	-4.667617	-1.568283
H	-2.042932	-4.327209	-2.616018
H	-2.296529	-5.752159	-1.592674
P	-1.206637	-0.282204	-0.198011
Pd	1.014815	0.263932	-0.031561
Br	1.317835	2.202256	-1.653850
C	2.978133	-0.206318	1.136534
C	3.391080	0.878180	1.942469
C	3.764139	-0.576110	0.034295
C	4.561627	1.548628	1.658491
H	2.776097	1.184070	2.791953
C	4.946801	0.098411	-0.256524
H	3.454806	-1.412867	-0.596811
C	5.346237	1.168690	0.551630
H	4.893423	2.388308	2.271050
H	5.535011	-0.210122	-1.119342
O	6.460285	1.892413	0.355247
C	7.278419	1.600799	-0.763882
H	8.096123	2.331383	-0.741555
H	7.701489	0.585072	-0.699518
H	6.722182	1.713055	-1.708283
C	1.643996	-0.875810	1.390349
O	1.321481	-1.655255	2.230198

int-6-cf3.log

SCF (RwB97XD) = -4662.62637504
E(SCF)+ZPE(0 K)= -4661.921199
H(298 K)= -4661.883786
G(298 K)= -4661.990811
Lowest Frequency = 17.2371cm-1

C	-2.543133	1.107656	-0.116162
C	-2.230975	2.408416	-0.514660
H	-1.664759	2.573507	-1.433512
C	-2.656862	3.492189	0.248829
H	-2.421950	4.507166	-0.072824
C	-3.369053	3.270563	1.425867
C	-3.691168	1.970746	1.826596
H	-4.255363	1.802447	2.746226
C	-3.290690	0.891970	1.049081
H	-3.548684	-0.126005	1.347108
C	0.546553	0.545182	1.653621
H	-0.516917	0.419120	1.904924
H	1.117274	0.185953	2.525293
C	0.789645	2.034871	1.414339
H	1.869269	2.247823	1.360637
H	0.367425	2.327238	0.438244
C	0.161415	2.895142	2.509460
H	0.592963	2.615380	3.486204
H	-0.913615	2.657767	2.573182
C	0.338416	4.388972	2.275253
H	-0.109442	4.697019	1.316889
H	1.403965	4.668662	2.245528
H	-0.138985	4.980480	3.070674
C	0.386671	-2.278111	0.950764
C	0.136472	-3.262936	-0.217764
H	-0.652637	-2.862863	-0.873680
H	1.038997	-3.368972	-0.834820
C	-0.277825	-4.641188	0.318317
H	-0.426259	-5.319804	-0.536133
C	-1.585696	-4.506912	1.106577
H	-2.385279	-4.126633	0.450492
H	-1.911912	-5.492627	1.477562
C	-1.365021	-3.550001	2.283704

H -2.304436 -3.428333 2.846200
C -0.274945 -4.108115 3.207154
H -0.577753 -5.091533 3.602780
H -0.131454 -3.442128 4.074650
C 1.032939 -4.236987 2.417930
H 1.827595 -4.626565 3.073939
C 1.466423 -2.857890 1.891474
H 2.423061 -2.971338 1.363775
H 1.641537 -2.172325 2.737076
C -0.928266 -2.169753 1.766915
H -0.788022 -1.507023 2.633607
H -1.728473 -1.742274 1.146346
C 0.826040 -5.190839 1.232940
H 0.551358 -6.193490 1.600102
H 1.766581 -5.302817 0.667402
C 2.608049 -0.463808 -0.245376
C 3.564592 -0.292053 0.955333
H 3.281075 0.592475 1.545528
H 3.503590 -1.158355 1.628353
C 5.012306 -0.125973 0.464501
H 5.670145 -0.018713 1.341449
C 5.426376 -1.362230 -0.345093
H 5.368947 -2.264968 0.286161
H 6.474619 -1.266794 -0.672862
C 4.504141 -1.508313 -1.563607
H 4.790973 -2.400770 -2.141695
C 4.615901 -0.259999 -2.446426
H 3.974545 -0.364496 -3.337039
H 5.650985 -0.140482 -2.806417
C 4.192099 0.967812 -1.632252
H 4.242983 1.870139 -2.260974
C 2.741552 0.784454 -1.161299
H 2.400651 1.691303 -0.644381
H 2.100406 0.670700 -2.055978
C 3.054047 -1.684317 -1.084549
H 2.989316 -2.608501 -0.494242
H 2.383837 -1.808401 -1.952345
C 5.112867 1.126621 -0.415416
H 6.153694 1.276698 -0.746558
H 4.828016 2.020957 0.163931
C -2.089423 -0.099630 -0.890737
O -2.751413 -1.085019 -1.019564
P 0.802553 -0.594618 0.244700
Br -1.046623 0.882616 -3.863790
Pd -0.318277 0.039524 -1.678362
C -3.776335 4.417963 2.316360
F -5.105741 4.445744 2.522981
F -3.427335 5.611006 1.816342
F -3.203363 4.318799 3.533717

int-6-cl.log

SCF (RwB97XD) = -4785.17656750
E(SCF)+ZPE(0 K)= -4784.485906
H(298 K)= -4784.450796
G(298 K)= -4784.552629
Lowest Frequency = 12.0991cm-1

C -2.551068 1.103931 -0.098751
C -2.225534 2.409334 -0.478587
H -1.633511 2.580969 -1.380035
C -2.676299 3.490971 0.270640
H -2.441166 4.511468 -0.033958
C -3.426619 3.257475 1.422141
C -3.759194 1.962007 1.817452
H -4.352123 1.796770 2.718092
C -3.330475 0.888128 1.044504
H -3.598884 -0.130048 1.333540
C 0.549543 0.546702 1.652956
H -0.516200 0.426662 1.898099
H 1.114149 0.184808 2.527499
C 0.802712 2.034449 1.413347
H 1.884202 2.241378 1.374547
H 0.396120 2.326353 0.430507
C 0.163648 2.902604 2.495431
H 0.564175 2.610943 3.482008
H -0.917703 2.687008 2.529059
C 0.378323 4.392805 2.270379
H -0.029562 4.710202 1.297289
H 1.449469 4.651925 2.278913
H -0.116879 4.992226 3.048994
C 0.391283 -2.276595 0.951059
C 0.135169 -3.260063 -0.217298
H -0.655616 -2.858029 -0.869811
H 1.035384 -3.366955 -0.837674
C -0.279485 -4.638083 0.318970
H -0.432531 -5.315658 -0.535535
C -1.584255 -4.501837 1.112107
H -2.384997 -4.118476 0.459246
H -1.911622 -5.487292 1.482869
C -1.357241 -3.547007 2.289736
H -2.294376 -3.424328 2.855863
C -0.264895 -4.108495 3.208324
H -0.567736 -5.092079 3.603629
H -0.117160 -3.443982 4.076273
C 1.039821 -4.238292 2.413970
H 1.836434 -4.629774 3.066520
C 1.473467 -2.859283 1.887158
H 2.428074 -2.973939 1.356077
H 1.652706 -2.174895 2.732875
C -0.919789 -2.166877 1.773339
H -0.773649 -1.505952 2.640466
H -1.721809 -1.736440 1.157541
C 0.826802 -5.190643 1.228853
H 0.551860 -6.193240 1.596069
H 1.764969 -5.303601 0.659545
C 2.611475 -0.463371 -0.246500
C 3.569440 -0.297225 0.953898
H 3.288584 0.586659 1.546322
H 3.506795 -1.164790 1.625002
C 5.017380 -0.134064 0.462639
H 5.676129 -0.030984 1.339432
C 5.427341 -1.369597 -0.350222
H 5.367568 -2.273708 0.278876
H 6.475724 -1.276496 -0.678325
C 4.504000 -1.510034 -1.568586
H 4.788005 -2.401911 -2.149009
C 4.618948 -0.259877 -2.448353
H 3.976698 -0.360330 -3.338765
H 5.654179 -0.142433 -2.808704
C 4.199077 0.967040 -1.630807
H 4.252103 1.870745 -2.257417
C 2.748393 0.786886 -1.159143
H 2.411564 1.693342 -0.639031
H 2.105175 0.678196 -2.052677
C 3.053611 -1.682816 -1.089303
H 2.986219 -2.608516 -0.501711
H 2.382503 -1.802223 -1.957037
C 5.121270 1.120273 -0.414363
H 6.162363 1.267955 -0.745837
H 4.839471 2.014083 0.167320
C -2.088781 -0.098070 -0.869321
O -2.737188 -1.093850 -0.992573
P 0.805533 -0.592669 0.243574
Br -1.058648 0.897212 -3.853453
Pd -0.322969 0.046263 -1.672962
Cl -3.946600 4.603038 2.386764

int-6-cn.log

SCF (RwB97XD) = -4417.76390892
E(SCF)+ZPE(0 K)= -4417.065069
H(298 K)= -4417.029281
G(298 K)= -4417.132618
Lowest Frequency = 13.5861cm-1

C -2.033397 2.130425 -0.803477
C -3.243742 1.556748 -0.403691
H -3.499430 0.546832 -0.730632
C -4.127714 2.279007 0.388457

H	-5.080470	1.841230	0.689291
C	-3.785703	3.571342	0.808832
C	-2.573333	4.150817	0.409555
H	-2.317066	5.160118	0.735097
C	-1.707834	3.433113	-0.406259
H	-0.769465	3.882235	-0.736619
C	-0.074660	0.671540	1.886947
H	0.675137	0.720098	2.693057
H	-0.187716	1.696937	1.505511
C	-1.434453	0.224291	2.422574
H	-2.099347	-0.026963	1.579019
H	-1.332407	-0.696318	3.019141
C	-2.098032	1.303793	3.275456
H	-2.181634	2.226643	2.676548
H	-1.442177	1.552029	4.128009
C	-3.475369	0.900537	3.783365
H	-3.942636	1.708993	4.365503
H	-3.421233	0.010575	4.430876
H	-4.151884	0.658444	2.947929
C	0.884500	-2.018056	1.038042
C	-0.484710	-2.743530	0.921091
H	-1.247532	-2.244428	1.533162
H	-0.849996	-2.711249	-0.123336
C	-0.365094	-4.214691	1.345304
H	-1.357876	-4.683290	1.260019
C	0.125226	-4.289513	2.797028
H	-0.597957	-3.797208	3.468759
H	0.196025	-5.341996	3.117160
C	1.496238	-3.609534	2.906146
H	1.853831	-3.659309	3.946761
C	2.497229	-4.314273	1.980478
H	2.618125	-5.366846	2.285172
H	3.489517	-3.839899	2.064201
C	1.995080	-4.239419	0.531824
H	2.716098	-4.733671	-0.138049
C	1.872598	-2.765722	0.112428
H	1.530999	-2.701150	-0.934629
H	2.868839	-2.304891	0.155282
C	1.370452	-2.131394	2.500018
H	2.341856	-1.633312	2.626992
H	0.662769	-1.630644	3.178044
C	0.629793	-4.927987	0.422919
H	0.715800	-5.990406	0.703910
H	0.269964	-4.897706	-0.618751
C	2.222296	0.624505	0.092469
C	1.971783	2.155572	0.092639
H	1.163042	2.409445	-0.607020
H	1.666810	2.494004	1.093741
C	3.247668	2.916828	-0.301782
H	3.023403	3.995302	-0.297150
C	4.349079	2.612063	0.721047
H	4.038056	2.945547	1.725424
H	5.268193	3.165749	0.468045
C	4.624684	1.103759	0.727254
H	5.407947	0.871415	1.466258
C	5.080546	0.657782	-0.669402
H	5.295879	-0.424167	-0.671166
H	6.017882	1.170366	-0.941562
C	3.983997	0.979879	-1.694632
H	4.304500	0.656556	-2.697337
C	2.697489	0.225976	-1.326595
H	1.906174	0.454542	-2.057810
H	2.881528	-0.855184	-1.385277
C	3.346084	0.339980	1.113104
H	3.023272	0.636363	2.124929
H	3.576319	-0.733462	1.148739
C	3.705843	2.487341	-1.700244
H	4.613670	3.043318	-1.987090
H	2.930822	2.726072	-2.446317
C	-1.035921	1.387649	-1.653369
O	-0.377792	1.915400	-2.498827
P	0.613262	-0.253735	0.465768
Br	-2.516698	-1.273344	-2.941065
Pd	-0.880672	-0.512641	-1.280712
C	-4.674723	4.299462	1.675819
N	-5.378454	4.876357	2.379560

int-6-h.log

SCF (RwB97XD) = -4325.56224043
E(SCF)+ZPE(0 K)= -4324.861944
H(298 K)= -4324.828023
G(298 K)= -4324.926591
Lowest Frequency = 18.5890cm-1

C	-2.559904	1.090616	-0.086515
C	-2.240640	2.402742	-0.450639
H	-1.635018	2.584518	-1.341066
C	-2.724868	3.468389	0.304162
H	-2.494163	4.492301	0.003524
C	-3.498228	3.229239	1.440222
C	-3.807783	1.920430	1.812386
H	-4.415752	1.730814	2.699558
C	-3.353216	0.852181	1.043795
H	-3.610660	-0.173728	1.315528
C	0.558529	0.555318	1.649656
H	-0.508150	0.440360	1.893240
H	1.121348	0.193599	2.525450
C	0.817398	2.041106	1.405102
H	1.899748	2.243817	1.366857
H	0.412221	2.330371	0.420899
C	0.180246	2.916368	2.482133
H	0.574483	2.624557	3.471345
H	-0.903184	2.710409	2.508994
C	0.409526	4.403773	2.252327
H	0.005205	4.721154	1.277770
H	1.483093	4.652901	2.260853
H	-0.078995	5.011363	3.029174
C	0.393816	-2.268347	0.949654
C	0.133595	-3.251224	-0.218270
H	-0.655823	-2.846245	-0.870462
H	1.033184	-3.361600	-0.838982
C	-0.285856	-4.627626	0.318366
H	-0.442000	-5.304809	-0.535932
C	-1.589700	-4.486373	1.112227
H	-2.389046	-4.099482	0.459745
H	-1.920907	-5.470590	1.482987
C	-1.358316	-3.532530	2.289796
H	-2.294683	-3.406194	2.856428
C	-0.267583	-4.098207	3.207683
H	-0.573586	-5.080864	3.602992
H	-0.117049	-3.434308	4.075646
C	1.036205	-4.232390	2.412481
H	1.831895	-4.626685	3.064531
C	1.474325	-2.854848	1.885280
H	2.428434	-2.972996	1.354068
H	1.656265	-2.171090	2.730946
C	-0.916000	-2.154162	1.773163
H	-0.766716	-1.493556	2.639980
H	-1.717002	-1.720375	1.158795
C	0.818937	-5.184148	1.227629
H	0.540629	-6.185742	1.595190
H	1.756315	-5.300521	0.657679
C	2.618556	-0.462121	-0.247667
C	3.576236	-0.299129	0.953419
H	3.298314	0.586297	1.544952
H	3.509513	-1.165874	1.625176
C	5.025387	-0.142174	0.463655
H	5.683743	-0.041278	1.341045
C	5.431112	-1.379906	-0.348012
H	5.366790	-2.283358	0.281624
H	6.480299	-1.291443	-0.674968
C	4.508474	-1.517265	-1.567286
H	4.789463	-2.410692	-2.146861
C	4.629571	-0.268121	-2.447666
H	3.987791	-0.366495	-3.338626
H	5.665702	-0.155033	-2.806957
C	4.213634	0.960873	-1.631242
H	4.270952	1.863976	-2.258387
C	2.761779	0.787085	-1.160770

H	2.428861	1.695224	-0.641197
H	2.118203	0.681041	-2.054158
C	3.056805	-1.683508	-1.089610
H	2.984797	-2.608765	-0.501939
H	2.386098	-1.800124	-1.957963
C	5.135388	1.111126	-0.414103
H	6.177440	1.254292	-0.744676
H	4.856654	2.006443	0.166767
C	-2.084743	-0.098929	-0.868412
O	-2.721943	-1.099658	-1.008586
P	0.811199	-0.585254	0.240771
Br	-1.044975	0.917496	-3.854326
Pd	-0.318402	0.061314	-1.671559
H	-3.863671	4.067401	2.037813

int-6-me.log

SCF (RwB97XD) = -4364.84313476
E(SCF)+ZPE(0 K)= -4364.115468
H(298 K)= -4364.079716
G(298 K)= -4364.183112
Lowest Frequency = 10.9046cm-1

C	-2.544515	1.111835	-0.094501
C	-2.209831	2.409771	-0.487878
H	-1.622052	2.566517	-1.394883
C	-2.650930	3.498806	0.258259
H	-2.400981	4.509410	-0.073496
C	-3.400328	3.321970	1.426600
C	-3.727399	2.013769	1.812869
H	-4.317563	1.851371	2.718771
C	-3.320123	0.920727	1.057579
H	-3.597131	-0.091684	1.359870
C	0.541752	0.532364	1.654388
H	-0.524414	0.411278	1.896837
H	1.105613	0.166267	2.527792
C	0.793645	2.021831	1.424294
H	1.874905	2.230389	1.385135
H	0.383011	2.320540	0.445494
C	0.155193	2.879621	2.515188
H	0.560269	2.581440	3.498111
H	-0.925193	2.659804	2.547282
C	0.364130	4.372310	2.301063
H	-0.061325	4.697494	1.338327
H	1.434929	4.633118	2.293937
H	-0.115787	4.964759	3.094858
C	0.393993	-2.288444	0.948739
C	0.138786	-3.271931	-0.219809
H	-0.654888	-2.871691	-0.869928
H	1.037832	-3.374852	-0.842672
C	-0.269923	-4.652003	0.315647
H	-0.422173	-5.329281	-0.539292
C	-1.573709	-4.521072	1.111274
H	-2.376886	-4.139686	0.460312
H	-1.897014	-5.508095	1.481661
C	-1.347870	-3.566382	2.289253
H	-2.284517	-3.447545	2.857023
C	-0.252047	-4.125148	3.205358
H	-0.550739	-5.110146	3.600491
H	-0.104903	-3.460794	4.073579
C	1.051730	-4.249784	2.408570
H	1.850848	-4.639253	3.059381
C	1.479621	-2.868868	1.882021
H	2.433591	-2.979930	1.348955
H	1.658348	-2.184842	2.728154
C	-0.916157	-2.184282	1.773360
H	-0.770676	-1.523824	2.640933
H	-1.720437	-1.755385	1.159497
C	0.839814	-5.201809	1.222997
H	0.568993	-6.205747	1.589761
H	1.777375	-5.310968	0.651893
C	2.605602	-0.465292	-0.247488
C	3.563036	-0.301452	0.953750
H	3.279769	0.579502	1.549373
H	3.502467	-1.171483	1.621743
C	5.010912	-0.133074	0.464017
H	5.669105	-0.032175	1.341558
C	5.424074	-1.364406	-0.353641
H	5.366070	-2.271176	0.271836
H	6.472441	-1.267610	-0.680902
C	4.501486	-1.502126	-1.572908
H	4.788048	-2.390892	-2.156898
C	4.613877	-0.248060	-2.447439
H	3.971984	-0.346357	-3.338325
H	5.649004	-0.126880	-2.807067
C	4.190830	0.974522	-1.624955
H	4.242084	1.880796	-2.248089
C	2.740427	0.788973	-1.154819
H	2.401267	1.692189	-0.630493
H	2.096526	0.683393	-2.047624
C	3.051351	-1.680241	-1.094788
H	2.986052	-2.608475	-0.510856
H	2.380702	-1.797907	-1.963111
C	5.112639	1.124928	-0.407916
H	6.153613	1.276065	-0.738427
H	4.828750	2.015792	0.177320
C	-2.101200	-0.100381	-0.852661
O	-2.749169	-1.098100	-0.962164
P	0.798991	-0.601540	0.240429
Br	-1.065609	0.851809	-3.868161
Pd	-0.339382	0.022789	-1.674955
C	-3.823228	4.495224	2.266701
H	-3.265537	4.514421	3.217054
H	-3.640633	5.448856	1.752278
H	-4.892938	4.441149	2.519598

int-6-nme2.log

SCF (RwB97XD) = -4459.46264289
E(SCF)+ZPE(0 K)= -4458.688970
H(298 K)= -4458.650718
G(298 K)= -4458.759032
Lowest Frequency = 16.0090cm-1

C	1.948081	-1.885964	1.344813
C	1.769818	-3.275545	1.263507
H	0.882012	-3.727429	1.711939
C	2.691124	-4.082213	0.617599
H	2.501689	-5.153488	0.567887
C	3.862111	-3.529547	0.040167
C	4.049461	-2.129494	0.157040
H	4.943204	-1.656143	-0.246181
C	3.104898	-1.333029	0.780513
H	3.277607	-0.257065	0.856649
C	0.219470	-0.811242	-1.663050
H	0.404459	-1.757301	-1.132223
H	-0.484836	-1.039261	-2.479753
C	1.557587	-0.323066	-2.215008
H	1.406800	0.518393	-2.910185
H	2.177316	0.064453	-1.389469
C	2.323459	-1.431213	-2.934910
H	1.712963	-1.816558	-3.770195
H	2.462987	-2.275567	-2.239085
C	3.678684	-0.971292	-3.455065
H	4.315806	-0.609006	-2.632270
H	3.574606	-0.145577	-4.177567
H	4.214849	-1.787677	-3.962996
C	-2.196109	-0.700341	-0.044119
C	-3.272704	-0.601652	-1.147621
H	-3.565091	0.443812	-1.315150
H	-2.875149	-0.983637	-2.102763
C	-4.526006	-1.400621	-0.748638
H	-5.276079	-1.297876	-1.549254
C	-5.090596	-0.839101	0.564223
H	-5.368418	0.220603	0.434453
H	-6.010522	-1.380127	0.841313
C	-4.039575	-0.976439	1.674506
H	-4.437605	-0.569600	2.617348
C	-3.671502	-2.452849	1.861709
H	-4.558831	-3.033426	2.164590

H	-2.924699	-2.556414	2.665254
C	-3.108998	-2.999763	0.544567
H	-2.821329	-4.055693	0.671671
C	-1.859073	-2.204277	0.135549
H	-1.476788	-2.624978	-0.805956
H	-1.078234	-2.333507	0.897198
C	-2.781252	-0.183516	1.293500
H	-2.023988	-0.278537	2.086709
H	-3.032958	0.883309	1.219292
C	-4.164928	-2.878770	-0.561078
H	-5.064373	-3.459431	-0.297027
H	-3.776649	-3.297796	-1.504747
C	-0.962354	1.889704	-1.228081
C	-1.371730	1.780465	-2.713744
H	-0.595054	1.250011	-3.285369
H	-2.298383	1.200143	-2.819779
C	-1.574500	3.180341	-3.316998
H	-1.876922	3.069008	-4.370582
C	-2.671207	3.920816	-2.539424
H	-3.622185	3.365621	-2.605730
H	-2.847468	4.913485	-2.985474
C	-2.246497	4.070539	-1.071744
H	-3.035754	4.591802	-0.507414
C	-0.939517	4.867853	-0.993101
H	-0.636445	4.999734	0.058628
H	-1.082079	5.875640	-1.417001
C	0.151257	4.116132	-1.764590
H	1.103460	4.665020	-1.696442
C	0.344500	2.725793	-1.140958
H	1.175759	2.209176	-1.638731
H	0.641627	2.856006	-0.084363
C	-2.047443	2.676203	-0.455993
H	-3.007158	2.142651	-0.480145
H	-1.757792	2.770519	0.604435
C	-0.260976	3.968379	-3.235151
H	-0.384023	4.962484	-3.696035
H	0.529444	3.447206	-3.801084
C	0.896460	-1.076397	2.004940
O	0.148422	-1.476321	2.849360
P	-0.613451	0.236397	-0.411030
Br	2.232450	1.790281	3.052242
Pd	0.733900	0.773922	1.392185
N	4.770963	-4.313402	-0.609708
C	4.577458	-5.744571	-0.682152
H	3.645958	-6.006436	-1.212502
H	5.410837	-6.196350	-1.231753
H	4.544463	-6.207852	0.318734
C	5.956070	-3.721612	-1.190594
H	6.605249	-3.258294	-0.427707
H	6.538201	-4.498894	-1.698260
H	5.701135	-2.951937	-1.938009

int-6-ome.log

SCF (RwB97XD) = -4440.05782935
E(SCF)+ZPE(0 K)= -4439.324669
H(298 K)= -4439.288299
G(298 K)= -4439.392195
Lowest Frequency = 17.4803cm-1

C	2.028693	-1.873972	1.174029
C	1.825883	-3.255153	1.012294
H	0.933424	-3.720871	1.435811
C	2.740580	-4.023163	0.313141
H	2.583539	-5.093287	0.168016
C	3.897754	-3.430337	-0.218817
C	4.124765	-2.060932	-0.031851
H	5.024647	-1.580022	-0.413119
C	3.182914	-1.292631	0.646492
H	3.360786	-0.224216	0.787694
C	0.178993	-0.786272	-1.738786
H	0.372155	-1.742907	-1.230885
H	-0.544402	-0.997817	-2.543104
C	1.505776	-0.291052	-2.311626
H	1.340483	0.559425	-2.992347
H	2.143004	0.085978	-1.494258
C	2.255041	-1.390383	-3.061803
H	1.623875	-1.768400	-3.884744
H	2.412505	-2.242938	-2.379614
C	3.595399	-0.922816	-3.612698
H	4.253268	-0.564968	-2.804210
H	3.470417	-0.091336	-4.325039
H	4.119360	-1.733644	-4.141732
C	-2.191412	-0.701459	-0.051301
C	-3.295316	-0.594161	-1.126611
H	-3.593617	0.452313	-1.276595
H	-2.920559	-0.965838	-2.094919
C	-4.536258	-1.400171	-0.704075
H	-5.306339	-1.291519	-1.484630
C	-5.068722	-0.853000	0.628097
H	-5.352391	0.207178	0.516177
H	-5.980121	-1.399087	0.922710
C	-3.990052	-0.998986	1.710474
H	-4.365021	-0.602530	2.667067
C	-3.614649	-2.476428	1.873288
H	-4.493211	-3.062016	2.191297
H	-2.848894	-2.586998	2.657882
C	-3.083494	-3.008710	0.537270
H	-2.790483	-4.065061	0.646737
C	-1.845840	-2.206036	0.105538
H	-1.485630	-2.617307	-0.848722
H	-1.046586	-2.340199	0.847414
C	-2.743141	-0.199326	1.305887
H	-1.965844	-0.300502	2.079077
H	-2.998781	0.867642	1.249273
C	-4.166664	-2.879108	-0.540746
H	-5.057474	-3.465210	-0.260328
H	-3.800966	-3.287041	-1.498149
C	-0.991884	1.905979	-1.237612
C	-1.438650	1.813155	-2.713595
H	-0.675851	1.290723	-3.310686
H	-2.366973	1.232669	-2.802584
C	-1.658141	3.219636	-3.294998
H	-1.987049	3.119737	-4.341679
C	-2.735155	3.949878	-2.481261
H	-3.687282	3.394937	-2.530280
H	-2.922787	4.947579	-2.911080
C	-2.273533	4.082727	-1.023079
H	-3.048493	4.596779	-0.433037
C	-0.965336	4.880220	-0.968371
H	-0.634894	4.999421	0.076606
H	-1.119620	5.893014	-1.375739
C	0.105900	4.139433	-1.776938
H	1.058980	4.688627	-1.726799
C	0.316644	2.741996	-1.175092
H	1.134468	2.231915	-1.700877
H	0.643153	2.861251	-0.125644
C	-2.057926	2.681538	-0.428862
H	-3.017182	2.146610	-0.435338
H	-1.742159	2.763885	0.625118
C	-0.343740	4.008355	-3.237939
H	-0.479820	5.007614	-3.683597
H	0.432468	3.495310	-3.830339
C	0.983861	-1.088261	1.891990
O	0.284138	-1.525715	2.757019
P	-0.622632	0.242412	-0.452104
Br	2.315256	1.769312	2.943101
Pd	0.773093	0.764615	1.318930
O	4.724737	-4.252546	-0.894980
C	5.885219	-3.704042	-1.489879
H	6.386127	-4.531341	-2.007459
H	5.629607	-2.923246	-2.224734
H	6.571596	-3.288102	-0.734396

int-7-cf3.log

SCF (RwB97XD) = -4663.80629947
E(SCF)+ZPE(0 K)= -4663.086047
H(298 K)= -4663.047061
G(298 K)= -4663.157922

Lowest Frequency = 8.0421cm-1

C	-2.506395	1.622672	-0.010393
C	-1.873542	2.444786	-0.947811
H	-1.270658	1.997395	-1.739932
C	-2.010031	3.824533	-0.875037
H	-1.512631	4.462477	-1.608164
C	-3.430754	3.580291	1.073380
H	-4.035564	4.022723	1.865613
C	-3.292829	2.197755	0.991395
H	-3.791005	1.549644	1.714405
C	0.257478	-0.007526	1.976147
H	-0.757750	-0.308450	2.270435
H	0.931471	-0.397217	2.755558
C	0.311657	1.521311	1.939975
H	1.354854	1.870833	1.898661
H	-0.168684	1.900458	1.026794
C	-0.372856	2.152053	3.150715
H	0.084902	1.761368	4.076286
H	-1.427967	1.829739	3.172723
C	-0.303542	3.673180	3.138675
H	-0.758166	4.083407	2.222931
H	0.738948	4.028064	3.177348
H	-0.835175	4.108691	3.998126
C	0.522315	-2.727169	0.973110
C	0.445275	-3.653335	-0.266196
H	-0.429894	-3.376736	-0.875885
H	1.332952	-3.534958	-0.902715
C	0.319171	-5.123311	0.160242
H	0.291135	-5.749076	-0.745429
C	-0.976350	-5.307230	0.958600
H	-1.845874	-5.043615	0.334443
H	-1.097405	-6.362522	1.254332
C	-0.927150	-4.412983	2.202265
H	-1.861359	-4.519750	2.775477
C	0.268394	-4.812097	3.077159
H	0.170066	-5.860914	3.402572
H	0.294712	-4.193685	3.990004
C	1.564045	-4.628867	2.277157
H	2.429561	-4.904827	2.900296
C	1.715679	-3.157281	1.853079
H	2.662437	-3.045349	1.305478
H	1.780941	-2.517724	2.748411
C	-0.780411	-2.940803	1.788069
H	-0.764790	-2.328752	2.701539
H	-1.653172	-2.618557	1.199426
C	1.524778	-5.515598	1.024670
H	1.454611	-6.577315	1.313428
H	2.459442	-5.400957	0.449632
C	2.274930	-0.417646	-0.152614
C	3.257249	-0.171929	1.018690
H	2.847333	0.565339	1.721656
H	3.418292	-1.098675	1.586733
C	4.606411	0.343594	0.490262
H	5.276353	0.512134	1.348327
C	5.221361	-0.699128	-0.451095
H	5.394544	-1.644189	0.090483
H	6.202476	-0.352535	-0.815560
C	4.271821	-0.933327	-1.632605
H	4.696105	-1.695203	-2.305358
C	4.068185	0.379729	-2.397754
H	3.407528	0.216618	-3.265580
H	5.032186	0.742309	-2.790728
C	3.453651	1.420544	-1.454243
H	3.287762	2.364728	-1.996397
C	2.100306	0.905918	-0.943948
H	1.627518	1.673523	-0.315776
H	1.422912	0.737393	-1.797229
C	2.921074	-1.448547	-1.107365
H	3.086163	-2.402851	-0.588314
H	2.259815	-1.656720	-1.962707
C	4.393373	1.661649	-0.265847
H	5.359497	2.056062	-0.621108
H	3.964656	2.421639	0.408803
C	-2.329732	0.121863	-0.001601
O	-2.976534	-0.571143	0.737527
P	0.563260	-0.934101	0.422419
Br	-2.840552	-0.494177	-2.997961
Pd	-1.038084	-0.683729	-1.270600
H	0.305120	-1.619657	-2.385536
H	-0.234523	-1.419676	-2.898714
C	-2.782076	4.391265	0.143201
C	-2.889042	5.894300	0.212481
F	-3.563642	6.316718	1.291980
F	-1.672604	6.474411	0.258073
F	-3.512963	6.401944	-0.866972

int-7-cl.log

SCF (RwB97XD) = -4786.35684884
E(SCF)+ZPE(0 K)= -4785.651055
H(298 K)= -4785.614456
G(298 K)= -4785.719110
Lowest Frequency = 12.6161cm-1

C	-2.506417	1.633407	0.002398
C	-1.872017	2.455455	-0.932921
H	-1.254907	2.008845	-1.714968
C	-2.022102	3.836833	-0.881678
H	-1.528620	4.477820	-1.613374
C	-3.467212	3.593820	1.056925
H	-4.086779	4.048639	1.831070
C	-3.309950	2.214140	0.990415
H	-3.810955	1.569501	1.714996
C	0.267211	-0.011595	1.987324
H	-0.746184	-0.315473	2.284585
H	0.945493	-0.400655	2.763294
C	0.316166	1.517402	1.951580
H	1.358514	1.870546	1.916354
H	-0.160979	1.894502	1.035877
C	-0.378834	2.145413	3.157684
H	0.075447	1.758274	4.086509
H	-1.432210	1.817159	3.173019
C	-0.319068	3.666843	3.143956
H	-0.774016	4.072384	2.226131
H	0.721097	4.028401	3.185877
H	-0.856587	4.099977	4.000943
C	0.526694	-2.727941	0.975223
C	0.443439	-3.650531	-0.266369
H	-0.434430	-3.371649	-0.871028
H	1.328015	-3.530720	-0.906920
C	0.318463	-5.121704	0.156267
H	0.286199	-5.744792	-0.751134
C	-0.973813	-5.307264	0.959500
H	-1.845743	-5.041363	0.339695
H	-1.094236	-6.363347	1.252772
C	-0.918888	-4.416566	2.205445
H	-1.850792	-4.524333	2.782228
C	0.279922	-4.819260	3.074242
H	0.182210	-5.869032	3.396839
H	0.310423	-4.203612	3.988836
C	1.572422	-4.634415	2.269480
H	2.440337	-4.912894	2.888202
C	1.723190	-3.161647	1.849181
H	2.667960	-3.048650	1.298390
H	1.792297	-2.524885	2.746216
C	-0.772681	-2.943265	1.795112
H	-0.752542	-2.334222	2.710477
H	-1.647599	-2.617795	1.211511
C	1.527434	-5.517275	1.014459
H	1.457911	-6.579864	1.300207
H	2.459717	-5.401313	0.435812
C	2.277330	-0.416941	-0.148922
C	3.263570	-0.174426	1.019648
H	2.856647	0.562948	1.724332
H	3.424723	-1.101919	1.586452
C	4.612078	0.339578	0.488021
H	5.285150	0.505211	1.344217
C	5.222251	-0.702293	-0.457420
H	5.395207	-1.648884	0.081561

H	6.202970	-0.356765	-0.824021
C	4.268754	-0.932261	-1.636575
H	4.689876	-1.693299	-2.312290
C	4.064625	0.382727	-2.398247
H	3.400864	0.222261	-3.264178
H	5.027860	0.744781	-2.793655
C	3.454723	1.422407	-1.450459
H	3.288465	2.367980	-1.990115
C	2.102368	0.908807	-0.936414
H	1.633427	1.675480	-0.304189
H	1.421201	0.743827	-1.787360
C	2.918923	-1.446442	-1.108227
H	3.084133	-2.402317	-0.592143
H	2.254373	-1.651448	-1.961826
C	4.399074	1.659487	-0.264890
H	5.364797	2.052681	-0.622709
H	3.974112	2.418974	0.412703
C	-2.323414	0.138623	0.022512
O	-2.957708	-0.556205	0.771684
P	0.566336	-0.932866	0.429605
Br	-2.849633	-0.488049	-2.978445
Pd	-1.042149	-0.674772	-1.255016
H	0.300773	-1.607036	-2.383403
H	-0.255064	-1.431696	-2.887522
C	-2.815508	4.395147	0.119899
Cl	-2.989120	6.120718	0.207929

int-7-cn.log

SCF (RwB97XD) = -4418.94464333
E(SCF)+ZPE(0 K)= -4418.230485
H(298 K)= -4418.193392
G(298 K)= -4418.298868
Lowest Frequency = 14.8961cm-1

C	-2.494295	1.675519	0.009244
C	-1.862289	2.479620	-0.942887
H	-1.240635	2.019038	-1.712658
C	-2.024214	3.858811	-0.919193
H	-1.531987	4.486268	-1.663597
C	-3.472672	3.642654	1.024183
H	-4.098156	4.104136	1.789859
C	-3.304561	2.264721	0.986249
H	-3.799927	1.627321	1.720432
C	0.286317	-0.021075	2.008161
H	-0.721348	-0.333141	2.315752
H	0.976098	-0.404474	2.776507
C	0.322475	1.508033	1.968244
H	1.362119	1.869479	1.936516
H	-0.151302	1.878238	1.047606
C	-0.384960	2.135050	3.167486
H	0.065757	1.753924	4.100350
H	-1.435934	1.798643	3.177423
C	-0.335623	3.656737	3.149788
H	-0.785120	4.057081	2.226903
H	0.701437	4.025843	3.199161
H	-0.883025	4.088833	4.000918
C	0.532192	-2.733811	0.979247
C	0.444090	-3.649242	-0.267423
H	-0.435584	-3.366210	-0.867450
H	1.326650	-3.527150	-0.910149
C	0.318099	-5.122528	0.147526
H	0.282908	-5.740375	-0.763309
C	-0.972712	-5.310596	0.952487
H	-1.845712	-5.040655	0.335911
H	-1.093830	-6.367944	1.240642
C	-0.913910	-4.426485	2.202913
H	-1.844711	-4.535747	2.781128
C	0.286120	-4.835626	3.067065
H	0.187161	-5.886879	3.384224
H	0.319531	-4.224961	3.984876
C	1.577360	-4.648873	2.260674
H	2.445976	-4.932258	2.876072
C	1.729922	-3.174052	1.848470
H	2.673810	-3.059001	1.296523
H	1.801624	-2.542771	2.749102
C	-0.766227	-2.951156	1.800247
H	-0.743924	-2.347221	2.718864
H	-1.641681	-2.621476	1.219550
C	1.528381	-5.524490	1.000803
H	1.457822	-6.588526	1.280599
H	2.459547	-5.406697	0.420784
C	2.283398	-0.419375	-0.141777
C	3.275768	-0.181162	1.022477
H	2.874101	0.556728	1.729858
H	3.437136	-1.109864	1.587386
C	4.622693	0.330545	0.484614
H	5.300724	0.492447	1.337505
C	5.225236	-0.710453	-0.466655
H	5.398512	-1.658977	0.068769
H	6.204909	-0.366424	-0.837238
C	4.265285	-0.934829	-1.641623
H	4.681158	-1.694995	-2.321495
C	4.059666	0.382511	-2.398722
H	3.391040	0.225492	-3.261537
H	5.021341	0.743829	-2.798377
C	3.457229	1.420957	-1.444861
H	3.289985	2.368292	-1.981043
C	2.106619	0.909070	-0.924298
H	1.643921	1.675014	-0.286515
H	1.420069	0.748382	-1.771780
C	2.917304	-1.447583	-1.107535
H	3.083220	-2.405206	-0.595010
H	2.247324	-1.648487	-1.958003
C	4.408765	1.652781	-0.263969
H	5.373394	2.044304	-0.626348
H	3.989757	2.411848	0.417757
C	-2.301533	0.174945	0.057784
O	-2.928035	-0.496265	0.833930
P	0.576475	-0.935677	0.446169
Br	-2.868378	-0.467399	-2.925289
Pd	-1.042333	-0.661233	-1.224527
H	0.283336	-1.598660	-2.354410
H	-0.281545	-1.431264	-2.852577
C	-2.827512	4.442384	0.070422
C	-2.979234	5.873259	0.116958
N	-3.093274	7.017269	0.159694

int-7-d2-cf3.log

SCF (RwB97XD) = -4663.80629947
E(SCF)+ZPE(0 K)= -4663.090366
H(298 K)= -4663.050874
G(298 K)= -4663.162889
Lowest Frequency = 8.0385cm-1

C	-2.506393	1.622676	-0.010388
C	-1.873534	2.444787	-0.947805
H	-1.270645	1.997393	-1.739922
C	-2.010023	3.824534	-0.875037
H	-1.512619	4.462475	-1.608163
C	-3.430757	3.580299	1.073372
H	-4.035572	4.022735	1.865600
C	-3.292832	2.197763	0.991393
H	-3.791012	1.549654	1.714403
C	0.257482	-0.007528	1.976153
H	-0.757745	-0.308453	2.270443
H	0.931476	-0.397219	2.755563
C	0.311659	1.521309	1.939982
H	1.354855	1.870833	1.898671
H	-0.168680	1.900455	1.026799
C	-0.372860	2.152050	3.150719
H	0.084896	1.761367	4.076291
H	-1.427971	1.829733	3.172723
C	-0.303550	3.673177	3.138678
H	-0.758172	4.083402	2.222931
H	0.738939	4.028065	3.177353
H	-0.835187	4.108688	3.998125
C	0.522316	-2.727170	0.973113
C	0.445279	-3.653335	-0.266194

H	-0.429889	-3.376736	-0.875884
H	1.332957	-3.534956	-0.902712
C	0.319176	-5.123311	0.160241
H	0.291142	-5.749075	-0.745431
C	-0.976346	-5.307233	0.958596
H	-1.845870	-5.043618	0.334439
H	-1.097401	-6.362526	1.254327
C	-0.927149	-4.412987	2.202263
H	-1.861359	-4.519756	2.775474
C	0.268394	-4.812102	3.077159
H	0.170066	-5.860919	3.402570
H	0.294710	-4.193692	3.990005
C	1.564046	-4.628870	2.277159
H	2.429561	-4.904831	2.900299
C	1.715680	-3.157283	1.853084
H	2.662438	-3.045349	1.305484
H	1.780940	-2.517727	2.748416
C	-0.780411	-2.940807	1.788069
H	-0.764790	-2.328757	2.701541
H	-1.653171	-2.618560	1.199426
C	1.524782	-5.515599	1.024670
H	1.454616	-6.577316	1.313427
H	2.459446	-5.400957	0.449634
C	2.274930	-0.417646	-0.152612
C	3.257253	-0.171932	1.018688
H	2.847338	0.565335	1.721657
H	3.418296	-1.098679	1.586729
C	4.606413	0.343591	0.490258
H	5.276358	0.512128	1.348321
C	5.221359	-0.699129	-0.451102
H	5.394544	-1.644192	0.090472
H	6.202474	-0.352536	-0.815570
C	4.271816	-0.933325	-1.632611
H	4.696098	-1.695200	-2.305367
C	4.068178	0.379732	-2.397756
H	3.407519	0.216623	-3.265581
H	5.032179	0.742313	-2.790733
C	3.453648	1.420546	-1.454241
H	3.287757	2.364730	-1.996393
C	2.100304	0.905919	-0.943943
H	1.627519	1.673523	-0.315768
H	1.422907	0.737397	-1.797222
C	2.921071	-1.448545	-1.107368
H	3.086161	-2.402851	-0.588320
H	2.259809	-1.656716	-1.962709
C	4.393373	1.661648	-0.265847
H	5.359497	2.056060	-0.621111
H	3.964659	2.421637	0.408805
C	-2.329731	0.121867	-0.001590
O	-2.976532	-0.571136	0.737541
P	0.563261	-0.934102	0.422424
Br	-2.840552	-0.494156	-2.997953
Pd	-1.038087	-0.683729	-1.270592
H	0.305115	-1.619657	-2.385531
H	-0.234530	-1.419677	-2.898708
C	-2.782073	4.391269	0.143194
C	-2.889041	5.894305	0.212467
F	-3.563653	6.316727	1.291957
F	-1.672604	6.474416	0.258070
F	-3.512950	6.401944	-0.866995

int-7-d2-cl.log

SCF (RwB97XD) = -4786.35684884
E(SCF)+ZPE(0 K)= -4785.655414
H(298 K)= -4785.618292
G(298 K)= -4785.724093
Lowest Frequency = 12.6049cm-1

C	-2.506417	1.633405	0.002391
C	-1.872017	2.455452	-0.932931
H	-1.254914	2.008839	-1.714982
C	-2.022093	3.836831	-0.881684
H	-1.528611	4.477816	-1.613381
C	-3.467192	3.593823	1.056928
H	-4.086752	4.048644	1.831078
C	-3.309941	2.214141	0.990413
H	-3.810946	1.569504	1.714995
C	0.267204	-0.011596	1.987323
H	-0.746191	-0.315474	2.284584
H	0.945485	-0.400656	2.763294
C	0.316160	1.517401	1.951580
H	1.358508	1.870546	1.916348
H	-0.160993	1.894503	1.035883
C	-0.378829	2.145409	3.157693
H	0.075461	1.758268	4.086513
H	-1.432204	1.817156	3.173038
C	-0.319062	3.666839	3.143969
H	-0.774019	4.072383	2.226149
H	0.721103	4.028396	3.185881
H	-0.856572	4.099971	4.000963
C	0.526692	-2.727941	0.975224
C	0.443443	-3.650532	-0.266368
H	-0.434424	-3.371652	-0.871031
H	1.328021	-3.530719	-0.906916
C	0.318470	-5.121704	0.156268
H	0.286209	-5.744793	-0.751133
C	-0.973808	-5.307268	0.959498
H	-1.845738	-5.041369	0.339691
H	-1.094230	-6.363351	1.252769
C	-0.918889	-4.416568	2.205442
H	-1.850794	-4.524337	2.782223
C	0.279920	-4.819260	3.074242
H	0.182210	-5.869032	3.396841
H	0.310417	-4.203610	3.988836
C	1.572421	-4.634412	2.269484
H	2.440335	-4.912889	2.888208
C	1.723187	-3.161643	1.849185
H	2.667958	-3.048646	1.298396
H	1.792290	-2.524881	2.746220
C	-0.772685	-2.943268	1.795108
H	-0.752549	-2.334225	2.710474
H	-1.647602	-2.617800	1.211506
C	1.527439	-5.517272	1.014463
H	1.457917	-6.579862	1.300211
H	2.459722	-5.401308	0.435818
C	2.277325	-0.416940	-0.148922
C	3.263564	-0.174420	1.019648
H	2.856639	0.562955	1.724329
H	3.424718	-1.101911	1.586455
C	4.612070	0.339585	0.488020
H	5.285142	0.505223	1.344215
C	5.222247	-0.702287	-0.457418
H	5.395205	-1.648876	0.081566
H	6.202965	-0.356757	-0.824020
C	4.268752	-0.932260	-1.636572
H	4.689875	-1.693299	-2.312285
C	4.064620	0.382725	-2.398248
H	3.400859	0.222255	-3.264180
H	5.027854	0.744780	-2.793657
C	3.454715	1.422406	-1.450464
H	3.288456	2.367977	-1.990123
C	2.102361	0.908805	-0.936419
H	1.633418	1.675479	-0.304197
H	1.421195	0.743821	-1.787364
C	2.918921	-1.446443	-1.108224
H	3.084133	-2.402316	-0.592136
H	2.254373	-1.651454	-1.961822
C	4.399065	1.659492	-0.264895
H	5.364787	2.052687	-0.622714
H	3.974101	2.418980	0.412695
C	-2.323421	0.138620	0.022502
O	-2.957722	-0.556207	0.771669
P	0.566331	-0.932866	0.429604
Br	-2.849624	-0.488043	-2.978460
Pd	-1.042152	-0.674777	-1.255021
H	0.300774	-1.607040	-2.383404
H	-0.255061	-1.431700	-2.887524
C	-2.815488	4.395147	0.119900
Cl	-2.989084	6.120719	0.207937

int-7-d2-cn.log

SCF (RwB97XD) = -4418.94464333
E(SCF)+ZPE(0 K)= -4418.234898
H(298 K)= -4418.197264
G(298 K)= -4418.303866
Lowest Frequency = 14.8832cm-1

C	-2.494301	1.675511	0.009239
C	-1.862295	2.479612	-0.942891
H	-1.240645	2.019032	-1.712666
C	-2.024214	3.858804	-0.919190
H	-1.531987	4.486262	-1.663594
C	-3.472667	3.642646	1.024189
H	-4.098146	4.104128	1.789869
C	-3.304562	2.264712	0.986248
H	-3.799930	1.627311	1.720429
C	0.286311	-0.021072	2.008157
H	-0.721353	-0.333137	2.315750
H	0.976094	-0.404471	2.776503
C	0.322471	1.508036	1.968239
H	1.362115	1.869480	1.936500
H	-0.151315	1.878241	1.047606
C	-0.384947	2.135055	3.167489
H	0.065778	1.753925	4.100348
H	-1.435923	1.798653	3.177437
C	-0.335603	3.656742	3.149793
H	-0.785107	4.057089	2.226913
H	0.701460	4.025842	3.199156
H	-0.882994	4.088839	4.000928
C	0.532189	-2.733809	0.979246
C	0.444095	-3.649241	-0.267424
H	-0.435577	-3.366212	-0.867455
H	1.326659	-3.527149	-0.910145
C	0.318105	-5.122527	0.147525
H	0.282920	-5.740376	-0.763309
C	-0.972709	-5.310598	0.952481
H	-1.845707	-5.040660	0.335901
H	-1.093826	-6.367946	1.240636
C	-0.913915	-4.426485	2.202906
H	-1.844717	-4.535749	2.781117
C	0.286112	-4.835623	3.067064
H	0.187155	-5.886875	3.384223
H	0.319518	-4.224957	3.984875
C	1.577356	-4.648868	2.260679
H	2.445969	-4.932251	2.876082
C	1.729917	-3.174047	1.848475
H	2.673806	-3.058994	1.296532
H	1.801613	-2.542764	2.749107
C	-0.766233	-2.951156	1.800239
H	-0.743935	-2.347221	2.718857
H	-1.641685	-2.621479	1.219539
C	1.528384	-5.524486	1.000809
H	1.457827	-6.588523	1.280605
H	2.459553	-5.406691	0.420793
C	2.283395	-0.419372	-0.141778
C	3.275762	-0.181156	1.022477
H	2.874095	0.556735	1.729856
H	3.437130	-1.109857	1.587389
C	4.622687	0.330550	0.484615
H	5.300717	0.492455	1.337507
C	5.225233	-0.710449	-0.466651
H	5.398510	-1.658972	0.068777
H	6.204906	-0.366419	-0.837233
C	4.265284	-0.934829	-1.641619
H	4.681160	-1.694997	-2.321488
C	4.059666	0.382508	-2.398722
H	3.391041	0.225487	-3.261537
H	5.021340	0.743826	-2.798376
C	3.457226	1.420956	-1.444864
H	3.289982	2.368289	-1.981048
C	2.106615	0.909070	-0.924303
H	1.643916	1.675014	-0.286522
H	1.420067	0.748378	-1.771785
C	2.917304	-1.447583	-1.107531
H	3.083220	-2.405204	-0.595003
H	2.247324	-1.648491	-1.957999
C	4.408760	1.652784	-0.263972
H	5.373389	2.044307	-0.626350
H	3.989750	2.411853	0.417752
C	-2.301544	0.174935	0.057774
O	-2.928050	-0.496276	0.833915
P	0.576470	-0.935676	0.446166
Br	-2.868386	-0.467427	-2.925293
Pd	-1.042337	-0.661238	-1.224533
H	0.283341	-1.598658	-2.354415
H	-0.281537	-1.431257	-2.852584
C	-2.827506	4.442377	0.070429
C	-2.979217	5.873252	0.116975
N	-3.093248	7.017263	0.159719

int-7-d2-nme2.log

SCF (RwB97XD) = -4460.64197600
E(SCF)+ZPE(0 K)= -4459.856961
H(298 K)= -4459.816826
G(298 K)= -4459.928703
Lowest Frequency = 13.3461cm-1

C	-2.627039	1.441199	-0.020386
C	-1.991500	2.293660	-0.931154
H	-1.385160	1.864839	-1.732068
C	-2.113086	3.669572	-0.840734
H	-1.603024	4.288052	-1.577675
C	-3.550784	3.396848	1.090150
H	-4.166545	3.800264	1.892784
C	-3.416730	2.023637	0.981601
H	-3.925873	1.372988	1.696000
C	0.154787	-0.009192	1.937395
H	-0.856582	-0.324257	2.231031
H	0.833493	-0.384829	2.720207
C	0.183051	1.520577	1.902610
H	1.218445	1.888650	1.834603
H	-0.334098	1.892553	1.007562
C	-0.477477	2.133602	3.136040
H	0.007290	1.741789	4.047634
H	-1.528433	1.801146	3.179141
C	-0.426169	3.655660	3.133929
H	-0.908729	4.062324	2.231123
H	0.612944	4.022778	3.151081
H	-0.942971	4.078105	4.009265
C	0.523249	-2.730431	0.988589
C	0.465287	-3.688570	-0.227125
H	-0.428804	-3.460340	-0.829131
H	1.338155	-3.549512	-0.880211
C	0.404217	-5.152196	0.234286
H	0.387842	-5.800017	-0.656148
C	-0.871254	-5.368003	1.056681
H	-1.759484	-5.151833	0.440716
H	-0.946635	-6.420324	1.377639
C	-0.839178	-4.443653	2.278693
H	-1.760023	-4.574243	2.868542
C	0.383816	-4.774741	3.143990
H	0.331995	-5.818451	3.495968
H	0.399111	-4.133405	4.041201
C	1.659191	-4.559536	2.319716
H	2.544324	-4.786059	2.935604
C	1.745225	-3.093801	1.858817
H	2.677932	-2.958975	1.292828
H	1.799834	-2.430548	2.737623
C	-0.757740	-2.977121	1.828354
H	-0.753342	-2.343201	2.726972
H	-1.651016	-2.703286	1.245950
C	1.636790	-5.476734	1.089005
H	1.613455	-6.533295	1.403721
H	2.557619	-5.338697	0.496870
C	2.168009	-0.377768	-0.201807
C	3.156889	-0.082641	0.952324
H	2.728811	0.642161	1.657050

H	3.363431	-0.997523	1.524395
C	4.477505	0.480275	0.400811
H	5.151669	0.684742	1.247870
C	5.120719	-0.548213	-0.537315
H	5.338651	-1.478727	0.013304
H	6.082659	-0.167985	-0.919408
C	4.164811	-0.833884	-1.702498
H	4.609436	-1.586446	-2.372760
C	3.900927	0.461805	-2.479382
H	3.235156	0.264210	-3.336059
H	4.845148	0.856387	-2.889678
C	3.258615	1.489124	-1.539590
H	3.049618	2.419598	-2.090743
C	1.932803	0.927975	-1.007081
H	1.434087	1.683665	-0.384386
H	1.253256	0.720606	-1.849934
C	2.841455	-1.393955	-1.153314
H	3.048946	-2.335601	-0.626106
H	2.177698	-1.636301	-1.997260
C	4.203052	1.780446	-0.366374
H	5.148523	2.208995	-0.738066
H	3.752727	2.529962	0.306054
C	-2.449554	-0.033071	-0.027498
O	-3.071444	-0.772038	0.692270
P	0.479239	-0.949515	0.392469
Br	-2.927388	-0.696743	-3.054575
Pd	-1.135367	-0.810060	-1.301132
H	0.242109	-1.748528	-2.434825
H	-0.308507	-1.571925	-2.940627
C	-2.890916	4.264185	0.183880
N	-2.989850	5.621413	0.302355
C	-3.822502	6.202697	1.331641
H	-3.783198	7.295298	1.256066
H	-4.877814	5.896480	1.230310
H	-3.480496	5.923990	2.342996
C	-2.304511	6.484231	-0.633701
H	-2.479802	7.530243	-0.357950
H	-1.214695	6.314251	-0.622741
H	-2.663116	6.343669	-1.668026

int-7-d2-ome.log

SCF (RwB97XD) = -4441.23724867
E(SCF)+ZPE(0 K)= -4440.493382
H(298 K)= -4440.454919
G(298 K)= -4440.563045
Lowest Frequency = 13.1158cm-1

C	-2.508290	1.599210	-0.003168
C	-1.867231	2.437754	-0.926678
H	-1.258202	1.999208	-1.719792
C	-2.001347	3.812561	-0.847825
H	-1.506937	4.469197	-1.565601
C	-3.442062	3.561321	1.087826
H	-4.060071	3.979461	1.881776
C	-3.298895	2.180180	0.992094
H	-3.806011	1.531465	1.709329
C	0.248263	-0.005408	1.971596
H	-0.770254	-0.301370	2.259663
H	0.916893	-0.401535	2.752646
C	0.309843	1.523344	1.943112
H	1.354619	1.868569	1.903244
H	-0.172826	1.909158	1.034181
C	-0.370786	2.149038	3.158748
H	0.081986	1.747460	4.082313
H	-1.428747	1.836363	3.174507
C	-0.288888	3.669609	3.158687
H	-0.734317	4.087139	2.241656
H	0.756266	4.015861	3.206602
H	-0.822594	4.102946	4.018363
C	0.519882	-2.722969	0.971576
C	0.440822	-3.651316	-0.265899
H	-0.436126	-3.376401	-0.873654
H	1.326521	-3.532327	-0.905207
C	0.318131	-5.120945	0.162630
H	0.288555	-5.748132	-0.742079
C	-0.975043	-5.305701	0.964642
H	-1.846372	-5.043612	0.342409
H	-1.094027	-6.360902	1.261933
C	-0.923785	-4.409762	2.207012
H	-1.856400	-4.517287	2.782794
C	0.274499	-4.806483	3.079269
H	0.178676	-5.855171	3.406163
H	0.302352	-4.186886	3.991316
C	1.567765	-4.622041	2.275658
H	2.435409	-4.896012	2.896892
C	1.715838	-3.150832	1.848888
H	2.661055	-3.038812	1.298710
H	1.782853	-2.509704	2.743040
C	-0.779967	-2.937932	1.790704
H	-0.761770	-2.324961	2.703565
H	-1.654713	-2.615903	1.205025
C	1.526477	-5.510581	1.024483
H	1.458723	-6.572108	1.314861
H	2.459460	-5.395183	0.446800
C	2.267796	-0.413164	-0.154375
C	3.249996	-0.168150	1.017036
H	2.839005	0.567121	1.721371
H	3.412394	-1.095478	1.583644
C	4.598859	0.349600	0.489983
H	5.268601	0.517774	1.348410
C	5.215196	-0.691414	-0.452442
H	5.389063	-1.636904	0.088242
H	6.196174	-0.343506	-0.816304
C	4.266059	-0.925334	-1.634387
H	4.691433	-1.686036	-2.307896
C	4.061715	0.388469	-2.398133
H	3.401265	0.225754	-3.266153
H	5.025659	0.752190	-2.790498
C	3.445766	1.427640	-1.453683
H	3.279191	2.372237	-1.994975
C	2.092868	0.911040	-0.944497
H	1.618325	1.677365	-0.316122
H	1.415611	0.742746	-1.797830
C	2.915499	-1.442122	-1.110124
H	3.081139	-2.397131	-0.592441
H	2.254557	-1.649689	-1.965770
C	4.384974	1.668322	-0.264779
H	5.351009	2.064151	-0.619059
H	3.954956	2.426983	0.410594
C	-2.341618	0.112952	-0.003493
O	-2.979480	-0.602495	0.724473
P	0.553516	-0.930146	0.415526
Br	-2.840094	-0.507052	-3.022406
Pd	-1.047109	-0.687490	-1.279554
H	0.309497	-1.620755	-2.413972
H	-0.242488	-1.442097	-2.919296
C	-2.785329	4.386992	0.166021
O	-2.841179	5.734721	0.176074
C	-3.599936	6.375477	1.182339
H	-3.221267	6.135526	2.189609
H	-3.493026	7.453315	1.008493
H	-4.667451	6.108121	1.118503

int-7-h.log

SCF (RwB97XD) = -4326.74224359
E(SCF)+ZPE(0 K)= -4326.026464
H(298 K)= -4325.991257
G(298 K)= -4326.091817
Lowest Frequency = 17.8462cm-1

C	-2.497924	1.653220	0.001927
C	-1.861024	2.459583	-0.946232
H	-1.238835	1.997399	-1.715351
C	-2.026524	3.841519	-0.916990
H	-1.530597	4.463918	-1.664778
C	-3.471422	3.628050	1.009247
H	-4.101839	4.084852	1.775245
C	-3.308166	2.246189	0.978324

H	-3.806222	1.608524	1.711297
C	0.271542	-0.021303	2.000131
H	-0.740566	-0.327573	2.299120
H	0.953354	-0.412518	2.771958
C	0.317127	1.507840	1.970204
H	1.358773	1.863226	1.933302
H	-0.164585	1.887997	1.058047
C	-0.375536	2.128180	3.181569
H	0.078632	1.733193	4.107267
H	-1.429762	1.802656	3.194171
C	-0.312799	3.649470	3.178896
H	-0.772697	4.060722	2.266150
H	0.728466	4.008551	3.218408
H	-0.845086	4.076551	4.042325
C	0.528941	-2.732206	0.977034
C	0.444351	-3.651958	-0.266592
H	-0.435447	-3.373046	-0.868250
H	1.327054	-3.529144	-0.909131
C	0.322523	-5.124442	0.152419
H	0.289556	-5.745220	-0.756571
C	-0.968118	-5.314026	0.957334
H	-1.841446	-5.047885	0.339616
H	-1.086460	-6.371024	1.248291
C	-0.912437	-4.426163	2.205268
H	-1.843217	-4.536710	2.783370
C	0.288377	-4.829422	3.071104
H	0.192591	-5.880133	3.391326
H	0.319561	-4.215893	3.987114
C	1.579363	-4.640884	2.264708
H	2.448640	-4.919876	2.881347
C	1.727415	-3.166909	1.847719
H	2.671016	-3.051365	1.295413
H	1.797562	-2.532272	2.746180
C	-0.768848	-2.951645	1.798435
H	-0.748322	-2.345010	2.715323
H	-1.644948	-2.625355	1.216965
C	1.533462	-5.520613	1.007527
H	1.465894	-6.584076	1.290601
H	2.464593	-5.401788	0.427575
C	2.274361	-0.416427	-0.144413
C	3.264696	-0.176418	1.021084
H	2.859662	0.558820	1.729113
H	3.428560	-1.105236	1.584990
C	4.610954	0.339899	0.486003
H	5.287280	0.503514	1.340055
C	5.218261	-0.698885	-0.464723
H	5.393585	-1.646989	0.070851
H	6.197533	-0.351810	-0.833833
C	4.260576	-0.925978	-1.641074
H	4.679839	-1.684744	-2.320548
C	4.052768	0.391105	-2.398140
H	3.385825	0.232544	-3.261954
H	5.014300	0.754978	-2.796149
C	3.445576	1.427562	-1.445043
H	3.276585	2.374544	-1.981399
C	2.095609	0.911442	-0.927372
H	1.627884	1.675703	-0.291297
H	1.411215	0.748341	-1.776022
C	2.913057	-1.442506	-1.109218
H	3.080864	-2.399858	-0.596659
H	2.245009	-1.645154	-1.960653
C	4.394402	1.661816	-0.262450
H	5.358642	2.056543	-0.622754
H	3.971469	2.419078	0.418904
C	-2.316355	0.158782	0.043554
O	-2.940966	-0.528595	0.807312
P	0.564977	-0.934755	0.437061
Br	-2.871631	-0.512976	-2.953149
Pd	-1.050916	-0.674699	-1.238659
H	0.286836	-1.615782	-2.377387
H	-0.275214	-1.448637	-2.876444
C	-2.829503	4.427436	0.062209
H	-2.958119	5.512091	0.085875

int-7-me.log

SCF (RwB97XD) = -4366.02269061
E(SCF)+ZPE(0 K)= -4365.279773
H(298 K)= -4365.242479
G(298 K)= -4365.348505
Lowest Frequency = 14.1966cm-1

C	-2.505516	1.597195	-0.019237
C	-1.865411	2.436232	-0.937396
H	-1.257053	2.000118	-1.732406
C	-2.001798	3.815467	-0.843543
H	-1.494668	4.456707	-1.569344
C	-3.425385	3.550821	1.076309
H	-4.036410	3.981886	1.873542
C	-3.294282	2.168818	0.984767
H	-3.798271	1.515827	1.700172
C	0.248285	0.002597	1.957906
H	-0.771585	-0.290024	2.244739
H	0.913700	-0.392173	2.742405
C	0.313309	1.531059	1.924845
H	1.358620	1.873611	1.877094
H	-0.174296	1.915351	1.017925
C	-0.356709	2.161349	3.144051
H	0.107245	1.765636	4.064611
H	-1.413624	1.846201	3.172921
C	-0.279924	3.682153	3.135191
H	-0.749729	4.093438	2.227565
H	0.765086	4.031677	3.158167
H	-0.795711	4.117252	4.004675
C	0.517988	-2.719629	0.969521
C	0.441029	-3.652380	-0.264710
H	-0.432982	-3.377549	-0.876804
H	1.329474	-3.538017	-0.901099
C	0.313255	-5.120060	0.169009
H	0.285133	-5.750659	-0.733362
C	-0.982927	-5.298678	0.967588
H	-1.851654	-5.036825	0.341630
H	-1.105400	-6.352478	1.268361
C	-0.933499	-4.398291	2.206788
H	-1.868177	-4.501372	2.780005
C	0.261104	-4.794311	3.084360
H	0.161842	-5.841468	3.415078
H	0.287512	-4.171250	3.994067
C	1.557287	-4.615989	2.284138
H	2.422308	-4.889548	2.909178
C	1.710230	-3.146782	1.852291
H	2.657465	-3.039079	1.304703
H	1.775899	-2.502451	2.744223
C	-0.784988	-2.928405	1.785277
H	-0.768403	-2.311914	2.695797
H	-1.657214	-2.607258	1.195389
C	1.517886	-5.509261	1.036284
H	1.446631	-6.569467	1.330608
H	2.453041	-5.398408	0.461230
C	2.275138	-0.417704	-0.158503
C	3.252980	-0.170177	1.016075
H	2.840533	0.568019	1.716474
H	3.411546	-1.095932	1.586308
C	4.604821	0.343527	0.492674
H	5.271204	0.513822	1.353284
C	5.223430	-0.701833	-0.443390
H	5.393819	-1.645492	0.101553
H	6.206324	-0.356668	-0.804671
C	4.278675	-0.938817	-1.628204
H	4.705458	-1.702680	-2.297204
C	4.079618	0.372445	-2.397689
H	3.422379	0.207712	-3.267785
H	5.045781	0.733021	-2.787478
C	3.461576	1.416188	-1.459680
H	3.298962	2.359047	-2.005274
C	2.105633	0.903740	-0.954426
H	1.629254	1.673005	-0.331051
H	1.431983	0.733282	-1.810163
C	2.925149	-1.451466	-1.107503
H	3.087068	-2.404704	-0.585422

H	2.267708	-1.661473	-1.965193
C	4.396125	1.659818	-0.267721
H	5.364231	2.052901	-0.619360
H	3.964425	2.421488	0.403200
C	-2.338207	0.105421	-0.030902
O	-2.986625	-0.611445	0.684704
P	0.557703	-0.929234	0.406853
Br	-2.812430	-0.499895	-3.053739
Pd	-1.032587	-0.687284	-1.298332
H	0.325916	-1.624910	-2.421901
H	-0.214098	-1.428665	-2.933942
C	-2.779664	4.397998	0.167603
C	-2.916954	5.892943	0.259842
H	-3.554106	6.276872	-0.553307
H	-3.369664	6.201235	1.212668
H	-1.939228	6.389699	0.167031

int-7-nme2.log

SCF (RwB97XD) = -4460.64197600
E(SCF)+ZPE(0 K)= -4459.852550
H(298 K)= -4459.812965
G(298 K)= -4459.923700
Lowest Frequency = 13.3536cm-1

C	-2.627035	1.441207	-0.020376
C	-1.991493	2.293669	-0.931140
H	-1.385143	1.864850	-1.732048
C	-2.113086	3.669580	-0.840723
H	-1.603020	4.288061	-1.577662
C	-3.550799	3.396854	1.090148
H	-4.166567	3.800269	1.892778
C	-3.416736	2.023643	0.981605
H	-3.925881	1.372994	1.696001
C	0.154799	-0.009195	1.937400
H	-0.856569	-0.324260	2.231036
H	0.833506	-0.384832	2.720213
C	0.183063	1.520573	1.902613
H	1.218457	1.888648	1.834606
H	-0.334086	1.892548	1.007564
C	-0.477466	2.133601	3.136042
H	0.007304	1.741795	4.047636
H	-1.528421	1.801140	3.179145
C	-0.426166	3.655659	3.133923
H	-0.908730	4.062316	2.231115
H	0.612945	4.022783	3.151071
H	-0.942970	4.078107	4.009257
C	0.523253	-2.730434	0.988587
C	0.465279	-3.688570	-0.227127
H	-0.428813	-3.460334	-0.829129
H	1.338146	-3.549517	-0.880217
C	0.404202	-5.152197	0.234282
H	0.387820	-5.800017	-0.656152
C	-0.871265	-5.367997	1.056683
H	-1.759497	-5.151823	0.440724
H	-0.946650	-6.420320	1.377640
C	-0.839178	-4.443650	2.278697
H	-1.760020	-4.574235	2.868550
C	0.383819	-4.774746	3.143987
H	0.331995	-5.818455	3.495964
H	0.399122	-4.133410	4.041199
C	1.659192	-4.559546	2.319707
H	2.544327	-4.786075	2.935590
C	1.745231	-3.093811	1.858809
H	2.677936	-2.958988	1.292816
H	1.799847	-2.430559	2.737615
C	-0.757734	-2.977118	1.828359
H	-0.753329	-2.343198	2.726977
H	-1.651011	-2.703277	1.245959
C	1.636779	-5.476743	1.088995
H	1.613440	-6.533303	1.403709
H	2.557606	-5.338709	0.496855
C	2.168019	-0.377771	-0.201803
C	3.156899	-0.082645	0.952327
H	2.728824	0.642158	1.657053
H	3.363442	-0.997528	1.524398
C	4.477516	0.480269	0.400812
H	5.151682	0.684736	1.247870
C	5.120728	-0.548219	-0.537315
H	5.338660	-1.478733	0.013303
H	6.082668	-0.167991	-0.919411
C	4.164818	-0.833891	-1.702498
H	4.609441	-1.586452	-2.372760
C	3.900933	0.461799	-2.479382
H	3.235162	0.264204	-3.336057
H	4.845155	0.856379	-2.889679
C	3.258624	1.489119	-1.539589
H	3.049627	2.419593	-2.090743
C	1.932812	0.927970	-1.007079
H	1.434097	1.683661	-0.384385
H	1.253266	0.720601	-1.849931
C	2.841463	-1.393961	-1.153309
H	3.048958	-2.335603	-0.626096
H	2.177701	-1.636314	-1.997248
C	4.203063	1.780440	-0.366374
H	5.148534	2.208988	-0.738067
H	3.752739	2.529956	0.306053
C	-2.449547	-0.033063	-0.027484
O	-3.071437	-0.772029	0.692286
P	0.479248	-0.949516	0.392473
Br	-2.927390	-0.696723	-3.054562
Pd	-1.135366	-0.810050	-1.301126
H	0.242111	-1.748510	-2.434826
H	-0.308500	-1.571892	-2.940628
C	-2.890929	4.264192	0.183882
N	-2.989873	5.621420	0.302351
C	-3.822540	6.202702	1.331625
H	-3.783248	7.295301	1.256044
H	-4.877847	5.896471	1.230286
H	-3.480541	5.924004	2.342985
C	-2.304533	6.484239	-0.633704
H	-2.479835	7.530251	-0.357960
H	-1.214716	6.314267	-0.622733
H	-2.663126	6.343668	-1.668032

int-7-ome.log

SCF (RwB97XD) = -4441.23724867
E(SCF)+ZPE(0 K)= -4440.489012
H(298 K)= -4440.451060
G(298 K)= -4440.558042
Lowest Frequency = 13.1237cm-1

C	-2.508286	1.599216	-0.003167
C	-1.867223	2.437754	-0.926679
H	-1.258193	1.999203	-1.719788
C	-2.001337	3.812561	-0.847834
H	-1.506923	4.469192	-1.565611
C	-3.442059	3.561335	1.087814
H	-4.060071	3.979479	1.881760
C	-3.298894	2.180193	0.992090
H	-3.806014	1.531482	1.709327
C	0.248264	-0.005415	1.971602
H	-0.770252	-0.301378	2.259669
H	0.916895	-0.401543	2.752650
C	0.309843	1.523338	1.943123
H	1.354618	1.868565	1.903260
H	-0.172822	1.909154	1.034190
C	-0.370794	2.149028	3.158756
H	0.081974	1.747450	4.082322
H	-1.428755	1.836349	3.174508
C	-0.288903	3.669599	3.158697
H	-0.734334	4.087129	2.241667
H	0.756250	4.015856	3.206613
H	-0.822610	4.102933	4.018373
C	0.519886	-2.722973	0.971578
C	0.440819	-3.651318	-0.265898
H	-0.436133	-3.376402	-0.873648
H	1.326514	-3.532329	-0.905210
C	0.318127	-5.120947	0.162629

H	0.288546	-5.748133	-0.742081
C	-0.975042	-5.305703	0.964648
H	-1.846374	-5.043613	0.342420
H	-1.094025	-6.360904	1.261938
C	-0.923777	-4.409767	2.207019
H	-1.856390	-4.517291	2.782806
C	0.274511	-4.806489	3.079269
H	0.178689	-5.855179	3.406162
H	0.302369	-4.186895	3.991317
C	1.567773	-4.622047	2.275652
H	2.435421	-4.896021	2.896880
C	1.715846	-3.150839	1.848883
H	2.661060	-3.038819	1.298700
H	1.782866	-2.509712	2.743035
C	-0.779960	-2.937936	1.790713
H	-0.761757	-2.324967	2.703574
H	-1.654708	-2.615906	1.205038
C	1.526477	-5.510586	1.024475
H	1.458724	-6.572113	1.314853
H	2.459458	-5.395188	0.446787
C	2.267795	-0.413165	-0.154373
C	3.249998	-0.168157	1.017036
H	2.839008	0.567109	1.721377
H	3.412397	-1.095488	1.583638
C	4.598859	0.349597	0.489982
H	5.268604	0.517767	1.348409
C	5.215193	-0.691411	-0.452450
H	5.389064	-1.636905	0.088228
H	6.196170	-0.343500	-0.816313
C	4.266053	-0.925326	-1.634394
H	4.691426	-1.686024	-2.307908
C	4.061707	0.388481	-2.398132
H	3.401253	0.225771	-3.266151
H	5.025649	0.752204	-2.790499
C	3.445759	1.427647	-1.453674
H	3.279183	2.372247	-1.994961
C	2.092864	0.911044	-0.944487
H	1.618322	1.677365	-0.316106
H	1.415604	0.742755	-1.797820
C	2.915496	-1.442118	-1.110129
H	3.081138	-2.397128	-0.592451
H	2.254551	-1.649683	-1.965774
C	4.384971	1.668323	-0.264771
H	5.351005	2.064153	-0.619053
H	3.954954	2.426980	0.410606
C	-2.341615	0.112959	-0.003482
O	-2.979473	-0.602484	0.724492
P	0.553517	-0.930149	0.415530
Br	-2.840096	-0.507034	-3.022398
Pd	-1.047114	-0.687492	-1.279546
H	0.309491	-1.620761	-2.413966
H	-0.242498	-1.442104	-2.919286
C	-2.785322	4.386999	0.166007
O	-2.841169	5.734729	0.176053
C	-3.599931	6.375490	1.182310
H	-3.221271	6.135542	2.189585
H	-3.493016	7.453327	1.008462
H	-4.667446	6.108138	1.118467

int-7prime-cf3.log

SCF (RwB97XD) = -4838.18108585
E(SCF)+ZPE(0 K)= -4837.332594
H(298 K)= -4837.287645
G(298 K)= -4837.412951
Lowest Frequency = 11.5413cm-1

C	-2.432159	1.614112	0.326277
C	-1.744471	2.619670	-0.361218
H	-0.939286	2.342530	-1.045386
C	-2.078935	3.956454	-0.183014
H	-1.539185	4.734635	-0.727095
C	-3.800255	3.309061	1.394616
H	-4.599886	3.577739	2.086380
C	-3.461828	1.972543	1.199916
H	-3.994527	1.181345	1.731756
C	0.284584	-0.087110	2.472500
H	-0.739300	-0.471645	2.575220
H	0.862204	-0.500309	3.314763
C	0.220707	1.438490	2.550386
H	1.224063	1.862765	2.714155
H	-0.128225	1.845307	1.589785
C	-0.713354	1.928581	3.654544
H	-0.370160	1.538264	4.628913
H	-1.715853	1.498789	3.487766
C	-0.815587	3.446980	3.705556
H	-1.172855	3.848386	2.744195
H	0.162155	3.910407	3.915953
H	-1.516817	3.780416	4.485595
C	0.807509	-2.667017	1.255397
C	0.992260	-3.463672	-0.058163
H	0.237544	-3.125305	-0.784970
H	1.975649	-3.253632	-0.499193
C	0.848002	-4.972017	0.185841
H	1.010219	-5.503959	-0.766811
C	-0.563427	-5.263398	0.707367
H	-1.314540	-4.946194	-0.034982
H	-0.699017	-6.346521	0.865332
C	-0.773661	-4.504045	2.021856
H	-1.791469	-4.689907	2.398904
C	0.257616	-4.969630	3.058051
H	0.143518	-6.048551	3.256364
H	0.093357	-4.447986	4.015643
C	1.668888	-4.676393	2.533215
H	2.416876	-5.000003	3.274723
C	1.838621	-3.165650	2.290872
H	2.864108	-2.980463	1.939478
H	1.719616	-2.622342	3.242225
C	-0.610196	-2.994077	1.793085
H	-0.779063	-2.486524	2.753762
H	-1.377337	-2.617608	1.098445
C	1.890593	-5.430388	1.214654
H	1.809512	-6.517523	1.380706
H	2.909024	-5.238657	0.835560
C	2.641984	-0.221069	0.706498
C	3.393005	-0.074665	2.051585
H	2.847834	0.597401	2.728897
H	3.471628	-1.045494	2.561059
C	4.803079	0.496506	1.820146
H	5.309491	0.585506	2.794527
C	5.600614	-0.443473	0.907137
H	5.698849	-1.436436	1.377797
H	6.622391	-0.055243	0.760572
C	4.879702	-0.568585	-0.441766
H	5.438378	-1.256313	-1.097711
C	4.776873	0.810877	-1.103277
H	4.273683	0.726669	-2.081346
H	5.783773	1.220126	-1.290971
C	3.981626	1.747503	-0.186148
H	3.888636	2.738896	-0.657064
C	2.574627	1.173228	0.028848
H	1.984341	1.870359	0.639768
H	2.054670	1.079040	-0.937046
C	3.473738	-1.143945	-0.211813
H	3.575599	-2.141135	0.239671
H	2.949906	-1.267588	-1.171766
C	4.697028	1.879710	1.164417
H	5.702010	2.311543	1.023617
H	4.140241	2.569175	1.820783
C	-2.045248	0.142911	0.178426
O	-2.776854	-0.679179	0.703076
P	0.869093	-0.821173	0.894576
Br	-1.846503	-0.009343	-2.998892
Pd	-0.409723	-0.385254	-0.936616
H	0.680992	-1.631373	-3.004196
H	0.929323	-0.845090	-1.830887
N	0.837681	-2.256224	-3.853871
C	0.851243	-1.374267	-5.040416
H	-0.130346	-0.895968	-5.129512
H	1.615233	-0.601227	-4.893659

H	1.083515	-1.969128	-5.933785
C	-0.266652	-3.237305	-3.894026
H	-0.253489	-3.823660	-2.967657
H	-1.213446	-2.688627	-3.959436
H	-0.136212	-3.899053	-4.760584
C	2.149256	-2.899449	-3.637543
H	2.916884	-2.120911	-3.551291
H	2.113810	-3.478122	-2.707927
H	2.380466	-3.559737	-4.483243
C	-3.107311	4.300658	0.699593
C	-3.418352	5.759317	0.905909
F	-2.371335	6.421217	1.445864
F	-4.464238	5.960982	1.723047
F	-3.700572	6.386375	-0.253351

int-7prime-cl.log

SCF (RwB97XD) = -4960.73056151
E(SCF)+ZPE(0 K)= -4959.896460
H(298 K)= -4959.853975
G(298 K)= -4959.972326
Lowest Frequency = 12.7603cm-1

C	-2.586507	1.894286	0.363330
C	-1.899160	2.902154	-0.318630
H	-1.087564	2.628758	-0.997264
C	-2.235434	4.242339	-0.148829
H	-1.700164	5.025847	-0.687200
C	-3.972111	3.588231	1.418649
H	-4.777421	3.867162	2.100250
C	-3.625698	2.254159	1.227968
H	-4.161605	1.463460	1.757912
C	0.132409	0.210263	2.511089
H	-0.890136	-0.177848	2.613640
H	0.711567	-0.201122	3.353238
C	0.062891	1.735670	2.589601
H	1.064316	2.163619	2.755993
H	-0.286253	2.141591	1.628875
C	-0.875695	2.221180	3.691941
H	-0.534333	1.830077	4.666709
H	-1.876503	1.789192	3.521021
C	-0.982580	3.739173	3.745540
H	-1.340559	4.140374	2.784177
H	-0.006337	4.204994	3.958062
H	-1.686004	4.068815	4.525195
C	0.669802	-2.367844	1.299294
C	0.860861	-3.166664	-0.011834
H	0.104053	-2.835239	-0.739589
H	1.842903	-2.950918	-0.452862
C	0.726873	-4.675228	0.235701
H	0.893730	-5.208141	-0.715699
C	-0.682897	-4.975096	0.757032
H	-1.435861	-4.665181	0.013468
H	-0.811047	-6.058725	0.917882
C	-0.899333	-4.213596	2.069338
H	-1.916141	-4.405501	2.446087
C	0.134316	-4.669529	3.107438
H	0.027277	-5.748671	3.308591
H	-0.034109	-4.146421	4.063507
C	1.544025	-4.368170	2.582816
H	2.293601	-4.684889	3.325728
C	1.703735	-2.856937	2.336501
H	2.728088	-2.665800	1.984978
H	1.580785	-2.311854	3.286332
C	-0.745929	-2.703149	1.836870
H	-0.918926	-2.194187	2.796069
H	-1.515079	-2.333062	1.141030
C	1.771765	-5.124070	1.266376
H	1.697796	-6.211325	1.435010
H	2.789147	-4.926382	0.887545
C	2.488512	0.087298	0.742853
C	3.239366	0.241173	2.087219
H	2.690143	0.910465	2.763871
H	3.324769	-0.728291	2.598197
C	4.645477	0.821304	1.854294
H	5.151559	0.915629	2.828373
C	5.448977	-0.115208	0.942995
H	5.554000	-1.106471	1.415785
H	6.468087	0.279549	0.795168
C	4.728340	-0.247972	-0.405316
H	5.291250	-0.933385	-1.060053
C	4.616399	1.129416	-1.069676
H	4.113657	1.039920	-2.047446
H	5.620615	1.544903	-1.258193
C	3.815127	2.062654	-0.154377
H	3.715627	3.052462	-0.627339
C	2.411974	1.479453	0.061820
H	1.816449	2.173966	0.670689
H	1.893197	1.379012	-0.904113
C	3.326186	-0.832081	-0.173691
H	3.434926	-1.827563	0.279967
H	2.803032	-0.961226	-1.133285
C	4.529943	2.202410	1.195770
H	5.532000	2.640709	1.053827
H	3.968721	2.889403	1.850967
C	-2.196200	0.428747	0.220366
O	-2.918021	-0.399050	0.750902
P	0.718737	-0.522459	0.933023
Br	-2.010716	0.270803	-2.957167
Pd	-0.563622	-0.099204	-0.899835
H	0.519661	-1.380994	-2.942272
H	0.774797	-0.561594	-1.800315
N	0.661061	-2.013133	-3.791146
C	0.681816	-1.132051	-4.978159
H	-0.290601	-0.633523	-5.057299
H	1.462993	-0.374943	-4.838767
H	0.893220	-1.731556	-5.873617
C	-0.459515	-2.975669	-3.824620
H	-0.451234	-3.562047	-2.898207
H	-1.397373	-2.411106	-3.884897
H	-0.345153	-3.640119	-4.691473
C	1.963169	-2.677092	-3.582706
H	2.741895	-1.910329	-3.489358
H	1.920446	-3.265468	-2.659487
H	2.184491	-3.331810	-4.435402
C	-3.269490	4.572981	0.725021
Cl	-3.681399	6.247905	0.966584

int-7prime-cn.log

SCF (RwB97XD) = -4593.32042615
E(SCF)+ZPE(0 K)= -4592.478047
H(298 K)= -4592.434915
G(298 K)= -4592.554639
Lowest Frequency = 14.9300cm-1

C	-2.630210	1.982604	0.373424
C	-1.931501	2.981757	-0.310510
H	-1.102379	2.699714	-0.963378
C	-2.283686	4.318985	-0.172462
H	-1.739361	5.095561	-0.712396
C	-4.054733	3.675153	1.366050
H	-4.880304	3.954112	2.023304
C	-3.692602	2.343449	1.209150
H	-4.231024	1.554339	1.738026
C	0.106084	0.255661	2.536547
H	-0.911862	-0.142392	2.644667
H	0.693437	-0.148264	3.376398
C	0.022067	1.780670	2.608974
H	1.017151	2.217426	2.790765
H	-0.311644	2.180574	1.639957
C	-0.940894	2.264009	3.691086
H	-0.610923	1.886660	4.674966
H	-1.933288	1.817273	3.508618
C	-1.067987	3.780831	3.727402
H	-1.415007	4.167704	2.755992
H	-0.101495	4.261668	3.950117
H	-1.787675	4.109797	4.492233
C	0.638135	-2.317660	1.309297
C	0.824965	-3.108929	-0.007070

H	0.067211	-2.772156	-0.731396
H	1.806538	-2.892245	-0.448738
C	0.688772	-4.618706	0.231872
H	0.852562	-5.146228	-0.723004
C	-0.720450	-4.919074	0.754394
H	-1.474464	-4.603524	0.014231
H	-0.850294	-6.003326	0.909202
C	-0.932659	-4.165072	2.071638
H	-1.948899	-4.357362	2.449648
C	0.102534	-4.628779	3.104782
H	-0.005978	-5.708857	3.299705
H	-0.062971	-4.111117	4.064289
C	1.511646	-4.326847	2.578919
H	2.262252	-4.649234	3.318262
C	1.673534	-2.814423	2.341441
H	2.697449	-2.622738	1.988857
H	1.553336	-2.274876	3.294752
C	-0.777090	-2.653497	1.848177
H	-0.946914	-2.150530	2.811014
H	-1.547284	-2.277684	1.156525
C	1.735153	-5.075360	1.257555
H	1.659619	-6.163399	1.420101
H	2.752018	-4.877354	0.877571
C	2.458545	0.140556	0.762631
C	3.211735	0.287990	2.106288
H	2.664175	0.956006	2.785834
H	3.296338	-0.683493	2.613649
C	4.618172	0.867140	1.872935
H	5.126538	0.956024	2.846279
C	5.418295	-0.066044	0.955224
H	5.522791	-1.059842	1.422735
H	6.437658	0.327898	0.807390
C	4.694990	-0.190908	-0.392406
H	5.255834	-0.873503	-1.051775
C	4.582977	1.189985	-1.049401
H	4.078274	1.106028	-2.026678
H	5.587102	1.605536	-1.237861
C	3.784699	2.119315	-0.127557
H	3.684910	3.111530	-0.595318
C	2.381438	1.536392	0.089213
H	1.788845	2.228219	0.704002
H	1.860060	1.441908	-0.875961
C	3.292744	-0.774714	-0.160948
H	3.401046	-1.772944	0.286664
H	2.767039	-0.897459	-1.120098
C	4.502843	2.251610	1.221525
H	5.505006	2.689373	1.079211
H	3.944113	2.936125	1.881428
C	-2.227028	0.510205	0.258284
O	-2.948474	-0.303036	0.810149
P	0.690368	-0.470519	0.955453
Br	-2.050467	0.345263	-2.918571
Pd	-0.600644	-0.030523	-0.865724
H	0.463613	-1.301610	-2.936508
H	0.728694	-0.500413	-1.765636
N	0.609458	-1.925726	-3.786834
C	0.624979	-1.039746	-4.970759
H	-0.350754	-0.547922	-5.049850
H	1.400880	-0.277914	-4.828108
H	0.841373	-1.635009	-5.867745
C	-0.504880	-2.895884	-3.824830
H	-0.493213	-3.485669	-2.900636
H	-1.446548	-2.337887	-3.884376
H	-0.384708	-3.555841	-4.694190
C	1.915918	-2.583296	-3.581796
H	2.690657	-1.812733	-3.487510
H	1.877433	-3.174846	-2.660482
H	2.139001	-3.233966	-4.437033
C	-3.349004	4.668130	0.669947
C	-3.708440	6.051617	0.834540
N	-3.991070	7.159148	0.970723

int-7prime-h.log

SCF (RwB97XD) = -4501.11447799

E(SCF)+ZPE(0 K)= -4500.270934
H(298 K)= -4500.229659
G(298 K)= -4500.344449
Lowest Frequency = 19.7238cm-1

C	-2.757397	2.207246	0.394471
C	-2.047280	3.207076	-0.277362
H	-1.209496	2.922639	-0.918969
C	-2.404428	4.547082	-0.140220
H	-1.845727	5.317826	-0.676442
C	-4.190525	3.911951	1.356990
H	-5.029741	4.187128	2.000579
C	-3.832353	2.573740	1.212613
H	-4.381609	1.785900	1.733344
C	-0.045041	0.496773	2.570293
H	-1.064273	0.099711	2.670183
H	0.537169	0.087761	3.411501
C	-0.129315	2.021208	2.652326
H	0.866349	2.457876	2.831673
H	-0.471957	2.427085	1.689325
C	-1.086086	2.494232	3.744354
H	-0.753191	2.103708	4.722413
H	-2.080662	2.054082	3.558740
C	-1.208693	4.010839	3.799300
H	-1.567889	4.407431	2.836390
H	-0.238037	4.486340	4.016766
H	-1.918758	4.331480	4.576868
C	0.507858	-2.072154	1.351083
C	0.708107	-2.868061	0.039697
H	-0.048367	-2.540491	-0.690000
H	1.690107	-2.645379	-0.397937
C	0.582959	-4.377819	0.284610
H	0.756388	-4.908447	-0.666964
C	-0.826603	-4.687486	0.800965
H	-1.579252	-4.381640	0.055406
H	-0.948317	-5.772135	0.960264
C	-1.052242	-3.928849	2.113353
H	-2.069037	-4.127724	2.486559
C	-0.019086	-4.379722	3.154237
H	-0.120144	-5.459775	3.353781
H	-0.193855	-3.858812	4.110363
C	1.390544	-4.069025	2.634655
H	2.139538	-4.382190	3.379712
C	1.541666	-2.556516	2.390676
H	2.565843	-2.358434	2.042377
H	1.412701	-2.013448	3.340841
C	-0.907380	-2.417171	1.883789
H	-1.086433	-1.911094	2.843307
H	-1.676532	-2.049300	1.186712
C	1.627239	-4.821758	1.318025
H	1.559454	-5.909726	1.484860
H	2.644593	-4.617128	0.942810
C	2.312968	0.394631	0.803172
C	3.060557	0.551403	2.148896
H	2.505119	1.215380	2.825713
H	3.151968	-0.418456	2.658206
C	4.462864	1.142008	1.919595
H	4.966830	1.237925	2.894657
C	5.274578	0.213216	1.007628
H	5.386041	-0.778203	1.478631
H	6.291044	0.615705	0.862179
C	4.557014	0.077949	-0.342080
H	5.125984	-0.601916	-0.997388
C	4.436395	1.455979	-1.003671
H	3.936004	1.365031	-1.982469
H	5.438008	1.879028	-1.189426
C	3.626820	2.381460	-0.087829
H	3.520900	3.371489	-0.558978
C	2.227753	1.787246	0.124262
H	1.624611	2.475965	0.732314
H	1.712796	1.683689	-0.843384
C	3.158690	-0.516930	-0.113908
H	3.274005	-1.512569	0.337908
H	2.637927	-0.647917	-1.074594
C	4.338398	2.523632	1.263777

H	5.337519	2.969523	1.124392
H	3.771066	3.205045	1.919560
C	-2.360970	0.741305	0.280334
O	-3.072483	-0.083861	0.828633
P	0.546002	-0.225152	0.989348
Br	-2.195635	0.550037	-2.897773
Pd	-0.736129	0.196924	-0.843732
H	0.343324	-1.102852	-2.876003
H	0.600228	-0.277890	-1.748875
N	0.478709	-1.734854	-3.728420
C	0.499098	-0.848651	-4.911389
H	-0.470332	-0.342914	-4.982080
H	1.286165	-0.097521	-4.772551
H	0.701453	-1.445098	-5.811006
C	-0.645747	-2.692396	-3.762255
H	-0.638183	-3.281205	-2.837375
H	-1.581305	-2.123559	-3.818788
H	-0.536091	-3.355340	-4.630963
C	1.778655	-2.404508	-3.527484
H	2.558986	-1.640711	-3.423110
H	1.733973	-3.005200	-2.612325
H	1.999634	-3.048523	-4.388452
C	-3.477522	4.901952	0.678004
H	-3.758321	5.952328	0.788366

int-7prime-me.log

SCF (RwB97XD) = -4540.39434907
E(SCF)+ZPE(0 K)= -4539.523076
H(298 K)= -4539.480181
G(298 K)= -4539.598464
Lowest Frequency = 16.4114cm-1

C	-2.685217	2.045779	0.407755
C	-2.013919	3.069018	-0.264791
H	-1.210950	2.811758	-0.960188
C	-2.361957	4.403474	-0.063168
H	-1.828453	5.187838	-0.607079
C	-4.052787	3.723972	1.502682
H	-4.853069	3.975429	2.205012
C	-3.712503	2.392685	1.294264
H	-4.238560	1.592695	1.820648
C	0.022675	0.398276	2.537151
H	-1.003824	0.018660	2.633495
H	0.594098	-0.020759	3.381016
C	-0.035024	1.923724	2.622926
H	0.971445	2.344471	2.776144
H	-0.398045	2.336139	1.670402
C	-0.952094	2.409524	3.743197
H	-0.593836	2.016459	4.711245
H	-1.957156	1.981567	3.589215
C	-1.054504	3.927848	3.799622
H	-1.433643	4.326619	2.845361
H	-0.072662	4.391648	3.990480
H	-1.739690	4.257931	4.595492
C	0.562866	-2.182202	1.333432
C	0.755840	-2.983955	0.024440
H	-0.000132	-2.653736	-0.704689
H	1.738264	-2.768946	-0.416055
C	0.621838	-4.492019	0.274923
H	0.790081	-5.027075	-0.675082
C	-0.788497	-4.791245	0.795122
H	-1.540567	-4.482904	0.050045
H	-0.916533	-5.874644	0.957983
C	-1.006802	-4.026955	2.105539
H	-2.024200	-4.218213	2.481114
C	0.025349	-4.480660	3.146009
H	-0.081666	-5.559457	3.349328
H	-0.144472	-3.955517	4.100744
C	1.435618	-4.179888	2.622624
H	2.184349	-4.494890	3.367197
C	1.595111	-2.669120	2.373288
H	2.620047	-2.478621	2.023291
H	1.470475	-2.122108	3.321824
C	-0.853441	-2.517059	1.869434
H	-1.028441	-2.005279	2.826849
H	-1.621364	-2.149788	1.170957
C	1.665398	-4.938457	1.308037
H	1.591554	-6.025434	1.478785
H	2.683277	-4.741156	0.930275
C	2.384161	0.268641	0.776555
C	3.131891	0.423574	2.122603
H	2.581804	1.093588	2.797644
H	3.215577	-0.545368	2.634744
C	4.539137	1.002273	1.892766
H	5.042571	1.098035	2.868139
C	5.344590	0.063430	0.985595
H	5.447533	-0.926970	1.460696
H	6.364462	0.457147	0.839815
C	4.627399	-0.071485	-0.364329
H	5.191410	-0.758867	-1.016122
C	4.518905	1.304646	-1.031867
H	4.018650	1.213625	-2.010737
H	5.524205	1.718568	-1.218443
C	3.715907	2.240592	-0.120812
H	3.619018	3.229582	-0.596092
C	2.311511	1.659359	0.092207
H	1.714253	2.355883	0.697008
H	1.795426	1.557113	-0.874918
C	3.223951	-0.653587	-0.135399
H	3.330583	-1.648104	0.320922
H	2.703586	-0.784589	-1.096148
C	4.426851	2.382231	1.231225
H	5.429828	2.819352	1.091644
H	3.864145	3.070856	1.883464
C	-2.299119	0.587915	0.232939
O	-3.020800	-0.257904	0.735197
P	0.611570	-0.337308	0.960530
Br	-2.105857	0.446824	-2.944228
Pd	-0.659492	0.078389	-0.883578
H	0.441316	-1.209528	-2.900858
H	0.688264	-0.373872	-1.786841
N	0.577550	-1.848390	-3.749611
C	0.601901	-0.969876	-4.937986
H	-0.367287	-0.464516	-5.014860
H	1.388475	-0.217787	-4.801341
H	0.807120	-1.571749	-5.833400
C	-0.548848	-2.803624	-3.779366
H	-0.542582	-3.388707	-2.852112
H	-1.483183	-2.232827	-3.838124
H	-0.441035	-3.470530	-4.645316
C	1.875702	-2.518691	-3.540596
H	2.658114	-1.755678	-3.446404
H	1.829739	-3.106808	-2.617306
H	2.095014	-3.174734	-4.392900
C	-3.384451	4.754697	0.824911
C	-3.755317	6.193159	1.069208
H	-3.189417	6.874305	0.417922
H	-4.828019	6.364068	0.888081
H	-3.553676	6.480943	2.113770

int-7prime-nme2.log

SCF (RwB97XD) = -4635.01106860
E(SCF)+ZPE(0 K)= -4634.094127
H(298 K)= -4634.048609
G(298 K)= -4634.172625
Lowest Frequency = 13.7271cm-1

C	-2.574131	1.655432	0.228558
C	-1.930611	2.731364	-0.392330
H	-1.150821	2.527613	-1.130637
C	-2.254001	4.047076	-0.093172
H	-1.725136	4.846904	-0.610307
C	-3.902108	3.258553	1.491846
H	-4.673505	3.434087	2.241143
C	-3.571935	1.951667	1.164717
H	-4.086011	1.122300	1.656985
C	0.059082	0.238441	2.395770
H	-0.972546	-0.138394	2.443922

H	0.589309	-0.188704	3.262743
C	0.001142	1.762244	2.509570
H	1.009439	2.184144	2.642539
H	-0.395057	2.193667	1.579602
C	-0.877497	2.215810	3.674290
H	-0.497070	1.777992	4.614525
H	-1.893179	1.810041	3.532031
C	-0.952336	3.731701	3.802698
H	-1.346458	4.183441	2.879041
H	0.041765	4.169700	3.991351
H	-1.611872	4.034403	4.630804
C	0.730766	-2.347557	1.291787
C	0.978235	-3.180728	0.012536
H	0.225249	-2.900033	-0.740498
H	1.960734	-2.941325	-0.415639
C	0.891151	-4.684345	0.306929
H	1.095978	-5.243660	-0.621874
C	-0.518461	-5.016505	0.809698
H	-1.266147	-4.758162	0.041587
H	-0.611896	-6.098328	1.003814
C	-0.789573	-4.221008	2.091620
H	-1.807301	-4.436799	2.452903
C	0.236642	-4.608088	3.164451
H	0.161716	-5.683477	3.398248
H	0.030883	-4.061141	4.099678
C	1.645952	-4.274662	2.658450
H	2.390042	-4.539923	3.426775
C	1.758875	-2.767926	2.364073
H	2.782668	-2.554319	2.024727
H	1.599979	-2.196469	3.292844
C	-0.683085	-2.714271	1.812325
H	-0.892089	-2.180294	2.750633
H	-1.449344	-2.395052	1.088796
C	1.928118	-5.063973	1.372709
H	1.887587	-6.147412	1.574365
H	2.946053	-4.842495	1.008837
C	2.484815	0.146247	0.718976
C	3.189866	0.341989	2.082939
H	2.595704	0.990920	2.740177
H	3.301248	-0.619099	2.603733
C	4.578341	0.975218	1.885461
H	5.050108	1.101029	2.873238
C	5.445590	0.059328	1.012460
H	5.575525	-0.920123	1.503339
H	6.452210	0.492792	0.888635
C	4.771445	-0.120205	-0.354158
H	5.379354	-0.792405	-0.981882
C	4.629471	1.243248	-1.041619
H	4.161408	1.122104	-2.032852
H	5.623339	1.693357	-1.204401
C	3.765455	2.157969	-0.165339
H	3.644825	3.137031	-0.655643
C	2.379262	1.524462	0.013284
H	1.735100	2.204588	0.587709
H	1.898718	1.388381	-0.967917
C	3.384971	-0.753945	-0.157661
H	3.515517	-1.739589	0.312395
H	2.898238	-0.912446	-1.131525
C	4.430718	2.342329	1.204876
H	5.419586	2.817114	1.088800
H	3.822964	3.014934	1.833126
C	-2.173029	0.229669	-0.024268
O	-2.891824	-0.670172	0.382860
P	0.727042	-0.515092	0.855106
Br	-1.820402	0.206267	-3.186509
Pd	-0.467229	-0.198763	-1.065748
H	0.689501	-1.599463	-2.911538
H	0.932535	-0.625628	-1.922170
N	0.802561	-2.280712	-3.735880
C	0.956964	-1.426858	-4.932549
H	0.050416	-0.819557	-5.039762
H	1.817252	-0.763794	-4.779391
H	1.118270	-2.058142	-5.816592
C	-0.404589	-3.128728	-3.801342
H	-0.505728	-3.674027	-2.855418
H	-1.277403	-2.478843	-3.934469
H	-0.310374	-3.838001	-4.634879
C	2.021862	-3.065137	-3.464466
H	2.854763	-2.375769	-3.279081
H	1.858971	-3.686453	-2.577188
H	2.251023	-3.702931	-4.328169
C	-3.244202	4.347265	0.871714
N	-3.539521	5.644114	1.210947
C	-2.844984	6.734926	0.569588
H	-3.226365	7.687570	0.955414
H	-1.757790	6.705539	0.763265
H	-2.995164	6.733673	-0.523737
C	-4.527608	5.919541	2.226672
H	-4.606342	7.002967	2.374539
H	-5.527821	5.546768	1.944613
H	-4.258102	5.468460	3.197981

int-7prime-ome.log

SCF (RwB97XD) = -4615.60843757
E(SCF)+ZPE(0 K)= -4614.732098
H(298 K)= -4614.688316
G(298 K)= -4614.809144
Lowest Frequency = 9.8983cm-1

C	-2.316991	2.179520	0.451141
C	-1.621908	3.094411	-0.352395
H	-0.946588	2.717582	-1.124466
C	-1.778239	4.461001	-0.182470
H	-1.241010	5.174347	-0.810335
C	-3.339692	4.052968	1.625234
H	-4.009638	4.405440	2.409575
C	-3.175526	2.682311	1.430204
H	-3.719546	1.973106	2.058784
C	0.415781	0.450398	2.389655
H	-0.630761	0.195543	2.607422
H	1.019656	0.021735	3.205789
C	0.531694	1.975036	2.382380
H	1.588281	2.285886	2.401021
H	0.108191	2.379744	1.451946
C	-0.192238	2.613016	3.566415
H	0.212903	2.204802	4.509465
H	-1.254573	2.317708	3.533171
C	-0.090012	4.132402	3.570569
H	-0.499496	4.554381	2.639291
H	0.957346	4.465954	3.654643
H	-0.648053	4.573077	4.411171
C	0.595901	-2.250204	1.368472
C	0.563173	-3.163949	0.121472
H	-0.241149	-2.819763	-0.546994
H	1.502291	-3.076087	-0.440928
C	0.328222	-4.628624	0.514699
H	0.334369	-5.248691	-0.397988
C	-1.030767	-4.750450	1.213742
H	-1.838315	-4.432089	0.533950
H	-1.230362	-5.800647	1.485772
C	-1.025130	-3.869618	2.468295
H	-2.003572	-3.932681	2.970033
C	0.080050	-4.342191	3.422098
H	-0.096899	-5.387463	3.726652
H	0.072328	-3.734117	4.342111
C	1.438816	-4.219495	2.720877
H	2.240717	-4.547893	3.401522
C	1.697580	-2.754481	2.325764
H	2.686390	-2.690412	1.849698
H	1.734181	-2.128510	3.231934
C	-0.770118	-2.403796	2.086183
H	-0.783353	-1.806584	3.009279
H	-1.580917	-2.016902	1.448984
C	1.444831	-5.093884	1.459050
H	1.299016	-6.153013	1.729324
H	2.423371	-5.022595	0.953850
C	2.550072	-0.035721	0.411577
C	3.451538	0.133980	1.657927
H	3.036939	0.891754	2.336552

H	3.508927	-0.804651	2.226304
C	4.868824	0.565234	1.243376
H	5.481468	0.674982	2.152757
C	5.487672	-0.499203	0.328764
H	5.558844	-1.462464	0.861977
H	6.514345	-0.209599	0.048723
C	4.618038	-0.653443	-0.925897
H	5.046941	-1.429514	-1.580967
C	4.556045	0.681793	-1.677104
H	3.947606	0.575507	-2.590841
H	5.567479	0.987079	-1.994228
C	3.939551	1.744397	-0.759395
H	3.876642	2.706278	-1.292586
C	2.521891	1.308851	-0.363227
H	2.052823	2.094476	0.245069
H	1.899802	1.191711	-1.264178
C	3.202824	-1.088184	-0.513050
H	3.272722	-2.059027	-0.001228
H	2.572750	-1.227838	-1.404657
C	4.802154	1.905118	0.499397
H	5.817693	2.238419	0.227129
H	4.373371	2.681806	1.154667
C	-2.111846	0.686729	0.319717
O	-2.860075	-0.057444	0.933671
P	0.762676	-0.454443	0.827343
Br	-2.261101	0.375587	-2.840145
Pd	-0.656257	-0.057556	-0.911956
H	0.023880	-1.698951	-2.817306
H	0.529319	-0.723167	-1.911545
N	-0.060647	-2.427663	-3.598328
C	0.000861	-1.666707	-4.864498
H	-0.840273	-0.963958	-4.884624
H	0.941116	-1.102370	-4.887700
H	-0.043631	-2.363265	-5.712164
C	-1.347127	-3.133510	-3.428629
H	-1.362202	-3.608869	-2.440751
H	-2.156372	-2.396071	-3.484170
H	-1.452895	-3.894615	-4.213228
C	1.095972	-3.331791	-3.451173
H	2.016197	-2.734753	-3.446100
H	1.009806	-3.875201	-2.504156
H	1.116503	-4.042885	-4.287366
C	-2.634723	4.950861	0.814789
O	-2.705268	6.299623	0.926397
C	-3.538501	6.847544	1.924042
H	-3.223972	6.535829	2.934439
H	-3.441762	7.937889	1.844922
H	-4.595476	6.572952	1.770060

int-8-cf3.log

SCF (RwB97XD) = -4838.19266643
E(SCF)+ZPE(0 K)= -4837.343585
H(298 K)= -4837.298596
G(298 K)= -4837.422458
Lowest Frequency = 13.6057cm-1

C	-2.555317	-2.178304	0.317121
C	-2.248037	-3.221019	-0.561261
H	-1.202477	-3.511520	-0.693694
C	-3.255186	-3.875495	-1.262209
H	-3.006073	-4.688099	-1.947327
C	-4.904703	-2.441420	-0.214943
H	-5.942703	-2.129972	-0.087509
C	-3.890420	-1.800071	0.488468
H	-4.122618	-0.985813	1.177901
C	-1.272076	1.029597	-1.273135
H	-1.810018	1.058299	-0.314179
H	-1.235851	2.067831	-1.641104
C	-2.079641	0.155465	-2.233151
H	-1.675175	0.223457	-3.254707
H	-1.990537	-0.902261	-1.941850
C	-3.557641	0.539514	-2.268518
H	-3.652378	1.605299	-2.541876
H	-3.977421	0.445992	-1.252507
C	-4.367236	-0.314484	-3.235369
H	-4.288250	-1.383005	-2.980851
H	-4.011128	-0.194850	-4.271591
H	-5.434853	-0.047042	-3.217452
C	1.029798	1.877945	0.264882
C	2.323505	1.434326	0.987560
H	2.122325	0.495121	1.526371
H	3.119663	1.201518	0.269332
C	2.807648	2.514079	1.964972
H	3.744587	2.172912	2.435905
C	1.738105	2.746330	3.038117
H	1.564531	1.819246	3.610625
H	2.075713	3.507991	3.760996
C	0.439850	3.197341	2.359162
H	-0.345791	3.343942	3.117364
C	0.688789	4.512647	1.608822
H	1.012317	5.299001	2.311357
H	-0.246296	4.863839	1.141366
C	1.761695	4.286658	0.536029
H	1.942982	5.224735	-0.012695
C	1.288181	3.214937	-0.461175
H	2.056858	3.093956	-1.236849
H	0.370995	3.558452	-0.966579
C	-0.037878	2.130838	1.360803
H	-0.974087	2.486970	0.907020
H	-0.280038	1.189448	1.880015
C	3.064285	3.820919	1.201908
H	3.436838	4.597539	1.890682
H	3.845384	3.664800	0.439159
C	1.360788	0.424175	-2.449914
C	0.931985	1.512027	-3.461897
H	-0.148577	1.463522	-3.655812
H	1.136859	2.514755	-3.060195
C	1.681006	1.333566	-4.793658
H	1.351469	2.121181	-5.490626
C	3.192267	1.450002	-4.556291
H	3.436611	2.446903	-4.151231
H	3.735336	1.349270	-5.511141
C	3.634742	0.357033	-3.574634
H	4.716438	0.443528	-3.383612
C	3.324684	-1.022579	-4.167404
H	3.659939	-1.814089	-3.477823
H	3.872958	-1.160933	-5.114831
C	1.813995	-1.137306	-4.404800
H	1.576784	-2.130547	-4.818083
C	1.079221	-0.972390	-3.067104
H	0.000445	-1.118886	-3.219431
H	1.409783	-1.750976	-2.362840
C	2.889482	0.533734	-2.241482
H	3.149424	1.518485	-1.827330
H	3.222117	-0.227550	-1.520613
C	1.358606	-0.046380	-5.383185
H	1.864890	-0.170998	-6.355237
H	0.275067	-0.134092	-5.571740
C	-1.466184	-1.418868	1.060131
O	-1.802294	-0.725191	2.005166
P	0.423079	0.466203	-0.822400
Br	2.949628	-2.310085	0.231094
Pd	0.412741	-1.587841	0.445531
H	2.054256	-1.988485	2.362481
H	0.188408	-2.881605	1.364733
N	2.103453	-1.972024	3.403648
C	2.131346	-3.390576	3.822125
H	3.027060	-3.865534	3.406213
H	2.142875	-3.444545	4.918171
H	1.237372	-3.883897	3.422423
C	0.899592	-1.270254	3.905395
H	0.970132	-1.175961	4.996633
H	0.845596	-0.281945	3.436234
H	0.009578	-1.837449	3.617129
C	3.349361	-1.257524	3.755539
H	3.476937	-1.265459	4.845415
H	4.193834	-1.756320	3.267090
H	3.280050	-0.225530	3.392322
C	-4.583867	-3.476966	-1.094825

C -5.671762 -4.195245 -1.850657
F -6.789872 -3.458620 -1.966714
F -5.290216 -4.526259 -3.097981
F -6.028684 -5.346305 -1.242330

int-8-cl.log

SCF (RwB97XD) = -4960.74244388
E(SCF)+ZPE(0 K)= -4959.907805
H(298 K)= -4959.865213
G(298 K)= -4959.982942
Lowest Frequency = 16.5803cm-1

C -2.579887 -2.174530 0.233887
C -2.251964 -3.201582 -0.655147
H -1.200409 -3.467012 -0.794981
C -3.241965 -3.883539 -1.357133
H -2.983160 -4.687642 -2.047583
C -4.928583 -2.498709 -0.289126
H -5.977183 -2.225734 -0.159713
C -3.925922 -1.834870 0.410360
H -4.179285 -1.032301 1.106504
C -1.270208 1.086040 -1.283357
H -1.804045 1.114051 -0.322273
H -1.224169 2.125835 -1.645620
C -2.090144 0.224518 -2.244169
H -1.699360 0.306338 -3.270176
H -1.997283 -0.836741 -1.967693
C -3.568845 0.606679 -2.252577
H -3.669783 1.676399 -2.507882
H -3.973032 0.496093 -1.231891
C -4.390455 -0.235133 -3.219663
H -4.306914 -1.306836 -2.979718
H -4.048192 -0.101377 -4.258892
H -5.457936 0.031167 -3.184316
C 1.043203 1.893606 0.257643
C 2.334960 1.431130 0.971757
H 2.129323 0.487460 1.500611
H 3.127117 1.199718 0.248741
C 2.831122 2.495603 1.959579
H 3.765565 2.139816 2.424533
C 1.766001 2.725046 3.037563
H 1.584749 1.791729 3.597607
H 2.112175 3.473852 3.769749
C 0.470506 3.196042 2.367244
H -0.311826 3.342161 3.129005
C 0.730125 4.516852 1.630137
H 1.063042 5.292612 2.340057
H -0.202790 4.881798 1.168898
C 1.798172 4.292347 0.552150
H 1.986463 5.234535 0.012869
C 1.312199 3.235655 -0.455195
H 2.077656 3.115319 -1.234278
H 0.397087 3.593415 -0.954466
C -0.019448 2.144906 1.358728
H -0.953384 2.514444 0.911229
H -0.269634 1.199694 1.867243
C 3.097970 3.808263 1.210238
H 3.478586 4.574457 1.906319
H 3.876273 3.653374 0.444390
C 1.353002 0.459959 -2.470372
C 0.933235 1.559377 -3.473407
H -0.148195 1.523096 -3.665177
H 1.149202 2.556919 -3.064516
C 1.677490 1.383043 -4.808115
H 1.354439 2.178920 -5.498710
C 3.190372 1.482365 -4.573372
H 3.445794 2.473897 -4.162028
H 3.730303 1.382761 -5.530156
C 3.623767 0.378117 -3.600303
H 4.706692 0.452347 -3.411093
C 3.298456 -0.994032 -4.202093
H 3.626807 -1.793562 -3.518443
H 3.843585 -1.131512 -5.151472
C 1.786186 -1.091828 -4.437326
H 1.538323 -2.079597 -4.857464
C 1.055732 -0.929054 -3.097085
H -0.024904 -1.062863 -3.248301
H 1.379335 -1.716039 -2.398798
C 2.883032 0.552588 -2.264365
H 3.153262 1.531787 -1.843614
H 3.209465 -0.216996 -1.549377
C 1.339820 0.010763 -5.406820
H 1.842696 -0.111884 -6.380912
H 0.254990 -0.064524 -5.593411
C -1.510416 -1.400568 0.983056
O -1.861163 -0.694845 1.914552
P 0.418555 0.498975 -0.841108
Br 2.924542 -2.288408 0.220896
Pd 0.383023 -1.566971 0.405644
H 1.949571 -1.960023 2.344550
H 0.141405 -2.866975 1.310866
N 1.992649 -1.980521 3.385610
C 2.014745 -3.414382 3.748948
H 2.905845 -3.876585 3.309571
H 2.030517 -3.511633 4.841891
H 1.117137 -3.887193 3.333210
C 0.784493 -1.297633 3.903124
H 0.823811 -1.284651 4.999910
H 0.757202 -0.275979 3.508657
H -0.103425 -1.829263 3.547470
C 3.236758 -1.283247 3.775403
H 3.344427 -1.316315 4.866966
H 4.086483 -1.777250 3.291391
H 3.182012 -0.242879 3.434000
C -4.574671 -3.520685 -1.169921
Cl -5.819096 -4.349854 -2.059649

int-8-cn.log

SCF (RwB97XD) = -4593.33152028
E(SCF)+ZPE(0 K)= -4592.488828
H(298 K)= -4592.445528
G(298 K)= -4592.564869
Lowest Frequency = 16.3737cm-1

C -2.598508 -2.180076 0.242241
C -2.276623 -3.227400 -0.625397
H -1.226535 -3.499681 -0.760273
C -3.273790 -3.915381 -1.306956
H -3.020630 -4.735367 -1.980965
C -4.947865 -2.491904 -0.261805
H -5.993050 -2.205637 -0.132025
C -3.939788 -1.821587 0.418767
H -4.180119 -1.003131 1.100219
C -1.265214 1.080925 -1.292997
H -1.803463 1.115402 -0.334556
H -1.219739 2.117891 -1.662961
C -2.077070 0.208720 -2.250712
H -1.682799 0.285640 -3.275724
H -1.976620 -0.850402 -1.967543
C -3.558288 0.580375 -2.268033
H -3.666097 1.644659 -2.541969
H -3.964109 0.485480 -1.246350
C -4.371995 -0.283837 -3.221991
H -4.281024 -1.350848 -2.964058
H -4.028675 -0.166074 -4.262684
H -5.441405 -0.024811 -3.193723
C 1.038346 1.891552 0.263359
C 2.325165 1.430060 0.987579
H 2.114083 0.489273 1.520144
H 3.121603 1.194334 0.270654
C 2.816214 2.498758 1.973902
H 3.748223 2.145180 2.445288
C 1.745656 2.733257 3.045443
H 1.563142 1.804229 3.611822
H 2.087703 3.487326 3.774111
C 0.453259 3.200008 2.366005
H -0.333524 3.347755 3.122791
C 0.716397 4.518803 1.626646

H	1.044625	5.296651	2.336379
H	-0.213861	4.882124	1.158850
C	1.790840	4.291591	0.555609
H	1.982003	5.232206	0.014701
C	1.310924	3.232098	-0.451267
H	2.080565	3.110542	-1.225850
H	0.398253	3.587743	-0.956452
C	-0.030871	2.145705	1.357572
H	-0.962026	2.514401	0.903590
H	-0.284186	1.202323	1.867861
C	3.087174	3.808825	1.221401
H	3.464995	4.576729	1.917031
H	3.868966	3.651351	0.459655
C	1.366834	0.458150	-2.463554
C	0.950054	1.555800	-3.469839
H	-0.129942	1.514998	-3.669148
H	1.159144	2.554389	-3.060043
C	1.704204	1.381735	-4.799316
H	1.383495	2.176712	-5.491952
C	3.215104	1.485633	-4.554086
H	3.464727	2.477800	-4.140756
H	3.761735	1.387909	-5.507178
C	3.645326	0.382390	-3.578484
H	4.726703	0.459836	-3.382000
C	3.327987	-0.990656	-4.182353
H	3.654242	-1.789344	-3.496703
H	3.879602	-1.126615	-5.128139
C	1.817645	-1.092554	-4.427677
H	1.575214	-2.081080	-4.849145
C	1.077399	-0.931582	-3.092488
H	-0.001782	-1.067579	-3.251799
H	1.397792	-1.718156	-2.392425
C	2.895343	0.554663	-2.247443
H	3.160973	1.534123	-1.824586
H	3.219015	-0.214487	-1.530827
C	1.374959	0.008527	-5.400462
H	1.885341	-0.112683	-6.370768
H	0.291815	-0.070125	-5.595158
C	-1.517771	-1.401394	0.982935
O	-1.870802	-0.699457	1.915660
P	0.421602	0.497317	-0.840235
Br	2.904606	-2.302971	0.218005
Pd	0.371101	-1.571395	0.400512
H	2.004175	-1.998351	2.337549
H	0.125946	-2.872997	1.301094
N	2.027016	-1.999214	3.379789
C	1.989355	-3.423965	3.775940
H	2.871147	-3.929130	3.365821
H	1.981492	-3.495843	4.870936
H	1.082662	-3.873502	3.354163
C	0.839152	-1.259414	3.864055
H	0.873118	-1.208011	4.959949
H	0.850449	-0.252687	3.432576
H	-0.064954	-1.772511	3.522780
C	3.289992	-1.337660	3.772189
H	3.381501	-1.349184	4.865661
H	4.128608	-1.871594	3.311781
H	3.275540	-0.304320	3.406135
C	-4.613948	-3.544818	-1.126601
C	-5.652818	-4.236753	-1.842614
N	-6.484708	-4.786276	-2.417556

int-8-d2-cf3.log

SCF (RwB97XD) = -4838.19266643
E(SCF)+ZPE(0 K)= -4837.349315
H(298 K)= -4837.303998
G(298 K)= -4837.428397
Lowest Frequency = 13.5979cm-1

C	-2.555315	-2.178304	0.317125
C	-2.248035	-3.221019	-0.561258
H	-1.202475	-3.511521	-0.693690
C	-3.255183	-3.875492	-1.262208
H	-3.006070	-4.688096	-1.947326
C	-4.904700	-2.441416	-0.214944
H	-5.942700	-2.129967	-0.087512
C	-3.890418	-1.800069	0.488469
H	-4.122615	-0.985811	1.177903
C	-1.272076	1.029594	-1.273133
H	-1.810018	1.058294	-0.314177
H	-1.235851	2.067829	-1.641101
C	-2.079641	0.155462	-2.233150
H	-1.675175	0.223455	-3.254706
H	-1.990537	-0.902263	-1.941850
C	-3.557640	0.539511	-2.268517
H	-3.652378	1.605297	-2.541876
H	-3.977422	0.445989	-1.252506
C	-4.367235	-0.314486	-3.235370
H	-4.288249	-1.383008	-2.980853
H	-4.011127	-0.194851	-4.271591
H	-5.434852	-0.047043	-3.217454
C	1.029798	1.877943	0.264881
C	2.323505	1.434325	0.987559
H	2.122325	0.495119	1.526371
H	3.119663	1.201518	0.269331
C	2.807648	2.514077	1.964973
H	3.744587	2.172911	2.435904
C	1.738105	2.746327	3.038117
H	1.564532	1.819243	3.610625
H	2.075712	3.507988	3.760997
C	0.439849	3.197338	2.359163
H	-0.345791	3.343937	3.117365
C	0.688788	4.512644	1.608824
H	1.012315	5.298998	2.311359
H	-0.246297	4.863836	1.141369
C	1.761693	4.286656	0.536031
H	1.942980	5.224734	-0.012692
C	1.288180	3.214937	-0.461174
H	2.056857	3.093955	-1.236848
H	0.370993	3.558451	-0.966577
C	-0.037878	2.130834	1.360803
H	-0.974087	2.486967	0.907020
H	-0.280038	1.189445	1.880014
C	3.064284	3.820918	1.201909
H	3.436836	4.597538	1.890684
H	3.845383	3.664800	0.439160
C	1.360787	0.424174	-2.449915
C	0.931985	1.512027	-3.461897
H	-0.148577	1.463523	-3.655811
H	1.136858	2.514754	-3.060194
C	1.681005	1.333566	-4.793658
H	1.351468	2.121181	-5.490626
C	3.192266	1.450002	-4.556291
H	3.436611	2.446903	-4.151231
H	3.735335	1.349270	-5.511142
C	3.634741	0.357032	-3.574635
H	4.716438	0.443527	-3.383614
C	3.324683	-1.022580	-4.167406
H	3.659937	-1.814090	-3.477824
H	3.872957	-1.160933	-5.114833
C	1.813994	-1.137306	-4.404801
H	1.576782	-2.130547	-4.818084
C	1.079221	-0.972391	-3.067105
H	0.000444	-1.118887	-3.219432
H	1.409783	-1.750977	-2.362840
C	2.889482	0.533733	-2.241482
H	3.149424	1.518484	-1.827330
H	3.222116	-0.227552	-1.520614
C	1.358604	-0.046379	-5.383186
H	1.864888	-0.170998	-6.355238
H	0.275065	-0.134091	-5.571740
C	-1.466182	-1.418868	1.060133
O	-1.802292	-0.725192	2.005169
P	0.423079	0.466201	-0.822400
Br	2.949627	-2.310088	0.231091
Pd	0.412742	-1.587842	0.445532
H	2.054252	-1.988483	2.362481
H	0.188408	-2.881605	1.364736
N	2.103451	-1.972022	3.403648

C 2.131344 -3.390572 3.822128
H 3.027058 -3.865532 3.406217
H 2.142873 -3.444538 4.918174
H 1.237370 -3.883894 3.422428
C 0.899593 -1.270248 3.905395
H 0.970135 -1.175952 4.996632
H 0.845599 -0.281939 3.436231
H 0.009577 -1.837443 3.617133
C 3.349361 -1.257523 3.755535
H 3.476939 -1.265456 4.845410
H 4.193832 -1.756320 3.267085
H 3.280051 -0.225528 3.392317
C -4.583865 -3.476962 -1.094826
C -5.671758 -4.195239 -1.850661
F -6.789870 -3.458615 -1.966716
F -5.290211 -4.526249 -3.097986
F -6.028679 -5.346301 -1.242339

int-8-d2-cl.log

SCF (RwB97XD) = -4960.74244388
E(SCF)+ZPE(0 K)= -4959.913485
H(298 K)= -4959.870562
G(298 K)= -4959.988840
Lowest Frequency = 16.5739cm-1

C -2.579888 -2.174526 0.233883
C -2.251965 -3.201577 -0.655153
H -1.200409 -3.467007 -0.794987
C -3.241966 -3.883533 -1.357140
H -2.983160 -4.687635 -2.047591
C -4.928584 -2.498704 -0.289133
H -5.977184 -2.225729 -0.159721
C -3.925924 -1.834866 0.410354
H -4.179287 -1.032297 1.106498
C -1.270206 1.086042 -1.283357
H -1.804041 1.114056 -0.322272
H -1.224166 2.125836 -1.645624
C -2.090145 0.224517 -2.244164
H -1.699365 0.306334 -3.270173
H -1.997284 -0.836741 -1.967685
C -3.568846 0.606677 -2.252569
H -3.669786 1.676396 -2.507878
H -3.973029 0.496095 -1.231881
C -4.390459 -0.235139 -3.219648
H -4.306918 -1.306841 -2.979697
H -4.048199 -0.101390 -4.258879
H -5.457940 0.031160 -3.184299
C 1.043204 1.893607 0.257642
C 2.334960 1.431131 0.971756
H 2.129323 0.487461 1.500610
H 3.127118 1.199718 0.248741
C 2.831122 2.495602 1.959580
H 3.765565 2.139816 2.424535
C 1.766000 2.725045 3.037563
H 1.584748 1.791728 3.597607
H 2.112173 3.473851 3.769749
C 0.470505 3.196041 2.367244
H -0.311827 3.342159 3.129004
C 0.730125 4.516851 1.630137
H 1.063041 5.292611 2.340058
H -0.202790 4.881797 1.168897
C 1.798172 4.292347 0.552151
H 1.986464 5.234535 0.012870
C 1.312201 3.235656 -0.455196
H 2.077658 3.115320 -1.234278
H 0.397089 3.593415 -0.954467
C -0.019447 2.144906 1.358727
H -0.953383 2.514443 0.911227
H -0.269633 1.199693 1.867241
C 3.097970 3.808263 1.210240
H 3.478586 4.574457 1.906322
H 3.876274 3.653375 0.444392
C 1.353003 0.459959 -2.470373
C 0.933235 1.559375 -3.473409
H -0.148195 1.523092 -3.665179
H 1.149199 2.556917 -3.064520
C 1.677489 1.383040 -4.808117
H 1.354437 2.178916 -5.498713
C 3.190371 1.482365 -4.573375
H 3.445792 2.473898 -4.162032
H 3.730302 1.382761 -5.530158
C 3.623768 0.378120 -3.600304
H 4.706693 0.452352 -3.411094
C 3.298460 -0.994031 -4.202092
H 3.626813 -1.793559 -3.518442
H 3.843589 -1.131511 -5.151472
C 1.786190 -1.091830 -4.437325
H 1.538329 -2.079600 -4.857462
C 1.055736 -0.929055 -3.097085
H -0.024900 -1.062867 -3.248300
H 1.379340 -1.716039 -2.398797
C 2.883032 0.552591 -2.264366
H 3.153261 1.531791 -1.843616
H 3.209467 -0.216991 -1.549377
C 1.339822 0.010759 -5.406821
H 1.842698 -0.111889 -6.380913
H 0.254992 -0.064530 -5.593411
C -1.510417 -1.400566 0.983054
O -1.861164 -0.694846 1.914552
P 0.418556 0.498976 -0.841109
Br 2.924541 -2.288409 0.220897
Pd 0.383022 -1.566970 0.405643
H 1.949570 -1.960023 2.344550
H 0.141403 -2.866974 1.310865
N 1.992648 -1.980522 3.385610
C 2.014746 -3.414382 3.748948
H 2.905847 -3.876584 3.309573
H 2.030516 -3.511633 4.841891
H 1.117138 -3.887194 3.333209
C 0.784491 -1.297634 3.903124
H 0.823809 -1.284653 4.999910
H 0.757200 -0.275980 3.508657
H -0.103426 -1.829265 3.547469
C 3.236757 -1.283247 3.775403
H 3.344425 -1.316315 4.866966
H 4.086482 -1.777250 3.291392
H 3.182011 -0.242880 3.434000
C -4.574672 -3.520680 -1.169928
Cl -5.819095 -4.349849 -2.059658

int-8-d2-cn.log

SCF (RwB97XD) = -4593.33152028
E(SCF)+ZPE(0 K)= -4592.494501
H(298 K)= -4592.450869
G(298 K)= -4592.570763
Lowest Frequency = 16.3551cm-1

C -2.598508 -2.180072 0.242244
C -2.276628 -3.227398 -0.625393
H -1.226542 -3.499684 -0.760270
C -3.273798 -3.915377 -1.306951
H -3.020641 -4.735364 -1.980959
C -4.947867 -2.491893 -0.261799
H -5.993051 -2.205622 -0.132018
C -3.939787 -1.821579 0.418772
H -4.180114 -1.003121 1.100223
C -1.265214 1.080923 -1.292999
H -1.803463 1.115399 -0.334558
H -1.219739 2.117888 -1.662961
C -2.077070 0.208717 -2.250714
H -1.682797 0.285637 -3.275726
H -1.976620 -0.850404 -1.967545
C -3.558287 0.580373 -2.268038
H -3.666096 1.644657 -2.541975
H -3.964109 0.485480 -1.246355
C -4.371993 -0.283839 -3.221995
H -4.281024 -1.350850 -2.964061
H -4.028672 -0.166078 -4.262688

H	-5.441403	-0.024813	-3.193729
C	1.038345	1.891551	0.263358
C	2.325164	1.430061	0.987578
H	2.114083	0.489273	1.520141
H	3.121603	1.194338	0.270653
C	2.816210	2.498759	1.973903
H	3.748219	2.145181	2.445290
C	1.745650	2.733256	3.045443
H	1.563135	1.804227	3.611819
H	2.087695	3.487323	3.774112
C	0.453254	3.200007	2.366004
H	-0.333529	3.347753	3.122789
C	0.716392	4.518802	1.626646
H	1.044619	5.296650	2.336378
H	-0.213866	4.882122	1.158849
C	1.790836	4.291592	0.555609
H	1.981999	5.232206	0.014702
C	1.310921	3.232098	-0.451267
H	2.080563	3.110543	-1.225850
H	0.398250	3.587743	-0.956453
C	-0.030874	2.145703	1.357570
H	-0.962029	2.514398	0.903587
H	-0.284188	1.202321	1.867860
C	3.087170	3.808827	1.221403
H	3.464988	4.576731	1.917033
H	3.868963	3.651353	0.459658
C	1.366835	0.458150	-2.463555
C	0.950055	1.555801	-3.469840
H	-0.129941	1.514997	-3.669150
H	1.159144	2.554389	-3.060043
C	1.704206	1.381736	-4.799316
H	1.383497	2.176713	-5.491951
C	3.215106	1.485635	-4.554084
H	3.464728	2.477802	-4.140754
H	3.761738	1.387912	-5.507176
C	3.645328	0.382392	-3.578483
H	4.726704	0.459840	-3.381997
C	3.327991	-0.990654	-4.182353
H	3.654246	-1.789342	-3.496702
H	3.879607	-1.126612	-5.128138
C	1.817649	-1.092553	-4.427677
H	1.575219	-2.081079	-4.849146
C	1.077401	-0.931582	-3.092490
H	-0.001779	-1.067579	-3.251801
H	1.397794	-1.718156	-2.392427
C	2.895344	0.554664	-2.247443
H	3.160972	1.534124	-1.824585
H	3.219016	-0.214485	-1.530826
C	1.374963	0.008529	-5.400463
H	1.885346	-0.112681	-6.370769
H	0.291819	-0.070124	-5.595160
C	-1.517767	-1.401395	0.982936
O	-1.870795	-0.699459	1.915664
P	0.421602	0.497315	-0.840236
Br	2.904608	-2.302970	0.218004
Pd	0.371102	-1.571398	0.400510
H	2.004181	-1.998372	2.337547
H	0.125953	-2.873001	1.301092
N	2.027021	-1.999224	3.379787
C	1.989365	-3.423970	3.775955
H	2.871158	-3.929138	3.365841
H	1.981503	-3.495836	4.870952
H	1.082672	-3.873514	3.354184
C	0.839154	-1.259422	3.864046
H	0.873123	-1.208000	4.959938
H	0.850443	-0.252703	3.432549
H	-0.064950	-1.772533	3.522783
C	3.289993	-1.337661	3.772182
H	3.381504	-1.349178	4.865654
H	4.128612	-1.871593	3.311776
H	3.275536	-0.304323	3.406122
C	-4.613954	-3.544809	-1.126594
C	-5.652828	-4.236740	-1.842606
N	-6.484722	-4.786259	-2.417547

int-8-d2-nme2.log

SCF (RwB97XD) = -4635.02439653
E(SCF)+ZPE(0 K)= -4634.112683
H(298 K)= -4634.066589
G(298 K)= -4634.191457
Lowest Frequency = 15.9194cm-1

C	-2.436049	-2.203254	0.603740
C	-2.156892	-3.229666	-0.304742
H	-1.113167	-3.506057	-0.481738
C	-3.165020	-3.892672	-0.988960
H	-2.890486	-4.683864	-1.685521
C	-4.804088	-2.510716	0.132342
H	-5.831770	-2.199398	0.316118
C	-3.780181	-1.868656	0.811722
H	-4.016130	-1.068183	1.517324
C	-1.304325	0.914714	-1.154578
H	-1.816356	0.952699	-0.181860
H	-1.301122	1.945268	-1.546393
C	-2.122101	0.004011	-2.071394
H	-1.740407	0.045467	-3.103183
H	-2.020368	-1.042327	-1.747282
C	-3.603720	0.376702	-2.085564
H	-3.712813	1.434431	-2.384906
H	-4.000685	0.303666	-1.059092
C	-4.427651	-0.508290	-3.012126
H	-4.342758	-1.567065	-2.721320
H	-4.088035	-0.422108	-4.057493
H	-5.494754	-0.237748	-2.986625
C	1.022654	1.875411	0.272718
C	2.345354	1.489553	0.976161
H	2.182651	0.567241	1.555755
H	3.127448	1.247478	0.245412
C	2.830001	2.617900	1.896678
H	3.785984	2.316191	2.356376
C	1.783251	2.870581	2.987506
H	1.643101	1.962633	3.598323
H	2.122897	3.666420	3.671862
C	0.458779	3.267846	2.326120
H	-0.309665	3.429342	3.098901
C	0.659559	4.556406	1.517272
H	0.984514	5.377602	2.178333
H	-0.295010	4.868563	1.061526
C	1.708699	4.308626	0.425971
H	1.855200	5.227164	-0.165001
C	1.233193	3.186322	-0.513496
H	1.983960	3.048744	-1.303880
H	0.295703	3.488539	-1.008232
C	-0.021373	2.151619	1.385359
H	-0.976114	2.470041	0.941988
H	-0.231128	1.227563	1.947714
C	3.037818	3.898097	1.075884
H	3.410018	4.709385	1.723914
H	3.802854	3.728118	0.299825
C	1.303907	0.324073	-2.389861
C	0.825650	1.365991	-3.427749
H	-0.259165	1.292589	-3.586667
H	1.024167	2.386535	-3.070112
C	1.538256	1.151335	-4.774099
H	1.174555	1.906693	-5.489780
C	3.053448	1.303473	-4.586200
H	3.289762	2.318747	-4.224097
H	3.571120	1.178257	-5.552377
C	3.544840	0.255668	-3.579108
H	4.630051	0.367541	-3.424078
C	3.243359	-1.150067	-4.112554
H	3.612984	-1.910214	-3.405608
H	3.766108	-1.312647	-5.070644
C	1.728723	-1.300482	-4.299245
H	1.498282	-2.312734	-4.668266
C	1.031656	-1.099548	-2.946382
H	-0.048100	-1.272963	-3.058343
H	1.399580	-1.844057	-2.223829
C	2.835515	0.467728	-2.231735

H 3.088686 1.471229 -1.860587
H 3.202559 -0.261245 -1.494345
C 1.224170 -0.254939 -5.302657
H 1.703074 -0.406959 -6.284776
H 0.137123 -0.367820 -5.454221
C -1.352118 -1.429237 1.293125
O -1.641884 -0.696505 2.227815
P 0.416733 0.404495 -0.735139
Br 3.076325 -2.271620 0.331474
Pd 0.513634 -1.594260 0.615173
H 2.114614 -1.927410 2.486676
H 0.344291 -2.859361 1.589159
N 2.232482 -1.894495 3.521815
C 2.310433 -3.305948 3.956168
H 3.185288 -3.771866 3.488978
H 2.392473 -3.344439 5.049890
H 1.401539 -3.818669 3.619530
C 1.047384 -1.206335 4.083335
H 1.173827 -1.104973 5.168866
H 0.953057 -0.222543 3.611334
H 0.151923 -1.787272 3.842471
C 3.484736 -1.155455 3.787779
H 3.676237 -1.144622 4.868410
H 4.305424 -1.648651 3.255038
H 3.377414 -0.130654 3.413646
C -4.522369 -3.541215 -0.796769
N -5.525116 -4.159830 -1.498783
C -6.893727 -3.727312 -1.338589
H -7.544412 -4.336402 -1.976834
H -7.243215 -3.847499 -0.299202
H -7.034141 -2.669433 -1.624646
C -5.201461 -5.148283 -2.501101
H -6.126530 -5.524306 -2.953249
H -4.575029 -4.731900 -3.310345
H -4.668313 -6.011580 -2.068562

int-8-d2-ome.log

SCF (RwB97XD) = -4615.61734205
E(SCF)+ZPE(0 K)= -4614.746594
H(298 K)= -4614.702059
G(298 K)= -4614.826264
Lowest Frequency = 8.1366cm-1

C -2.490698 -1.757663 0.615024
C -2.027291 -2.858033 -0.118703
H -0.948118 -2.993940 -0.237094
C -2.909575 -3.757279 -0.697674
H -2.550034 -4.612687 -1.273062
C -4.774389 -2.476067 0.170110
H -5.843839 -2.303664 0.289787
C -3.869915 -1.586581 0.747252
H -4.235549 -0.728521 1.316042
C -1.284326 1.310983 -1.393031
H -1.860923 1.489460 -0.474375
H -1.271846 2.264285 -1.946038
C -2.015864 0.234525 -2.195751
H -1.615448 0.172433 -3.220105
H -1.845263 -0.752616 -1.740812
C -3.520841 0.484321 -2.270323
H -3.707311 1.477633 -2.715718
H -3.925681 0.527335 -1.244890
C -4.258977 -0.586283 -3.063434
H -4.087680 -1.584324 -2.629927
H -3.918231 -0.616407 -4.111408
H -5.345440 -0.407105 -3.071949
C 0.900191 2.519453 0.070228
C 2.181205 2.278223 0.905410
H 2.012818 1.437170 1.595634
H 3.020367 1.982364 0.262868
C 2.563101 3.536555 1.697672
H 3.491564 3.333200 2.255202
C 1.433743 3.879508 2.675662
H 1.279538 3.045294 3.379809
H 1.698311 4.767228 3.275392
C 0.149262 4.139814 1.881116
H -0.677052 4.367605 2.573460
C 0.364985 5.320336 0.924811
H 0.617586 6.232756 1.491313
H -0.565232 5.532247 0.371024
C 1.495472 4.977441 -0.053997
H 1.651885 5.817156 -0.750493
C 1.124942 3.724368 -0.867060
H 1.935665 3.515218 -1.579461
H 0.216983 3.921950 -1.460379
C -0.229736 2.894742 1.064769
H -1.159395 3.117751 0.520847
H -0.448627 2.044600 1.729930
C 2.786982 4.708162 0.730978
H 3.086327 5.611654 1.288701
H 3.610138 4.472234 0.035262
C 1.423523 0.633581 -2.331513
C 1.006207 1.507779 -3.536620
H -0.059587 1.370549 -3.767434
H 1.146325 2.573874 -3.307344
C 1.832302 1.140612 -4.781561
H 1.511337 1.781129 -5.619197
C 3.323190 1.367492 -4.499243
H 3.505557 2.429230 -4.260703
H 3.920183 1.131956 -5.396673
C 3.754767 0.480826 -3.323941
H 4.820924 0.648648 -3.102315
C 3.528493 -0.993364 -3.680691
H 3.854861 -1.637366 -2.847962
H 4.131774 -1.268261 -4.562746
C 2.037976 -1.217280 -3.963848
H 1.861053 -2.277009 -4.209058
C 1.226216 -0.858594 -2.710904
H 0.162654 -1.074507 -2.887410
H 1.543059 -1.489573 -1.866214
C 2.933469 0.849001 -2.078120
H 3.132184 1.901380 -1.830080
H 3.258077 0.240747 -1.221312
C 1.596008 -0.333144 -5.137823
H 2.160011 -0.598276 -6.048075
H 0.528706 -0.502195 -5.360155
C -1.548238 -0.743946 1.228058
O -2.014694 0.123281 1.945474
P 0.404981 0.914560 -0.775751
Br 2.981344 -1.531066 0.859628
Pd 0.395463 -0.873813 0.837479
H 2.088538 -2.811443 2.418104
H 0.125390 -1.976538 1.966888
N 2.002734 -3.510634 3.193751
C 1.073673 -4.567734 2.737729
H 1.451009 -4.985565 1.796968
H 1.013790 -5.351527 3.503849
H 0.088668 -4.120024 2.564732
C 1.490038 -2.786834 4.378170
H 1.409594 -3.484499 5.221891
H 2.184153 -1.973956 4.621503
H 0.511636 -2.360789 4.128533
C 3.366835 -4.038425 3.415856
H 3.344518 -4.770827 4.232995
H 3.718999 -4.512183 2.492539
H 4.032911 -3.206119 3.669206
C -4.293482 -3.570254 -0.559382
O -5.080995 -4.488282 -1.167916
C -6.481576 -4.330159 -1.089650
H -6.809711 -3.381727 -1.546997
H -6.919403 -5.164727 -1.651754
H -6.840595 -4.375902 -0.047856

int-8doubleprime.log

SCF (RwB97XD) = -4155.81790760
E(SCF)+ZPE(0 K)= -4155.084211
H(298 K)= -4155.050349
G(298 K)= -4155.148854
Lowest Frequency = 23.1657cm-1

C	0.200563	-0.252230	2.218461
H	-0.342012	-1.155956	1.910801
H	0.972655	-0.589527	2.927998
C	-0.796309	0.674207	2.910126
H	-0.282898	1.555959	3.324073
H	-1.501778	1.070510	2.157319
C	-1.564843	-0.009158	4.040397
H	-2.059093	-0.919642	3.655662
H	-0.849758	-0.361624	4.803783
C	-2.602635	0.898276	4.689092
H	-3.351927	1.237120	3.954909
H	-2.133690	1.799384	5.115622
H	-3.139495	0.386116	5.502184
C	2.151588	1.733806	1.307853
C	1.337039	3.053414	1.379668
H	0.475474	2.941303	2.053419
H	0.920066	3.276397	0.384528
C	2.213133	4.221832	1.854999
H	1.591218	5.129790	1.907279
C	2.792800	3.908742	3.240720
H	1.977541	3.777784	3.972707
H	3.410838	4.750982	3.595136
C	3.636587	2.629764	3.159518
H	4.052246	2.394814	4.152959
C	4.779394	2.830869	2.155423
H	5.439008	3.649532	2.489002
H	5.400179	1.920282	2.100700
C	4.191819	3.151959	0.774570
H	5.007473	3.284856	0.045592
C	3.311398	1.978570	0.316816
H	2.903289	2.180837	-0.687364
H	3.945102	1.084036	0.238169
C	3.355894	4.434284	0.855302
H	3.988282	5.282276	1.168738
H	2.947512	4.683868	-0.137758
C	2.745592	1.459861	2.707440
H	3.332250	0.529560	2.707682
H	1.940407	1.329360	3.446361
C	1.957743	-1.110379	0.084807
C	0.963600	-2.291062	-0.062672
H	0.538401	-2.564021	0.914910
H	0.129322	-1.976431	-0.713068
C	1.651109	-3.536247	-0.642787
H	0.907670	-4.346491	-0.722851
C	2.783525	-3.965377	0.300343
H	2.374313	-4.224127	1.291764
H	3.280381	-4.869924	-0.088519
C	3.796200	-2.820191	0.429104
H	4.611052	-3.120280	1.107527
C	4.371413	-2.490743	-0.955795
H	4.901603	-3.367782	-1.363811
H	5.111938	-1.677106	-0.874845
C	3.232258	-2.073124	-1.896422
H	3.641962	-1.823027	-2.887938
C	2.525752	-0.833157	-1.329919
H	1.701515	-0.524529	-1.993738
H	3.235052	0.002931	-1.308997
C	3.105189	-1.571571	1.006746
H	3.853841	-0.773916	1.117405
H	2.721989	-1.793562	2.016526
C	2.224257	-3.219377	-2.028039
H	1.414188	-2.930204	-2.718247
H	2.709037	-4.114422	-2.453099
P	0.972612	0.396791	0.667354
H	-1.952149	-0.034323	-0.605989
N	-2.877096	-0.593438	-0.603286
C	-3.961095	0.393077	-0.413676
H	-3.811314	0.898994	0.547709
H	-3.898416	1.132709	-1.221377
H	-4.929539	-0.124111	-0.424925
C	-2.811507	-1.558958	0.507939
H	-1.952593	-2.222430	0.356463
H	-2.691056	-1.008318	1.447750
H	-3.737481	-2.148134	0.538982
C	-2.955809	-1.258098	-1.920889
H	-2.906364	-0.483976	-2.696729
H	-2.095900	-1.930124	-2.029227
H	-3.893618	-1.825356	-1.988522
Pd	-0.375057	1.202253	-0.927262
Br	-1.734041	2.182407	-2.767252

int-8-h.log

SCF (RwB97XD) = -4501.12681404
E(SCF)+ZPE(0 K)= -4500.282366
H(298 K)= -4500.241048
G(298 K)= -4500.355463
Lowest Frequency = 20.5194cm-1

C	-2.597206	-2.151316	0.227444
C	-2.268146	-3.189587	-0.649281
H	-1.215665	-3.455658	-0.781862
C	-3.265882	-3.875835	-1.339160
H	-2.997907	-4.689632	-2.016918
C	-4.941595	-2.482505	-0.295151
H	-5.988620	-2.200074	-0.161360
C	-3.942946	-1.804355	0.399089
H	-4.190906	-0.991586	1.085584
C	-1.255252	1.099969	-1.295908
H	-1.791630	1.138993	-0.336684
H	-1.197614	2.137080	-1.664391
C	-2.082093	0.241148	-2.253124
H	-1.686852	0.310422	-3.278475
H	-2.004587	-0.818935	-1.967674
C	-3.555371	0.643605	-2.268659
H	-3.640466	1.713240	-2.530549
H	-3.964209	0.543361	-1.248770
C	-4.385894	-0.192625	-3.232880
H	-4.325807	-1.262244	-2.977040
H	-4.032580	-0.077676	-4.270830
H	-5.447791	0.096937	-3.208959
C	1.058415	1.895684	0.248188
C	2.344465	1.427985	0.969030
H	2.130539	0.488566	1.502034
H	3.137353	1.186864	0.250040
C	2.845277	2.494210	1.952578
H	3.775488	2.133977	2.422578
C	1.778490	2.737371	3.025882
H	1.588522	1.808511	3.590371
H	2.127713	3.487733	3.755083
C	0.488577	3.213874	2.348828
H	-0.295038	3.369778	3.107334
C	0.760107	4.528675	1.605381
H	1.096606	5.305877	2.312103
H	-0.168744	4.897937	1.139380
C	1.829917	4.290584	0.531873
H	2.026602	5.228521	-0.011843
C	1.339597	3.231693	-0.471179
H	2.106721	3.101089	-1.247024
H	0.428892	3.593374	-0.975665
C	-0.005728	2.160796	1.344573
H	-0.935614	2.534527	0.892251
H	-0.264596	1.220072	1.857032
C	3.124079	3.800783	1.196923
H	3.508040	4.568051	1.890026
H	3.903712	3.635935	0.434495
C	1.365977	0.443413	-2.469911
C	0.959235	1.539320	-3.481903
H	-0.121746	1.510498	-3.677502
H	1.181827	2.537862	-3.078885
C	1.707329	1.347745	-4.812371
H	1.393843	2.141496	-5.509845
C	3.220095	1.435970	-4.572294
H	3.482205	2.428139	-4.166669
H	3.762965	1.325302	-5.526245
C	3.640436	0.334845	-3.589998
H	4.723223	0.401299	-3.397061
C	3.305883	-1.038593	-4.183796
H	3.624789	-1.836110	-3.493380

H 3.853638 -1.187047 -5.130050
C 1.793715 -1.125378 -4.424388
H 1.539326 -2.113989 -4.838650
C 1.059413 -0.947247 -3.088263
H -0.021846 -1.073021 -3.242296
H 1.373522 -1.732185 -2.383335
C 2.895742 0.524684 -2.258383
H 3.172218 1.504592 -1.843303
H 3.212513 -0.242559 -1.536511
C 1.360674 -0.025785 -5.403247
H 1.866622 -0.159285 -6.374371
H 0.276011 -0.093253 -5.593813
C -1.527811 -1.376489 0.974809
O -1.874326 -0.658254 1.898407
P 0.426042 0.499195 -0.844341
Br 2.913677 -2.296741 0.252423
Pd 0.370134 -1.558713 0.412867
H 1.893053 -1.949206 2.365681
H 0.111457 -2.849106 1.327511
N 1.933999 -1.963579 3.406987
C 1.938887 -3.395086 3.779593
H 2.826616 -3.869609 3.346513
H 1.949380 -3.485289 4.873225
H 1.037897 -3.860649 3.363036
C 0.731483 -1.263839 3.914831
H 0.765463 -1.245504 5.011760
H 0.718311 -0.244186 3.514451
H -0.160938 -1.786464 3.556938
C 3.184414 -1.277810 3.796410
H 3.288997 -1.306651 4.888432
H 4.029558 -1.783201 3.316187
H 3.142014 -0.238578 3.450001
C -4.604116 -3.521584 -1.164707
H -5.387064 -4.054623 -1.709641

int-8-me.log

SCF (RwB97XD) = -4540.40693917
E(SCF)+ZPE(0 K)= -4539.535308
H(298 K)= -4539.491988
G(298 K)= -4539.611595
Lowest Frequency = 19.6263cm-1

C -2.565700 -2.150568 0.304677
C -2.253820 -3.186168 -0.578563
H -1.205110 -3.462041 -0.721582
C -3.260119 -3.860405 -1.267404
H -2.994357 -4.671795 -1.950354
C -4.912833 -2.470591 -0.212472
H -5.957624 -2.178943 -0.070603
C -3.911120 -1.802720 0.481815
H -4.156888 -0.993080 1.172962
C -1.245433 1.062657 -1.274114
H -1.777619 1.107182 -0.312775
H -1.194690 2.096891 -1.651735
C -2.071690 0.190632 -2.219812
H -1.681425 0.252363 -3.247528
H -1.987141 -0.865920 -1.923987
C -3.547850 0.582295 -2.232970
H -3.644236 1.645722 -2.515478
H -3.948970 0.498014 -1.208747
C -4.376384 -0.281565 -3.174373
H -4.293618 -1.345306 -2.901187
H -4.037029 -0.178351 -4.218169
H -5.442707 -0.009274 -3.142546
C 1.074417 1.884288 0.248190
C 2.366352 1.428627 0.966162
H 2.159314 0.492684 1.507823
H 3.156451 1.185053 0.244918
C 2.867891 2.505748 1.937319
H 3.801885 2.153684 2.406095
C 1.805576 2.755295 3.013599
H 1.621123 1.831009 3.587365
H 2.155915 3.513476 3.734158
C 0.510775 3.221228 2.338528
H -0.269416 3.381864 3.099571
C 0.773857 4.529580 1.580896
H 1.111472 5.314827 2.278176
H -0.158750 4.891082 1.116297
C 1.838804 4.284543 0.504144
H 2.029572 5.217772 -0.049727
C 1.347039 3.213920 -0.485885
H 2.110622 3.078165 -1.264362
H 0.432418 3.567248 -0.989204
C 0.015084 2.156525 1.347441
H -0.918454 2.522385 0.896096
H -0.237855 1.220266 1.870826
C 3.138040 3.805956 1.167661
H 3.522402 4.581296 1.851496
H 3.914589 3.636533 0.403080
C 1.371472 0.404540 -2.455650
C 0.955328 1.489563 -3.475551
H -0.126650 1.455555 -3.664604
H 1.176949 2.492602 -3.083283
C 1.696307 1.287972 -4.808467
H 1.376176 2.074019 -5.511684
C 3.210157 1.383478 -4.577929
H 3.471153 2.380219 -4.182917
H 3.748099 1.265836 -5.533856
C 3.639839 0.293008 -3.587864
H 4.723496 0.364846 -3.401810
C 3.306599 -1.086981 -4.167144
H 3.632446 -1.877137 -3.471544
H 3.849241 -1.241959 -5.115310
C 1.793330 -1.181160 -4.397726
H 1.539823 -2.174515 -4.801073
C 1.066482 -0.993111 -3.058869
H -0.015088 -1.124750 -3.205123
H 1.387990 -1.770016 -2.348364
C 2.902096 0.492747 -2.253816
H 3.177521 1.477395 -1.849432
H 3.225320 -0.266808 -1.526686
C 1.350686 -0.092286 -5.384200
H 1.851037 -0.233387 -6.357159
H 0.265091 -0.165050 -5.567448
C -1.489949 -1.381214 1.041008
O -1.822437 -0.657524 1.966066
P 0.440915 0.473295 -0.824959
Br 2.946718 -2.307384 0.277634
Pd 0.401989 -1.570149 0.457807
H 1.940484 -1.941560 2.398464
H 0.152910 -2.851905 1.388174
N 1.994592 -1.938683 3.439315
C 2.013776 -3.363622 3.835804
H 2.903450 -3.838049 3.406630
H 2.030479 -3.434852 4.930788
H 1.114488 -3.843597 3.432006
C 0.793815 -1.238173 3.950268
H 0.847772 -1.189966 5.045460
H 0.761542 -0.230198 3.522401
H -0.099376 -1.779339 3.623560
C 3.245019 -1.238412 3.801903
H 3.366858 -1.254500 4.892389
H 4.086637 -1.742066 3.313662
H 3.188317 -0.203519 3.444967
C -4.605164 -3.512077 -1.100825
C -5.697013 -4.207331 -1.868298
H -5.318941 -5.091064 -2.401350
H -6.511182 -4.532483 -1.202521
H -6.141546 -3.529724 -2.615661

int-8-nme2.log

SCF (RwB97XD) = -4635.02439653
E(SCF)+ZPE(0 K)= -4634.107012
H(298 K)= -4634.061250
G(298 K)= -4634.185565
Lowest Frequency = 15.9314cm-1

C -2.436048 -2.203256 0.603739

C	-2.156893	-3.229672	-0.304738
H	-1.113170	-3.506068	-0.481732
C	-3.165024	-3.892678	-0.988953
H	-2.890491	-4.683875	-1.685511
C	-4.804087	-2.510712	0.132340
H	-5.831769	-2.199388	0.316112
C	-3.780178	-1.868651	0.811718
H	-4.016125	-1.068174	1.517316
C	-1.304326	0.914711	-1.154577
H	-1.816357	0.952695	-0.181859
H	-1.301123	1.945265	-1.546391
C	-2.122101	0.004008	-2.071394
H	-1.740404	0.045462	-3.103181
H	-2.020369	-1.042330	-1.747280
C	-3.603719	0.376701	-2.085567
H	-3.712809	1.434430	-2.384911
H	-4.000686	0.303667	-1.059097
C	-4.427648	-0.508291	-3.012131
H	-4.342757	-1.567066	-2.721324
H	-4.088030	-0.422109	-4.057498
H	-5.494751	-0.237747	-2.986633
C	1.022654	1.875411	0.272719
C	2.345354	1.489554	0.976162
H	2.182651	0.567241	1.555755
H	3.127447	1.247479	0.245412
C	2.830000	2.617901	1.896678
H	3.785983	2.316193	2.356375
C	1.783250	2.870581	2.987506
H	1.643102	1.962633	3.598324
H	2.122896	3.666421	3.671862
C	0.458778	3.267845	2.326121
H	-0.309666	3.429340	3.098903
C	0.659556	4.556406	1.517273
H	0.984511	5.377601	2.178334
H	-0.295014	4.868561	1.061526
C	1.708696	4.308626	0.425971
H	1.855196	5.227163	-0.165001
C	1.233190	3.186321	-0.513495
H	1.983958	3.048744	-1.303879
H	0.295700	3.488537	-1.008231
C	-0.021374	2.151618	1.385361
H	-0.976115	2.470038	0.941989
H	-0.231128	1.227562	1.947715
C	3.037816	3.898098	1.075884
H	3.410015	4.709387	1.723913
H	3.802852	3.728119	0.299825
C	1.303907	0.324073	-2.389860
C	0.825650	1.365991	-3.427747
H	-0.259166	1.292588	-3.586666
H	1.024165	2.386535	-3.070110
C	1.538255	1.151336	-4.774097
H	1.174553	1.906695	-5.489778
C	3.053447	1.303476	-4.586199
H	3.289760	2.318749	-4.224096
H	3.571119	1.178260	-5.552377
C	3.544840	0.255670	-3.579107
H	4.630051	0.367545	-3.424078
C	3.243360	-1.150065	-4.112555
H	3.612986	-1.910211	-3.405610
H	3.766109	-1.312644	-5.070645
C	1.728725	-1.300481	-4.299246
H	1.498285	-2.312733	-4.668267
C	1.031658	-1.099549	-2.946382
H	-0.048098	-1.272965	-3.058343
H	1.399582	-1.844057	-2.223830
C	2.835516	0.467729	-2.231735
H	3.088685	1.471230	-1.860586
H	3.202560	-0.261245	-1.494345
C	1.224171	-0.254938	-5.302656
H	1.703074	-0.406956	-6.284776
H	0.137123	-0.367819	-5.454220
C	-1.352115	-1.429240	1.293123
O	-1.641881	-0.696508	2.227814
P	0.416733	0.404494	-0.735137
Br	3.076329	-2.271619	0.331475
Pd	0.513637	-1.594262	0.615172
H	2.114621	-1.927413	2.486675
H	0.344296	-2.859366	1.589154
N	2.232486	-1.894496	3.521815
C	2.310437	-3.305949	3.956171
H	3.185296	-3.771866	3.488987
H	2.392469	-3.344438	5.049893
H	1.401546	-3.818672	3.619528
C	1.047386	-1.206336	4.083330
H	1.173827	-1.104971	5.168860
H	0.953058	-0.222545	3.611326
H	0.151927	-1.787275	3.842465
C	3.484738	-1.155455	3.787780
H	3.676236	-1.144619	4.868413
H	4.305429	-1.648653	3.255045
H	3.377418	-0.130656	3.413643
C	-4.522372	-3.541216	-0.796764
N	-5.525121	-4.159834	-1.498775
C	-6.893729	-3.727306	-1.338589
H	-7.544418	-4.336407	-1.976820
H	-7.243216	-3.847468	-0.299201
H	-7.034139	-2.669431	-1.624670
C	-5.201467	-5.148282	-2.501097
H	-6.126537	-5.524308	-2.953242
H	-4.575040	-4.731895	-3.310344
H	-4.668313	-6.011579	-2.068563

int-8-ome.log

SCF (RwB97XD) = -4615.61734205
E(SCF)+ZPE(0 K)= -4614.740899
H(298 K)= -4614.696688
G(298 K)= -4614.820350
Lowest Frequency = 8.1433cm-1

C	-2.490697	-1.757663	0.615026
C	-2.027293	-2.858032	-0.118702
H	-0.948120	-2.993942	-0.237092
C	-2.909579	-3.757275	-0.697675
H	-2.550041	-4.612684	-1.273063
C	-4.774390	-2.476060	0.170110
H	-5.843839	-2.303653	0.289787
C	-3.869914	-1.586576	0.747254
H	-4.235546	-0.728517	1.316044
C	-1.284327	1.310979	-1.393031
H	-1.860924	1.489454	-0.474375
H	-1.271847	2.264282	-1.946036
C	-2.015864	0.234522	-2.195752
H	-1.615446	0.172430	-3.220106
H	-1.845265	-0.752619	-1.740814
C	-3.520840	0.484319	-2.270328
H	-3.707308	1.477632	-2.715724
H	-3.925683	0.527335	-1.244896
C	-4.258976	-0.586283	-3.063441
H	-4.087682	-1.584325	-2.629934
H	-3.918227	-0.616408	-4.111414
H	-5.345439	-0.407104	-3.071958
C	0.900189	2.519451	0.070228
C	2.181204	2.278221	0.905409
H	2.012818	1.437168	1.595633
H	3.020366	1.982364	0.262867
C	2.563099	3.536554	1.697672
H	3.491563	3.333199	2.255201
C	1.433741	3.879505	2.675663
H	1.279538	3.045290	3.379809
H	1.698309	4.767224	3.275393
C	0.149259	4.139810	1.881117
H	-0.677054	4.367600	2.573463
C	0.364981	5.320333	0.924813
H	0.617581	6.232752	1.491315
H	-0.565236	5.532243	0.371026
C	1.495468	4.977439	-0.053996
H	1.651879	5.817155	-0.750491
C	1.124938	3.724366	-0.867059
H	1.935661	3.515218	-1.579460

H	0.216979	3.921948	-1.460378
C	-0.229738	2.894738	1.064770
H	-1.159397	3.117747	0.520848
H	-0.448628	2.044596	1.729930
C	2.786978	4.708162	0.730978
H	3.086323	5.611653	1.288701
H	3.610134	4.472234	0.035262
C	1.423523	0.633580	-2.331513
C	1.006206	1.507778	-3.536620
H	-0.059587	1.370547	-3.767434
H	1.146324	2.573872	-3.307344
C	1.832301	1.140611	-4.781562
H	1.511336	1.781128	-5.619197
C	3.323189	1.367492	-4.499243
H	3.505556	2.429230	-4.260703
H	3.920183	1.131957	-5.396673
C	3.754767	0.480825	-3.323942
H	4.820924	0.648648	-3.102315
C	3.528493	-0.993364	-3.680692
H	3.854862	-1.637366	-2.847963
H	4.131775	-1.268261	-4.562747
C	2.037976	-1.217281	-3.963849
H	1.861054	-2.277010	-4.209059
C	1.226216	-0.858595	-2.710905
H	0.162654	-1.074509	-2.887411
H	1.543060	-1.489575	-1.866214
C	2.933469	0.849001	-2.078120
H	3.132183	1.901379	-1.830080
H	3.258077	0.240747	-1.221312
C	1.596009	-0.333145	-5.137824
H	2.160012	-0.598277	-6.048076
H	0.528706	-0.502197	-5.360156
C	-1.548235	-0.743949	1.228062
O	-2.014688	0.123276	1.945483
P	0.404981	0.914557	-0.775751
Br	2.981347	-1.531067	0.859626
Pd	0.395465	-0.873816	0.837479
H	2.088543	-2.811443	2.418105
H	0.125394	-1.976542	1.966887
N	2.002740	-3.510633	3.193752
C	1.073682	-4.567736	2.737731
H	1.451021	-4.985567	1.796971
H	1.013801	-5.351528	3.503851
H	0.088676	-4.120028	2.564732
C	1.490040	-2.786833	4.378170
H	1.409598	-3.484497	5.221892
H	2.184152	-1.973953	4.621503
H	0.511637	-2.360791	4.128532
C	3.366842	-4.038420	3.415861
H	3.344526	-4.770822	4.233000
H	3.719009	-4.512178	2.492545
H	4.032915	-3.206112	3.669212
C	-4.293486	-3.570247	-0.559383
O	-5.081001	-4.488272	-1.167919
C	-6.481581	-4.330146	-1.089652
H	-6.809714	-3.381713	-1.546997
H	-6.919411	-5.164713	-1.651756
H	-6.840600	-4.375889	-0.047858

int-8prime-ome.log

SCF (RwB97XD) = -4615.62090544
E(SCF)+ZPE(0 K)= -4614.743565
H(298 K)= -4614.699810
G(298 K)= -4614.819499
Lowest Frequency = 16.5865cm-1

C	0.145054	0.042278	2.252478
H	-0.677089	-0.685088	2.182920
H	0.882887	-0.387624	2.949203
C	-0.430473	1.336999	2.826768
H	0.374044	2.046659	3.073764
H	-1.056241	1.836125	2.072464
C	-1.267889	1.091853	4.080582
H	-2.088357	0.397366	3.832335
H	-0.650711	0.580027	4.840258
C	-1.846529	2.374324	4.663708
H	-2.462394	2.901526	3.918428
H	-1.049650	3.065733	4.982902
H	-2.480876	2.171002	5.540495
C	2.248270	1.337074	0.645914
C	1.594565	2.722225	0.392028
H	0.815729	2.926406	1.140670
H	1.098292	2.710706	-0.590698
C	2.647544	3.838345	0.425526
H	2.143260	4.803661	0.258865
C	3.349203	3.847140	1.790208
H	2.616181	4.039238	2.592239
H	4.091996	4.661578	1.832500
C	4.037041	2.494132	2.017938
H	4.539793	2.494100	2.998840
C	5.067370	2.249059	0.907875
H	5.840282	3.036079	0.926625
H	5.582841	1.287752	1.074698
C	4.352673	2.236566	-0.449571
H	5.081150	2.042086	-1.252893
C	3.301740	1.114579	-0.463766
H	2.805333	1.073622	-1.444678
H	3.818347	0.155793	-0.311056
C	3.674715	3.591508	-0.685974
H	4.428458	4.397176	-0.697434
H	3.175195	3.597158	-1.668142
C	2.983710	1.372682	2.006108
H	3.475642	0.409498	2.204079
H	2.278468	1.542947	2.831110
C	1.522673	-1.661441	0.366337
C	0.370516	-2.645238	0.696819
H	0.072451	-2.545879	1.750870
H	-0.519838	-2.401178	0.095409
C	0.806075	-4.100118	0.464098
H	-0.039185	-4.762874	0.710440
C	1.996280	-4.418190	1.379275
H	1.700872	-4.302485	2.435749
H	2.313068	-5.466441	1.246047
C	3.156480	-3.470432	1.048363
H	4.013438	-3.689498	1.705578
C	3.569242	-3.659563	-0.418299
H	3.918184	-4.692767	-0.584087
H	4.411578	-2.991382	-0.664275
C	2.372434	-3.351023	-1.328241
H	2.668181	-3.472434	-2.383009
C	1.921109	-1.900622	-1.108701
H	1.066761	-1.661422	-1.757318
H	2.724874	-1.219692	-1.414274
C	2.722140	-2.011023	1.271927
H	3.574213	-1.352533	1.052173
H	2.461909	-1.860727	2.332364
C	1.217142	-4.302800	-0.998429
H	0.361042	-4.104703	-1.664713
H	1.519888	-5.350136	-1.167659
P	0.850617	0.085101	0.550483
Pd	-0.811646	0.572004	-1.129153
Br	0.715612	1.050379	-3.211702
C	-3.170097	1.030073	0.740470
C	-3.069048	2.399827	0.458688
C	-4.038374	0.623551	1.756167
C	-3.813309	3.331165	1.164482
H	-2.386935	2.726365	-0.331324
C	-4.784633	1.545839	2.486953
H	-4.119467	-0.442441	1.981459
H	-3.738906	4.397948	0.945486
H	-5.441224	1.193812	3.282327
C	-2.347105	0.008693	0.002104
O	-2.657224	-1.172522	0.089790
H	-2.078803	0.947158	-2.054136
H	-1.943141	-0.376380	-2.930487
N	-2.258039	-1.183655	-3.530510
C	-2.123939	-0.758792	-4.938834
H	-2.752380	0.124894	-5.101274
H	-1.076365	-0.492779	-5.122663

H -2.443047 -1.576381 -5.598465
C -3.662090 -1.439714 -3.144012
H -3.689421 -1.688438 -2.076547
H -4.246692 -0.528895 -3.319919
H -4.060975 -2.266463 -3.745565
C -1.370914 -2.316737 -3.192796
H -0.343101 -2.041931 -3.457227
H -1.438822 -2.492872 -2.112330
H -1.686723 -3.208103 -3.750377
C -4.672263 2.910315 2.191515
O -5.339756 3.889118 2.844043
C -6.180657 3.530240 3.920067
H -5.618249 3.028568 4.725239
H -6.599665 4.466415 4.309965
H -7.008211 2.879225 3.592030

int-8tripleprime.log

SCF (RwB97XD) = -4155.81521684
E(SCF)+ZPE(0 K)= -4155.080886
H(298 K)= -4155.046763
G(298 K)= -4155.146750
Lowest Frequency = 12.3989cm-1

C -0.054089 0.616810 1.413587
H -0.938594 0.016030 1.156996
H 0.277505 0.275366 2.408313
C -0.497934 2.077900 1.466238
H 0.293875 2.705672 1.905783
H -0.646856 2.448215 0.436795
C -1.781364 2.277194 2.271429
H -2.595324 1.696353 1.802289
H -1.649280 1.851800 3.282075
C -2.196061 3.739127 2.382279
H -2.353166 4.185012 1.386965
H -1.421588 4.335787 2.890918
H -3.131321 3.853949 2.951726
C 2.751890 1.078512 0.676297
C 2.669381 2.517851 0.100122
H 1.774608 3.032712 0.476971
H 2.552856 2.462256 -0.994042
C 3.920522 3.333110 0.457952
H 3.811379 4.347975 0.043225
C 4.071675 3.409793 1.982898
H 3.197890 3.917517 2.425555
H 4.959841 4.007273 2.250050
C 4.198179 1.989962 2.550447
H 4.302409 2.036085 3.646920
C 5.428774 1.302520 1.943274
H 6.344083 1.857535 2.209960
H 5.537351 0.285700 2.357747
C 5.274787 1.236618 0.417319
H 6.151166 0.733690 -0.022792
C 4.015367 0.426359 0.071276
H 3.901528 0.348123 -1.022940
H 4.145546 -0.594327 0.458150
C 5.155423 2.656582 -0.148543
H 6.064864 3.236876 0.083487
H 5.064570 2.620688 -1.246640
C 2.932106 1.186031 2.206924
H 2.998601 0.189438 2.665254
H 2.062835 1.686331 2.660002
C 1.385244 -1.682052 0.411310
C -0.027382 -2.321124 0.397423
H -0.620210 -1.957449 1.249626
H -0.559978 -2.018616 -0.518542
C 0.046709 -3.852930 0.492683
H -0.978066 -4.259891 0.473005
C 0.726942 -4.233946 1.814756
H 0.135229 -3.857003 2.666007
H 0.776765 -5.331025 1.918182
C 2.138549 -3.635192 1.844902
H 2.632113 -3.896303 2.794944
C 2.953159 -4.193376 0.669429
H 3.045387 -5.289168 0.757512
H 3.976554 -3.782284 0.689100
C 2.264099 -3.822217 -0.651380
H 2.852073 -4.211472 -1.498163
C 2.173924 -2.294738 -0.771884
H 1.682626 -2.010210 -1.716601
H 3.187305 -1.875893 -0.820225
C 2.063850 -2.101357 1.732148
H 3.084704 -1.698136 1.786887
H 1.509945 -1.692310 2.593315
C 0.852535 -4.418327 -0.682371
H 0.360486 -4.168218 -1.637851
H 0.895081 -5.519055 -0.620785
P 1.185636 0.180586 0.110003
Pd 0.559045 0.732500 -1.955854
Br -0.205834 1.427833 -4.257795
H -1.558914 -0.145592 -3.758659
N -2.174836 -0.960434 -3.495919
C -2.760354 -0.626127 -2.179298
H -1.930909 -0.389655 -1.495730
H -3.395152 0.260808 -2.289059
H -3.349928 -1.476852 -1.813285
C -1.271828 -2.129712 -3.416955
H -0.852480 -2.323377 -4.411116
H -0.455541 -1.874399 -2.727405
H -1.832951 -3.003443 -3.060774
C -3.194564 -1.100293 -4.553877
H -3.785497 -0.178046 -4.602582
H -2.690465 -1.259126 -5.514603
H -3.846977 -1.953023 -4.325657

int-9.log

SCF (RwB97XD) = -4155.82305872
E(SCF)+ZPE(0 K)= -4155.091701
H(298 K)= -4155.058412
G(298 K)= -4155.154865
Lowest Frequency = 18.7517cm-1

C 0.225344 -0.160174 2.064222
H -0.351750 -1.000085 1.655079
H 0.915800 -0.584740 2.808828
C -0.757995 0.802136 2.728545
H -0.219065 1.572224 3.302755
H -1.325614 1.339259 1.949363
C -1.733111 0.084787 3.661071
H -2.301790 -0.663239 3.081611
H -1.163633 -0.484111 4.416644
C -2.699361 1.034878 4.357050
H -3.304819 1.594395 3.625817
H -2.161046 1.773227 4.972992
H -3.392807 0.493119 5.018122
C 2.336234 1.761942 1.374276
C 1.556908 3.103858 1.406661
H 0.632322 2.999413 1.993523
H 1.261042 3.375163 0.382781
C 2.423250 4.221001 2.006417
H 1.833350 5.151248 2.021532
C 2.841147 3.846435 3.434188
H 1.949360 3.725521 4.072547
H 3.447109 4.653664 3.878663
C 3.645788 2.540355 3.400143
H 3.941042 2.258461 4.423809
C 4.896849 2.728843 2.533407
H 5.540613 3.514892 2.962530
H 5.490537 1.799023 2.519002
C 4.470068 3.105491 1.109104
H 5.361143 3.228172 0.472979
C 3.606900 1.978101 0.521227
H 3.321774 2.222164 -0.512611
H 4.209285 1.058700 0.496108
C 3.671224 4.413810 1.136193
H 4.293708 5.231747 1.536852
H 3.377614 4.697560 0.112870
C 2.771286 1.415929 2.818840
H 3.329628 0.468159 2.840514

H	1.895271	1.287831	3.470610
C	2.098835	-1.037746	0.016487
C	1.057422	-2.152600	-0.267325
H	0.533014	-2.439571	0.655760
H	0.296459	-1.777654	-0.971046
C	1.739098	-3.405520	-0.838757
H	0.967069	-4.170201	-1.020352
C	2.759133	-3.932327	0.179928
H	2.250181	-4.209027	1.118654
H	3.244102	-4.845453	-0.203357
C	3.811689	-2.849227	0.448679
H	4.544703	-3.217785	1.184109
C	4.526196	-2.491455	-0.861997
H	5.048366	-3.376491	-1.262292
H	5.294397	-1.721895	-0.677460
C	3.498916	-1.976236	-1.879626
H	4.008767	-1.706605	-2.817775
C	2.805322	-0.723774	-1.325201
H	2.068235	-0.346846	-2.050425
H	3.545158	0.076088	-1.202690
C	3.133169	-1.592185	1.020315
H	3.904339	-0.838447	1.234491
H	2.646163	-1.837934	1.977968
C	2.451245	-3.061587	-2.151407
H	1.722135	-2.703525	-2.897075
H	2.928761	-3.962443	-2.572155
P	1.180521	0.479570	0.628569
H	-1.111010	0.348408	-0.199147
N	-1.910422	1.670183	-2.374747
C	-2.147213	3.120281	-2.406921
H	-2.372765	3.478371	-1.393180
H	-1.246055	3.631406	-2.766912
H	-2.998857	3.363818	-3.069942
C	-3.127022	0.996613	-1.896964
H	-2.967103	-0.088450	-1.869720
H	-3.370011	1.338388	-0.883096
H	-3.983128	1.215571	-2.563364
C	-1.593348	1.186899	-3.725724
H	-0.693028	1.693495	-4.094025
H	-1.399750	0.105878	-3.694536
H	-2.434377	1.379411	-4.418333
Pd	-0.176865	1.244797	-1.015464
Br	1.423090	2.693208	-2.458741

int-s1-cf3.log

SCF (RwB97XD) = -4662.61169390
E(SCF)+ZPE(0 K)= -4661.908042
H(298 K)= -4661.869851
G(298 K)= -4661.978817
Lowest Frequency = 13.9515cm-1

C	-1.689740	-1.744596	0.651886
H	-2.642866	-2.057178	0.198437
H	-0.910271	-2.364944	0.190228
C	-1.692834	-2.039292	2.154290
H	-0.902995	-1.466484	2.658950
H	-2.641378	-1.716964	2.611176
C	-1.475666	-3.523487	2.441986
H	-2.255076	-4.114285	1.929719
H	-0.514091	-3.832607	1.999117
C	-1.475593	-3.844923	3.929831
H	-0.682594	-3.288097	4.454588
H	-1.303254	-4.916282	4.110927
H	-2.435293	-3.575814	4.400233
C	-1.528135	-0.159949	-1.775053
C	-0.637268	-1.318257	-2.291937
H	-0.928466	-2.275182	-1.835106
H	0.409876	-1.122638	-2.011667
C	-0.743023	-1.453460	-3.818958
H	-0.103209	-2.291148	-4.139141
C	-2.201021	-1.739070	-4.201364
H	-2.537107	-2.684069	-3.742147
H	-2.289392	-1.863635	-5.293138
C	-3.084433	-0.577965	-3.728146
H	-4.134464	-0.776551	-3.995607
C	-2.619603	0.728530	-4.385549
H	-2.717832	0.658241	-5.481577
H	-3.259514	1.565162	-4.058516
C	-1.159169	0.998888	-4.001288
H	-0.822249	1.943202	-4.456648
C	-1.037142	1.129381	-2.475523
H	0.010659	1.324258	-2.204140
H	-1.605321	2.004314	-2.137476
C	-0.277162	-0.155897	-4.488395
H	-0.338498	-0.250541	-5.585241
H	0.778499	0.042960	-4.240006
C	-2.987604	-0.438889	-2.199475
H	-3.641236	0.384160	-1.878279
H	-3.363687	-1.357719	-1.721928
C	-2.606654	1.049942	0.885641
C	-3.977611	0.345352	1.036244
H	-4.391463	0.093521	0.049701
H	-3.879930	-0.596320	1.591665
C	-4.966484	1.254465	1.786676
H	-5.926765	0.722050	1.878149
C	-5.169412	2.559383	1.007133
H	-5.590217	2.344746	0.010216
H	-5.894825	3.204547	1.530095
C	-3.820737	3.275658	0.869161
H	-3.947898	4.205526	0.292629
C	-3.266177	3.604147	2.260719
H	-3.959378	4.273618	2.796881
H	-2.304887	4.135296	2.169526
C	-3.072624	2.301092	3.045402
H	-2.665055	2.524047	4.044194
C	-2.072603	1.408084	2.297701
H	-1.886663	0.495000	2.881611
H	-1.110325	1.934556	2.200621
C	-2.836538	2.369035	0.112531
H	-1.881585	2.891619	-0.033446
H	-3.254761	2.155550	-0.881676
C	-4.414491	1.570070	3.182985
H	-4.282358	0.638075	3.758314
H	-5.131965	2.193493	3.741881
P	-1.305053	-0.042066	0.086922
Pd	0.936347	0.634584	0.569625
Br	0.767914	3.127507	0.022134
C	1.439791	-1.235095	1.196030
C	1.594794	-1.454256	2.573932
C	1.769039	-2.281545	0.326359
C	2.018527	-2.684931	3.068442
H	1.379527	-0.652910	3.285790
C	2.192024	-3.519517	0.811041
H	1.697959	-2.146142	-0.753901
C	2.314308	-3.724834	2.184791
H	2.123095	-2.831722	4.145335
H	2.432004	-4.322602	0.111506
C	2.816025	1.078525	0.885241
O	3.898757	1.347475	1.049401
C	2.695303	-5.077078	2.721388
F	3.396061	-4.996425	3.866723
F	3.438947	-5.785550	1.853848
F	1.606485	-5.831582	2.995285

int-s1-cl.log

SCF (RwB97XD) = -4785.16006896
E(SCF)+ZPE(0 K)= -4784.470873
H(298 K)= -4784.435022
G(298 K)= -4784.537973
Lowest Frequency = 21.3050cm-1

C	-1.695261	-1.745130	0.653263
H	-2.649038	-2.056761	0.200342
H	-0.915991	-2.367317	0.193628
C	-1.698804	-2.036938	2.156016
H	-0.901117	-1.471836	2.657068
H	-2.642717	-1.703723	2.614683
C	-1.496984	-3.522625	2.446848

H	-2.281732	-4.107001	1.935231
H	-0.537851	-3.842006	2.005703
C	-1.501568	-3.840024	3.935620
H	-0.712130	-3.279121	4.461343
H	-1.325318	-4.910501	4.119856
H	-2.463771	-3.573533	4.402278
C	-1.535737	-0.162101	-1.775020
C	-0.652883	-1.325699	-2.293437
H	-0.948501	-2.280683	-1.835375
H	0.395721	-1.135965	-2.015173
C	-0.762335	-1.460865	-3.820143
H	-0.128085	-2.302452	-4.141228
C	-2.222669	-1.737825	-4.199936
H	-2.563637	-2.680659	-3.739858
H	-2.313811	-1.862024	-5.291555
C	-3.098181	-0.571399	-3.725155
H	-4.149900	-0.763655	-3.990675
C	-2.626761	0.732189	-4.383708
H	-2.727564	0.662270	-5.479556
H	-3.260990	1.572735	-4.055628
C	-1.163989	0.993965	-4.002296
H	-0.822268	1.936057	-4.458706
C	-1.038468	1.124159	-2.476789
H	0.010921	1.312989	-2.207042
H	-1.600870	2.002555	-2.137971
C	-0.289910	-0.166293	-4.490783
H	-0.353828	-0.260753	-5.587525
H	0.767373	0.026112	-4.244293
C	-2.997489	-0.432758	-2.196697
H	-3.646018	0.393862	-1.874426
H	-3.377806	-1.349432	-1.718282
C	-2.603466	1.052677	0.886153
C	-3.977031	0.353800	1.039059
H	-4.393578	0.103474	0.053289
H	-3.882347	-0.588272	1.594312
C	-4.961189	1.266974	1.790740
H	-5.923453	0.738409	1.883947
C	-5.160161	2.572316	1.010859
H	-5.583234	2.358737	0.014659
H	-5.882370	3.220543	1.534528
C	-3.808895	3.283318	0.870564
H	-3.933338	4.213423	0.293785
C	-3.251276	3.610345	2.261232
H	-3.941309	4.282562	2.798105
H	-2.288142	4.137848	2.168496
C	-3.061436	2.306867	3.046103
H	-2.651485	2.528717	4.044180
C	-2.066004	1.409751	2.297235
H	-1.882264	0.496423	2.881426
H	-1.101961	1.932568	2.198170
C	-2.829141	2.372565	0.113071
H	-1.872176	2.891228	-0.034070
H	-3.249356	2.160748	-0.880644
C	-4.405855	1.581039	3.186066
H	-4.276324	0.648716	3.761484
H	-5.120167	2.207347	3.745840
P	-1.306177	-0.044102	0.086282
Pd	0.938837	0.625261	0.564415
Br	0.778237	3.121692	0.022841
C	1.441847	-1.251729	1.178278
C	1.595249	-1.487983	2.552553
C	1.773226	-2.289710	0.300053
C	2.015786	-2.725530	3.039449
H	1.380604	-0.695765	3.275168
C	2.194028	-3.536460	0.768756
H	1.706515	-2.145099	-0.779600
C	2.303956	-3.748434	2.139417
H	2.120620	-2.893065	4.112828
H	2.437875	-4.337011	0.068028
C	2.819464	1.052860	0.882912
O	3.904808	1.310091	1.049917
Cl	2.805804	-5.306052	2.736318

int-s1-cn.log

SCF (RwB97XD) = -4417.75039686
E(SCF)+ZPE(0 K)= -4417.052561
H(298 K)= -4417.016257
G(298 K)= -4417.119659
Lowest Frequency = 24.4893cm-1

C	-1.708865	-1.747008	0.657283
H	-2.664771	-2.054137	0.206062
H	-0.934614	-2.373833	0.195343
C	-1.711328	-2.036726	2.160565
H	-0.919912	-1.463679	2.662735
H	-2.658377	-1.710239	2.617418
C	-1.497196	-3.520037	2.453654
H	-2.277195	-4.111690	1.943469
H	-0.536365	-3.831797	2.010470
C	-1.497415	-3.835869	3.942714
H	-0.715034	-3.265059	4.468470
H	-1.309218	-4.904013	4.128296
H	-2.462544	-3.580078	4.409048
C	-1.535556	-0.165810	-1.771977
C	-0.653372	-1.331112	-2.287752
H	-0.950953	-2.285422	-1.829568
H	0.395187	-1.142085	-2.008540
C	-0.760510	-1.467354	-3.814586
H	-0.127099	-2.310233	-4.133810
C	-2.220641	-1.742334	-4.196526
H	-2.563953	-2.684250	-3.736378
H	-2.309951	-1.867306	-5.288139
C	-3.095121	-0.574117	-3.724345
H	-4.146639	-0.764916	-3.991440
C	-2.620480	0.728250	-4.382918
H	-2.719348	0.657668	-5.478836
H	-3.253922	1.570078	-4.056695
C	-1.157979	0.987924	-3.999186
H	-0.813930	1.929141	-4.455562
C	-1.034977	1.119236	-2.473544
H	0.014249	1.306584	-2.202243
H	-1.596822	1.998663	-2.136564
C	-0.284858	-0.174031	-4.485287
H	-0.347156	-0.269212	-5.581987
H	0.772378	0.016861	-4.237357
C	-2.997172	-0.434687	-2.195788
H	-3.645072	0.392998	-1.875008
H	-3.379468	-1.350634	-1.717608
C	-2.605844	1.054998	0.887522
C	-3.980980	0.358864	1.039829
H	-4.396435	0.107069	0.053966
H	-3.888911	-0.581937	1.597644
C	-4.964423	1.275533	1.788233
H	-5.927661	0.748784	1.880911
C	-5.159767	2.579718	1.005636
H	-5.581907	2.365236	0.009266
H	-5.881372	3.230254	1.527149
C	-3.806942	3.287768	0.865984
H	-3.928605	4.216973	0.287248
C	-3.250282	3.616159	2.256703
H	-3.939465	4.290809	2.791477
H	-2.285915	4.141494	2.164245
C	-3.064407	2.313803	3.044359
H	-2.655353	2.536654	4.042545
C	-2.069690	1.413034	2.298821
H	-1.889729	0.500168	2.884935
H	-1.104303	1.933544	2.201039
C	-2.828073	2.373722	0.111324
H	-1.870242	2.890573	-0.035872
H	-3.247604	2.160490	-0.882326
C	-4.410512	1.591062	3.183765
H	-4.283927	0.659680	3.761317
H	-5.124241	2.220028	3.741168
P	-1.312812	-0.048204	0.089788
Pd	0.932844	0.607484	0.577319
Br	0.781473	3.098598	0.029955
C	1.429245	-1.267887	1.190754
C	1.581154	-1.502934	2.567501
C	1.770076	-2.301360	0.307521

C 2.018478 -2.731820 3.050413
H 1.354087 -0.712841 3.287938
C 2.207601 -3.538736 0.773967
H 1.698842 -2.152507 -0.770835
C 2.329725 -3.761726 2.151432
H 2.124399 -2.896441 4.124366
H 2.461350 -4.333398 0.069714
C 2.813527 1.051389 0.900799
O 3.894911 1.322096 1.069588
C 2.768793 -5.040007 2.640799
N 3.113107 -6.065964 3.033435

int-s1-h.log

SCF (RwB97XD) = -4325.54470367
E(SCF)+ZPE(0 K)= -4324.845801
H(298 K)= -4324.811247
G(298 K)= -4324.910445
Lowest Frequency = 27.1661cm-1

C -1.697565 -1.747750 0.654221
H -2.649017 -2.063135 0.198745
H -0.913355 -2.367882 0.199988
C -1.704792 -2.035614 2.157524
H -0.904345 -1.473335 2.657288
H -2.648098 -1.697755 2.614133
C -1.508942 -3.521155 2.451991
H -2.291211 -4.104413 1.935015
H -0.545971 -3.841793 2.020148
C -1.527592 -3.835203 3.941469
H -0.737035 -3.280191 4.471651
H -1.363592 -4.907059 4.130019
H -2.490930 -3.558961 4.400172
C -1.540053 -0.163464 -1.773878
C -0.661570 -1.330098 -2.292545
H -0.961365 -2.284133 -1.835231
H 0.387215 -1.145272 -2.012477
C -0.769980 -1.463636 -3.819402
H -0.138702 -2.307467 -4.140541
C -2.230982 -1.734382 -4.201118
H -2.576210 -2.676267 -3.742228
H -2.321648 -1.857067 -5.293009
C -3.102292 -0.564997 -3.725866
H -4.154551 -0.752722 -3.992665
C -2.625006 0.737310 -4.382853
H -2.725180 0.668787 -5.478894
H -3.256101 1.580136 -4.054527
C -1.161512 0.992985 -3.999921
H -0.815619 1.934032 -4.455417
C -1.037044 1.121369 -2.474224
H 0.012745 1.305830 -2.202861
H -1.596133 2.001840 -2.135241
C -0.291672 -0.170438 -4.488545
H -0.354807 -0.263633 -5.585504
H 0.766077 0.017355 -4.240588
C -3.002310 -0.428204 -2.197151
H -3.648349 0.400342 -1.874845
H -3.386373 -1.343937 -1.719794
C -2.606181 1.050607 0.886148
C -3.981170 0.354306 1.037093
H -4.397211 0.105538 0.050717
H -3.888748 -0.588549 1.591422
C -4.964576 1.268672 1.788286
H -5.927975 0.741871 1.880327
C -5.160400 2.574687 1.008681
H -5.582525 2.362067 0.011847
H -5.882283 3.223920 1.531658
C -3.807778 3.283581 0.870287
H -3.930088 4.214140 0.293724
C -3.251603 3.609264 2.261828
H -3.941436 4.282212 2.798157
H -2.287584 4.135348 2.170520
C -3.064500 2.305087 3.046149
H -2.655321 2.525824 4.044818
C -2.069736 1.406773 2.297897
H -1.887444 0.493301 2.882186
H -1.104802 1.928069 2.199496
C -2.828331 2.371576 0.113861
H -1.869943 2.888420 -0.031080
H -3.247235 2.161652 -0.880797
C -4.410206 1.581274 3.184351
H -4.282583 0.648401 3.759342
H -5.124244 2.208333 3.743737
P -1.308396 -0.046889 0.087382
Pd 0.936266 0.621887 0.565251
Br 0.780452 3.126226 0.029382
C 1.444313 -1.257449 1.173361
C 1.595920 -1.495005 2.547772
C 1.788898 -2.284703 0.287147
C 2.038725 -2.731416 3.021374
H 1.366801 -0.705574 3.269549
C 2.231175 -3.524252 0.758851
H 1.719281 -2.130570 -0.791330
C 2.353933 -3.754652 2.127551
H 2.144080 -2.891472 4.097424
H 2.488645 -4.311278 0.045321
C 2.817971 1.037144 0.885177
O 3.905135 1.287111 1.053227
H 2.702403 -4.722092 2.496073

int-s1-me.log

SCF (RwB97XD) = -4364.82434792
E(SCF)+ZPE(0 K)= -4364.098179
H(298 K)= -4364.061729
G(298 K)= -4364.165643
Lowest Frequency = 25.3943cm-1

C -1.693375 -1.743414 0.651204
H -2.644184 -2.059637 0.195084
H -0.907880 -2.359996 0.194726
C -1.698089 -2.036663 2.153664
H -0.906036 -1.465255 2.656285
H -2.646946 -1.714645 2.610601
C -1.478567 -3.520487 2.440760
H -2.255670 -4.113467 1.927044
H -0.514253 -3.822341 1.999010
C -1.479044 -3.842151 3.928658
H -0.693502 -3.275646 4.454033
H -1.294751 -4.912026 4.110515
H -2.442912 -3.585651 4.397586
C -1.533398 -0.158847 -1.774442
C -0.647677 -1.321571 -2.290191
H -0.945779 -2.276993 -1.834699
H 0.399510 -1.133269 -2.005657
C -0.750406 -1.455387 -3.817452
H -0.114020 -2.296087 -4.136747
C -2.208965 -1.733482 -4.203473
H -2.550754 -2.677066 -3.745456
H -2.295793 -1.856840 -5.295612
C -3.087692 -0.568422 -3.731146
H -4.138166 -0.761598 -4.001156
C -2.614912 0.736038 -4.387103
H -2.711356 0.666694 -5.483436
H -3.251236 1.575771 -4.060936
C -1.153920 0.999105 -3.999693
H -0.811409 1.941859 -4.454236
C -1.034670 1.128198 -2.473685
H 0.013313 1.318019 -2.199269
H -1.599131 2.005903 -2.136429
C -0.276635 -0.159910 -4.485395
H -0.335915 -0.253688 -5.582537
H 0.779345 0.033428 -4.234183
C -2.993053 -0.430630 -2.202138
H -3.643887 0.395035 -1.881925
H -3.374319 -1.347970 -1.725638
C -2.610995 1.050878 0.883689
C -3.983358 0.349245 1.032621
H -4.396566 0.098013 0.045608
H -3.887590 -0.592841 1.587902

C -4.971597 1.260060 1.781775
H -5.933372 0.729945 1.871992
C -5.170567 2.565531 1.001973
H -5.589929 2.351575 0.004253
H -5.895858 3.212033 1.523633
C -3.820295 3.279441 0.866274
H -3.945037 4.209770 0.289846
C -3.267657 3.606682 2.258877
H -3.960664 4.277216 2.794160
H -2.305258 4.136019 2.169274
C -3.077420 2.302975 3.043269
H -2.670844 2.524931 4.042743
C -2.078279 1.408283 2.296496
H -1.894024 0.495011 2.880581
H -1.114783 1.932655 2.200034
C -2.836274 2.371173 0.111401
H -1.879259 2.891241 -0.031568
H -3.252515 2.159958 -0.884142
C -4.420961 1.574597 3.178917
H -4.291264 0.642185 3.754193
H -5.138156 2.199271 3.736957
P -1.307316 -0.040901 0.087539
Pd 0.934293 0.637133 0.569029
Br 0.773205 3.140401 0.026281
C 1.439682 -1.236543 1.196728
C 1.598595 -1.457951 2.572214
C 1.769274 -2.284312 0.331637
C 2.022666 -2.693402 3.061321
H 1.387725 -0.656047 3.286023
C 2.193057 -3.521767 0.824465
H 1.700603 -2.151598 -0.749919
C 2.319670 -3.754158 2.197161
H 2.129258 -2.833118 4.141276
H 2.434662 -4.321742 0.118240
C 2.815963 1.045379 0.885974
O 3.905257 1.288292 1.051484
C 2.723838 -5.101890 2.732859
H 1.835840 -5.707487 2.980449
H 3.314529 -5.669842 1.999460
H 3.320242 -5.008396 3.652471

int-s1-nme2.log

SCF (RwB97XD) = -4459.43898322
E(SCF)+ZPE(0 K)= -4458.666942
H(298 K)= -4458.628023
G(298 K)= -4458.737304
Lowest Frequency = 18.5137cm-1

C -1.610204 -1.715710 0.657989
H -2.564780 -2.057077 0.228918
H -0.821255 -2.318194 0.188778
C -1.570909 -1.994749 2.162509
H -0.769614 -1.411191 2.635944
H -2.509804 -1.677507 2.642995
C -1.328928 -3.473516 2.457859
H -2.114999 -4.079515 1.973617
H -0.375035 -3.771405 1.992358
C -1.282756 -3.779793 3.948235
H -0.493166 -3.193451 4.445075
H -1.073361 -4.844296 4.134580
H -2.237511 -3.535750 4.442153
C -1.534583 -0.146779 -1.783365
C -0.607196 -1.271871 -2.308924
H -0.849481 -2.235939 -1.838593
H 0.435298 -1.030903 -2.048057
C -0.734709 -1.423704 -3.832537
H -0.067635 -2.237690 -4.158599
C -2.186739 -1.770213 -4.185953
H -2.475496 -2.724148 -3.713026
H -2.290535 -1.907667 -5.274961
C -3.107622 -0.641406 -3.705168
H -4.153470 -0.884233 -3.952624
C -2.707124 0.676933 -4.380791
H -2.822633 0.594035 -5.474410
H -3.374390 1.489632 -4.047759
C -1.251727 1.008333 -4.024783
H -0.961645 1.961762 -4.493407
C -1.106868 1.155164 -2.502298
H -0.063012 1.393169 -2.250554
H -1.703289 2.009405 -2.161109
C -0.333110 -0.113986 -4.519991
H -0.409725 -0.219518 -5.615046
H 0.717785 0.129273 -4.291605
C -2.988848 -0.484777 -2.179846
H -3.667039 0.316420 -1.853668
H -3.321580 -1.412423 -1.687434
C -2.594284 1.054534 0.885173
C -3.946123 0.321281 1.066186
H -4.371638 0.051400 0.089273
H -3.817428 -0.613563 1.627238
C -4.942090 1.214719 1.826367
H -5.889130 0.662735 1.939251
C -5.187420 2.508839 1.040672
H -5.620889 2.277228 0.052978
H -5.917565 3.142431 1.571373
C -3.857409 3.253403 0.873281
H -4.015632 4.175408 0.291622
C -3.286534 3.605833 2.252355
H -3.985426 4.264338 2.794991
H -2.339043 4.157308 2.140254
C -3.050086 2.313649 3.043359
H -2.630189 2.553655 4.033136
C -2.043784 1.437426 2.284429
H -1.825340 0.534212 2.872213
H -1.095672 1.984386 2.164614
C -2.867702 2.361703 0.106486
H -1.926586 2.902876 -0.062298
H -3.300600 2.130051 -0.877500
C -4.372928 1.554331 3.210229
H -4.209702 0.630201 3.790326
H -5.094406 2.166169 3.777019
P -1.276400 -0.008966 0.073217
Pd 0.959282 0.723374 0.504647
Br 0.728351 3.222861 -0.034961
C 1.522921 -1.142966 1.115890
C 1.732005 -1.374416 2.481061
C 1.843259 -2.189394 0.247506
C 2.173454 -2.604162 2.965632
H 1.544608 -0.578028 3.207860
C 2.284657 -3.428678 0.712595
H 1.751887 -2.058866 -0.832670
C 2.436898 -3.679757 2.091347
H 2.311637 -2.715954 4.040790
H 2.509125 -4.203227 -0.020284
C 2.837333 1.142956 0.790248
O 3.929403 1.385395 0.940987
N 2.817510 -4.922963 2.564400
C 3.158602 -5.072571 3.959475
H 2.310737 -4.802929 4.608906
H 3.398993 -6.123309 4.163285
H 4.028750 -4.457934 4.259823
C 3.301712 -5.912937 1.631159
H 2.534032 -6.167847 0.883190
H 4.210230 -5.586435 1.089771
H 3.543344 -6.835208 2.173541

int-s1-ome.log

SCF (RwB97XD) = -4440.03678237
E(SCF)+ZPE(0 K)= -4439.305170
H(298 K)= -4439.268061
G(298 K)= -4439.373193
Lowest Frequency = 22.1193cm-1

C -1.690482 -1.745847 0.647385
H -2.643174 -2.060520 0.193924
H -0.906637 -2.363497 0.189473
C -1.692403 -2.039408 2.149512
H -0.886455 -1.483611 2.647635

H	-2.631883	-1.698703	2.611986
C	-1.503114	-3.527444	2.436854
H	-2.292558	-4.103984	1.923036
H	-0.545448	-3.851442	1.995831
C	-1.511263	-3.848447	3.924862
H	-0.713428	-3.299821	4.450422
H	-1.349375	-4.921664	4.106967
H	-2.469784	-3.570252	4.392456
C	-1.539166	-0.160461	-1.778659
C	-0.658662	-1.325054	-2.298981
H	-0.957620	-2.279937	-1.842793
H	0.390184	-1.139520	-2.018639
C	-0.767968	-1.457308	-3.825924
H	-0.135023	-2.299348	-4.148679
C	-2.228766	-1.731263	-4.206390
H	-2.571265	-2.674369	-3.747897
H	-2.320245	-1.853297	-5.298302
C	-3.102538	-0.564377	-3.729394
H	-4.154580	-0.754636	-3.995309
C	-2.629141	0.739417	-4.386229
H	-2.730361	0.671278	-5.482215
H	-3.261941	1.580484	-4.056697
C	-1.165925	0.998432	-4.004558
H	-0.822777	1.940627	-4.459763
C	-1.040112	1.125893	-2.478970
H	0.009452	1.312917	-2.208687
H	-1.600963	2.004691	-2.138495
C	-0.293546	-0.162458	-4.494670
H	-0.357333	-0.255235	-5.591645
H	0.763889	0.028046	-4.247433
C	-3.001176	-0.428177	-2.200706
H	-3.648456	0.398941	-1.877140
H	-3.382944	-1.344877	-1.723359
C	-2.604045	1.049362	0.884041
C	-3.977502	0.350676	1.036840
H	-4.395250	0.102782	0.050916
H	-3.882181	-0.592939	1.589517
C	-4.960669	1.262334	1.791600
H	-5.923233	0.734165	1.884750
C	-5.159853	2.569503	1.014693
H	-5.583781	2.358064	0.018357
H	-5.881533	3.216841	1.540313
C	-3.808516	3.280589	0.874708
H	-3.933429	4.212103	0.300230
C	-3.249874	3.604448	2.265723
H	-3.939457	4.275590	2.804652
H	-2.286707	4.131913	2.173343
C	-3.059382	2.299132	3.047382
H	-2.648444	2.518626	4.045596
C	-2.064976	1.403695	2.295288
H	-1.879662	0.489326	2.877307
H	-1.101050	1.926671	2.195514
C	-2.829415	2.371312	0.114586
H	-1.871847	2.889527	-0.031339
H	-3.249933	2.162530	-0.879715
C	-4.403812	1.573234	3.187062
H	-4.273708	0.639513	3.760110
H	-5.117556	2.198310	3.749062
P	-1.304710	-0.043462	0.082410
Pd	0.939441	0.632240	0.554330
Br	0.780709	3.135202	0.014713
C	1.446062	-1.244552	1.176289
C	1.612853	-1.470671	2.553518
C	1.766991	-2.291981	0.313719
C	2.032261	-2.701676	3.043916
H	1.407898	-0.670179	3.270456
C	2.189617	-3.540955	0.789189
H	1.691889	-2.160399	-0.767534
C	2.315514	-3.753702	2.163050
H	2.150122	-2.867208	4.116863
H	2.418799	-4.328875	0.071262
O	2.703148	-4.923982	2.733170
C	2.993762	-6.013638	1.887335
H	3.274667	-6.849184	2.541167
H	2.115828	-6.312468	1.289117
H	3.836867	-5.794044	1.210485
C	2.823076	1.029828	0.865714
O	3.915228	1.262346	1.027788

int-s2-ome.log

SCF (RwB97XD) = -4441.23077648
E(SCF)+ZPE(0 K)= -4440.482085
H(298 K)= -4440.444287
G(298 K)= -4440.550780
Lowest Frequency = 15.7767cm-1

C	0.201186	-0.528121	1.868444
H	-0.477334	-1.387963	1.942264
H	1.024814	-0.725481	2.572121
C	-0.567684	0.729375	2.281517
H	0.128254	1.550263	2.512457
H	-1.187623	1.090015	1.449643
C	-1.471212	0.490159	3.488868
H	-2.176499	-0.324701	3.251092
H	-0.864704	0.132008	4.339066
C	-2.250039	1.735147	3.891894
H	-2.871948	2.096495	3.057118
H	-1.574530	2.556351	4.181456
H	-2.916801	1.535948	4.744746
C	2.049450	0.838463	0.021089
C	1.201582	2.070393	-0.392299
H	0.406647	2.256932	0.344618
H	0.712218	1.865433	-1.356800
C	2.086031	3.318698	-0.522193
H	1.448739	4.171621	-0.805210
C	2.775698	3.606429	0.817623
H	2.021727	3.795904	1.600464
H	3.393333	4.516567	0.739688
C	3.649783	2.406900	1.206511
H	4.141472	2.603069	2.172921
C	4.709650	2.168590	0.124186
H	5.361583	3.053007	0.029216
H	5.356338	1.320808	0.407162
C	4.008248	1.877044	-1.207928
H	4.758169	1.685055	-1.991245
C	3.132343	0.622490	-1.061359
H	2.658490	0.382355	-2.023049
H	3.779673	-0.222942	-0.788161
C	3.141304	3.076670	-1.608399
H	3.770213	3.973772	-1.736201
H	2.649997	2.882017	-2.575130
C	2.767083	1.155778	1.356988
H	3.389768	0.306610	1.672043
H	2.043054	1.337131	2.161917
C	1.767882	-2.263029	0.141982
C	0.781106	-3.358629	0.623257
H	0.461451	-3.164506	1.657343
H	-0.127465	-3.349167	0.002425
C	1.444291	-4.744308	0.579444
H	0.711987	-5.489374	0.928397
C	2.668742	-4.747799	1.504121
H	2.360964	-4.539646	2.542839
H	3.145505	-5.742120	1.503578
C	3.665953	-3.685481	1.025031
H	4.545253	-3.673755	1.688541
C	4.103522	-4.000097	-0.411772
H	4.611331	-4.978257	-0.447480
H	4.831017	-3.247581	-0.759798
C	2.872926	-4.007574	-1.328648
H	3.181877	-4.220199	-2.364085
C	2.195030	-2.630315	-1.299478
H	1.313048	-2.637490	-1.957222
H	2.876203	-1.877487	-1.714528
C	3.008156	-2.295273	1.063043
H	3.746782	-1.547171	0.743333
H	2.725655	-2.050737	2.099840
C	1.879244	-5.073222	-0.852997
H	1.002191	-5.096671	-1.520411
H	2.341016	-6.074053	-0.890538

P	0.867913	-0.615755	0.159541
Pd	-0.735595	-0.650451	-1.533981
Br	0.648870	-0.149810	-3.677120
C	-3.115325	0.048160	0.162698
C	-3.016635	1.330532	-0.400759
C	-4.134966	-0.197261	1.088242
C	-3.903255	2.332613	-0.047403
H	-2.221143	1.551034	-1.118060
C	-5.030958	0.799839	1.459153
H	-4.217559	-1.192650	1.529654
H	-3.828252	3.330709	-0.481962
H	-5.807653	0.575148	2.189573
C	-2.148963	-1.050540	-0.161232
O	-2.298594	-2.165182	0.278961
H	-2.346477	-0.942207	-2.516169
H	-1.838520	-0.688354	-3.056850
C	-4.915317	2.077184	0.892455
O	-5.719702	3.115949	1.189824
C	-6.731593	2.934256	2.161541
H	-7.241567	3.900374	2.259191
H	-7.464463	2.173684	1.846682
H	-6.306591	2.653658	3.139135

int-s3-ome.log

SCF (RwB97XD) = -4441.21793946
E(SCF)+ZPE(0 K)= -4440.470211
H(298 K)= -4440.432093
G(298 K)= -4440.541268
Lowest Frequency = 11.3970cm-1

C	1.788490	0.417270	0.436056
H	1.352591	-0.470095	0.918166
H	2.869911	0.377827	0.642184
C	1.163059	1.653960	1.083458
H	1.715428	2.560294	0.789190
H	0.133247	1.794749	0.710769
C	1.135892	1.567245	2.608744
H	0.552416	0.679603	2.910206
H	2.161185	1.396523	2.980021
C	0.551203	2.810707	3.266225
H	-0.484841	2.988399	2.935366
H	1.136766	3.709576	3.014879
H	0.538430	2.719149	4.362787
C	2.315521	1.600632	-2.215364
C	1.318197	2.790135	-2.189643
H	1.052815	3.050701	-1.154962
H	0.388722	2.494180	-2.698460
C	1.918382	4.019752	-2.887377
H	1.190002	4.844629	-2.834033
C	3.220343	4.430676	-2.187029
H	3.018745	4.699016	-1.135948
H	3.647142	5.325235	-2.670798
C	4.220155	3.268539	-2.250828
H	5.156544	3.555567	-1.745834
C	4.509505	2.919012	-3.716727
H	4.959599	3.784692	-4.230668
H	5.241439	2.095375	-3.772743
C	3.202196	2.511763	-4.408698
H	3.403272	2.244667	-5.458253
C	2.613798	1.282503	-3.698886
H	1.689831	0.960763	-4.202995
H	3.338230	0.459158	-3.775050
C	2.205360	3.675561	-4.353664
H	2.617631	4.553402	-4.879069
H	1.269696	3.398690	-4.866060
C	3.628392	2.044704	-1.531011
H	4.362544	1.225705	-1.532869
H	3.446014	2.308335	-0.478371
C	2.293593	-1.469724	-1.724338
C	1.768480	-2.510724	-0.700360
H	2.068015	-2.234506	0.321488
H	0.665117	-2.539134	-0.725326
C	2.332038	-3.908821	-1.000955
H	1.937490	-4.612987	-0.251065
C	3.863411	-3.861274	-0.911729
H	4.176351	-3.568829	0.104914
H	4.285852	-4.861720	-1.102730
C	4.399639	-2.855706	-1.938457
H	5.499017	-2.812788	-1.877979
C	3.974766	-3.285856	-3.349766
H	4.406360	-4.271387	-3.591453
H	4.364683	-2.572617	-4.095392
C	2.442834	-3.344842	-3.428503
H	2.133001	-3.642652	-4.442588
C	1.858167	-1.957574	-3.128392
H	0.759049	-1.995395	-3.182484
H	2.178712	-1.253100	-3.905546
C	3.834534	-1.455567	-1.641774
H	4.251652	-0.744145	-2.368886
H	4.161474	-1.124969	-0.641781
C	1.909789	-4.353653	-2.405473
H	0.810952	-4.413034	-2.470934
H	2.302406	-5.362000	-2.618604
P	1.460875	0.169627	-1.359555
Pd	-0.974442	-0.056715	-1.674631
Br	-1.154561	0.403010	-4.106310
C	-3.984397	0.548549	-1.811158
C	-3.654885	1.904540	-1.668584
C	-5.333869	0.189739	-1.893516
C	-4.643440	2.871053	-1.602039
H	-2.604277	2.201756	-1.621445
C	-6.338992	1.149099	-1.833151
H	-5.594887	-0.864605	-2.007858
H	-4.394276	3.928247	-1.496459
H	-7.380431	0.836085	-1.902789
C	-2.941530	-0.514066	-1.858619
O	-3.195350	-1.687974	-1.932083
H	-0.976379	-0.617586	-0.017977
H	-1.437812	0.062355	0.008613
C	-5.995040	2.500715	-1.687375
O	-6.887690	3.509713	-1.622195
C	-8.264221	3.208300	-1.740979
H	-8.794957	4.166515	-1.679476
H	-8.492900	2.734537	-2.709660
H	-8.609777	2.556815	-0.921608

int-s4-ome.log

SCF (RwB97XD) = -4615.60941197
E(SCF)+ZPE(0 K)= -4614.732466
H(298 K)= -4614.688418
G(298 K)= -4614.810718
Lowest Frequency = 10.4197cm-1

C	2.096296	0.434344	1.941187
H	1.733446	-0.349467	2.621287
H	3.196490	0.406424	1.990531
C	1.575469	1.771193	2.467703
H	2.043952	2.608748	1.927841
H	0.492292	1.845883	2.273838
C	1.834887	1.948733	3.963115
H	1.331655	1.135610	4.516362
H	2.913774	1.827269	4.164244
C	1.362384	3.295414	4.496153
H	0.282061	3.433836	4.328838
H	1.881564	4.127478	3.993949
H	1.549066	3.392351	5.576649
C	2.232176	1.164649	-0.902355
C	1.201510	2.324411	-0.958244
H	1.039114	2.747635	0.043816
H	0.233736	1.926089	-1.299816
C	1.672109	3.429817	-1.913758
H	0.925770	4.240437	-1.911037
C	3.030870	3.973418	-1.451406
H	2.942322	4.410152	-0.441817
H	3.365909	4.782301	-2.122421
C	4.059234	2.834301	-1.444155
H	5.036753	3.217959	-1.109044
C	4.189087	2.251291	-2.857565

H	4.543230	3.027217	-3.557210
H	4.940783	1.443485	-2.866322
C	2.826373	1.708967	-3.307610
H	2.912747	1.272264	-4.315326
C	2.372061	0.607098	-2.337244
H	1.410363	0.184303	-2.666018
H	3.118299	-0.200708	-2.358769
C	1.799378	2.847448	-3.326353
H	2.112899	3.630665	-4.037307
H	0.821624	2.469216	-3.665852
C	3.600971	1.736502	-0.468208
H	4.358364	0.939249	-0.432212
H	3.543903	2.166455	0.542566
C	2.358215	-1.773378	0.083749
C	1.944487	-2.645092	1.296270
H	2.308621	-2.201147	2.235243
H	0.842610	-2.678436	1.347413
C	2.516240	-4.066193	1.184460
H	2.204886	-4.644616	2.070514
C	4.048476	-3.984209	1.135141
H	4.434326	-3.524234	2.060870
H	4.481597	-4.996698	1.075042
C	4.473213	-3.153943	-0.083578
H	5.572675	-3.090806	-0.123232
C	3.945633	-3.817546	-1.363532
H	4.381614	-4.824493	-1.475520
H	4.254023	-3.233570	-2.246827
C	2.414430	-3.902826	-1.300561
H	2.028680	-4.364611	-2.222924
C	1.823505	-2.490648	-1.179707
H	0.724908	-2.536877	-1.142048
H	2.066909	-1.921618	-2.084365
C	3.898986	-1.730395	0.027114
H	4.230889	-1.144278	-0.842071
H	4.305858	-1.235274	0.924188
C	1.992844	-4.740084	-0.088557
H	0.893551	-4.822332	-0.054396
H	2.391472	-5.765488	-0.168249
P	1.508084	-0.106756	0.277211
Pd	-0.888625	-0.266253	0.259194
Br	-1.275425	-0.641337	-2.253670
C	-3.732863	0.853385	0.271815
C	-3.152371	2.128349	0.224681
C	-5.119097	0.750903	0.131522
C	-3.930380	3.263421	0.057149
H	-2.067134	2.219333	0.322773
C	-5.917091	1.878112	-0.048874
H	-5.575179	-0.241571	0.156671
H	-3.481191	4.257868	0.022612
H	-6.994151	1.758355	-0.165728
C	-2.895795	-0.380960	0.472298
O	-3.463370	-1.437643	0.729171
H	-0.896728	0.068367	1.803695
H	-1.553686	-1.458618	2.267335
N	-1.758458	-2.054416	3.103594
C	-2.984279	-1.504553	3.726911
H	-2.835964	-0.431096	3.897216
H	-3.823194	-1.648532	3.039270
H	-3.163939	-2.019092	4.679459
C	-0.591060	-1.909190	3.998878
H	0.302743	-2.272554	3.481452
H	-0.464634	-0.847766	4.242436
H	-0.760873	-2.490024	4.914095
C	-1.938691	-3.445539	2.633323
H	-2.751710	-3.453907	1.899912
H	-1.012090	-3.775951	2.150055
H	-2.168661	-4.091401	3.490451
C	-5.321054	3.145608	-0.083136
O	-6.001502	4.305111	-0.246790
C	-7.402797	4.248484	-0.404060
H	-7.743206	5.285137	-0.522071
H	-7.690340	3.675884	-1.301610
H	-7.896173	3.811780	0.480583

int-s5-ome.log

SCF (RwB97XD) = -4615.60438085
E(SCF)+ZPE(0 K)= -4614.729749
H(298 K)= -4614.686453
G(298 K)= -4614.803778
Lowest Frequency = 19.5282cm-1

C	-2.321072	1.768833	-0.012492
C	-1.569160	2.512218	-0.938524
H	-0.943770	2.008661	-1.684471
C	-1.610499	3.894831	-0.930130
H	-1.030514	4.474302	-1.650357
C	-3.167658	3.841599	0.933170
H	-3.791907	4.343465	1.672039
C	-3.121534	2.451731	0.909248
H	-3.709199	1.876424	1.627689
C	0.218888	-0.030489	2.024093
H	-0.804877	-0.330053	2.285998
H	0.876419	-0.493553	2.776996
C	0.309325	1.495656	2.109708
H	1.361536	1.817021	2.142866
H	-0.120080	1.960006	1.211784
C	-0.418310	2.047446	3.333636
H	-0.016984	1.574829	4.247320
H	-1.481165	1.754504	3.278552
C	-0.312630	3.562729	3.442352
H	-0.720991	4.049618	2.542095
H	0.735550	3.886320	3.546739
H	-0.867553	3.944948	4.312776
C	0.469480	-2.682051	0.867262
C	0.398899	-3.552359	-0.412176
H	-0.478384	-3.252383	-1.007012
H	1.283386	-3.397514	-1.044728
C	0.271636	-5.040937	-0.057610
H	0.249521	-5.622395	-0.992704
C	-1.029133	-5.261016	0.722908
H	-1.895426	-4.968994	0.105721
H	-1.153483	-6.328702	0.968461
C	-0.988518	-4.424165	2.006576
H	-1.926090	-4.558353	2.568905
C	0.201605	-4.864557	2.869391
H	0.099618	-5.926707	3.147072
H	0.222918	-4.287246	3.808863
C	1.502103	-4.647679	2.086065
H	2.363341	-4.952395	2.701484
C	1.659081	-3.159626	1.728147
H	2.604181	-3.028085	1.182865
H	1.730089	-2.561972	2.651159
C	-0.837634	-2.933774	1.663680
H	-0.828629	-2.367083	2.605509
H	-1.706896	-2.579418	1.087087
C	1.470338	-5.476431	0.794960
H	1.395796	-6.550263	1.033393
H	2.408310	-5.336651	0.231620
C	2.246162	-0.318035	-0.114653
C	3.236689	-0.190950	1.068638
H	2.843813	0.496082	1.830691
H	3.387042	-1.161451	1.560925
C	4.593009	0.340712	0.573711
H	5.272546	0.416467	1.437888
C	5.179054	-0.628105	-0.461492
H	5.337479	-1.620427	-0.005371
H	6.164550	-0.269077	-0.801845
C	4.217075	-0.738947	-1.651033
H	4.621321	-1.446165	-2.392662
C	4.027728	0.638719	-2.296643
H	3.343202	0.569733	-3.156930
H	4.993884	1.015427	-2.672015
C	3.447491	1.603940	-1.256614
H	3.290749	2.591659	-1.716811
C	2.087444	1.072492	-0.783637
H	1.638869	1.790369	-0.083494
H	1.413644	1.001538	-1.651370
C	2.862415	-1.275657	-1.160325
H	3.016556	-2.273292	-0.723329

H	2.186868	-1.390275	-2.020040
C	4.403164	1.723722	-0.062763
H	5.376114	2.125790	-0.391067
H	3.997256	2.430238	0.681110
C	-2.264883	0.276412	0.050083
O	-2.961063	-0.356053	0.810602
P	0.532666	-0.855756	0.414251
Pd	-1.067095	-0.597415	-1.267715
H	0.067560	-1.598467	-2.444783
H	0.102115	-0.897242	-2.802423
C	-2.402276	4.571862	0.011970
O	-2.363300	5.917460	-0.042071
C	-3.124878	6.658782	0.890304
H	-2.815195	6.446286	1.926807
H	-2.933491	7.716578	0.671680
H	-4.203796	6.460755	0.781395
N	-2.806802	-0.826768	-2.693134
C	-3.368010	0.473678	-3.094979
H	-3.779518	0.993079	-2.220907
H	-2.572706	1.085970	-3.541072
H	-4.178349	0.331185	-3.832787
C	-3.847110	-1.643623	-2.050573
H	-3.424292	-2.605580	-1.730549
H	-4.245081	-1.131980	-1.167339
H	-4.676458	-1.837672	-2.754811
C	-2.334690	-1.526357	-3.901840
H	-1.565663	-0.919698	-4.398245
H	-1.911713	-2.502032	-3.627583
H	-3.174958	-1.692118	-4.599521
Br	0.274257	1.169952	-4.172183

int-s6-ome.log

SCF (RwB97XD) = -3981.43009112
E(SCF)+ZPE(0 K)= -3980.824407
H(298 K)= -3980.796708
G(298 K)= -3980.881543
Lowest Frequency = 28.6070cm-1

C	1.170659	0.229247	1.828578
H	0.908142	-0.685897	2.377795
H	2.145511	0.562722	2.219123
C	0.083560	1.268479	2.102996
H	0.379544	2.251033	1.702346
H	-0.840675	0.981394	1.572258
C	-0.215480	1.415677	3.594290
H	-0.551040	0.442557	3.992226
H	0.717750	1.660750	4.130962
C	-1.269036	2.476430	3.886040
H	-2.219948	2.243151	3.380935
H	-0.944536	3.470504	3.538253
H	-1.475026	2.552481	4.964403
C	1.934829	1.171894	-0.883205
C	0.657210	1.970515	-1.262938
H	0.095914	2.265160	-0.365951
H	-0.018068	1.335525	-1.865606
C	1.016505	3.219227	-2.082037
H	0.088480	3.764597	-2.313859
C	1.957490	4.116488	-1.267175
H	1.459386	4.448208	-0.340754
H	2.203630	5.024383	-1.842176
C	3.237436	3.339714	-0.930292
H	3.916753	3.978637	-0.344140
C	3.930844	2.899610	-2.226628
H	4.219743	3.782499	-2.820084
H	4.859146	2.351256	-1.993306
C	2.979879	2.007755	-3.036366
H	3.476947	1.677406	-3.961936
C	2.622410	0.764388	-2.206594
H	1.957984	0.103283	-2.787837
H	3.544427	0.201578	-2.005838
C	1.707715	2.790435	-3.381424
H	1.959154	3.674918	-3.989596
H	1.027560	2.166643	-3.984570
C	2.878140	2.103161	-0.089591

H	3.796560	1.570439	0.196048
H	2.395936	2.432566	0.843018
C	2.679015	-1.656060	0.183549
C	2.303916	-2.652631	1.310728
H	2.305039	-2.147751	2.287638
H	1.287706	-3.047911	1.145955
C	3.310057	-3.812683	1.371138
H	3.004180	-4.494966	2.179843
C	4.706797	-3.250843	1.666380
H	4.711223	-2.733823	2.640643
H	5.439987	-4.071416	1.734363
C	5.104556	-2.277464	0.550245
H	6.103299	-1.861210	0.756990
C	5.118977	-3.016165	-0.795561
H	5.866479	-3.826392	-0.773980
H	5.417411	-2.326697	-1.603424
C	3.724113	-3.591477	-1.079581
H	3.728115	-4.115425	-2.048172
C	2.699859	-2.449363	-1.146535
H	1.694715	-2.855261	-1.344589
H	2.946291	-1.792519	-1.990367
C	4.095799	-1.117340	0.484054
H	4.418424	-0.414572	-0.297145
H	4.099341	-0.564618	1.437966
C	3.327607	-4.564638	0.035764
H	2.334007	-4.994180	-0.172946
H	4.040189	-5.404543	0.083038
P	1.367280	-0.324662	0.093953
Pd	-0.573965	-1.011175	-0.819677
Br	-2.682962	-1.659080	-1.876100
H	-0.383200	-2.046323	0.242660

int-s7-ome.log

SCF (RwB97XD) = -1866.35338037
E(SCF)+ZPE(0 K)= -1865.615140
H(298 K)= -1865.579936
G(298 K)= -1865.681916
Lowest Frequency = 12.0878cm-1

C	2.450088	0.200434	2.019187
H	1.910960	-0.553445	2.610602
H	3.520297	-0.057481	2.079774
C	2.183253	1.561836	2.661047
H	2.826999	2.334564	2.211581
H	1.142782	1.863978	2.454491
C	2.417770	1.549645	4.171150
H	1.758708	0.793923	4.632562
H	3.451322	1.220259	4.377766
C	2.172722	2.905384	4.821864
H	1.137156	3.244378	4.658241
H	2.842135	3.676192	4.406654
H	2.343278	2.868184	5.908713
C	2.779766	1.162328	-0.759529
C	2.031878	2.519318	-0.656070
H	1.988187	2.860820	0.386994
H	0.987821	2.385316	-0.980923
C	2.715312	3.589114	-1.520193
H	2.168832	4.538443	-1.402058
C	4.171175	3.769456	-1.068741
H	4.204067	4.106652	-0.018952
H	4.663466	4.550190	-1.672466
C	4.918154	2.437611	-1.216288
H	5.964354	2.560254	-0.892816
C	4.881149	1.985787	-2.682612
H	5.387675	2.728293	-3.321489
H	5.427261	1.034285	-2.800003
C	3.421941	1.813503	-3.126384
H	3.389209	1.476459	-4.174824
C	2.751051	0.745618	-2.247590
H	1.708150	0.591128	-2.572344
H	3.282286	-0.205854	-2.392240
C	2.680507	3.148303	-2.988033
H	3.149243	3.912247	-3.630764
H	1.636112	3.039353	-3.324062

C	4.248014	1.374371	-0.330047
H	4.811644	0.432740	-0.396892
H	4.297288	1.701357	0.719618
C	2.333404	-1.828421	-0.066319
C	1.830867	-2.711564	1.106242
H	2.354690	-2.447648	2.036461
H	0.754874	-2.533105	1.277223
C	2.085657	-4.199988	0.822715
H	1.715363	-4.788188	1.677483
C	3.594995	-4.424322	0.654866
H	4.125877	-4.149720	1.582056
H	3.803266	-5.491428	0.470042
C	4.106108	-3.576942	-0.517452
H	5.190464	-3.728568	-0.640735
C	3.376136	-3.991141	-1.803387
H	3.587056	-5.048585	-2.034741
H	3.744163	-3.396683	-2.656784
C	1.865658	-3.779386	-1.626760
H	1.338714	-4.064342	-2.551035
C	1.586582	-2.296831	-1.340940
H	0.500518	-2.144954	-1.212615
H	1.885410	-1.699117	-2.211594
C	3.843600	-2.085721	-0.241955
H	4.239472	-1.496527	-1.082025
H	4.391849	-1.768881	0.660709
C	1.355446	-4.626064	-0.455725
H	0.268069	-4.488797	-0.336634
H	1.526901	-5.697448	-0.652901
P	1.834187	-0.056199	0.305583
Pd	-0.537449	0.126092	0.102972
C	-3.736866	0.529924	0.053746
C	-3.783321	1.625317	0.926187
C	-4.936038	0.034449	-0.472617
C	-4.991279	2.213400	1.261751
H	-2.844280	2.007101	1.335698
C	-6.157718	0.610877	-0.143765
H	-4.902588	-0.819508	-1.153264
H	-5.037351	3.067522	1.939657
H	-7.074221	0.201527	-0.568243
C	-2.443637	-0.091988	-0.303724
O	-2.353432	-1.038562	-1.079367
H	-0.742804	1.299856	1.082793
C	-6.188387	1.709417	0.729056
O	-7.316515	2.343646	1.111404
C	-8.558517	1.879018	0.622095
H	-9.324310	2.534212	1.055561
H	-8.613870	1.944848	-0.476942
H	-8.756197	0.841307	0.937210

p-anisaldehyde.log

SCF (RwB97XD) = -459.799176955
E(SCF)+ZPE(0 K)= -459.655777
H(298 K)= -459.645897
G(298 K)= -459.690179
Lowest Frequency = 57.7214cm-1

C	-1.353507	-0.180327	-0.001451
C	-0.814904	1.112987	-0.001363
C	0.568740	1.272833	-0.000645
C	1.425302	0.169791	0.000070
C	0.871952	-1.123231	-0.000034
C	-0.497864	-1.298621	-0.000814
H	-1.457112	1.992992	-0.001834
H	0.990103	2.282393	-0.000578
H	1.540847	-1.986656	0.000511
H	-0.942216	-2.295447	-0.000917
O	-2.672022	-0.454133	-0.002135
C	-3.591266	0.621535	-0.002512
H	-3.481042	1.246510	0.898573
H	-3.479806	1.246987	-0.903108
H	-4.591212	0.171000	-0.003333
C	2.884338	0.378509	0.001113
H	3.200086	1.450720	0.000681
O	3.714490	-0.502241	0.002443

p-tolualdehyde.log

SCF (RwB97XD) = -384.584626290
E(SCF)+ZPE(0 K)= -384.446423
H(298 K)= -384.437311
G(298 K)= -384.480181
Lowest Frequency = 39.5256cm-1

C	-1.821462	0.083641	0.000270
C	-1.244389	-1.190814	0.016861
C	0.139925	-1.345339	0.017153
C	0.975548	-0.225237	-0.000387
C	0.406354	1.056680	-0.016795
C	-0.972356	1.204045	-0.016115
H	-1.888519	-2.073565	0.029851
H	0.578094	-2.347611	0.030262
H	1.062834	1.929601	-0.030288
H	-1.410166	2.205722	-0.028819
C	2.442784	-0.410186	-0.002901
H	2.777767	-1.475922	0.018261
O	3.254505	0.485568	-0.026815
C	-3.313953	0.267926	-0.001331
H	-3.843348	-0.694722	0.014067
H	-3.638927	0.849404	0.875651
H	-3.640049	0.821826	-0.895625

trimethylamine.log

SCF (RwB97XD) = -174.356887168
E(SCF)+ZPE(0 K)= -174.236217
H(298 K)= -174.229897
G(298 K)= -174.263455
Lowest Frequency = 254.0801cm-1

N	0.482624	-0.501583	-0.000072
C	0.908830	0.190906	1.197620
H	0.519259	1.220203	1.197798
H	0.516195	-0.319312	2.090209
H	2.016265	0.245352	1.295530
C	0.906644	0.192450	-1.197644
H	0.512367	-0.316602	-2.090177
H	0.517090	1.221751	-1.195760
H	2.013900	0.246998	-1.297516
C	0.913167	-1.883735	-0.001335
H	0.524555	-2.402029	0.888404
H	0.523044	-2.400901	-0.891067
H	2.020936	-1.992444	-0.002340

trimethylammoniumbromide.log

SCF (RwB97XD) = -2749.22609136
E(SCF)+ZPE(0 K)= -2749.090616
H(298 K)= -2749.081957
G(298 K)= -2749.124283
Lowest Frequency = 48.4223cm-1

N	0.509921	-0.522265	-0.009854
C	0.898065	0.196855	1.218532
H	0.474590	1.207673	1.185753
H	0.487639	-0.334637	2.085396
H	1.992771	0.249050	1.291097
C	0.921988	0.194349	-1.231860
H	0.531673	-0.340619	-2.105835
H	0.495399	1.204175	-1.210989
H	2.017882	0.248992	-1.281270
C	0.960740	-1.927404	-0.004191
H	0.559211	-2.424915	0.886556
H	0.571677	-2.427874	-0.898849
H	2.058360	-1.967074	0.003484
Br	-2.572181	-0.595989	-0.025982
H	-0.569082	-0.548094	-0.018980

ts-1-cf3.log

SCF (RwB97XD) = -4549.26397814
E(SCF)+ZPE(0 K)= -4548.571701
H(298 K)= -4548.536255
G(298 K)= -4548.641399
Lowest Frequency = -164.1991cm-1

C	-1.140285	0.351562	-2.125773
H	-0.814093	-0.433503	-2.823325
H	-2.237614	0.280025	-2.088476
C	-0.686715	1.710976	-2.674186
H	-1.406957	2.052520	-3.436928
H	-0.709019	2.470639	-1.875239
C	0.711352	1.674071	-3.289376
H	1.401514	1.161147	-2.594030
H	0.686246	1.054489	-4.202884
C	1.267706	3.055637	-3.603161
H	2.258184	2.994077	-4.078084
H	1.378756	3.650900	-2.682509
H	0.605692	3.614838	-4.284118
C	-1.843763	0.355509	0.726252
C	-3.083850	-0.555904	0.649792
H	-2.808519	-1.588819	0.910186
H	-3.486818	-0.577059	-0.376513
C	-4.168089	-0.072689	1.628433
H	-5.040691	-0.740457	1.548823
C	-3.613276	-0.116460	3.059222
H	-3.328164	-1.148653	3.324097
H	-4.390211	0.196161	3.776785
C	-2.393327	0.809552	3.161890
H	-1.983031	0.771754	4.183466
C	-2.807867	2.246217	2.823005
H	-3.570923	2.603039	3.534784
H	-1.940812	2.921627	2.913514
C	-3.358306	2.284749	1.392389
H	-3.644786	3.315650	1.129741
C	-2.279475	1.808503	0.408716
H	-2.680976	1.877454	-0.613864
H	-1.402129	2.476680	0.457663
C	-4.581281	1.363646	1.282565
H	-5.376933	1.704797	1.965651
H	-4.995730	1.403527	0.261035
C	-1.308461	0.344369	2.178824
H	-0.426938	1.002314	2.250491
H	-0.969866	-0.662396	2.459971
C	-0.142156	-1.898087	-0.572577
C	0.122584	-2.490140	0.827998
H	0.898819	-1.901199	1.344617
H	-0.790422	-2.432761	1.439771
C	0.552417	-3.961695	0.721586
H	0.732021	-4.351927	1.735946
C	1.837139	-4.070529	-0.109588
H	2.651139	-3.507288	0.376144
H	2.162733	-5.122438	-0.169962
C	1.578687	-3.514295	-1.515769
H	2.500230	-3.575328	-2.116232
C	0.462040	-4.318538	-2.194085
H	0.760680	-5.375493	-2.293210
H	0.285465	-3.937001	-3.213928
C	-0.820494	-4.207131	-1.359890
H	-1.629817	-4.776072	-1.845082
C	-1.246722	-2.731667	-1.260552
H	-2.189542	-2.664566	-0.701307
H	-1.448464	-2.347695	-2.272369
C	1.158203	-2.042058	-1.405303
H	1.014254	-1.620930	-2.414434
H	1.963564	-1.457535	-0.929780
C	-0.567563	-4.766183	0.047036
H	-1.490409	-4.708859	0.648762
H	-0.288891	-5.831590	-0.012348
P	-0.453883	-0.056870	-0.458859
Pd	1.493940	1.187208	0.069145
C	3.770206	4.679117	-1.303367
C	4.303075	3.416291	-1.592402
C	3.952011	2.314082	-0.825980
C	2.511136	3.742683	0.535700
C	2.874121	4.837526	-0.245555
H	5.001755	3.293749	-2.423041
H	4.386852	1.335591	-1.035746
H	1.842457	3.875645	1.387870
H	2.459480	5.820557	-0.016432
C	3.027101	2.470569	0.226287
Br	3.249073	1.079996	1.895584
C	4.147807	5.842760	-2.177012
F	3.562817	5.771127	-3.393530
F	5.475158	5.897994	-2.404552
F	3.790995	7.025519	-1.649882

ts-1-cl.log

SCF (RwB97XD) = -4671.81225653
E(SCF)+ZPE(0 K)= -4671.134245
H(298 K)= -4671.101221
G(298 K)= -4671.199850
Lowest Frequency = -150.3022cm-1

C	-1.154053	0.380297	-2.096266
H	-0.822179	-0.397909	-2.798753
H	-2.250511	0.297070	-2.056747
C	-0.718428	1.746692	-2.642183
H	-1.450634	2.084020	-3.395540
H	-0.738884	2.502578	-1.839596
C	0.672363	1.727911	-3.274052
H	1.383942	1.253831	-2.573354
H	0.651815	1.082609	-4.169858
C	1.185885	3.114277	-3.636908
H	2.177602	3.067460	-4.111687
H	1.278975	3.745615	-2.738572
H	0.505152	3.626769	-4.336195
C	-1.855211	0.357670	0.754857
C	-3.089814	-0.560284	0.669112
H	-2.808387	-1.593725	0.920768
H	-3.491954	-0.574938	-0.357628
C	-4.177950	-0.092294	1.650881
H	-5.046712	-0.764251	1.564131
C	-3.624402	-0.145992	3.081818
H	-3.333951	-1.178966	3.337764
H	-4.403857	0.155879	3.801327
C	-2.409611	0.785681	3.194072
H	-1.999991	0.740687	4.215637
C	-2.831921	2.223066	2.868278
H	-3.597706	2.568862	3.582632
H	-1.968706	2.902339	2.965910
C	-3.381346	2.271825	1.437568
H	-3.673674	3.303526	1.184475
C	-2.299139	1.810975	0.450363
H	-2.700625	1.886688	-0.571798
H	-1.425483	2.483393	0.505634
C	-4.598923	1.344834	1.317951
H	-5.397239	1.675012	2.003387
H	-5.012520	1.392003	0.296347
C	-1.321275	0.335870	2.207720
H	-0.443356	0.997570	2.286213
H	-0.977495	-0.671735	2.479587
C	-0.139661	-1.873022	-0.564757
C	0.132239	-2.476228	0.829704
H	0.905479	-1.886480	1.349906
H	-0.779957	-2.430422	1.443714
C	0.571601	-3.943866	0.709094
H	0.756328	-4.342115	1.719414
C	1.855070	-4.036515	-0.126038
H	2.666193	-3.471841	0.362820
H	2.187907	-5.085556	-0.196470
C	1.589519	-3.469578	-1.526585
H	2.509976	-3.519197	-2.129778
C	0.476650	-4.275198	-2.209452
H	0.782163	-5.329191	-2.318926
H	0.295053	-3.885752	-3.225426
C	-0.804591	-4.179754	-1.371270
H	-1.611328	-4.749682	-1.859678
C	-1.240272	-2.707999	-1.257568

H -2.182178 -2.652396 -0.695510
H -1.447320 -2.316435 -2.265402
C 1.159571 -2.001279 -1.401973
H 1.010600 -1.572158 -2.406984
H 1.962090 -1.415196 -0.923472
C -0.544559 -4.749840 0.029945
H -1.466339 -4.704196 0.634334
H -0.259026 -5.812853 -0.039744
P -0.461174 -0.033931 -0.432933
Pd 1.482358 1.204629 0.099258
C 3.836566 4.551993 -1.441477
C 4.341027 3.269751 -1.662712
C 3.957740 2.224902 -0.827892
C 2.565432 3.768672 0.449723
C 2.956954 4.808727 -0.391264
H 5.032490 3.087735 -2.487095
H 4.366414 1.224710 -0.981825
H 1.902509 3.969751 1.293160
H 2.579464 5.819372 -0.226371
C 3.039407 2.466250 0.210508
Br 3.202902 1.151969 1.962659
Cl 4.310714 5.849963 -2.498471

ts-1-cn.log

SCF (RwB97XD) = -4304.40348496
E(SCF)+ZPE(0 K)= -4303.717434
H(298 K)= -4303.683750
G(298 K)= -4303.784035
Lowest Frequency = -161.2499cm-1

C -1.165593 0.360911 -2.115927
H -0.840121 -0.421870 -2.816325
H -2.262369 0.284783 -2.072331
C -0.722317 1.722795 -2.666152
H -1.442157 2.053668 -3.433903
H -0.757335 2.485090 -1.870153
C 0.678956 1.698972 -3.274466
H 1.374320 1.206919 -2.568773
H 0.668598 1.065849 -4.178818
C 1.213515 3.084752 -3.606823
H 2.209496 3.033916 -4.071951
H 1.303558 3.698153 -2.695593
H 0.547416 3.618859 -4.303638
C -1.850174 0.357374 0.742002
C -3.086632 -0.559751 0.673902
H -2.804457 -1.591784 0.930401
H -3.497579 -0.581118 -0.349201
C -4.165172 -0.082601 1.661777
H -5.035201 -0.754352 1.588354
C -3.598791 -0.125299 3.088070
H -3.306904 -1.156432 3.349676
H -4.371394 0.183025 3.812072
C -2.382448 0.806295 3.182108
H -1.963903 0.769476 4.200325
C -2.806281 2.241332 2.847925
H -3.565368 2.593788 3.566021
H -1.941825 2.920850 2.932425
C -3.368179 2.278798 1.421780
H -3.661496 3.308596 1.162455
C -2.294848 1.808647 0.429162
H -2.704514 1.876695 -0.590228
H -1.420335 2.481076 0.472018
C -4.587676 1.352112 1.320629
H -5.379329 1.689004 2.010388
H -5.010577 1.391114 0.302560
C -1.303198 0.346963 2.190233
H -0.424229 1.008871 2.255973
H -0.958001 -0.658653 2.467544
C -0.147653 -1.886769 -0.573418
C 0.129770 -2.480984 0.823838
H 0.907283 -1.890171 1.336467
H -0.779051 -2.428921 1.442262
C 0.565197 -3.950420 0.710364
H 0.753826 -4.342492 1.722333
C 1.844346 -4.051410 -0.130453
H 2.659566 -3.486063 0.350811
H 2.174065 -5.101673 -0.196037
C 1.573371 -3.492657 -1.533228
H 2.490774 -3.548030 -2.140466
C 0.455366 -4.299823 -2.205731
H 0.758043 -5.355066 -2.310191
H 0.269750 -3.916174 -3.223157
C -0.821525 -4.196589 -1.361882
H -1.631808 -4.767713 -1.842807
C -1.253652 -2.723398 -1.255502
H -2.192512 -2.661819 -0.688994
H -1.464737 -2.337651 -2.264712
C 1.147386 -2.022518 -1.415821
H 0.994845 -1.599279 -2.422771
H 1.953775 -1.436036 -0.944208
C -0.556081 -4.758079 0.041745
H -1.474888 -4.706442 0.650089
H -0.273198 -5.822065 -0.022351
P -0.468588 -0.048096 -0.453885
Pd 1.486076 1.203044 0.056876
C 3.822826 4.631362 -1.384692
C 4.337475 3.348343 -1.639368
C 3.959851 2.274540 -0.847378
C 2.536380 3.763680 0.473457
C 2.922185 4.831399 -0.326965
H 5.036961 3.197539 -2.463610
H 4.376697 1.282950 -1.030716
H 1.863260 3.926366 1.316862
H 2.527829 5.830081 -0.130062
C 3.028826 2.472310 0.193672
Br 3.220394 1.127585 1.900232
C 4.210276 5.736895 -2.214053
N 4.515421 6.621725 -2.885108

ts-1-h.log

SCF (RwB97XD) = -4212.19751580
E(SCF)+ZPE(0 K)= -4211.510039
H(298 K)= -4211.478140
G(298 K)= -4211.574331
Lowest Frequency = -187.2736cm-1

C -1.157668 0.360331 -2.117079
H -0.832720 -0.422288 -2.817962
H -2.254287 0.281963 -2.071072
C -0.717731 1.722380 -2.669849
H -1.452279 2.063360 -3.419411
H -0.726871 2.479880 -1.868677
C 0.669366 1.691035 -3.309107
H 1.377257 1.199832 -2.616738
H 0.634892 1.056483 -4.212210
C 1.201686 3.074116 -3.656647
H 2.184667 3.015895 -4.148019
H 1.322500 3.686312 -2.748433
H 0.519129 3.610834 -4.336060
C -1.842548 0.357415 0.736626
C -3.081154 -0.556219 0.663354
H -2.802686 -1.589323 0.919611
H -3.489005 -0.575452 -0.361085
C -4.162344 -0.078560 1.648216
H -5.033886 -0.748068 1.570466
C -3.601225 -0.125312 3.076476
H -3.312682 -1.157697 3.336994
H -4.375767 0.183443 3.798420
C -2.382711 0.802869 3.176269
H -1.967706 0.763013 4.195895
C -2.801848 2.239664 2.843701
H -3.562441 2.592782 3.560101
H -1.935634 2.916362 2.932040
C -3.358816 2.281264 1.415696
H -3.648858 3.312421 1.157642
C -2.283368 1.810335 0.425810
H -2.689899 1.881238 -0.594716
H -1.407045 2.479813 0.472361

C -4.580357 1.357908 1.308783
H -5.373719 1.695096 1.996630
H -4.999615 1.399969 0.289252
C -1.301357 0.343078 2.186944
H -0.420528 1.001947 2.256319
H -0.959493 -0.663932 2.463418
C -0.145599 -1.888575 -0.578338
C 0.127628 -2.485611 0.818510
H 0.906217 -1.897738 1.332557
H -0.781935 -2.430810 1.435716
C 0.557596 -3.956586 0.705061
H 0.743563 -4.350041 1.717076
C 1.837427 -4.062128 -0.134135
H 2.653757 -3.499488 0.348293
H 2.163503 -5.113679 -0.199567
C 1.570232 -3.501671 -1.536976
H 2.488299 -3.560475 -2.143038
C 0.450049 -4.304520 -2.211083
H 0.748422 -5.361134 -2.315417
H 0.267202 -3.919780 -3.228657
C -0.827534 -4.196108 -1.368898
H -1.639563 -4.763948 -1.851059
C -1.253502 -2.720994 -1.262356
H -2.193266 -2.656228 -0.697742
H -1.461424 -2.334348 -2.271898
C 1.149761 -2.030043 -1.419295
H 0.999623 -1.605779 -2.426237
H 1.957342 -1.445909 -0.947327
C -0.565919 -4.759630 0.034670
H -1.485244 -4.704542 0.642022
H -0.287254 -5.824827 -0.029790
P -0.454223 -0.045814 -0.455811
Pd 1.487316 1.187284 0.072904
C 3.808637 4.645391 -1.322054
C 4.325211 3.375746 -1.591923
C 3.959284 2.280042 -0.813455
C 2.532296 3.741370 0.529601
C 2.914355 4.822423 -0.266235
H 5.027055 3.234742 -2.417419
H 4.382816 1.292802 -1.006942
H 1.863664 3.886764 1.380268
H 2.513410 5.815155 -0.047566
C 3.036528 2.462684 0.232535
Br 3.224280 1.071725 1.924053
H 4.109624 5.498870 -1.933029

ts-1-me.log

SCF (RwB97XD) = -4251.47716098
E(SCF)+ZPE(0 K)= -4250.762105
H(298 K)= -4250.728486
G(298 K)= -4250.827920
Lowest Frequency = -171.0123cm-1

C -1.128375 0.355668 -2.116048
H -0.790550 -0.423530 -2.814743
H -2.224990 0.270722 -2.083056
C -0.690540 1.721728 -2.661262
H -1.421025 2.061385 -3.415433
H -0.709815 2.476608 -1.857964
C 0.701828 1.700625 -3.289687
H 1.406117 1.207296 -2.595265
H 0.677450 1.073349 -4.198165
C 1.230997 3.089028 -3.621153
H 2.218845 3.038730 -4.103853
H 1.340539 3.692754 -2.705807
H 0.552252 3.628650 -4.302050
C -1.841609 0.354160 0.731103
C -3.080398 -0.558254 0.646966
H -2.805395 -1.591520 0.906245
H -3.478918 -0.577514 -0.381161
C -4.170129 -0.079131 1.621658
H -5.041570 -0.747872 1.536214
C -3.622155 -0.125733 3.055004
H -3.336963 -1.158263 3.318581
H -4.403078 0.184047 3.769631
C -2.403712 0.801370 3.165598
H -1.998205 0.761544 4.189063
C -2.818493 2.238391 2.828541
H -3.585309 2.592596 3.537765
H -1.952447 2.914262 2.924433
C -3.362352 2.279746 1.395468
H -3.648985 3.311071 1.134248
C -2.278517 1.807191 0.415591
H -2.676186 1.877471 -0.608479
H -1.402123 2.475964 0.469515
C -4.583740 1.357560 1.277785
H -5.383140 1.695761 1.958115
H -4.993388 1.399551 0.254333
C -1.313758 0.340205 2.186317
H -0.432961 0.998334 2.263101
H -0.975258 -0.666924 2.466406
C -0.137209 -1.895111 -0.567010
C 0.120294 -2.491880 0.832901
H 0.895539 -1.905608 1.353874
H -0.795045 -2.434015 1.441194
C 0.547111 -3.964132 0.724915
H 0.721389 -4.357426 1.739085
C 1.835318 -4.073847 -0.100673
H 2.647972 -3.513176 0.390145
H 2.159029 -5.126386 -0.162090
C 1.584349 -3.513453 -1.506592
H 2.508547 -3.575146 -2.103026
C 0.469131 -4.313940 -2.191763
H 0.765768 -5.371465 -2.291910
H 0.297892 -3.929709 -3.211557
C -0.816980 -4.201327 -1.363225
H -1.625402 -4.767458 -1.853412
C -1.240014 -2.724961 -1.262253
H -2.185634 -2.657391 -0.707860
H -1.435825 -2.338325 -2.274252
C 1.166492 -2.040643 -1.394235
H 1.027738 -1.616754 -2.402995
H 1.970456 -1.458125 -0.914258
C -0.571627 -4.764488 0.043433
H -1.497024 -4.706302 0.641237
H -0.295296 -5.830526 -0.017328
P -0.441850 -0.051287 -0.447581
Pd 1.495142 1.180171 0.089987
C 3.783927 4.675509 -1.341646
C 4.301641 3.396327 -1.598683
C 3.953944 2.298068 -0.819897
C 2.528545 3.740906 0.529474
C 2.896926 4.824179 -0.270728
H 4.999852 3.256735 -2.429055
H 4.387817 1.316647 -1.020138
H 1.866433 3.887218 1.385090
H 2.486481 5.812049 -0.043513
C 3.035844 2.466625 0.232010
Br 3.249933 1.070161 1.924017
C 4.169644 5.843126 -2.210085
H 3.805809 6.793832 -1.795202
H 5.262385 5.918680 -2.320605
H 3.747349 5.738238 -3.223004

ts-1-nme2.log

SCF (RwB97XD) = -4346.09189768
E(SCF)+ZPE(0 K)= -4345.330685
H(298 K)= -4345.294701
G(298 K)= -4345.398783
Lowest Frequency = -162.7565cm-1

C -1.082803 0.420229 -2.061477
H -0.750360 -0.343327 -2.779738
H -2.180577 0.348088 -2.037691
C -0.624709 1.795327 -2.565366
H -1.348059 2.168105 -3.310869
H -0.634695 2.525552 -1.739364
C 0.769339 1.772564 -3.190163

H	1.461338	1.241149	-2.512108
H	0.736528	1.181411	-4.122434
C	1.324823	3.162842	-3.464713
H	2.311521	3.114660	-3.949791
H	1.446985	3.724910	-2.524806
H	0.656385	3.744344	-4.121108
C	-1.822800	0.337184	0.774764
C	-3.071926	-0.554555	0.639851
H	-2.813792	-1.599503	0.867784
H	-3.454978	-0.533625	-0.394058
C	-4.170814	-0.095148	1.613807
H	-5.050535	-0.746944	1.489635
C	-3.646709	-0.201851	3.052773
H	-3.380897	-1.247408	3.283076
H	-4.434350	0.093948	3.766039
C	-2.416825	0.702343	3.215598
H	-2.027958	0.618473	4.242919
C	-2.806376	2.156562	2.926427
H	-3.579678	2.494911	3.636453
H	-1.932467	2.815361	3.061579
C	-3.326855	2.258863	1.487742
H	-3.595368	3.303174	1.260948
C	-2.235360	1.806994	0.506864
H	-2.617629	1.919772	-0.519329
H	-1.350179	2.459958	0.596884
C	-4.558708	1.359265	1.316897
H	-5.364705	1.683225	1.996509
H	-4.950415	1.445600	0.289117
C	-1.317994	0.262445	2.236221
H	-0.429471	0.904449	2.350126
H	-0.997834	-0.758935	2.484388
C	-0.118781	-1.882485	-0.575073
C	0.125610	-2.524741	0.806975
H	0.899053	-1.958552	1.352258
H	-0.794571	-2.482104	1.409263
C	0.546528	-3.994903	0.655817
H	0.712290	-4.420902	1.658131
C	1.839751	-4.084911	-0.164309
H	2.650980	-3.542556	0.348890
H	2.159972	-5.136400	-0.255582
C	1.600595	-3.480231	-1.553872
H	2.528294	-3.528279	-2.146182
C	0.485708	-4.253039	-2.270851
H	0.777561	-5.308450	-2.402853
H	0.323000	-3.836029	-3.279097
C	-0.805189	-4.159637	-1.447283
H	-1.613253	-4.706244	-1.959835
C	-1.221213	-2.684745	-1.302679
H	-2.170004	-2.629663	-0.752162
H	-1.409141	-2.265985	-2.303320
C	1.189799	-2.009554	-1.397851
H	1.060259	-1.553041	-2.393475
H	1.993510	-1.446808	-0.894494
C	-0.571252	-4.768293	-0.057580
H	-1.500046	-4.725058	0.536271
H	-0.299259	-5.833223	-0.150448
P	-0.410655	-0.040047	-0.399696
Pd	1.520239	1.163147	0.189566
C	3.919306	4.646503	-1.190905
C	4.417268	3.344596	-1.432379
C	4.014969	2.261168	-0.661439
C	2.610139	3.727881	0.658688
C	3.016800	4.809973	-0.116058
H	5.132037	3.165288	-2.234705
H	4.434840	1.272894	-0.858876
H	1.936403	3.891948	1.502081
H	2.623777	5.795071	0.132691
C	3.074562	2.436902	0.365641
Br	3.221821	1.028868	2.073017
N	4.301864	5.713499	-1.972628
C	3.871612	7.046794	-1.625282
H	4.250926	7.757973	-2.368744
H	2.771947	7.129577	-1.623135
H	4.238903	7.365951	-0.632195
C	5.321952	5.536880	-2.978479
H	5.021222	4.792953	-3.734957
H	5.484829	6.486296	-3.501846
H	6.289442	5.215565	-2.550380

ts-1-ome.log

SCF (RwB97XD) = -4326.68936031
E(SCF)+ZPE(0 K)= -4325.968871
H(298 K)= -4325.934576
G(298 K)= -4326.035651
Lowest Frequency = -146.5208cm-1

C	-0.950430	0.340631	-2.123919
H	-0.569079	-0.440956	-2.796963
H	-2.046692	0.251312	-2.156228
C	-0.487688	1.704931	-2.652403
H	-1.172279	2.031648	-3.454004
H	-0.561418	2.467354	-1.859401
C	0.940392	1.687386	-3.194018
H	1.607754	1.232715	-2.439551
H	0.982013	1.027227	-4.078318
C	1.465220	3.071642	-3.548706
H	2.483104	3.019809	-3.964481
H	1.503103	3.718403	-2.657071
H	0.823915	3.567437	-4.295947
C	-1.832538	0.350400	0.675771
C	-3.066460	-0.559089	0.519642
H	-2.809346	-1.592261	0.796876
H	-3.404089	-0.579151	-0.530035
C	-4.211448	-0.075117	1.426561
H	-5.078215	-0.741453	1.289610
C	-3.750209	-0.120518	2.890154
H	-3.484127	-1.153383	3.171537
H	-4.571287	0.192555	3.556640
C	-2.538106	0.803764	3.072122
H	-2.194812	0.764504	4.118253
C	-2.928378	2.241076	2.707962
H	-3.735954	2.598616	3.368682
H	-2.068001	2.914984	2.855277
C	-3.384864	2.281071	1.244572
H	-3.652489	3.312750	0.965229
C	-2.245747	1.803920	0.331994
H	-2.580770	1.872283	-0.714386
H	-1.372487	2.470740	0.437113
C	-4.599333	1.361986	1.055266
H	-5.437627	1.703912	1.684993
H	-4.945429	1.403176	0.008554
C	-1.392884	0.338181	2.160054
H	-0.516577	0.994649	2.287441
H	-1.073608	-0.668874	2.461873
C	-0.055727	-1.904505	-0.512074
C	0.111955	-2.497463	0.902755
H	0.854426	-1.911083	1.469123
H	-0.839584	-2.435770	1.452112
C	0.541781	-3.970665	0.826442
H	0.650647	-4.360805	1.850929
C	1.879349	-4.085129	0.085008
H	2.660018	-3.524052	0.624900
H	2.204131	-5.138500	0.047841
C	1.718789	-3.528658	-1.335904
H	2.679105	-3.594444	-1.872148
C	0.647400	-4.329103	-2.087937
H	0.947914	-5.387351	-2.166638
H	0.541036	-3.946998	-3.117387
C	-0.688112	-4.212342	-1.342560
H	-1.464782	-4.778826	-1.881297
C	-1.114449	-2.735074	-1.272455
H	-2.092655	-2.664992	-0.778065
H	-1.246247	-2.351671	-2.295968
C	1.297474	-2.054721	-1.254671
H	1.223339	-1.634625	-2.271887
H	2.070239	-1.471816	-0.726640
C	-0.532693	-4.771929	0.078224
H	-1.493807	-4.711405	0.616638
H	-0.253870	-5.838305	0.037624

P -0.364375 -0.059537 -0.415994
Pd 1.515916 1.192258 0.254810
C 3.639330 4.900895 -0.803934
C 4.233984 3.689483 -1.175359
C 3.914950 2.515820 -0.490370
C 2.411160 3.768996 0.953440
C 2.732850 4.931589 0.265686
H 4.949959 3.639347 -1.995713
H 4.399573 1.577771 -0.767433
H 1.727022 3.807188 1.803331
H 2.288815 5.887283 0.551381
C 2.977079 2.544798 0.550061
Br 3.221315 1.010033 2.127139
O 3.883501 6.091114 -1.407564
C 4.745564 6.111098 -2.523374
H 4.365623 5.478641 -3.343395
H 4.782544 7.152234 -2.868345
H 5.766892 5.790168 -2.257565

ts-2-cf3.log

SCF (RwB97XD) = -4662.59686861
E(SCF)+ZPE(0 K)= -4661.893666
H(298 K)= -4661.856234
G(298 K)= -4661.963701
Lowest Frequency = -264.1161cm-1

C 2.282356 -0.139181 1.973449
H 2.037467 -1.208520 2.029637
H 3.380919 -0.054516 1.971473
C 1.680341 0.534147 3.206913
H 2.036300 1.573059 3.298076
H 0.584069 0.580165 3.100886
C 2.022209 -0.218699 4.491312
H 1.642036 -1.250800 4.409835
H 3.119508 -0.298949 4.585732
C 1.444884 0.441224 5.736705
H 1.705341 -0.120311 6.646735
H 0.345725 0.494296 5.682800
H 1.822563 1.469338 5.860734
C 2.050755 2.189988 0.160975
C 0.939324 2.994036 0.887239
H 0.873973 2.698277 1.943371
H -0.038717 2.765412 0.436592
C 1.204552 4.503575 0.789376
H 0.403123 5.035469 1.326039
C 2.561620 4.832142 1.425218
H 2.559381 4.552077 2.492037
H 2.750374 5.917410 1.378572
C 3.666820 4.070383 0.682903
H 4.644222 4.295393 1.138413
C 3.675573 4.483127 -0.794467
H 3.886837 5.561328 -0.885921
H 4.478717 3.951383 -1.332174
C 2.313827 4.154748 -1.421299
H 2.316868 4.435133 -2.486485
C 2.060414 2.641845 -1.317124
H 1.102634 2.392543 -1.798475
H 2.849672 2.118299 -1.874377
C 1.208542 4.921727 -0.685885
H 1.376102 6.007764 -0.775027
H 0.228445 4.708243 -1.143935
C 3.412007 2.558490 0.797627
H 4.229126 2.010735 0.307430
H 3.429029 2.275187 1.859649
C 2.586508 -0.692872 -0.878678
C 4.081696 -0.329116 -1.011819
H 4.201293 0.704981 -1.364984
H 4.577399 -0.395810 -0.029623
C 4.774921 -1.274224 -2.008322
H 5.835673 -0.986592 -2.084569
C 4.103614 -1.143937 -3.382342
H 4.189022 -0.107293 -3.749885
H 4.615729 -1.787521 -4.116546
C 2.625918 -1.543834 -3.269432
H 2.138269 -1.442850 -4.252139
C 2.515160 -2.991880 -2.779154
H 2.998252 -3.677040 -3.495474
H 1.455353 -3.288902 -2.712052
C 3.182575 -3.106089 -1.403981
H 3.091504 -4.137875 -1.030056
C 2.493902 -2.166470 -0.402201
H 2.986135 -2.284674 0.573887
H 1.439700 -2.456245 -0.264956
C 1.914714 -0.617447 -2.272837
H 0.857576 -0.917287 -2.184090
H 1.919415 0.410526 -2.658857
C 4.663963 -2.722867 -1.516801
H 5.159127 -2.830332 -0.537182
H 5.182033 -3.401252 -2.214757
P 1.638961 0.370341 0.339270
Pd -0.644880 -0.082901 0.069610
Br -0.829589 -1.890533 1.795484
C -2.698976 -0.134316 -0.637924
C -3.666335 0.523293 0.128384
C -3.067910 -1.193861 -1.471158
C -4.991340 0.101838 0.088003
H -3.386826 1.364027 0.767332
C -4.392616 -1.619083 -1.513982
H -2.319040 -1.699534 -2.085014
C -5.349639 -0.971695 -0.730964
H -5.744817 0.606218 0.695888
H -4.677448 -2.456238 -2.153012
C -6.798240 -1.386445 -0.808778
F -7.416645 -1.296477 0.380859
F -7.490961 -0.601769 -1.661700
F -6.948807 -2.649154 -1.238491
C -1.271103 0.990820 -1.309354
O -1.451903 1.718891 -2.185301

ts-2-cl.log

SCF (RwB97XD) = -4785.14716874
E(SCF)+ZPE(0 K)= -4784.458770
H(298 K)= -4784.423582
G(298 K)= -4784.525901
Lowest Frequency = -262.3237cm-1

C 1.509061 -0.211535 -2.296688
H 0.837478 0.521399 -2.763902
H 2.541002 0.078498 -2.551910
C 1.163312 -1.583092 -2.875778
H 1.897667 -2.339546 -2.556120
H 0.182491 -1.904752 -2.488872
C 1.112087 -1.562784 -4.402420
H 0.362757 -0.820143 -4.724202
H 2.083317 -1.213456 -4.795492
C 0.773382 -2.922010 -5.000804
H 0.744630 -2.882389 -6.100358
H -0.212239 -3.272454 -4.654879
H 1.516694 -3.684100 -4.714685
C 2.392076 -1.171925 0.358221
C 3.734351 -1.359600 -0.388733
H 4.280949 -0.407615 -0.448256
H 3.556467 -1.690936 -1.421713
C 4.604871 -2.406691 0.325837
H 5.552642 -2.510324 -0.226017
C 4.889475 -1.948951 1.762156
H 5.432741 -0.989012 1.753628
H 5.537719 -2.680091 2.272862
C 3.563386 -1.798181 2.518806
H 3.758176 -1.456598 3.547658
C 2.831352 -3.144698 2.550329
H 3.443817 -3.897347 3.073889
H 1.886958 -3.050081 3.111376
C 2.548095 -3.595132 1.112174
H 2.006437 -4.554133 1.121732
C 1.669614 -2.544759 0.416922
H 1.408797 -2.890269 -0.592997
H 0.721744 -2.441410 0.967414

C	3.869195	-3.752654	0.348736
H	4.496349	-4.521154	0.830109
H	3.674194	-4.095516	-0.681266
C	2.693521	-0.745657	1.812696
H	1.755178	-0.604422	2.370493
H	3.227264	0.214478	1.828232
C	1.741433	1.816781	-0.209814
C	1.262008	2.271466	1.191627
H	0.173932	2.115183	1.273308
H	1.727924	1.669664	1.983253
C	1.582519	3.755235	1.421669
H	1.251141	4.033350	2.434853
C	0.838770	4.599308	0.380608
H	-0.249496	4.452139	0.480541
H	1.036348	5.671628	0.544677
C	1.300491	4.184296	-1.020719
H	0.758272	4.768974	-1.780271
C	2.809558	4.425281	-1.158621
H	3.040489	5.493904	-1.015213
H	3.144604	4.156954	-2.174426
C	3.553334	3.580431	-0.116673
H	4.638982	3.740032	-0.213672
C	3.255871	2.087856	-0.342039
H	3.822693	1.500749	0.394469
H	3.610872	1.784159	-1.340298
C	1.005567	2.695091	-1.256034
H	1.335642	2.437106	-2.272650
H	-0.079091	2.505604	-1.215832
C	3.095140	3.982374	1.292071
H	3.633687	3.389793	2.051005
H	3.337441	5.041283	1.481290
P	1.244102	0.033284	-0.503676
Pd	-1.000507	-0.211370	0.150391
Br	-2.027821	0.237116	-2.091333
C	-2.839955	-0.447942	1.253637
C	-3.511181	-1.671598	1.168911
C	-3.545814	0.707343	1.603802
C	-4.883327	-1.739102	1.394842
H	-2.965627	-2.584210	0.916973
C	-4.917805	0.652450	1.832224
H	-3.027942	1.665267	1.693431
C	-5.574063	-0.573010	1.722295
H	-5.414683	-2.688850	1.317261
H	-5.475972	1.553105	2.092385
Cl	-7.285979	-0.650341	2.010516
C	-1.020551	-0.546452	1.977549
O	-0.832673	-0.731699	3.098928

ts-2-cn.log

SCF (RwB97XD) = -4417.73425854
E(SCF)+ZPE(0 K)= -4417.037324
H(298 K)= -4417.001563
G(298 K)= -4417.104948
Lowest Frequency = -271.7872cm-1

C	0.988518	-0.007347	2.570690
H	0.276816	-0.792338	2.862048
H	1.971440	-0.323836	2.953735
C	0.547223	1.296393	3.235848
H	1.312093	2.076759	3.100288
H	-0.365687	1.676578	2.744770
C	0.280498	1.123421	4.730559
H	-0.500515	0.357106	4.873138
H	1.189042	0.727477	5.216660
C	-0.143815	2.418407	5.411351
H	-1.065219	2.822062	4.961442
H	0.635255	3.192816	5.323158
H	-0.338714	2.263757	6.483214
C	2.313452	1.175735	0.210430
C	1.635225	2.571537	0.173020
H	1.215546	2.825616	1.157022
H	0.799517	2.550637	-0.541738
C	2.643187	3.650915	-0.247815
H	2.130359	4.625640	-0.249974
C	3.815739	3.682083	0.741088
H	3.454305	3.928909	1.753871
H	4.532500	4.469992	0.456320
C	4.510083	2.313880	0.748297
H	5.349349	2.325520	1.462037
C	5.030238	1.992308	-0.658577
H	5.770846	2.747339	-0.970555
H	5.546213	1.017230	-0.658134
C	3.851810	1.966136	-1.640259
H	4.213942	1.721275	-2.651208
C	2.853519	0.880324	-1.208022
H	2.017070	0.830198	-1.920987
H	3.368107	-0.091059	-1.226780
C	3.161128	3.334937	-1.656090
H	3.868892	4.113748	-1.985913
H	2.325191	3.329269	-2.373932
C	3.507904	1.234383	1.192300
H	4.016066	0.260320	1.243781
H	3.163640	1.471344	2.209679
C	1.472230	-1.824980	0.342865
C	0.525952	-2.766657	1.133274
H	0.660176	-2.632607	2.216326
H	-0.525629	-2.525883	0.901355
C	0.813939	-4.237690	0.794559
H	0.124565	-4.870014	1.376347
C	2.263948	-4.566454	1.174733
H	2.415108	-4.421630	2.257676
H	2.482047	-5.625213	0.958199
C	3.213358	-3.659382	0.382069
H	4.257181	-3.884496	0.653341
C	3.007397	-3.889227	-1.121881
H	3.246203	-4.934164	-1.380603
H	3.694028	-3.249472	-1.701252
C	1.552737	-3.571237	-1.493411
H	1.402731	-3.723990	-2.573467
C	1.243867	-2.104303	-1.162940
H	0.200673	-1.873572	-1.429127
H	1.867554	-1.450048	-1.784483
C	2.926452	-2.185627	0.716737
H	3.633537	-1.551914	0.163385
H	3.102440	-2.007050	1.790265
C	0.605615	-4.481531	-0.704036
H	-0.440511	-4.270974	-0.982249
H	0.798000	-5.539844	-0.946558
P	1.005192	-0.055179	0.735052
Pd	-1.166767	0.345433	-0.052008
Br	-0.760963	1.008343	-2.439073
C	-3.335405	0.492840	-0.110901
C	-3.894626	1.768752	0.008965
C	-4.045807	-0.530022	-0.746958
C	-5.143559	2.037252	-0.539759
H	-3.350661	2.563921	0.523798
C	-5.295608	-0.272741	-1.298186
H	-3.619200	-1.532437	-0.824787
C	-5.842481	1.014503	-1.196555
H	-5.579617	3.034704	-0.464616
H	-5.849034	-1.061683	-1.810081
C	-7.137614	1.285667	-1.763732
N	-8.173894	1.501540	-2.214525
C	-2.232222	-0.074160	1.402633
O	-2.702409	-0.406995	2.399872

ts-2-h.log

SCF (RwB97XD) = -4325.53284718
E(SCF)+ZPE(0 K)= -4324.835271
H(298 K)= -4324.801131
G(298 K)= -4324.901194
Lowest Frequency = -266.7471cm-1

C	-0.990222	-0.254830	2.256712
H	-0.169746	0.301803	2.729697
H	-1.934561	0.177160	2.625423
C	-0.853168	-1.716795	2.681916
H	-1.742538	-2.295107	2.384204

H	0.010133	-2.169374	2.167411
C	-0.657314	-1.852589	4.190878
H	0.260340	-1.312320	4.478171
H	-1.490019	-1.349681	4.713695
C	-0.562394	-3.301815	4.649541
H	-0.408191	-3.370592	5.737114
H	0.280522	-3.816827	4.161518
H	-1.480022	-3.862909	4.407548
C	-2.186112	-0.788932	-0.396322
C	-3.495699	-0.839412	0.425966
H	-3.879645	0.174917	0.604821
H	-3.313377	-1.290036	1.412012
C	-4.561451	-1.667090	-0.312021
H	-5.480011	-1.677585	0.295863
C	-4.852008	-1.033468	-1.678872
H	-5.235250	-0.007387	-1.547749
H	-5.635884	-1.604275	-2.203668
C	-3.564471	-1.013608	-2.513426
H	-3.761395	-0.546144	-3.491074
C	-3.059312	-2.446783	-2.716318
H	-3.812748	-3.041869	-3.258669
H	-2.145249	-2.443818	-3.333011
C	-2.769737	-3.074095	-1.347544
H	-2.389900	-4.099521	-1.479838
C	-1.697398	-2.244490	-0.626835
H	-1.440685	-2.723108	0.328242
H	-0.776298	-2.234318	-1.230182
C	-4.053013	-3.101145	-0.507195
H	-4.822405	-3.712054	-1.007837
H	-3.856286	-3.571221	0.471005
C	-2.499146	-0.181015	-1.781966
H	-1.583684	-0.131240	-2.390873
H	-2.873097	0.846799	-1.675239
C	-1.032937	1.989904	0.395439
C	-0.585884	2.515277	-0.991694
H	0.456642	2.209819	-1.178881
H	-1.192613	2.076822	-1.794868
C	-0.685676	4.046631	-1.051683
H	-0.387527	4.378148	-2.059126
C	0.254836	4.652724	-0.003854
H	1.295830	4.355366	-0.212882
H	0.218347	5.754050	-0.045969
C	-0.164954	4.163045	1.386487
H	0.515953	4.574223	2.148129
C	-1.602901	4.612186	1.678044
H	-1.671209	5.712542	1.657274
H	-1.903051	4.290119	2.689252
C	-2.543991	4.006624	0.629055
H	-3.580796	4.316073	0.836443
C	-2.472118	2.470732	0.681247
H	-3.172927	2.061068	-0.060160
H	-2.802969	2.115213	1.670655
C	-0.095145	2.629712	1.452909
H	-0.390220	2.317378	2.464876
H	0.939912	2.283564	1.301424
C	-2.129841	4.484208	-0.769290
H	-2.809935	4.065191	-1.530207
H	-2.212708	5.581692	-0.834800
P	-0.806322	0.130708	0.477907
Pd	1.321292	-0.395524	-0.382154
Br	2.544174	-0.393501	1.809124
C	3.019387	-0.835440	-1.632710
C	3.416277	-2.171273	-1.749357
C	3.929485	0.191862	-1.901413
C	4.731479	-2.477254	-2.095245
H	2.700558	-2.975926	-1.562211
C	5.243697	-0.118000	-2.246474
H	3.616041	1.236547	-1.831323
C	5.644756	-1.451334	-2.339945
H	5.044594	-3.520941	-2.172826
H	5.958531	0.684722	-2.441438
H	6.675074	-1.692630	-2.611272
C	1.166712	-0.486955	-2.230094
O	0.886054	-0.480530	-3.346760

ts-2-me.log

SCF (RwB97XD) = -4364.81282689
E(SCF)+ZPE(0 K)= -4364.087388
H(298 K)= -4364.051637
G(298 K)= -4364.154981
Lowest Frequency = -268.8301cm-1

C	-1.175237	0.267309	2.324254
H	-0.922975	1.336404	2.336411
H	-2.240509	0.178568	2.591710
C	-0.286755	-0.422271	3.359212
H	-0.604661	-1.465826	3.513983
H	0.749951	-0.454708	2.985841
C	-0.305045	0.302662	4.703683
H	0.036355	1.340460	4.552086
H	-1.344965	0.368005	5.069583
C	0.568626	-0.374117	5.751853
H	0.240054	-1.408859	5.943308
H	0.540506	0.166721	6.710030
H	1.619611	-0.414259	5.423601
C	-2.172368	0.862060	-0.354184
C	-3.653728	0.483263	-0.138449
H	-3.902298	0.516077	0.935018
H	-3.847637	-0.542474	-0.482531
C	-4.570614	1.444147	-0.915816
H	-5.617394	1.145660	-0.745576
C	-4.355452	2.882284	-0.427850
H	-4.604969	2.961234	0.643449
H	-5.028302	3.570266	-0.966261
C	-2.891941	3.280400	-0.659678
H	-2.720908	4.303668	-0.290621
C	-2.569502	3.207881	-2.156294
H	-3.213637	3.904161	-2.718691
H	-1.526116	3.516134	-2.335023
C	-2.784667	1.770679	-2.644327
H	-2.543632	1.699985	-3.716935
C	-1.851853	0.828705	-1.869834
H	-1.944759	-0.189590	-2.270736
H	-0.805334	1.137091	-2.028252
C	-4.244432	1.355917	-2.413451
H	-4.919934	2.011548	-2.987609
H	-4.408824	0.327841	-2.778130
C	-1.980064	2.325051	0.124844
H	-0.924990	2.624179	0.018585
H	-2.229760	2.413084	1.191897
C	-1.386934	-2.031549	0.471702
C	-2.550925	-2.424150	1.412470
H	-2.308936	-2.160612	2.452239
H	-3.465616	-1.875005	1.147879
C	-2.819790	-3.936147	1.332310
H	-3.656857	-4.179868	2.005750
C	-1.564420	-4.702448	1.768516
H	-1.303513	-4.442851	2.808326
H	-1.754253	-5.788351	1.745907
C	-0.404007	-4.349788	0.829261
H	0.506335	-4.884041	1.143966
C	-0.764908	-4.742010	-0.608409
H	-0.947272	-5.827494	-0.674077
H	0.074264	-4.514327	-1.286340
C	-2.017192	-3.969341	-1.038963
H	-2.277471	-4.230638	-2.076942
C	-1.750303	-2.457060	-0.969113
H	-2.650602	-1.928413	-1.311587
H	-0.936208	-2.190464	-1.660234
C	-0.129405	-2.840050	0.890158
H	0.709523	-2.594561	0.220961
H	0.188926	-2.564603	1.904923
C	-3.185225	-4.320167	-0.107573
H	-4.096670	-3.784607	-0.422770
H	-3.409512	-5.397995	-0.168818
P	-0.946640	-0.211852	0.573652
Pd	1.211163	0.258981	-0.255532
Br	1.779607	2.046217	1.413931
C	3.031697	0.379312	-1.384881

C 3.178463 1.406089 -2.320301
C 4.170310 -0.252459 -0.872797
C 4.450941 1.823386 -2.706551
H 2.297569 1.892581 -2.747268
C 5.436539 0.173132 -1.260671
H 4.070575 -1.071967 -0.156442
C 5.598466 1.220937 -2.178631
H 4.553258 2.636596 -3.430076
H 6.320417 -0.315056 -0.840449
C 6.976368 1.679056 -2.573291
H 6.939749 2.446091 -3.359520
H 7.506960 2.108721 -1.708747
H 7.585782 0.840106 -2.943646
C 1.478362 -0.817080 -1.745217
O 1.465385 -1.536781 -2.642971

ts-2-nme2.log

SCF (RwB97XD) = -4459.42986640
E(SCF)+ZPE(0 K)= -4458.658689
H(298 K)= -4458.620286
G(298 K)= -4458.730346
Lowest Frequency = -255.3041cm-1

C 2.096010 -0.726594 1.771887
H 1.772533 -1.775050 1.719258
H 3.197845 -0.721243 1.756371
C 1.560016 -0.151910 3.082819
H 1.994848 0.840820 3.281498
H 0.469555 -0.013271 3.001734
C 1.854141 -1.066620 4.270281
H 1.389502 -2.049156 4.083010
H 2.942227 -1.244613 4.334346
C 1.344811 -0.505158 5.591328
H 0.253704 -0.354544 5.562306
H 1.808530 0.468076 5.821708
H 1.563966 -1.184044 6.429534
C 2.001412 1.795725 0.233683
C 3.396727 2.004838 0.868174
H 4.166028 1.463666 0.299434
H 3.416819 1.604462 1.892147
C 3.749496 3.501222 0.911624
H 4.749481 3.613338 1.360098
C 3.753903 4.073223 -0.512040
H 4.508776 3.553929 -1.126488
H 4.033716 5.139653 -0.492762
C 2.360352 3.903801 -1.131798
H 2.358348 4.299875 -2.159647
C 1.324877 4.654078 -0.285914
H 1.562310 5.730644 -0.261170
H 0.323231 4.553897 -0.735993
C 1.324561 4.076258 1.134218
H 0.571834 4.595766 1.748177
C 0.961846 2.585368 1.074092
H 0.898883 2.180657 2.093371
H -0.038906 2.471382 0.629385
C 2.713586 4.246526 1.762914
H 2.972796 5.316435 1.827025
H 2.715122 3.852912 2.793242
C 2.008203 2.408635 -1.185004
H 1.024531 2.275844 -1.660475
H 2.745228 1.899751 -1.821483
C 2.330005 -0.979847 -1.125077
C 1.630990 -0.725997 -2.484414
H 0.560348 -0.971427 -2.393435
H 1.688546 0.334365 -2.766446
C 2.260386 -1.585694 -3.589991
H 1.754195 -1.359758 -4.542021
C 2.076491 -3.067625 -3.242676
H 1.003128 -3.309859 -3.171729
H 2.499826 -3.703525 -4.037972
C 2.770878 -3.357781 -1.907586
H 2.629752 -4.415714 -1.635680
C 4.269099 -3.048653 -2.027864
H 4.728481 -3.678992 -2.807442
H 4.782627 -3.283109 -1.080454
C 4.452062 -1.565879 -2.373549
H 5.525616 -1.330983 -2.453391
C 3.839816 -0.690349 -1.266413
H 4.013002 0.365351 -1.518482
H 4.354700 -0.883470 -0.310945
C 2.164007 -2.487206 -0.796544
H 2.675357 -2.730284 0.145891
H 1.098711 -2.730533 -0.653552
C 3.755701 -1.259932 -3.707124
H 3.891919 -0.197689 -3.971790
H 4.211891 -1.853282 -4.516862
P 1.460829 -0.000078 0.217462
Pd -0.883215 -0.249854 -0.068745
Br -1.126476 -2.217456 1.466058
C -2.927024 -0.163840 -0.605634
C -3.825551 0.524697 0.215664
C -3.444370 -1.052381 -1.553750
C -5.194175 0.307104 0.127129
H -3.455227 1.242579 0.952717
C -4.810142 -1.280304 -1.656864
H -2.770421 -1.591064 -2.225481
C -5.725421 -0.606047 -0.813981
H -5.853339 0.855511 0.799010
H -5.164050 -1.992295 -2.401573
N -7.076628 -0.820780 -0.912769
C -7.587695 -1.796893 -1.846685
H -7.329865 -1.542162 -2.888966
H -7.206152 -2.812282 -1.639011
H -8.681181 -1.830247 -1.776761
C -7.983313 -0.149967 -0.010501
H -9.013558 -0.439108 -0.248111
H -7.794114 -0.414435 1.045100
H -7.917682 0.947572 -0.103638
C -1.411350 1.021892 -1.321494
O -1.590734 1.829032 -2.118406

ts-2-ome.log

SCF (RwB97XD) = -4440.02668148
E(SCF)+ZPE(0 K)= -4439.296055
H(298 K)= -4439.259521
G(298 K)= -4439.364221
Lowest Frequency = -251.6018cm-1

C -1.958346 0.346711 -1.867161
H -3.054574 0.238550 -1.841159
H -1.735792 1.422425 -1.875478
C -1.376802 -0.248277 -3.149630
H -0.277637 -0.279362 -3.072729
H -1.717332 -1.287055 -3.288788
C -1.763662 0.566076 -4.382742
H -2.864184 0.632801 -4.445395
H -1.397432 1.598450 -4.254977
C -1.208375 -0.015891 -5.675972
H -0.107493 -0.054326 -5.651391
H -1.499400 0.589512 -6.547882
H -1.573724 -1.041813 -5.846642
C -2.202280 0.741980 1.014151
C -2.159700 2.239354 0.609937
H -2.685680 2.392187 -0.343525
H -1.117615 2.562163 0.454897
C -2.840417 3.111030 1.676282
H -2.784182 4.162198 1.352430
C -4.308567 2.685877 1.809920
H -4.833366 2.830103 0.850633
H -4.822348 3.314627 2.555949
C -4.371414 1.211979 2.229718
H -5.422711 0.894965 2.319844
C -3.659230 1.030384 3.577075
H -4.166642 1.622723 4.356471
H -3.708864 -0.025560 3.892847
C -2.195136 1.472931 3.445429
H -1.677785 1.335323 4.408343
C -1.490126 0.615705 2.384438

H	-0.443635	0.946521	2.281893
H	-1.459511	-0.429739	2.719330
C	-2.131898	2.945635	3.025297
H	-2.610092	3.583454	3.787193
H	-1.081339	3.270476	2.944508
C	-3.683541	0.334835	1.169305
H	-3.768175	-0.717770	1.474769
H	-4.208031	0.436157	0.205089
C	-1.625222	-2.065830	-0.189194
C	-2.994226	-2.435181	-0.807649
H	-3.810353	-1.933299	-0.268980
H	-3.046612	-2.098087	-1.852753
C	-3.210707	-3.956842	-0.765249
H	-4.194778	-4.182839	-1.205746
C	-3.168580	-4.445284	0.688362
H	-3.968260	-3.961301	1.274251
H	-3.352879	-5.531609	0.729672
C	-1.797707	-4.116578	1.294647
H	-1.764003	-4.452285	2.343091
C	-0.695841	-4.816995	0.490890
H	-0.835127	-5.910156	0.527236
H	0.291093	-4.602931	0.933696
C	-0.742629	-4.322875	-0.959892
H	0.055440	-4.806686	-1.545029
C	-0.515711	-2.804185	-0.985319
H	-0.485992	-2.452930	-2.025843
H	0.468920	-2.574512	-0.549456
C	-1.582340	-2.594736	1.262509
H	-0.617199	-2.346757	1.729865
H	-2.366653	-2.119490	1.867679
C	-2.109064	-4.652133	-1.575090
H	-2.143173	-4.318224	-2.625704
H	-2.271210	-5.742728	-1.579070
P	-1.255017	-0.229710	-0.279814
Pd	1.043323	0.262500	-0.027612
Br	1.108473	2.157810	-1.669600
C	3.091022	0.392009	0.554883
C	3.483384	1.385028	1.464083
C	4.056408	-0.202917	-0.257173
C	4.804545	1.799566	1.526386
H	2.745926	1.848446	2.124509
C	5.389169	0.206847	-0.206701
H	3.773898	-0.995174	-0.955352
C	5.766119	1.217366	0.685473
H	5.118038	2.582141	2.219977
H	6.116969	-0.266941	-0.865083
O	7.026639	1.688499	0.815918
C	8.031845	1.172466	-0.031677
H	8.954733	1.708602	0.222736
H	8.190158	0.093764	0.135026
H	7.797772	1.349426	-1.094458
C	1.708009	-0.884051	1.274559
O	1.952736	-1.644499	2.101489

ts-3-cf3.log

SCF (RwB97XD) = -4838.17285512
E(SCF)+ZPE(0 K)= -4837.329988
H(298 K)= -4837.285099
G(298 K)= -4837.409341
Lowest Frequency = -797.2294cm-1

C	-2.278398	1.852043	-1.093335
C	-1.601440	2.863969	-1.781882
H	-0.842798	2.596379	-2.519761
C	-1.893824	4.199190	-1.536106
H	-1.364466	4.984680	-2.079488
C	-3.549703	3.532735	0.103110
H	-4.306632	3.792364	0.844277
C	-3.256454	2.196870	-0.157164
H	-3.783511	1.401298	0.372880
C	0.290462	0.232298	1.102493
H	-0.744059	-0.132009	1.177603
H	0.838232	-0.213498	1.948362
C	0.264856	1.756133	1.226853
H	1.283041	2.153514	1.357314
H	-0.119455	2.205122	0.299719
C	-0.599428	2.224578	2.395705
H	-0.223485	1.776958	3.332499
H	-1.623636	1.836716	2.260866
C	-0.641981	3.741043	2.525823
H	-1.026663	4.206129	1.604822
H	0.361332	4.157416	2.712010
H	-1.292338	4.057564	3.355315
C	0.861369	-2.348834	-0.095576
C	1.054705	-3.130834	-1.416090
H	0.293344	-2.801502	-2.139879
H	2.033647	-2.906333	-1.858212
C	0.928307	-4.642106	-1.183394
H	1.095820	-5.163245	-2.140535
C	-0.479535	-4.953463	-0.663329
H	-1.234026	-4.640546	-1.403829
H	-0.602167	-6.038991	-0.512714
C	-0.698583	-4.206890	0.657332
H	-1.714403	-4.406792	1.032521
C	0.337491	-4.668047	1.690301
H	0.235010	-5.749454	1.880256
H	0.167605	-4.155618	2.651965
C	1.745249	-4.355267	1.167500
H	2.497026	-4.675670	1.906421
C	1.897155	-2.840739	0.937811
H	2.920328	-2.640770	0.588747
H	1.769958	-2.305208	1.892745
C	-0.553287	-2.693365	0.438167
H	-0.731421	-2.190197	1.399912
H	-1.321959	-2.330206	-0.260901
C	1.976231	-5.095307	-0.157670
H	1.908326	-6.184494	-0.000351
H	2.992268	-4.888347	-0.534803
C	2.676542	0.109464	-0.641651
C	3.419730	0.262715	0.707665
H	2.863277	0.922117	1.387406
H	3.510504	-0.709694	1.211649
C	4.822315	0.854349	0.483529
H	5.320967	0.951717	1.460956
C	5.638038	-0.075968	-0.423017
H	5.748346	-1.066210	0.050380
H	6.654562	0.326758	-0.565652
C	4.925707	-0.213625	-1.774884
H	5.494692	-0.896137	-2.426829
C	4.808792	1.162754	-2.440413
H	4.313777	1.069920	-3.421791
H	5.811336	1.584308	-2.622614
C	3.996859	2.091912	-1.530309
H	3.894692	3.081167	-2.003485
C	2.595543	1.500835	-1.324566
H	1.988940	2.192814	-0.723739
H	2.088773	1.400402	-2.297162
C	3.525887	-0.807208	-1.552021
H	3.636981	-1.801289	-1.096714
H	3.020710	-0.945525	-2.518468
C	4.700106	2.235140	-0.174466
H	5.699453	2.681256	-0.309115
H	4.129285	2.917358	0.477456
C	-1.947722	0.384064	-1.291586
O	-2.681090	-0.453654	-0.818964
P	0.910135	-0.507090	-0.459154
Br	-1.792872	0.276587	-4.387666
Pd	-0.336597	-0.120691	-2.370124
H	1.017089	-1.175595	-3.937196
H	1.121415	-0.510147	-3.308958
N	1.193388	-2.068609	-5.046404
C	1.199035	-1.152562	-6.184052
H	0.221906	-0.657930	-6.252113
H	1.965867	-0.380792	-6.023232
H	1.418040	-1.681924	-7.127789
C	0.085059	-3.016431	-5.102006
H	0.110539	-3.665508	-4.216396
H	-0.862445	-2.461296	-5.101202
H	0.139806	-3.649358	-6.005656

C 2.484632 -2.722615 -4.875176
H 3.266393 -1.962092 -4.734808
H 2.460607 -3.368985 -3.988134
H 2.748757 -3.337984 -5.753398
C -2.865376 4.532486 -0.587430
C -3.129519 5.990521 -0.311480
F -2.035302 6.606867 0.184479
F -4.117624 6.182599 0.575507
F -3.467136 6.661797 -1.428902

ts-3-cl.log

SCF (RwB97XD) = -4960.72284596
E(SCF)+ZPE(0 K)= -4959.894322
H(298 K)= -4959.851862
G(298 K)= -4959.969685
Lowest Frequency = -811.2874cm-1

C -2.286513 1.843881 -1.077687
C -1.614793 2.860667 -1.762169
H -0.849311 2.599286 -2.495758
C -1.915419 4.198241 -1.525725
H -1.395257 4.990800 -2.065287
C -3.579570 3.519730 0.109359
H -4.341806 3.787302 0.842663
C -3.272738 2.187442 -0.146624
H -3.798295 1.390558 0.383523
C 0.302273 0.239828 1.104409
H -0.731874 -0.124275 1.185332
H 0.854147 -0.202975 1.949218
C 0.276527 1.764172 1.223026
H 1.294745 2.161982 1.352760
H -0.107951 2.209077 0.294081
C -0.589095 2.237001 2.389092
H -0.213166 1.794738 3.328502
H -1.612577 1.847198 2.254916
C -0.634736 3.754068 2.511444
H -1.022265 4.212959 1.588237
H 0.368040 4.173371 2.694540
H -1.285165 4.073329 3.339817
C 0.867635 -2.344501 -0.090182
C 1.051077 -3.129197 -1.410553
H 0.284432 -2.800792 -2.129134
H 2.026706 -2.905626 -1.860540
C 0.926245 -4.639981 -1.174182
H 1.086476 -5.162933 -2.131616
C -0.477591 -4.950349 -0.642940
H -1.237669 -4.638546 -1.378152
H -0.599049 -6.035651 -0.489584
C -0.686551 -4.201437 0.678026
H -1.699507 -4.400591 1.061299
C 0.357198 -4.661049 1.703897
H 0.256168 -5.742201 1.896260
H 0.194555 -4.147063 2.665997
C 1.760908 -4.348921 1.169999
H 2.518359 -4.668091 1.903687
C 1.910754 -2.834704 0.936699
H 2.931509 -2.635199 0.580551
H 1.789975 -2.297748 1.891679
C -0.542848 -2.688326 0.454898
H -0.713584 -2.183275 1.417021
H -1.316805 -2.326220 -0.238759
C 1.981890 -5.091325 -0.155610
H 1.915249 -6.180250 0.004248
H 2.994973 -4.884962 -0.540938
C 2.679562 0.111533 -0.650666
C 3.428758 0.267174 0.694989
H 2.875891 0.929095 1.375262
H 3.520307 -0.703970 1.201213
C 4.831184 0.856331 0.463713
H 5.334179 0.955093 1.438809
C 5.641712 -0.077062 -0.444355
H 5.752350 -1.066526 0.030621
H 6.658316 0.323717 -0.591996
C 4.923558 -0.216454 -1.792974
H 5.489036 -0.900973 -2.445910
C 4.805607 1.158721 -2.460769
H 4.306289 1.064451 -3.439815
H 5.807924 1.578724 -2.647985
C 3.998488 2.090582 -1.549148
H 3.895495 3.078998 -2.023960
C 2.597346 1.501523 -1.336138
H 1.994156 2.195365 -0.734040
H 2.086093 1.399618 -2.306208
C 3.523905 -0.807653 -1.563145
H 3.635664 -1.801288 -1.107107
H 3.014043 -0.946410 -2.527045
C 4.707952 2.235846 -0.196745
H 5.707304 2.680406 -0.336731
H 4.140807 2.920151 0.456214
C -1.950466 0.381998 -1.275317
O -2.674023 -0.463046 -0.798661
P 0.913035 -0.503236 -0.459097
Br -1.805779 0.274653 -4.378701
Pd -0.342970 -0.119889 -2.364198
H 1.008114 -1.181808 -3.944076
H 1.109517 -0.514294 -3.311150
N 1.177287 -2.070177 -5.046202
C 1.175506 -1.157497 -6.186789
H 0.198537 -0.661669 -6.248246
H 1.944760 -0.386565 -6.034029
H 1.386328 -1.690417 -7.130276
C 0.067796 -3.017526 -5.090918
H 0.099301 -3.663681 -4.203441
H -0.879051 -2.461297 -5.084829
H 0.115966 -3.652983 -5.993044
C 2.469538 -2.724094 -4.880888
H 3.252295 -1.963098 -4.749211
H 2.451115 -3.366303 -3.990696
H 2.727060 -3.343347 -5.758198
C -2.894041 4.515799 -0.584987
Cl -3.259207 6.185626 -0.263637

ts-3-cn.log

SCF (RwB97XD) = -4593.31157469
E(SCF)+ZPE(0 K)= -4592.474670
H(298 K)= -4592.431633
G(298 K)= -4592.550793
Lowest Frequency = -770.4211cm-1

C -2.282170 1.877028 -1.057608
C -1.606206 2.886249 -1.749144
H -0.823358 2.617609 -2.460880
C -1.930123 4.221695 -1.545519
H -1.406574 5.007945 -2.091407
C -3.619666 3.551511 0.071647
H -4.401716 3.818652 0.784209
C -3.289863 2.220565 -0.149748
H -3.813387 1.424461 0.382702
C 0.332020 0.232330 1.128450
H -0.697815 -0.140585 1.221059
H 0.895835 -0.200641 1.970197
C 0.294527 1.757279 1.233174
H 1.308449 2.162428 1.374336
H -0.075990 2.190526 0.292794
C -0.594123 2.238185 2.378216
H -0.228250 1.815337 3.330236
H -1.610915 1.834024 2.235019
C -0.658587 3.756443 2.473971
H -1.033150 4.195147 1.535497
H 0.335865 4.190118 2.667956
H -1.327613 4.083116 3.284302
C 0.880061 -2.350343 -0.083795
C 1.049555 -3.125605 -1.411670
H 0.278970 -2.788645 -2.122086
H 2.022811 -2.903467 -1.867382
C 0.918959 -4.637463 -1.185804
H 1.069101 -5.153678 -2.148465
C -0.482221 -4.944986 -0.646003

H -1.246500 -4.624139 -1.372927
H -0.607783 -6.030740 -0.499835
C -0.677197 -4.205300 0.682208
H -1.687997 -4.402378 1.072096
C 0.372320 -4.677833 1.696293
H 0.267565 -5.759956 1.880672
H 0.219515 -4.170874 2.663691
C 1.773291 -4.368112 1.154028
H 2.534930 -4.696517 1.879159
C 1.928290 -2.852718 0.932029
H 2.947542 -2.654515 0.571067
H 1.815727 -2.323122 1.892073
C -0.527881 -2.691139 0.469753
H -0.688273 -2.193203 1.437343
H -1.305647 -2.319770 -0.214748
C 1.980518 -5.101175 -0.179013
H 1.910256 -6.190917 -0.026718
H 2.991369 -4.896506 -0.570955
C 2.699105 0.103081 -0.640601
C 3.453875 0.252542 0.702554
H 2.906517 0.917180 1.385012
H 3.541704 -0.719754 1.207384
C 4.858406 0.834794 0.466817
H 5.365911 0.928303 1.440030
C 5.660095 -0.100379 -0.447261
H 5.767165 -1.091873 0.024247
H 6.678261 0.295199 -0.597630
C 4.936195 -0.231781 -1.793595
H 5.495672 -0.916987 -2.450863
C 4.821945 1.145941 -2.456695
H 4.318463 1.056951 -3.434120
H 5.825333 1.561937 -2.646664
C 4.023038 2.078733 -1.538847
H 3.922370 3.068970 -2.010274
C 2.619940 1.495693 -1.321079
H 2.023827 2.190461 -0.713030
H 2.103682 1.400152 -2.289204
C 3.534673 -0.816917 -1.560249
H 3.643119 -1.813018 -1.109084
H 3.020543 -0.949257 -2.522859
C 4.739251 2.216724 -0.189291
H 5.740114 2.656539 -0.332798
H 4.178765 2.902268 0.468077
C -1.932628 0.407866 -1.232252
O -2.654094 -0.425976 -0.735046
P 0.932899 -0.507447 -0.438993
Br -1.816175 0.300540 -4.328250
Pd -0.337202 -0.103846 -2.330588
H 0.993902 -1.157880 -3.907486
H 1.109533 -0.496643 -3.284823
N 1.158113 -2.054802 -5.030041
C 1.151626 -1.132648 -6.162489
H 0.175835 -0.633367 -6.214394
H 1.924161 -0.365045 -6.008926
H 1.354654 -1.657379 -7.112500
C 0.047965 -3.000536 -5.078645
H 0.083032 -3.655942 -4.198015
H -0.898939 -2.444530 -5.062924
H 0.090278 -3.627589 -5.987219
C 2.450095 -2.711936 -4.878898
H 3.234844 -1.953815 -4.741923
H 2.435740 -3.365474 -3.996822
H 2.703588 -3.321012 -5.764785
C -2.938791 4.556174 -0.630763
C -3.267950 5.938488 -0.401617
N -3.525344 7.043981 -0.212197

ts-3-d2-cf3.log

SCF (RwB97XD) = -4838.17285512
E(SCF)+ZPE(0 K)= -4837.334276
H(298 K)= -4837.289112
G(298 K)= -4837.413797
Lowest Frequency = -584.6642cm-1

C -2.278398 1.852043 -1.093335
C -1.601440 2.863969 -1.781882
H -0.842798 2.596379 -2.519761
C -1.893824 4.199190 -1.536106
H -1.364466 4.984680 -2.079488
C -3.549703 3.532735 0.103110
H -4.306632 3.792364 0.844277
C -3.256454 2.196870 -0.157164
H -3.783511 1.401298 0.372880
C 0.290462 0.232298 1.102493
H -0.744059 -0.132009 1.177603
H 0.838232 -0.213498 1.948362
C 0.264856 1.756133 1.226853
H 1.283041 2.153514 1.357314
H -0.119455 2.205122 0.299719
C -0.599428 2.224578 2.395705
H -0.223485 1.776958 3.332499
H -1.623636 1.836716 2.260866
C -0.641981 3.741043 2.525823
H -1.026663 4.206129 1.604822
H 0.361332 4.157416 2.712010
H -1.292338 4.057564 3.355315
C 0.861369 -2.348834 -0.095576
C 1.054705 -3.130834 -1.416090
H 0.293344 -2.801502 -2.139879
H 2.033647 -2.906333 -1.858212
C 0.928307 -4.642106 -1.183394
H 1.095820 -5.163245 -2.140535
C -0.479535 -4.953463 -0.663329
H -1.234026 -4.640546 -1.403829
H -0.602167 -6.038991 -0.512714
C -0.698583 -4.206890 0.657332
H -1.714403 -4.406792 1.032521
C 0.337491 -4.668047 1.690301
H 0.235010 -5.749454 1.880256
H 0.167605 -4.155618 2.651965
C 1.745249 -4.355267 1.167500
H 2.497026 -4.675670 1.906421
C 1.897155 -2.840739 0.937811
H 2.920328 -2.640770 0.588747
H 1.769958 -2.305208 1.892745
C -0.553287 -2.693365 0.438167
H -0.731421 -2.190197 1.399912
H -1.321959 -2.330206 -0.260901
C 1.976231 -5.095307 -0.157670
H 1.908326 -6.184494 -0.000351
H 2.992268 -4.888347 -0.534803
C 2.676542 0.109464 -0.641651
C 3.419730 0.262715 0.707665
H 2.863277 0.922117 1.387406
H 3.510504 -0.709694 1.211649
C 4.822315 0.854349 0.483529
H 5.320967 0.951717 1.460956
C 5.638038 -0.075968 -0.423017
H 5.748346 -1.066210 0.050380
H 6.654562 0.326758 -0.565652
C 4.925707 -0.213625 -1.774884
H 5.494692 -0.896137 -2.426829
C 4.808792 1.162754 -2.440413
H 4.313777 1.069920 -3.421791
H 5.811336 1.584308 -2.622614
C 3.996859 2.091912 -1.530309
H 3.894692 3.081167 -2.003485
C 2.595543 1.500835 -1.324566
H 1.988940 2.192814 -0.723739
H 2.088773 1.400402 -2.297162
C 3.525887 -0.807208 -1.552021
H 3.636981 -1.801289 -1.096714
H 3.020710 -0.945525 -2.518468
C 4.700106 2.235140 -0.174466
H 5.699453 2.681256 -0.309115
H 4.129285 2.917358 0.477456
C -1.947722 0.384064 -1.291586
O -2.681090 -0.453654 -0.818964
P 0.910135 -0.507090 -0.459154

Br -1.792872 0.276587 -4.387666
Pd -0.336597 -0.120691 -2.370124
H 1.017089 -1.175595 -3.937196
H 1.121415 -0.510147 -3.308958
N 1.193388 -2.068609 -5.046404
C 1.199035 -1.152562 -6.184052
H 0.221906 -0.657930 -6.252113
H 1.965867 -0.380792 -6.023232
H 1.418040 -1.681924 -7.127789
C 0.085059 -3.016431 -5.102006
H 0.110539 -3.665508 -4.216396
H -0.862445 -2.461296 -5.101202
H 0.139806 -3.649358 -6.005656
C 2.484632 -2.722615 -4.875176
H 3.266393 -1.962092 -4.734808
H 2.460607 -3.368985 -3.988134
H 2.748757 -3.337984 -5.753398
C -2.865376 4.532486 -0.587430
C -3.129519 5.990521 -0.311480
F -2.035302 6.606867 0.184479
F -4.117624 6.182599 0.575507
F -3.467136 6.661797 -1.428902

ts-3-d2-cl.log

SCF (RwB97XD) = -4960.72284596
E(SCF)+ZPE(0 K)= -4959.898611
H(298 K)= -4959.855877
G(298 K)= -4959.974140
Lowest Frequency = -595.1330cm-1

C -2.286513 1.843881 -1.077687
C -1.614793 2.860667 -1.762169
H -0.849311 2.599286 -2.495758
C -1.915419 4.198241 -1.525725
H -1.395257 4.990800 -2.065287
C -3.579570 3.519730 0.109359
H -4.341806 3.787302 0.842663
C -3.272738 2.187442 -0.146624
H -3.798295 1.390558 0.383523
C 0.302273 0.239828 1.104409
H -0.731874 -0.124275 1.185332
H 0.854147 -0.202975 1.949218
C 0.276527 1.764172 1.223026
H 1.294745 2.161982 1.352760
H -0.107951 2.209077 0.294081
C -0.589095 2.237001 2.389092
H -0.213166 1.794738 3.328502
H -1.612577 1.847198 2.254916
C -0.634736 3.754068 2.511444
H -1.022265 4.212959 1.588237
H 0.368040 4.173371 2.694540
H -1.285165 4.073329 3.339817
C 0.867635 -2.344501 -0.090182
C 1.051077 -3.129197 -1.410553
H 0.284432 -2.800792 -2.129134
H 2.026706 -2.905626 -1.860540
C 0.926245 -4.639981 -1.174182
H 1.086476 -5.162933 -2.131616
C -0.477591 -4.950349 -0.642940
H -1.237669 -4.638546 -1.378152
H -0.599049 -6.035651 -0.489584
C -0.686551 -4.201437 0.678026
H -1.699507 -4.400591 1.061299
C 0.357198 -4.661049 1.703897
H 0.256168 -5.742201 1.896260
H 0.194555 -4.147063 2.665997
C 1.760908 -4.348921 1.169999
H 2.518359 -4.668091 1.903687
C 1.910754 -2.834704 0.936699
H 2.931509 -2.635199 0.580551
H 1.789975 -2.297748 1.891679
C -0.542848 -2.688326 0.454898
H -0.713584 -2.183275 1.417021
H -1.316805 -2.326220 -0.238759
C 1.981890 -5.091325 -0.155610
H 1.915249 -6.180250 0.004248
H 2.994973 -4.884962 -0.540938
C 2.679562 0.111533 -0.650666
C 3.428758 0.267174 0.694989
H 2.875891 0.929095 1.375262
H 3.520307 -0.703970 1.201213
C 4.831184 0.856331 0.463713
H 5.334179 0.955093 1.438809
C 5.641712 -0.077062 -0.444355
H 5.752350 -1.066526 0.030621
H 6.658316 0.323717 -0.591996
C 4.923558 -0.216454 -1.792974
H 5.489036 -0.900973 -2.445910
C 4.805607 1.158721 -2.460769
H 4.306289 1.064451 -3.439815
H 5.807924 1.578724 -2.647985
C 3.998488 2.090582 -1.549148
H 3.895495 3.078998 -2.023960
C 2.597346 1.501523 -1.336138
H 1.994156 2.195365 -0.734040
H 2.086093 1.399618 -2.306208
C 3.523905 -0.807653 -1.563145
H 3.635664 -1.801288 -1.107107
H 3.014043 -0.946410 -2.527045
C 4.707952 2.235846 -0.196745
H 5.707304 2.680406 -0.336731
H 4.140807 2.920151 0.456214
C -1.950466 0.381998 -1.275317
O -2.674023 -0.463046 -0.798661
P 0.913035 -0.503236 -0.459097
Br -1.805779 0.274653 -4.378701
Pd -0.342970 -0.119889 -2.364198
H 1.008114 -1.181808 -3.944076
H 1.109517 -0.514294 -3.311150
N 1.177287 -2.070177 -5.046202
C 1.175506 -1.157497 -6.186789
H 0.198537 -0.661669 -6.248246
H 1.944760 -0.386565 -6.034029
H 1.386328 -1.690417 -7.130276
C 0.067796 -3.017526 -5.090918
H 0.099301 -3.663681 -4.203441
H -0.879051 -2.461297 -5.084829
H 0.115966 -3.652983 -5.993044
C 2.469538 -2.724094 -4.880888
H 3.252295 -1.963098 -4.749211
H 2.451115 -3.366303 -3.990696
H 2.727060 -3.343347 -5.758198
C -2.894041 4.515799 -0.584987
Cl -3.259207 6.185626 -0.263637

ts-3-d2-cn.log

SCF (RwB97XD) = -4593.31157469
E(SCF)+ZPE(0 K)= -4592.478976
H(298 K)= -4592.435662
G(298 K)= -4592.555268
Lowest Frequency = -565.3107cm-1

C -2.282170 1.877028 -1.057608
C -1.606206 2.886249 -1.749144
H -0.823358 2.617609 -2.460880
C -1.930123 4.221695 -1.545519
H -1.406574 5.007945 -2.091407
C -3.619666 3.551511 0.071647
H -4.401716 3.818652 0.784209
C -3.289863 2.220565 -0.149748
H -3.813387 1.424461 0.382702
C 0.332020 0.232330 1.128450
H -0.697815 -0.140585 1.221059
H 0.895835 -0.200641 1.970197
C 0.294527 1.757279 1.233174
H 1.308449 2.162428 1.374336
H -0.075990 2.190526 0.292794
C -0.594123 2.238185 2.378216

H	-0.228250	1.815337	3.330236
H	-1.610915	1.834024	2.235019
C	-0.658587	3.756443	2.473971
H	-1.033150	4.195147	1.535497
H	0.335865	4.190118	2.667956
H	-1.327613	4.083116	3.284302
C	0.880061	-2.350343	-0.083795
C	1.049555	-3.125605	-1.411670
H	0.278970	-2.788645	-2.122086
H	2.022811	-2.903467	-1.867382
C	0.918959	-4.637463	-1.185804
H	1.069101	-5.153678	-2.148465
C	-0.482221	-4.944986	-0.646003
H	-1.246500	-4.624139	-1.372927
H	-0.607783	-6.030740	-0.499835
C	-0.677197	-4.205300	0.682208
H	-1.687997	-4.402378	1.072096
C	0.372320	-4.677833	1.696293
H	0.267565	-5.759956	1.880672
H	0.219515	-4.170874	2.663691
C	1.773291	-4.368112	1.154028
H	2.534930	-4.696517	1.879159
C	1.928290	-2.852718	0.932029
H	2.947542	-2.654515	0.571067
H	1.815727	-2.323122	1.892073
C	-0.527881	-2.691139	0.469753
H	-0.688273	-2.193203	1.437343
H	-1.305647	-2.319770	-0.214748
C	1.980518	-5.101175	-0.179013
H	1.910256	-6.190917	-0.026718
H	2.991369	-4.896506	-0.570955
C	2.699105	0.103081	-0.640601
C	3.453875	0.252542	0.702554
H	2.906517	0.917180	1.385012
H	3.541704	-0.719754	1.207384
C	4.858406	0.834794	0.466817
H	5.365911	0.928303	1.440030
C	5.660095	-0.100379	-0.447261
H	5.767165	-1.091873	0.024247
H	6.678261	0.295199	-0.597630
C	4.936195	-0.231781	-1.793595
H	5.495672	-0.916987	-2.450863
C	4.821945	1.145941	-2.456695
H	4.318463	1.056951	-3.434120
H	5.825333	1.561937	-2.646664
C	4.023038	2.078733	-1.538847
H	3.922370	3.068970	-2.010274
C	2.619940	1.495693	-1.321079
H	2.023827	2.190461	-0.713030
H	2.103682	1.400152	-2.289204
C	3.534673	-0.816917	-1.560249
H	3.643119	-1.813018	-1.109084
H	3.020543	-0.949257	-2.522859
C	4.739251	2.216724	-0.189291
H	5.740114	2.656539	-0.332798
H	4.178765	2.902268	0.468077
C	-1.932628	0.407866	-1.232252
O	-2.654094	-0.425976	-0.735046
P	0.932899	-0.507447	-0.438993
Br	-1.816175	0.300540	-4.328250
Pd	-0.337202	-0.103846	-2.330588
H	0.993902	-1.157880	-3.907486
H	1.109533	-0.496643	-3.284823
N	1.158113	-2.054802	-5.030041
C	1.151626	-1.132648	-6.162489
H	0.175835	-0.633367	-6.214394
H	1.924161	-0.365045	-6.008926
H	1.354654	-1.657379	-7.112500
C	0.047965	-3.000536	-5.078645
H	0.083032	-3.655942	-4.198015
H	-0.898939	-2.444530	-5.062924
H	0.090278	-3.627589	-5.987219
C	2.450095	-2.711936	-4.878898
H	3.234844	-1.953815	-4.741923
H	2.435740	-3.365474	-3.996822
H	2.703588	-3.321012	-5.764785
C	-2.938791	4.556174	-0.630763
C	-3.267950	5.938488	-0.401617
N	-3.525344	7.043981	-0.212197

ts-3-d2-nme2.log

SCF (RwB97XD) = -4635.00331017
E(SCF)+ZPE(0 K)= -4634.096334
H(298 K)= -4634.051220
G(298 K)= -4634.174104
Lowest Frequency = -627.7413cm-1

C	-2.368234	1.666254	-1.180798
C	-1.710709	2.722091	-1.824364
H	-0.954270	2.497154	-2.579779
C	-1.987246	4.044050	-1.519331
H	-1.426361	4.821273	-2.036813
C	-3.620512	3.311231	0.100351
H	-4.382789	3.507965	0.853079
C	-3.333849	1.992956	-0.223023
H	-3.865065	1.183920	0.283920
C	0.195834	0.270235	1.011183
H	-0.838704	-0.097730	1.071843
H	0.730498	-0.166337	1.870713
C	0.158014	1.794573	1.132221
H	1.173928	2.203527	1.243629
H	-0.254051	2.237116	0.214711
C	-0.686210	2.251339	2.321044
H	-0.286950	1.803933	3.248687
H	-1.709576	1.856781	2.203491
C	-0.740244	3.767194	2.460418
H	-1.153698	4.232163	1.551803
H	0.263727	4.192243	2.623861
H	-1.370973	4.070979	3.310173
C	0.853708	-2.324980	-0.095808
C	1.072612	-3.145908	-1.388187
H	0.305741	-2.860487	-2.124749
H	2.046933	-2.908665	-1.834509
C	0.987383	-4.651374	-1.105229
H	1.171177	-5.200158	-2.043828
C	-0.412403	-4.983945	-0.576510
H	-1.173575	-4.716542	-1.327852
H	-0.504992	-6.067095	-0.390341
C	-0.654836	-4.200280	0.718521
H	-1.665577	-4.416193	1.098774
C	0.391729	-4.598615	1.766998
H	0.319126	-5.675847	1.992187
H	0.206027	-4.059848	2.711251
C	1.791057	-4.263591	1.235275
H	2.550189	-4.537345	1.985598
C	1.900888	-2.754001	0.953822
H	2.918532	-2.538608	0.598046
H	1.758960	-2.190143	1.890291
C	-0.550731	-2.691360	0.449658
H	-0.741383	-2.162003	1.394864
H	-1.328937	-2.370694	-0.259301
C	2.045096	-5.041309	-0.063762
H	2.006680	-6.126244	0.129512
H	3.055816	-4.818879	-0.446451
C	2.602300	0.160239	-0.705192
C	3.333043	0.359692	0.644781
H	2.754425	1.014731	1.309633
H	3.448345	-0.599849	1.167464
C	4.720434	0.985183	0.418374
H	5.210137	1.114344	1.396812
C	5.567499	0.060495	-0.465053
H	5.702061	-0.916749	0.028688
H	6.573380	0.488681	-0.609893
C	4.867650	-0.123651	-1.817898
H	5.459577	-0.802706	-2.453019
C	4.718132	1.236020	-2.510920
H	4.231655	1.110691	-3.492868
H	5.710401	1.680776	-2.695251
C	3.875246	2.160359	-1.624172

H	3.750282	3.137282	-2.117424
C	2.489423	1.534698	-1.417023
H	1.857372	2.220887	-0.836149
H	1.993807	1.399708	-2.391082
C	3.483016	-0.751023	-1.591325
H	3.618954	-1.732412	-1.115194
H	2.987153	-0.922366	-2.557259
C	4.565582	2.349041	-0.267007
H	5.553758	2.819278	-0.403927
H	3.972066	3.027687	0.368261
C	-2.025474	0.232262	-1.422412
O	-2.732127	-0.656968	-0.997758
P	0.847611	-0.495379	-0.528545
Br	-1.797960	0.141693	-4.534461
Pd	-0.373276	-0.209364	-2.476474
H	1.027571	-1.266837	-4.023552
H	1.104910	-0.571487	-3.399020
N	1.213072	-2.172629	-5.076910
C	1.230707	-1.299247	-6.247845
H	0.252902	-0.810605	-6.344184
H	1.993342	-0.519946	-6.104739
H	1.463196	-1.863044	-7.167878
C	0.105200	-3.123014	-5.106582
H	0.115733	-3.728253	-4.190310
H	-0.841024	-2.567196	-5.146063
H	0.176034	-3.797568	-5.978058
C	2.503127	-2.816812	-4.863076
H	3.280911	-2.048967	-4.742016
H	2.467316	-3.426055	-3.950738
H	2.778746	-3.466098	-5.712449
C	-2.946459	4.381179	-0.534523
N	-3.185654	5.684545	-0.161148
C	-2.886474	6.815918	-1.011541
H	-2.321665	7.583916	-0.457990
H	-2.291516	6.525141	-1.881638
H	-3.815027	7.279370	-1.390925
C	-4.102802	5.970293	0.917178
H	-4.006350	7.028482	1.195038
H	-5.158900	5.785390	0.643338
H	-3.865001	5.374000	1.810168

ts-3-d2-ome.log

SCF (RwB97XD) = -4615.60196072
E(SCF)+ZPE(0 K)= -4614.735534
H(298 K)= -4614.691395
G(298 K)= -4614.812567
Lowest Frequency = -630.3583cm-1

C	-2.291000	1.809628	-1.082155
C	-1.617855	2.841971	-1.751315
H	-0.862717	2.591069	-2.499499
C	-1.901202	4.169862	-1.479172
H	-1.383053	4.976391	-2.000906
C	-3.553497	3.481320	0.156196
H	-4.309051	3.708725	0.907983
C	-3.261767	2.151285	-0.138228
H	-3.791182	1.350275	0.382603
C	0.283521	0.248544	1.088332
H	-0.754348	-0.107000	1.161152
H	0.826184	-0.200798	1.935870
C	0.269399	1.772768	1.212493
H	1.290792	2.162613	1.341201
H	-0.116575	2.223829	0.287345
C	-0.588610	2.247257	2.383638
H	-0.222282	1.787997	3.318820
H	-1.618843	1.878990	2.242223
C	-0.605660	3.763684	2.523054
H	-0.969241	4.238735	1.598323
H	0.402455	4.160807	2.725359
H	-1.261238	4.086358	3.346557
C	0.858294	-2.338984	-0.092247
C	1.046872	-3.130798	-1.407708
H	0.281492	-2.807117	-2.129726
H	2.023146	-2.907465	-1.856625
C	0.924703	-4.640543	-1.163521
H	1.088782	-5.168570	-2.117566
C	-0.479980	-4.950957	-0.634508
H	-1.238468	-4.644255	-1.373470
H	-0.599757	-6.035772	-0.475876
C	-0.694154	-4.195373	0.681890
H	-1.707877	-4.394692	1.063194
C	0.347563	-4.647638	1.713055
H	0.248320	-5.728044	1.910933
H	0.181221	-4.128841	2.671975
C	1.752092	-4.335268	1.181385
H	2.508164	-4.648977	1.918956
C	1.899490	-2.822074	0.939874
H	2.920836	-2.622882	0.585262
H	1.775540	-2.279943	1.891584
C	-0.552734	-2.683247	0.450970
H	-0.726795	-2.173072	1.409852
H	-1.325519	-2.325950	-0.246372
C	1.978282	-5.084434	-0.139520
H	1.913356	-6.172694	0.025878
H	2.992091	-4.878000	-0.522987
C	2.667155	0.115673	-0.657057
C	3.413386	0.274069	0.689944
H	2.858417	0.935419	1.368817
H	3.505612	-0.696316	1.197341
C	4.815571	0.864922	0.461353
H	5.316072	0.966189	1.437566
C	5.629845	-0.069018	-0.442822
H	5.740755	-1.057358	0.034508
H	6.646275	0.333012	-0.588806
C	4.914977	-0.212251	-1.792802
H	5.482963	-0.897515	-2.442904
C	4.797416	1.161454	-2.463720
H	4.300465	1.064673	-3.443698
H	5.799891	1.582048	-2.649268
C	3.986756	2.094248	-1.556175
H	3.884068	3.081622	-2.033260
C	2.585795	1.503991	-1.345988
H	1.979516	2.198609	-0.748005
H	2.077292	1.398734	-2.317052
C	3.515359	-0.804437	-1.565072
H	3.627354	-1.796894	-1.106348
H	3.007413	-0.945735	-2.529529
C	4.692350	2.243016	-0.202093
H	5.691577	2.688748	-0.339895
H	4.122189	2.927624	0.447955
C	-1.965544	0.358211	-1.301379
O	-2.685484	-0.502015	-0.843185
P	0.898847	-0.498937	-0.472811
Br	-1.803517	0.252931	-4.412456
Pd	-0.347725	-0.130462	-2.386472
H	1.022058	-1.196603	-3.978594
H	1.108508	-0.524078	-3.330767
N	1.192634	-2.069862	-5.061641
C	1.193578	-1.162160	-6.206770
H	0.216617	-0.666685	-6.271415
H	1.962544	-0.390906	-6.054950
H	1.406412	-1.700214	-7.146579
C	0.081626	-3.016407	-5.102190
H	0.111393	-3.656498	-4.210364
H	-0.863696	-2.457549	-5.100100
H	0.131351	-3.656810	-6.000376
C	2.484417	-2.723393	-4.887891
H	3.267052	-1.961406	-4.762198
H	2.463234	-3.356181	-3.991069
H	2.741803	-3.351131	-5.758805
C	-2.866554	4.500135	-0.515181
O	-3.057123	5.821178	-0.297491
C	-3.988421	6.215995	0.687850
H	-3.709718	5.837473	1.685532
H	-3.971416	7.313021	0.706918
H	-5.009798	5.881592	0.441296

ts-3-h.log

SCF (RwB97XD) = -4501.10747051
E(SCF)+ZPE(0 K)= -4500.269175
H(298 K)= -4500.227988
G(298 K)= -4500.342441
Lowest Frequency = -830.3174cm-1

C	-2.276301	1.862788	-1.082109
C	-1.591640	2.866207	-1.774884
H	-0.818897	2.587178	-2.494449
C	-1.899295	4.207381	-1.559709
H	-1.365279	4.983299	-2.112710
C	-3.575990	3.562128	0.056527
H	-4.351067	3.834007	0.776833
C	-3.272605	2.221478	-0.166209
H	-3.803774	1.430916	0.368247
C	0.304255	0.229047	1.113611
H	-0.728431	-0.138524	1.195667
H	0.860281	-0.216466	1.954352
C	0.272357	1.752479	1.241504
H	1.289662	2.154421	1.366724
H	-0.122196	2.201478	0.318767
C	-0.587047	2.211192	2.417820
H	-0.203808	1.760104	3.350193
H	-1.610458	1.820635	2.285405
C	-0.636572	3.726645	2.556979
H	-1.041918	4.191810	1.644605
H	0.367495	4.148013	2.728770
H	-1.274685	4.033304	3.399790
C	0.870896	-2.347835	-0.089898
C	1.055539	-3.130903	-1.411015
H	0.288189	-2.802966	-2.128887
H	2.030577	-2.904726	-1.861091
C	0.933714	-4.642233	-1.176534
H	1.094753	-5.163812	-2.134641
C	-0.469494	-4.955978	-0.645580
H	-1.230209	-4.644745	-1.380367
H	-0.588883	-6.041718	-0.493366
C	-0.679773	-4.208812	0.676109
H	-1.692243	-4.410451	1.059417
C	0.365098	-4.667542	1.701357
H	0.266138	-5.749103	1.892721
H	0.201604	-4.154752	2.663959
C	1.768252	-4.352290	1.167684
H	2.526313	-4.671027	1.900991
C	1.915371	-2.837568	0.935786
H	2.935548	-2.635908	0.579017
H	1.794600	-2.301760	1.891411
C	-0.538824	-2.695193	0.455080
H	-0.710009	-2.192406	1.418230
H	-1.313628	-2.331978	-0.237252
C	1.990302	-5.092986	-0.158697
H	1.925620	-6.182236	0.000019
H	3.002947	-4.884329	-0.543983
C	2.676704	0.113076	-0.647514
C	3.428068	0.267813	0.696914
H	2.874176	0.925908	1.380041
H	3.523988	-0.704425	1.200327
C	4.828021	0.862396	0.464546
H	5.332718	0.960411	1.438890
C	5.640013	-0.065821	-0.447543
H	5.754954	-1.056098	0.024734
H	6.654970	0.338851	-0.596217
C	4.919573	-0.204326	-1.795038
H	5.486253	-0.885107	-2.450900
C	4.795685	1.172249	-2.458981
H	4.294728	1.078823	-3.437246
H	5.796289	1.596116	-2.646961
C	3.987028	2.098982	-1.543479
H	3.879811	3.088248	-2.015637
C	2.588506	1.504255	-1.329669
H	1.982772	2.194256	-0.725608
H	2.076245	1.401938	-2.299145
C	3.522385	-0.800976	-1.563874
H	3.638442	-1.795386	-1.110434
H	3.010579	-0.938922	-2.526893
C	4.698678	2.243202	-0.192098
H	5.696241	2.691678	-0.332824
H	4.130284	2.923707	0.463772
C	-1.947363	0.397037	-1.261795
O	-2.668475	-0.443086	-0.772641
P	0.911333	-0.505218	-0.455461
Br	-1.821097	0.245530	-4.371526
Pd	-0.348183	-0.123850	-2.356810
H	1.002360	-1.201095	-3.952057
H	1.098257	-0.535278	-3.308012
N	1.168887	-2.077791	-5.047699
C	1.161130	-1.164594	-6.188136
H	0.182657	-0.671254	-6.245502
H	1.928944	-0.391949	-6.037184
H	1.370191	-1.697163	-7.132064
C	0.061127	-3.027749	-5.088380
H	0.098152	-3.674257	-4.201445
H	-0.886407	-2.472760	-5.077354
H	0.107401	-3.662059	-5.991253
C	2.463530	-2.728113	-4.885284
H	3.244169	-1.964511	-4.756480
H	2.449032	-3.368992	-3.994070
H	2.719844	-3.347489	-5.762663
C	-2.890085	4.557441	-0.641609
H	-3.129439	5.609650	-0.469721

ts-3-me.log

SCF (RwB97XD) = -4540.38767571
E(SCF)+ZPE(0 K)= -4539.522151
H(298 K)= -4539.479138
G(298 K)= -4539.597452
Lowest Frequency = -852.6027cm-1

C	-2.284075	1.823474	-1.075556
C	-1.621409	2.851062	-1.750694
H	-0.865987	2.600723	-2.498811
C	-1.920180	4.184030	-1.479965
H	-1.394167	4.974109	-2.022289
C	-3.543106	3.490874	0.151797
H	-4.294021	3.735846	0.908283
C	-3.254999	2.159948	-0.123920
H	-3.774377	1.357162	0.404012
C	0.304682	0.256472	1.088139
H	-0.732542	-0.099222	1.168746
H	0.852444	-0.189656	1.934080
C	0.290173	1.781012	1.206579
H	1.312472	2.172698	1.322146
H	-0.106631	2.227591	0.283801
C	-0.555018	2.258696	2.385703
H	-0.167476	1.815292	3.320006
H	-1.582715	1.875961	2.265967
C	-0.590499	3.776296	2.506433
H	-0.989576	4.231940	1.586555
H	0.417135	4.190726	2.674111
H	-1.227047	4.099946	3.344153
C	0.865534	-2.334903	-0.090645
C	1.045185	-3.128907	-1.405985
H	0.277744	-2.803250	-2.124922
H	2.020259	-2.909716	-1.859521
C	0.918633	-4.637889	-1.159511
H	1.076258	-5.167448	-2.113802
C	-0.484531	-4.942741	-0.623413
H	-1.245576	-4.634243	-1.358983
H	-0.607259	-6.026944	-0.463015
C	-0.689677	-4.184954	0.693130
H	-1.702187	-4.380220	1.079657
C	0.355415	-4.639626	1.719798
H	0.253334	-5.719452	1.919304
H	0.195471	-4.119202	2.678918
C	1.758414	-4.332729	1.181046
H	2.516977	-4.648245	1.915259
C	1.909768	-2.820269	0.937393
H	2.930210	-2.624773	0.578209
H	1.791671	-2.276947	1.889148

C	-0.544162	-2.673577	0.459632
H	-0.712120	-2.161733	1.418674
H	-1.318962	-2.314906	-0.234766
C	1.975615	-5.084203	-0.140102
H	1.907708	-6.172013	0.026998
H	2.988226	-4.881799	-0.528834
C	2.680531	0.112903	-0.666651
C	3.431602	0.272498	0.677478
H	2.881208	0.938336	1.355786
H	3.521484	-0.696611	1.187746
C	4.835319	0.857155	0.442643
H	5.339401	0.959118	1.416922
C	5.642897	-0.082374	-0.461749
H	5.751376	-1.069913	0.017781
H	6.660462	0.315135	-0.612058
C	4.923131	-0.226325	-1.809043
H	5.486423	-0.915351	-2.459234
C	4.808337	1.146037	-2.483171
H	4.307701	1.048436	-3.461199
H	5.811715	1.562431	-2.673254
C	4.004188	2.084202	-1.575314
H	3.903606	3.070701	-2.054708
C	2.601689	1.499799	-1.358676
H	2.000204	2.197915	-0.759880
H	2.089050	1.394515	-2.327564
C	3.522091	-0.812586	-1.575275
H	3.631718	-1.804438	-1.114744
H	3.010536	-0.954122	-2.537785
C	4.715115	2.233909	-0.224118
H	5.715558	2.675402	-0.366642
H	4.149871	2.922424	0.426096
C	-1.954047	0.368118	-1.295915
O	-2.676091	-0.489210	-0.837741
P	0.911213	-0.495636	-0.473787
Br	-1.800129	0.262221	-4.405966
Pd	-0.340418	-0.123830	-2.383693
H	1.019052	-1.197630	-3.973750
H	1.111323	-0.522309	-3.332477
N	1.183050	-2.078649	-5.056743
C	1.181921	-1.174470	-6.204574
H	0.206471	-0.675662	-6.266335
H	1.954415	-0.405540	-6.058873
H	1.388411	-1.716085	-7.143809
C	0.069169	-3.022020	-5.090492
H	0.101208	-3.660839	-4.197860
H	-0.874685	-2.460696	-5.085108
H	0.112661	-3.664139	-5.987836
C	2.473577	-2.735678	-4.887266
H	3.258951	-1.975858	-4.765429
H	2.454218	-3.367647	-3.989849
H	2.725965	-3.365087	-5.758503
C	-2.881000	4.527652	-0.521969
C	-3.205928	5.964695	-0.215666
H	-2.487808	6.652384	-0.684242
H	-4.210765	6.227327	-0.584156
H	-3.200574	6.151843	0.869270

ts-3-nme2.log

SCF (RwB97XD) = -4635.00331017
E(SCF)+ZPE(0 K)= -4634.092097
H(298 K)= -4634.047256
G(298 K)= -4634.169700
Lowest Frequency = -856.0881cm-1

C	-2.368234	1.666254	-1.180798
C	-1.710709	2.722091	-1.824364
H	-0.954270	2.497154	-2.579779
C	-1.987246	4.044050	-1.519331
H	-1.426361	4.821273	-2.036813
C	-3.620512	3.311231	0.100351
H	-4.382789	3.507965	0.853079
C	-3.333849	1.992956	-0.223023
H	-3.865065	1.183920	0.283920
C	0.195834	0.270235	1.011183
H	-0.838704	-0.097730	1.071843
H	0.730498	-0.166337	1.870713
C	0.158014	1.794573	1.132221
H	1.173928	2.203527	1.243629
H	-0.254051	2.237116	0.214711
C	-0.686210	2.251339	2.321044
H	-0.286950	1.803933	3.248687
H	-1.709576	1.856781	2.203491
C	-0.740244	3.767194	2.460418
H	-1.153698	4.232163	1.551803
H	0.263727	4.192243	2.623861
H	-1.370973	4.070979	3.310173
C	0.853708	-2.324980	-0.095808
C	1.072612	-3.145908	-1.388187
H	0.305741	-2.860487	-2.124749
H	2.046933	-2.908665	-1.834509
C	0.987383	-4.651374	-1.105229
H	1.171177	-5.200158	-2.043828
C	-0.412403	-4.983945	-0.576510
H	-1.173575	-4.716542	-1.327852
H	-0.504992	-6.067095	-0.390341
C	-0.654836	-4.200280	0.718521
H	-1.665577	-4.416193	1.098774
C	0.391729	-4.598615	1.766998
H	0.319126	-5.675847	1.992187
H	0.206027	-4.059848	2.711251
C	1.791057	-4.263591	1.235275
H	2.550189	-4.537345	1.985598
C	1.900888	-2.754001	0.953822
H	2.918532	-2.538608	0.598046
H	1.758960	-2.190143	1.890291
C	-0.550731	-2.691360	0.449658
H	-0.741383	-2.162003	1.394864
H	-1.328937	-2.370694	-0.259301
C	2.045096	-5.041309	-0.063762
H	2.006680	-6.126244	0.129512
H	3.055816	-4.818879	-0.446451
C	2.602300	0.160239	-0.705192
C	3.333043	0.359692	0.644781
H	2.754425	1.014731	1.309633
H	3.448345	-0.599849	1.167464
C	4.720434	0.985183	0.418374
H	5.210137	1.114344	1.396812
C	5.567499	0.060495	-0.465053
H	5.702061	-0.916749	0.028688
H	6.573380	0.488681	-0.609893
C	4.867650	-0.123651	-1.817898
H	5.459577	-0.802706	-2.453019
C	4.718132	1.236020	-2.510920
H	4.231655	1.110691	-3.492868
H	5.710401	1.680776	-2.695251
C	3.875246	2.160359	-1.624172
H	3.750282	3.137282	-2.117424
C	2.489423	1.534698	-1.417023
H	1.857372	2.220887	-0.836149
H	1.993807	1.399708	-2.391082
C	3.483016	-0.751023	-1.591325
H	3.618954	-1.732412	-1.115194
H	2.987153	-0.922366	-2.557259
C	4.565582	2.349041	-0.267007
H	5.553758	2.819278	-0.403927
H	3.972066	3.027687	0.368261
C	-2.025474	0.232262	-1.422412
O	-2.732127	-0.656968	-0.997758
P	0.847611	-0.495379	-0.528545
Br	-1.797960	0.141693	-4.534461
Pd	-0.373276	-0.209364	-2.476474
H	1.027571	-1.266837	-4.023552
H	1.104910	-0.571487	-3.399020
N	1.213072	-2.172629	-5.076910
C	1.230707	-1.299247	-6.247845
H	0.252902	-0.810605	-6.344184
H	1.993342	-0.519946	-6.104739
H	1.463196	-1.863044	-7.167878
C	0.105200	-3.123014	-5.106582

H	0.115733	-3.728253	-4.190310
H	-0.841024	-2.567196	-5.146063
H	0.176034	-3.797568	-5.978058
C	2.503127	-2.816812	-4.863076
H	3.280911	-2.048967	-4.742016
H	2.467316	-3.426055	-3.950738
H	2.778746	-3.466098	-5.712449
C	-2.946459	4.381179	-0.534523
N	-3.185654	5.684545	-0.161148
C	-2.886474	6.815918	-1.011541
H	-2.321665	7.583916	-0.457990
H	-2.291516	6.525141	-1.881638
H	-3.815027	7.279370	-1.390925
C	-4.102802	5.970293	0.917178
H	-4.006350	7.028482	1.195038
H	-5.158900	5.785390	0.643338
H	-3.865001	5.374000	1.810168

ts-3-ome.log

SCF (RwB97XD) = -4615.60196070
E(SCF)+ZPE(0 K)= -4614.731278
H(298 K)= -4614.687412
G(298 K)= -4614.808133
Lowest Frequency = -859.9104cm-1

C	-2.291052	1.809474	-1.082225
C	-1.618111	2.841856	-1.751528
H	-0.863061	2.590992	-2.499814
C	-1.901568	4.169731	-1.479409
H	-1.383597	4.976294	-2.001266
C	-3.553504	3.481090	0.156282
H	-4.308958	3.708443	0.908187
C	-3.261698	2.151073	-0.138149
H	-3.790966	1.350029	0.382784
C	0.283513	0.248623	1.088327
H	-0.754366	-0.106903	1.161125
H	0.826118	-0.200664	1.935931
C	0.269402	1.772855	1.212354
H	1.290800	2.162715	1.340966
H	-0.116620	2.223818	0.287176
C	-0.588541	2.247480	2.383486
H	-0.222279	1.788205	3.318687
H	-1.618823	1.879349	2.242078
C	-0.605383	3.763915	2.522826
H	-0.968910	4.238957	1.598069
H	0.402793	4.160911	2.725076
H	-1.260894	4.086733	3.346324
C	0.858295	-2.338990	-0.092134
C	1.046862	-3.130853	-1.407576
H	0.281477	-2.807196	-2.129604
H	2.023134	-2.907532	-1.856502
C	0.924687	-4.640586	-1.163341
H	1.088764	-5.168634	-2.117375
C	-0.479996	-4.950973	-0.634319
H	-1.238482	-4.644290	-1.373290
H	-0.599772	-6.035784	-0.475660
C	-0.694168	-4.195348	0.682059
H	-1.707889	-4.394650	1.063374
C	0.347546	-4.647595	1.713235
H	0.248293	-5.727995	1.911139
H	0.181211	-4.128773	2.672144
C	1.752075	-4.335247	1.181558
H	2.508147	-4.648942	1.919134
C	1.899471	-2.822058	0.940019
H	2.920829	-2.622869	0.585442
H	1.775484	-2.279916	1.891718
C	-0.552740	-2.683227	0.451085
H	-0.726815	-2.173009	1.409942
H	-1.325516	-2.325959	-0.246282
C	1.978263	-5.084448	-0.139328
H	1.913337	-6.172703	0.026101
H	2.992071	-4.878027	-0.522805
C	2.667202	0.115608	-0.656966
C	3.413348	0.274099	0.690071
H	2.858361	0.935526	1.368853
H	3.505502	-0.696250	1.197553
C	4.815567	0.864879	0.461527
H	5.316002	0.966220	1.437766
C	5.629868	-0.069174	-0.442507
H	5.740705	-1.057474	0.034923
H	6.646325	0.332800	-0.588458
C	4.915088	-0.212505	-1.792523
H	5.483090	-0.897854	-2.442522
C	4.797630	1.161143	-2.463574
H	4.300753	1.064294	-3.443583
H	5.800136	1.581683	-2.649079
C	3.986928	2.094046	-1.556177
H	3.884312	3.081381	-2.033358
C	2.585931	1.503852	-1.346049
H	1.979608	2.198549	-0.748203
H	2.077515	1.398496	-2.317149
C	3.515431	-0.804612	-1.564836
H	3.627356	-1.797038	-1.106030
H	3.007543	-0.945966	-2.529312
C	4.692425	2.242915	-0.202055
H	5.691669	2.688621	-0.339818
H	4.122223	2.927588	0.447889
C	-1.965539	0.358074	-1.301485
O	-2.685509	-0.502180	-0.843382
P	0.898875	-0.498952	-0.472754
Br	-1.803308	0.252663	-4.412568
Pd	-0.347635	-0.130602	-2.386466
H	1.021657	-1.196517	-3.978916
H	1.108677	-0.524564	-3.330503
N	1.192433	-2.069427	-5.062104
C	1.193237	-1.161821	-6.207305
H	0.216142	-0.666639	-6.272106
H	1.961959	-0.390331	-6.055458
H	1.406351	-1.699894	-7.147042
C	0.081653	-3.016247	-5.102609
H	0.111450	-3.656124	-4.210629
H	-0.863791	-2.457595	-5.100751
H	0.131643	-3.656842	-6.000641
C	2.484363	-2.722625	-4.888203
H	3.266839	-1.960423	-4.762848
H	2.463385	-3.355072	-3.991135
H	2.741810	-3.350632	-5.758901
C	-2.866805	4.499946	-0.515281
O	-3.057528	5.820982	-0.297676
C	-3.988409	6.215758	0.688073
H	-3.709257	5.837250	1.685635
H	-3.971450	7.312785	0.707138
H	-5.009882	5.881315	0.441975

ts-4-cf3.log

SCF (RwB97XD) = -4838.18920912
E(SCF)+ZPE(0 K)= -4837.341400
H(298 K)= -4837.296563
G(298 K)= -4837.420968
Lowest Frequency = -562.9742cm-1

C	2.321678	0.473080	-0.143889
H	2.297923	1.500999	-0.531367
H	3.099790	0.437040	0.635753
C	2.689555	-0.436494	-1.315186
H	2.911769	-1.457251	-0.963992
H	1.824000	-0.514562	-1.995657
C	3.891701	0.089280	-2.097052
H	3.644648	1.086932	-2.497102
H	4.739054	0.235632	-1.404722
C	4.316916	-0.830527	-3.234086
H	3.499163	-0.973483	-3.959549
H	4.604970	-1.827118	-2.861283
H	5.176682	-0.421163	-3.785934
C	0.682310	-1.474503	1.337455
C	0.337134	-2.451538	0.182093
H	1.082492	-2.368616	-0.621265
H	-0.636543	-2.178866	-0.248908

C	0.289364	-3.902343	0.682511
H	0.054506	-4.561943	-0.168810
C	1.647277	-4.289527	1.282229
H	2.435466	-4.221767	0.512903
H	1.627145	-5.336169	1.630195
C	1.970390	-3.349400	2.451088
H	2.946784	-3.618374	2.885328
C	0.878187	-3.470809	3.522905
H	0.843785	-4.501447	3.914264
H	1.109781	-2.810818	4.376030
C	-0.478095	-3.088292	2.914373
H	-1.263926	-3.161172	3.683680
C	-0.413192	-1.637061	2.414141
H	-1.387819	-1.334023	2.001548
H	-0.208625	-0.982507	3.273225
C	-0.804658	-4.029664	1.749082
H	-0.871604	-5.070851	2.107235
H	-1.783690	-3.770631	1.314354
C	2.039029	-1.899051	1.942110
H	2.322593	-1.234837	2.770575
H	2.835884	-1.822065	1.186916
C	0.636439	1.560839	1.954673
C	1.049171	2.909470	1.308721
H	2.091029	2.863920	0.959481
H	0.423167	3.109719	0.423516
C	0.937254	4.060565	2.320335
H	1.236788	4.995132	1.819764
C	1.876724	3.779632	3.500938
H	2.920543	3.720798	3.148886
H	1.830103	4.604390	4.231726
C	1.469509	2.459847	4.168816
H	2.143875	2.246990	5.014213
C	0.024209	2.567669	4.675691
H	-0.052957	3.365169	5.433655
H	-0.276612	1.627346	5.168169
C	-0.910320	2.864229	3.494920
H	-1.950353	2.929811	3.851949
C	-0.814721	1.731202	2.464956
H	-1.466971	1.954671	1.608945
H	-1.192753	0.800543	2.908168
C	1.569325	1.304936	3.156001
H	1.296445	0.368965	3.664378
H	2.612489	1.196557	2.815720
C	-0.504231	4.182575	2.827711
H	-1.184279	4.404701	1.988389
H	-0.587610	5.018407	3.542743
P	0.655616	0.253194	0.602122
Pd	-1.108930	0.666270	-0.924525
Br	0.222526	2.183724	-2.673452
C	-3.594699	0.257378	0.679471
C	-3.887477	1.623065	0.669859
C	-4.057497	-0.534904	1.736204
C	-4.620553	2.198042	1.703684
H	-3.524283	2.242835	-0.153718
C	-4.771460	0.034530	2.783825
H	-3.841593	-1.605072	1.731533
H	-4.845722	3.265308	1.685040
H	-5.114258	-0.585893	3.614556
C	-2.722382	-0.377671	-0.382380
O	-2.918889	-1.550110	-0.684091
H	-2.599510	0.635621	-1.617444
H	-0.636110	0.260014	-3.482030
N	-0.912249	-0.513216	-4.130184
C	-2.285724	-0.208565	-4.587274
H	-2.947656	-0.192568	-3.713550
H	-2.288255	0.777579	-5.066009
H	-2.610018	-0.979099	-5.298142
C	-0.850202	-1.786893	-3.374628
H	0.160963	-1.903361	-2.967807
H	-1.574762	-1.755486	-2.548985
H	-1.080963	-2.616020	-4.056407
C	0.064050	-0.476129	-5.239997
H	0.021023	0.508310	-5.719475
H	1.068856	-0.634393	-4.832357
H	-0.179001	-1.264169	-5.963966
C	-5.052671	1.403922	2.766913
C	-5.782336	2.015330	3.933860
F	-4.971694	2.171428	5.003576
F	-6.285639	3.228201	3.653099
F	-6.809801	1.250027	4.348469

ts-4-cl.log

SCF (RwB97XD) = -4960.73849221
E(SCF)+ZPE(0 K)= -4959.904875
H(298 K)= -4959.862572
G(298 K)= -4959.980151
Lowest Frequency = -572.6776cm-1

C	2.309601	0.479029	-0.156085
H	2.285600	1.512252	-0.528895
H	3.094961	0.430077	0.615516
C	2.660965	-0.414076	-1.345030
H	2.879980	-1.442194	-1.013504
H	1.787661	-0.474793	-2.017450
C	3.857331	0.116557	-2.132192
H	3.630916	1.140723	-2.473015
H	4.728056	0.199068	-1.458439
C	4.218197	-0.751333	-3.330436
H	3.382436	-0.803894	-4.047899
H	4.459967	-1.783016	-3.026432
H	5.088864	-0.351879	-3.872306
C	0.685632	-1.474369	1.333266
C	0.337353	-2.452277	0.179936
H	1.078156	-2.365975	-0.627397
H	-0.639667	-2.182862	-0.245180
C	0.298095	-3.903616	0.679492
H	0.060567	-4.563525	-0.170882
C	1.660917	-4.286213	1.270953
H	2.444387	-4.215343	0.497055
H	1.646651	-5.333019	1.618782
C	1.987562	-3.345106	2.438060
H	2.967593	-3.610595	2.866281
C	0.902282	-3.470843	3.516439
H	0.874227	-4.501648	3.907936
H	1.136530	-2.809995	4.368198
C	-0.459102	-3.093570	2.916207
H	-1.240112	-3.169825	3.690100
C	-0.403110	-1.641923	2.416203
H	-1.381476	-1.342713	2.009492
H	-0.196215	-0.987149	3.274536
C	-0.788999	-4.035786	1.752531
H	-0.849564	-5.077425	2.110569
H	-1.771641	-3.780258	1.324039
C	2.047816	-1.894577	1.928427
H	2.335803	-1.229087	2.754422
H	2.838792	-1.815803	1.167010
C	0.631464	1.559217	1.953576
C	1.035604	2.910979	1.308879
H	2.076886	2.871374	0.957020
H	0.406442	3.109443	0.425531
C	0.920558	4.059779	2.322822
H	1.213935	4.996772	1.823086
C	1.864753	3.781590	3.500299
H	2.907890	3.728857	3.145247
H	1.815922	4.604725	4.232825
C	1.466277	2.458378	4.166729
H	2.144014	2.247538	5.009968
C	0.021762	2.558086	4.678085
H	-0.056631	3.353723	5.437979
H	-0.272732	1.615194	5.169572
C	-0.917726	2.852239	3.500584
H	-1.958095	2.911193	3.858936
C	-0.818755	1.722026	2.468314
H	-1.474037	1.944086	1.614636
H	-1.192061	0.788811	2.909947
C	1.569525	1.305953	3.151239
H	1.303897	0.367308	3.658490
H	2.612142	1.204279	2.807089
C	-0.520098	4.173792	2.834548

H -1.203734 4.393619 1.997583
H -0.605434 5.008237 3.551052
P 0.648027 0.253937 0.598530
Pd -1.126471 0.665988 -0.918721
Br 0.195181 2.196226 -2.670768
C -3.599149 0.246301 0.703066
C -3.879408 1.615738 0.708976
C -4.066785 -0.547212 1.755669
C -4.602953 2.192354 1.748465
H -3.513672 2.240814 -0.109912
C -4.771800 0.016974 2.814938
H -3.860755 -1.619611 1.744298
H -4.822377 3.261137 1.749186
H -5.119535 -0.599263 3.645701
C -2.732863 -0.385441 -0.361969
O -2.917909 -1.561301 -0.658497
H -2.621491 0.625704 -1.601246
H -0.607865 0.226714 -3.442554
N -0.871565 -0.550140 -4.091432
C -2.231160 -0.237429 -4.583906
H -2.912906 -0.208039 -3.725839
H -2.213445 0.744650 -5.070472
H -2.544953 -1.011501 -5.295624
C -0.839109 -1.819350 -3.326861
H 0.161248 -1.942170 -2.896123
H -1.581188 -1.776190 -2.517478
H -1.061619 -2.650460 -4.008981
C 0.131995 -0.528838 -5.176858
H 0.113098 0.454610 -5.659947
H 1.124506 -0.699451 -4.743841
H -0.103107 -1.316445 -5.903969
C -5.030568 1.386938 2.802898
Cl -5.900899 2.104915 4.128643

ts-4-cn.log

SCF (RwB97XD) = -4593.32817001
E(SCF)+ZPE(0 K)= -4592.486418
H(298 K)= -4592.443407
G(298 K)= -4592.562788
Lowest Frequency = -563.8985cm-1

C 2.314977 0.478148 -0.151046
H 2.287976 1.505731 -0.539140
H 3.096362 0.443898 0.625358
C 2.679384 -0.431838 -1.323081
H 2.904773 -1.451881 -0.971913
H 1.811207 -0.512013 -2.000012
C 3.877396 0.095423 -2.110306
H 3.625332 1.090361 -2.513987
H 4.725820 0.248342 -1.420753
C 4.303895 -0.827929 -3.243978
H 3.484354 -0.980585 -3.965461
H 4.599805 -1.820516 -2.866737
H 5.158832 -0.416133 -3.801444
C 0.685082 -1.470833 1.339438
C 0.337086 -2.449082 0.185944
H 1.079517 -2.365669 -0.620019
H -0.638451 -2.178299 -0.242073
C 0.293265 -3.899633 0.687484
H 0.056184 -4.560077 -0.162523
C 1.653937 -4.284441 1.282443
H 2.439210 -4.216064 0.510213
H 1.636582 -5.330851 1.631148
C 1.979949 -3.343091 2.449491
H 2.958313 -3.610320 2.880268
C 0.891940 -3.465295 3.525466
H 0.860344 -4.495663 3.917664
H 1.125773 -2.804419 4.377268
C -0.467131 -3.085212 2.921724
H -1.249960 -3.158769 3.694040
C -0.406100 -1.634194 2.420374
H -1.382657 -1.332800 2.011054
H -0.199275 -0.978751 3.278261
C -0.796528 -4.027875 1.758245
H -0.860486 -5.068860 2.117388
H -1.777665 -3.770989 1.326923
C 2.044748 -1.893022 1.939217
H 2.330609 -1.227845 2.766077
H 2.838566 -1.815540 1.180884
C 0.638475 1.565142 1.954903
C 1.049093 2.913484 1.306996
H 2.089682 2.867565 0.954187
H 0.420089 3.113637 0.423943
C 0.940953 4.064984 2.318519
H 1.238726 4.999267 1.816445
C 1.884449 3.784558 3.495948
H 2.927014 3.725493 3.140327
H 1.840383 4.609616 4.226484
C 1.479386 2.465087 4.165693
H 2.156458 2.252437 5.008911
C 0.035870 2.573106 4.677506
H -0.038731 3.370875 5.435348
H -0.262972 1.632927 5.171511
C -0.902743 2.869336 3.499827
H -1.941606 2.936141 3.860672
C -0.811012 1.735722 2.469994
H -1.465741 1.959065 1.615654
H -1.187192 0.804903 2.914727
C 1.575578 1.309738 3.153087
H 1.304673 0.373970 3.662854
H 2.617412 1.201295 2.808878
C -0.498740 4.187418 2.830728
H -1.181478 4.409585 1.993597
H -0.579679 5.023327 3.545830
P 0.653308 0.256303 0.603526
Pd -1.120322 0.667285 -0.913776
Br 0.201356 2.191476 -2.659241
C -3.600868 0.238052 0.695766
C -3.899047 1.604300 0.697423
C -4.058535 -0.567743 1.744345
C -4.632178 2.167197 1.734312
H -3.538249 2.231515 -0.121493
C -4.773805 -0.015545 2.799379
H -3.837057 -1.636529 1.727671
H -4.865256 3.233315 1.732841
H -5.114942 -0.640889 3.626330
C -2.727388 -0.385256 -0.373645
O -2.923709 -1.555773 -0.682789
H -2.612819 0.637175 -1.602203
H -0.640462 0.264516 -3.475153
N -0.914284 -0.508131 -4.124733
C -2.287326 -0.204131 -4.583776
H -2.950905 -0.190525 -3.711260
H -2.290083 0.782844 -5.060786
H -2.609519 -0.973765 -5.296550
C -0.852281 -1.783092 -3.371391
H 0.158482 -1.899939 -2.963754
H -1.577792 -1.753659 -2.546568
H -1.081879 -2.611136 -4.054838
C 0.064172 -0.468030 -5.232609
H 0.019817 0.516600 -5.711559
H 1.068472 -0.624314 -4.822881
H -0.175601 -1.256117 -5.957598
C -5.059587 1.357794 2.797388
C -5.789060 1.939102 3.892610
N -6.370081 2.403200 4.771152

ts-4-h.log

SCF (RwB97XD) = -4501.12302935
E(SCF)+ZPE(0 K)= -4500.280126
H(298 K)= -4500.238838
G(298 K)= -4500.354140
Lowest Frequency = -565.7411cm-1

C 2.342116 0.476142 -0.134666
H 2.322904 1.506869 -0.514665
H 3.118761 0.432537 0.646139
C 2.707849 -0.425668 -1.312428

H 2.925953 -1.450193 -0.969509
H 1.841679 -0.493994 -1.992668
C 3.911222 0.100723 -2.091679
H 3.677034 1.112549 -2.462868
H 4.768219 0.213382 -1.404751
C 4.306638 -0.793969 -3.259137
H 3.481402 -0.889736 -3.983970
H 4.568773 -1.809674 -2.920126
H 5.174764 -0.390803 -3.802487
C 0.698937 -1.472996 1.338329
C 0.358286 -2.449080 0.180969
H 1.106115 -2.364802 -0.619975
H -0.614114 -2.175994 -0.252137
C 0.309338 -3.900475 0.679416
H 0.077615 -4.559176 -0.173538
C 1.665291 -4.288048 1.283276
H 2.456034 -4.219312 0.516632
H 1.644327 -5.335063 1.630307
C 1.984259 -3.348816 2.454008
H 2.959336 -3.617935 2.891308
C 0.888412 -3.471947 3.521991
H 0.853423 -4.503126 3.912114
H 1.116898 -2.812726 4.376636
C -0.466028 -3.089236 2.909372
H -1.254626 -3.163345 3.675724
C -0.400358 -1.637423 2.410987
H -1.374125 -1.334090 1.996435
H -0.198257 -0.984126 3.271669
C -0.788451 -4.029325 1.741922
H -0.856575 -5.071062 2.098554
H -1.765713 -3.769195 1.304063
C 2.053837 -1.897902 1.946553
H 2.335219 -1.234438 2.776442
H 2.852831 -1.820221 1.193557
C 0.649320 1.560965 1.958039
C 1.060406 2.911072 1.314096
H 2.103164 2.868414 0.967069
H 0.435521 3.110326 0.427806
C 0.944030 4.061168 2.326310
H 1.242598 4.996904 1.827246
C 1.881812 3.781217 3.508559
H 2.926432 3.724640 3.158471
H 1.832327 4.605361 4.239999
C 1.476097 2.460019 4.174609
H 2.149213 2.248128 5.021346
C 0.029463 2.564612 4.678649
H -0.050171 3.361589 5.437200
H -0.269806 1.623002 5.169765
C -0.903277 2.859700 3.495908
H -1.945228 2.921581 3.848525
C -0.803148 1.727889 2.465258
H -1.455420 1.949656 1.608994
H -1.181238 0.796512 2.906548
C 1.580192 1.306126 3.161024
H 1.307963 0.369162 3.667993
H 2.624414 1.199967 2.823201
C -0.498734 4.179697 2.830917
H -1.177676 4.400491 1.990488
H -0.584820 5.015099 3.546422
P 0.671856 0.255488 0.603220
Pd -1.092416 0.670017 -0.922990
Br 0.235532 2.175397 -2.698550
C -3.588358 0.246120 0.682143
C -3.879683 1.613943 0.682061
C -4.072386 -0.555954 1.721948
C -4.640105 2.173846 1.706407
H -3.496833 2.240224 -0.128394
C -4.812940 0.006602 2.759259
H -3.852158 -1.625771 1.710220
H -4.867939 3.242224 1.693146
H -5.173136 -0.623780 3.575895
C -2.706519 -0.377680 -0.373797
O -2.881972 -1.553162 -0.679851
H -2.586595 0.638758 -1.605331
H -0.671774 0.247124 -3.468733
N -0.946081 -0.532836 -4.109749
C -2.344759 -0.276917 -4.517653
H -2.977952 -0.295936 -3.623065
H -2.401938 0.713345 -4.984202
H -2.661925 -1.052211 -5.226585
C -0.815839 -1.803945 -3.358194
H 0.211619 -1.887640 -2.986086
H -1.514007 -1.794111 -2.509143
H -1.043044 -2.640239 -4.032445
C -0.011847 -0.460397 -5.253713
H -0.102658 0.524479 -5.725815
H 1.011641 -0.587979 -4.883814
H -0.256209 -1.252747 -5.972564
C -5.098372 1.372945 2.753421
H -5.682092 1.814004 3.564990

ts-4-me.log

SCF (RwB97XD) = -4540.40296042
E(SCF)+ZPE(0 K)= -4539.532313
H(298 K)= -4539.489464
G(298 K)= -4539.607383
Lowest Frequency = -551.9627cm-1

C 2.325841 0.460871 -0.141977
H 2.317042 1.496042 -0.509936
H 3.106344 0.398682 0.633685
C 2.673530 -0.430150 -1.333505
H 2.876816 -1.462953 -1.006430
H 1.803833 -0.475288 -2.011764
C 3.882026 0.088831 -2.109836
H 3.672063 1.118283 -2.445254
H 4.749755 0.154539 -1.430325
C 4.238068 -0.776015 -3.311725
H 3.406527 -0.810860 -4.035146
H 4.462288 -1.813327 -3.013351
H 5.118350 -0.386038 -3.844958
C 0.671030 -1.478796 1.329716
C 0.320331 -2.449015 0.170677
H 1.068121 -2.368398 -0.630770
H -0.650338 -2.167218 -0.260620
C 0.261419 -3.901442 0.664959
H 0.022488 -4.555924 -0.189256
C 1.615777 -4.301281 1.264348
H 2.405195 -4.236622 0.495947
H 1.587475 -5.349028 1.608651
C 1.945017 -3.367531 2.436636
H 2.919188 -3.645237 2.870595
C 0.851071 -3.484875 3.507169
H 0.809608 -4.516631 3.895134
H 1.086784 -2.829223 4.362608
C -0.502132 -3.090582 2.899086
H -1.289306 -3.160999 3.667256
C -0.426941 -1.637948 2.404510
H -1.399431 -1.325984 1.993169
H -0.217766 -0.988645 3.266479
C -0.834666 -4.024919 1.729820
H -0.909963 -5.067152 2.083629
H -1.810919 -3.755845 1.295194
C 2.024434 -1.916078 1.932280
H 2.314540 -1.256775 2.762473
H 2.821108 -1.843337 1.176158
C 0.648335 1.552429 1.961114
C 1.068812 2.901967 1.322104
H 2.110631 2.852745 0.973133
H 0.444334 3.109528 0.437553
C 0.963294 4.048859 2.339084
H 1.268194 4.984190 1.843129
C 1.901052 3.756932 3.518427
H 2.944582 3.694145 3.166206
H 1.858898 4.578272 4.253529
C 1.486757 2.435933 4.179540
H 2.159917 2.215363 5.024031
C 0.041993 2.549603 4.687050
H -0.029669 3.343758 5.449377

H -0.263759 1.608158 5.174471
C -0.890757 2.857214 3.507523
H -1.931465 2.926201 3.862601
C -0.801808 1.729059 2.472015
H -1.453959 1.959706 1.618247
H -1.187081 0.799059 2.909983
C 1.579821 1.285373 3.160959
H 1.301488 0.348603 3.664950
H 2.622421 1.172441 2.820191
C -0.477465 4.176673 2.847049
H -1.156375 4.405773 2.008824
H -0.555608 5.009924 3.566001
P 0.655868 0.252434 0.600295
Pd -1.105057 0.691120 -0.924400
Br 0.249716 2.213932 -2.670948
C -3.596944 0.276693 0.678904
C -3.862245 1.648894 0.701836
C -4.092241 -0.520644 1.716050
C -4.599266 2.211144 1.740018
H -3.473252 2.281140 -0.101155
C -4.809256 0.048216 2.764635
H -3.894592 -1.594884 1.698148
H -4.797607 3.286537 1.738288
H -5.170447 -0.587290 3.578313
C -2.726421 -0.350754 -0.380729
O -2.908086 -1.524564 -0.689311
H -2.596886 0.669351 -1.613137
H -0.578505 0.248693 -3.435468
N -0.846347 -0.522530 -4.089647
C -2.201464 -0.195548 -4.584836
H -2.885601 -0.164658 -3.728728
H -2.173563 0.789127 -5.065629
H -2.519498 -0.962908 -5.301980
C -0.827350 -1.795893 -3.331548
H 0.171061 -1.930221 -2.899796
H -1.570459 -1.749602 -2.522999
H -1.056128 -2.621311 -4.018553
C 0.160751 -0.504187 -5.171602
H 0.153224 0.482458 -5.648361
H 1.150192 -0.687092 -4.736535
H -0.079590 -1.285018 -5.904328
C -5.075311 1.423672 2.796394
C -5.874523 2.032610 3.917210
H -5.592896 3.081578 4.090630
H -6.952225 2.016923 3.684110
H -5.735662 1.480835 4.858411

ts-4-nme2.log

SCF (RwB97XD) = -4635.01972678
E(SCF)+ZPE(0 K)= -4634.103864
H(298 K)= -4634.058185
G(298 K)= -4634.183001
Lowest Frequency = -572.6462cm-1

C 2.266291 0.518373 -0.141955
H 2.221706 1.548101 -0.522862
H 3.035092 0.495596 0.647426
C 2.670963 -0.371433 -1.316287
H 2.915346 -1.389458 -0.971435
H 1.814616 -0.464559 -2.006470
C 3.865602 0.190650 -2.083979
H 3.605413 1.196276 -2.454468
H 4.713505 0.325593 -1.389892
C 4.295442 -0.689002 -3.250688
H 3.479127 -0.803630 -3.983055
H 4.581864 -1.698631 -2.913168
H 5.156513 -0.260633 -3.785922
C 0.654093 -1.475771 1.307113
C 0.360297 -2.460964 0.145056
H 1.121236 -2.358038 -0.641414
H -0.610771 -2.212869 -0.305468
C 0.340030 -3.913417 0.642977
H 0.141123 -4.577548 -0.214156
C 1.694334 -4.265844 1.271644
H 2.496867 -4.177400 0.519416
H 1.694100 -5.312810 1.619668
C 1.967767 -3.317781 2.446655
H 2.941263 -3.561556 2.902217
C 0.856092 -3.468997 3.494700
H 0.840932 -4.500690 3.884956
H 1.051870 -2.803816 4.352962
C -0.496751 -3.122356 2.857380
H -1.296860 -3.217020 3.609334
C -0.460722 -1.669559 2.358914
H -1.434244 -1.392266 1.925427
H -0.292064 -1.011120 3.222823
C -0.773141 -4.071290 1.685276
H -0.819211 -5.114390 2.041651
H -1.748622 -3.837647 1.229335
C 2.008724 -1.865776 1.938950
H 2.259528 -1.195250 2.772779
H 2.818104 -1.768098 1.199225
C 0.520937 1.551764 1.933061
C 0.905960 2.913910 1.298639
H 1.953259 2.898010 0.963004
H 0.286430 3.100630 0.405953
C 0.750045 4.057380 2.312919
H 1.031207 5.001994 1.820432
C 1.680740 3.796498 3.505196
H 2.730333 3.766214 3.166853
H 1.603264 4.616752 4.238715
C 1.299828 2.463585 4.162516
H 1.968334 2.265356 5.016270
C -0.154473 2.531539 4.650415
H -0.262198 3.324374 5.410029
H -0.436349 1.581407 5.135506
C -1.079888 2.806934 3.457450
H -2.127148 2.842030 3.797951
C -0.940708 1.681515 2.424296
H -1.589304 1.889572 1.562356
H -1.301786 0.740044 2.858082
C 1.443232 1.316157 3.146528
H 1.187878 0.371218 3.647478
H 2.493715 1.236470 2.821229
C -0.700879 4.138819 2.801246
H -1.375901 4.344571 1.953854
H -0.815348 4.970065 3.517879
P 0.591281 0.252829 0.571965
Pd -1.154269 0.628157 -0.989508
Br 0.185402 2.180373 -2.735015
C -3.660075 0.159626 0.569983
C -3.943657 1.528843 0.589013
C -4.177566 -0.625791 1.606116
C -4.708632 2.100689 1.595651
H -3.544824 2.164954 -0.206460
C -4.921194 -0.069517 2.637616
H -3.970460 -1.698660 1.607164
H -4.902256 3.171916 1.557629
H -5.279400 -0.721219 3.433743
C -2.755622 -0.458429 -0.452806
O -2.876487 -1.646787 -0.739176
H -2.631297 0.545191 -1.703928
H -0.569788 0.163534 -3.466073
N -0.796228 -0.624497 -4.116002
C -2.147158 -0.346562 -4.650789
H -2.853939 -0.325008 -3.812919
H -2.137434 0.631728 -5.145023
H -2.421786 -1.133614 -5.364508
C -0.759095 -1.886160 -3.339083
H 0.232923 -1.986678 -2.884253
H -1.520046 -1.849113 -2.546525
H -0.950245 -2.726639 -4.019398
C 0.238618 -0.591756 -5.171076
H 0.211612 0.384578 -5.667743
H 1.221232 -0.731776 -4.705676
H 0.045110 -1.394390 -5.894204
C -5.214020 1.314712 2.657683
N -5.958020 1.874405 3.670338
C -6.306009 1.079208 4.825202

H	-6.903451	1.684638	5.517214
H	-6.916239	0.205282	4.545437
H	-5.416351	0.714026	5.371371
C	-6.102095	3.310144	3.740816
H	-6.737347	3.570952	4.595608
H	-5.133214	3.828921	3.864598
H	-6.588616	3.710922	2.837247

ts-4-ome.log

SCF (RwB97XD) = -4615.61643958
E(SCF)+ZPE(0 K)= -4614.740777
H(298 K)= -4614.696968
G(298 K)= -4614.818087
Lowest Frequency = -565.9936cm-1

C	2.340438	0.478944	-0.135297
H	2.321118	1.509577	-0.515632
H	3.115111	0.436489	0.647594
C	2.710188	-0.422810	-1.311826
H	2.930282	-1.446523	-0.967646
H	1.845172	-0.493672	-1.993236
C	3.913209	0.105696	-2.090139
H	3.676540	1.116231	-2.463222
H	4.768991	0.221902	-1.402266
C	4.312612	-0.789744	-3.255686
H	3.488143	-0.889773	-3.980843
H	4.578070	-1.803896	-2.914561
H	5.179708	-0.384571	-3.799217
C	0.694943	-1.471525	1.333091
C	0.359549	-2.448327	0.174864
H	1.110285	-2.363537	-0.623288
H	-0.611601	-2.176137	-0.261575
C	0.310435	-3.899735	0.673366
H	0.082503	-4.558852	-0.180311
C	1.664595	-4.285821	1.282201
H	2.457981	-4.216439	0.518377
H	1.643534	-5.332793	1.629459
C	1.978405	-3.345882	2.453721
H	2.952136	-3.613873	2.894745
C	0.878818	-3.469956	3.517804
H	0.843692	-4.501087	3.908142
H	1.103511	-2.810154	4.373046
C	-0.473896	-3.088970	2.900199
H	-1.265109	-3.164023	3.663873
C	-0.408342	-1.637182	2.401640
H	-1.380859	-1.335131	1.983167
H	-0.210210	-0.983386	3.262882
C	-0.791052	-4.029795	1.731902
H	-0.859025	-5.071585	2.088557
H	-1.766885	-3.770879	1.290210
C	2.048058	-1.895012	1.946182
H	2.326017	-1.231152	2.776867
H	2.849513	-1.816559	1.195874
C	0.642856	1.561296	1.953904
C	1.055719	2.912016	1.312421
H	2.099973	2.870244	0.969694
H	0.434279	3.111180	0.423646
C	0.934027	4.061576	2.324703
H	1.234203	4.997785	1.827494
C	1.866757	3.781615	3.510977
H	2.913005	3.725890	3.165611
H	1.813391	4.605308	4.242670
C	1.459084	2.459748	4.174512
H	2.128753	2.247969	5.024055
C	0.010144	2.563310	4.672599
H	-0.073124	3.359697	5.431388
H	-0.290520	1.621033	5.161769
C	-0.917511	2.858589	3.485963
H	-1.961665	2.920123	3.832617
C	-0.811237	1.727876	2.455203
H	-1.458300	1.950573	1.595939
H	-1.192128	0.795786	2.892772
C	1.568720	1.306458	3.160640
H	1.295022	0.369034	3.666000
H	2.614580	1.201312	2.827531
C	-0.510915	4.179039	2.823306
H	-1.186811	4.399528	1.980393
H	-0.600745	5.014141	3.538668
P	0.667781	0.257115	0.597289
Pd	-1.096316	0.670947	-0.933171
Br	0.238538	2.171004	-2.713885
C	-3.582416	0.243575	0.682265
C	-3.843577	1.620497	0.718366
C	-4.082246	-0.554315	1.712227
C	-4.574204	2.181725	1.753497
H	-3.455603	2.257451	-0.081370
C	-4.798234	-0.003793	2.775064
H	-3.891975	-1.629735	1.685919
H	-4.783440	3.252824	1.781196
H	-5.156221	-0.655892	3.571613
C	-2.710667	-0.377571	-0.375430
O	-2.876059	-1.555883	-0.679092
H	-2.592795	0.639701	-1.609289
H	-0.671889	0.240673	-3.472916
N	-0.945345	-0.541837	-4.111561
C	-2.344551	-0.289211	-4.519538
H	-2.976736	-0.304399	-3.624137
H	-2.403142	0.698772	-4.990712
H	-2.661496	-1.068194	-5.224541
C	-0.813230	-1.810299	-3.355932
H	0.214394	-1.891201	-2.983673
H	-1.511325	-1.798581	-2.506710
H	-1.039223	-2.649089	-4.027538
C	-0.011623	-0.471747	-5.256002
H	-0.103823	0.511562	-5.731101
H	1.012198	-0.596661	-4.886049
H	-0.255099	-1.266688	-5.972328
C	-5.043914	1.374014	2.800368
O	-5.720205	2.013139	3.784767
C	-6.208407	1.254025	4.869423
H	-5.391663	0.757984	5.420528
H	-6.709169	1.962101	5.541743
H	-6.939870	0.496432	4.541649

ts-s1-cf3.log

SCF (RwB97XD) = -4662.58892411
E(SCF)+ZPE(0 K)= -4661.886140
H(298 K)= -4661.848657
G(298 K)= -4661.956291
Lowest Frequency = -268.5208cm-1

C	-1.572302	-1.687477	0.777774
H	-2.507055	-2.092237	0.357512
H	-0.752243	-2.271691	0.334397
C	-1.531999	-1.914909	2.288372
H	-0.654376	-1.407243	2.719281
H	-2.412292	-1.467492	2.774477
C	-1.480517	-3.399892	2.642960
H	-2.366490	-3.904516	2.220267
H	-0.608402	-3.859000	2.148032
C	-1.412831	-3.653235	4.142793
H	-0.524718	-3.177727	4.589420
H	-1.363773	-4.728933	4.369051
H	-2.296749	-3.243694	4.657909
C	-1.533692	-0.190407	-1.705475
C	-0.615738	-1.351005	-2.169375
H	-0.914406	-2.297783	-1.696000
H	0.422501	-1.143912	-1.861670
C	-0.684106	-1.531669	-3.693754
H	-0.021938	-2.365248	-3.978159
C	-2.129158	-1.859852	-4.092970
H	-2.452249	-2.798673	-3.612166
H	-2.196095	-2.017797	-5.182095
C	-3.046034	-0.705566	-3.668499
H	-4.086473	-0.935535	-3.948647
C	-2.597197	0.589303	-4.360206
H	-2.676377	0.481790	-5.454861
H	-3.259271	1.422480	-4.070511

C -1.148826 0.904735 -3.962719
H -0.825521 1.840281 -4.445358
C -1.056544 1.085863 -2.440732
H -0.018579 1.313924 -2.153783
H -1.651234 1.957396 -2.140718
C -0.233239 -0.245596 -4.395281
H -0.270822 -0.376440 -5.489546
H 0.812880 -0.014682 -4.133519
C -2.977464 -0.516298 -2.143192
H -3.656162 0.300023 -1.858068
H -3.337884 -1.427776 -1.638783
C -2.654806 1.063913 0.905823
C -4.003604 0.326195 1.071072
H -4.400682 0.026077 0.090922
H -3.878267 -0.594701 1.658427
C -5.024159 1.232368 1.780834
H -5.972903 0.681160 1.882522
C -5.251411 2.504704 0.953797
H -5.652291 2.244041 -0.040379
H -6.002085 3.146485 1.444475
C -3.922579 3.257328 0.807419
H -4.072382 4.163774 0.199541
C -3.395691 3.647021 2.193707
H -4.115305 4.311346 2.700977
H -2.449867 4.204236 2.096113
C -3.171293 2.374999 3.020159
H -2.779600 2.641391 4.014816
C -2.139470 1.489501 2.306963
H -1.922237 0.607432 2.926316
H -1.195165 2.043912 2.191604
C -2.903126 2.355715 0.093005
H -1.954839 2.895480 -0.049886
H -3.297671 2.107433 -0.902679
C -4.493681 1.610829 3.170192
H -4.340849 0.702447 3.777557
H -5.233174 2.231791 3.702760
P -1.300172 0.018982 0.145767
Pd 0.977598 0.748374 0.596196
Br 0.858737 3.190972 0.168485
C 2.062079 -1.046569 1.212410
C 1.969481 -1.405364 2.562616
C 2.260388 -2.039417 0.242201
C 2.022342 -2.744669 2.938487
H 1.857876 -0.633175 3.327337
C 2.297377 -3.378420 0.612015
H 2.386138 -1.764048 -0.806742
C 2.168427 -3.727098 1.960014
H 1.940125 -3.019566 3.990530
H 2.427242 -4.152223 -0.147355
C 2.814952 0.585055 0.932940
O 3.946361 0.798287 1.021551
C 2.208238 -5.188732 2.337133
F 1.392913 -5.921602 1.556086
F 1.833404 -5.401276 3.606562
F 3.443292 -5.704998 2.196879

ts-s1-cl.log

SCF (RwB97XD) = -4785.13959086
E(SCF)+ZPE(0 K)= -4784.451296
H(298 K)= -4784.416134
G(298 K)= -4784.518330
Lowest Frequency = -271.4551cm-1

C -1.589926 -1.671338 0.838026
H -2.520959 -2.083319 0.416255
H -0.764523 -2.260925 0.411420
C -1.562304 -1.869862 2.352914
H -0.694205 -1.344165 2.781996
H -2.451213 -1.420374 2.821018
C -1.500497 -3.345321 2.741875
H -2.379407 -3.867703 2.325952
H -0.620218 -3.808963 2.265643
C -1.441107 -3.559664 4.248306
H -0.564234 -3.058121 4.689353
H -1.376339 -4.628578 4.502173
H -2.335074 -3.152036 4.747302
C -1.537601 -0.199381 -1.664101
C -0.646873 -1.387522 -2.109471
H -0.977313 -2.321777 -1.632425
H 0.392227 -1.208822 -1.790454
C -0.702302 -1.577551 -3.633115
H -0.058322 -2.429766 -3.903764
C -2.150268 -1.871626 -4.047602
H -2.502925 -2.798864 -3.565165
H -2.208694 -2.034139 -5.136605
C -3.041570 -0.691913 -3.639635
H -4.084702 -0.896737 -3.928972
C -2.552884 0.586652 -4.335021
H -2.623489 0.473386 -5.429679
H -3.196218 1.438588 -4.058082
C -1.101159 0.868572 -3.924850
H -0.748870 1.791526 -4.411468
C -1.021384 1.059290 -2.403539
H 0.019080 1.263548 -2.106645
H -1.597442 1.947673 -2.115718
C -0.210578 -0.308420 -4.338122
H -0.238777 -0.446079 -5.431850
H 0.838114 -0.102863 -4.065603
C -2.984003 -0.495415 -2.114569
H -3.649269 0.335659 -1.841672
H -3.367861 -1.396615 -1.608591
C -2.668625 1.084334 0.924753
C -4.022202 0.352929 1.079974
H -4.408746 0.044474 0.098377
H -3.906856 -0.562659 1.677726
C -5.048611 1.269007 1.768129
H -6.000708 0.722155 1.862169
C -5.260052 2.534378 0.926254
H -5.647574 2.265538 -0.071068
H -6.015665 3.182884 1.400327
C -3.926971 3.281704 0.791423
H -4.065809 4.183359 0.173842
C -3.417400 3.681645 2.181194
H -4.141741 4.352114 2.673542
H -2.468718 4.235139 2.091552
C -3.207717 2.415895 3.020788
H -2.827601 2.689428 4.018007
C -2.169946 1.520484 2.328762
H -1.964897 0.643933 2.959456
H -1.222323 2.070219 2.221107
C -2.900458 2.371222 0.098657
H -1.947953 2.906300 -0.032962
H -3.280526 2.118581 -0.901360
C -4.534882 1.658190 3.160764
H -4.393607 0.754739 3.778233
H -5.278773 2.286834 3.678045
P -1.311183 0.026642 0.186297
Pd 0.970316 0.739112 0.641708
Br 0.845793 3.196187 0.285090
C 2.033735 -1.086746 1.151896
C 1.918726 -1.551361 2.469911
C 2.287970 -2.003515 0.122027
C 2.003933 -2.910664 2.751675
H 1.767275 -0.843177 3.287983
C 2.362282 -3.366652 0.389368
H 2.437651 -1.652044 -0.901215
C 2.211643 -3.808236 1.704363
H 1.906178 -3.275496 3.775128
H 2.543940 -4.082564 -0.413495
C 2.805063 0.564708 0.998302
O 3.932459 0.770752 1.135744
Cl 2.290555 -5.506056 2.046283

ts-s1-cn.log

SCF (RwB97XD) = -4417.72620525
E(SCF)+ZPE(0 K)= -4417.029417
H(298 K)= -4416.993777
G(298 K)= -4417.096463

Lowest Frequency = -266.6544cm-1

C	-1.611378	-1.660680	0.903047
H	-2.544499	-2.074918	0.488238
H	-0.789450	-2.260887	0.484361
C	-1.582817	-1.833399	2.420791
H	-0.709737	-1.306209	2.838491
H	-2.466291	-1.366970	2.882430
C	-1.534556	-3.301968	2.836914
H	-2.427204	-3.819302	2.445207
H	-0.669896	-3.787982	2.353602
C	-1.452836	-3.489336	4.346098
H	-0.557775	-2.999457	4.763594
H	-1.408221	-4.554373	4.619451
H	-2.328403	-3.052219	4.852536
C	-1.532590	-0.227371	-1.620996
C	-0.651067	-1.432569	-2.038306
H	-1.002003	-2.355861	-1.554778
H	0.386178	-1.266210	-1.706497
C	-0.688932	-1.642162	-3.559883
H	-0.051716	-2.505417	-3.810734
C	-2.134756	-1.925590	-3.989247
H	-2.503857	-2.842639	-3.499828
H	-2.181187	-2.101239	-5.076750
C	-3.017678	-0.731027	-3.607593
H	-4.059481	-0.927969	-3.906712
C	-2.506327	0.532732	-4.313703
H	-2.565251	0.405469	-5.407430
H	-3.143023	1.395578	-4.056004
C	-1.056353	0.804285	-3.890114
H	-0.687897	1.716196	-4.385457
C	-0.993755	1.015675	-2.370776
H	0.045371	1.213175	-2.063299
H	-1.564846	1.913646	-2.102861
C	-0.173908	-0.388377	-4.275434
H	-0.189482	-0.540942	-5.367371
H	0.873634	-0.190854	-3.992334
C	-2.976504	-0.515608	-2.084584
H	-3.638008	0.324055	-1.829706
H	-3.373533	-1.407127	-1.571581
C	-2.686974	1.098273	0.937273
C	-4.043019	0.371344	1.090335
H	-4.419512	0.045530	0.110440
H	-3.934238	-0.533436	1.705872
C	-5.075459	1.300926	1.751191
H	-6.029684	0.757577	1.843176
C	-5.274282	2.552327	0.885589
H	-5.649796	2.267322	-0.111821
H	-6.034585	3.209759	1.339363
C	-3.938629	3.295706	0.754589
H	-4.068732	4.187408	0.120924
C	-3.444331	3.717081	2.143504
H	-4.173170	4.396179	2.616887
H	-2.494039	4.268018	2.056229
C	-3.246593	2.464547	3.005498
H	-2.876506	2.753389	4.002138
C	-2.203351	1.555565	2.339904
H	-2.008703	0.688828	2.987266
H	-1.253395	2.101365	2.235188
C	-2.905739	2.372742	0.088461
H	-1.950572	2.904101	-0.038828
H	-3.273602	2.106212	-0.912446
C	-4.577136	1.712584	3.142966
H	-4.445805	0.819601	3.777596
H	-5.325271	2.352031	3.640401
P	-1.325760	0.026497	0.227092
Pd	0.949162	0.746921	0.715921
Br	0.839003	3.202648	0.391113
C	2.076702	-1.082282	1.150984
C	1.899698	-1.670079	2.411192
C	2.394532	-1.884276	0.045814
C	1.987989	-3.048795	2.559318
H	1.700282	-1.044449	3.283840
C	2.475786	-3.264858	0.181316
H	2.585215	-1.424544	-0.926363
C	2.266396	-3.846399	1.439970
H	1.841954	-3.511864	3.536391
H	2.706694	-3.893364	-0.680172
C	2.765316	0.589608	1.154242
O	3.869587	0.830789	1.392874
C	2.339959	-5.276612	1.586760
N	2.389017	-6.419700	1.704550

ts-s1-h.log

SCF (RwB97XD) = -4325.52559723
E(SCF)+ZPE(0 K)= -4324.827371
H(298 K)= -4324.793539
G(298 K)= -4324.891746
Lowest Frequency = -275.1211cm-1

C	-1.604120	-1.661144	0.889813
H	-2.533157	-2.077747	0.467820
H	-0.775289	-2.255463	0.476221
C	-1.584162	-1.837632	2.407288
H	-0.713973	-1.310564	2.830064
H	-2.471458	-1.375155	2.865741
C	-1.533367	-3.307102	2.819621
H	-2.422528	-3.826408	2.421802
H	-0.661828	-3.785455	2.341611
C	-1.459212	-3.496692	4.329079
H	-0.563068	-3.011560	4.749492
H	-1.420448	-4.562279	4.602199
H	-2.334964	-3.056624	4.832920
C	-1.535488	-0.216954	-1.628019
C	-0.660952	-1.424203	-2.052927
H	-1.014632	-2.347616	-1.571625
H	0.376911	-1.264666	-1.722195
C	-0.703276	-1.627324	-3.575152
H	-0.069845	-2.491996	-3.830874
C	-2.151021	-1.902645	-4.002882
H	-2.523082	-2.820191	-3.516473
H	-2.200968	-2.073178	-5.091169
C	-3.027750	-0.705985	-3.613466
H	-4.071266	-0.896904	-3.910896
C	-2.512662	0.558667	-4.315368
H	-2.574851	0.436494	-5.409598
H	-3.145144	1.423020	-4.052242
C	-1.060534	0.822216	-3.894053
H	-0.689222	1.734533	-4.386631
C	-0.993084	1.026668	-2.374028
H	0.047632	1.217537	-2.068111
H	-1.558563	1.926452	-2.100081
C	-0.184339	-0.372768	-4.286597
H	-0.202978	-0.520339	-5.379298
H	0.864537	-0.181395	-4.004534
C	-2.981598	-0.497256	-2.089543
H	-3.639744	0.343509	-1.830031
H	-3.380704	-1.389557	-1.579373
C	-2.683601	1.096006	0.935928
C	-4.039128	0.367369	1.086744
H	-4.416472	0.046185	0.105698
H	-3.929393	-0.540475	1.697433
C	-5.072199	1.292471	1.752827
H	-6.025777	0.747531	1.843410
C	-5.273585	2.547403	0.892938
H	-5.649688	2.266143	-0.105364
H	-6.034375	3.201950	1.350266
C	-3.938881	3.292809	0.763817
H	-4.070711	4.187145	0.134109
C	-3.444307	3.709050	2.154195
H	-4.174024	4.384928	2.631079
H	-2.494979	4.261768	2.068507
C	-3.243664	2.453069	3.010461
H	-2.873263	2.738239	4.008089
C	-2.199714	1.548490	2.340044
H	-2.002757	0.679538	2.983585
H	-1.250509	2.095746	2.236161
C	-2.905063	2.374120	0.093157
H	-1.950635	2.907160	-0.031973

H	-3.272942	2.111924	-0.908888
C	-4.573071	1.698658	3.145948
H	-4.439582	0.802895	3.776239
H	-5.321743	2.334684	3.647162
P	-1.319728	0.029003	0.220963
Pd	0.954415	0.748410	0.703877
Br	0.821625	3.215164	0.381845
C	2.026771	-1.094802	1.110266
C	1.880377	-1.691001	2.370873
C	2.361055	-1.888257	0.003257
C	2.014055	-3.070412	2.510197
H	1.672511	-1.071223	3.246402
C	2.483216	-3.268485	0.143761
H	2.534733	-1.421181	-0.969110
C	2.304930	-3.858975	1.395806
H	1.893487	-3.532444	3.492307
H	2.730142	-3.883756	-0.724109
C	2.774054	0.588015	1.132287
O	3.882108	0.805456	1.368167
H	2.409919	-4.940511	1.507482

ts-s1-me.log

SCF (RwB97XD) = -4364.80667626
E(SCF)+ZPE(0 K)= -4364.081030
H(298 K)= -4364.045406
G(298 K)= -4364.147681
Lowest Frequency = -271.6162cm-1

C	-1.587046	-1.674861	0.820691
H	-2.511787	-2.089677	0.387802
H	-0.753101	-2.255934	0.399399
C	-1.571334	-1.881916	2.334656
H	-0.716117	-1.345325	2.775143
H	-2.473133	-1.451895	2.796719
C	-1.484306	-3.358559	2.713732
H	-2.342847	-3.898130	2.277266
H	-0.581970	-3.793038	2.251663
C	-1.447072	-3.583124	4.219364
H	-0.588881	-3.065994	4.678402
H	-1.364449	-4.652393	4.467680
H	-2.358358	-3.198719	4.705282
C	-1.523631	-0.189602	-1.672294
C	-0.626305	-1.372602	-2.118049
H	-0.957387	-2.310355	-1.648303
H	0.409831	-1.193087	-1.790350
C	-0.671028	-1.554497	-3.643031
H	-0.022599	-2.403248	-3.914048
C	-2.115276	-1.850413	-4.069219
H	-2.468602	-2.781344	-3.594297
H	-2.165941	-2.007001	-5.159547
C	-3.012844	-0.675596	-3.660825
H	-4.053468	-0.881853	-3.958295
C	-2.523119	0.608160	-4.345935
H	-2.586026	0.500863	-5.441712
H	-3.170747	1.456701	-4.068515
C	-1.075071	0.891921	-3.924290
H	-0.722113	1.818554	-4.403451
C	-1.006248	1.074410	-2.401528
H	0.031328	1.280016	-2.096094
H	-1.586591	1.959666	-2.112621
C	-0.178214	-0.280187	-4.337879
H	-0.198462	-0.411937	-5.432591
H	0.867859	-0.073156	-4.056755
C	-2.966006	-0.487284	-2.134303
H	-3.635583	0.340351	-1.861472
H	-3.350822	-1.392319	-1.635845
C	-2.677546	1.075183	0.914665
C	-4.029633	0.338441	1.056466
H	-4.408741	0.034854	0.070419
H	-3.915061	-0.580526	1.649192
C	-5.063769	1.246741	1.743601
H	-6.014706	0.696204	1.828130
C	-5.274073	2.516665	0.908284
H	-5.653925	2.252738	-0.093325
H	-6.035087	3.159655	1.381346
C	-3.942634	3.269129	0.786686
H	-4.080458	4.174210	0.173852
C	-3.443116	3.662235	2.182068
H	-4.172421	4.327726	2.673956
H	-2.495463	4.218850	2.101519
C	-3.234805	2.392066	3.015271
H	-2.862264	2.660714	4.016693
C	-2.189902	1.504135	2.324699
H	-1.986479	0.624141	2.951083
H	-1.242899	2.056805	2.226574
C	-2.908608	2.366319	0.095102
H	-1.957093	2.905300	-0.027056
H	-3.281321	2.118534	-0.908940
C	-4.560411	1.629229	3.141937
H	-4.420077	0.722475	3.754793
H	-5.309759	2.252300	3.658197
P	-1.309395	0.027479	0.180924
Pd	0.963206	0.748723	0.655527
Br	0.824710	3.210811	0.284385
C	1.992465	-1.075770	1.190336
C	1.889820	-1.518265	2.516643
C	2.268395	-2.011476	0.183126
C	2.010155	-2.871006	2.817536
H	1.724566	-0.796871	3.320841
C	2.374306	-3.364032	0.490232
H	2.413387	-1.679096	-0.847447
C	2.240914	-3.817308	1.810422
H	1.923958	-3.200670	3.856084
H	2.577642	-4.082060	-0.308614
C	2.797279	0.579131	1.022511
O	3.925456	0.775146	1.160534
C	2.320467	-5.282761	2.135557
H	2.646507	-5.449703	3.171860
H	1.330990	-5.756391	2.021333
H	3.014287	-5.809323	1.464704

ts-s1-nme2.log

SCF (RwB97XD) = -4459.42705402
E(SCF)+ZPE(0 K)= -4458.655422
H(298 K)= -4458.617251
G(298 K)= -4458.725068
Lowest Frequency = -275.2418cm-1

C	-1.440222	-1.671883	0.748811
H	-2.364642	-2.105342	0.334169
H	-0.602088	-2.222854	0.297486
C	-1.379291	-1.907215	2.257595
H	-0.519410	-1.369184	2.684711
H	-2.273835	-1.501326	2.754259
C	-1.262094	-3.391164	2.600985
H	-2.117288	-3.934531	2.162258
H	-0.358224	-3.801416	2.119786
C	-1.205951	-3.648893	4.100803
H	-0.342524	-3.140492	4.559370
H	-1.123302	-4.723602	4.325271
H	-2.111444	-3.275154	4.605795
C	-1.507172	-0.159768	-1.723846
C	-0.548781	-1.272052	-2.221564
H	-0.781114	-2.233731	-1.740841
H	0.485172	-1.010771	-1.946067
C	-0.655028	-1.451748	-3.743712
H	0.036973	-2.252322	-4.051428
C	-2.094164	-1.845410	-4.102242
H	-2.358525	-2.799854	-3.616157
H	-2.186418	-2.002930	-5.189705
C	-3.051000	-0.736630	-3.645337
H	-4.087462	-1.013889	-3.896512
C	-2.683296	0.580240	-4.343049
H	-2.790313	0.473753	-5.435582
H	-3.374995	1.379950	-4.028924
C	-1.239922	0.961125	-3.985711
H	-0.974985	1.912529	-4.473050
C	-1.111031	1.140098	-2.465882

H	-0.077159	1.414572	-2.207169
H	-1.735861	1.981963	-2.144371
C	-0.285088	-0.143511	-4.451573
H	-0.347968	-0.271413	-5.545203
H	0.756165	0.134547	-4.218214
C	-2.946524	-0.549441	-2.121916
H	-3.653168	0.234212	-1.813746
H	-3.250866	-1.477942	-1.611912
C	-2.602656	1.043168	0.919137
C	-3.920100	0.258347	1.120662
H	-4.335610	-0.051307	0.151035
H	-3.746764	-0.659937	1.698977
C	-4.950998	1.125183	1.864231
H	-5.875197	0.538883	1.994158
C	-5.250162	2.388494	1.046656
H	-5.671469	2.113199	0.064892
H	-6.008266	3.003188	1.560277
C	-3.954196	3.188280	0.861126
H	-4.154986	4.088423	0.258547
C	-3.400707	3.597183	2.231284
H	-4.128575	4.234926	2.760695
H	-2.479554	4.188779	2.105071
C	-3.104959	2.334223	3.049550
H	-2.693985	2.615428	4.032317
C	-2.063731	1.485472	2.306080
H	-1.799486	0.609963	2.916752
H	-1.141782	2.070580	2.165052
C	-2.923250	2.325286	0.116323
H	-2.000611	2.900373	-0.053342
H	-3.337807	2.062678	-0.867533
C	-4.393277	1.522084	3.237907
H	-4.188161	0.619605	3.838781
H	-5.139422	2.114575	3.793485
P	-1.230540	0.045702	0.123078
Pd	1.028946	0.827981	0.511393
Br	0.777864	3.287048	0.092753
C	2.009383	-0.969201	1.060804
C	2.028799	-1.345973	2.413150
C	2.262474	-1.965142	0.105377
C	2.213750	-2.664325	2.796877
H	1.896498	-0.587246	3.189397
C	2.432438	-3.292219	0.468520
H	2.326123	-1.701687	-0.952970
C	2.386763	-3.684808	1.829631
H	2.216253	-2.902468	3.859566
H	2.607655	-4.028575	-0.314877
C	2.890101	0.713178	0.790754
O	4.025449	0.880209	0.868791
N	2.501629	-4.994967	2.196146
C	2.445418	-5.364197	3.593297
H	1.499740	-5.042807	4.061354
H	2.508753	-6.454301	3.684385
H	3.279573	-4.928891	4.170049
C	2.687587	-6.017207	1.190717
H	1.847723	-6.045435	0.475585
H	3.619976	-5.870172	0.619408
H	2.745546	-6.998130	1.675646

ts-s1-ome.log

SCF (RwB97XD) = -4440.02103466
E(SCF)+ZPE(0 K)= -4439.290214
H(298 K)= -4439.253758
G(298 K)= -4439.358124
Lowest Frequency = -272.0581cm-1

C	-1.586906	-1.670658	0.833081
H	-2.515079	-2.084002	0.406138
H	-0.756776	-2.256555	0.410843
C	-1.565493	-1.870789	2.347798
H	-0.704376	-1.338349	2.781483
H	-2.461311	-1.430745	2.811910
C	-1.489841	-3.345827	2.735673
H	-2.356894	-3.879388	2.308544
H	-0.595357	-3.795147	2.272154
C	-1.445372	-3.559609	4.242643
H	-0.577307	-3.050094	4.691457
H	-1.372909	-4.627781	4.497829
H	-2.348591	-3.160294	4.731790
C	-1.537180	-0.196111	-1.666452
C	-0.649931	-1.385860	-2.113973
H	-0.981693	-2.319454	-1.636456
H	0.389339	-1.209668	-1.795528
C	-0.707785	-1.575028	-3.637567
H	-0.065818	-2.428440	-3.909655
C	-2.156796	-1.865558	-4.050779
H	-2.511200	-2.792251	-3.568411
H	-2.217050	-2.027151	-5.139893
C	-3.044834	-0.684089	-3.640720
H	-4.088847	-0.886279	-3.928928
C	-2.554092	0.593758	-4.336020
H	-2.626371	0.481310	-5.430716
H	-3.195189	1.446927	-4.057700
C	-1.101317	0.872165	-3.927236
H	-0.747394	1.794567	-4.413757
C	-1.019221	1.061659	-2.405938
H	0.021789	1.263429	-2.109741
H	-1.592621	1.951255	-2.116486
C	-0.213931	-0.306609	-4.342410
H	-0.243636	-0.443410	-5.436286
H	0.835412	-0.103737	-4.070569
C	-2.984732	-0.488659	-2.115563
H	-3.648033	0.343580	-1.841678
H	-3.370104	-1.389303	-1.609671
C	-2.665931	1.084827	0.922965
C	-4.019831	0.353718	1.077591
H	-4.407146	0.047220	0.095695
H	-3.904781	-0.562946	1.673617
C	-5.045819	1.268949	1.767626
H	-5.997996	0.722075	1.861462
C	-5.257669	2.535535	0.927678
H	-5.645874	2.268139	-0.069807
H	-6.012902	3.183538	1.403168
C	-3.924442	3.282573	0.792974
H	-4.063333	4.185103	0.176610
C	-3.414070	3.680754	2.182978
H	-4.138022	4.350866	2.676524
H	-2.465275	4.233999	2.093368
C	-3.204173	2.413941	3.020916
H	-2.823657	2.686198	4.018349
C	-2.166979	1.519046	2.327483
H	-1.962184	0.641478	2.956815
H	-1.218918	2.068111	2.220062
C	-2.898378	2.372776	0.098669
H	-1.945957	2.907848	-0.032647
H	-3.278954	2.121470	-0.901530
C	-4.531432	1.656304	3.160544
H	-4.389877	0.752022	3.776735
H	-5.275119	2.284359	3.678954
P	-1.307096	0.028037	0.184399
Pd	0.970186	0.739245	0.639064
Br	0.829863	3.203366	0.261501
C	1.966200	-1.094034	1.144398
C	1.886762	-1.562771	2.468895
C	2.241573	-2.014218	0.126694
C	2.023120	-2.910282	2.753679
H	1.722943	-0.856468	3.286659
C	2.366574	-3.375292	0.395835
H	2.370381	-1.667824	-0.901520
C	2.249486	-3.830207	1.716125
H	1.955576	-3.281010	3.778001
H	2.567838	-4.064192	-0.424006
O	2.349959	-5.121130	2.086845
C	2.577089	-6.099546	1.090257
H	2.621688	-7.063543	1.611491
H	1.755439	-6.126463	0.356114
H	3.532930	-5.931469	0.567900
C	2.808613	0.565429	0.996800
O	3.938254	0.742385	1.141172

ts-s2-ome.log

SCF (RwB97XD) = -4615.60333400
E(SCF)+ZPE(0 K)= -4614.732089
H(298 K)= -4614.688419
G(298 K)= -4614.807754
Lowest Frequency = -429.2819cm-1

C	0.222047	-0.410357	1.866290
H	-0.482919	-1.216834	2.107930
H	1.071658	-0.522356	2.557808
C	-0.489277	0.925491	2.091794
H	0.241098	1.746518	2.164721
H	-1.124847	1.166717	1.227901
C	-1.362006	0.919207	3.344676
H	-2.090879	0.094489	3.265171
H	-0.739298	0.694142	4.228285
C	-2.105041	2.232738	3.547601
H	-2.738358	2.462703	2.675710
H	-1.406067	3.074234	3.681630
H	-2.756445	2.196654	4.434313
C	2.024037	0.542334	-0.262272
C	1.215766	1.744398	-0.818096
H	0.483864	2.093376	-0.075218
H	0.650858	1.420073	-1.705426
C	2.151809	2.900678	-1.197805
H	1.541085	3.736495	-1.574764
C	2.945120	3.351480	0.035930
H	2.257343	3.709934	0.820593
H	3.604304	4.196931	-0.223422
C	3.778168	2.175134	0.561997
H	4.344397	2.489708	1.453585
C	4.748730	1.704695	-0.528933
H	5.439149	2.520765	-0.800734
H	5.367096	0.871629	-0.153420
C	3.946039	1.254410	-1.755424
H	4.631350	0.896080	-2.540025
C	3.017805	0.095417	-1.359233
H	2.465553	-0.262257	-2.239648
H	3.637905	-0.735667	-0.993797
C	3.117326	2.428093	-2.291225
H	3.780952	3.254307	-2.597317
H	2.552515	2.115097	-3.183966
C	2.841867	1.021013	0.961996
H	3.436895	0.194918	1.377293
H	2.179133	1.371661	1.765065
C	1.594074	-2.475200	0.357832
C	0.581773	-3.416467	1.060893
H	0.347480	-3.048774	2.070383
H	-0.367806	-3.432195	0.504747
C	1.154826	-4.835527	1.192341
H	0.405928	-5.467455	1.696045
C	2.438226	-4.783796	2.031789
H	2.214980	-4.401664	3.042160
H	2.854942	-5.797281	2.155933
C	3.460302	-3.875381	1.336675
H	4.383266	-3.826189	1.936409
C	3.778115	-4.434775	-0.057065
H	4.223996	-5.439643	0.031184
H	4.521967	-3.795603	-0.561835
C	2.489078	-4.496166	-0.887442
H	2.713482	-4.883379	-1.893972
C	1.899510	-3.086189	-1.030292
H	0.976449	-3.124292	-1.625837
H	2.595168	-2.454405	-1.595698
C	2.889863	-2.452940	1.197495
H	3.648079	-1.814799	0.722304
H	2.693851	-2.033346	2.197657
C	1.469026	-5.406440	-0.194792
H	0.547476	-5.468559	-0.797276
H	1.865382	-6.431838	-0.104333
P	0.776326	-0.796353	0.158251
Pd	-0.990034	-0.984421	-1.388711
Br	0.300335	-0.945794	-3.646331
C	-3.272161	0.011127	0.324119
C	-3.195509	1.206197	-0.407203
C	-4.251447	-0.100604	1.315435
C	-4.067596	2.252118	-0.157058
H	-2.431951	1.315177	-1.182385
C	-5.136015	0.940402	1.581957
H	-4.314064	-1.028223	1.888524
H	-4.010211	3.183250	-0.723220
H	-5.884206	0.818584	2.364649
C	-2.331191	-1.135816	0.089875
O	-2.474583	-2.174933	0.699707
H	-2.418687	-0.989182	-2.373675
H	-2.390214	-1.872990	-2.432506
N	-2.804995	-3.370706	-2.848408
C	-1.760413	-4.274095	-2.394024
H	-0.807543	-3.990625	-2.862581
H	-1.651581	-4.186068	-1.303636
H	-1.982053	-5.329287	-2.644809
C	-2.916289	-3.322015	-4.296596
H	-3.646128	-2.550511	-4.582995
H	-1.943111	-3.047268	-4.726575
H	-3.243110	-4.289506	-4.723822
C	-4.078655	-3.603254	-2.189767
H	-3.939510	-3.572546	-1.100233
H	-4.788479	-2.810284	-2.469192
H	-4.520793	-4.579972	-2.464966
C	-5.045894	2.127773	0.842647
O	-5.845488	3.199535	1.021620
C	-6.848638	3.140936	2.016404
H	-6.415333	3.014604	3.022350
H	-7.378345	4.100683	1.975194
H	-7.567359	2.328484	1.820162

ts-s3-ome.log

SCF (RwB97XD) = -4615.58695447
E(SCF)+ZPE(0 K)= -4614.717043
H(298 K)= -4614.672986
G(298 K)= -4614.794392
Lowest Frequency = -837.3638cm-1

C	2.106558	-0.036285	1.988234
H	1.690288	-0.894057	2.535313
H	3.201530	-0.111894	2.079874
C	1.601798	1.233406	2.669876
H	1.982399	2.132735	2.163485
H	0.502263	1.281625	2.576109
C	2.007220	1.308906	4.141704
H	1.734709	0.368019	4.651440
H	3.106995	1.370489	4.207093
C	1.381802	2.489825	4.872930
H	0.281107	2.425946	4.870384
H	1.655378	3.444926	4.396756
H	1.708633	2.535223	5.922732
C	2.471269	1.069338	-0.709312
C	1.523701	2.298939	-0.678469
H	1.307745	2.598512	0.357485
H	0.563914	2.024281	-1.139421
C	2.140895	3.483030	-1.437391
H	1.449251	4.338570	-1.378078
C	3.489052	3.860041	-0.809151
H	3.347162	4.164455	0.242046
H	3.927616	4.723712	-1.336792
C	4.439244	2.657271	-0.884506
H	5.408172	2.920565	-0.429969
C	4.646934	2.255375	-2.350306
H	5.106845	3.086586	-2.910812
H	5.342868	1.401515	-2.413076
C	3.293573	1.881567	-2.968240
H	3.434637	1.576118	-4.017329
C	2.688399	0.698910	-2.195159
H	1.729458	0.404611	-2.647355
H	3.372935	-0.156959	-2.279468
C	2.347750	3.086603	-2.904325
H	2.770573	3.931919	-3.473252
H	1.380209	2.833852	-3.366980

C	3.833708	1.478743	-0.103365
H	4.532972	0.629390	-0.117518
H	3.718275	1.779852	0.948370
C	2.333831	-1.986220	-0.146772
C	1.821062	-2.988226	0.922364
H	2.189297	-2.711112	1.921358
H	0.719175	-2.964160	0.958949
C	2.303731	-4.414628	0.614596
H	1.921556	-5.089552	1.397449
C	3.838058	-4.437600	0.615421
H	4.221739	-4.144114	1.607389
H	4.203149	-5.459338	0.418444
C	4.358709	-3.473265	-0.457867
H	5.460717	-3.480580	-0.461441
C	3.833651	-3.904675	-1.834351
H	4.204513	-4.913518	-2.082016
H	4.212812	-3.222488	-2.613780
C	2.298675	-3.891767	-1.824188
H	1.917985	-4.190276	-2.813712
C	1.796137	-2.474711	-1.514849
H	0.695261	-2.461400	-1.504399
H	2.102139	-1.797883	-2.321585
C	3.875951	-2.044644	-0.152445
H	4.284113	-1.362341	-0.911594
H	4.275958	-1.718828	0.821961
C	1.780479	-4.859960	-0.755350
H	0.678096	-4.868084	-0.756401
H	2.113521	-5.888657	-0.973503
P	1.598219	-0.300718	0.236546
Pd	-0.830420	-0.479113	0.054470
Br	-1.103473	0.070656	-2.376287
C	-3.814945	0.221234	0.046518
C	-3.432387	1.569677	0.059739
C	-5.173956	-0.086149	0.156800
C	-4.375311	2.577042	0.185320
H	-2.375153	1.826430	-0.039029
C	-6.135714	0.911990	0.290524
H	-5.477394	-1.135606	0.141218
H	-4.081614	3.628201	0.186155
H	-7.186070	0.633679	0.375251
C	-2.807941	-0.889899	-0.035568
O	-3.156177	-2.045840	-0.107832
H	-0.733256	-1.048864	1.608247
H	-1.429474	-0.580031	2.015903
N	-2.204410	-0.333718	3.312003
C	-3.356248	-1.227208	3.317928
H	-4.091026	-0.888737	2.577526
H	-3.033674	-2.242523	3.047094
H	-3.840660	-1.260728	4.310637
C	-2.570072	1.067796	3.465898
H	-1.694007	1.700920	3.262491
H	-3.354272	1.326577	2.743652
H	-2.937613	1.287566	4.485146
C	-1.201383	-0.751671	4.280429
H	-0.841139	-1.759696	4.025352
H	-0.350432	-0.060483	4.249929
H	-1.602326	-0.770648	5.309315
C	-5.736350	2.255543	0.304392
O	-6.582726	3.301552	0.426826
C	-7.968864	3.043881	0.520471
H	-8.460461	4.021874	0.595646
H	-8.345768	2.522343	-0.374639
H	-8.213547	2.452927	1.418776

ts-s4-ome.log

SCF (RwB97XD) = -4441.20506366
E(SCF)+ZPE(0 K)= -4440.459519
H(298 K)= -4440.422126
G(298 K)= -4440.530198
Lowest Frequency = -1131.4536cm-1

C	2.325196	0.077299	1.980252
H	2.004903	-0.795603	2.565947
H	3.426043	0.077182	1.997832
C	1.777620	1.327945	2.666689
H	2.200354	2.236639	2.210259
H	0.686062	1.387544	2.511746
C	2.074209	1.346435	4.165656
H	1.623076	0.454279	4.633934
H	3.163085	1.253442	4.321306
C	1.562990	2.603134	4.858594
H	0.471979	2.707032	4.744156
H	2.027234	3.509378	4.437297
H	1.783723	2.587088	5.936607
C	2.425539	1.115022	-0.775653
C	1.414110	2.289536	-0.690773
H	1.273692	2.604388	0.353929
H	0.434984	1.951949	-1.061991
C	1.898301	3.483863	-1.526237
H	1.163572	4.299198	-1.430654
C	3.267006	3.954050	-1.016833
H	3.190377	4.281401	0.034058
H	3.611651	4.824604	-1.599489
C	4.276834	2.805131	-1.138500
H	5.260790	3.134121	-0.766837
C	4.393654	2.377022	-2.606745
H	4.758641	3.218540	-3.218953
H	5.131862	1.563200	-2.706282
C	3.021006	1.909456	-3.107724
H	3.098593	1.584648	-4.157385
C	2.551789	0.713969	-2.263376
H	1.582797	0.347347	-2.633265
H	3.282756	-0.099327	-2.377500
C	2.009949	3.056661	-2.994841
H	2.330509	3.908432	-3.617895
H	1.025668	2.731137	-3.368202
C	3.804150	1.616057	-0.284480
H	4.549555	0.808064	-0.333119
H	3.749811	1.940082	0.765021
C	2.498606	-1.933205	-0.118539
C	2.104918	-2.924207	1.008841
H	2.520102	-2.599634	1.974276
H	1.007403	-2.952748	1.116049
C	2.641785	-4.332619	0.709065
H	2.344001	-5.000793	1.532736
C	4.172564	-4.275797	0.614536
H	4.599530	-3.933591	1.572444
H	4.578885	-5.282685	0.422793
C	4.576002	-3.319803	-0.515569
H	5.674413	-3.270443	-0.586800
C	3.991873	-3.820480	-1.843969
H	4.399619	-4.816396	-2.084934
H	4.285568	-3.145816	-2.665672
C	2.461945	-3.885560	-1.737490
H	2.036893	-4.231705	-2.692709
C	1.905309	-2.486715	-1.437373
H	0.808111	-2.527782	-1.362284
H	2.128713	-1.820223	-2.278910
C	4.038853	-1.908293	-0.221249
H	4.359131	-1.229808	-1.025059
H	4.481343	-1.530944	0.715214
C	2.060858	-4.845825	-0.612919
H	0.962402	-4.911199	-0.546242
H	2.434501	-5.862034	-0.822830
P	1.728659	-0.272181	0.275593
Pd	-0.629456	-0.475565	0.301346
Br	-1.143554	-0.250105	-2.164176
C	-3.695823	0.246385	0.458996
C	-3.264717	1.560058	0.695426
C	-5.039246	0.019394	0.141364
C	-4.152871	2.618087	0.624281
H	-2.214281	1.749396	0.933193
C	-5.941584	1.072643	0.059482
H	-5.374543	-1.001956	-0.050530
H	-3.827548	3.643496	0.807018
H	-6.980520	0.866890	-0.196305
C	-2.762364	-0.897118	0.539422
O	-3.071075	-2.054570	0.482056
H	-0.515253	-0.606279	1.882980

H	-1.605616	-0.630472	1.585343
C	-5.500033	2.382068	0.303948
O	-6.292918	3.469712	0.256396
C	-7.661389	3.302622	-0.062554
H	-8.104295	4.305870	-0.047759
H	-7.794033	2.868156	-1.066631
H	-8.175570	2.673848	0.682665

ts-s5-ome.log

SCF (RwB97XD) = -4615.58337409
E(SCF)+ZPE(0 K)= -4614.707093
H(298 K)= -4614.663491
G(298 K)= -4614.784069
Lowest Frequency = -170.2786cm-1

C	2.279417	0.053263	2.069204
H	1.759988	-0.730280	2.639847
H	3.349339	-0.210186	2.093199
C	2.060794	1.381193	2.789897
H	2.646685	2.183565	2.318100
H	1.006274	1.693060	2.682292
C	2.439464	1.319017	4.269950
H	1.909289	0.480746	4.757283
H	3.511784	1.073599	4.352222
C	2.149636	2.613510	5.018624
H	1.073521	2.854016	5.005360
H	2.680192	3.465118	4.563827
H	2.461842	2.551828	6.072138
C	2.645584	1.147816	-0.616475
C	1.886055	2.495532	-0.485734
H	1.810125	2.797407	0.569357
H	0.860618	2.364251	-0.862520
C	2.593481	3.604045	-1.278762
H	2.034030	4.544606	-1.146839
C	4.028730	3.776587	-0.763240
H	4.018381	4.070906	0.300727
H	4.539395	4.584248	-1.314693
C	4.792026	2.456596	-0.935101
H	5.823286	2.572791	-0.562960
C	4.819597	2.070644	-2.420104
H	5.342650	2.847251	-3.003641
H	5.382458	1.131240	-2.556338
C	3.381135	1.901347	-2.925933
H	3.391039	1.608368	-3.988068
C	2.686928	0.792225	-2.120645
H	1.661781	0.642242	-2.492448
H	3.242232	-0.144077	-2.274817
C	2.616302	3.220147	-2.762934
H	3.096961	4.015473	-3.357942
H	1.585745	3.104444	-3.134402
C	4.095884	1.350662	-0.122095
H	4.671170	0.418002	-0.209689
H	4.113128	1.634323	0.941078
C	2.190571	-1.871780	-0.078650
C	1.660922	-2.824237	1.025696
H	2.130523	-2.590923	1.993508
H	0.573281	-2.682482	1.141224
C	1.973332	-4.290800	0.690140
H	1.579166	-4.928484	1.498475
C	3.494526	-4.467533	0.588406
H	3.972103	-4.219412	1.551910
H	3.742626	-5.519593	0.368552
C	4.035590	-3.554168	-0.518710
H	5.129289	-3.668481	-0.593262
C	3.382856	-3.930692	-1.856292
H	3.635754	-4.971390	-2.121313
H	3.775470	-3.289236	-2.663178
C	1.861099	-3.763759	-1.745506
H	1.387027	-4.019811	-2.705956
C	1.523370	-2.304763	-1.409047
H	0.430510	-2.185619	-1.328093
H	1.841085	-1.656399	-2.234789
C	3.714680	-2.084750	-0.190528
H	4.134176	-1.449062	-0.982867
H	4.213786	-1.798647	0.750265
C	1.317651	-4.676293	-0.640725
H	0.222817	-4.568515	-0.571007
H	1.527063	-5.733707	-0.875747
P	1.629881	-0.115408	0.344100
Pd	-0.706175	-0.170251	-0.099402
Br	-1.128806	1.058460	-2.382413
C	-3.842619	-0.190601	-0.030905
C	-3.749045	1.193919	0.164239
C	-5.107916	-0.777306	-0.036787
C	-4.884156	1.965166	0.360619
H	-2.763921	1.667339	0.124009
C	-6.261103	-0.020273	0.169538
H	-5.186702	-1.854340	-0.202284
H	-4.817482	3.047957	0.484900
H	-7.232897	-0.513718	0.163245
C	-2.623074	-1.061665	-0.179707
O	-2.714741	-2.176382	-0.665487
H	-1.767563	-1.008105	0.872250
H	-1.755615	0.266291	2.128640
N	-2.052411	0.435116	3.115477
C	-3.382952	-0.189953	3.305937
H	-4.122774	0.338207	2.695399
H	-3.326312	-1.238416	2.990107
H	-3.650327	-0.129675	4.368479
C	-2.081243	1.899893	3.324107
H	-1.109180	2.315709	3.035302
H	-2.865383	2.331732	2.692652
H	-2.288370	2.111427	4.380525
C	-1.023798	-0.230668	3.946190
H	-0.983450	-1.291447	3.672950
H	-0.056955	0.235307	3.740607
H	-1.285308	-0.116414	5.005480
C	-6.151936	1.360888	0.370988
O	-7.202247	2.187978	0.579222
C	-8.507219	1.651882	0.501858
H	-9.195898	2.492900	0.651153
H	-8.702573	1.200649	-0.484808
H	-8.688089	0.901292	1.289685

ts-s6-ome.log

SCF (RwB97XD) = -1866.35426179
E(SCF)+ZPE(0 K)= -1865.616764
H(298 K)= -1865.582323
G(298 K)= -1865.681516
Lowest Frequency = -473.1318cm-1

C	1.099109	0.197437	1.381324
H	0.503868	-0.614698	1.822835
H	2.061425	0.211726	1.919297
C	0.332195	1.501310	1.604914
H	0.972591	2.368385	1.376885
H	-0.521428	1.554613	0.910081
C	-0.189753	1.631240	3.035018
H	-0.868063	0.786257	3.244531
H	0.651006	1.535251	3.744614
C	-0.924723	2.942571	3.279309
H	-1.765719	3.056192	2.577275
H	-0.257720	3.809078	3.140976
H	-1.331000	2.993592	4.301151
C	2.414154	0.986164	-1.151457
C	1.449375	2.116807	-1.599561
H	0.899020	2.522805	-0.739641
H	0.693676	1.703478	-2.285106
C	2.220382	3.249200	-2.293303
H	1.505908	4.037162	-2.579457
C	3.272196	3.822362	-1.333871
H	2.781659	4.240695	-0.438638
H	3.817894	4.649740	-1.817464
C	4.250187	2.711808	-0.927247
H	5.006835	3.117755	-0.236797
C	4.939461	2.148913	-2.177625
H	5.519205	2.940607	-2.680803
H	5.654908	1.359223	-1.891504

C	3.879622	1.583041	-3.133173
H	4.369904	1.164347	-4.026619
C	3.109374	0.456677	-2.425831
H	2.356408	0.028352	-3.108676
H	3.818106	-0.345738	-2.175769
C	2.911420	2.697685	-3.546130
H	3.457651	3.501036	-4.068439
H	2.159649	2.306014	-4.250831
C	3.481157	1.588104	-0.211877
H	4.186606	0.812829	0.121140
H	3.007492	1.998333	0.692877
C	2.276202	-1.959609	-0.185611
C	1.502812	-2.835892	0.834309
H	1.533252	-2.376957	1.833255
H	0.440201	-2.905032	0.540813
C	2.120706	-4.239235	0.933246
H	1.543715	-4.825558	1.666049
C	3.576305	-4.111198	1.403183
H	3.613419	-3.641232	2.400239
H	4.033363	-5.109793	1.501362
C	4.362834	-3.266685	0.392422
H	5.408524	-3.166377	0.724727
C	4.321816	-3.945040	-0.984699
H	4.802246	-4.936261	-0.933000
H	4.893015	-3.349710	-1.716952
C	2.863561	-4.087659	-1.444788
H	2.832041	-4.566600	-2.436280
C	2.224487	-2.695749	-1.548559
H	1.172565	-2.793561	-1.876328
H	2.737980	-2.116499	-2.326732
C	3.740646	-1.862968	0.287164
H	4.335530	-1.263214	-0.416737
H	3.790808	-1.355350	1.264790
C	2.080201	-4.931985	-0.433562
H	1.036692	-5.051689	-0.769140
H	2.513732	-5.943313	-0.359945
P	1.352600	-0.335875	-0.360607
Pd	-0.701731	-0.808215	-1.464686
C	-2.855921	0.824930	-0.263710
C	-2.959200	-0.156807	0.730494
C	-3.444679	2.071282	-0.037870
C	-3.620498	0.102105	1.919704
H	-2.502806	-1.135790	0.557663
C	-4.112794	2.349127	1.152055
H	-3.366395	2.839323	-0.810462
H	-3.703276	-0.658599	2.698086
H	-4.554506	3.334542	1.298107
C	-2.076952	0.608823	-1.529498
O	-2.104965	1.401516	-2.439187
H	-2.184059	-1.097533	-2.044858
C	-4.195558	1.362862	2.143838
O	-4.798316	1.534117	3.340555
C	-5.398663	2.782260	3.621887
H	-5.818297	2.701530	4.632398
H	-4.659187	3.599960	3.607083
H	-6.212932	3.010076	2.914285

ts-s7-ome.log

SCF (RwB97XD) = -4615.57606479
E(SCF)+ZPE(0 K)= -4614.705515
H(298 K)= -4614.662046
G(298 K)= -4614.781406
Lowest Frequency = -1404.9236cm-1

C	2.423675	0.131187	2.035278
H	2.136231	-0.731629	2.651433
H	3.523214	0.130265	1.988265
C	1.907741	1.394461	2.725044
H	2.394374	2.289403	2.305888
H	0.830443	1.511525	2.517322
C	2.128329	1.372567	4.236613
H	1.637688	0.477946	4.660031
H	3.205011	1.252306	4.447663
C	1.602292	2.621271	4.932745
H	0.519581	2.745212	4.766300
H	2.098076	3.529154	4.553633
H	1.770145	2.580402	6.019664
C	2.369651	1.122462	-0.728746
C	1.393205	2.321749	-0.595999
H	1.333321	2.655082	0.450578
H	0.384252	2.001890	-0.894205
C	1.848556	3.493017	-1.478946
H	1.136952	4.324418	-1.351650
C	3.254717	3.941767	-1.060919
H	3.247912	4.285168	-0.012235
H	3.582030	4.796143	-1.676774
C	4.230755	2.769098	-1.225774
H	5.242046	3.080908	-0.917702
C	4.251084	2.320794	-2.692537
H	4.595785	3.147450	-3.336314
H	4.965763	1.490718	-2.825038
C	2.841783	1.875732	-3.103292
H	2.848471	1.536037	-4.151220
C	2.399436	0.701606	-2.215866
H	1.401839	0.352996	-2.520466
H	3.105456	-0.128495	-2.362047
C	1.862212	3.044704	-2.945461
H	2.161081	3.882456	-3.598004
H	0.851439	2.733550	-3.253813
C	3.785056	1.601736	-0.327540
H	4.511553	0.779200	-0.405702
H	3.798499	1.944332	0.717225
C	2.434943	-1.916490	-0.038050
C	2.109064	-2.892824	1.123233
H	2.585583	-2.558869	2.056974
H	1.020971	-2.911202	1.297116
C	2.615945	-4.308276	0.805975
H	2.368260	-4.965273	1.655174
C	4.137140	-4.266946	0.608882
H	4.630872	-3.919065	1.532170
H	4.521538	-5.279352	0.400954
C	4.471092	-3.325942	-0.555775
H	5.562699	-3.286653	-0.700631
C	3.795883	-3.835395	-1.836891
H	4.179111	-4.837286	-2.093677
H	4.039642	-3.171732	-2.683614
C	2.275859	-3.885898	-1.628436
H	1.786343	-4.239819	-2.549270
C	1.750876	-2.479239	-1.308513
H	0.660783	-2.508408	-1.159361
H	1.922663	-1.823417	-2.170138
C	3.965427	-1.907276	-0.241515
H	4.238933	-1.240064	-1.071373
H	4.471248	-1.524572	0.660213
C	1.943451	-4.830549	-0.468437
H	0.851411	-4.884317	-0.327619
H	2.293034	-5.852684	-0.691478
P	1.706655	-0.241103	0.381392
Pd	-0.598414	-0.439435	0.529224
Br	-1.257773	-0.076712	-1.941354
C	-3.696003	0.265590	0.501369
C	-3.309151	1.585019	0.768727
C	-4.995488	0.024509	0.056068
C	-4.196133	2.635556	0.602450
H	-2.285163	1.782738	1.100626
C	-5.899056	1.067784	-0.128014
H	-5.296290	-1.004243	-0.154647
H	-3.899653	3.666943	0.802009
H	-6.904208	0.847537	-0.487034
C	-2.743498	-0.863769	0.734629
O	-3.094457	-2.026265	0.629380
H	-0.118937	-0.727945	1.956356
H	-2.032509	-0.606623	1.967882
N	-2.396941	-0.669808	3.372284
C	-3.843568	-0.639755	3.585612
H	-4.244142	0.332247	3.272646
H	-4.318519	-1.426053	2.983504
H	-4.088427	-0.806201	4.648544
C	-1.725302	0.455693	4.011504

H	-0.657987	0.427191	3.756027
H	-2.150196	1.397712	3.640281
H	-1.831190	0.419656	5.109017
C	-1.825073	-1.950762	3.777552
H	-2.327300	-2.759260	3.230797
H	-0.756812	-1.968615	3.520112
H	-1.936464	-2.114823	4.862562

C	-5.499700	2.383103	0.146420
O	-6.295204	3.467017	0.004060
C	-7.587733	3.291655	-0.539415
H	-8.033776	4.292093	-0.603812
H	-7.545394	2.854087	-1.550476
H	-8.220768	2.660675	0.106473

5. References

(1) Sergeev, A. G.; Spannenberg, A.; Beller, M. Palladium-Catalyzed Formylation of Aryl Bromides: Elucidation of the Catalytic Cycle of an Industrially Applied Coupling Reaction. *J. Am. Chem. Soc.* **2008**, *130*, 15549–15563.
(2) Auburn, P. R.; Mackenzie, P. B.; Bosnich, B. Asymmetric synthesis. Asymmetric catalytic allylation using palladium chiral phosphine complexes. *J. Am. Chem. Soc.* **1985**, *107* (7), 2033-2046.
(3) Garçon, M.; White, A. J. P.; Crimmin, M. R. Palladium-catalysed magnesiation of benzene. *Chem. Commun.* **2018**, *54* (87), 12326-12328.
(4) Zhang, G.; Hanson, S. K. Cobalt-Catalyzed Acceptorless Alcohol Dehydrogenation: Synthesis of Imines from Alcohols and Amines. *Org. Lett.* **2013**, *15* (3), 650-653.
(5) Natte, K.; Dumrath, A.; Neumann, H.; Beller, M. Palladium-Catalyzed Carbonylations of Aryl Bromides using Paraformaldehyde: Synthesis of Aldehydes and Esters. *Angew. Chem. Int. Ed.* **2014**, *53*, 10090 – 10094.
(6) Burés, J. Variable Time Normalization Analysis: General Graphical Elucidation of Reaction Orders from Concentration Profiles. *Angew. Chem. Int. Ed.* **2016**, *55* (52), 16084-16087.
(7) Nielsen, C. D. T.; Burés, J. Visual kinetic analysis. *Chem. Sci.* **2019**, *10* (2), 348-353.
(8) Blackmond, D. G. Reaction Progress Kinetic Analysis: A Powerful Methodology for Mechanistic Studies of Complex Catalytic Reactions. *Angew. Chem. Int. Ed.* **2005**, *44* (28), 4302-4320.
(9) Frisch, M. J.; Trucks, G. W.; Schlegel, H. B.; Scuseria, G. E.; Robb, M. A.; Cheeseman, J. R.; Scalmani, G.; Barone, V.; Mennucci, B.; Petersson, G. A.; Nakatsuji, H.; Caricato, M.; Li, X.; Hratchian, H. P.; Izmaylov, A. F.; Bloino, J.; Zheng, G.; Sonnenberg, J. L.; Hada, M.; Ehara, M.; Toyota, K.; Fukuda, R.; Hasegawa, J.; Ishida, M.; Nakajima, T.; Honda, Y.; Kitao, O.; Nakai, H.; Vreven, T.; Montgomery, J. A., Jr.; Peralta, J. E.; Ogliaro, F.; Bearpark, M.; Heyd, J. J.; Brothers, E.; Kudin, K. N.; Staroverov, V. N.; Kobayashi, R.; Normand, J.; Raghavachari, K.; Rendell, A.; Burant, J. C.; Iyengar, S. S.; Tomasi, J.; Cossi, M.; Rega, N.; Millam, J. M.; Klene, M.; Knox, J. E.; Cross, J. B.; Bakken, V.; Adamo, C.; Jaramillo, J.; Gomperts, R.; Stratmann, R. E.; Yazyev, O.; Austin, A. J.; Cammi, R.; Pomelli, C.; Ochterski, J. W.; Martin, R. L.; Morokuma, K.; Zakrzewski, V. G.; Voth, G. A.; Salvador, P.; Dannenberg, J. J.; Dapprich, S.; Daniels, A. D.; Farkas, Ö.; Foresman, J. B.; Ortiz, J. V.; Cioslowski, J.; Fox, D. J. Gaussian 09, Revision D.01; Gaussian Inc., Wallingford, CT, **2009**.
(10) Chai, J.-D.; Head-Gordon, M. Long-range corrected hybrid density functionals with damped atom–atom dispersion corrections. *Phys. Chem. Chem. Phys.* **2008**, *10* (44), 6615-6620.
(11) Perdew, J. P.; Chevary, J. A.; Vosko, S. H.; Jackson, K. A.; Pederson, M. R.; Singh, D. J.; Fiolhais, C. Atoms, molecules, solids, and surfaces: Applications of the generalized gradient approximation for exchange and correlation. *Phys. Rev. B* **1992**, *46* (11), 6671-6687.
(12) Perdew, J. P.; Chevary, J. A.; Vosko, S. H.; Jackson, K. A.; Pederson, M. R.; Singh, D. J.; Fiolhais, C. Erratum: Atoms, molecules, solids, and surfaces: Applications of the generalized gradient approximation for exchange and correlation. *Phys. Rev. B* **1993**, *48* (7), 4978-4978.
(13) Adamo, C.; Cossi, M.; Barone, V. An accurate density functional method for the study of magnetic properties: the PBE0 model. *J. Mol. Struct. Theochem* **1999**, *493* (1), 145-157.
(14) Zhao, Y.; Truhlar, D. G. A new local density functional for main-group thermochemistry, transition metal bonding, thermochemical kinetics, and noncovalent interactions. *J. Chem. Phys.* **2006**, *125* (19), 194101.
(15) Zhao, Y.; Truhlar, D. G. The M06 suite of density functionals for main group thermochemistry, thermochemical kinetics, noncovalent interactions, excited states, and transition elements: two new functionals and systematic testing of four M06-class functionals and 12 other functionals. *Theor. Chem. Acc.* **2008**, *120* (1), 215-241.
(16) Grimme, S.; Antony, J.; Ehrlich, S.; Krieg, H. A consistent and accurate ab initio parametrization of density functional dispersion correction (DFT-D) for the 94 elements H-Pu. *J. Chem. Phys.* **2010**, *132* (15), 154104.

(17) Becke, A. D.; Johnson, E. R. A density-functional model of the dispersion interaction. *J. Chem. Phys.* **2005**, *123* (15), 154101.
(18) Johnson, E. R.; Becke, A. D. A post-Hartree–Fock model of intermolecular interactions. *J. Chem. Phys.* **2005**, *123* (2), 024101.
(19) Johnson, E. R.; Becke, A. D. A post-Hartree-Fock model of intermolecular interactions: Inclusion of higher-order corrections. *J. Chem. Phys.* **2006**, *124* (17), 174104.
(20) Grimme, S.; Ehrlich, S.; Goerigk, L. Effect of the damping function in dispersion corrected density functional theory. *J. Comput. Chem.* **2011**, *32* (7), 1456-1465.
(21) Andrae, D.; Häußermann, U.; Dolg, M.; Stoll, H.; Preuß, H. Energy-adjustedab initio pseudopotentials for the second and third row transition elements. *Theor. Chim. Acta* **1990**, *77* (2), 123-141.
(22) Weigend, F.; Ahlrichs, R. Balanced basis sets of split valence, triple zeta valence and quadruple zeta valence quality for H to Rn: Design and assessment of accuracy. *Phys. Chem. Chem. Phys.* **2005**, *7* (18), 3297-3305.
(23) Feller, D. The role of databases in support of computational chemistry calculations. *J. Comput. Chem.* **1996**, *17* (13), 1571-1586.
(24) Schuchardt, K. L.; Didier, B. T.; Elsethagen, T.; Sun, L.; Gurumoorthi, V.; Chase, J.; Li, J.; Windus, T. L. Basis Set Exchange: A Community Database for Computational Sciences. *J. Chem. Inf. Model.* **2007**, *47* (3), 1045-1052.
(25) Pritchard, B. P.; Altarawy, D.; Didier, B.; Gibson, T. D.; Windus, T. L. New Basis Set Exchange: An Open, Up-to-Date Resource for the Molecular Sciences Community. *J. Chem. Inf. Model.* **2019**, *59* (11), 4814-4820.
(26) Marenich, A. V.; Cramer, C. J.; Truhlar, D. G. Universal Solvation Model Based on Solute Electron Density and on a Continuum Model of the Solvent Defined by the Bulk Dielectric Constant and Atomic Surface Tensions. *J. Phys. Chem. B* **2009**, *113* (18), 6378-6396.
(27) Dennington, R.; Keith, T.; Milliam, J. GaussView 6.0; Semichem Inc., Shawnee Mission, KS, **2019**.
(28) Pracht, P.; Bohle, F.; Grimme, S. Automated exploration of the low-energy chemical space with fast quantum chemical methods. *Phys. Chem. Chem. Phys.* **2020**, *22* (14), 7169-7192.
(29) Grimme, S. Exploration of Chemical Compound, Conformer, and Reaction Space with Meta-Dynamics Simulations Based on Tight-Binding Quantum Chemical Calculations. *J. Chem. Theory Comput.* **2019**, *15* (5), 2847-2862.
(30) Grimme, S.; Bohle, F.; Hansen, A.; Pracht, P.; Spicher, S.; Stahn, M. Efficient Quantum Chemical Calculation of Structure Ensembles and Free Energies for Nonrigid Molecules. *J. Phys. Chem. A* **2021**, *125* (19), 4039-4054.
(31) Luchini, G.; Alegre-Requena, J. V.; Funes-Ardoiz, I.; R.S., P. GoodVibes: automated thermochemistry for heterogeneous computational chemistry data. *F1000Research* **2020**, *9*, 291.
(32) Jáuregui-Haza, U. J.; Pardillo-Fontdevila, E. J.; Wilhelm, A. M.; Delmas, H. *Lat. Am. Appl. Res.* **2004**, *34*, 71–74.
(33) Neese, F. The ORCA program system. *WIREs Comput. Mol. Sci.* **2012**, *2* (1), 73-78.
(34) Neese, F. Software update: The ORCA program system—Version 5.0. *WIREs Comput. Mol. Sci.* **2022**, *12* (5), e1606.
(35) Caldeweyher, E.; Bannwarth, C.; Grimme, S. Extension of the D3 dispersion coefficient model. *J. Chem. Phys.* **2017**, *147* (3), 034112.
(36) Caldeweyher, E.; Ehlert, S.; Hansen, A.; Neugebauer, H.; Spicher, S.; Bannwarth, C.; Grimme, S. A generally applicable atomic-charge dependent London dispersion correction. *J. Chem. Phys.* **2019**, *150* (15), 154122.
(37) NBO 6.0. Glendening, E. D.; Badenhoop, J. K.; Reed, A. E.; Carpenter, J. E.; Bohmann, J. A.; Morales, C. M.; Landis, C. R.; Weinhold, F. Theoretical Chemistry Institute, University of Wisconsin, Madison (2013).
(38) Glendening, E. D.; Landis, C. R.; Weinhold, F. NBO 6.0: Natural bond orbital analysis program. *J. Comput. Chem.* **2013**, *34* (16), 1429-1437.
